# Particle-based simulations of polarity establishment reveal stochastic promotion of Turing pattern formation

**DOI:** 10.1371/journal.pcbi.1006016

**Published:** 2018-03-12

**Authors:** Michael Pablo, Samuel A. Ramirez, Timothy C. Elston

**Affiliations:** 1 Department of Chemistry, The University of North Carolina, Chapel Hill, NC, United States of America; 2 Program in Molecular and Cellular Biophysics, The University of North Carolina, Chapel Hill, NC, United States of America; 3 Department of Pharmacology, The University of North Carolina, Chapel Hill, NC, United States of America; Rice University, UNITED STATES

## Abstract

Polarity establishment, the spontaneous generation of asymmetric molecular distributions, is a crucial component of many cellular functions. *Saccharomyces cerevisiae* (yeast) undergoes directed growth during budding and mating, and is an ideal model organism for studying polarization. In yeast and many other cell types, the Rho GTPase Cdc42 is the key molecular player in polarity establishment. During yeast polarization, multiple patches of Cdc42 initially form, then resolve into a single front. Because polarization relies on strong positive feedback, it is likely that the amplification of molecular-level fluctuations underlies the generation of multiple nascent patches. In the absence of spatial cues, these fluctuations may be key to driving polarization. Here we used particle-based simulations to investigate the role of stochastic effects in a Turing-type model of yeast polarity establishment. In the model, reactions take place either between two molecules on the membrane, or between a cytosolic and a membrane-bound molecule. Thus, we developed a computational platform that explicitly simulates molecules at and near the cell membrane, and implicitly handles molecules away from the membrane. To evaluate stochastic effects, we compared particle simulations to deterministic reaction-diffusion equation simulations. Defining macroscopic rate constants that are consistent with the microscopic parameters for this system is challenging, because diffusion occurs in two dimensions and particles exchange between the membrane and cytoplasm. We address this problem by empirically estimating macroscopic rate constants from appropriately designed particle-based simulations. Ultimately, we find that stochastic fluctuations speed polarity establishment and permit polarization in parameter regions predicted to be Turing stable. These effects can operate at Cdc42 abundances expected of yeast cells, and promote polarization on timescales consistent with experimental results. To our knowledge, our work represents the first particle-based simulations of a model for yeast polarization that is based on a Turing mechanism.

## Author summary

Many cells need to generate and maintain biochemical signals in specific subcellular regions. This phenomenon is broadly called polarity establishment, and is important in fundamental processes such as cell migration and differentiation. A key polarity factor found in diverse organisms, including yeast and humans, is the protein Cdc42. In yeast, Cdc42-dependent polarization occurs through a self-reinforcing biochemical signaling loop. Directional cues can guide polarity establishment, but interestingly, yeast can polarize in the absence of such a cue. The mechanism thought to underlie this symmetry breaking involves the amplification of inhomogeneities in molecular distributions that arise from molecular-level fluctuations. We investigated the effects of random fluctuations on polarization by performing particle-based simulations of the Cdc42 signaling network. We found that fluctuations can facilitate polarization, allowing faster polarization, and polarization over a broader range of concentrations. Our observations may help understand how polarity works in other systems.

## Introduction

Cell polarity refers to the localization of signaling molecules to specific regions of the plasma membrane, and is required for fundamental cellular processes such as migration, directed growth, and differentiation. In the yeast *Saccharomyces cerevisiae*, polarization is required for directed growth during budding and mating. Because of its experimental tractability, yeast represents a powerful model organism for studying polarity establishment. Normally, yeast polarization involves internal or external spatial cues such as bud scars and pheromone gradients. However, polarization still occurs if these cues are removed [1].

Mathematical models have been used to explain spontaneous pattern formation by biochemical systems since the 1950s [2,3]. These models use diffusion-driven instabilities to generate symmetry breaking without relying on mechanisms such as diffusional barriers, directed transport, and molecular cross-linking. Instead, these systems require: (1) positive feedback to amplify local fluctuations; (2) chemical species that diffuse at different rates; and (3) a mechanism for limiting the growth of the polarity site. Models in which patterning can be induced by an arbitrarily weak perturbation (e.g. molecular-level fluctuations) are called Turing-type. Goryachev and Polkhilko were the earliest to use a Turing-type model to study yeast polarization [4]. Other, non-Turing type models of polarity require perturbations of finite strength to induce pattern formation [5].

A common approach to modeling the spatiotemporal dynamics of polarizing biochemical systems is to use reaction-diffusion equations (RDEs) in the form of non-linear partial differential equations (PDEs). RDEs are deterministic and ignore stochastic effects intrinsic to chemical reactions and thermal diffusion. In some systems, stochastic effects have been shown to expand the parameter space that leads to patterning and accelerate pattern formation [6,7]. Many modeling approaches are used to study stochastic effects in biological signaling networks, including stochastic differential equations, such as chemical Langevin equations [8,9]; spatially discretized, temporally-continuous approaches, such as the spatial Gillespie algorithm [10–12]; exact Brownian dynamics, such as Green’s function reaction dynamics [13,14]; and direct particle-based simulations, as implemented in Smoldyn and MCell [15,16]. We cannot adequately cover the full spectrum of approaches and computational tools here, but refer the reader to excellent reviews that describe the theoretical underpinnings and software implementations of these methods [17–20]. In Table 1, we describe advantages and limitations for some of the more common methods. Hybrid approaches, such as the method described in [21], that mix particle simulations with a deterministic partial differential equation solver are most similar to the approach we take here.

**Table 1:** Computational approaches for simulating spatial and temporal stochasticity in biochemical reaction networks. Additional approaches not listed here are referenced via reviews in the text.

Approach	Spatially discretized?	Temporally discretized?	Comment
Stochastic partial differential equations	Yes	Yes	More efficient than particle-based simulations. Breaks down in low concentration limits.
Spatial Gillespie	Yes	No	Can be more efficient than particle-based simulations. Can suffer from artifacts due to spatial discretization.
Hybrid particle-based-PDE methods (this work)	No	Yes	Approximation of full particle-based methods by explicitly modeling only a portion of the domain of interest and implicitly modeling the remainder using either deterministic or stochastic methods.
Fully particle-based	No	Yes	More accurate than PDE and Gillespie approaches. Computationally demanding.
Exact Brownian dynamics	No	No	More accurate than particle-based simulation in terms of physical description, but can come at a higher computational demand [22]. Also called an “event-driven” approach, but this can cause confusion with Gillespie-type approaches.

**Fig 1:**
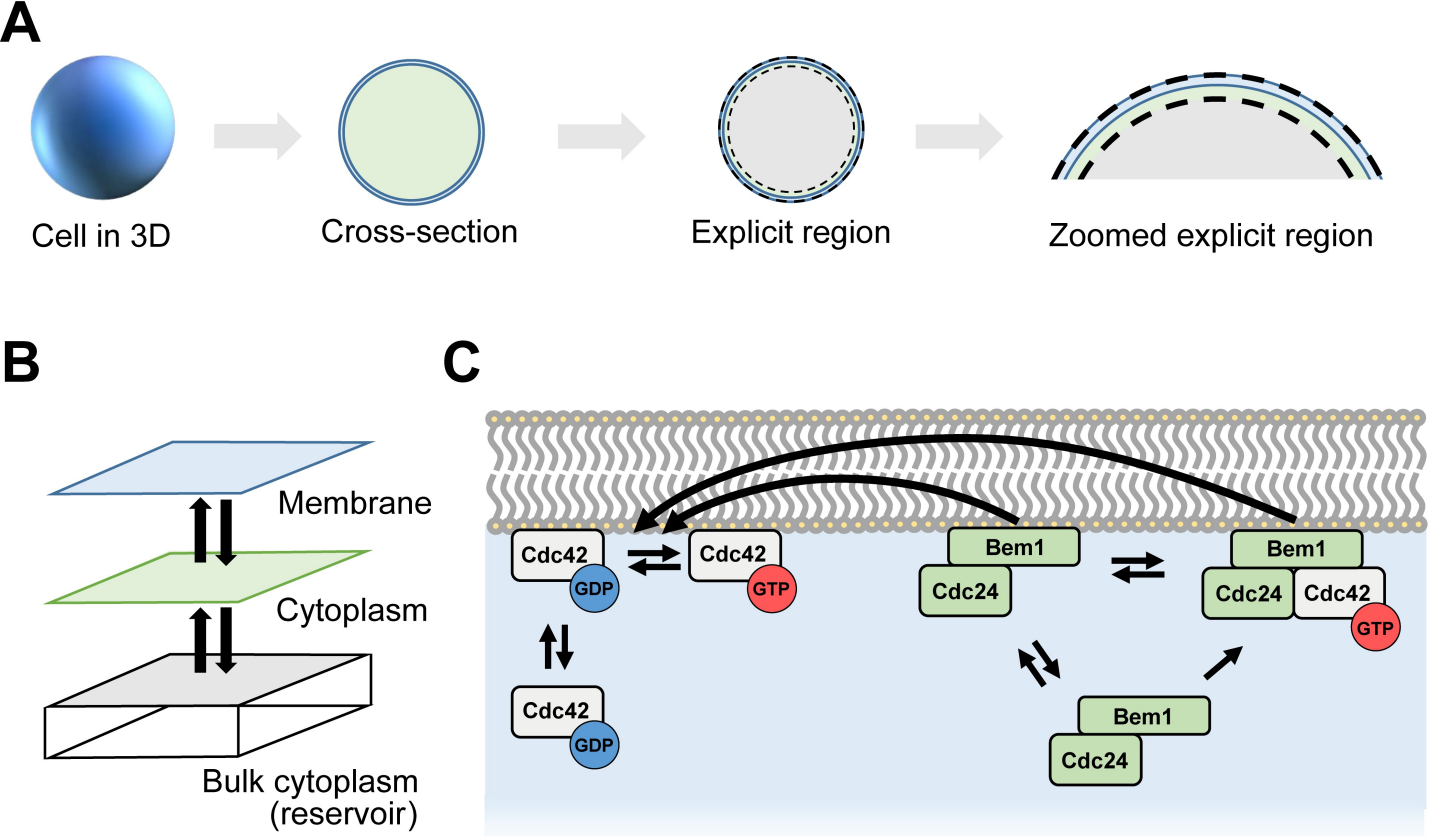
Computational modeling schematics. (**A**) Molecules at the cell membrane and a thin slice of adjacent cytoplasm are simulated explicitly. Both compartments are modeled as 2D surface. (**B**) In the quasi-3D simulations, a well-stirred compartment representing bulk cytoplasm is added to approximate 3D effects. (**C**) Reaction scheme for a Turing-type model of Cdc42-dependent yeast polarity establishment from Goryachev and Polkhilko [4].

The effects of noise in non-Turing models of yeast polarization have been investigated using a variety of stochastic methods [21,23–25]. Typically, these models make simplifying assumptions to reduce the complexity of the polarity system. Some models, such as the neutral drift polarity model, used particle-based approaches; others, like models based on wave-pinning, used Gillespie or stochastic PDE-based approaches [26,27]. Other investigations of stochasticity in polarization with more detailed signaling models, including the Turing-type Goryachev–Pokhilko model, leveraged Gillespie and stochastic PDE approaches [28,29]. Here, we present particle-based simulations of the Goryachev–Pokhilko model, and compare them to RDE simulations of the same system to evaluate stochastic effects on polarization. In this model, reactions occur between either two reactants on the membrane, or between a reactant on the membrane and a reactant in the cytoplasm. Exchange can occur between the membrane and cytoplasm. We design our simulations to explicitly track molecules at and near the cell membrane, where polarization occurs, and implicitly handle molecules away from the membrane. We consider two different scenarios. In the first, we treat the cell membrane and the nearby cytoplasm as purely two-dimensional (2D) and ignore the remaining bulk cytoplasm. In the second, we approximate the bulk cytoplasm by attaching a molecular reservoir in which we only track molecular abundances. Molecules are stochastically exchanged between the 2D particle-based domain and the reservoir with rates determined by diffusion, thus creating a quasi-three dimensional system (Fig 1).

An outline of our paper is as follows. First, we demonstrate that our particle-based simulations generate results consistent with deterministic rate equations in the well-stirred limit. We then show how deviations from this idealized behavior occur as spatial effects become important. These deviations occur when the 2D reactions become diffusion-influenced, and it is no longer possible to describe the kinetics of second-order reactions with a single macroscopic rate constant [30,31]. Interestingly, existing models of the yeast polarity system contain second-order rate constants that appear to fall within this diffusion-influenced regime, calling into question the validity of the model equations (Fig C in S1 Text). However, existing theories for computing second-order rate constants from microscopic parameters do not take into account the situation in which chemical species can transition between different diffusional states, e.g. membrane versus cytosolic. Therefore, we empirically determined second-order rate constants by fitting rate equations to results from particle-based simulations. Each potentially diffusion-influenced bimolecular reaction was simulated in combination with the relevant membrane-cytoplasm exchange reactions. Our results demonstrate that in many cases the chemical kinetics of this expanded system can be well-approximated using a single second-order rate constant. This empirical mapping between the microscopic and macroscopic regimes allows us to compare the polarization results from particle-based simulations to solutions of the corresponding RDEs.

We show that molecular fluctuations increase the rate at which polarization occurs in a purely 2D system, lacking the cytoplasmic reservoir. Polarity also occurs over a broader range of Cdc42 concentrations. These observations are consistent with previous reports in other systems where Turing patterning was enhanced due to either particle-based fluctuations [6,7] or sufficiently strong perturbations [32,33]. We also show that stochastic effects inherent to particle-based simulations can generate large scale variability in polarization dynamics and metastable multi-patch states. This is in agreement with theoretical and experimental [4,34,35] demonstrations of emergent, competing multi-polar states. Moving on to particle-based simulations with the quasi-3D molecular reservoir, we find that the particle-based simulations still exhibit enhanced polarization compared to the deterministic RDEs within parameters representative of a typical yeast cell. In the quasi-3D particle-based simulations, the resolution of multi-patch states takes place on a timescale of minutes, consistent with experimental measurements [34,35]. To our knowledge, our work represents the first particle-based simulations of a model for yeast polarization that is based on a Turing mechanism. Our simulations underscore important effects of stochasticity on polarity establishment, including more rapid competition between polarity sites and increased robustness to changes in molecular abundances.

## Results

### Formulation of the 2D particle-based simulation approach

We first considered a purely 2D computational domain representing molecules in the cell membrane and a thin volume of cytoplasm adjacent to the membrane. Molecules in the membrane or cytoplasmic layer were differentiated by their diffusivity and reactivity. We neglect the rest of the cytoplasm until later (see subsection “A quasi-3D approach to full cell simulations”). The spatial coordinates of molecules were treated as continuous variables, while time was discretized in intervals of Δ*t*. Thermal diffusion was handled using the Euler-Maruyama method [36]. First-order or unimolecular reactions were assigned probabilities of occurring in Δ*t* given by *P*_*i*_ = 1 –exp(-*k*_*i*_Δ*t*), where *k*_*i*_ was the rate constant for the *i*-th reaction. If the first-order reaction involved the dissociation of two molecules, then the two products were placed a distance of $\bar{\sigma }$ apart, with one of the molecules located at the position of the complex, and the orientation angle chosen at random from a uniform distribution. For second-order or bimolecular reactions, we assumed that two molecules react with probability *P*_*λ*_ *= λ*Δ*t* when they are within a distance $\bar{\varrho }$. Thus, if the two reactants are within a reactive range $\bar{\varrho }$, they react with an average rate *λ*. This approach is based on the Doi method [37]. It is distinct from the classic diffusion-limited Smoluchowski approach, where molecules react upon finding one another for the first time and molecular radii are adjusted to reach the desired kinetics [38].

### Connection to the macroscopic limit

Investigating the role of molecular fluctuations in polarity establishment requires a way to compare particle-based simulation results to the deterministic behavior of the system in the macroscopic limit, where the spatiotemporal dynamics of biochemical concentrations are governed by reaction-diffusion equations (RDEs). Therefore, we needed a way to relate microscopic parameters to macroscopic rate constants in two dimensions. For first-order reactions, this is trivial, and follows the relation noted above. The situation is more complicated for second-order reactions.

**Fig 2:**
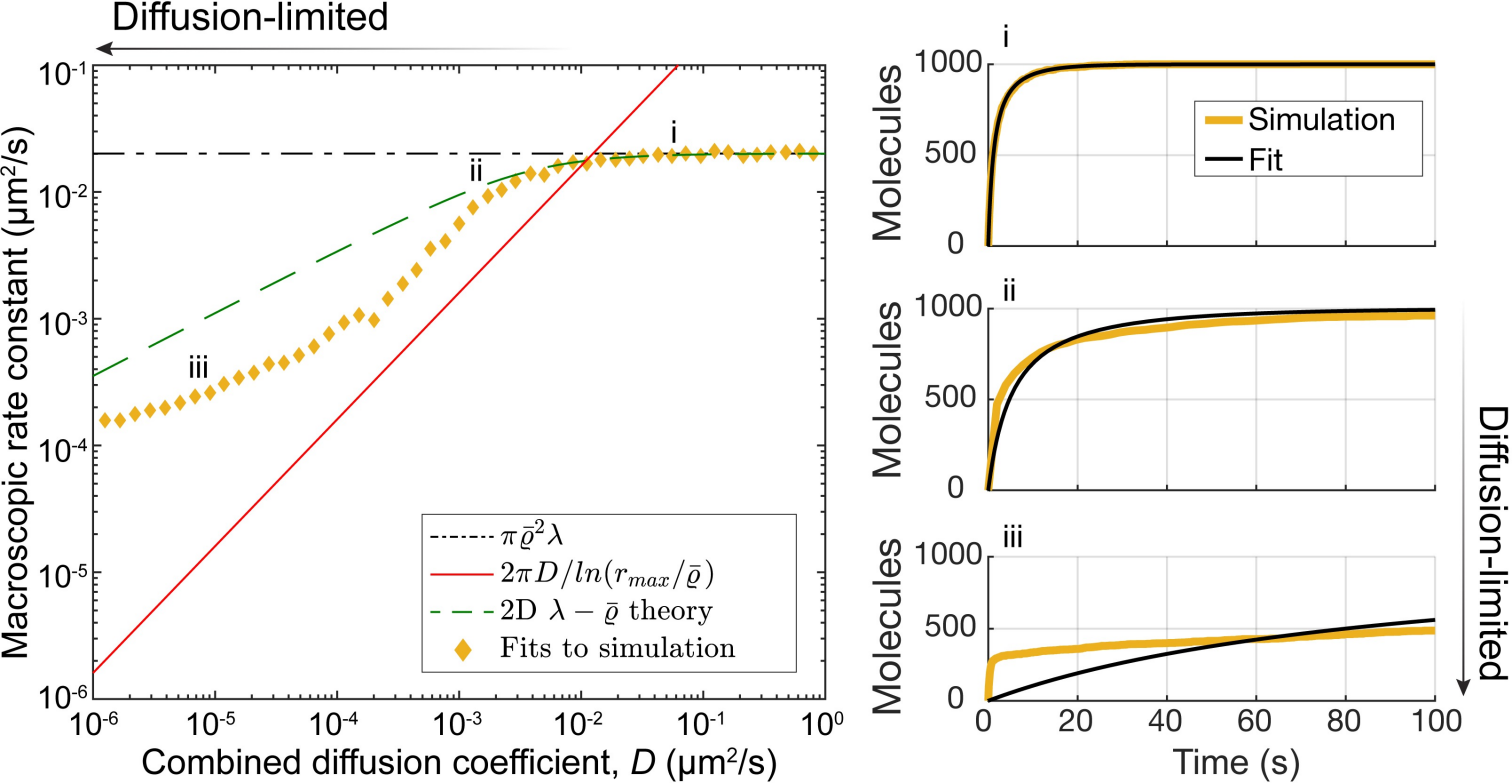
Illustration of the different reaction regimes (reaction-limited, diffusion-influenced, and diffusion-limited regimes) and the range of validity of the 2D $\lambda -\bar{\varrho }$ theory. The left panel shows estimated rate constants (yellow diamonds) for the 2D second order reaction A+B → C obtained by fitting chemical rate equations (black curves, right panels) to results from particle-based simulations (yellow curves, right panels). The reaction limit, $k_{RL}=\pi {\bar{\varrho }}^{2}\lambda$, is indicated by the black dot-dashed line and the estimate for the diffusion limit $k_{DL}=2\pi D/\ln\left(r_{max}/\bar{\varrho }\right)$ is represented by the red curve. The results from the 2D $\lambda -\bar{\varrho }$ theory are shown as the green-dashed line. Parameters chosen are *D*_A_ = *D*_B_ = ½*D* (x-axis of left panel), *λ* = 2.5554 s^-1^, *r*_max_ = 2.5 μm, $\bar{\varrho }$ = 0.05 μm. Simulations were conducted on a *L* = 5 μm domain.

In chemical kinetic theory, there are two limiting regimes for second-order reactions. The first is the diffusion limit, in which two particles react when they encounter one another for the first time. The diffusion limit represents the maximum rate at which a second-order reaction can proceed. In 3D, it is possible to define a macroscopic rate constant in the diffusion limit by considering the diffusional flux through an absorbing sphere of radius $\bar{\varrho }$ located at the origin, when the concentration *C* of the reactant is held fixed at infinity [39,40]. The flux into the sphere is given by $J=4\pi D\bar{\varrho }C$, where *D* is the sum of the diffusion coefficients of the reactants. From this expression, the second-order rate constant in the 3D diffusion limit is defined to be $k=4\pi D\bar{\varrho }$. In 2D, diffusion-limited second-order rate constants are not well-defined [11,30]. However, we were able to estimate a time scale by computing the flux through an absorbing circle of radius $\bar{\varrho }$ when the computational domain remains finite (see Appendix A in S1 Text for details). In this case, the flux is given by $J=2\pi DC/\ln\left(r_{max}/\bar{\varrho }\right)$, where *r*_*max*_ characterizes the size of the computational domain. In contrast to the 3D case, in the limit *r*_*max*_ → ∞, the 2D flux goes to zero. Thus, we used the flux on a finite domain to estimate a time scale for second-order diffusion-limited reactions, $k_{DL}=2\pi D/\ln\left(r_{max}/\bar{\varrho }\right)$, which has the units of a 2D second-order rate constant. This expression is represented by the red curve in Fig 2.

The other regime for second-order reactions is the reaction limit. In this limit, multiple encounters on average are required before the reaction occurs. We computed a second-order rate constant in the reaction limit by assuming the reactants are uniformly distributed. In 2D, this produces an overall reaction rate of $(\pi {\bar{\varrho }}^{2}/A)\lambda N_{A}N_{B}$, where $\pi {\bar{\varrho }}^{2}$ is the capture area, *A* is the area of the system, *λ* is the microscopic reaction rate, and *N*_*A*_ and *N*_*B*_ are the particle numbers for the two reactants. This leads to a second-order rate constant of $k_{RL}=\pi {\bar{\varrho }}^{2}\lambda$. The reaction limit is illustrated by the black dashed line in Fig 2.

In 3D, the $\lambda -\bar{\varrho }$ theory of Erban, Chapman and co-workers can be used to compute macroscopic rate constants from the underlying microscopic parameters (*λ*, *D*, and $\bar{\varrho }$) regardless of the reactants' diffusion coefficients [39,40]. The theory assumes the two reactants have a summed diffusivity *D*, and that reactions proceed with rate *λ* if the two reactants are within $\bar{\varrho }$ of one another. In general, the $\lambda -\bar{\varrho }$ theory cannot be extended to 2D, because in the diffusion limit, the rate at which a 2D second-order reaction proceeds cannot be described using a single rate constant [30]. Despite that, we used the $\lambda -\bar{\varrho }$ formulism to compute 2D rate constants (Appendix A in S1 Text, Figs A and B in S1 Text). Comparing calculations using the $\lambda -\bar{\varrho }$ formulism (green dashed line, left panel, Fig 2) to the results based on particle simulations (yellow diamonds, left panel, Fig 2), we find they are accurate predictions of reaction kinetics if the system is not too far from the reaction limit. We then attempted to estimate *λ* values from rate constants used in published models of yeast polarity establishment. In doing so, we discovered that several published second-order rate constants appeared to be larger than our estimate for the diffusion limit, *k*_*DL*_ (Fig C in S1 Text). However, the considerations discussed above do not take into account the fact that molecules involved in polarity can transition between the cell membrane and cytoplasm. As discussed next, the different diffusion coefficients associated with these different cellular compartments further complicates the mapping between microscopic and macroscopic parameters.

In the biochemical network that drives polarity, reactive chemical species can exchange between the membrane, where diffusion is relatively slow, and the cytoplasm, where diffusion is relatively fast. The reactivity of these species also changes depending upon whether they are in the membrane or cytoplasm. Existing methods to estimate 2D macroscopic rate constants from microscopic parameters under diffusion-limited conditions [30] do not consider the effect of membrane-cytoplasm exchange. Here, we were able to overcome this issue by empirically estimating effective rate constants by fitting chemical kinetic equations to results from our particle-based simulations.

**Fig 3:**
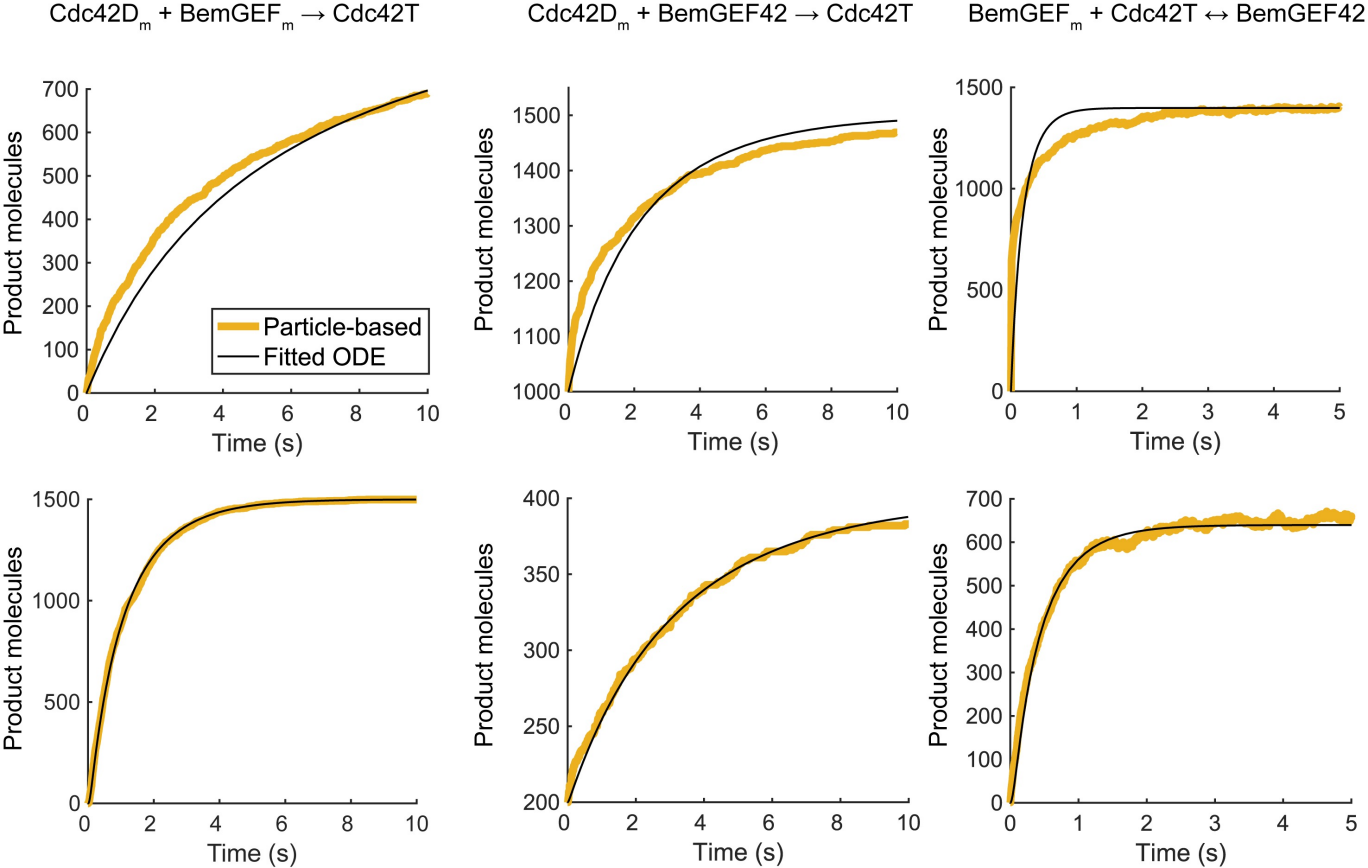
Empirical estimates for macroscopic rate constants in the yeast polarity model for the two different parameter sets. Results from particle-based simulations that include membrane exchange are shown as yellow curves. Fits to the simulation results using appropriate rate equations are shown as black curves. Top row, parameters used for purely 2D simulation. Bottom row, parameters used for quasi-3D simulation. The rate constants are reported in Table 2.

We conducted particle-based simulations of reversible second-order association reactions that accounted for mass exchange between the cytoplasm and membrane in a purely 2D system. Briefly, for each parameter set, we started with previously published rate constants from RDE models for polarity establishment [28,35,41], used the $\lambda -\bar{\varrho }$ formalism to estimate *λ*’s, then performed particle simulations and fit rate equations to the simulation results to compute the macroscopic rate constants. For purely 2D simulations, significant changes to the published parameter values were made to facilitate polarization for benchmarking purposes. For whole cell, quasi-3D simulations, parameters were held close to published values with exceptions for the bimolecular reactions obtained from the fitting procedure. We present the fits for the purely 2D and quasi-3D cases (Fig 3; Figs. I and M in S1 Text), as well as the rate constants (Table 2, Table A in S1 Text). Fitting the simulation results to appropriate chemical rate equations produced good estimates for the quasi-3D case (Fig 3, bottom row) and reasonable ones for the purely 2D case (Fig 3, top row). Additional analyses of the polarity network, discussed below, further supported the validity of the mapping.

**Table 2:** Microscopic parameters and effective macroscopic rate constants for reversible/irreversible bimolecular reactions of the form A + B ↔ C.

Usage	Reaction	Membrane on/off rates				Mean fitted	
		k_Aon_ (1/s)	k_Aoff_ (1/s)	k_Bon_ (1/s)	k_Boff_ (1/s)	k_f_	k_r_ (1/s)
2D	Cdc42D_m_ + BemGEF_m_ → Cdc42T	36	13	10	40	0.040 μm^2^s^-1^	-
	Cdc42D_m_ + BemGEF42 → Cdc42T	36	13	-	-	0.184 μm^2^s^-1^	-
	BemGEF_m_ + Cdc42T ↔ BemGEF42	10	40	-	-	0.054 μm^2^s^-1^	31.4
q3D	Cdc42D_m_ + BemGEF_m_ → Cdc42T	36	0.65	10	10	0.16 μM^-1^s^-1^	-
	Cdc42D_m_ + BemGEF42 → Cdc42T	36	0.65	-	-	0.16 μM^-1^s^-1^	-
	BemGEF_m_ + Cdc42T ↔ BemGEF42	10	10	-	-	0.79 μM^-1^s^-1^	0.37

The rates constants *k*_Aon_ and *k*_Bon_ are the rates at which the reactants, A and B, associate with the membrane, respectively, and the rate constants *k*_Aoff_ and *k*_Boff_ are the corresponding off rates. Mean fits were computed over simulations using five separate initial conditions; see Table A in S1 Text for more details, and Table 3 for pre-fitting target parameters. These values refer to parameters used in Fig 3.

### Microscopic fluctuations speed polarity establishment and increase robustness

**Fig 4:**
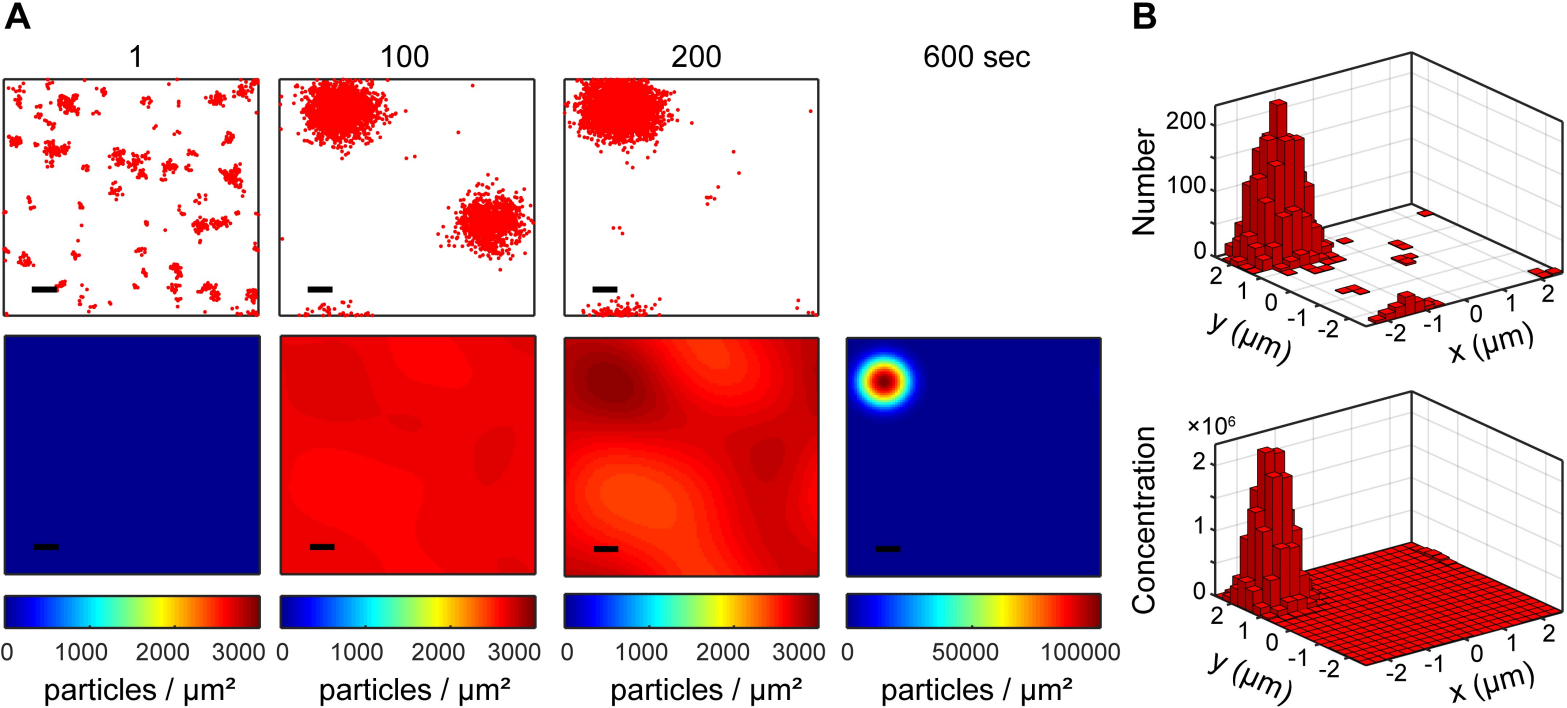
Simulations of polarity establishment within the Turing unstable regime. Snapshots of total Cdc42-GTP (both Cdc42-GTP and Bem1-GEF-Cdc42-GTP). Top: Particle-based simulations. Red dots represent individual molecules. Bottom: Reaction-diffusion partial differential equation simulations. **(A)** Individual molecules in particle-based simulations, and individual pixels in 100x100 grid RDE simulations. Scale bar, 0.5 μm. **(B)** To compare the polarity patches, 2D histograms of the final polarized states were computed, where both distributions were binned on coarsened 20x20 grids.

We compared stochastic particle-based and deterministic reaction-diffusion-based simulations. First, we focus on our results in the purely 2D system. Our initial conditions for the particle-based simulations had all molecules in the cytoplasm in inactive and uncomplexed states. As expected, stochastic fluctuations permitted escape from this spatially homogeneous initial state, ultimately leading to polarization (Fig 4A, S1 Movie). To fairly compare particle simulation results with solutions to the RDEs, molecular distributions from particle-based simulations at *t* = 1 second were used as initial conditions for the RDEs (see *Models and Methods* and Fig D in S1 Text). The two simulation methods generated similar polarized distributions (Fig 4B).

**Fig 5:**
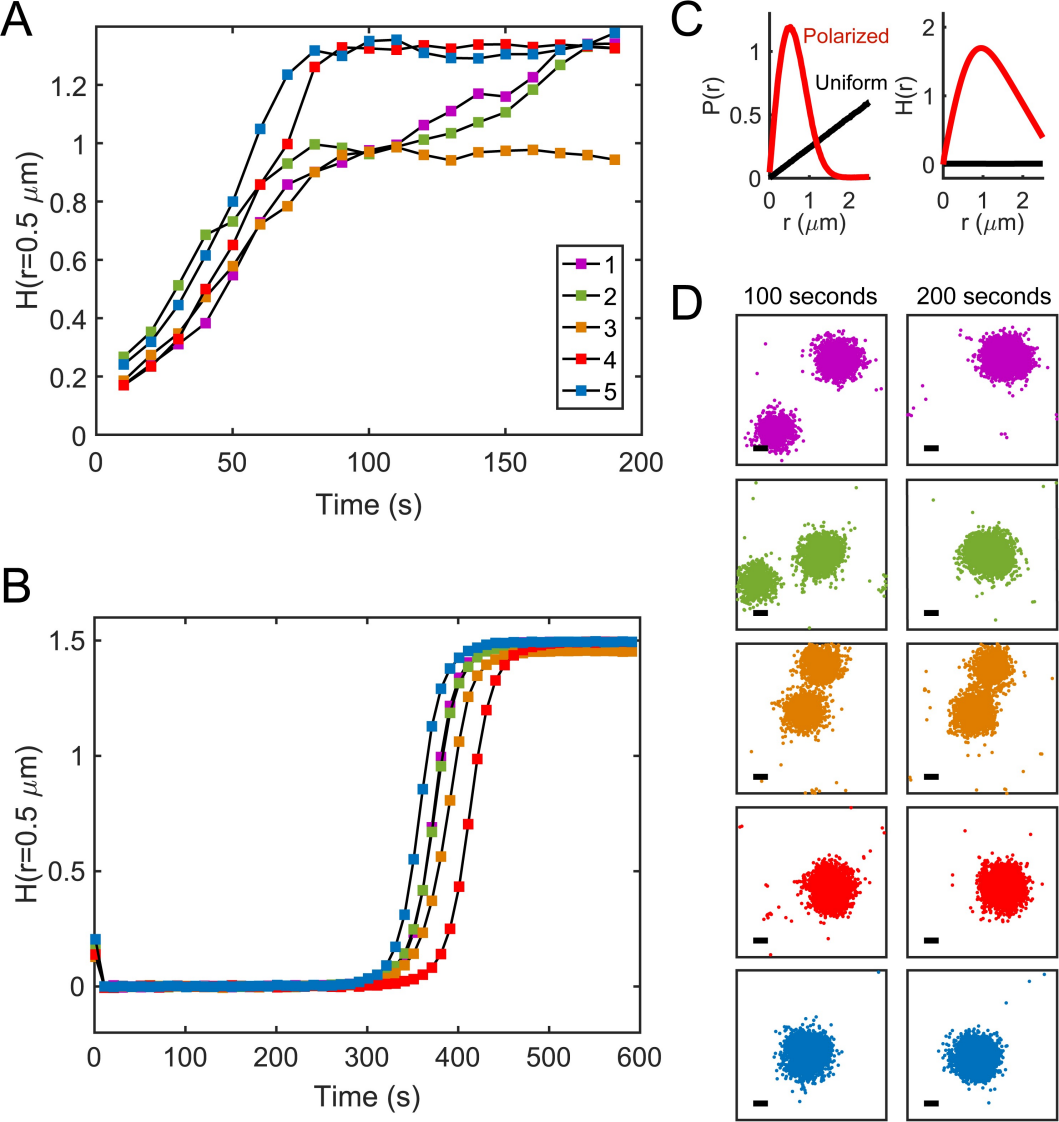
Variability in 2D polarization from microscopic fluctuations. **(A)** Measurements of *H*(*r* = 0.5 μm) at 10 second intervals across *n* = 5 particle-based simulation realizations. **(B)** Measurements of *H*(*r* = 0.5 μm) across the corresponding RDE simulations. (**C**) The pairwise distance distribution *P*(*r*) and our polarity metric *H*(*r*) for polarized (red) and uniform (black) particle distributions. **(D)** Snapshots of total Cdc42-GTP for each particle-based realization at *t* = 100 and *t* = 200 seconds. In some cases, particle coordinates were re-centered after simulation to keep polarity patches from visually wrapping around to the other side of the periodic domain. Corresponding RDE snapshots are in Fig H in S1 Text. Scale bars 0.5 μm.

To quantitatively compare polarization between the two approaches, we used the function *H*(*r*), which measures the deviation of a particle distribution from a uniform distribution based on the pairwise distance distribution (see *Models and Methods* and Fig 5C). *H*(*r*) and the related metric, Ripley's K-function, have been used frequently to study clustering in biology [42,43]. Positive values of *H*(*r*) correspond to increased density of the distribution at distances *r* relative to a uniform distribution. A maximum in *H*(*r*) denotes a characteristic size. We use this measure of spatial heterogeneity to quantify polarity. At steady-state, polarized distributions from the particle-based and RDE simulations had essentially identical *H*(*r*) curves (Fig E in S1 Text), suggesting the two systems were equivalently parameterized. We calculated *H*(*r*) over time for simulations using different parameter values to quantitatively compare polarization dynamics from particle-based and RDE simulations. Rather than choosing the *r* that maximizes *H*(*r*) under different conditions, we chose *r* = 0.5 μm for our analyses. This value allowed comparisons across all data sets, including those where the simulation domain size was varied. Qualitative features of our results do not depend on our choice of *r*, nor on the particle-based time point used to initialize the RDEs (Figs F and G in S1 Text).

To account for variability in polarization dynamics, we considered multiple realizations of single simulation conditions (Fig 5). In several cases, metastable multi-polar states emerged from initially unpolarized distributions, consistent with prior theoretical and experimental [4,34,35] work. Though it is not possible to identify multi-polar states from looking at *H*(*r*) alone, if the system goes through a slow phase of competition wherein metastable patches exist, then the time course of *H*(*r*) temporarily plateaus. For one realization, resolution into a single polarity site did not occur by 200 seconds (Fig 5, Simulation 3). For other realizations of the same parameter set, the simulation yielded a unique polarity site in half the time. Overall, the particle-based simulations polarized more rapidly than the RDEs, which were completely unpolarized at *t* = 200s. This indicates that molecular fluctuations increased the rate at which polarity establishment occurred. The RDEs did not exhibit transient plateaus in *H*(*r*), indicating metastable multi-patch states did not emerge, which is a direct consequence of the initial conditions (see also Fig H in S1 Text).

**Fig 6:**
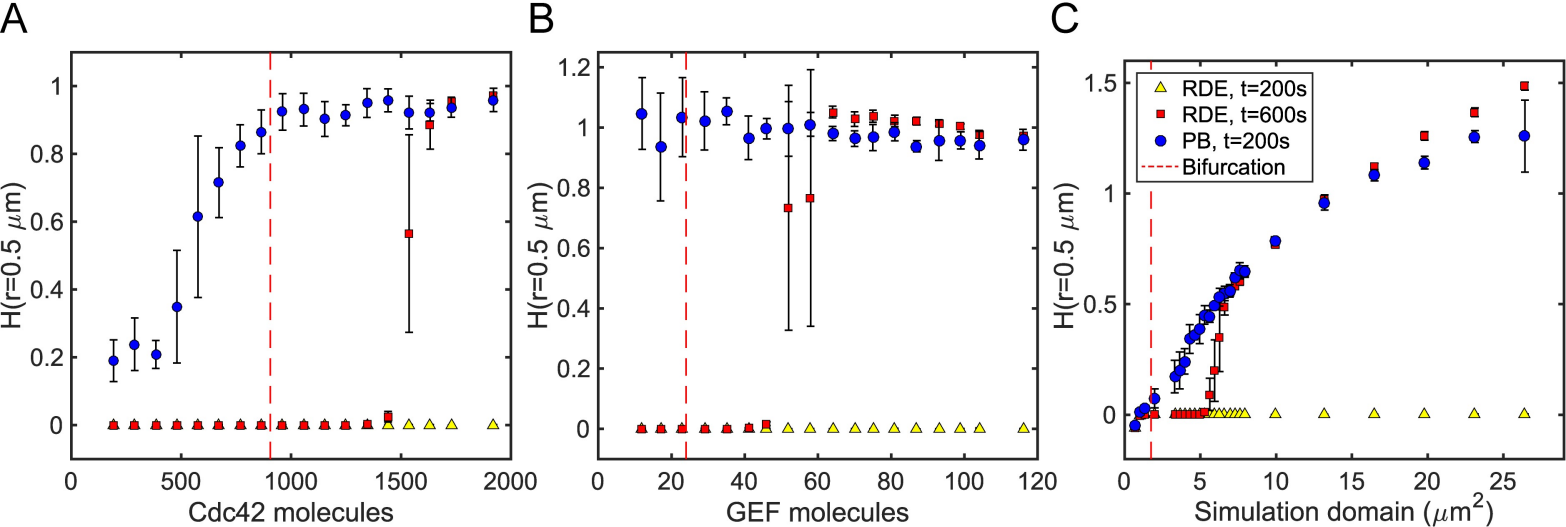
Stochasticity facilitates polarization. Bifurcation plots showing polarization, measured by *H*(*r* = 0.5 μm), versus parameters influencing the total particle numbers in the simulation. **(A)** Varying Cdc42 concentration on a fixed domain. **(B)** Varying GEF concentration on a fixed domain. **(C)** Varying the simulation area and particle numbers at constant concentrations. Bifurcations were found via linear stability analysis of the deterministic RDEs.

It has been demonstrated that sufficiently strong fluctuations can allow polarization outside of the Turing unstable regime [6,7,32,33]. These investigations relied on simplified models or phenomenological methods for introducing noise into the system. To test if intrinsic fluctuations are sufficient to produce “noise-induced” polarity, we examined 2D polarity establishment as a function of Cdc42 concentration, Bem1-Cdc24 (GEF) concentration, and total particle number at fixed concentration, generating bifurcation diagrams for these parameters. We used linear stability analysis of the RDEs to determine the bifurcation point at which the spatially homogenous solution goes through a Turing instability as molecular abundances and system size were varied (see *Models and Methods* and Fig J in S1 Text). This analysis established threshold values at which the RDEs no longer polarize, i.e. the homogeneous stable regime. The bifurcation plots are shown in Fig 6.

Across all parameters tested, none of the RDE simulations polarized to a measurable degree after 200 seconds. In contrast, most particle-based simulations exhibited polarity by then. Within the Turing unstable regime, the RDE simulations show similar levels of polarization around 600 seconds compared to the particle-based simulations. However, near the bifurcation point within the Turing unstable regime, the RDEs did not polarize even after 600s, consistent with the slowed patterning expected from bifurcation theory. In this parameter regime, the particle-based simulations still clearly exhibited polarity within 200 seconds. Furthermore, for the Cdc42 and GEF bifurcation diagrams, the particle-based simulations showed polarization below the critical point, in the Turing stable regime, showing that molecular fluctuations can increase the range over which polarity establishment occurs. Together, our observations reveal that stochastic effects facilitate polarization in this 2D instance of the Turing-type model by decreasing time to polarize and expanding the parameter space in which polarity can occur.

### A quasi-3D approach to full cell simulations

**Fig 7:**
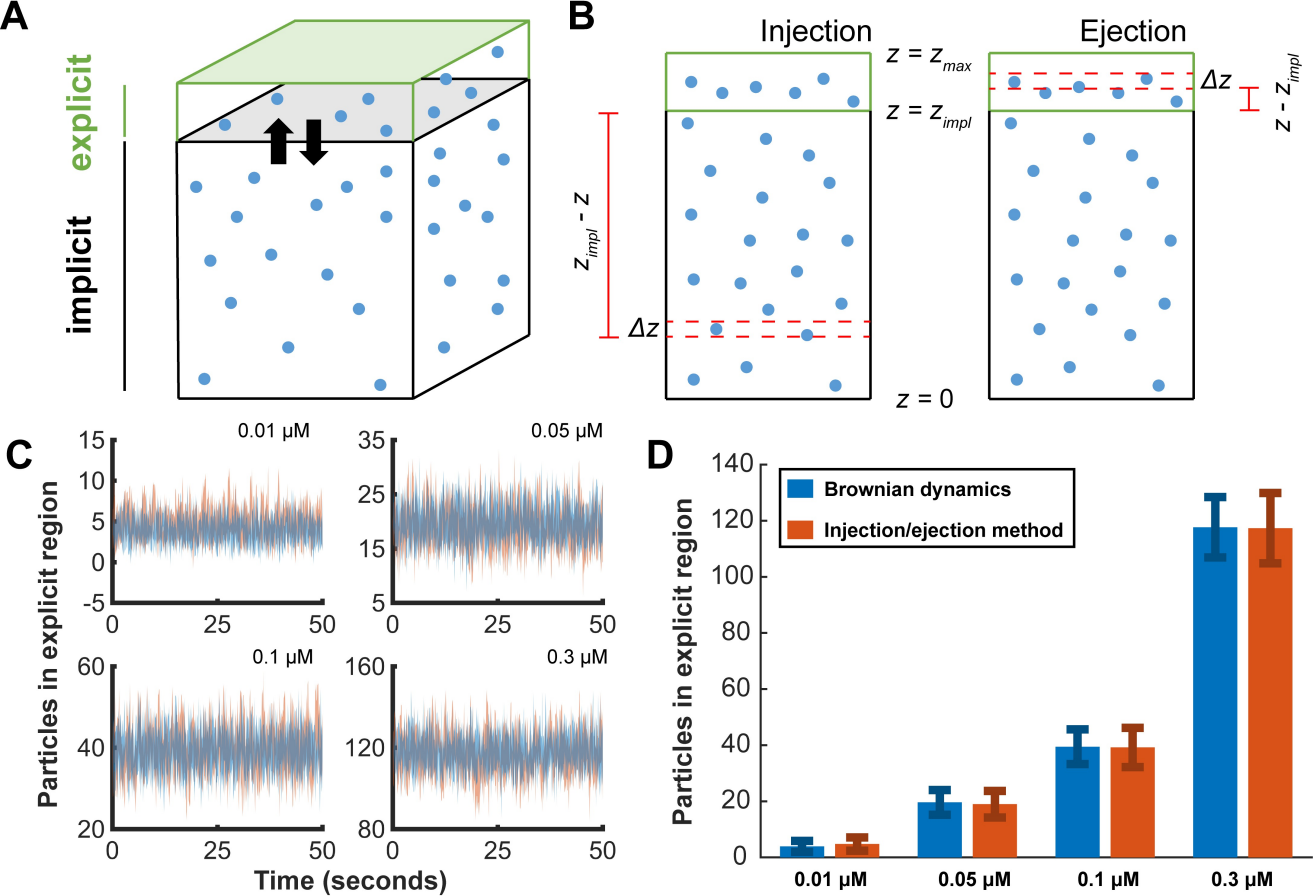
Reservoir approach schematics and validation. **(A)** Molecules can diffuse in and out of the reservoir. Although distinct molecules are shown for illustration, the reservoir is perfectly mixed. **(B)** Particles at a depth *z* must diffuse a distance of either *z*_*impl*_—*z* to enter, or *z*–*z*_*impl*_ to exit, the explicit simulation domain. The integrals are solved numerically over discrete slices with thickness Δz. **(C)** Time courses of the number of molecules in the explicit domain, comparing our approach and a non-reactive Brownian dynamics simulation. The shaded regions represent the mean±1 S.D. over 5 realizations. **(D)** Time-averaged comparisons, mean±1 S.D. of fluctuations, over 500s, 1 realization.

We next expanded our approach to approximate a whole cell by introducing a molecular reservoir to account for contributions from the bulk cytoplasm, yielding a quasi-3D approach (Fig 7A and 7B). The cytoplasmic reservoir was treated implicitly: we only tracked the number of molecules in the reservoir, instead of the dynamics of individual particles. To simulate stochastic exchange between the explicitly-modeled and implicitly-modeled regions of the cytoplasm, we took a similar approach as described in [44], using diffusional probability distributions to determine the number of molecules injected into (*n*_*inj*_) and ejected from (*n*_*ejc*_) the explicitly-modeled cytoplasm at each time step. Diffusional probability densities were integrated to obtain *P*_*inj*_ and *P*_*ejc*_, which correspond to the probability that a single molecule at a depth *z* diffuses the distance required to enter (*z*_*impl*_—*z*) or exit (*z*–*z*_*impl*_) the explicit simulation region (see Appendix D in S1 Text for derivation).

$$P_{inj}(z)=\frac{1}{2}\left[\mathrm{erf}\left(\frac{z_{max}-z}{\sqrt{4D\Delta t}}\right)-\mathrm{erf}\left(\frac{z_{impl}-z}{\sqrt{4D\Delta t}}\right)\right]$$

$$P_{ejc}(z)=\frac{1}{2}\left[\mathrm{erf}\left(\frac{z}{\sqrt{4D\Delta t}}\right)-\mathrm{erf}\left(\frac{-(z_{impl}-z)}{\sqrt{4D\Delta t}}\right)\right]$$

where *z*_*max*_ is the total height of the implicit and explicit domains, and *z*_*impl*_ is the height of the implicit domain. *P*_*inj*_(*z*) and *P*_*ejc*_(*z*) are approximations, since the probability densities in the derivation correspond to a freely diffusing particle on an infinite domain. Next, to calculate the mean number of particles that are injected and ejected, we integrated the injection and ejection probability densities over the appropriate domain, and multiplied by the 3D concentration and the surface area.

$$\langle n_{inj}\rangle (t)=c_{impl}(t)∙A_{r}∙\int_{0}^{z_{max}-z_{impl}}P_{inj}(z)dz$$

$$\langle n_{ejc}\rangle (t)=c_{expl,cyto}(t)∙A_{r}∙\int_{z_{max}-z_{impl}}^{z_{max}}P_{ejc}(z)dz$$

Finally, to approximate the stochastic fluctuations introduced by particles diffusing in and out of the explicit simulation domain, we sampled from Poisson distributions at each time step with means ⟨*n*_*inj*_⟩ and ⟨*n*_*ejc*_⟩. Coupling this reservoir to the cytoplasmic layer of the 2D particle-based method yielded our quasi-3D full-cell particle-based approach. Comparisons between this approximate method and Brownian dynamics simulations of diffusing particles showed that our molecular reservoir approach was consistent with both the mean and standard deviation for particle number over time (Fig 7C and 7D).

### Polarity establishment in a quasi-3D whole cell model

**Fig 8:**
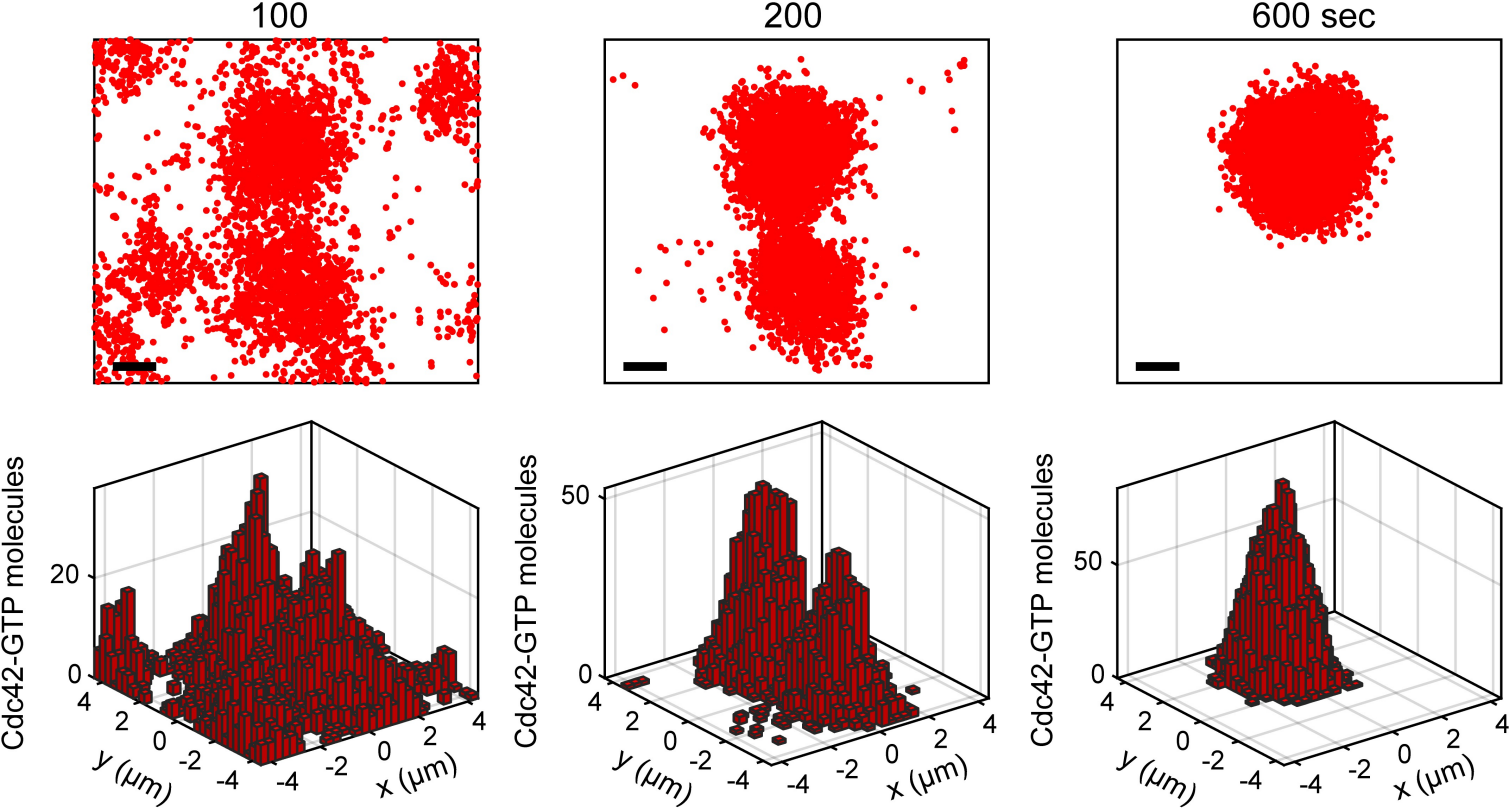
Quasi-3D particle-based simulations of the polarity establishment model. Shown are snapshots of total Cdc42-GTP. Scale bar, 1.0 μm. Corresponding 2D histograms of the local number of molecules are shown.

We performed quasi-3D simulations of a whole yeast cell by combining our 2D particle-based approach with stochastic exchange to and from a molecular reservoir representing the bulk cytoplasm. Empirical estimation of rate constants was again performed by fitting rate equations to the particle-based simulations (Fig M in S1 Text). We conducted simulations using 0.050~0.3 μM Cdc42, and 0.06 μM BemGEF (*N*_Cdc42_ = 1,970~11,820 and *N*_BemGEF_ = 2364, assuming a volume corresponding to a spherical cell with a 5 μm diameter). Quantitative Western blotting experiments by Watson et al. support 5,000–10,000 Cdc42 copies per cell, consistent with our choice for concentration range [45], while previous models assumed [Cdc42] ranging from 19.3 nM [46] to 5 μM [28]. Other models specify [BemGEF] ranging from 0.017 μM [4,28] to 0.06 μM [41]. Since prior experimental work showed that multi-polar states can resolve within 2 minutes [34,35], we initially limited our particle-based simulations to 200 seconds. This simulation time was insufficient for complete polarization, as multiple or misshapen patches were observed at *t* = 200s (Fig 8 middle; Fig N in S1 Text).

**Fig 9:**
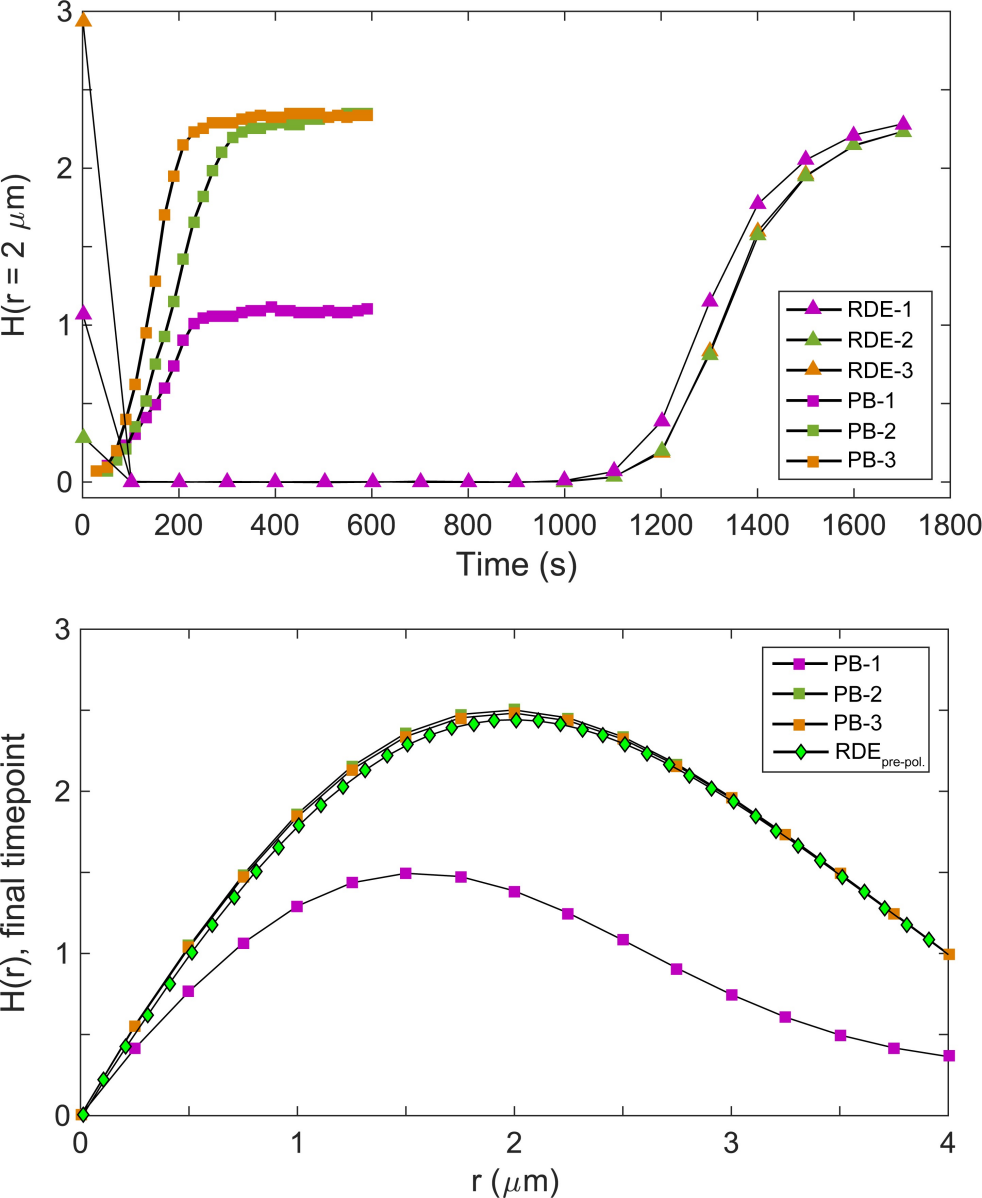
Quantitative comparisons of polarization in quasi-3D particle-based simulations and corresponding RDEs. Top: time courses of *H*(*r* = 2 μm). Results across multiple realizations of [Cdc42] = 0.150 μM are shown. Bottom: Plots of *H*(*r*) at final time points. By 1800s, the q3D-RDEs did not fully polarize, so the *H*(*r*) starting from a pre-polarized distribution is shown instead.

Before performing longer particle-based simulations, we determined the bifurcation point as a function of Cdc42 concentration in the analogous quasi-3D RDEs (see Models). Rather than perform linear stability analysis of these equations, we generated pre-polarized distributions and examined whether they decayed towards homogeneity to estimate the bifurcation point (Fig P in S1 Text). We found that [Cdc42] ≥ 0.055 μM was sufficient for polarization, but [Cdc42] = 0.050 μM could not sustain polarity. Therefore, we chose to extend simulations with [Cdc42] = 0.050, 0.055, 0.060, 0.150, and 0.155 μM for another 400 seconds. This simulation time was sufficient to tighten misshapen polarity sites if no competitor patch existed (Fig 9, Simulations 2 and 3; see also Fig N in S1 Text). In one case, two co-existing patches resolved into one within the 400s extension period (Fig 8). In another case, the patches did not resolve (Fig 9, Simulation 1). The capacity to resolve competition within the 400s window suggests that biologically relevant competition time scales can be obtained purely through stochastic molecular fluctuations. The time scale for competition observed here is consistent with Wu et al.’s theoretical work on this signaling model, where about 5 minutes was needed to resolve two-patch competition in the context of an RDE with Gaussian noise added [28].

To compare with the deterministic case, we ran quasi-3D RDE simulations for 1800s total, initialized with molecular distributions from *t* = 1 s of the quasi-3D particle-based simulations. Polarization dynamics were quantified using *H*(*r* = 2 μm), which matched the size of a fully-formed polarity site. Similar to the purely 2D case, we found that fully polarized particle-based simulations were quantitatively consistent with fully polarized RDE simulations, and that the RDE simulations took much longer to polarize than the corresponding particle-based simulations (Fig 9). No multi-patch states emerged in the RDEs, but we expect multi-patch states to compete even more slowly, supporting the importance of molecular fluctuations in using a Turing-type model to capture appropriate polarization timescales.

**Fig 10:**
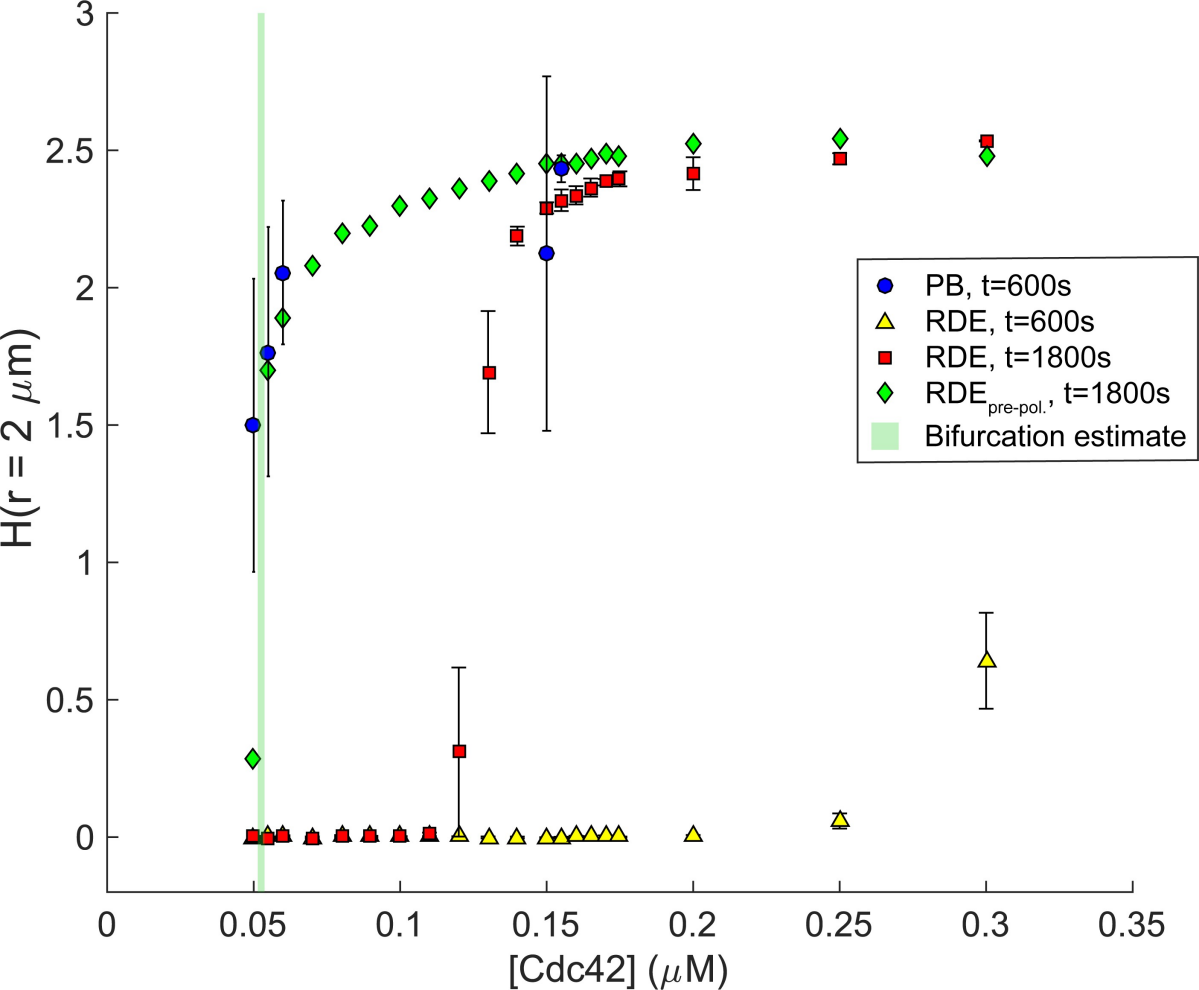
The effect of Cdc42 concentration on polarization for quasi-3D particle-based simulations. A bifurcation diagram comparing polarity, measured via *H*(*r* = 2 μm), in the particle-based and reaction-diffusion simulations as a function of Cdc42 concentration. Simulations with pre-polarized RDEs were used to identify an estimated range for the bifurcation point. All other points are given by the mean±1s.d. (*n* = 5 realizations, except for *t* = 600s particle-based simulations at [Cdc42] = 0.150 μM, *n* = 3, and 0.155 μM, *n =* 4).

Finally, to examine the robustness of this behavior over realistic concentration regimes, we compared polarization in the quasi-3D particle-based and quasi-3D RDE systems as a function of Cdc42 concentration. Our observations here were consistent with the purely 2D results. Particle-based simulations at *t* = 600s exhibited clear polarization, even at [Cdc42] = 0.050 μM, outside the deterministically non-polarizing region (Fig 10, S2 Movie). At the highest concentration, quasi-3D RDE simulations exhibited partial polarization at *t* = 600s, but by *t* = 1800s, most of the RDEs beyond the bifurcation exhibited measurable polarization. The macroscopic system we studied here represents a 3-compartment model (membrane, near-membrane, and bulk cytoplasm). Though Wu et al. reported a similar competition time scale, they utilized a volume-adjusted, two-compartment model of the RDEs [28]. To facilitate comparison, we also performed particle-based simulations to examine the volume-adjusted, two-compartment system's bifurcation diagram with respect to Cdc42 concentration. There is qualitatively no change in our results, and linear stability analysis of the volume-adjusted, two-compartment system is consistent with the numerically determined bifurcation point for the q3D-RDEs (Fig P in S1 Text).

## Discussion

Strong positive feedback to amplify heterogeneities in molecular distributions is an important component of many models of cellular polarity establishment. Given the stochastic nature of biochemical reactions involved in the polarity circuit, local heterogeneities are expected to emerge everywhere along the cell. Work in both non-Turing type [21,23,24], and Turing-type systems [6,7,32,33] has shown that stochasticity can aid pattern formation. Here, we provide the first simulations of particle-based Turing-type yeast polarity establishment. Both our 2D and quasi-3D particle-based simulations capture microscopic stochastic effects, which indeed facilitate polarization. As anticipated, differences between the particle-based and reaction-diffusion approaches were most obvious around the bifurcation point (Fig 6, Fig 10). Stochastic fluctuations allowed for polarization outside of the Turing unstable regime and more rapid polarity establishment across all parameters tested. Turing-type patterning mechanisms have been described as slow relative to other hypothesized patterning mechanisms, such as wave-pinning [5], making it a less likely biological mechanism in some contexts. Our simulations highlight that molecular fluctuations can alleviate such issues. Given our simulations do not include other sources of fluctuations, such as endocytic and exocytic events [47,48], our results represent the minimal level of variability expected to be observed in polarity establishment. This minimal variability is sufficient to generate significant variations in competition times across multiple realizations of a single parameter set (Fig 5, Fig 9), even at molecular abundances representative of whole yeast cells. Therefore, particle-based simulations are an important computational tool for understanding the dynamics and control of biological pattern formation.

Polarity establishment is often modeled using reaction-diffusion equations that ignore the discrete nature of biomolecules, and treat concentrations of molecular species as continuous variables. The chemical rate constants that appear in these equations represent macroscopic quantities that depend on microscopic properties, such as diffusion coefficients and molecular size. In three-dimensional domains where particles diffuse with a single diffusion coefficient, theories for computing macroscopic rate constants from the underlying microscopic dynamics are well established [15,40]. However, for two-dimensional systems, second-order rate constants in the diffusion limit are not well-defined [30]. Additionally, in the polarity system, molecular species transition between the cytoplasm, where diffusion is relatively fast, to the plasma membrane, where diffusion is relatively slow. Developing theories for computing appropriate rate constants under these conditions is an active area of research, and we did not attempt to provide a theoretical framework here. Instead, we took an empirical approach, estimating effective second-order rate constants by fitting rate equations to the results of particle-based simulations of isolated reactions (Fig 3; Figs I and M in S1 Text). This approach allowed us to make fair comparisons between our particle-based and RDE simulation simulations, as evidenced by quantitative similarities in polarization (Fig 9; Fig E in S1 Text) and equivalent kinetics under non-polarizing conditions (Figs B, K, and P in S1 Text). Still, our empirical approach to estimating rate constants cannot capture the correct kinetics under all conditions: in general, a single rate constant is inappropriate for describing 2D diffusion-limited reactions [30]. While this discrepancy presents challenges for comparing particle-based simulations to RDEs, it also highlights an advantage of particle-based simulations: the real behavior of a system might not be well-described with macroscopic approximations. We note that many polarity models based on RDEs employ effective kinetics, such as Michaelis-Menten or Hill kinetics. To perform particle-based simulations of these models requires “unpacking” these effective kinetic schemes into their elementary chemical steps. Doing so not only allows an investigation into the effects of molecular-level noise, but also provides a rigorous test for the validity of the approximate reaction schemes, whose derivations typically rely on a separation of time scales.

We also note that, in the context of our parameterization, the slow diffusivity on the membrane is important. Simulations using *D*_*m*_ = 0.03 μm^2^s^-1^ [46], an order of magnitude faster than the *D*_*m*_ used throughout our work, lose polarization within the pure 2D system if all other parameters are fixed (Fig L in S1 Text). This occurs even though *D*_*m*_ = 0.03 μm^2^s^-1^ maintains more than two orders of magnitude difference from the cytoplasmic diffusivity *D*_*c*_.

It is important to acknowledge some limitations of our approach. First, while treating the membrane and adjacent cytoplasm as a single 2D plane seems reasonable, it ignores effects from 3D curvature, which can play a role in the polarization process [49,50]. Additionally, the implied geometry of our system, a rectangular prism, means we likely overestimate cytoplasmic protein abundances near the cell membrane. To illustrate, a typical yeast cell has a diameter of 5 μm. This corresponds to a surface area of 78.5 μm^2^, treating the cell as a sphere. Then, mapping this surface area to a square, we obtain a square side length of 8.86 μm. The volume for a spherical *d* = 5 μm yeast cell is 26.2 μm^3^. To achieve an equal-volume rectangular prism, with a top face surface area of 78.5 μm^2^, the depth of the prism must be 0.833 μm. This is much smaller than the cell radius of 2.5 μm. Aside from the geometry of the system, our method also neglects gradients that might develop between the cell membrane and interior of the cell, either via chemical means or sufficiently slow cytoplasmic diffusion. The polarity network studied here does not involve reactions between two cytoplasmic species, so the reservoir component of the simulation is chemically inert. If reactions did occur within the cytoplasm, our particle-based approach could be extended to include chemical rate equations for the concentrations of the reservoir species, and our method for injection and ejection of particles would still be sufficient as long as cytoplasmic gradients were not of interest. However, if gradients of cytoplasmic components were required, then the reservoir would need to be modeled with PDEs, and the methods defining particle injection and ejection would need to be suitably adapted, along the lines of work done in [51]. Full treatment of the reservoir with a PDE approach would make the approach presented here more similar to hybrid methods such as [21].

In summary, we have found that molecular stochasticity can facilitate cellular polarity establishment by promoting the speed of polarization and expanding the effectively Turing unstable regime. We examined this phenomenon in the context of a Turing-type model of yeast signaling involving Cdc42 and Bem1-Cdc24 in a positive feedback loop. In particular, polarization within the quasi-3D system appears to occur roughly on biologically relevant timescales, which does not seem possible with deterministic RDEs. We also have highlighted general considerations for comparing the spatiotemporal dynamics of membrane-bound proteins at molecular, particle-based scales and at coarser, concentration-based scales. Symmetry breaking in many contexts involves guiding cues not considered here, such as a pheromone gradients or bud scars in yeast [1]. However, these cues can be surprisingly weak: a computational study of yeast pheromone receptors in a pheromone gradient predicted differences in receptor occupancy as small as 45±50 molecules between the front (towards with the gradient) and the back [44]. Future work may focus on examining how weak cues may allow robust polarization along shallow gradients. We have also developed a computational approach that is tailored to modeling biochemical signaling at and near the cell membrane with molecular resolution, while more coarsely handling the remaining cell cytoplasm. This approach is not intended to compete with highly optimized simulation platforms, but the concepts and mathematics derived may be useful in extending their functionalities.

## Models and methods

### The molecular circuit for polarity establishment

The molecular signaling network used in this work, illustrated in Fig 1C, was taken from Wu et al. [28] and originally derives from work by Goryachev and Pokhilko [4]. The network contains a positive feedback loop because Cdc42-GTP can bind a Bem1-Cdc42 complex to increase the GEF's catalytic activity. Cdc24 is a GEF, while Bem1 is a scaffold protein. We assume, as done previously, that Cdc24 and Bem1 function as essentially a single unit [41]. Tables 2 and 3 provide parameters used for simulations. The corresponding reaction-diffusion equations (RDEs) that govern the system are as follows:

$$\frac{\partial Cdc42T}{\partial t}=(k_{2a}BemGEF_{m}+k_{3}BemGEF42)∙Cdc42D_{m}-(k_{2b}+k_{4a}BemGEF_{m}+k_{7}BemGEF_{c})∙Cdc42T+k_{4b}BemGEF42+D_{m}\Delta Cdc42T$$

$$\frac{\partial Cdc42D_{m}}{\partial t}=k_{2b}Cdc42T-(k_{2a}BemGEF_{m}+k_{3}BemGEF42+k_{5b})∙Cdc42D_{m}+k_{5a}Cdc42D_{c}+D_{m}\Delta Cdc42D_{m}$$

$$\frac{\partial BemGEF42}{\partial t}={(k}_{4a}BemGEF_{m}+k_{7}BemGEF_{C})∙Cdc42T-k_{4b}BemGEF42+D_{m}\Delta BemGEF42$$

$$\frac{\partial BemGEF_{m}}{\partial t}=k_{1a}BemGEF_{c}+k_{4b}BemGEF42-(k_{1b}+k_{4a}Cdc42T)BemGEF_{m}+D_{m}\Delta BemGEF_{m}$$

$$\frac{\partial BemGEF_{c}}{\partial t}=k_{1b}BemGEF_{m}-(k_{1a}+k_{7}Cdc42T)∙BemGEF_{c}+k_{inj}BemGEF_{c,res}-k_{ejc}BemGEF_{c}+D_{c}\Delta BemGEF_{c}$$

$$\frac{dBemGEF_{c,res}}{dt}=\int_{\Omega \in Reservoir}^{}[-k_{inj}BemGEF_{c,res}+k_{ejc}BemGEF_{c}]d\Omega$$

$$\frac{\partial Cdc42D_{c}}{\partial t}=k_{5b}Cdc42D_{m}-k_{5a}Cdc42D_{c}+k_{inj}Cdc42D_{c,res}-k_{ejc}Cdc42D_{c}+D_{C}\Delta Cdc42D_{c}$$

$$\frac{dCdc42D_{c,res}}{dt}=\int_{\Omega \in Reservoir}^{}[-k_{inj}Cdc42D_{c,res}+k_{ejc}Cdc42D_{c}]d\Omega$$

$$k_{inj}=\int_{0}^{z_{max}-z_{impl}}P_{inj}(z)dz$$

$$k_{ejc}=\int_{z_{max}-z_{impl}}^{z_{max}}P_{ejc}(z)dz$$

where Δ here denotes the two-dimensional Laplacian, and the terms containing the rates *k*_inj_ and *k*_ejc_ are for the cytoplasmic reservoir. In the purely 2D form of the RDEs, these reservoir terms are absent. The quasi-3D form directly follows schematics shown in Fig 1B and Fig 7, using a 2D membrane compartment, a 2D cytoplasmic compartment, and a reservoir to and from which mass is deterministically exchanged. The reservoir is assumed perfectly mixed, but the explicitly modeled cytoplasmic compartment is not. With this formulation, spatial gradients are possible in the *xy* plane (i.e. along the cell membrane), but they are ignored along *z* (i.e. moving into the cell). Previous work has considered cytoplasmic diffusion coefficients from 1 μm^2^/s up to infinity (i.e., perfectly well-mixed). We chose a finite diffusion coefficient here. We also considered an RDE system that treats the membrane and cytoplasm as a two-compartment system, as in [28]. Results from this system were qualitatively similar to our 3-compartment model (see Fig Q in S1 Text).

**Table 3:** Parameters used to perform simulations described in the main text.

Description	Parameter	For purely 2D simulations	For quasi-3D simulations	Ref.
BemGEF_c_ → BemGEF_m_	k_1a_	10 s^-1^	10 s^-1^	[52]
BemGEF_m_ → BemGEF_c_	k_1b_	40 s^-1^	10 s^-1^	[52]
Cdc42D_m_ + BemGEF_m_ → Cdc42T	k_2a_	Target 0.032 μm^2^s^-1^ Fitted 0.040 μm^2^s^-1^	Target 0.16 μM^-1^s^-1^ Fitted 0.16 μM^-1^s^-1^	[52]
	*λ*_2a_	5.3 s^-1^	5.3 s^-1^	—
Cdc42T → Cdc42D_m_	k_2b_	0.35 s^-1^	0.32 s^-1^	[35]
Cdc42D_m_ + BemGEF42 → Cdc42T	k_3_	Target 0.280 μm^2^s^-1^ Fitted 0.184 μm^2^s^-1^	Target 0.35 μM^-1^s^-1^ Fitted 0.16 μM^-1^s^-1^	[52]
	*λ*_3_	180 s^-1^	15.7 s^-1^	—
BemGEF_m_ + Cdc42T → BemGEF42	k_4a_	Target 0.050 μm^2^s^-1^ Fitted 0.054 μm^2^s^-1^	Target 10 μM^-1^s^-1^ Fitted 0.79 μM^-1^s^-1^	[52]
	*λ*_4a_	9.6 s^-1^	8250 s^-1^	—
BemGEF42 → BemGEF_m_ + Cdc42T	k_4b_	Target 40 s^-1^ Fitted 31.4 s^-1^	Target 10 s^-1^ Fitted 0.37 s^-1^	[52]
Cdc42D_c_ → Cdc42D_m_	k_5a_	36 s^-1^	36 s^-1^	[52]
Cdc42D_m_ → Cdc42D_c_	k_5b_	13 s^-1^	0.65 s^-1^	[52]
BemGEF_c_ + Cdc42T → BemGEF42	k_7_	2.0014 μm^2^s^-1^	10 μM^-1^s^-1^	[52]
	*λ*_7_	256 s^-1^	256 s^-1^	—
Diffusion coefficient in cytoplasm	D_cyto_	15 μm^2^s^-1^	15 μm^2^s^-1^	—
Diffusion coefficient on membrane	D_memb_	0.0025 μm^2^s^-1^	0.0025 μm^2^s^-1^	[52]
Membrane to cytoplasm volume ratio	*η*	1	0.01006	[52]
Membrane surface area	A	0.21–10.5π	25π	[52]
Molecular interaction radii	$\bar{\sigma },\bar{\varrho }$	0.050 μm	0.050 μm	—
Total Cdc42		14.5–145.5 particles/μm^2^	0.05–0.30 μM	—
Total BemGEF		0.87–8.8 particles/μm^2^	0.06 μM	[41]

References are for the quasi-3D parameters. “Target” and “fitted” values for *k*_*2a*_, *k*_*3*_, *k*_*4a*_, and *k*_*4b*_ exist because of the empirical fitting described in the main text. The target was used as the input to 2D $\lambda -\bar{\varrho }$ theory as the starting point, producing the corresponding microscopic rates *λ*_*2a*_, *λ*_*3*_, *λ*_*4a*_, and *λ*_*7*_. Fitted macroscopic rates were obtained after fitting as described earlier (see Fig 3). For particle simulations, we used Δ*t* = 0.1 ms, and for RDE simulations, we used Δ*t* = 1 ms. For quasi-3D simulations, we assumed a cell volume corresponding to a 5 μm diameter sphere.

### Particle-based simulation implementation

All simulation code was developed and run in MATLAB R2016a/b, and was also run in R2013a. Some analysis code is known to not function on R2013a. Main components of the code are publicly available in the S1 Dataset and at https://github.com/mikepab. Simulations were performed both on desktop machines and on the University of North Carolina KillDevil computing cluster. See Appendix B in S1 Text for more detail.

Unimolecular and bimolecular reactions were handled as described in the Results section. To perform stochastic simulation of diffusion, we used the Euler-Maruyama method [36]:

$$x(t+\Delta t)=x(t)+\xi_{i}\sqrt{2D\Delta t}$$

$$y(t+\Delta t)=y(t)+\xi_{j}\sqrt{2D\Delta t}$$

where *x* and *y* are particle coordinates, and *ξ*_*i*_ and *ξ*_*j*_ are normally distributed random numbers *ξ* ∼ *N*(0,1). We vectorize this calculation across the set of particles that need diffusional updates. However, molecules that undergo association or dissociation events are not updated by the Euler-Maruyama method. When two molecules bind, their positions are updated by moving one of the particles to the exactly same position as its binding partner. When a complex dissociates, one of the constituent chemical species is placed a distance $\bar{\sigma }$ away, at a random angle drawn from a uniform distribution. Periodic boundary conditions are assumed in both spatial directions, so that intermolecular distances for various calculations were solved as the minimum Euclidean distance along the 2D surface of a torus. For visualization, all particle coordinates were translated on the periodic domain to keep polarity sites off of the boundaries. Movies were generated using the QTWriter package for MATLAB available at https://github.com/horchler/QTWriter.

### Empirically mapping macroscopic rate constants to microscopic parameters

A starting macroscopic rate constant was used to estimate an initial microscopic rate parameter *λ* via 2D *λ* – $\bar{\varrho }$ theory (see Table 3). Then, particle-based simulations of the individual (ir)reversible bimolecular reactions were performed, allowing the reactants to undergo membrane-cytoplasm exchange as appropriate for each target reaction. Whether the parameter set was intended for 2D or quasi-3D simulations, we performed these simulations on a 2D domain. For bimolecular reactions of the form A_m_+B_m_ ↔ C, with membrane-cytoplasm exchange reactions A_c_ ↔ A_m_ and B_c_ ↔ B_m_, time courses for particle numbers of A_m_, A_c_, B_m_, B_c_, and C were extracted from each simulation. The membrane-cytoplasm exchange rates (specified during the particle-based simulation) were fixed during the fitting procedure, so that only *k*_*f*_ and *k*_*r*_ were fit. Fitting was done using MATLAB’s built-in function fminsearch, where the sum of squared errors along normalized time courses (each scaled so that each species’ maximum value in the time course was 1) was minimized. The rate constant *k*_*7*_ did not need to be fit, as it involves a cytoplasmic reactant and is not diffusion-limited. We assume a height *h* = 0.0083 μm for the cytoplasmic volume adjacent to the cell membrane, which is consistent with parameterizations of the membrane-to-cytoplasm volume ratio *η* used previously for this system [28]. To convert between 3D bimolecular rate constants, *k*_*3D*_ (μm^3^s^-1^), and 2D rate constants *k*_*2D*_ (μm^2^s^-1^), we scale by *h*. We believe our empirical mapping is a fair comparison between the particle-based and RDE systems based on the quantitative similarities between polarity sites in the two methods (Fig E in S1 Text; Fig 9, bottom), as well as the consistency of species time courses in a Turing-stable regime (Fig K in S1 Text).

### Performing RD-PDE simulations

Initial conditions were generated by binning molecular distributions at *t* = 1 sec from each realization of the particle-based simulation at each simulation condition. These pixellations were obtained on 100 x 100 grids using MATLAB's histcounts2 function, to be consistent with the grid size used for RD-PDE simulation. Simulations were conducted using an operator splitting method, where reaction terms were solved using an adaptive Euler step, and diffusion terms were solved using a Fourier transform-based approach. As in the particle-based simulations, periodic boundary conditions were taken.

### Quantifying polarization

Polarization was measured using the H-function *H*(*r*), a rescaled version of Ripley’s K-function. Ripley’s K-function is a commonly used metric in experimental biology, and has been applied to study both experimental and simulated protein clustering [42,53]. The *H*(*r*) is related to the cumulative distribution of pairwise inter-particle distances *P*(*r*):

$$H(r)=\sqrt{\frac{L^{2}}{\pi }\int_{0}^{r}P(r^{\prime })dr^{\prime }}-r$$

$$P(r)=\frac{1}{N(N-1)}\sum_{i=1}^{N}m_{i}(r)$$

where *N* is the total number of particles subjected to cluster analysis, *m*_*i*_(*r*)Δ*r* is the number of particles at a distance *d* from particle *i* such that *r–*Δ*r/2* ≤ *d* ≤ *r +* Δ*r/2*, and *L* is the length along either the x or y axis of the square simulation domain. Since the simulation domain boundaries are periodic, *d* is the minimum Euclidean distance along the two-dimensional surface of a torus. *P*(*r*) can also be related to the well-known pair correlation function *g*(*r*) by normalizing *m*_*i*_(*r*)Δ*r* by the expected density of particles within the area defined by *r* ± Δ*r*/2. See Fig 5C and Fig E in S1 Text for examples.

*H*(*r*) = 0 if particles are distributed according to a uniform distribution. *H*(*r*) > 0 indicate the presence of more particles at inter-particle distances *r* as compared to a uniform distribution, while negative values of *H(r)* indicate fewer particles. Positive values of *H*(*r*) therefore indicate spatial clustering, or polarization, and the value of *r* for which *H*(*r*) is maximized reflects the characteristic cluster size. When "total Cdc42-GTP" levels were quantified or visualized, this meant both Cdc42-GTP and Bem1-GEF-Cdc42-GTP complexes, to reflect the total population of Cdc42-GTP molecules.

We also quantified *H*(*r*) in the RDE simulations by producing discrete particle distributions from continuous concentration data. We computed a cumulative distribution function corresponding to the concentrations in each spatially discretized bin, then drew pairs of random numbers *p*_*1*_, *p*_*2*_ ϵ Unif(0,1) to randomly select values from the cumulative distribution function, thus choosing two of the bins. Because the PDE assumes that molecules are uniformly distributed within each bin, four more uniform random numbers were drawn to pick locations within the two bins. This generated two sets of coordinates (*x*_*1*_,*y*_*1*_) and (*x*_*2*_,*y*_*2*_) such that (*x*_*1*_,*y*_*1*_) fits within the first bin, and (*x*_*2*_,*y*_*2*_) fits within the second. The pairwise distance between the points was recorded, and the process repeated many times to ensure sufficient sampling, typically *n* = 500,000. The resultant pairwise distance distribution is analogous to *P*(*r*), permitting similar steps as above to compute a reaction-diffusion analogue of *H*(*r*).

### Molecular injection and ejection from a cytoplasmic reservoir

To compute ⟨*n*_*inj*_⟩(*t*) and ⟨*n*_*ejc*_⟩(*t*) in practice, we numerically integrate over 100,000 discrete slices using the trapezoidal rule over the appropriate domains. Instead of re-calculating the integrals each simulation step, we pre-calculate the integrals and store the results in a table to save computation time. This yields the mean behavior, which is sufficient for the q3D-RDEs. However, to stochastically perform injection and ejection in the particle-based simulations, we draw uniform random numbers *x* ϵ [0,1] and use the inverse Poisson cumulative distribution function to obtain a Poisson-distributed particle number. To ensure physical validity, the tails of these probability distributions are cut off at the number of available particles in each compartment each time step.

### Linear stability analysis

We used linear stability analysis to determine conditions where the homogeneous steady state of the 2D reaction-diffusion system was Turing unstable [54]. In this analysis, the full reaction diffusion equations are linearized around the homogenous steady-state, and the effect of an arbitrary small spatial perturbation is evaluated. The small perturbation is represented as a linear combination of a particular set of spatial functions, which are eigenfunctions (modes) of the Laplacian operator and are subject to appropriate boundary conditions. Because of the linearity, the initial growth of each mode is proportional to $e^{\lambda_{n}t}$ where the eigenvalue *λ*_*n*_ depends on the corresponding wave number *k*_*n*_^*2*^. A small spatial perturbation will grow if Re[*λ*] is greater than zero for at least one mode. For a square domain of side *L* with periodic boundary conditions, the eigenfunctions are [55]:

$$W_{nm}(x,y)=\left(a_{n}\cos\frac{2n\pi x}{L}+b_{n}\sin\frac{2n\pi x}{L}\right)\left(a_{m}\cos\frac{2m\pi y}{L}+b_{m}\sin\frac{2m\pi y}{L}\right)$$

where

$$k_{nm}^{2}={\left(\frac{2n\pi }{L}\right)}^{2}+{\left(\frac{2m\pi }{L}\right)}^{2}$$

and *n* and *m* are integers. The coefficients *a*_*n*_, *b*_*n*_, *a*_*m*_, and *b*_*m*_ are constants determined by the initial perturbation and are not relevant in this analysis.

We calculate the dispersion relation Re[*λ*(*k*^*2*^_*nm*_)] for the set of PDEs that define the signaling network, noting that the system is unstable if Re[*λ*(*k*^*2*^_*nm*_)] > 0 for any mode *n*,*m*. If the system is in a Turing-unstable state, then decreasing the concentration of a particular species will induce a bifurcation where the system becomes stable to spatial perturbations. For the cases examined here (decreasing domain size or species concentration) the bifurcation occurs when Re[*λ*(*k*^*2*^_*nm*_)] becomes zero for the wave numbers *k*^*2*^_*01*_ and *k*^*2*^_*10*_ because these are the smallest relevant wave numbers (the mode *n* = 0, *m* = 0 corresponds to a uniform function and is not relevant in this analysis). See Fig H in S1 Text for an example.

### Numerical determination of the bifurcation point by pre-polarization

For the quasi-3D RDE system, where linear stability analysis was more difficult, we used this simpler, numerical approach to determine the [Cdc42] threshold below which the system cannot polarize. The initial conditions were as follows: we started with a system where all the Cdc42 and BemGEF was inactive, in the “explicit” cytoplasmic compartment. We then specified a square *L*/5 x *L*/5 centered on the origin, where *L* is the domain length, and converted 90% of the Cdc42 into the active membrane-bound state. All remaining cytoplasmic mass was partitioned according to diffusional equilibrium.

Simulations to determine the bifurcation point by pre-polarization were carried out for 600s, which under our conditions was sufficient to distinguish loss or maintenance of polarity on the basis of *H*(*r* = 2μm) (Fig P in S1 Text).

## Supporting information

S1 Text Supplemental information. This text describes our derivations for 2D bimolecular reactions (Appendix A), our particle-based simulation implementation (Appendix B), our additional results for 2D polarization (Appendix C), our derivation for the quasi-3D reservoir equations (Appendix D), and our additional results for quasi-3D polarization (Appendix E).
(DOCX)

S1 Movie Particle-based simulation of polarity establishment within the Turing unstable regime. Red dots represent individual molecules of total Cdc42-GTP (both Cdc42-GTP and Bem1-GEF-Cdc42-GTP). The movie shows 200 simulated seconds.
(MOV)

S2 Movie Quasi-3D particle-based simulation of polarity establishment outside of the Turing unstable regime. Red dots represent individual molecules of total Cdc42-GTP (both Cdc42-GTP and Bem1-GEF-Cdc42-GTP). The movie shows 600 simulated seconds. [Cdc42] = 0.050 μM.
(MOV)

S1 Dataset Key pieces of code and data. This .zip file contains a General folder with code for procedures described in the main text, and folders with data and code to render data-based parts of Figs 2–10.
(ZIP)
