# Supplementary material for: Particle-based simulations of polarity establishment reveal stochastic promotion of Turing pattern formation
Source: PLoS Comput Biol. 2018 Mar 12;14(3):e1006016. doi: 10.1371/journal.pcbi.1006016 (PMC5864077; doi:10.1371/journal.pcbi.1006016)
Supplement: S1 Text — This text describes our derivations for 2D bimolecular reactions (Appendix A), our particle-based simulation implementation (Appendix B), our additional results for 2D polarization (Appendix C), our derivation for the quasi-3D reservoir equations (Appendix D), and our additional results for quasi-3D polarization (Appendix E). (DOCX) [file pcbi.1006016.s001.docx]

**­­­­­­­­­­**Particle-based Simulations of Polarity Establishment Reveal Stochastic Promotion of Turing Pattern Formation

Michael Pablo^1,2^, Samuel A. Ramirez^3^, Timothy C. Elston^3^*

^1^Department of Chemistry, ^2^Program in Molecular and Cellular Biophysics, and ^3^Department of Pharmacology, The University of North Carolina, Chapel Hill, NC 27599, USA.

* Corresponding author

Email: timothy_elston@med.unc.edu

Table of contents

Appendix SA. Limiting regimes for 2D bimolecular reactions, 2D $\lambda-\bar{\varrho}$ theory, and investigation of diffusion-limited behaviors.

Figure SA. Comparisons between the deterministic rate equations in 2D and multiple realizations of the particle-based simulation for the simple reversible bimolecular reaction A+B $\leftrightarrow$ C.

Figure SB. Comparisons between the deterministic rate equations in 2D and multiple realizations of the particle-based simulation for the polarity establishment network under non-polarizing, reaction-limited conditions.

Figure SC. Accepted parameters for the yeast polarization model appear supra-diffusive in a 2D context.

Appendix SB. Particle-based simulation implementation.

Appendix SC. Additional details for purely 2D polarization.

Figure SD. Alternative view of the initial time point for the polarization snapshots in Fig. 4.

Figure SE. Representative *H*(*r*) curves for the particle-based and RDE simulations showing similarity in polarization.

Figure SF. Different choices of *r* in *H*(*r*) do not change the qualitative features of the main text results.

Figure SG. Polarity establishment is still slower for the RDE model if equations are seeded with later distributions from the particle-based simulations.

Figure SH. Polarization variability for the RDEs seeded with the *t* = 1 sec distributions from Fig. 5.

Figure SI. Fitting simulations using 2D parameters to the deterministic rate equations.

Table SA. Microscopic parameters and effective macroscopic parameters for reversible/irreversible bimolecular reactions of the form A + B ↔ C, individual fits to each initial condition.

Figure SJ. Bifurcation point identification with linear stability analysis.

Figure SK. Comparisons of deterministic rate equations and the polarity establishment network in a Turing stable regime.

Figure SL. Loss of polarization with increasing membrane diffusivity.

Appendix SD. Derivation and explanation of the quasi-3D injection/ejection integrals.

Appendix SE. Additional details for quasi-3D polarization.

Figure SM. Fitting simulations using quasi-3D parameters to the deterministic rate equations.

Figure SN. Quasi-3D particle-based simulations extended out to 600 seconds.

Figure SO. A reaction-limited quasi-3D RDE simulation of the polarity establishment network versus the corresponding particle-based system.

Figure SP. Quantifying H(r) at t=1800s for pre-polarized q3D-RDEs is a reasonable marker for maintenance vs. loss of polarity.

Figure SQ. Considering the volume-adjusted, two-compartment RDE system makes no qualitative difference for our observations.

Appendix SA. Derivation of limiting behaviors for 2D bimolecular reactions and 2D $\lambda-\bar{\varrho}$ theory.

*Deriving an estimate of the 2D diffusion limit*

Consider a circular capture zone, centered at the origin of a circular domain. Let $\bar{\varrho}$ denote the radius of the capture zone, and *r_max_* denote the radius of the domain. Then, introduce a species with diffusivity *D* with Brownian diffusion. This species, whose non-dimensionalized concentration we denote by *c*(*r*), is absorbed at $\bar{\varrho}$. We fix its concentration at *r_max_*. The system can be described at steady state by:

$$D\left( \frac{d^{2}c}{dr^{2}}+\frac{1}{r}\frac{dc}{dr} \right)=0, \mathrm{for}\bar{\varrho}\leq r\leq r_{max}$$

with boundary conditions:

$$c\left( r \right)=0, \mathrm{for}0\leq r\leq\bar{\varrho}$$

$$c\left( r \right)=C, \mathrm{for}r=r_{max}$$

The general solution to the differential equation is:

$$c\left( r \right)=a_{1}+a_{2}\ln\left( r \right), \mathrm{for}\bar{\varrho}\leq r\leq r_{max}$$

Using the boundary conditions, we can find:

$$a_{1}= -\frac{C\ln\left( \bar{\varrho} \right)}{\ln\left( \frac{r_{max}}{\bar{\varrho}} \right)} \mathrm{and}a_{2}=\frac{C}{\ln\left( \frac{r_{max}}{\bar{\varrho}} \right)}$$

Substituting in the integration constants, we have:

$$c\left( r \right)=\frac{C(\ln\left( r \right)-\ln\left( \bar{\varrho} \right))}{\ln\left( r_{max} \right)-\ln(\bar{\varrho})}, \mathrm{for}\bar{\varrho}\leq r\leq r_{max}$$

The capture rate is the total flux into *r_B_*, which we can use as an estimate for the time scale of the diffusion-limited 2D reaction:

$$Total Flux=J=2\pi D\bar{\varrho}\left. \frac{dc}{dr} \right|_{r=\bar{\varrho}}= \frac{2\pi DC}{\ln\left( r_{max}/\bar{\varrho} \right)}$$

Our estimate of the time scale for the diffusion-limited second-order rate constant is:

$$k_{DL}= \frac{2\pi D}{\ln\left( r_{max}/\bar{\varrho} \right)}$$

*Derivation of the 2D* $\lambda-\bar{\varrho}$ *theory.*

The ratio between the unbinding and binding radii $\alpha\equiv\bar{\sigma}/\bar{\varrho}$ is crucial in defining the appropriate relation that describes the microscopic rate constant *λ* for the reversible bimolecular reaction A + B C. In a manner similar to Lipkova et al [1], we begin by considering a coordinate system with a particle of B placed at the origin. In this frame of reference, the independent diffusion of A and B can be equivalently described as the random walk of a molecule A with diffusion coefficient $D_{A}+D_{B}$. When a molecule A diffuses within a distance $\bar{\varrho}$ of B, it is removed with a rate *λ*. The reverse dissociation reaction can be described with the introduction of new molecules of A at a distance $\bar{\sigma}$ from the origin. In our 2D system, we must work with 2D bimolecular rate constants – to convert a 3D rate constant to a 2D rate constant, we simply divide by the assumed depth of our explicit region (0.00833 μm for this work).

Let *c*(*r*) be the equilibrium concentration of molecules of A at distance *r* from the origin. We non-dimensionalize the problem by defining the reduced quantities:

| $\beta\equiv\bar{\varrho}\sqrt{\frac{\lambda}{D_{A}+D_{B}}}$ | $\hat{r}\equiv\frac{r}{\bar{\varrho}}$ |
| --- | --- |
| $\kappa\equiv\frac{k_{1}}{\left( D_{A}+D_{B} \right)}$ | $\hat{c}=\frac{c}{c_{\infty}}$ |

where we have scaled lengths by $\bar{\varrho}$ and time by $\bar{\varrho}^{2}\left( D_{A}+D_{B} \right)^{-1}$, and normalized the concentration by the bulk concentration:

$$\lim_{r\to\infty} c\left( r \right)=c_{\infty}$$

We consider the case where $\alpha>1$, and ignore $\alpha\leq1$, as the second case turns out to be unusable.

If $\alpha>1$, the dissociation radius $\bar{\sigma}$ is greater than the binding radius $\bar{\varrho}$. The equations describing the microscopic reaction-diffusion system in polar coordinates are:

| $\left( D_{A}+D_{B} \right)\left( \frac{d^{2}c}{dr^{2}}+\frac{1}{r}\frac{dc}{dr} \right)-\lambda c=0, \mathrm{for}r\leq\bar{\varrho}$ |  |
| --- | --- |
| $\left( D_{A}+D_{B} \right)\left( \frac{d^{2}c}{dr^{2}}+\frac{1}{r}\frac{dc}{dr} \right)+Q\left( r-\bar{\sigma} \right)=0, \mathrm{for}r\geq\bar{\varrho}$ |  |

where $Q(r-\bar{\sigma})$ is a Dirac-like distribution describing the production of molecules at $r=\bar{\sigma}$. In terms of the non-dimensional variables, we have:

| $\frac{d^{2}\hat{c}}{d\hat{r}^{2}}+\frac{1}{\hat{r}}\frac{d\hat{c}}{d\hat{r}}-\beta^{2}\hat{c}=0, \mathrm{for}\hat{r}\leq1$ | (Eq. S1) |
| --- | --- |
| $\frac{d^{2}\hat{c}}{d\hat{r}^{2}}+\frac{1}{\hat{r}}\frac{d\hat{c}}{d\hat{r}}+\omega\delta\left( \hat{r}-\alpha\right)=0, \mathrm{for}\hat{r}\geq1$ | (Eq. S2) |

where $\delta\left( \hat{r}-\alpha\right)$ is a Dirac-delta function, and $\omega$ is the rate of creation of molecules at $\hat{r}=\alpha$. The average number of molecules produced by the forward and backward reactions must balance at equilibrium, and therefore we have:

| $2\pi\alpha\omega=\kappa=2\pi\left. \frac{d\hat{c}}{d\hat{r}} \right\vert_{\hat{r}=1}$ | (Eq. S3) |
| --- | --- |

where $\kappa$ represents the association rate constant. This $\kappa$ is directly equal to the flux through the circle of radius 1, because it corresponds to a macroscopic bimolecular rate constant, in contrast to the synthesis term $\omega$. Substituting $\omega= \kappa/(2\pi\alpha)$ into Eq. S2, we obtain:

| $\frac{d^{2}\hat{c}}{d\hat{r}^{2}}+\frac{1}{\hat{r}}\frac{d\hat{c}}{d\hat{r}}+\frac{\kappa\delta\left( \hat{r}-\alpha\right)}{2\pi\alpha}=0, \mathrm{for}\hat{r}\geq1$ | (Eq. S4) |
| --- | --- |

We can write down the general solutions of Eq. S1 and Eq. S4 in the form:

$$\hat{c}\left( \hat{r} \right)=a_{1}I_{0}\left( \beta\hat{r} \right)+a_{2}K_{0}\left( -\beta\hat{r} \right), \mathrm{for}\hat{r}\leq1$$

$$\hat{c}\left( \hat{r} \right)=a_{3}+a_{4}\ln\left( \hat{r} \right)+sgn\left( \alpha-\hat{r} \right)\frac{\kappa}{4\pi}\ln\left( \frac{\hat{r}}{\alpha} \right), \mathrm{for}\hat{r}\geq1$$

where $a_{1}$, $a_{2}$, $a_{3}$, and $a_{4}$ are real constants to be determined, $I_{0}(x)$ and $K_{0}(x)$ are zeroth-order modified Bessel functions of the 1^st^ and 2^nd^ kind, respectively, and $\mathrm{sgn}(x)$ is the signum function.

We specify the constants by using particular boundary conditions, as follows.

| Going out to infinity, the concentration approaches the bulk concentration |  | $\lim_{\hat{r}\to\infty} \hat{c}\left( \hat{r} \right)=1$ |
| --- | --- | --- |
|  |  |  |
| $\hat{c}(0)$ is a finite value |  | $\hat{c}\left( 0 \right)=\hat{c}_{0}$ |
|  |  |  |
| The dimensionless concentration equations must agree at the boundary $\hat{r}=1$ |  | $\hat{c}_{\hat{r}\leq1}\left( 1 \right)=\hat{c}_{\hat{r}\geq1}(1)$ |
|  |  |  |

To determine the integration constants, begin by using the first boundary condition.

$$\lim_{\hat{r} \to\infty} a_{3}+a_{4}\ln\left( \hat{r} \right)+sgn\left( \alpha-\hat{r} \right)\frac{\kappa}{4\pi}\ln\left( \frac{\hat{r}}{\alpha} \right)=1$$

$$a_{3}-1+\lim_{\hat{r} \to\infty} a_{4}\ln\left( \hat{r} \right)-\frac{\kappa}{4\pi}[ln \hat{r}-\ln\alpha]=0$$

$$a_{3}-1+\frac{\kappa}{4\pi}\ln\alpha+\lim_{\hat{r} \to\infty} a_{4}\ln\left( \hat{r} \right)-\frac{\kappa}{4\pi}[ln \hat{r}]=0$$

$$a_{3}-1+\frac{\kappa}{4\pi}\ln\alpha+\lim_{\hat{r} \to\infty} \ln\left( \hat{r} \right)\left[ a_{4}-\frac{\kappa}{4\pi} \right]=0$$

This requires that $a_{4}=\frac{\kappa}{4\pi}$ , which then makes it apparent that $a_{3}=1-\frac{\kappa}{4\pi}\ln(\alpha)$

Next, note that at $\hat{c}\left( 0 \right)=\hat{c}_{0}$, $K_{0}(\beta\hat{r})$ is infinite, requiring that *a_2_* must be zero. Finally, requiring that the two solutions are equal at $\hat{r}=1$ determines *a_1_*:

$$a_{1}I_{0}\left( \beta\right)=1+\frac{\kappa}{4\pi}\ln\left( \frac{\hat{r}}{\alpha} \right)\left[ 1+\mathrm{sgn}\left( \alpha-\hat{r} \right) \right]$$

Since $\hat{r}=1$ and $\hat{\alpha}>1$,

$$a_{1}I_{0}\left( \beta\right)=1+\frac{\kappa}{2\pi}\ln\left( \frac{1}{\alpha} \right)$$

$$a_{1}=\frac{1-\frac{\kappa}{2\pi}\ln\left( \alpha\right)}{I_{0}\left( \beta\right)}$$

Substitution of all constants into the equations for $\hat{c}\left( \hat{r} \right)$ yields:

$$\hat{c}\left( \hat{r} \right)=\frac{I_{0}\left( \beta\hat{r} \right)}{I_{0}(\beta)}\left[ 1-\frac{\kappa\ln\left( \alpha\right)}{2\pi} \right], \mathrm{for}\hat{r}\leq1$$

$$\hat{c}\left( \hat{r} \right)=1+\frac{\kappa}{4\pi}\ln\left( \frac{\hat{r}}{\alpha} \right)\left[ 1+\mathrm{sgn}\left( \alpha-\hat{r} \right) \right], \mathrm{for}\hat{r}\geq1$$

To get an expression for $\kappa$ in terms of *β*, which contains our microscopic rate *λ*, we evaluate the derivative $\hat{c}'\left( \hat{r} \right)$ at $\hat{r}=1$ and set it equal to $\kappa$/2π.

$$\kappa=\frac{2\pi\beta\frac{I_{1}(\beta)}{I_{0}(\beta)}}{\left( 1+\ln\left( \alpha\right)\beta\frac{I_{1}\left( \beta\right)}{I_{0}\left( \beta\right)} \right)}, \alpha>1$$

Note that, if we take the limit as *α* → 1 and hold *β* constant, *κ* increases. This is consistent with the idea that, if the probability of reacting within $\bar{\varrho}$ is kept the same, as we shrink the release radius, molecules of A will react with B more rapidly.

Returning to experimentally measurable quantities *k_1_*, *D_A_*, *D_B_*, and the model parameters $\bar{\varrho}$, $\bar{\sigma}$, and *λ:*

$$k_{1}=\left( D_{AB} \right)\frac{2\pi\bar{\varrho}\sqrt{\frac{\lambda}{D_{AB}}}\left( \frac{I_{1}\left( \bar{\varrho}\sqrt{\frac{\lambda}{D_{AB}}} \right)}{I_{0}\left( \bar{\varrho}\sqrt{\frac{\lambda}{D_{AB}}} \right)} \right)}{\left( 1+\ln\left( \frac{\bar{\sigma}}{\bar{\varrho}} \right)\bar{\varrho}\sqrt{\frac{\lambda}{D_{AB}}}\frac{I_{1}\left( \bar{\varrho}\sqrt{\frac{\lambda}{D_{AB}}} \right)}{I_{0}\left( \bar{\varrho}\sqrt{\frac{\lambda}{D_{AB}}} \right)} \right)}, \alpha>1$$

where $D_{AB}\equiv D_{A}+D_{B}$, and $I_{0}\left( x \right)$ and $I_{1}\left( x \right)$ are the zeroth- and first-order modified Bessel functions of the first kind. Although there is no analytic inverse of $I_{\nu}\left( x \right)$, we can numerically solve for the microscopic rate constant *λ*.

*Validating the 2D* $\lambda-\bar{\varrho}$ *theory.*

We compared our particle-based simulations, using *2D* $\lambda-\bar{\varrho}$ theory, to the deterministic rate equations for a reversible bimolecular reaction in 2D. We numerically computed an ODE solution for a given parameter set and compared it to several realizations of particle-based simulations.


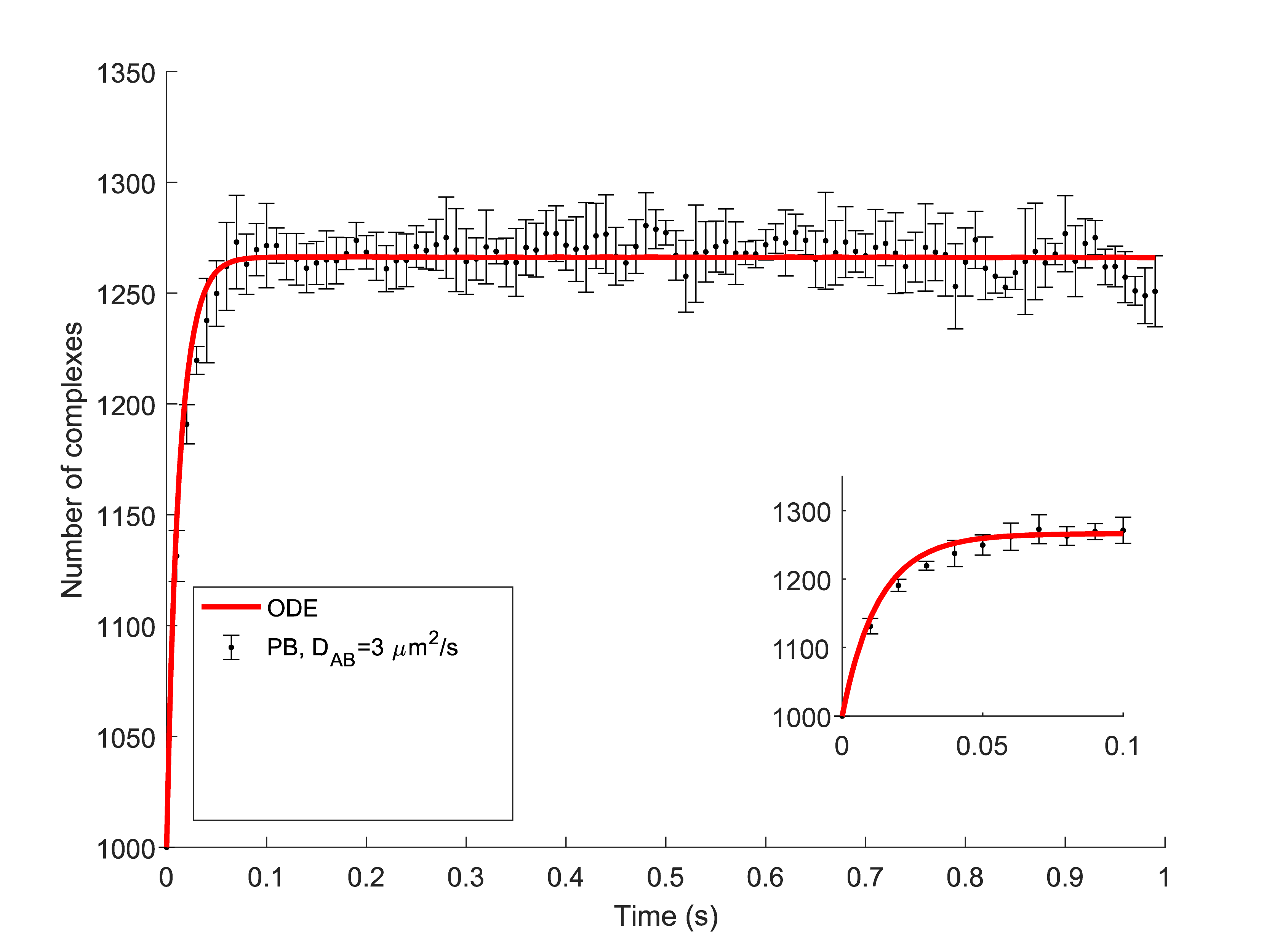


**Fig SA. Comparisons between the deterministic rate equations in 2D and multiple realizations of the particle-based simulation for the simple reversible bimolecular reaction A+B** $\boldsymbol{\leftrightarrow}$ **C.** The error bars for the particle-based simulations represent the mean±1s.d., n=5.

Finally, to ensure that our microscopic approach was consistent with macroscopic theory in a more complicated reaction network, we performed particle-based simulations and compared the kinetic profiles of each molecular species to equivalent, deterministic reaction-diffusion simulations. Here our studies were done in non-polarizing conditions to ensure that polarity establishment – which can effect the kinetics and steady-state levels of the various molecular populations – did not interfere with our comparisons. To ensure such conditions, we used a high diffusivity (*D_m_* = *D_c_* = 1.5 μm^2^/s), which also alleviates complications that would arise within the diffusion-limited regime.


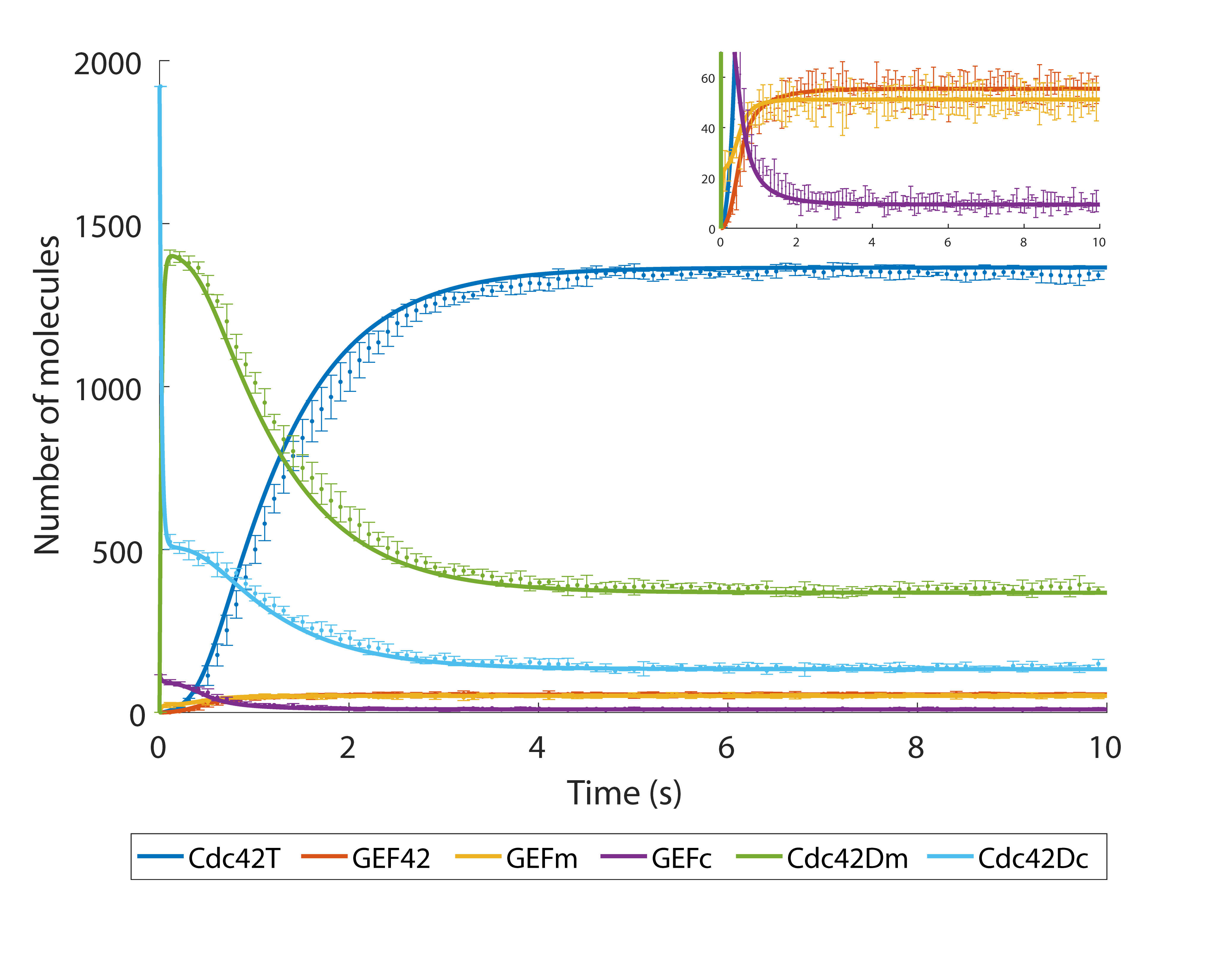


**Fig SB. Comparisons between the deterministic rate equations in 2D and multiple realizations of the particle-based simulation for the polarity establishment network under reaction-limited, non-polarizing conditions.** The error bars for the particle-based simulations represent the mean±1s.d., n=5.

*Consideration of the 2D diffusion limit.*

In the diffusion-limited regime, the particle-based simulations of even simple reversible bimolecular reactions are no longer consistent with macroscopic theory. As mentioned in the main text, this is because of loss of validity of the well-stirred assumption (which includes spatiotemporal dependence of the macroscopic rate "constant" *k*) and the 2D $\lambda-\bar{\varrho}$ theory. To assess whether the diffusion-limited conditions are relevant to published parameters for the yeast polarization network, we can use our estimate for the time scale of 2D diffusion-limited reactions, $k_{DL}=2\pi D/\ln(r_{max}/\bar{\varrho})$ (Fig S3). Several of the rate constants appear to exceed the diffusion limit. However, as we note in the main text, this calculation does not take into account the effect of particle exchange between the membrane and cytoplasm, which can lead to significantly larger effective rate constants.


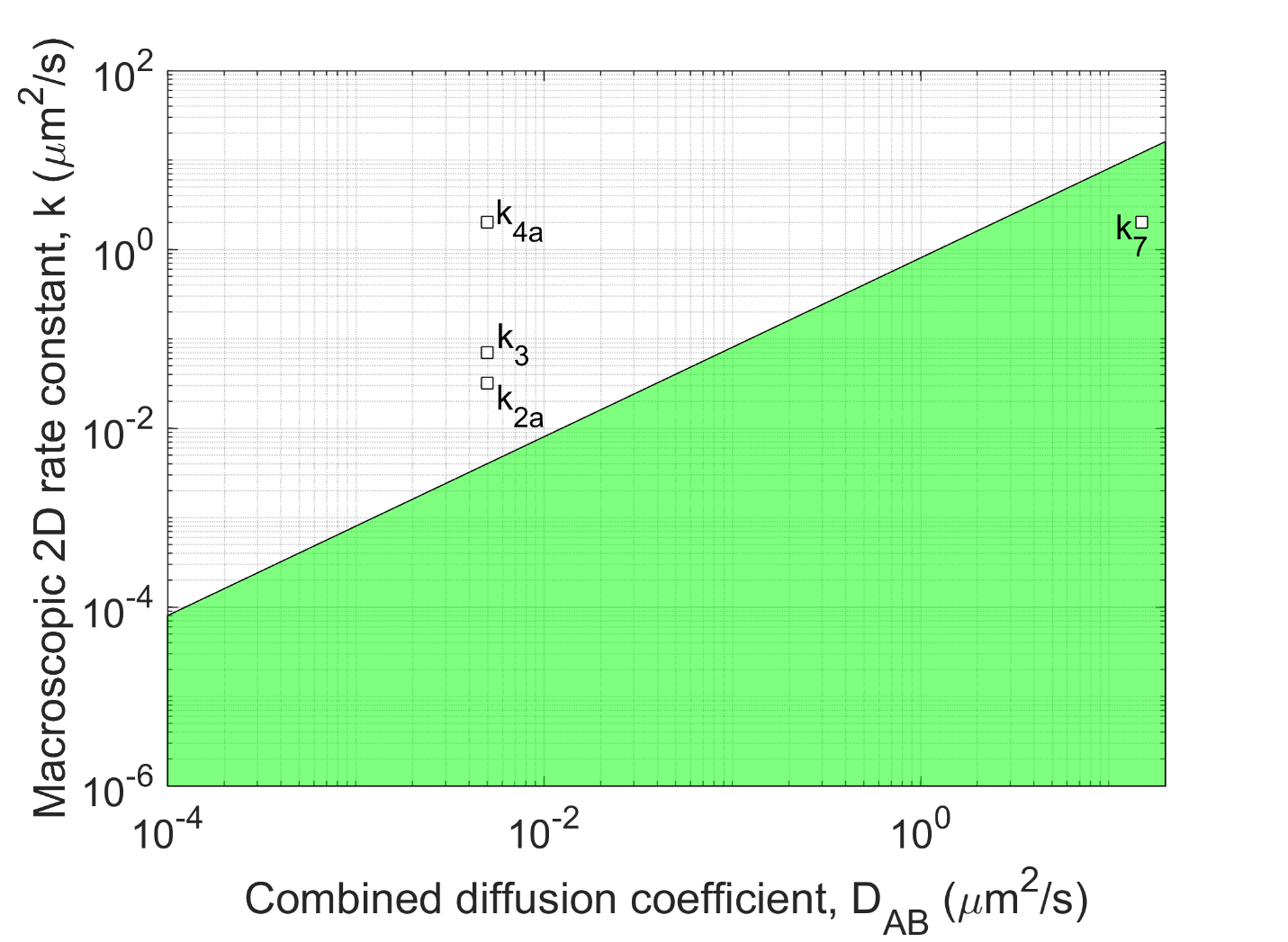


**Fig SC. Accepted parameters for the yeast polarization model appear supra-diffusive in a 2D context.** The blank line is an estimate of the macroscopic rate constant using $2\pi D/\ln(r_{max}/\bar{\varrho})$. This neglects membrane-cytoplasm exchange. It is important to remember that, strictly, there is no well-defined diffusion-limited rate constant. Parameters: *r*_max_ = 2.5 µm, $\bar{\varrho}$ = 0.05 µm. Simulations were conducted on a *L* = 5 µm domain.

Appendix SB. Particle-based simulation implementation.

Our modeling approach for the reactions describing polarity establishment proceeds using the following steps.

Initialization

1. Compute the total number of molecules based on cellular volume and concentration.
2. *Tabulate integrals for diffusion in and out of the implicit domain.
3. Calculate the $P_{\lambda}$ value for each macroscopic bimolecular rate constants.
4. *Estimate the initial number of molecules to explicitly model.
5. Initialize the data export matrix.
6. Initialize all molecules in a matrix, and mark molecules as implicit when needed.

Simulation Loop

1. *Check how many of each molecule type are currently explicitly simulated.
2. Draw random numbers for diffusion, dissociation, membrane interaction, and GTP hydrolysis.
3. Compute a collision matrix for bimolecular reaction checks.
4. Check all reactions, update molecule states, and set event flags as appropriate.
   1. Bimolecular association reactions.
   2. Unimolecular GEF42 dissociation reaction.
   3. Bimolecular, catalyzed GDP→GTP exchange.
   4. Unimolecular GTP hydrolysis reaction.
   5. Unimolecular membrane association and dissociation reactions.
5. Update positions due to Brownian diffusion as well as complex association/dissociation.
6. *Inject/eject particles to/from the explicit simulation domain.
7. Overwrite the current system state with new system state.
8. Store current system state to output matrix if matching to specified time resolution.
9. Repeat from i.

*Only if performing quasi-3D simulations.

Appendix SC. Additional details for purely 2D polarization.


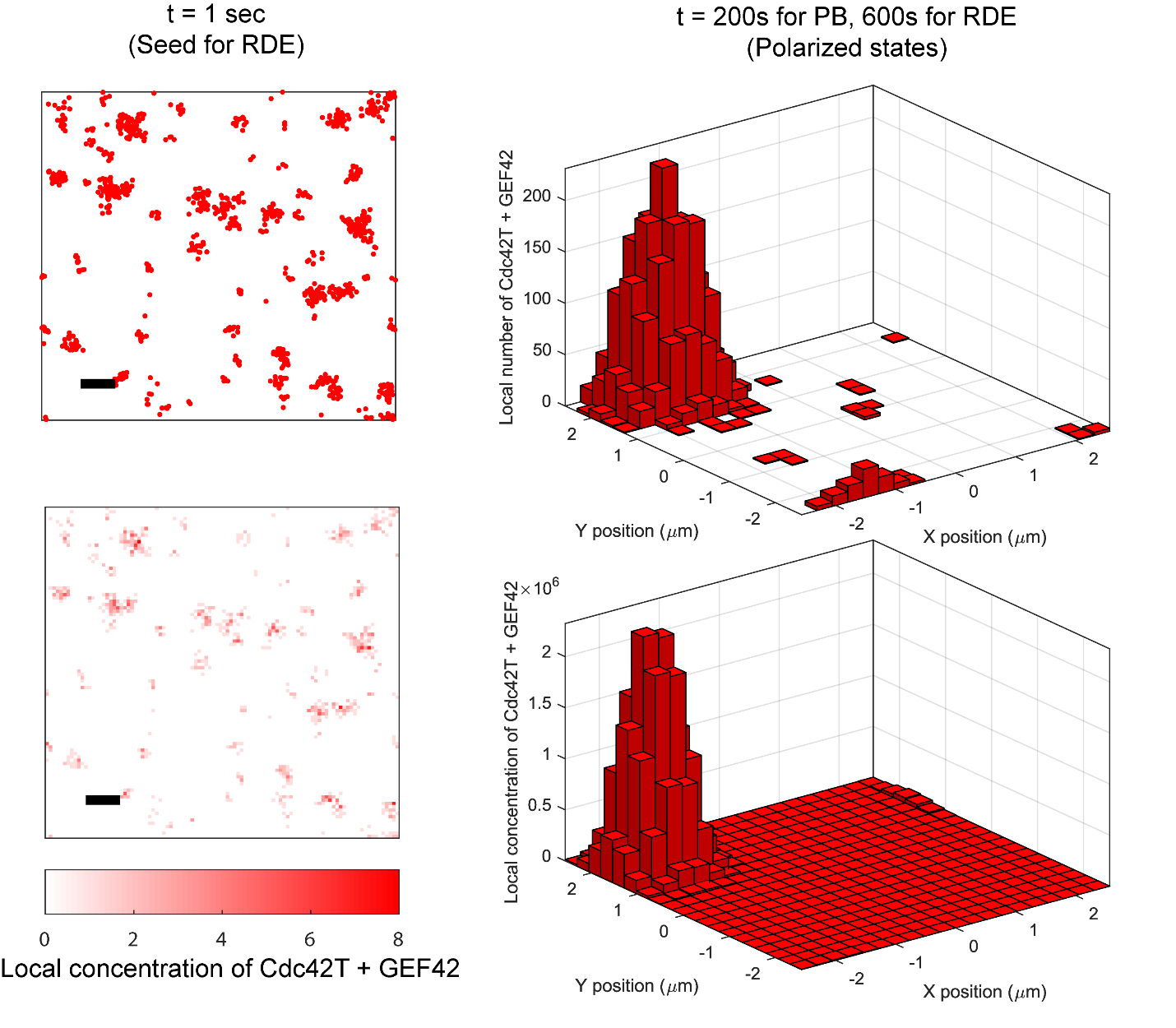


**Fig SD. Alternative view of the initial time points for the polarization snapshots in Fig. 4.** The initial RDE snapshot has been re-scaled on the color axis to show equivalence with the particle-based data.


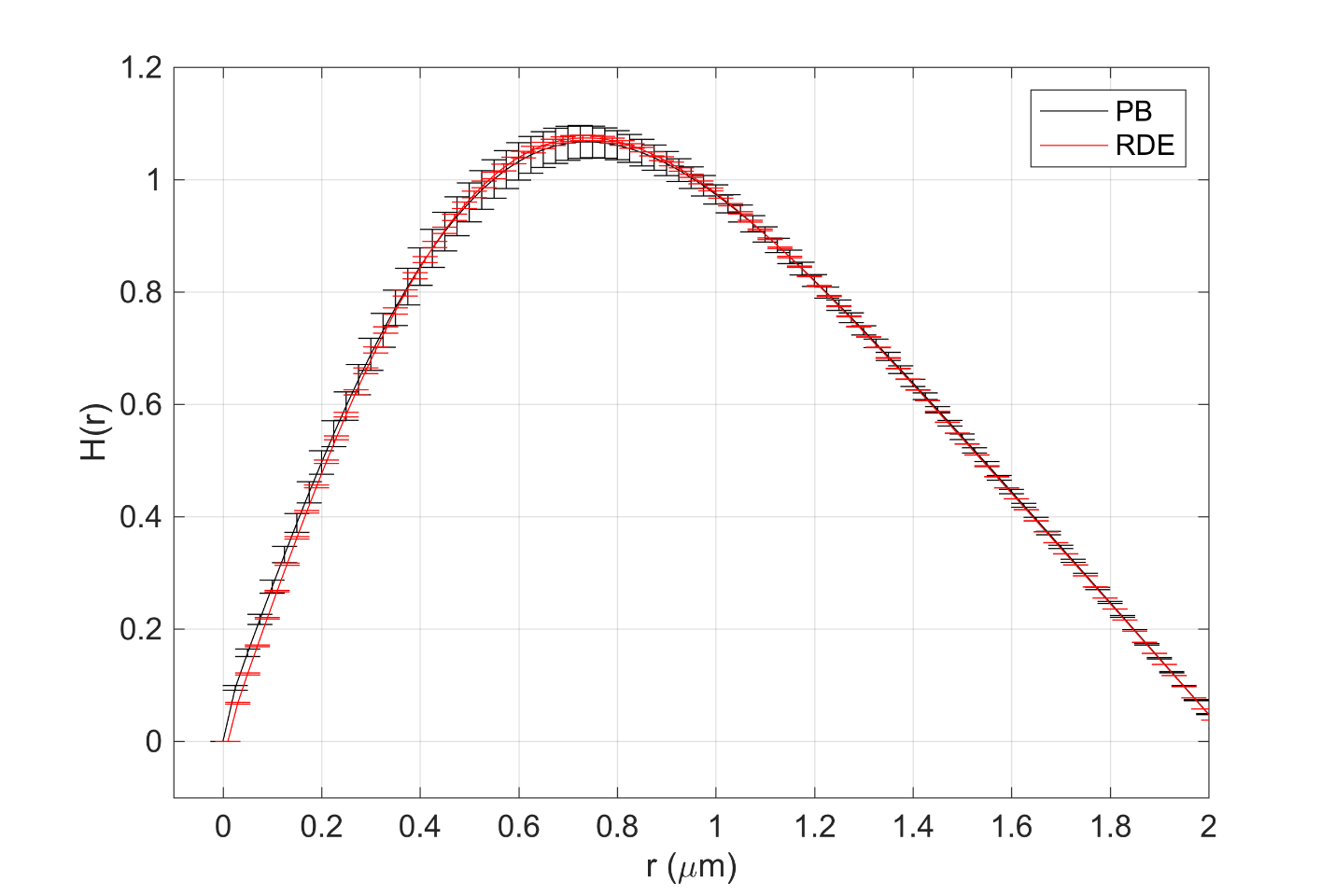


**Fig SE. Representative H(r) curves for the particle-based and RDE simulations showing similarity in polarization.** Quantification was performed at steady-state polarity (t=200s for particle-based simulation, t=600s for RDE simulation.) Uses the parameters in Fig. 6a/b with *N*_Cdc42_ = 1921 and *N*_GEF_ = 116.


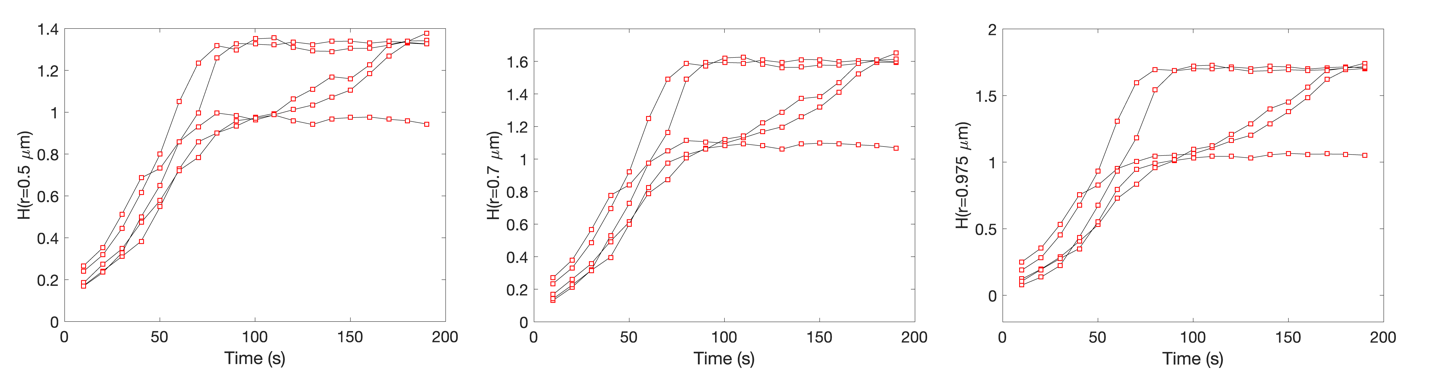


**Fig SF. Different choices of *r* in *H*(*r*) do not change the qualitative features of the main text results.** Fig. 5a has been replicated here alongside versions with *H*(*r* = 0.7 μm) and *H*(*r* = 0.975 μm).

**
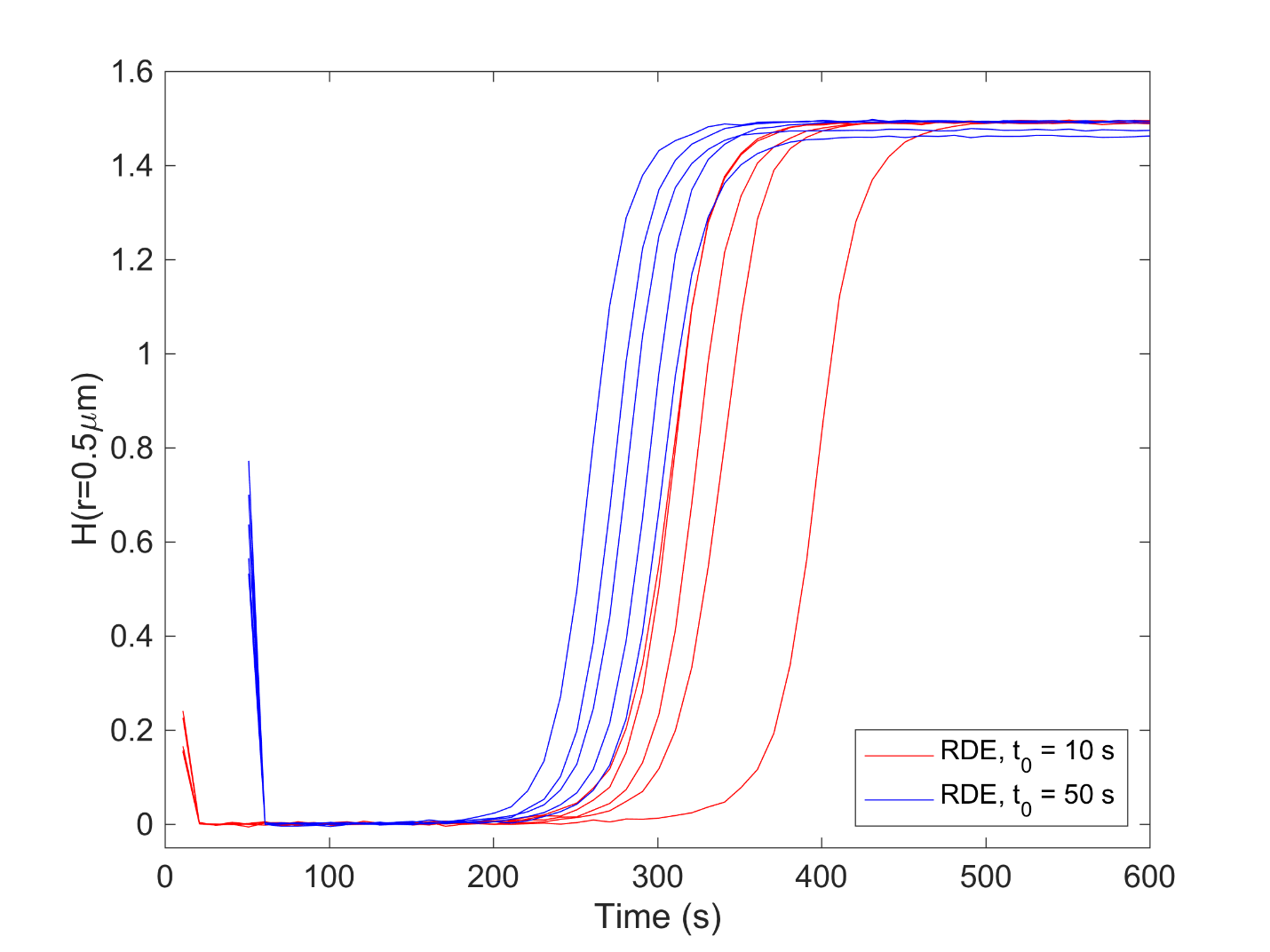
**

**Fig SH. Polarity establishment is still slower for the RDE model if equations are seeded with later distributions from the particle-based simulations.** The corresponding particle-based simulations are all polarized by 200s under these conditions, showing that the RDEs are consistently slow in comparison to the particle-based simulations.


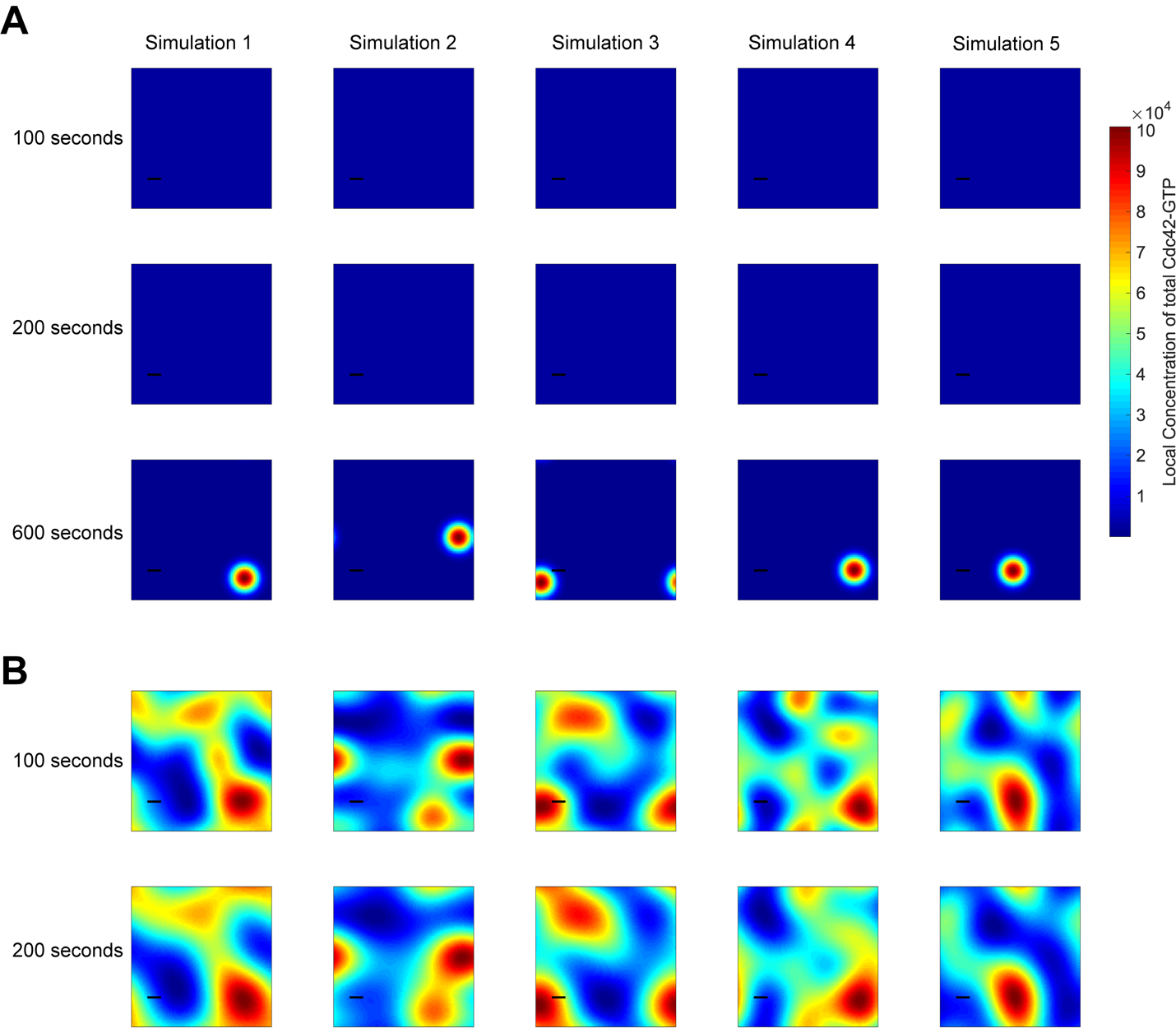


**Fig SI. Polarization variability for the RDEs seeded with the *t* = 1 sec distributions from Fig. 5.** Distributions of total Cdc42-GTP en route to polarization. (A) Distributions all scaled to the same color map. (B) Distributions on the bottom individually scaled to show heterogeneity exists even though the magnitude of variation is small.

Note that none of the RD equations polarize by the 200 second time-point. Examining self-scaled distributions shows underlying non-homogeneous behavior en-route to polarized states, but these are very shallow concentration differences.

**
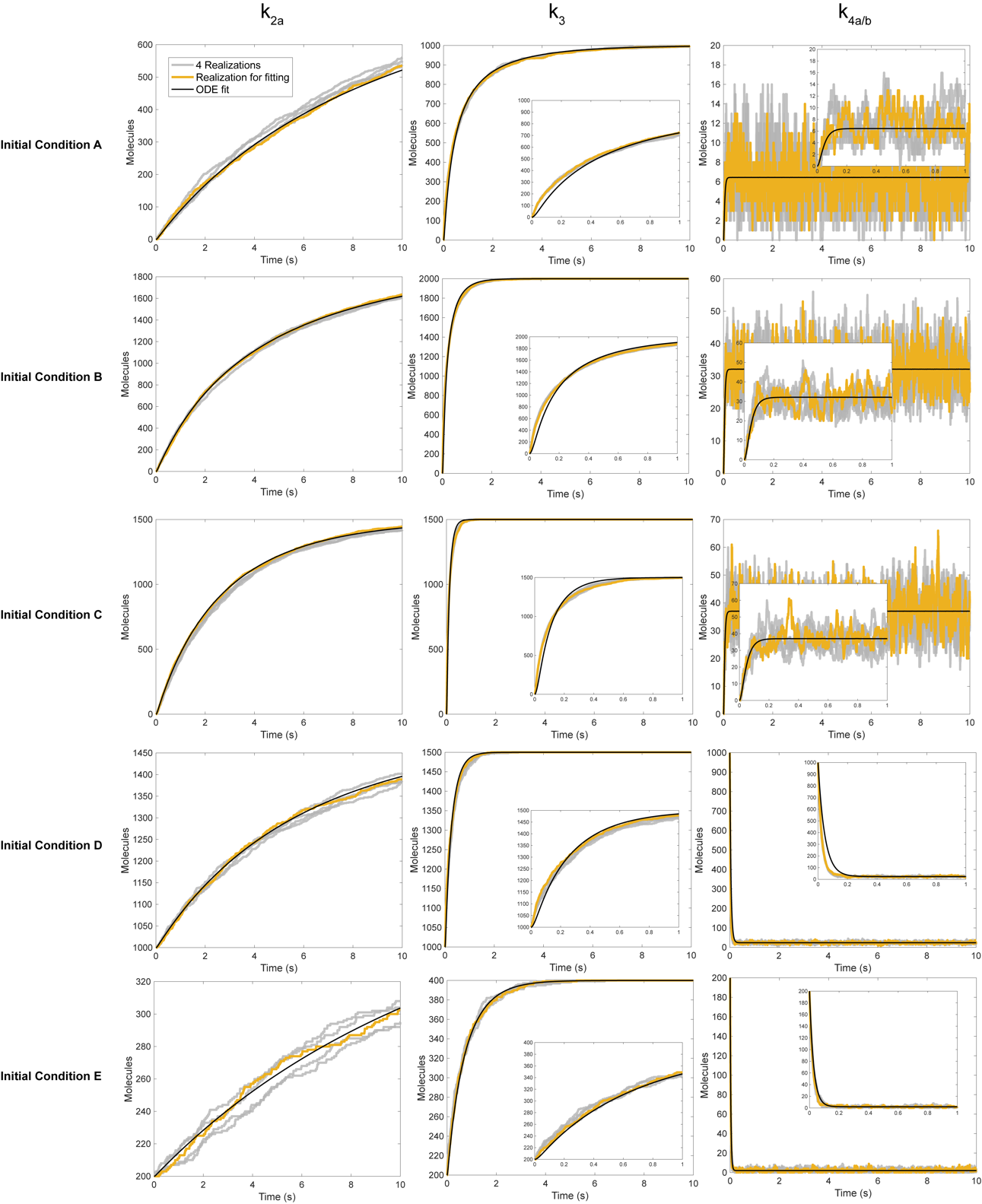
**

**Figure SI. Fitting simulations using 2D parameters to the deterministic rate equations.** Only the amount of product C formed is shown. Each ODE was fit to a single realization (gold). Additional realizations of each condition are provided to show the amount of intrinsic variability (gray).

**Table SA**. **Microscopic parameters and effective macroscopic parameters for reversible/irreversible bimolecular reactions of the form A + B ↔ C, individual fits to each initial condition.**

|  | Reaction | IC | Membrane on/off rates | | | | Microscopic parameters | | Fitted | |
| --- | --- | --- | --- | --- | --- | --- | --- | --- | --- | --- |
|  |  |  | k_Aon_ (1/s) | k_Aoff_ (1/s) | k_Bon_ (1/s) | k_Boff_ (1/s) | λ  (1/s) | k_r_  (1/s) | k_f_  (2D: μm^2^/s;  3D: uM^-1^/s) | k_r_ (1/s) |
| Parameters for quasi-3D simulation | Cdc42D_m_ + BemGEF_m_  → Cdc42T | A | 36 | 0.65 | 10 | 10 | 5.30 | - | 0.1664 | - |
|  |  | B |  |  |  |  |  | - | 0.1584 | - |
|  |  | C |  |  |  |  |  | - | 0.1599 | - |
|  |  | D |  |  |  |  |  | - | 0.1634 | - |
|  |  | E |  |  |  |  |  | - | 0.1679 | - |
|  | Cdc42D_m_ + BemGEF42 → Cdc42T | A | 36 | 0.65 | - | - | 15.7 | - | 0.14444 | - |
|  |  | B |  |  |  |  |  | - | 0.1484 | - |
|  |  | C |  |  |  |  |  | - | 0.2054 | - |
|  |  | D |  |  |  |  |  | - | 0.1669 | - |
|  |  | E |  |  |  |  |  | - | 0.1374 | - |
|  | BemGEF_m_ + Cdc42T  ↔ BemGEF42 | A | 10 | 10 | - | - | 8245 | 10 | 0.9943 | 0.492 |
|  |  | B |  |  |  |  |  |  | 1.0043 | 0.480 |
|  |  | C |  |  |  |  |  |  | 1.2541 | 0.546 |
|  |  | D |  |  |  |  |  |  | 0.3997 | 0.179 |
|  |  | E |  |  |  |  |  |  | 0.3098 | 0.157 |
| Parameters for purely 2D simulation | Cdc42D_m_ + BemGEF_m_  → Cdc42T | A | 36 | 13 | 10 | 40 | 5.30 | - | 0.0367 | - |
|  |  | B |  |  |  |  |  | - | 0.0392 | - |
|  |  | C |  |  |  |  |  | - | 0.0411 | - |
|  |  | D |  |  |  |  |  | - | 0.0398 | - |
|  |  | E |  |  |  |  |  | - | 0.0432 | - |
|  | Cdc42D_m_ + BemGEF42  → Cdc42T | A | 36 | 13 | - | - | 178 | - | 0.162 | - |
|  |  | B |  |  |  |  |  | - | 0.181 | - |
|  |  | C |  |  |  |  |  | - | 0.220 | - |
|  |  | D |  |  |  |  |  | - | 0.192 | - |
|  |  | E |  |  |  |  |  | - | 0.166 | - |
|  | BemGEF_m_ + Cdc42T  ↔ BemGEF42 | A | 10 | 40 | - | - | 9.60 | 40 | 0.0570 | 32.8 |
|  |  | B |  |  |  |  |  |  | 0.0586 | 33.3 |
|  |  | C |  |  |  |  |  |  | 0.0595 | 32.8 |
|  |  | D |  |  |  |  |  |  | 0.0379 | 21.9 |
|  |  | E |  |  |  |  |  |  | 0.0593 | 36.1 |

D_m_ = 0.0025 μm^2^/s and D_c_ = 15 μm^2^/s were used in the simulations. The domain area was 64 μm^2^. Fits to rate equations were performed to five separate particle-based simulations using different initial conditions (ICs). The fitted rates were then averaged. Initial conditions were as follows. **Set A**: A_0_ = 1000, B_0_ = 1200, C_0_ = 0. **Set B**: A_0_ = 3000, B_0_ = 2000, C_0_ = 0. **Set C**: A_0_ = 4500, B_0_ = 1500, C_0_ = 0. **Set D**: A_0_ = 2000, B_0_ = 500, C_0_ = 1000. **Set E**: A_0_ = 200, B_0_ = 800, C_0_ = 200.


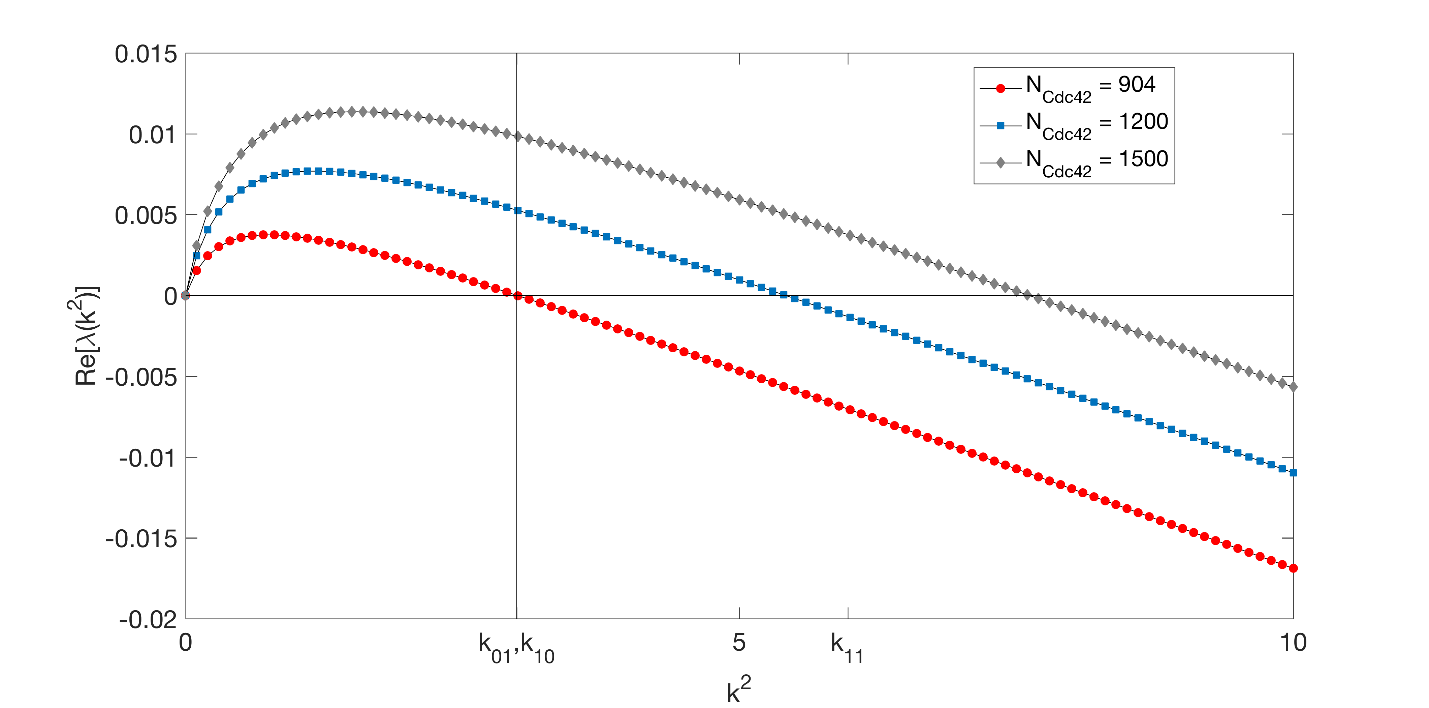


**Figure SJ. Bifurcation point identification with linear stability analysis.** The dispersion relation Re[*λ*(k^2^)] is plotted against wave numbers k^2^. The smallest number of Cdc42 molecules such that both Re[*λ*(k_01_^2^)] > 0 and Re[*λ*(k_01_^2^)] > 0 is the bifurcation point. This result corresponds to Fig. 6a.


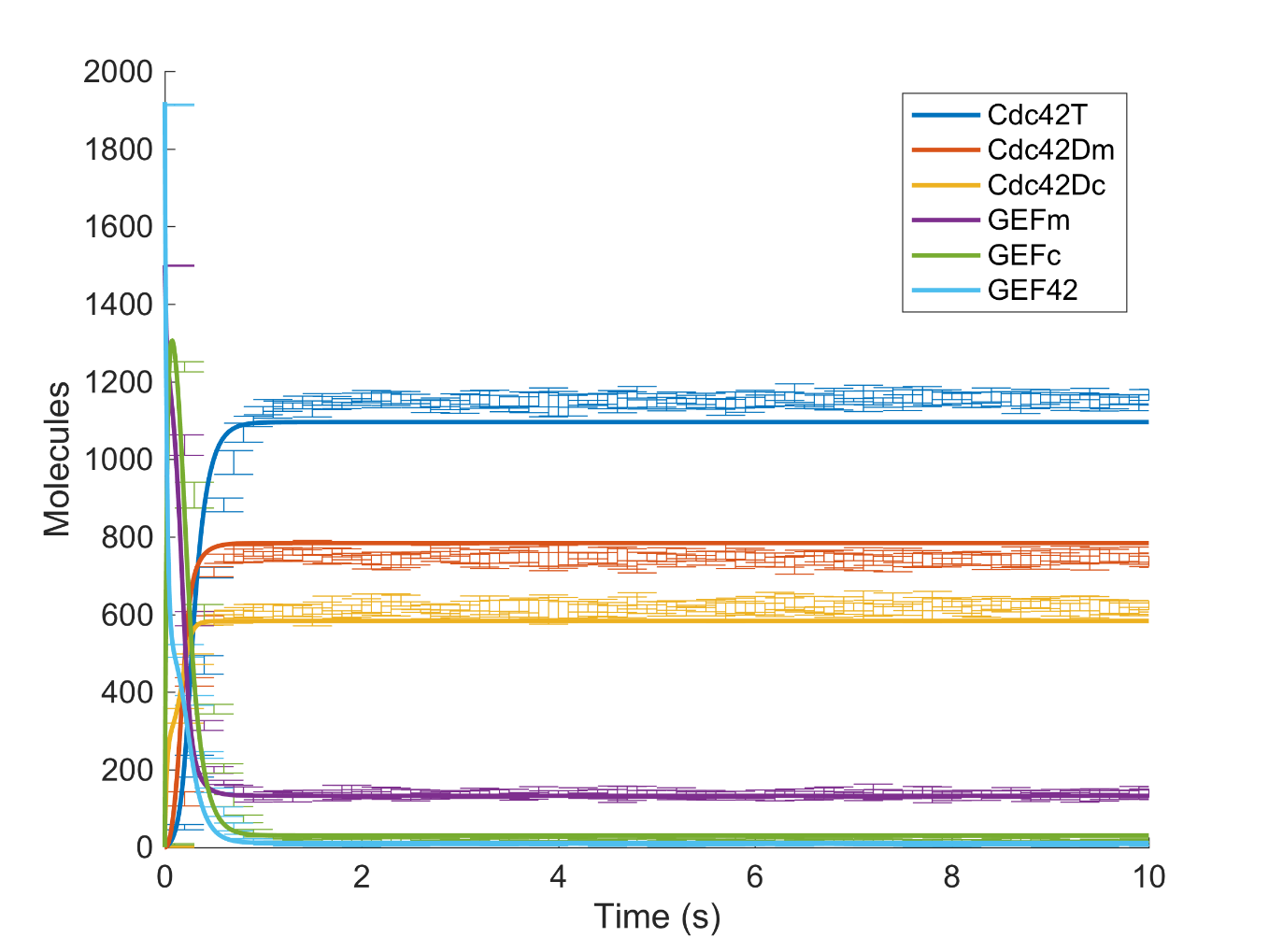


**Figure SK. Comparisons of deterministic rate equations in 2D and the polarity establishment network in a Turing stable regime.** Linear stability analysis was used to identify a high [Cdc42] regime, consistent with parameters used in Fig. 6b. Here, *N*_Cdc42_ = 1921 and *N*_GEF_ = 1500. All other parameters are the same as in the main text. Error bars are the mean±1s.d. from *n*=5 realizations of the particle-based simulation, solid lines are the solutions of the ordinary differential equations.

*
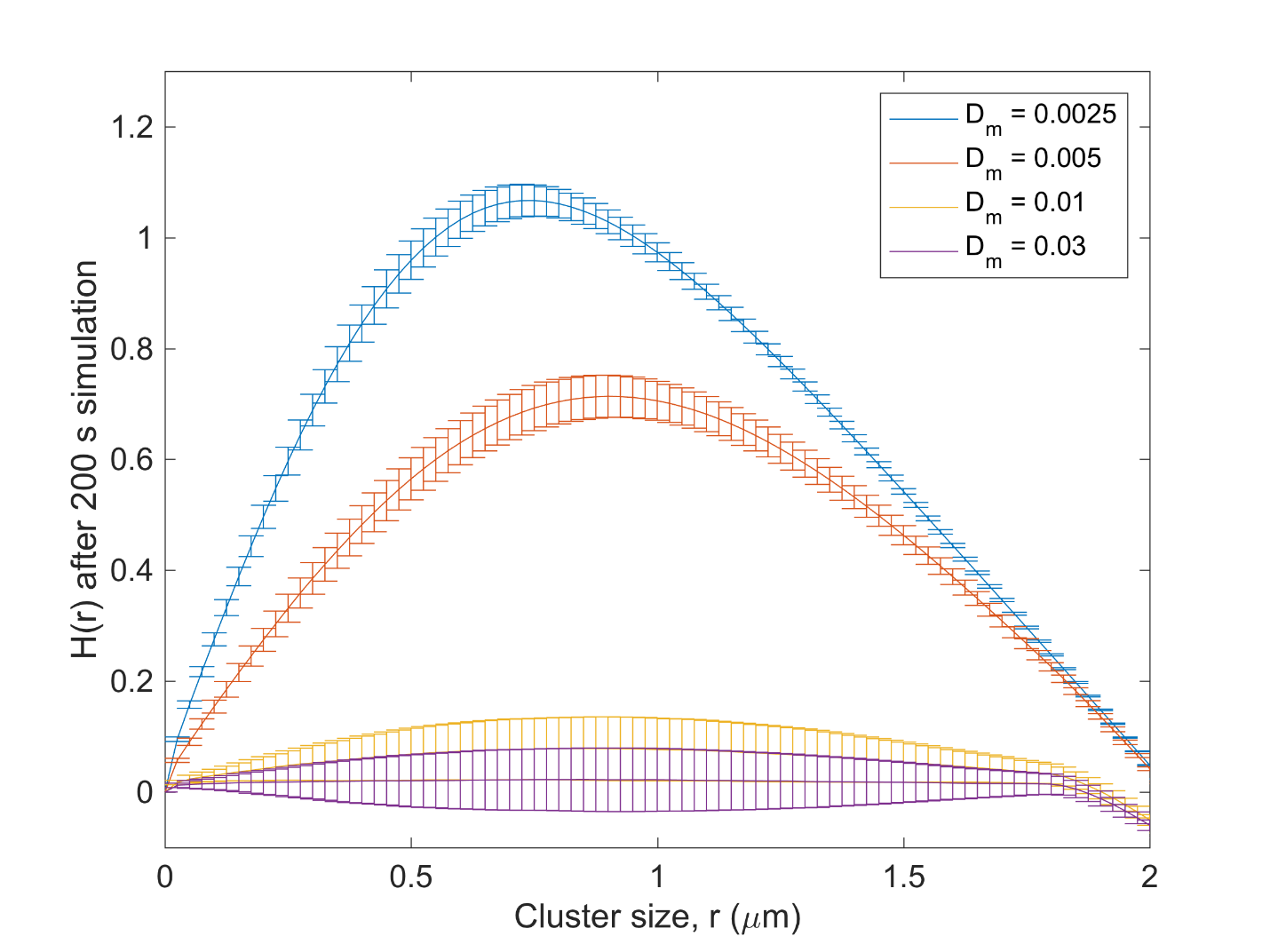
*

**Figure SL. Loss of polarization with increasing membrane diffusivity.** The parameters in Fig. 6a/b with *N*_Cdc42_ = 1921 and *N*_GEF_ = 116, were used to create the results for D_m_ *=* 0.0025 µm^2^/s. We then increased the diffusivity on the membrane while keeping all other parameters fixed. Each curve represents the mean±1s.d. from *n*=5 realizations.

Appendix SD. Derivation and explanation of the quasi-3D injection/ejection integrals.

*Particle injection and ejection.*

We begin by considering the diffusional probability density defined by the diffusion equation in one dimension. We assume that diffusing particles are not confined – while this is not truly the case, we demonstrate that this assumption turns out to be reasonable.

$$\partial_{t}p\left( z,t | x_{0},t_{0} \right)=D\partial_{z}^{2}p(z,t|z_{0},t_{0})$$

$$p\left( z, t_{0} | z_{0},t_{0} \right)=\delta(z-z_{0})$$

$$p\left( z\to\pm\infty,t | z_{0},t_{0} \right)=0$$

The corresponding Green’s function solution is:

| $p\left( z,t \vert z_{0},t_{0} \right)=\frac{1}{\sqrt{4\pi D\left( t-t_{0} \right)}}\exp\left[ -\frac{\left( z-z_{0} \right)^{2}}{4D(t-t_{0})} \right]$ | (Eq. S5) |
| --- | --- |

If we consider diffusion distributions p_i_ emerging from Dirac-Delta distributions at z_i_ within the cytoplasm, then each probability distribution p_i_ satisfies Eq. S5 at each time step. We further assume that the explicit and implicit cytoplasmic domains are perfectly well-mixed along the z-axis. We rewrite this solution after substituting Δ*t* = *t – t_0_* and also redefine *z* = *z – z_0_* for convenience.

$$p\left( z \right)=\frac{1}{\sqrt{4\pi D\Delta t}}\exp\left[ -\frac{z^{2}}{4D\Delta t} \right]$$

To compute the probability of diffusing from one compartment to the other, we integrate the probability distribution *p* ranging from the minimal to the maximal distance that must be traveled. We either assume that diffusion must halt at the maximal distance (subsequently denoted the ‘exact case’), or include diffusional probabilities that bypass the maximal distance towards infinity (subsequently denoted the ‘approximate case’). Throughout the manuscript, we use the exact case – but the approximate case can be intuitively examined for correctness, so we present it here.

| $P_{inj}\left( z_{i} \right)=\int_{z_{impl}-z}^{z_{max}-z} \frac{1}{\sqrt{4\pi D\Delta t}}\exp\left[ -\frac{z^{2}}{4D\Delta t} \right]dz \approx\int_{z_{impl}-z}^{\infty} \frac{1}{\sqrt{4\pi D\Delta t}}\exp\left[ -\frac{z^{2}}{4D\Delta t} \right]dz$ |
| --- |
|  |
| $P_{inj}\left( z \right)=\frac{1}{2}\left[ \mathrm{erf} \left( \frac{z_{max}-z}{\sqrt{4D\Delta t}} \right)-\mathrm{erf} \left( \frac{z_{impl}-z}{\sqrt{4D\Delta t}} \right) \right]\approx\frac{1}{2}\left[ 1-\mathrm{erf} \left( \frac{z_{impl}-z}{\sqrt{4D\Delta t}} \right) \right]$ |
|  |
| $P_{ejc}\left( z \right)=\int_{z-z_{impl}}^{z} \frac{1}{\sqrt{4\pi D\Delta t}}\exp\left[ -\frac{z^{2}}{4D\Delta t} \right]dz \approx\int_{z-z_{impl}}^{-\infty} \frac{1}{\sqrt{4\pi D\Delta t}}\exp\left[ -\frac{z^{2}}{4D\Delta t} \right]dz$ |
|  |
| $P_{ejc}\left( z \right)=\frac{1}{2}\left[ \mathrm{erf} \left( \frac{z}{\sqrt{4D\Delta t}} \right)-\mathrm{erf} \left( \frac{-(z_{impl}-z)}{\sqrt{4D\Delta t}} \right) \right]\approx\frac{1}{2}\left[ 1-\mathrm{erf} \left( \frac{-(z_{impl}-z)}{\sqrt{4D\Delta t}} \right) \right]$ |
|  |

The term *z* is the depth of the particle in coordinates where z = 0 is the bottom of the implicit reservoir, *z_impl_* is the depth marking the interface between the implicit and explicit domains, and *z_max_* is the total height of the implicit and explicit domains, as illustrated in Fig. 7.

Starting from the approximate forms, we note that if we consider a slice at the explicit-implicit boundary *z* = *z_impl_*, we have *P_inj_ = P_ejc_ =* 0.5, which reflects that a Brownian particle is equally likely to diffuse up or diffuse down. Cutting the integrals at the cytoplasmic boundaries alters this probability slightly, but not by a noticeable amount. We choose to use the exact integrals since there is very minimal computational advantage to using the approximate form.

Appendix SE. Additional details for quasi-3D polarization.

**
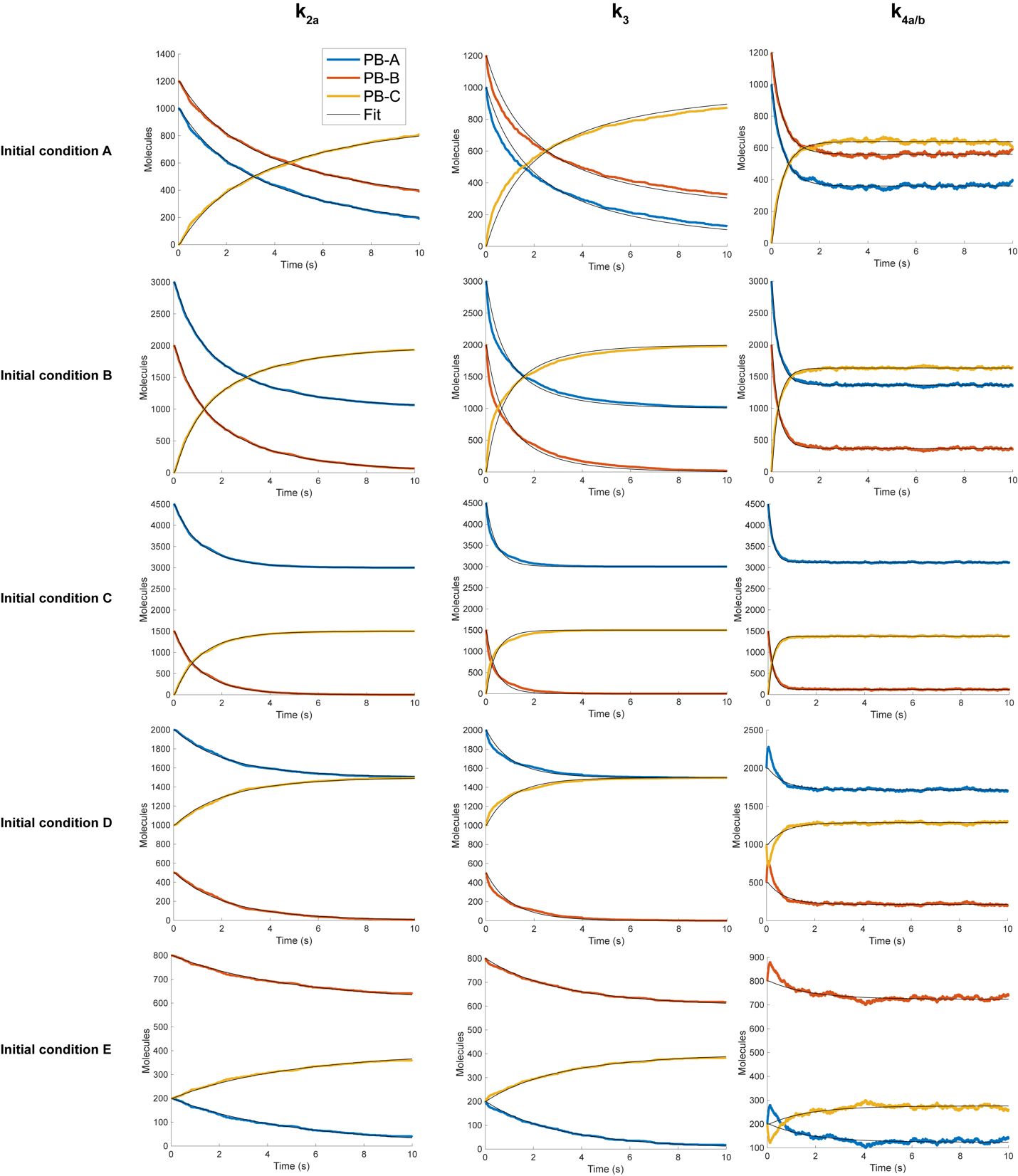
**

**Fig SM. Fitting simulations using quasi-3D parameters to the deterministic rate equations.**


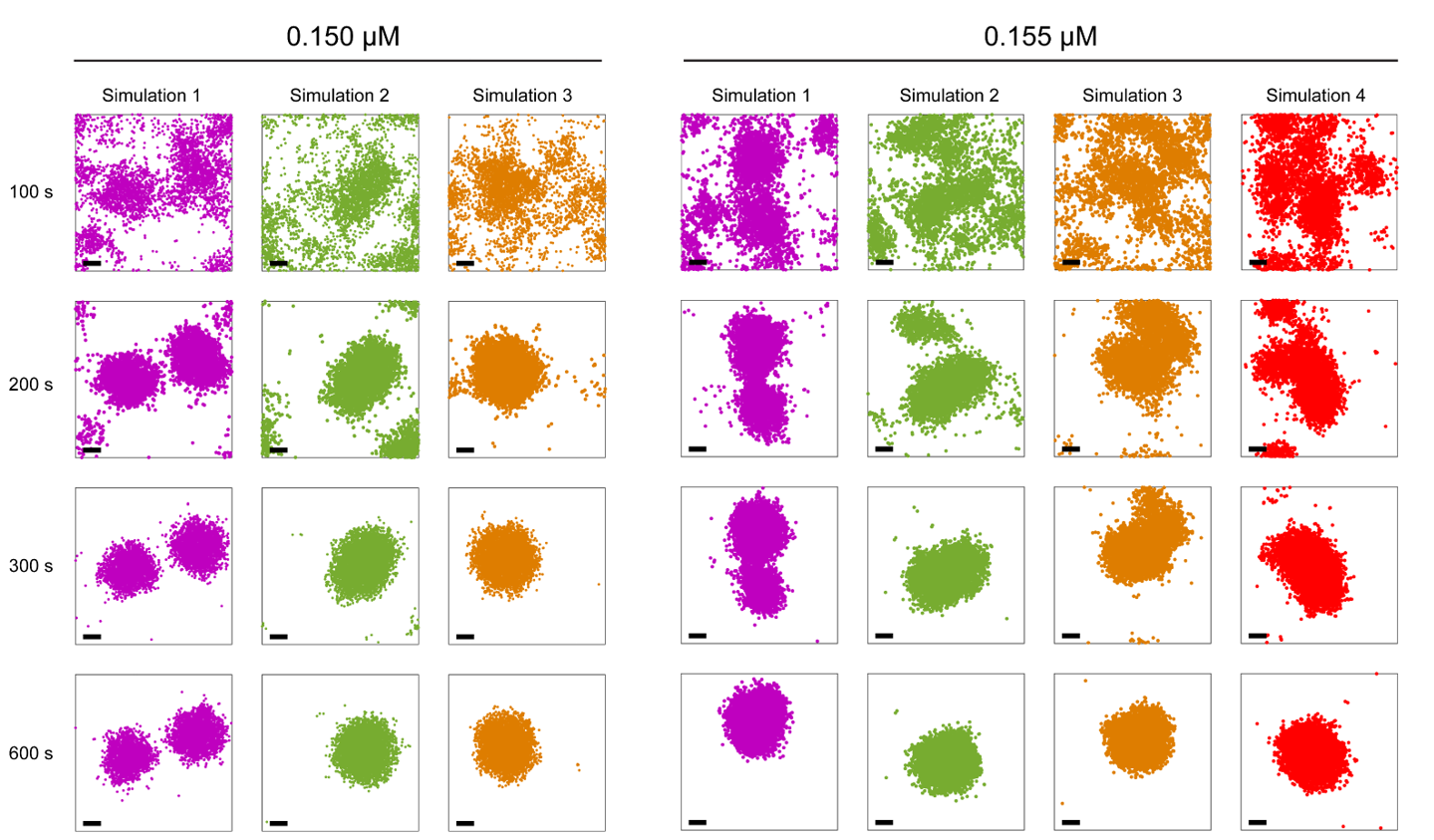


**Fig SN. Quasi-3D particle-based simulations extended out to 600 seconds.** Shown are snapshots of total Cdc42-GTP, scale bar 1.0 μm. [Cdc42] = 0.150 μM.


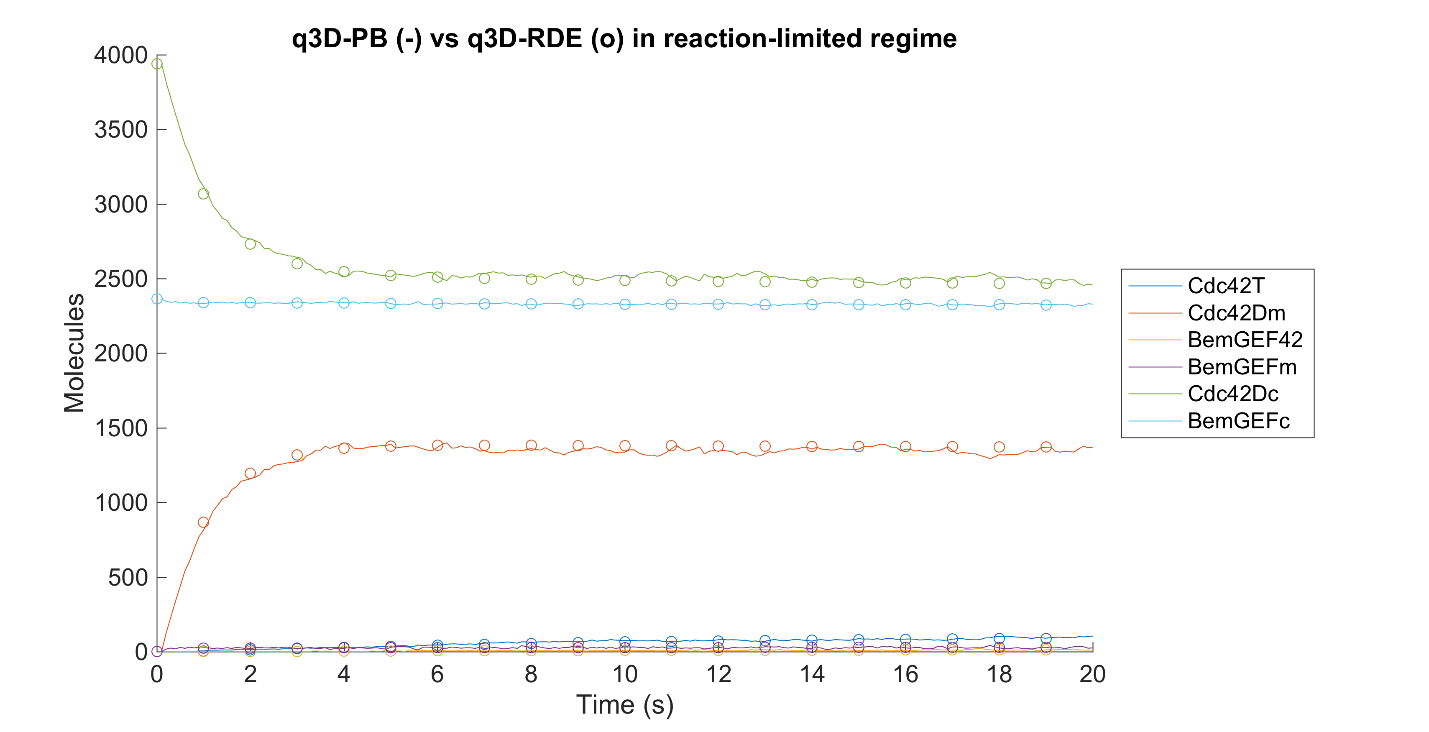


**Fig SO. A reaction-limited quasi-3D RDE simulation of the polarity establishment network versus the corresponding particle-based system.** Comparison between the q3D-RDE and q3D-PB system in the reaction-limited regime (D_m_ = D_c_ = 5 µm^2^/s).


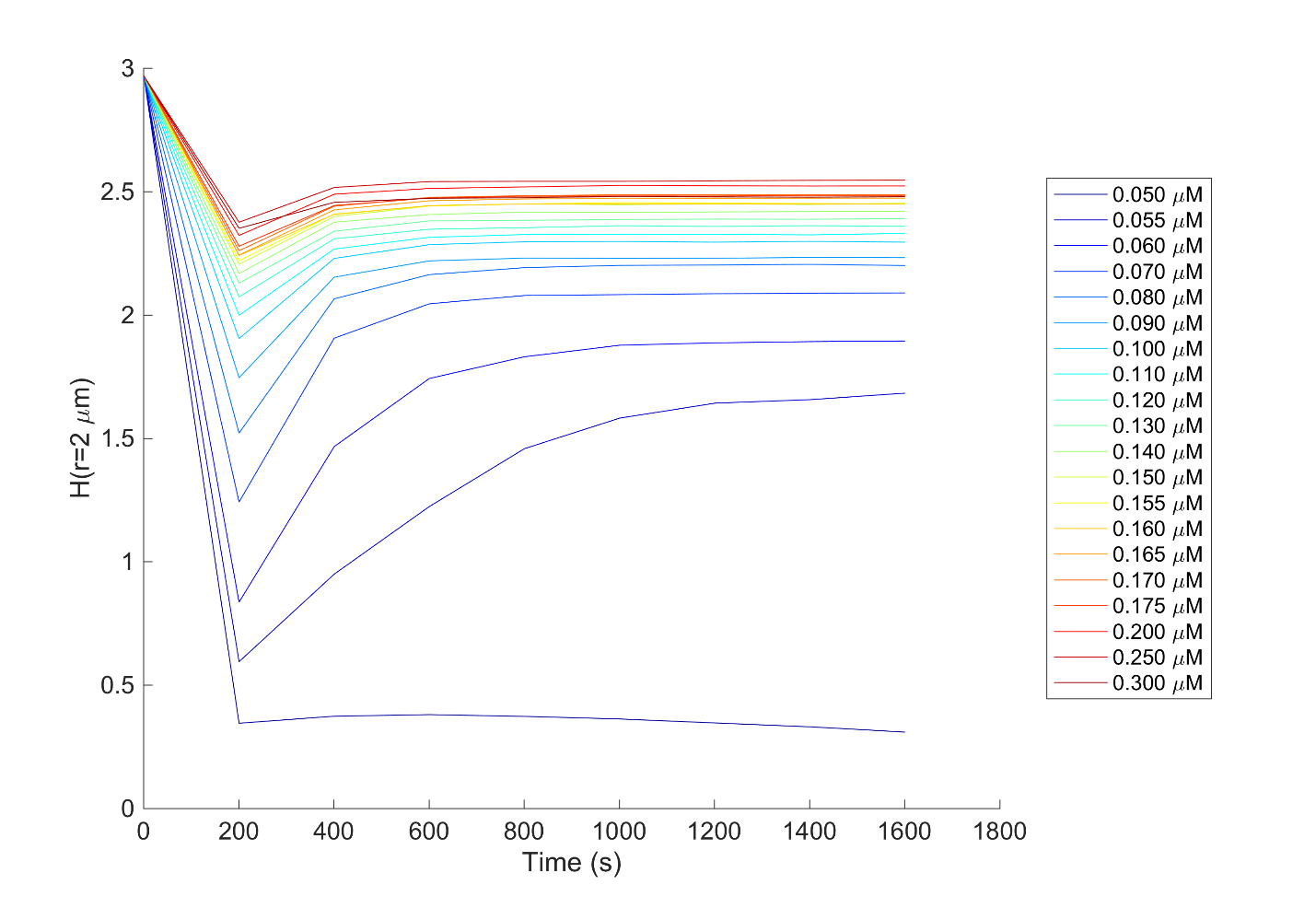


**Fig SP. Quantifying H(r) at t=1800s for pre-polarized q3D-RDEs is a reasonable marker for maintenance vs. loss of polarity.**

**
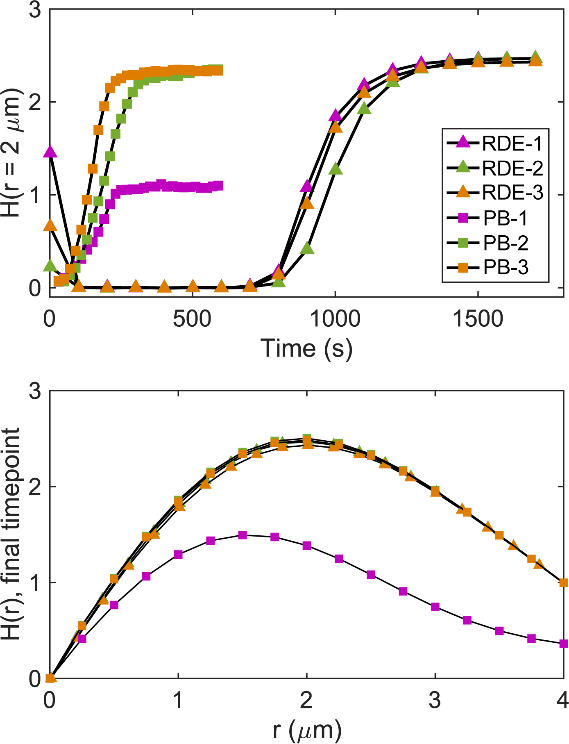

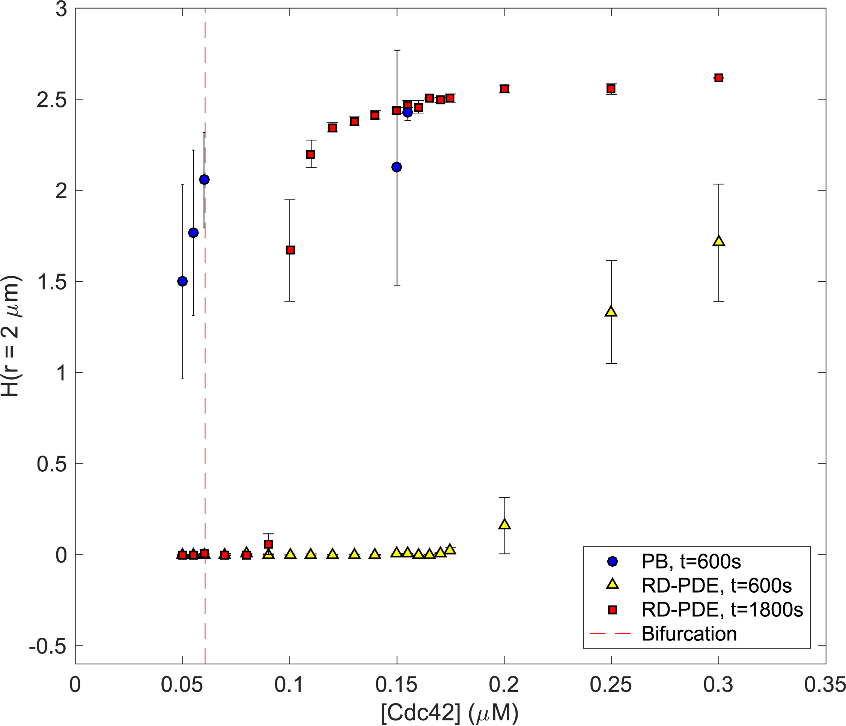
**

**Fig SQ**. **Considering the volume-adjusted, two-compartment RDE system makes no qualitative difference for our observations.** Instead of the q3D-RDE setup described in the main text, a volume-adjusted form of the RDEs was used, similar to [2]. Left: polarization dynamics and quantitative polarity site similarity, as in the Fig. 9 main text. Here, 1800s was sufficient for full polarization, so the RDE H(r) curves from Simulations 1-3 were overlaid with the PB simulations. Right: Bifurcation diagram. The bifurcation point was calculated using linear stability analysis.

**References**

1. Lipková J, Zygalakis KC, Chapman SJ, Erban R. Analysis of Brownian Dynamics Simulations of Reversible Bimolecular Reactions. SIAM J Appl Math. 2011;71(3):714.

2. Wu C-F, Chiou J-G, Minakova M, Woods B, Tsygankov D, Zyla TR, et al. Role of competition between polarity sites in establishing a unique front. Elife. 2015;4:e11611.
