# Supplementary material for: Particle-based simulations of polarity establishment reveal stochastic promotion of Turing pattern formation
Source: PLoS Comput Biol. 2018 Mar 12;14(3):e1006016. doi: 10.1371/journal.pcbi.1006016 (PMC5864077; doi:10.1371/journal.pcbi.1006016)
Supplement: S1 Dataset — This .zip file contains a General folder with code for procedures described in the main text, and folders with data and code to render data-based parts of Figs 2–10. (ZIP) [file pcbi.1006016.s004.zip › General/Overview.docx]

**General pieces of code**

RDE_2D.m

- Simulation code for the 2D reaction-diffusion PDEs.

RDE_volAdj3D.m

- Simulation code for the volume-adjusted reaction-diffusion PDEs.

RDE_q3D.m

- Simulation code for the quasi-3D reaction-diffusion PDEs.
- By setting option = 1 in line 48, can also perform the numerical bifurcation estimation.

sqEuclTorus_mex.c

- C file for use with MATLAB (MEX-C) to calculate nearest interparticle distances on the simulation domain.
- Must be compiled for specific platforms. Best to recompile for your own system. Feel free to try our pre-compiled versions:
  - sqEuclTorus_mex.mexa64 - Unix version for UNC’s Killdevil Compute Cluster
  - sqEuclTorus_mex.mexmaci64 – Mac version used on a local machine

pbsim_2d.m

- Simulation code for the 2D particle-based simulations.

pbsim_q3d.m

- Simulation code for the quasi-3D particle-based simulations.

vis_2d_clust_Cdc42T_postCentering.m

- Renders the Cdc42-GTP particle distributions as a movie given the parameter and data files.
- Uncomment line 41 to allow an automated coordinate shift, keeping the final polarized distribution from wrapping around the domain boundaries.
- Requires the QTWriter package for MATLAB. The version used by us is included.

CoordExtractor.m

- For reading the particle coordinates from the data file.

GeneratePDEInitEquiv_fromPB.m

- For creating 2D and volume-adjusted RDE initial condition files.
- Use for 2D PB data 🡪 RDE_2D init file
- Use for q3D PB data 🡪 RDE_volAdj3D init file

make_q3D_pb_inits.m

- For creating quasi-3D PDE initial condition files.
- Use for q3D PB data 🡪 RDE_q3D init file

PDE_Hfn_clustQuant_600s1800s.m

- For calculating H(r) at t=600s and 1800s for either q3D-RDE data, or the volume-adjusted RDE data. Can use to calculate H(r) at the t=200s and t=600s 2D RDE data, just need to modify the time points specified.
- Can also be modified to calculate H(r) at different/more time points.

PB_Hfn_clustQuant.m

- For calculating H(r) at t=600s, but can be modified for different/more time points.
- Works for both the 2D and q3D particle simulation data.

LSA (directory)

- Contains code for linear stability analysis of the 2D RDEs.
- Adjust the parameters_eta1_polar.m to set the parameters to search for LSA.

Empirical Fitting (directory)

- Contains code to perform simulations, and to fit ODEs to the resulting simulations.
