# Supplementary material for: Particle-based simulations of polarity establishment reveal stochastic promotion of Turing pattern formation
Source: PLoS Comput Biol. 2018 Mar 12;14(3):e1006016. doi: 10.1371/journal.pcbi.1006016 (PMC5864077; doi:10.1371/journal.pcbi.1006016)
Supplement: S1 Dataset — This .zip file contains a General folder with code for procedures described in the main text, and folders with data and code to render data-based parts of Figs 2–10. (ZIP) [file pcbi.1006016.s004.zip › General/QTWriter-master-2/References/qtff.pdf]

QuickTime File Format Specification

Developer

Contents

Introduction to QuickTime File Format Specification 17
Organization of This Document 17
Licensing Information 18
Special Fonts 18
Updates to This Specification 18
For More Information 19

Overview of QTFF 20
Media Description 20
Atoms 20
    Atom Layout 22
    Atom Structure 22
QT Atoms and Atom Containers 24
    QT Atom Containers 27
QuickTime Movie Files 30
    The File Type Compatibility Atom 33
    Free Space Atoms 35
    Movie Data Atoms 35
    Preview Atoms 35

Movie Atoms 37
Overview of Movie Atoms 38
    The Movie Atom 40
    The Movie Profile Atom 42
    Movie Header Atoms 43
    Color Table Atoms 46
    User Data Atoms 47
Track Atoms 55
    Track Profile Atom 57
    Track Header Atoms 57
    Track Exclude From Autoselection Atoms 61
    Track Aperture Mode Dimension Atoms 61
    Clipping Atoms 66
    Clipping Region Atoms 67

Copyright © 2004, 2012 Apple Inc. All Rights Reserved.

- Track Matte Atoms 68
- Compressed Matte Atoms 69
- Edit Atoms 69
- Edit List Atoms 70
- Track Load Settings Atoms 71
- Track Reference Atoms 73
- Track Input Map Atoms 76

Media Atoms 79
- Media Header Atoms 80
- Extended Language Tag Atom 82
- Handler Reference Atoms 84
- Media Information Atoms 85
- Video Media Information Atoms 86
- Video Media Information Header Atoms 87
- Sound Media Information Atoms 88
- Sound Media Information Header Atoms 89
- Base Media Information Atoms 89
- Base Media Information Header Atoms 90
- Base Media Info Atoms 91
- Data Information Atoms 92
- Data Reference Atoms 94

Sample Atoms 95
- Sample Table Atoms 96
- Sample Description Atoms 99
- Time-to-Sample Atoms 101
- Composition Offset Atom 103
- Composition Shift Least Greatest Atom 105
- Using Composition Offset and Composition Shift Least Greatest Atoms 106
- Sync Sample Atoms 107
- Partial Sync Sample Atom 108
- Sample-to-Chunk Atoms 109
- Sample Size Atoms 111
- Chunk Offset Atoms 113
- Sample Dependency Flags Atom 115
- Using Sample Atoms 116

Compressed Movie Resources 117
- Allowing QuickTime to Compress the Movie Resource 118
- Structure of a Compressed Movie Resource 118

Reference Movies 119

- Reference Movie Atom 120
- Reference Movie Descriptor Atom 121
- Data Reference Atom 122
- Data Rate Atom 123
- CPU Speed Atom 124
- Version Check Atom 124
- Component Detect Atom 125
- Constants 127
- Quality Atom 127

Metadata 128

Overview 128
- Data Type 128
- Meaning or Purpose 128
- Data Location 129
- Localization 129
- Storage Location in a QuickTime File 129

Metadata Structure 129
- Metadata Atom 129
- Metadata Handler Atom 130
- Metadata Header Atom 131

Extensibility 132
Localization List Sets 132
Country List Atom 133
Language List Atom 134
Metadata Item Keys Atom 135
Metadata Item List Atom 137
Metadata Item Atom 138
Value Atom 139
Type Indicator 139
Locale Indicator 139
Item Information Atom (ID and flags) 141
Name 141
Data Atom Structure 142
Data Ordering 142
Well-Known Types 143
Location Metadata 144
QuickTime Metadata Keys 144
Direction Definition 153

Copyright © 2004, 2012 Apple Inc. All Rights Reserved.

Media Data Atom Types 154
Video Media 155
- Video Sample Description 155
- Video Sample Data 169

Sound Media 176
- Sound Sample Descriptions 176
- Sound Sample Data 187

Timecode Media 189
- Timecode Sample Description 190
- Timecode Media Information Atom 191

Text Media 193
- Text Sample Description 193
- Text Media Information Atom 196
- Text Sample Data 196
- Hypertext and Wired Text 197

Closed Captioning Media 198
- Closed Captioning Sample Description 198
- Closed Captioning Sample Data 199

Subtitle Media 199
- Subtitle Sample Description 200
- Font Table Atom 201
- Subtitle Sample Data 202
- Subtitle Style Atom 203
- Text Box atom 204
- Subtitle Track Header Size and Placement 205
- Referencing a Related Forced Subtitle Track 206

Music Media 206
- Music Sample Description 207
- Music Sample Data 207

MPEG-1 Media 207
- MPEG-1 Sample Description 207
- MPEG-1 Sample Data 207

Sprite Media 208
- Sprite Sample Description 208
- Sprite Sample Data 208

Sprite Track Properties 210
Sprite Track Media Format 211
- Sprite Media Format Atoms 213
- Sprite Media Format Extensions 213

2012-08-14 | Copyright © 2004, 2012 Apple Inc. All Rights Reserved.

- Sprite Track Property Atoms 214
Sprite Media Atom and Data Types 215
Sprite Button Behaviors 220
QT Atom Container Description Key 221
Sprite Media Handler Track Properties QT Atom Container Format 222
Sprite Media Handler Sample QT Atom Container Formats 223
Wired Action Grammar 225
Flash Media 234
Tween Media 235
- Tween Sample Description 235
- Tween Sample Data 236
- Tween Type Categories 237
- Tween QT Atom Container 238
Modifier Tracks 247
- Limitations of Spatial Modifier Tracks 247
Track References 248
Chapter Lists 248
3D Media 249
- 3D Sample Description 249
- 3D Sample Data 250
Streaming Media 250
- Streaming Media Sample Description 250
Hint Media 251
- Adding Hint Tracks to a Movie 252
- Packetization Hint Media Header Atom 253
- Hint Track User Data Atom 253
- Movie Hint Info Atom 254
Finding an Original Media Track From a Hint Track 255
RTP Hint Tracks 255
Hint Sample Data Format 256
Packetization Hint Sample Data for Data Format 'rtp ' 259
- Data Modes 263
VR Media 267
- VR World Atom Container 268
Node Parent Atom 273
Node Location Atom Structure 273
Custom Cursor Atoms 274
Node Information Atom Container 275
- Node Header Atom Structure 276

2012-08-14 | Copyright © 2004, 2012 Apple Inc. All Rights Reserved.

- Hot Spot Parent Atom 277
- Hot Spot Information Atom 278
- Specific Information Atoms 280
- Link Hot Spot Atom 280

URL Hot Spot Atom 283
Support for Wired Actions 283
QuickTime VR File Format 284
- Single-Node Panoramic Movies 285
- Single-Node Object Movies 286
- Multinode Movies 287

QTVR Track 288
- QuickTime VR Sample Description Structure 288

Panorama Tracks 289
- Panorama Sample Atom Structure 289
- Panorama Image Track 293
- Cylindrical Panoramas 295

Cubic Panoramas 296
- Image Tracks in Cubic Nodes 297

Panorama Tracks in Cubic Nodes 297
Nonstandard Cubes 299
Hot Spot Image Tracks 300
- Low-Resolution Image Tracks 301
- Track Reference Entry Structure 301

Object Tracks 302
- Object Sample Atom Structure 302

Track References for Object Tracks 309
Movie Media 310
- Movie Sample Description 310
- Movie Media Sample Format 310

Basic Data Types 316

Language Code Values 316
- Macintosh Language Codes 317
- ISO Language Codes 319

Calendar Date and Time Values 320
Matrices 320
Graphics Modes 321
RGB Colors 322
Balance 323

Copyright © 2004, 2012 Apple Inc. All Rights Reserved.

Some Useful Examples and Scenarios 324
Creating, Copying, and Disposing of Atom Containers 325
- Creating New Atoms 325
- Copying Existing Atoms 328
- Retrieving Atoms From an Atom Container 329
- Modifying Atoms 332
- Removing Atoms From an Atom Container 333

Preparing Sound and Subtitle Alternate Groups for Use with Apple Devices 334
- General 334
- Alternate Subtitle Tracks 335
- Alternate Sound Tracks 335
- Relationships Across Alternate Groups 336

Creating an Effect Description 337
- Structure of an Effect Description 337
- Required Atoms of an Effects Description 338
- Parameter Atoms of an Effects Description 338
- Creating an Input Map 340

Creating Movies with Modifier Tracks 343
Authoring Movies with External Movie Targets 345
- Target Atoms for Embedded Movies 346

Adding Wired Actions To a Flash Track 347
- Extending the SWF Format 347

Creating Video Tracks at 30 Frames per Second 349
Creating Video Tracks at 29.97 Frames per Second 350
Creating Sound Tracks at 44.1 kHz 351
Creating a Timecode Track for 29.97 FPS Video 352
Playing with Edit Lists 356
Interleaving Movie Data 358
Referencing Two Data Files With a Single Track 359
Getting the Name of a QuickTime VR Node 361
Adding Custom Atoms in a QuickTime VR Movie 363
Adding Atom Containers in a QuickTime VR Movie 364
Optimizing QuickTime VR Movies for Web Playback 365
- The QTVR Flattener 366
- Sample Atom Container for the QTVR Flattener 368

QuickTime Image File Format 370
Atom Types in QuickTime Image Files 370
Recommended File Type and Suffix 372

Copyright © 2004, 2012 Apple Inc. All Rights Reserved.

Defining Media Data Layouts 373
Using QuickTime Files and Media Layouts 373

Random Access 375
Seeking With a QuickTime File 375

Metadata Handling 377
Digital Video File Formats 377
Digital Audio File Formats 378
Still Image File Formats 379
Animation and 3D File Formats 381

Summary of VR World and Node Atom Types 382
C Summary 382
    Constants 382
    Data Types 385

Profile Atom Guidelines 393
About This Appendix 393
Profile Atom Specification 394
    Definition 394
    Syntax 396
    Semantics 396
Universal Features 397
    Table of Features 398
    Maximum Video Bit Rate 399
    Average Video Bit Rate 400
    Maximum Audio Bit Rate 401
    Average Audio Bit Rate 403
    QuickTime Video Codec Type 404
    QuickTime Audio Codec Type 405
    MPEG-4 Video Profile 406
    MPEG-4 Video Codec 407
    MPEG-4 Video Object Type 408
    MPEG-4 Audio Codec 409
    Maximum Video Size in a Movie 411
    Maximum Video Size in a Track 412
    Maximum Video Frame Rate in a Single Track 414
    Average Video Frame Rate in a Single Track 415
    Video Variable Frame Rate Indication 416

2012-08-14 | Copyright © 2004, 2012 Apple Inc. All Rights Reserved.

Audio Sample Rate for a Sample Entry 417
Audio Variable Bit Rate Indication 418
Audio Channel Count 419

Audio Priming - Handling Encoder Delay in AAC 421
Background – AAC Encoding 421
The Timing and Synchronization Problem 422
Historical Solution—Implicit Encoder Delay 423
Using Track Structures to Represent Encoder Delay Explicitly 424
Edit List Atom 424
Sample Group Structures 425
Example—Representing Encoder Delay Explicitly 429
Audio Data 430
Track Structures 430
Summary—Using Track Structures to Represent Encoder Delay 432

Document Revision History 433

Glossary 436

2012-08-14 | Copyright © 2004, 2012 Apple Inc. All Rights Reserved.

Figures, Tables, and Listings

Overview of QTFF 20

Figure 1-1 A sample atom 22
Figure 1-2 Calculating atom sizes 24
Figure 1-3 QT atom layout 26
Figure 1-4 QT atom container with parent and child atoms 28
Figure 1-5 A QT atom container with two child atoms 29
Figure 1-6 The structure of a QuickTime movie file 32
Figure 1-7 The layout of a preview atom 35
Table 1-1 Basic atom types of a QuickTime file 32

Movie Atoms 37

Figure 2-1 Sample organization of a one-track video movie 39
Figure 2-2 The layout of a movie atom 41
Figure 2-3 The layout of a movie header atom 44
Figure 2-4 The layout of a color table atom 46
Figure 2-5 The layout of a user data atom 48
Figure 2-6 The layout of a track atom 56
Figure 2-7 The layout of a track header atom 58
Figure 2-8 Example of alternate tracks in two alternate groups 60
Figure 2-9 The layout of a track aperture mode dimensions atom 63
Figure 2-10 The layout of a track clean aperture dimensions atom 64
Figure 2-11 The layout of a track production aperture dimensions atom 65
Figure 2-12 The layout of a track encoded pixels dimensions atom 66
Figure 2-13 The layout of a clipping atom 67
Figure 2-14 The layout of a track matte atom 68
Figure 2-15 The layout of an edit atom 70
Figure 2-16 The layout of an edit list table entry 71
Figure 2-17 The layout of a track load settings atom 72
Figure 2-18 The layout of a track reference atom 74
Figure 2-19 The layout of a track input map atom 76
Figure 2-20 The layout of a media atom 79
Figure 2-21 The layout of a media header atom 81
Figure 2-22 The layout of an extended language tag atom 83
Figure 2-23 The layout of a handler reference atom 84

2012-08-14 | Copyright © 2004, 2012 Apple Inc. All Rights Reserved.

Figure 2-24 The layout of a media information atom for video 86
Figure 2-25 The layout of a media information header atom for video 87
Figure 2-26 The layout of a media information atom for sound 88
Figure 2-27 The layout of a sound media information header atom 89
Figure 2-28 The layout of a base media information atom 90
Figure 2-29 The layout of a base media information header atom 90
Figure 2-30 The layout of a base media info atom 91
Figure 2-31 The layout of a data information atom 93
Figure 2-32 Samples in a media 96
Figure 2-33 The layout of a sample table atom 98
Figure 2-34 The layout of a sample description atom 99
Figure 2-35 The layout of a time-to-sample atom 101
Figure 2-36 The layout of a time-to-sample table entry 102
Figure 2-37 An example of a time-to-sample table 103
Figure 2-38 The layout of a composition offset atom 104
Figure 2-39 The layout of a composition-offset table entry 105
Figure 2-40 The layout of a composition shift least greatest atom 105
Figure 2-41 The layout of a sync sample atom 107
Figure 2-42 The layout of a sync sample table 108
Figure 2-43 The layout of a partial sync sample atom 108
Figure 2-44 The layout of a partial sync sample table 109
Figure 2-45 The layout of a sample-to-chunk atom 110
Figure 2-46 The layout of a sample-to-chunk table entry 110
Figure 2-47 An example of a sample-to-chunk table 111
Figure 2-48 The layout of a sample size atom 112
Figure 2-49 An example of a sample size table 113
Figure 2-50 The layout of a chunk offset atom 114
Figure 2-51 An example of a chunk offset table 115
Figure 2-52 The layout of a sample dependency flags atom 115
Figure 2-53 An example of a sample dependency flags table 116
Figure 2-54 A movie atom containing a 'rmra' atom instead of a 'mvhd' atom 119
Figure 2-55 A 'rmra' atom with multiple 'rmda' atoms 120
Figure 2-56 Reference movie descriptor atom 121
Table 2-1 User data list entry types 48
Table 2-2 Track reference types 74
Table 2-3 Input types 78
Table 2-4 Data reference types 95
Table 2-5 Contents of complete compressed movie 118

 Copyright © 2004, 2012 Apple Inc. All Rights Reserved.

Metadata 128
Figure 3-1 Sample of a metadata atom and subatoms 130
Figure 3-2 A typical metadata item keys atom 137
Figure 3-3 An example of a metadata item keys atom 137
Figure 3-4 The metadata item list atom and the item/key Connection 138
Table 3-1 Example country list atom 133
Table 3-2 Example Language List atom 135
Table 3-3 Country and language indicators 139
Table 3-4 Example metadata tags 140
Table 3-5 Well-known data types 143
Table 3-6 Metadata keys 144
Table 3-7 Auxiliary keys for metadata 150

Media Data Atom Types 154
Figure 4-1 The layout of a color atom 162
Figure 4-2 Transfer between RGB and Y´CbCr color spaces 163
Figure 4-3 Normalized values, using the symbol E with a subscript for Y´, Cb, or Cr 163
Figure 4-4 Equations for stored Y´CbCr values of bit-depth of n in scheme A 164
Figure 4-5 Equations for stored Y´CbCr values of bit-depth n in scheme B 164
Figure 4-6 Equations for index code 1 166
Figure 4-7 Equations for index code 7 166
Figure 4-8 Matrix values for index code 1 167
Figure 4-9 Matrix values for index code 6 167
Figure 4-10 Matrix values for index code 7 167
Figure 4-11 Motion-JPEG A dual-field sample data 172
Figure 4-12 Motion-JPEG B dual-field sample data 174
Figure 4-13 A key frame sample atom container 212
Figure 4-14 Atoms that describe a sprite and its properties 212
Figure 4-15 Atoms that describe sprite images 213
Figure 4-16 Packet attribute flags 261
Figure 4-17 No-op data mode format 263
Figure 4-18 Immediate data mode table entry 264
Figure 4-19 Sample mode table entry format 264
Figure 4-20 Sample description mode format 266
Figure 4-21 Structure of the VR world atom container 269
Figure 4-22 Structure of the node information atom container 276
Figure 4-23 The structure of a single-node panoramic movie file 285
Figure 4-24 The structure of a single-node object movie file 286
Figure 4-25 The structure of a multinode movie file 287

2012-08-14 | Copyright © 2004, 2012 Apple Inc. All Rights Reserved.

Figure 4-26 Creating an image track for a panorama 294
Figure 4-27 Creating an image track for a panorama, with the image track oriented horizontally 295
Figure 4-28 Cubic node sample order versus cube face orientation 297
Figure 4-29 The structure of an image track for an object 309
Table 4-1 Some image compression formats 155
Table 4-2 Video sample description extensions 158
Table 4-3 Common pixel aspect ratios 160
Table 4-4 Table of primaries, index, and values 165
Table 4-5 Table of transfer function index and values 166
Table 4-6 Table of matrix index and values 167
Table 4-7 Partial list of supported QuickTime audio formats. 176
Table 4-8 Text face values 191
Table 4-9 Font face values 195
Table 4-10 Text sample extensions 196
Table 4-11 Font face values 201
Table 4-12 Subtitle sample extensions 203
Table 4-13 Sprite properties 209
Table 4-14 Sprite track properties 211
Table 4-15 Tween type values 236
Table 4-16 Tween types 243
Table 4-17 The 'hinf' atom type containing child atoms 254
Table 4-18 Hint track sample description 257
Table 4-19 The structure of table entries 258
Table 4-20 Packetization hint data elements 259
Table 4-21 Packet entry data elements 260
Table 4-22 RTP header information elements 261
Table 4-23 Extra information TLVs 262
Table 4-24 TLV type 262
Table 4-25 Data table entries 263
Table 4-26 Fields and their special values as represented in the pano sample data atom, providing backward compatibility to QuickTime VR 2.2 298
Table 4-27 Values for min and max fields 299
Table 4-28 Values used for representing sides 300
Listing 4-1 Streaming media sample description 250

Basic Data Types 316
Figure 5-1 How display matrices are used in QuickTime 321
Figure 5-2 Applying the transform 321
Table 5-1 QuickTime language code values 317

 Copyright © 2004, 2012 Apple Inc. All Rights Reserved.

Table 5-2 5-bit values of UTF-8 characters 320
Table 5-3 QuickTime graphics modes 322

Some Useful Examples and Scenarios 324
Figure 6-1 QT atom container after inserting an atom 326
Figure 6-2 QT atom container after inserting a second atom 327
Figure 6-3 Two QT atom containers, A and B 328
Figure 6-4 QT atom container after child atoms have been inserted 329
Figure 6-5 An example effect description for the Push effect 340
Figure 6-6 An example of an input map referencing two sources 342
Figure 6-7 Non-interleaved movie data 358
Figure 6-8 Interleaved movie data 358
Table 6-1 Example data values for time scale of 600 350
Table 6-2 Example data values for time scale of 2997 350
Table 6-3 Example data values for time scale of 44100 351
Table 6-4 Example track reference data values 355
Table 6-5 Example edit atom data values 356
Listing 6-1 Creating a new atom container 325
Listing 6-2 Disposing of an atom container 325
Listing 6-3 Creating a new QT atom container and calling `QTInsertChild` to add an atom. 326
Listing 6-4 Inserting a child atom 327
Listing 6-5 Inserting a container into another container 329
Listing 6-6 Finding a child atom by index 330
Listing 6-7 Finding a child atom by ID 331
Listing 6-8 Modifying an atom's data 332
Listing 6-9 Removing atoms from a container 333
Listing 6-10 Adding a `kParameterWhatName` atom to the atom container `effectDescription` 338
Listing 6-11 Adding an input reference atom to an input map 342
Listing 6-12 Linking a modifier track to the track it modifies 343
Listing 6-13 Updating the input map 344
Listing 6-14 Getting a node's name 361
Listing 6-15 Typical hot spot intercept procedure 363
Listing 6-16 Adding atom containers to a track 365
Listing 6-17 Using the flattener 366
Listing 6-18 Specifying a preview file for the flattener to use 368
Listing 6-19 Overriding the compression settings 368

QuickTime Image File Format 370
Figure A-1 An `'idsc'` atom followed by an `'idat'` atom 371

2012-08-14 | Copyright © 2004, 2012 Apple Inc. All Rights Reserved.

Table A-1 A QuickTime image file containing JPEG-compressed data 371

Profile Atom Guidelines 393

Figure F-1 The layout of a profile atom 396

Figure F-2 Layout of a typical feature 397

Table F-1 Universal features 398

Audio Priming - Handling Encoder Delay in AAC 421

Figure G-1 AAC encoded audio 422

Figure G-2 The layout of a sample group description atom 426

Figure G-3 The layout of a sample-to-group atom 428

Figure G-4 The layout of the table data format 429

2012-08-14 | Copyright © 2004, 2012 Apple Inc. All Rights Reserved.

Introduction to QuickTime File Format Specification

The QuickTime File Format (QTFF) is designed to accommodate the many kinds of data that need to be stored in order to work with digital multimedia. The QTFF is an ideal format for the exchange of digital media between devices, applications, and operating systems, because it can be used to describe almost any media structure.

The file format is object-oriented, consisting of a flexible collection of objects that is easily parsed and easily expanded. Unknown objects can simply be ignored or skipped, allowing considerable forward compatibility as new object types are introduced.

QuickTime itself provides a number of high-level functions that you can use to create and manipulate QuickTime files, without requiring you to understand the actual file format. These functions serve to insulate developers from the low-level details of operation. That said, not all kinds of QuickTime files can be created without the information presented here.

> **Important:** The QuickTime File Format has been used as the basis of the MPEG-4 standard and the JPEG-2000 standard, developed by the International Organization for Standardization (ISO). Although these file types have similar structures and contain many functionally identical elements, they are distinct file types.

> **Warning:** Do not use this specification to interpret a file that conforms to a different specification, however similar.

The *QuickTime File Format Specification* assumes that you are familiar with the basic concepts of digital video and audio, as well as with programming QuickTime and the QuickTime API. Note that this version of the document supersedes all previous versions of the *QuickTime File Format Specification*.

Organization of This Document

This document begins with an overview of QuickTime atoms, then presents the structure of the QuickTime file format in detail. This is followed by a series of code examples for manipulating a QuickTime file using the QuickTime API. Finally, a number of related topics are described in a series of appendixes. These include such topics as the handling of metadata when importing files into QuickTime, random access, and the QuickTime Image File format.

2012-08-14 | Copyright © 2004, 2012 Apple Inc. All Rights Reserved.

QuickTime files are described in general, rather than how they are supported on a specific computing platform or in a specific programming language. As a result, the file format information is presented in a tabular manner, rather than in coded data structures. Similarly, field names are presented in English rather than as programming language tags. Furthermore, to the extent possible, data types are described generically. For example, this book uses "32-bit signed integer" rather than "long" to define a 32-bit integer value.

QuickTime files are used to store QuickTime movies, as well as other data. If you are writing an application that parses QuickTime files, you should recognize that there may be non-movie data in the files.

QuickTime is a rich technology that continues to evolve as new practices and needs arise in audio/visual media. Because of this, certain elements of QuickTime technology may become deprecated over time. In order to preserve sufficient information about these legacy components for existing QuickTime files that include them, deprecated elements are marked with a note at the top of their section in this revision of the *QuickTime File Format Specification* .

Licensing Information

The *QuickTime File Format Specification* is provided for informational purposes. Apple may have patents, patent applications, trademarks, copyrights, or other intellectual property rights covering subject matter in this document. The furnishing of this document does not give you a license to any patents, trademarks, copyrights, or other intellectual property.

> **Important:** For more information about licensing the QuickTime File Format, contact: Apple, Inc., Software Licensing Department, 12545 Riata Vista Circle, MS 198 3-SWL, Austin, TX 78727. Email Address: sw.license@apple.com

Special Fonts

All code listings, reserved words, and the names of actual data structures, constants, fields, parameters, and routines are shown in `code voice`.

Words that appear in **boldface** are key terms or concepts and are defined in the "Glossary" (page 436).

Updates to This Specification

For updates or changes to this specification, go to the QuickTime documentation site at

2012-08-14 | Copyright © 2004, 2012 Apple Inc. All Rights Reserved.

QuickTime Reference Library

and click the File Format Specification link.

For More Information

For information about membership in Apple's developer program and developer technical support, you should go to this URL:

Apple Developer

For information on registering signatures, file types, and other technical information, contact

Apple Developer Technical Support (DTS)

Apple, Inc.

1 Infinite Loop, M/S 303-2T

Cupertino, CA 95014

2012-08-14 | Copyright © 2004, 2012 Apple Inc. All Rights Reserved.

Overview of QTFF

QuickTime movies are stored on disk, using two basic structures for storing information: **atoms** (also known as **simple atoms** or **classic atoms**) and **QT atoms**. To understand how QuickTime movies are stored, you need to understand the basic atom structures described in this chapter. Most atoms that you encounter in the QuickTime File Format are simple or classic atoms. Both simple atoms and QT atoms, however, allow you to construct arbitrarily complex hierarchical data structures. Both also allow your application to ignore data that they don't understand.

Media Description

A QuickTime file stores the description of its media separately from the media data.

The description is called the **movie resource**, **movie atom**, or simply **the movie**, and contains information such as the number of tracks, the video compression format, and timing information. The movie resource also contains an index describing where all the media data is stored.

The media data is the actual sample data, such as video frames and audio samples, used in the movie. The media data may be stored in the same file as the QuickTime movie, in a separate file, in multiple files, in alternate sources such as databases or real-time streams, or in some combination of these.

Atoms

The basic data unit in a QuickTime file is the atom. Each atom contains size and type fields that precede any other data. The size field indicates the total number of bytes in the atom, including the size and type fields. The type field specifies the type of data stored in the atom and, by implication, the format of that data. In some cases, the size and type fields are followed by a version field and a flags field. An atom with these version and flags fields is sometimes called a **full atom**.

> **Note:** An **atom**, as described in this document, is functionally identical to a **box**, as described in the ISO specifications for MPEG-4 and Motion JPEG-2000. An atom that includes version and flags fields is functionally identical to a **full box** as defined in those specifications.

Atom types are specified by a 32-bit unsigned integer, typically interpreted as a four-character ASCII code. Apple, Inc. reserves all four-character codes consisting entirely of lowercase letters. Unless otherwise stated, all data in a QuickTime movie is stored in big-endian byte ordering, also known as network byte ordering, in which the most significant bytes are stored and transmitted first.

Atoms are hierarchical in nature. That is, one atom can contain other atoms, which can contain still others, and so on. This hierarchy is sometimes described in terms of a parent, children, siblings, grandchildren, and so on. An atom that contains other atoms is called a **container atom**. The **parent atom** is the container atom exactly one level above a given atom in the hierarchy.

For example, a movie atom contains several different kinds of atoms, including one track atom for each track in the movie. The track atoms, in turn, contain one media atom each, along with other atoms that define other track characteristics. The movie atom is the parent atom of the track atoms. The track atoms are siblings. The track atoms are parent atoms of the media atoms. The movie atom is *not* the parent of the media atoms, because it is more than one layer above them in the hierarchy.

An atom that does not contain other atoms is called a **leaf atom**, and typically contains data as one or more fields or tables. Some leaf atoms act as flags or placeholders, however, and contain no data beyond their size and type fields.

The format of the data stored within a given atom cannot always be determined by the type field of the atom alone; the type of the parent atom may also be significant. In other words, a given atom type can contain different kinds of information depending on its parent atom. For example, the profile atom inside a movie atom contains information about the movie, while the profile atom inside a track atom contains information about the track. This means that all QuickTime file readers must take into consideration not only the atom type, but also the atom's containment hierarchy.

2012-08-14 | Copyright © 2004, 2012 Apple Inc. All Rights Reserved.

Atom Layout

Figure 1-1 (page 22) shows the layout of a sample atom. Each atom carries its own size and type information as well as its data. Throughout this document, the name of a container atom (an atom that contains other atoms, including other container atoms) is printed in a gray box, and the name of a leaf atom (an atom that contains no other atoms) is printed in a white box. Leaf atoms contain data, usually in the form of tables.

Figure 1-1 A sample atom

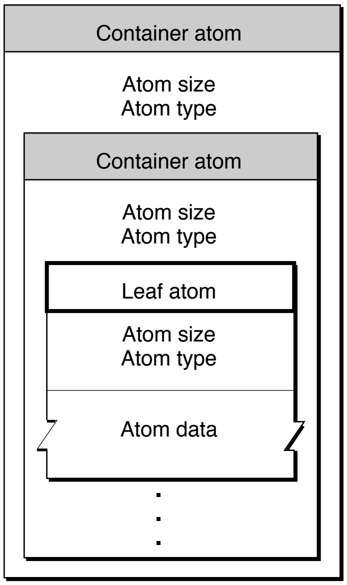

A leaf atom, as shown in Figure 1-1 (page 22), simply contains a series of data fields accessible by offsets.

Atoms within container atoms do not generally have to be in any particular order, unless such an order is specifically called out in this document. One such example is the handler description atom, which must come before the data being handled. For example, a media handler description atom must come before a media information atom, and a data handler description atom must come before a data information atom.

Atom Structure

Atoms consist of a header, followed by atom data. The header contains the atom's size and type fields, giving the size of the atom in bytes and its type. It may also contain an extended size field, giving the size of a large atom as a 64-bit integer. If an extended size field is present, the size field is set to 1. The actual size of an atom cannot be less than 8 bytes (the minimum size of the type and size fields).

Some atoms also contain version and flags fields. These are sometimes called full atoms. The flag and version fields are not treated as part of the atom header in this document; they are treated as data fields specific to each atom type that contains them. Such fields must always be set to zero, unless otherwise specified.

2012-08-14 | Copyright © 2004, 2012 Apple Inc. All Rights Reserved.

An atom header consists of the following fields:

Atom size

A 32-bit integer that indicates the size of the atom, including both the atom header and the atom's contents, including any contained atoms. Normally, the `size` field contains the actual size of the atom, in bytes, expressed as a 32-bit unsigned integer. However, the `size` field can contain special values that indicate an alternate method of determining the atom size. (These special values are normally used only for media data (`'mdat'`) atoms.)

Two special values are valid for the `size` field:

- 0, which is allowed only for a top-level atom, designates the last atom in the file and indicates that the atom extends to the end of the file.
- 1, which means that the actual size is given in the `extended size` field, an optional 64-bit field that follows the `type` field.

  This accommodates media data atoms that contain more than 2^32 bytes.

Figure 1-2 (page 24) shows how to calculate the size of an atom.

Type

A 32-bit integer that contains the type of the atom. This can often be usefully treated as a four-character field with a mnemonic value, such as `'moov' (0x6D6F6F76)` for a movie atom, or `'trak' (0x7472616B)` for a track atom, but non-ASCII values (such as `0x00000001`) are also used.

Knowing an atom's type allows you to interpret its data. An atom's data can be arranged as any arbitrary collection of fields, tables, or other atoms. The data structure is specific to the atom type. An atom of a given type has a defined data structure.

If your application encounters an atom of an unknown type, it should not attempt to interpret the atom's data. Use the atom's `size` field to skip this atom and all of its contents. This allows a degree of forward compatibility with extensions to the QuickTime file format.

> **Warning:** The internal structure of a given type of atom can change when a new version is introduced. Always check the version field, if one exists. Never attempt to interpret data that falls outside of the atom, as defined by the Size or Extended Size fields.

Extended Size

If the `size` field of an atom is set to 1, the `type` field is followed by a 64-bit `extended size` field, which contains the actual size of the atom as a 64-bit unsigned integer. This is used when the size of a media data atom exceeds 2^32 bytes.

2012-08-14 | Copyright © 2004, 2012 Apple Inc. All Rights Reserved.

When the `size` field contains the actual size of the atom, the `extended size` field is not present. This means that when a QuickTime atom is modified by adding data, and its size crosses the 2^32 byte limit, there is no `extended size` field in which to record the new atom size. Consequently, it is not always possible to enlarge an atom beyond 2^32 bytes without copying its contents to a new atom.

To prevent this inconvenience, media data atoms are typically created with a 64-bit placeholder atom immediately preceding them in the movie file. The placeholder atom has a type of `kWideAtomPlaceholderType` (`'wide'`).

Much like a `'free'` or `'skip'` atom, the `'wide'` atom is reserved space, but in this case the space is reserved for a specific purpose. If a `'wide'` atom immediately precedes a second atom, the second atom can be extended from a 32-bit size to a 64-bit size simply by starting the atom header 8 bytes earlier (overwriting the `'wide'` atom), setting the `size` field to 1, and adding an `extended size` field. This way the offsets for sample data do not need to be recalculated.

The `'wide'` atom is exactly 8 bytes in size, and consists solely of its `size` and `type` fields. It contains no other data.

Note: A common error is thinking that the `'wide'` atom contains the extended size. The `'wide'` atom is merely a placeholder that can be overwritten if necessary, by an atom header containing an `extended size` field.

Figure 1-2 Calculating atom sizes

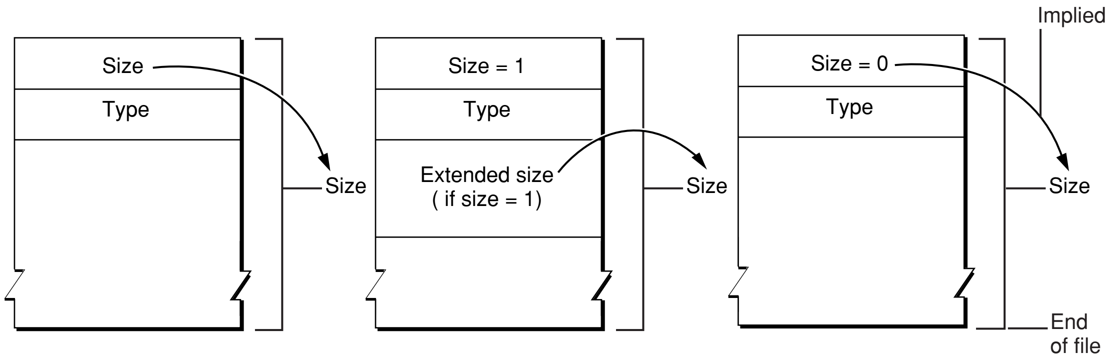

QT Atoms and Atom Containers

QT atoms are an enhanced data structure that provide a more general-purpose storage format and remove some of the ambiguities that arise when using simple atoms. A QT atom has an expanded header; the size and type fields are followed by fields for an atom ID and a count of child atoms.

2012-08-14 | Copyright © 2004, 2012 Apple Inc. All Rights Reserved.

This allows multiple child atoms of the same type to be specified through identification numbers. It also makes it possible to parse the contents of a QT atom of unknown type, by walking the tree of its child atoms.

QT atoms are normally wrapped in an **atom container**, a data structure with a header containing a lock count. Each atom container contains exactly one **root** atom, which is the QT atom. Atom containers are not atoms, and are not found in the hierarchy of atoms that makes up a QuickTime movie file. Atom containers may be found as data structures inside some atoms, however. Examples include media input maps and media property atoms.

> **Important:** An **atom container** is *not* the same as a **container atom**. An atom container is a *container*, not an atom.

Figure 1-3 (page 26) depicts the layout of a QT atom. Each QT atom starts with a QT atom container header, followed by the root atom. The root atom's type is the QT atom's type. The root atom contains any other atoms that are part of the structure.

Each container atom starts with a QT atom header followed by the atom's contents. The contents are either child atoms or data, but never both. If an atom contains children, it also contains all of its children's data and descendants. The root atom is always present and never has any siblings.

Figure 1-3 QT atom layout

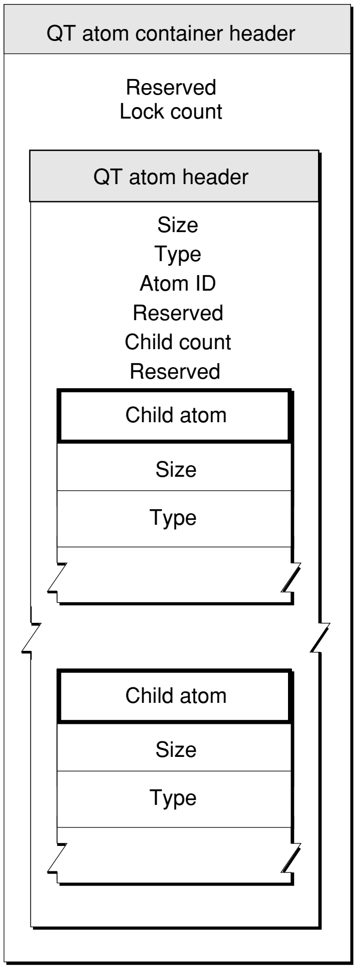

A QT atom container header contains the following data:

Reserved
: A 10-byte element that must be set to 0.

Lock count
: A 16-bit integer that must be set to 0.

Each QT atom header contains the following data:

Copyright © 2004, 2012 Apple Inc. All Rights Reserved.

Size

A 32-bit integer that indicates the size of the atom in bytes, including both the QT atom header and the atom's contents. If the atom is a leaf atom, then this field contains the size of the single atom. The size of container atoms includes all of the contained atoms. You can walk the atom tree using the size and child count fields.

Type

A 32-bit integer that contains the type of the atom. If this is the root atom, the type value is set to `'sean'`.

Atom ID

A 32-bit integer that contains the atom's ID value. This value must be unique among its siblings. The root atom always has an atom ID value of 1.

Reserved

A 16-bit integer that must be set to 0.

Child count

A 16-bit integer that specifies the number of child atoms that an atom contains. This count includes only immediate children. If this field is set to 0, the atom is a leaf atom and contains only data.

Reserved

A 32-bit integer that must be set to 0.

QT Atom Containers

A QuickTime atom container is a basic structure for storing information in QuickTime. An atom container is a tree-structured hierarchy of QT atoms. You can think of a newly created QT atom container as the root of a tree structure that contains no children.

An atom container is a container, not an atom. It has a reserved field and a lock count in its header, not a size field and type field. Atom containers are not found in the atom hierarchy of a QuickTime movie file, because they are not atoms. They may be found as data inside some atoms, however, such as in media input maps, media property atoms, video effects sample data, and tween sample data.

2012-08-14 | Copyright © 2004, 2012 Apple Inc. All Rights Reserved.

A QT atom container contains QT atoms, as shown in Figure 1-4 (page 28). Each QT atom contains either data or other atoms. If a QT atom contains other atoms, it is a parent atom and the atoms it contains are its child atoms. Each parent's child atom is uniquely identified by its atom type and atom ID. A QT atom that contains data is called a leaf atom.

Figure 1-4 QT atom container with parent and child atoms

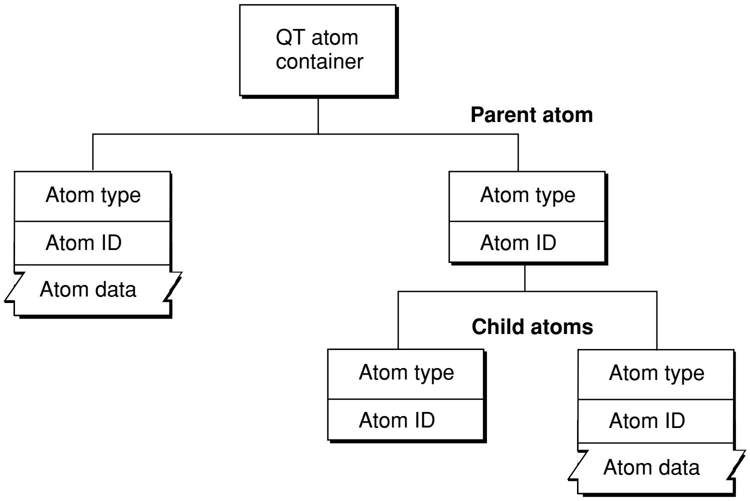

Each QT atom has an offset that describes the atom's position within the QT atom container. In addition, each QT atom has a type and an ID. The atom type describes the kind of information the atom represents. The atom ID is used to differentiate child atoms of the same type with the same parent; an atom's ID must be unique for a given parent and type. In addition to the atom ID, each atom has a 1-based index that describes its order relative to other child atoms of the same parent with the same atom type. You can uniquely identify a QT atom in one of three ways:

- By its offset within its QT atom container
- By its parent atom, type, and index
- By its parent atom, type, and ID

You can store and retrieve atoms in a QT atom container by index, ID, or both. For example, to use a QT atom container as a dynamic array or tree structure, you can store and retrieve atoms by index. To use a QT atom container as a database, you can store and retrieve atoms by ID. You can also create, store, and retrieve atoms using both ID and index to create an arbitrarily complex, extensible data structure.

2012-08-14 | Copyright © 2004, 2012 Apple Inc. All Rights Reserved.

> **Warning:** Since QT atoms are offsets into a data structure, they can be changed during editing operations on QT atom containers, such as inserting or deleting atoms. For a given atom, editing child atoms is safe, but editing sibling or parent atoms invalidates that atom's offset.

> **Note:** For cross-platform purposes, all data in a QT atom is expected to be in big-endian format. However, leaf data can be little-endian if it is custom to an application.

Figure 1-5 (page 29) shows a QT atom container that has two child atoms. The first child atom (offset = 10) is a leaf atom that has an atom type of `'abcd'`, an ID of 1000, and an index of 1. The second child atom (offset = 20) has an atom type of `'abcd'`, an ID of 900, and an index of 2. Because the two child atoms have the same type, they must have different IDs. The second child atom is also a parent atom of three atoms.

Figure 1-5 A QT atom container with two child atoms

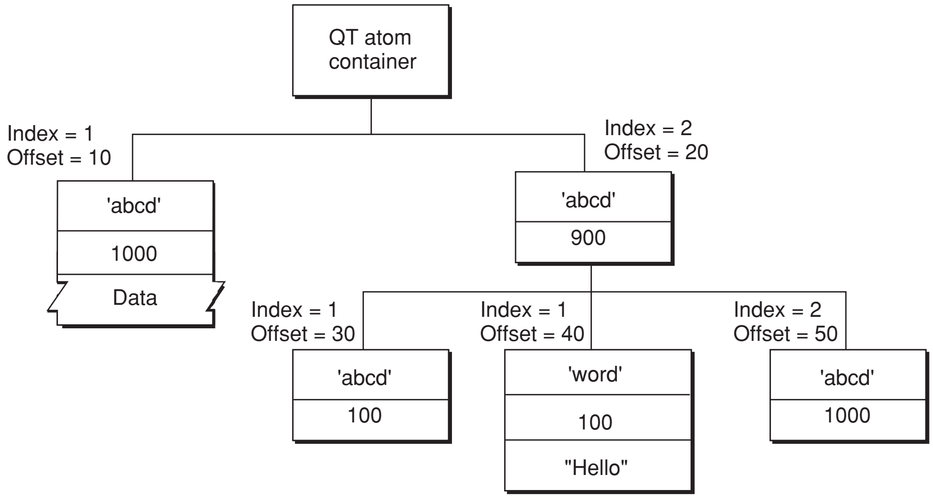

The first child atom (offset = 30) has an atom type of `'abcd'`, an ID of 100, and an index of 1. It does not have any children, nor does it have data. The second child atom (offset = 40) has an atom type of `'word'`, an ID of 100, and an index of 1. The atom has data, so it is a leaf atom. The second atom (offset = 40) has the same ID as the first atom (offset = 30), but a different atom type. The third child atom (offset = 50) has an atom type of `'abcd'`, an ID of 1000, and an index of 2. Its atom type and ID are the same as that of another atom (offset = 10) with a different parent.

Copyright © 2004, 2012 Apple Inc. All Rights Reserved.

> **Note:** If you are working with the QuickTime API, you do not need to parse QT atoms. Instead, the QT atom functions can be used to create atom containers, add atoms to and remove atoms from atom containers, search for atoms in atom containers, and retrieve data from atoms in atom containers.

Most QT atom functions take two parameters to specify a particular atom: the atom container that contains the atom, and the offset of the atom in the atom container data structure. You obtain an atom's offset by calling either `QTFindChildByID` or `QTFindChildByIndex`. An atom's offset may be invalidated if the QT atom container that contains it is modified.

When calling any QT atom function for which you specify a parent atom as a parameter, you can pass the constant `kParentAtomIsContainer` as an atom offset to indicate that the specified parent atom is the atom container itself. For example, you would call the `QTFindChildByIndex` function and pass `kParentAtomIsContainer` constant for the parent atom parameter to indicate that the requested child atom is a child of the atom container itself.

QuickTime Movie Files

The QuickTime file format describes the characteristics of QuickTime movie files. A QuickTime movie file contains a QuickTime movie resource, or else points to one or more external sources using movie references. The media samples used by the movie (such as video frames or groups of audio samples) may be included in the movie file, or may be external to the movie file in one or more files, streams, or other sources.

A QuickTime movie is not limited to video and audio; it may use any subset or combination of media types that QuickTime supports, including video, sound, still images, text, Flash, 3D models, and virtual reality panoramas. It supports both time-based and nonlinear interactive media.

In file systems that support filename extensions, QuickTime movie files should have an extension of `.mov`. On the Macintosh platform, QuickTime files have a Mac OS file type of `'MooV'`. QuickTime movie files should always be associated with the MIME type `"video/quicktime"`, whether or not the movie contains video.

2012-08-14 | Copyright © 2004, 2012 Apple Inc. All Rights Reserved.

> **Note:** The use of resource forks for the storage of QuickTime media is deprecated in the QuickTime file format. The information below is intended to document existing content and should not be used for new development.
>
> In file systems that support both a resource fork and a data fork, the movie resource may be contained in the resource fork. The default, however, is for the movie resource to be contained in the data fork for all file systems. If media sample data is included in the movie file, it is always in the data fork.

A QuickTime movie file is structured as a collection of atoms that together identify the file as a QuickTime movie, describe the structure of the movie, and may contain the sample data needed to play the movie. Not all atoms are required.

The file format is extensible, and from time to time new atom types are introduced. If your application encounters an unknown atom type in a QuickTime file, it should simply ignore it. This allows the file format to be extended without breaking existing applications, and provides a measure of forward compatibility. Because the first field in any atom contains its size, including any contained atoms, it is easy to skip to the end of an unknown atom type and continue parsing the file.

> **Important:** Generally speaking, atoms can be present in any order. Do not conclude that a particular atom is not present until you have parsed all the atoms in the file.

An exception is the file type atom, which typically identifies the file as a QuickTime movie. If present, this atom precedes any movie atom, movie data, preview, or free space atoms. If you encounter one of these other atom types prior to finding a file type atom, you may assume the file type atom is not present. (This atom is introduced in the *QuickTime File Format Specification* for 2004, and is not present in QuickTime movie files created prior to 2004).

While other atoms can be in any order, unless specified in this document, for practical reasons there is a recommended order that you should use when creating a QuickTime movie file. For example, the atom containing the movie resource should precede any atoms containing the movie's sample data. If you follow this recommended atom order, it is possible to play a movie over a network while the movie file is in the process of downloading.

A QuickTime movie file must contain a movie atom, which contains either the movie structure or a reference to one or more alternate movie sources external to the file. Generally speaking, these alternate sources will be QuickTime movie files that contain movie structures.

A QuickTime movie file typically contains one or more movie data atoms, which contain media sample data such as video frames and groups of audio samples. There may be no movie data atoms in the file, however, as the movie may depend on sample data external to the movie file, such as external data files or live streams

2012-08-14 | Copyright © 2004, 2012 Apple Inc. All Rights Reserved.

on the Internet. A single movie data atom may contain sample data for a variety of different media. Generally speaking, it is possible to contain all the media samples used by a movie in a single movie data atom. Movie data atoms can be quite large, and sometimes exceed 2^32 bytes.

Figure 1-6 (page 32) shows the essential atom types in a QuickTime movie file within which other atoms are stored. In addition, the file may contain free space atoms, preview atoms, and other atoms not enumerated in this file format specification. Unknown atom types should be ignored.

Figure 1-6 The structure of a QuickTime movie file

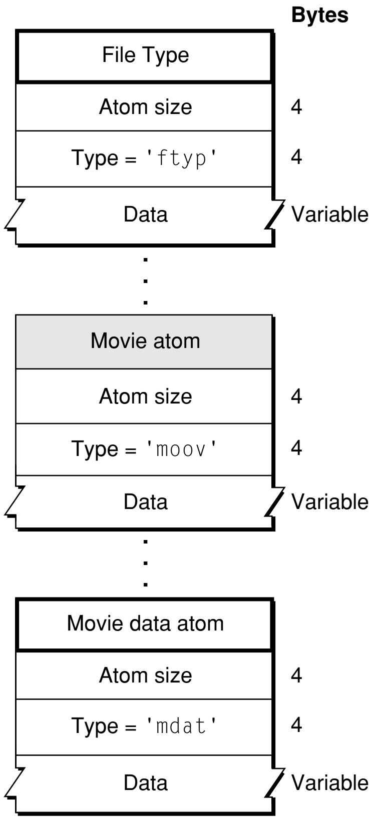

Table 1-1 (page 32) lists the basic atom types.

Table 1-1 Basic atom types of a QuickTime file

Atom type	Use
'ftyp'	File type compatibility—identifies the file type and differentiates it from similar file types, such as MPEG-4 files and JPEG-2000 files.
'moov'	Movie resource metadata about the movie (number and type of tracks, location of sample data, and so on). Describes where the movie data can be found and how to interpret it.

Atom type	Use
'mdat'	Movie sample data—media samples such as video frames and groups of audio samples. Usually this data can be interpreted only by using the movie resource.
'free'	Unused space available in file.
'skip'	Unused space available in file.
'wide'	Reserved space—can be overwritten by an extended size field if the following atom exceeds 2^32 bytes, without displacing the contents of the following atom.
'pnot'	Reference to movie preview data.

The following sections describe these basic atom types (except for the movie atom) in more detail, including descriptions of other atoms that each basic atom may contain. The movie atom is described separately in "Movie Atoms" (page 37)

The File Type Compatibility Atom

The file type compatibility atom, also called the file type atom, allows the reader to determine whether this is a type of file that the reader understands. Specifically, the file type atom identifies the file type specifications with which the file is compatible. This allows the reader to distinguish among closely related file types, such as QuickTime movie files, MPEG-4, and JPEG-2000 files (all of which may contain file type atoms, movie atoms, and movie data atoms).

When a file is compatible with more than one specification, the file type atom lists all the compatible types and indicates the preferred brand, or best use, among the compatible types. For example, a music player using a QuickTime-compatible file format might identify a file's best use as a music file for that player but also identify it as a QuickTime movie.

The file type atom serves a further purpose of distinguishing among different versions or specifications of the same file type, allowing it to convey more information than the file extension or MIME type alone. The file type atom also has the advantage of being internal to the file, where it is less subject to accidental alteration than a file extension or MIME type.

Note: The file type atom described here is functionally identical to the file type box defined in the ISO specifications for MPEG-4 and JPEG-2000.

The file type atom is optional, but strongly recommended. If present, it must be the first significant atom in the file, preceding the movie atom (and any free space atoms, preview atom, or movie data atoms).

2012-08-14 | Copyright © 2004, 2012 Apple Inc. All Rights Reserved.

The file type atom has an atom type value of `'ftyp'` and contains the following fields:

Size
: A 32-bit unsigned integer that specifies the number of bytes in this atom.

Type
: A 32-bit unsigned integer that identifies the atom type, typically represented as a four-character code; this field must be set to `'ftyp'`.

Major_Brand
: A 32-bit unsigned integer that should be set to `'qt  '` (note the two trailing ASCII space characters) for QuickTime movie files. If a file is compatible with multiple brands, all such brands are listed in the `Compatible_Brands` fields, and the `Major_Brand` identifies the preferred brand or best use.

Minor_Version
: A 32-bit field that indicates the file format specification version. For QuickTime movie files, this takes the form of four binary-coded decimal values, indicating the century, year, and month of the *QuickTime File Format Specification*, followed by a binary coded decimal zero. For example, for the June 2004 minor version, this field is set to the BCD values `20 04 06 00`.

Compatible_Brands[ ]
: A series of unsigned 32-bit integers listing compatible file formats. The major brand must appear in the list of compatible brands. One or more “placeholder” entries with value zero are permitted; such entries should be ignored.

If none of the Compatible_Brands fields is set to `'qt  '`, then the file is not a QuickTime movie file and is not compatible with this specification. Applications should return an error and close the file, or else invoke a file importer appropriate to one of the specified brands, preferably the major brand. QuickTime currently returns an error when attempting to open a file whose file type, file extension, or MIME type identifies it as a QuickTime movie, but whose file type atom does not include the `'qt  '` brand.

Note: A common source of this error is an MPEG-4 file incorrectly named with the `.mov` file extension or with the MIME type incorrectly set to “video/quicktime”. MPEG-4 files are automatically imported by QuickTime only when they are correctly identified as MPEG-4 files using the Mac OS file type, file extension, or MIME type.

If you are creating a file type that is fully compatible with the QuickTime file format, one of the Compatible_Brand fields must be set to `'qt  '`; otherwise QuickTime will not recognize the file as a QuickTime movie.

Warning: Use of the QuickTime file format in this manner is subject to license from Apple, Inc.

2012-08-14 | Copyright © 2004, 2012 Apple Inc. All Rights Reserved.

Free Space Atoms

Both `free` and `skip` atoms designate unused space in the movie data file. These atoms consist of only an atom header (size and type fields), followed by the appropriate number of bytes of free space. When reading a QuickTime movie, your application may safely skip these atoms. When writing or updating a movie, you may reuse the space associated with these atom types.

A `wide` atom typically precedes a movie data atom. The `wide` atom consists only of a type and size field. This occupies 8 bytes—enough space to add an extended size field to the header of the atom that follows, without displacing the contents of that atom. If an atom grows to exceed 2^32 bytes in size, and it is preceded by a `wide` atom, you may create a new atom header containing an extended size field by overwriting the existing atom header and the preceding `wide` atom.

Movie Data Atoms

As with the free and skip atoms, the movie data atom is structured quite simply. It consists of an atom header (atom size and type fields), followed by the movie's media data. Your application can understand the data in this atom only by using the metadata stored in the movie atom. This atom can be quite large, and may exceed 2^32 bytes, in which case the size field will be set to 1, and the header will contain a 64-bit extended size field.

Preview Atoms

The preview atom contains information that allows you to find the preview image associated with a QuickTime movie. The preview image, or poster, is a representative image suitable for display to the user in, for example, Open dialog boxes. Figure 1-7 (page 35) depicts the layout of the preview atom.

Figure 1-7 The layout of a preview atom

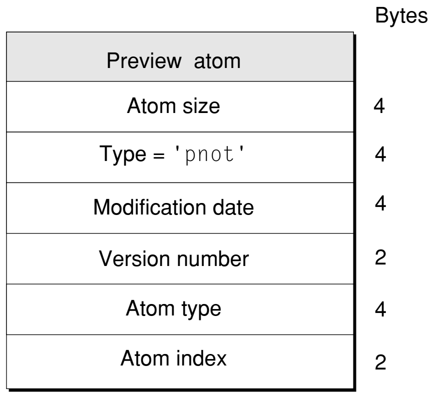

The preview atom has an atom type value of `'pnot'` and, following its atom header, contains the following fields:

Size
: A 32-bit integer that specifies the number of bytes in this preview atom.

Type
: A 32-bit integer that identifies the atom type; this field must be set to `'pnot'`.

Modification date
: A 32-bit unsigned integer containing a date that indicates when the preview was last updated. The data is in standard Macintosh format.

Version number
: A 16-bit integer that must be set to 0.

Atom type
: A 32-bit integer that indicates the type of atom that contains the preview data. Typically, this is set to `'PICT'` to indicate a QuickDraw picture.

Atom index
: A 16-bit integer that identifies which atom of the specified type is to be used as the preview. Typically, this field is set to 1 to indicate that you should use the first atom of the type specified in the atom type field.

Note: This specification defines the preview atom primarily for backward compatibility. Current practice is normally to define movie previews by placing information in the movie header atom. See "Movie Header Atoms" (page 43).

Copyright © 2004, 2012 Apple Inc. All Rights Reserved.

Movie Atoms

This chapter provides a general introduction to QuickTime movie atoms, as well as specific details on the layout and usage of these atoms. Each atom type discussed in this chapter is shown with an accompanying illustration that contains offset information, followed by field descriptions.

This chapter is divided into the following major sections:

- "Overview of Movie Atoms" (page 38) discusses QuickTime movie atoms, which act as containers for information that describes a movie's data. A conceptual illustration is provided that shows the organization of a simple, one-track QuickTime movie. Color table atoms and user data atoms are also discussed.
- "Track Atoms" (page 55) describes track atoms, which define a single track of a movie. Track user data atoms and hint tracks are also discussed.
- "Media Atoms" (page 79) discusses media atoms, which define a track's movie data, such as the media type and media time scale.
- "Sample Atoms" (page 95) discusses sample table atoms, which specify where media samples are located, their duration, and so on. The section also includes examples of how you use these atoms.

> **Note:** Media atoms and sample atoms do *not* contain actual sample data, such as video frames or audio samples. They contain metadata used to locate and interpret such samples.

- "Compressed Movie Resources" (page 117) discusses compressed movie resources, in which a lossless compression algorithm is used to compress the contents of the movie atom, including any track, media, or sample atoms. The contents must be decompressed before the movie atom can be parsed.
- "Reference Movies" (page 119) discusses movies that contain a reference movie atom (a list of references to alternate movies, as well as the criteria for selecting the correct movie from a list of alternates). Movie atoms that contain a reference movie atom do not necessarily contain track, media, or sample atoms.

Copyright © 2004, 2012 Apple Inc. All Rights Reserved.

Overview of Movie Atoms

QuickTime movie atoms have an atom type of `'moov'`. These atoms act as a container for the information that describes a movie's data. This information, or metadata, is stored in a number of different types of atoms. Generally speaking, only metadata is stored in a movie atom. Sample data for the movie, such as audio or video samples, are referenced in the movie atom, but are not contained in it.

The movie atom is essentially a container of other atoms. These atoms, taken together, describe the contents of a movie. At the highest level, movie atoms typically contain track atoms, which in turn contain media atoms. At the lowest level are the leaf atoms, which contain non-atom data, usually in the form of a table or a set of data elements. For example, a track atom contains an edit atom, which in turn contains an *edit list atom* , a leaf atom which contains data in the form of an edit list table. All of these atoms are discussed later in this document.

Figure 2-1 (page 39) provides a conceptual view of the organization of a simple, one-track QuickTime movie. Each nested box in the illustration represents an atom that belongs to its parent atom. The figure does not show the data regions of any of the atoms. These areas are described in the sections that follow.

Note that this figure shows the organization of a standard movie atom. It is possible to compress the movie metadata using a lossless compression algorithm. In such cases, the movie atom contains only a single child atom—the compressed movie atom (`'cmov'`). When this child atom is uncompressed, its contents conform to the structure shown in the following illustration. For details, see "Compressed Movie Resources" (page 117)

2012-08-14 | Copyright © 2004, 2012 Apple Inc. All Rights Reserved.

It is also possible to create a reference movie, a movie that refers to other movies; in this case the movie atom may contain only a reference movie atom (`'rmra'`). For details, see "Reference Movies". Ultimately, the chain must end in either a standard movie atom, such as the one in Figure 2-1 (page 39), or a compressed movie atom, which can be uncompressed to obtain the same structure.

Figure 2-1 Sample organization of a one-track video movie

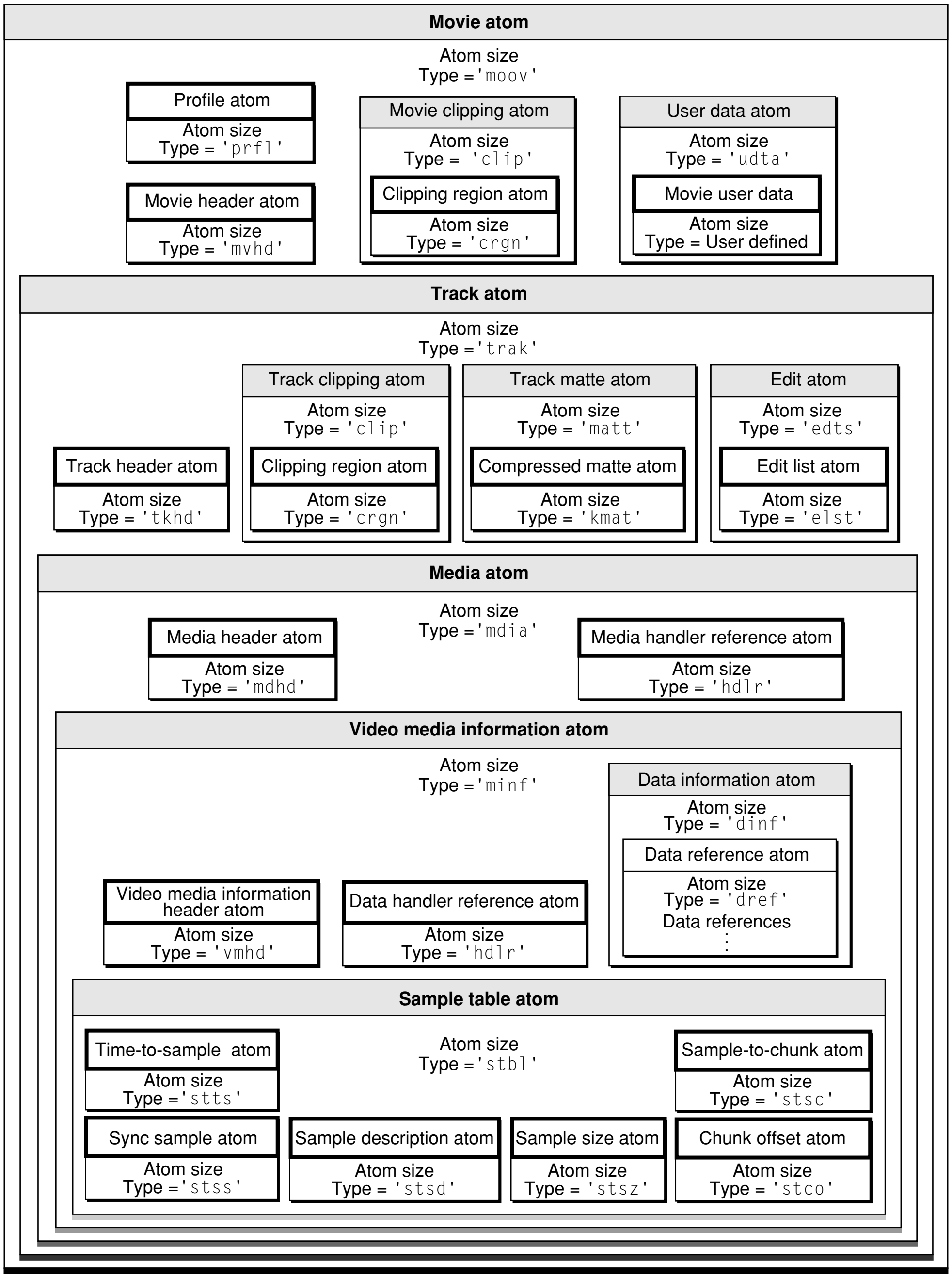

Copyright © 2004, 2012 Apple Inc. All Rights Reserved.

Note: Additional atoms may be present in a one-track QuickTime movie file that do not appear in Figure 2-1 (page 39).

The Movie Atom

You use movie atoms to specify the information that defines a movie—that is, the information that allows your application to interpret the sample data that is stored elsewhere. The movie atom usually contains a movie header atom, which defines the time scale and duration information for the entire movie, as well as its display characteristics. Existing movies may contain a movie profile atom, which summarizes the main features of the movie, such as the necessary codecs and maximum bit rate. In addition, the movie atom contains a track atom for each track in the movie.

The movie atom has an atom type of `'moov'`. It contains other types of atoms, including at least one of three possible atoms—the movie header atom (`'mvhd'`), the compressed movie atom (`'cmov'`), or a reference movie atom (`'rmra'`). An uncompressed movie atom can contain both a movie header atom and a reference movie atom, but it must contain at least one of the two. It can also contain several other atoms, such as a clipping atom (`'clip'`), one or more track atoms (`'trak'`), a color table atom (`'ctab'`), and a user data atom (`'udta'`).

Compressed movie atoms and reference movie atoms are discussed separately. This section describes normal uncompressed movie atoms.

Figure 2-2 (page 41) shows the layout of a typical movie atom.

Copyright © 2004, 2012 Apple Inc. All Rights Reserved.

Note: As previously mentioned, leaf atoms are shown as white boxes, while container atoms are shown as gray boxes.

Figure 2-2 The layout of a movie atom

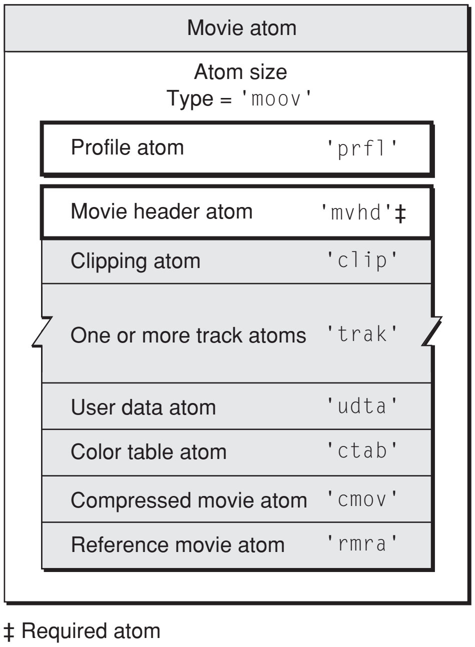

A movie atom may contain the following fields:

Size

The number of bytes in this movie atom.

Type

The type of this movie atom; this field must be set to `'moov'`.

Profile atom

See "The Movie Profile Atom" (page 42) for more information.

Movie header atom

See "Movie Header Atoms" (page 43) for more information.

Movie clipping atom

See "Clipping Atoms" (page 66) for more information.

Track atoms

See "Track Atoms" (page 55) for details on track atoms and their associated atoms.

User data atom

See "User Data Atoms" (page 47) for more information about user data atoms.

2012-08-14 | Copyright © 2004, 2012 Apple Inc. All Rights Reserved.

Color table atom

See “Color Table Atoms” (page 46) for a discussion of the color table atom.

Compressed movie atom

See “Compressed Movie Resources” (page 117) for a discussion of compressed movie atoms.

Reference movie atom

See “Reference Movies” (page 119) for a discussion of reference movie atoms.

The Movie Profile Atom

Note: Profile atoms are deprecated in the QuickTime file format. The information that follows is intended to document existing content containing profile atoms and should not be used for new development.

The movie profile atom summarizes the features and complexity of a movie, such as the required codecs and maximum bit rate, in order to help player applications or devices quickly determine whether they have the necessary resources to play the movie.

Features for a movie typically include the movie’s maximum video and audio bit rate, a list of audio and video codec types, the movie’s video dimensions, and any applicable MPEG-4 profiles and levels. This is all information that can also be obtained by examining the contents of the movie file in more detail. This summary is intended to allow applications or devices to quickly determine whether they can play the movie. It is not intended as a container for information that is not found elsewhere in the movie, and should not be used as one.

Note: The fact that a feature does not appear in the profile atom does not mean it is not present in the movie. The profile atom itself may not be present, or may list only a subset of movie features. The features listed in the profile atom are all present, but the list is not necessarily complete.

When creating a profile atom, it is permissible to omit some features that are present in the movie, but it is required to fully specify any features that are included in the profile. For example, a movie containing video may or may not have a video codec type feature in the profile atom, but if any video codec type feature is included in the profile atom, every required video codec must be listed in the profile atom.

The movie profile atom is a profile atom (`'prfl'`) whose parent is a movie atom. This is distinct from the track profile atom, whose parent is a track atom. The structure of the profile atom is identical in both cases, but the contents of a movie profile atom describe the movie as a whole, while the contents of a track profile atom are specific to a particular track.

2012-08-14 | Copyright © 2004, 2012 Apple Inc. All Rights Reserved.

The profile atom contains a list of features. In a movie profile atom, these features summarize the movie as a whole. In a track profile atom, these features describe a particular track.

Each entry in the feature list consists of four 32-bit fields:

- The first field is reserved and must be set to zero.
- The second field is the part-ID, which defines the feature as being either brand-specific or universal. Brand-specific features are particular to a specific brand. Universal features are can be found in any file type that uses the profile atom. Universal features have a part-ID of four ASCII spaces (0x20202020). Brand-specific features have a part-ID that is one of the Compatible_Brand codes for that file type, as specified in the file type atom (`'ftyp'`). For example, the part-ID for QuickTime-specific features is `'qt  '`. All features described in this document, however, are universal.
- The third field is the feature code, or name, a 32-bit unsigned integer that is usually best interpreted as four ASCII characters. Example: the maximum video bit rate feature has a feature code or name of `'mvbr'`. It is permissible to use a feature code value of zero (0x00000000, *not* four ASCII zero characters) as a placeholder in one or more name-value pairs. The reader should ignore feature codes of value zero.
- The fourth field is the value, which is also a 32-bit field. The value may be a signed or unsigned integer, or a fixed-point value, or contain subfields, or consist of a packed array; it can be interpreted only in relation to the specific feature.

For details on the structure and contents of profile atoms, see "Profile Atom Guidelines" (page 393).

Movie Header Atoms

You use the movie header atom to specify the characteristics of an entire QuickTime movie. The data contained in this atom defines characteristics of the entire QuickTime movie, such as time scale and duration. It has an atom type value of `'mvhd'`.

Copyright © 2004, 2012 Apple Inc. All Rights Reserved.

Figure 2-3 (page 44) shows the layout of the movie header atom. The movie header atom is a leaf atom.

Figure 2-3 The layout of a movie header atom

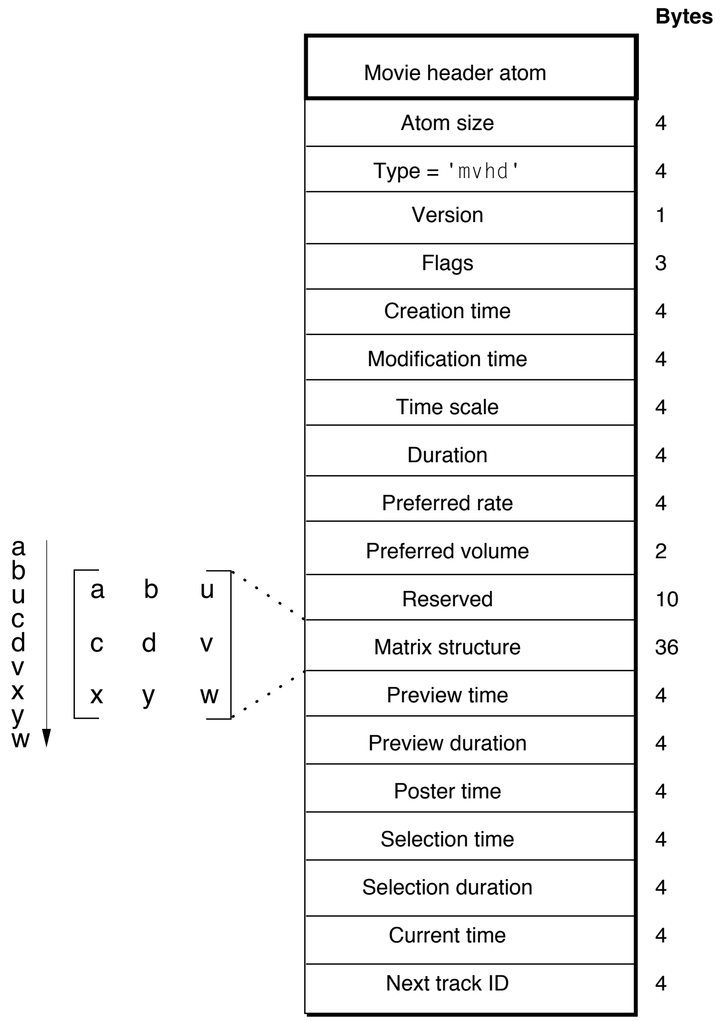

You define a movie header atom by specifying the following data elements.

Size
: A 32-bit integer that specifies the number of bytes in this movie header atom.

Type
: A 32-bit integer that identifies the atom type; must be set to `'mvhd'`.

Version
: A 1-byte specification of the version of this movie header atom.

2012-08-14 | Copyright © 2004, 2012 Apple Inc. All Rights Reserved.

Flags

Three bytes of space for future movie header flags.

Creation time

A 32-bit integer that specifies the calendar date and time (in seconds since midnight, January 1, 1904) when the movie atom was created. It is strongly recommended that this value should be specified using coordinated universal time (UTC).

Modification time

A 32-bit integer that specifies the calendar date and time (in seconds since midnight, January 1, 1904) when the movie atom was changed. BooleanIt is strongly recommended that this value should be specified using coordinated universal time (UTC).

Time scale

A time value that indicates the time scale for this movie—that is, the number of time units that pass per second in its time coordinate system. A time coordinate system that measures time in sixtieths of a second, for example, has a time scale of 60.

Duration

A time value that indicates the duration of the movie in time scale units. Note that this property is derived from the movie's tracks. The value of this field corresponds to the duration of the longest track in the movie.

Preferred rate

A 32-bit fixed-point number that specifies the rate at which to play this movie. A value of 1.0 indicates normal rate.

Preferred volume

A 16-bit fixed-point number that specifies how loud to play this movie's sound. A value of 1.0 indicates full volume.

Reserved

Ten bytes reserved for use by Apple. Set to 0.

Matrix structure

The matrix structure associated with this movie. A matrix shows how to map points from one coordinate space into another. See "Matrices" (page 320) for a discussion of how display matrices are used in QuickTime.

Preview time

The time value in the movie at which the preview begins.

Preview duration

The duration of the movie preview in movie time scale units.

Poster time

The time value of the time of the movie poster.

Copyright © 2004, 2012 Apple Inc. All Rights Reserved.

Selection time
: The time value for the start time of the current selection.

Selection duration
: The duration of the current selection in movie time scale units.

Current time
: The time value for current time position within the movie.

Next track ID
: A 32-bit integer that indicates a value to use for the track ID number of the next track added to this movie. Note that 0 is not a valid track ID value.

Note: The creation and modification date should be set using coordinated universal time (UTC). In prior versions of the QuickTime file format, this was not specified, and these fields were commonly set to local time for the time zone where the movie was created.

Color Table Atoms

Color table atoms define a list of preferred colors for displaying the movie on devices that support only 256 colors. The list may contain up to 256 colors. These optional atoms have a type value of `'ctab'`. The color table atom contains a Macintosh color table data structure.

Figure 2-4 (page 46) shows the layout of the color table atom.

Figure 2-4 The layout of a color table atom

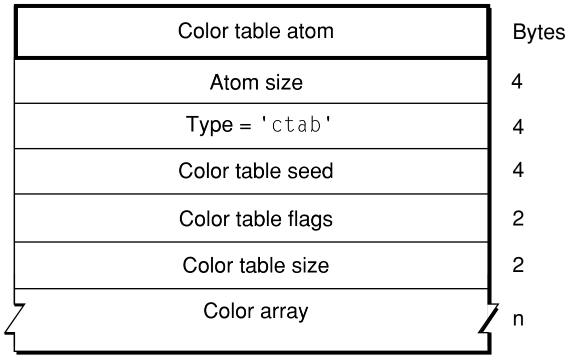

The color table atom contains the following data elements.

Size
: A 32-bit integer that specifies the number of bytes in this color table atom.

Copyright © 2004, 2012 Apple Inc. All Rights Reserved.

Type
: A 32-bit integer that identifies the atom type; this field must be set to `'ctab'`.

Color table seed
: A 32-bit integer that must be set to 0.

Color table flags
: A 16-bit integer that must be set to 0x8000.

Color table size
: A 16-bit integer that indicates the number of colors in the following color array. This is a zero-relative value; setting this field to 0 means that there is one color in the array.

Color array
: An array of colors. Each color is made of four unsigned 16-bit integers. The first integer must be set to 0, the second is the red value, the third is the green value, and the fourth is the blue value.

User Data Atoms

User data atoms allow you to define and store data associated with a QuickTime object, such as a movie `'moov'`, track `'trak'`, or media `'mdia'`. This includes both information that QuickTime looks for, such as copyright information or whether a movie should loop, and arbitrary information—provided by and for your application—that QuickTime simply ignores.

A user data atom whose immediate parent is a movie atom contains data relevant to the movie as a whole. A user data atom whose parent is a track atom contains information relevant to that specific track. A QuickTime movie file may contain many user data atoms, but only one user data atom is allowed as the immediate child of any given movie atom or track atom.

The user data atom has an atom type of `'udta'`. Inside the user data atom is a list of atoms describing each piece of user data. User data provides a simple way to extend the information stored in a QuickTime movie. For example, user data atoms can store a movie's window position, playback characteristics, or creation information.

This section describes the data atoms that QuickTime recognizes. You may create new data atom types that your own application recognizes. Applications should ignore any data atom types that they do not recognize.

2012-08-14 | Copyright © 2004, 2012 Apple Inc. All Rights Reserved.

Figure 2-5 (page 48) shows the layout of a user data atom.

Figure 2-5 The layout of a user data atom

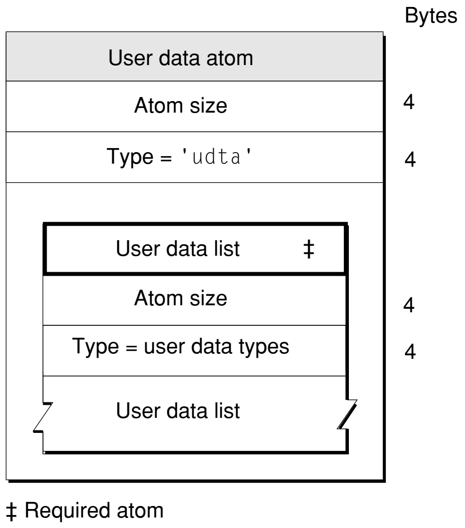

The user data atom contains the following data elements.

Size

A 32-bit integer that specifies the number of bytes in this user data atom.

Type

A 32-bit integer that identifies the atom type; this field must be set to `'udta'`.

User data list

A user data list that is formatted as a series of atoms. Each data element in the user data list contains size and type information along with its payload data. For historical reasons, the data list is optionally terminated by a 32-bit integer set to 0. If you are writing a program to read user data atoms, you should allow for the terminating 0. However, if you are writing a program to create user data atoms, you can safely leave out the terminating 0.

Table 2-1 (page 48) lists the currently defined list entry types.

Table 2-1 User data list entry types

List entry type	Description	For Sorting
`'©arg'`	Name of arranger	
`'©ark'`	Keywords for arranger	X

Copyright © 2004, 2012 Apple Inc. All Rights Reserved.

List entry type	Description	For Sorting
`'©cok'`	Keywords for composer	X
`'©com'`	Name of composer	
`'©cpy'`	Copyright statement	
`'©day'`	Date the movie content was created	
`'©dir'`	Name of movie’s director	
`'©ed1'` to `'©ed9'`	Edit dates and descriptions	
`'©fmt'`	Indication of movie format (computer-generated, digitized, and so on)	
`'©inf'`	Information about the movie	
`'©isr'`	ISRC code	
`'©lab'`	Name of record label	
`'©lal'`	URL of record label	
`'©mak'`	Name of file creator or maker	
`'©mal'`	URL of file creator or maker	
`'©nak'`	Title keywords of the content	X
`'©nam'`	Title of the content	
`'©pdk'`	Keywords for producer	X
`'©phg'`	Recording copyright statement, normally preceded by the symbol ℗	
`'©prd'`	Name of producer	
`'©prf'`	Names of performers	
`'©prk'`	Keywords of main artist and performer	X
`'©prl'`	URL of main artist and performer	
`'©req'`	Special hardware and software requirements	

Copyright © 2004, 2012 Apple Inc. All Rights Reserved.

List entry type	Description	For Sorting
'©snk'	Subtitle keywords of the content	X
'©snm'	Subtitle of content	
'©src'	Credits for those who provided movie source content	
'©swf'	Name of songwriter	
'©swk'	Keywords for songwriter	X
'©swr'	Name and version number of the software (or hardware) that generated this movie	
'©wrt'	Name of movie's writer	
'AllF'	Play all frames—byte indicating that all frames of video should be played, regardless of timing	
'hinf'	Hint track information—statistical data for real-time streaming of a particular track. For more information, see "Hint Track User Data Atom" (page 253).	
'hnti'	Hint info atom—data used for real-time streaming of a movie or a track. For more information, see "Movie Hint Info Atom" (page 254) and "Hint Track User Data Atom" (page 253).	
'name'	Name of object	
'tnam'	Localized track name optionally present in Track user data. The payload is described in "Track Name" (page 53).	
'tagc'	Media characteristic optionally present in Track user data—specialized text that describes something of interest about the track. For more information, see "Media Characteristic Tags" (page 52).	
'LOOP'	Long integer indicating looping style. This atom is not present unless the movie is set to loop. Values are 0 for normal looping, 1 for palindromic looping.	
'ptv '	Print to video—display movie in full screen mode. This atom contains a 16-byte structure, described in "Print to Video (Full Screen Mode)" (page 54).	

Copyright © 2004, 2012 Apple Inc. All Rights Reserved.

List entry type	Description	For Sorting
`'SelO'`	Play selection only—byte indicating that only the selected area of the movie should be played	
`'WLOC'`	Default window location for movie—two 16-bit values, {x,y}	

The user-data items labelled "keywords" and marked as "For Sorting" are for use when the display text does not have a pre-determined sorting order (for example, in oriental languages when the sorting depends on the contextual meaning). These keywords can be sorted algorithmically to place the corresponding items in correct order.

The window location, looping, play selection only, play all frames, and print to video atoms control the way QuickTime displays a movie. These atoms are interpreted *only* if the user data atom's immediate parent is a movie atom (`'moov'`). If they are included as part of a track atom's user data, they are ignored.

User Data Text Strings and Language Codes

All user data list entries whose type begins with the © character (ASCII 169) are defined to be international text. These list entries must contain a list of text strings with associated language codes. By storing multiple versions of the same text, a single user data text item can contain translations for different languages.

The list of text strings uses a small integer atom format, which is identical to the QuickTime atom format except that it uses 16-bit values for size and type instead of 32-bit values. The first value is the size of the string, including the size and type, and the second value is the language code for the string.

User data text strings may use either Macintosh text encoding or Unicode text encoding. The format of the language code determines the text encoding format. Macintosh language codes are followed by Macintosh-encoded text. If the language code is specified using the ISO language codes listed in specification ISO 639-2/T, the text uses Unicode text encoding. When Unicode is used, the text is in UTF-8 unless it starts with a byte-order-mark (BOM, 0xFEFF), in which case the text is in UTF-16. Both the BOM and the UTF-16 text should be big-endian. Multiple versions of the same text may use different encoding schemes.

> **Important:** Language code values less than 0x400 are Macintosh language codes. Language code values greater than or equal to 0x400 are ISO language codes. The exception to this rule is language code 0x7FFF, which indicates an unspecified Macintosh language.

ISO language codes are three-character codes. In order to fit inside a 16-bit field, the characters must be packed into three 5-bit subfields. This packing is described in "ISO Language Codes" (page 319).

Copyright © 2004, 2012 Apple Inc. All Rights Reserved.

Media Characteristic Tags

A track (`'trak'`) atom's user data atom may contain zero or more media characteristic tag atoms (`'tagc'`).

The media characteristic tag atom's payload data is a tag that indicates something of interest about the track. This is a specialized string consisting of a subset of US-ASCII (7 bits plus a clear high bit) characters and conforming to the structure described in the following paragraphs. This is not a C string; there is no terminating null, so the number of characters is determined from the atom's size. Legal characters are alphabetic (A-Z, a-z), digits (0-9), dash (-), period (.), underscore (_), and tilde (~).

Any track of a QuickTime file can be associated with one or more tags that indicate the media's characteristics. Tags indicate something of interest about a track. For example, a tag could indicate the purpose of the track (it is commentary), an abstract characteristic of the track (it requires hardware decoding), or an indication that the track includes legible text (a chapter track and subtitle track both can be read by the user).

Comparison of tags is case sensitive; two tags match if the bytes of the strings match exactly. Two tag strings differing only by case should not be used to avoid possible confusion for developers or content creators.

Duplicate tags in a single track are allowed but are discouraged. Duplication has no special meaning.

Tag strings are not localized and are meant to be machine interpreted; however, mnemonic strings are encouraged.

A tag is either public or private:

- Public tags allow shared semantics to be deployed widely. Public tags are currently defined by Apple.
- Private tags can be defined for private use.

Tag strings have the following structure:

- A public tag starts with the prefix “public.”, which is followed by one or more segments separated by periods. Examples (not defined) might be public.subtitle or public.commentary.director.

  Note: Public tags are public because they are documented in this specification or are available in Apple APIs. Other definitions of tags with the "public." prefix are prohibited; use private tags instead.

- A private tag starts with the private entity's domain using a reverse DNS naming convention. For example, apple.com becomes com.apple. This is followed by one or more segments separated by periods. Examples (not defined) might be com.apple.this-is-a-tag, com.apple.video.includes-sign-language, and org.w3c.html5.referenced-video.

2012-08-14 | Copyright © 2004, 2012 Apple Inc. All Rights Reserved.

- The only allowed prefixes are “public.” and reversed domains. All other prefixes are reserved for future use.

> **Note:** Generic top-level domains other than “public" (if it were to be assigned) are supported. The string “public" is reserved to signal public media characteristic tags.

This specification defines the following public media characteristic tags. Other public and private tags could be defined outside the specification; unrecognized tags should be ignored.

- public.auxiliary-content (valid for all media types)

  Indicates that the track’s content has been marked by the content author as auxiliary to the presentation of the media file. For example, a commentary audio or subtitle track might be marked with this tag, because it is not program content. If this tag is not present, a track can still be inferred to be tagged with this characteristic if the track is a member of an alternate group and the track is excluded from autoselection using the Track Exclude From Autoselection atom; see “Track Exclude From Autoselection Atoms” (page 61).

- public.accessibility.transcribes-spoken-dialog (valid for legible media)

  Indicates that the track includes legible content in the language of the track’s locale that transcribes spoken dialogue.

- public.accessibility.describes-music-and-sound (valid for legible media)

  Indicates that the track includes legible content in the language of the track’s locale that describes music and sound effects occurring in program audio.

- public.accessibility.describes-video (valid for audible media)

  Indicates that the track includes audible content that describes the visual portion of the presentation.

- public.easy-to-read (valid for legible media)

  Indicates that a track provides legible content in the language of its specified locale that has been edited for ease of reading.

Track Name

A movie atom’s user data atom may contain a track name atom (`'tnam'`).

The track name atom’s payload data consists of the following data.

- Reserved: 32-bit integer that must be set to zero.
- Language: 16-bit integer holding a packed ISO 639-2/T code as described in “User Data Text Strings and Language Codes” (page 51).

2012-08-14 | Copyright © 2004, 2012 Apple Inc. All Rights Reserved.

- Name: Null-terminated UTF-8 or UTF-16 string holding the track name. If this is a UTF-16 string, the string must start with a byte-order mark (0xFEFF).

A track can have multiple `'tnam'` atoms with different language codes. Normally it is sufficient for each track to have a single `'tnam'` atom in the same language as the track content. Alternate tracks might also have `'tnam'` atoms; their presence implies only that the name is a good user-readable label for the track.

Print to Video (Full Screen Mode)

A movie atom's user data atom may contain a print to video atom (`'ptv '`). Note that the fourth character is an ASCII blank (0x20). If a print to video atom is present, QuickTime plays the movie in full-screen mode, with no window and no visible controller. Any portion of the screen not occupied by the movie is cleared to black. The user must press the Esc (Escape) key to exit full-screen mode.

This atom is often added and removed transiently to control the display mode of a movie for a single presentation, but it can also be stored as part of the permanent movie file.

The print to video atom's payload data consists of the following.

Display size

A 16-bit *little-endian* integer indicating the display size for the movie: 0 indicates that the movie should be played at its normal size; 1 indicates that the movie should be played at double size; 2 indicates that the movie should be played at half size; 3 indicates that the movie should be scaled to fill the screen; 4 indicates that the movie should be played at its current size (this last value is normally used when the print to video atom is inserted transiently and the movie has been temporarily resized).

Reserved1

A 16-bit integer whose value should be 0.

Reserved2

A 16-bit integer whose value should be 0.

Slide show

An 8-bit Boolean whose value is 1 for a slide show. In slide show mode, the movie advances one frame each time the Right Arrow key is pressed. Audio is muted.

Play on open

An 8-bit Boolean whose value is normally 1, indicating that the movie should play when opened. Since there is no visible controller in full-screen mode, applications should always set this field to 1 to prevent user confusion.

2012-08-14 | Copyright © 2004, 2012 Apple Inc. All Rights Reserved.

Track Atoms

Track atoms define a single track of a movie. A movie may consist of one or more tracks. Each track is independent of the other tracks in the movie and carries its own temporal and spatial information. Each track atom contains its associated media atom.

Tracks are used specifically for the following purposes:

- To contain media data references and descriptions (media tracks).
- To contain modifier tracks (tweens, and so forth).
- To contain packetization information for streaming protocols (hint tracks). Hint tracks may contain references to media sample data or copies of media sample data. For more information about hint tracks, refer to "Hint Media" (page 251).

Note: A QuickTime movie cannot consist solely of hint tracks or modifier tracks; there must be at least one media track. Furthermore, media tracks cannot be deleted from a hinted movie, even if the hint tracks contain copies of the media sample data—in addition to the hint tracks, the entire unhinted movie must remain.

Figure 2-6 (page 56) shows the layout of a track atom. Track atoms have an atom type value of `'trak'`. The track atom requires a track header atom (`'tkhd'`) and a media atom (`'mdia'`). Other child atoms are optional, and may include a track clipping atom (`'clip'`), a track matte atom (`'matt'`), an edit atom (`'edts'`), a track reference atom (`'tref'`), a track load settings atom (`'load'`), a track input map atom (`'imap'`), and a user data atom (`'udta'`).

2012-08-14 | Copyright © 2004, 2012 Apple Inc. All Rights Reserved.

Note: Figure 2-6 (page 56) contains an optional track profile atom `'prfl'`. Track profile atoms are deprecated in the current version of QuickTime but may be present in existing QuickTime files. The inclusion here is intended to document existing content containing profile atoms, they should not be used for new development.

Figure 2-6 The layout of a track atom

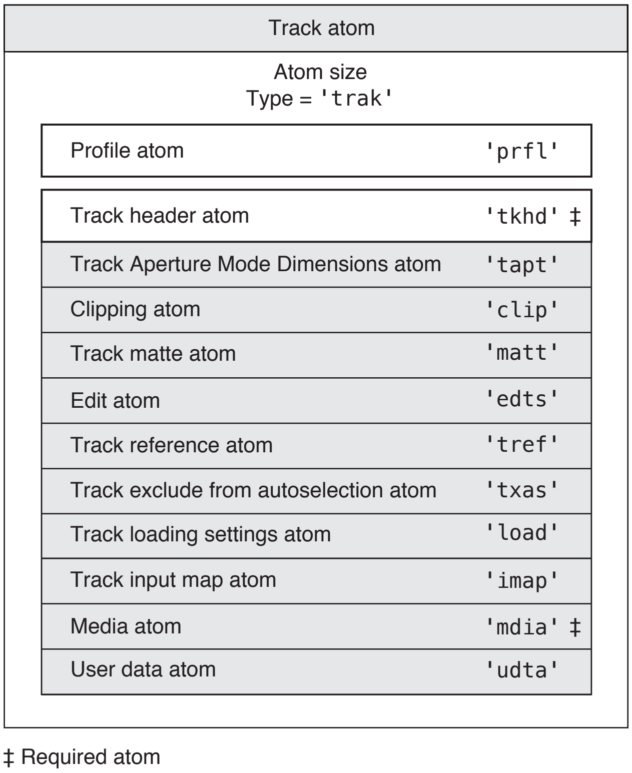

Track atoms contain the following data elements.

Size

A 32-bit integer that specifies the number of bytes in this track atom.

Type

A 32-bit integer that identifies the atom type; this field must be set to `'trak'`.

Track profile atom

See "Track Profile Atom" (page 57) for details.

Track header atom

See "Track Header Atoms" (page 57) for details.

Track aperture mode dimensions atom

See "Track Aperture Mode Dimension Atoms" (page 61) for details.

2012-08-14 | Copyright © 2004, 2012 Apple Inc. All Rights Reserved.

Clipping atom
: See “Clipping Atoms” (page 66) for more information.

Track matte atom
: See “Track Matte Atoms” (page 68) for more information.

Edit atom
: See “Edit Atoms” (page 69) for details.

Track reference atom
: See “Track Reference Atoms” (page 73)” for details.

Track exclude from autoselection atom
: See “Track Exclude From Autoselection Atoms” (page 61) for details.

Track load settings atom
: See “Track Load Settings Atoms” (page 71) for details.

Track input map atom
: See “Track Input Map Atoms” (page 76)” for details.

Media atom
: See “Media Atoms” (page 79) for details.

User-defined data atom
: See “User Data Atoms” (page 47) for more information.

Track Profile Atom

Note: Profile atoms are deprecated in the QuickTime file format. The information that follows is intended to document existing content containing profile atoms and should not be used for new development.

Profile atoms can be children of movie atoms or track atoms. For details on profile atoms, see “The Movie Profile Atom” (page 42).

Track Header Atoms

The track header atom specifies the characteristics of a single track within a movie. A track header atom contains a size field that specifies the number of bytes and a type field that indicates the format of the data (defined by the atom type `'tkhd'`).

Copyright © 2004, 2012 Apple Inc. All Rights Reserved.

[Figure 2-7](page 58) (page 58) shows the structure of the track header atom.

Figure 2-7 The layout of a track header atom

Track header atom	Bytes
Atom size	4
Type = `'tkhd'`	4
Version	1
Flags	3
Creation time	4
Modification time	4
Track ID	4
Reserved	4
Duration	4
Reserved	8
Layer	2
Alternate group	2
Volume	2
Reserved	2
Matrix structure	36
Track width	4
Track height	4

The track header atom contains the track characteristics for the track, including temporal, spatial, and volume information.

Track header atoms contain the following data elements.

Size

A 32-bit integer that specifies the number of bytes in this track header atom.

Type

A 32-bit integer that identifies the atom type; this field must be set to `'tkhd'`.

Version

A 1-byte specification of the version of this track header.

Flags

Three bytes that are reserved for the track header flags. These flags indicate how the track is used in the movie. The following flags are valid (all flags are enabled when set to 1).

Track enabled

Indicates that the track is enabled. Flag value is 0x0001.

Track in movie

Indicates that the track is used in the movie. Flag value is 0x0002.

Track in preview

Indicates that the track is used in the movie's preview. Flag value is 0x0004.

Track in poster

Indicates that the track is used in the movie's poster. Flag value is 0x0008.

Creation time

A 32-bit integer that indicates the calendar date and time (expressed in seconds since midnight, January 1, 1904) when the track header was created. It is strongly recommended that this value should be specified using coordinated universal time (UTC).

Modification time

A 32-bit integer that indicates the calendar date and time (expressed in seconds since midnight, January 1, 1904) when the track header was changed. It is strongly recommended that this value should be specified using coordinated universal time (UTC).

Track ID

A 32-bit integer that uniquely identifies the track. The value 0 cannot be used.

Reserved

A 32-bit integer that is reserved for use by Apple. Set this field to 0.

Duration

A time value that indicates the duration of this track (in the movie's time coordinate system). Note that this property is derived from the track's edits. The value of this field is equal to the sum of the durations of all of the track's edits. If there is no edit list, then the duration is the sum of the sample durations, converted into the movie timescale.

Reserved

An 8-byte value that is reserved for use by Apple. Set this field to 0.

Layer

A 16-bit integer that indicates this track's spatial priority in its movie. The QuickTime Movie Toolbox uses this value to determine how tracks overlay one another. Tracks with lower layer values are displayed in front of tracks with higher layer values.

2012-08-14 | Copyright © 2004, 2012 Apple Inc. All Rights Reserved.

Alternate group

A 16-bit integer that identifies a collection of movie tracks that contain alternate data for one another. This same identifier appears in each 'tkhd' atom of the other tracks in the group. QuickTime chooses one track from the group to be used when the movie is played. The choice may be based on such considerations as playback quality, language, or the capabilities of the computer.

A value of zero indicates that the track is not in an alternate track group.

The most common reason for having alternate tracks is to provide versions of the same track in different languages. Figure 2-8 (page 60) shows an example of several tracks. The video track's Alternate Group ID is 0, which means that it is not in an alternate group (and its language codes are empty; normally, video tracks should have the appropriate language tags). The three sound tracks have the same Group ID, so they form one alternate group, and the subtitle tracks have a different Group ID, so they form another alternate group. The tracks would not be adjacent in an actual QuickTime file; this is just a list of example track field values.

Figure 2-8 Example of alternate tracks in two alternate groups

Track Type	Alternate Group ID	Extended Language Tag	Language Code
video (vide)	0		
sound (soun)	1	en-US	eng
sound	1	fr-FR	fra
sound	1	jp-JP	jpn
subtitle (subt)	2	en-US	eng
subtitle	2	fr-FR	fra

Volume

A 16-bit fixed-point value that indicates how loudly this track's sound is to be played. A value of 1.0 indicates normal volume.

Reserved

A 16-bit integer that is reserved for use by Apple. Set this field to 0.

Matrix structure

The matrix structure associated with this track. See Figure 2-3 (page 44) for an illustration of a matrix structure.

Track width

A 32-bit fixed-point number that specifies the width of this track in pixels.

Track height

A 32-bit fixed-point number that indicates the height of this track in pixels.

2012-08-14 | Copyright © 2004, 2012 Apple Inc. All Rights Reserved.

Track Exclude From Autoselection Atoms

Some alternate tracks contain something other than a direct translation (or untranslated written form) of the primary content. Commentary tracks are one example. These tracks should not be automatically selected. The presence of the Track Exclude From Autoselection atom in a track indicates that this track should not be automatically selected.

Such tracks should have user-readable names that help users to identify the purpose of the track. These names are stored in one or more track name (`'tnam'`) atoms, each translated into a different language, within a user data (`'udta'`) atom within the `'trak'` atom.

The type of the Track Exclude From Autoselection atom is `'txas'`. This atom, if used, must be somewhere after the `'tkhd'` atom.

Track exclude from autoselection atoms contain the following data elements.

Size
: A 32-bit integer that specifies the number of bytes in the track exclude from autoselection atom. This must be 8, as this atom must contain no data.

Type
: A 32-bit integer that identifies the atom type; this field must be set to `'txas'`.

Track Aperture Mode Dimension Atoms

A video track in a QuickTime Movie can signal clean aperture and pixel aspect ratio information through image description extensions. The clean aperture defines the part of the encoded pixels to be displayed. The pixel aspect ratio is the aspect ratio of the encoded pixels. Conceptually the encoded pixels are decompressed, stretched (or shrunk) based on the pixel aspect ratio, and extra pixels are cropped off according to the clean aperture.

> **Note:** QuickTime tracks define simple dimensions for their content in the track header dimensions. In the absence of a track aperture mode dimensions atom, the dimensions in the track header are used for all modes.

Considering this context, the dimensions recorded in the image description define the dimensions of the encoded pixels (encoded dimensions). What's actually displayed is a result of applying the pixel aspect ratio and the clean aperture (display dimensions).

2012-08-14 | Copyright © 2004, 2012 Apple Inc. All Rights Reserved.

Although the result of applying the clean aperture and the pixel aspect ratio is what is intended for final display, there are cases where it is useful to display all the pixels that exist in the content for various different purposes. Readers parsing QuickTime movies require information allowing these different display modes in order to provide this flexibility:

Clean Mode
: In this mode both the clean aperture and the pixel aspect ratio are applied. The dimensions of the track become equal to the clean dimensions which are equal to the display dimensions (with conformed contents).

Production Mode
: This mode applies the pixel aspect ratio but not the clean aperture. The image is presented in the correct aspect ratio, but the extra pixels outside of the image that exists in the source material will be presented. The track dimensions are equal to the result of applying the pixel aspect ratio.

Classic Mode
: This mode displays the image without applying either the pixel aspect ratio or the clean aperture. The image is displayed using the track header dimensions, meaning the decompressed picture is scaled into the track header dimensions if the encoded dimensions are different.

Encoded Pixels
: The encoded pixels are displayed intact in this mode. Under this mode the track dimensions are equal to the encoded dimensions. No scaling or transformation takes place.

The information needed for each of these presentation modes are represented in the optional track aperture mode dimensions atoms.

Note: Older applications built prior to QuickTime 7 will continue to use the dimension values stored in the track header.

Track Aperture Mode Dimensions Atom

A container atom that stores information for video correction in the form of three required atoms. This atom is optionally included in the track atom. The type of the track aperture mode dimensions atom is `'tapt'`.

Copyright © 2004, 2012 Apple Inc. All Rights Reserved.

Figure 2-9 (page 63) shows the layout of the track aperture mode dimensions atom.

Figure 2-9 The layout of a track aperture mode dimensions atom

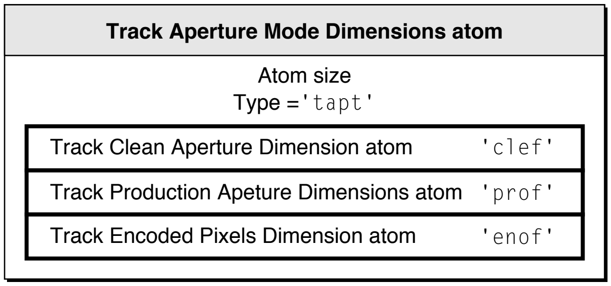

Size

A 32-bit integer that specifies the number of bytes in the track aperture mode dimensions atom.

Type

A 32-bit integer that identifies the atom type; this field must be set to `'tapt'`.

Track Clean Aperture Dimensions atom

See "Track Clean Aperture Dimensions Atom" (page 63)

Track Production Aperture Dimensions atom

See "Track Production Aperture Dimensions Atom" (page 64)

Track Encoded Pixels Dimensions atom

See "Track Encoded Pixels Dimensions Atom" (page 65)

Track Clean Aperture Dimensions Atom

This atom carries the pixel dimensions of the track's clean aperture. The type of the track clean aperture dimensions atom is `'clef'`.

Figure 2-10 (page 64) shows the layout of the track clean aperture dimensions atom.

Figure 2-10 The layout of a track clean aperture dimensions atom

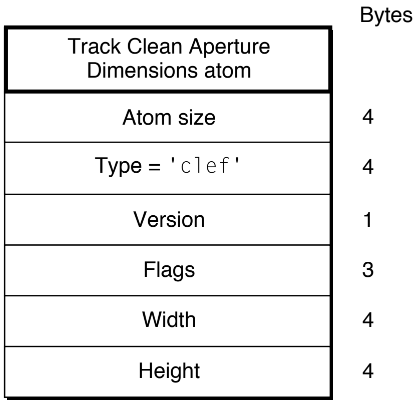

Size

A 32-bit integer that specifies the number of bytes in the track aperture mode dimensions atom.

Type

A 32-bit integer that identifies the atom type; this field must be set to `'clef'`.

Version

A 1-byte specification of the version of this atom.

Flags

Three bytes that are reserved for the atom flags.

Width

A 32-bit fixed-point number that specifies the width of the track clean aperture in pixels.

Height

A 32-bit fixed-point number that specifies the height of the track clean aperture in pixels.

Track Production Aperture Dimensions Atom

This atom carries the pixel dimensions of the track's production aperture. The type of the track production aperture dimensions atom is `'prof'`.

2012-08-14 | Copyright © 2004, 2012 Apple Inc. All Rights Reserved.

[Figure 2-11](page 65) shows the layout of the track production aperture dimensions atom.

Figure 2-11 The layout of a track production aperture dimensions atom

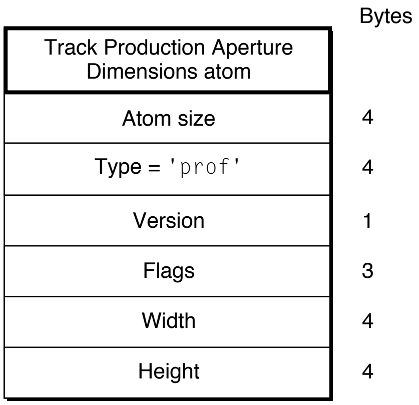

Size

A 32-bit integer that specifies the number of bytes in the track aperture mode dimensions atom.

Type

A 32-bit integer that identifies the atom type; this field must be set to `'prof'`.

Version

A 1-byte specification of the version of this atom.

Flags

Three bytes that are reserved for the atom flags.

Width

A 32-bit fixed-point number that specifies the width of the track production aperture in pixels.

Height

A 32-bit fixed-point number that specifies the height of the track production aperture in pixels.

Track Encoded Pixels Dimensions Atom

This atom carries the pixel dimensions of the track's encoded pixels. The type of the track encoded pixels dimensions atom is `'enof'`.

2012-08-14 | Copyright © 2004, 2012 Apple Inc. All Rights Reserved.

Figure 2-12 (page 66) shows the layout of this atom.

Figure 2-12 The layout of a track encoded pixels dimensions atom

Track Encoded Pixels Dimensions atom	Bytes
Atom size	4
Type = 'enof'	4
Version	1
Flags	3
Width	4
Height	4

Size
: A 32-bit integer that specifies the number of bytes in the track aperture mode dimensions atom.

Type
: A 32-bit integer that identifies the atom type; this field must be set to `‘enof’`.

Version
: A 1-byte specification of the version of this atom.

Flags
: Three bytes that are reserved for the atom flags.

Width
: A 32-bit fixed-point number that specifies the width of the track encoded pixels dimensions in pixels.

Height
: A 32-bit fixed-point number that specifies the height of the track encoded pixels dimensions in pixels.

Clipping Atoms

Clipping atoms specify the clipping regions for movies and for tracks. The clipping atom has an atom type value of `'clip'`.

Copyright © 2004, 2012 Apple Inc. All Rights Reserved.

Figure 2-13 (page 67) shows the layout of this atom.

Figure 2-13 The layout of a clipping atom

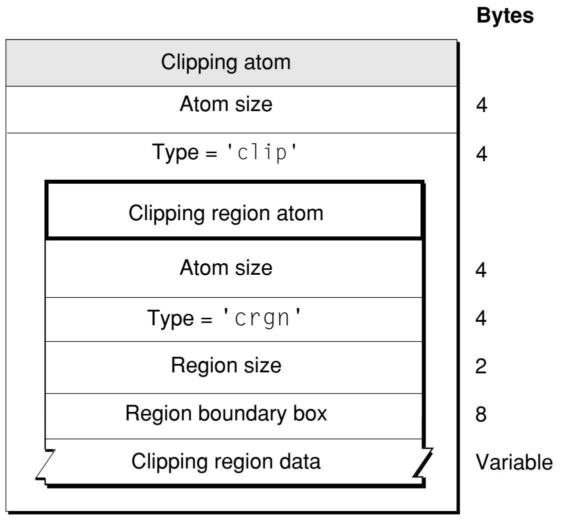

Clipping atoms contain the following data elements.

Size

A 32-bit integer that specifies the number of bytes in this clipping atom.

Type

A 32-bit integer that identifies the atom type; this field must be set to `'clip'`.

Clipping region atom

See "Clipping Region Atoms" (page 67).

Clipping Region Atoms

The clipping region atom contains the data that specifies the clipping region, including its size, bounding box, and region. Clipping region atoms have an atom type value of `'crgn'`.

The layout of the clipping region atom is shown in Figure 2-13 (page 67).

Clipping region atoms contain the following data elements.

Size

A 32-bit integer that specifies the number of bytes in this clipping region atom.

Type

A 32-bit integer that identifies the atom type; this field must be set to `'crgn'`.

Copyright © 2004, 2012 Apple Inc. All Rights Reserved.

Region size
: The region size, region boundary box, and clipping region data fields constitute a QuickDraw region.

Region boundary box
: The region size, region boundary box, and clipping region data fields constitute a QuickDraw region.

Clipping region data
: The region size, region boundary box, and clipping region data fields constitute a QuickDraw region.

Track Matte Atoms

Track matte atoms are used to visually blend the track's image when it is displayed.

Track matte atoms have an atom type value of `'matt'`.

Figure 2-14 (page 68) shows the layout of track matte atoms.

Figure 2-14 The layout of a track matte atom

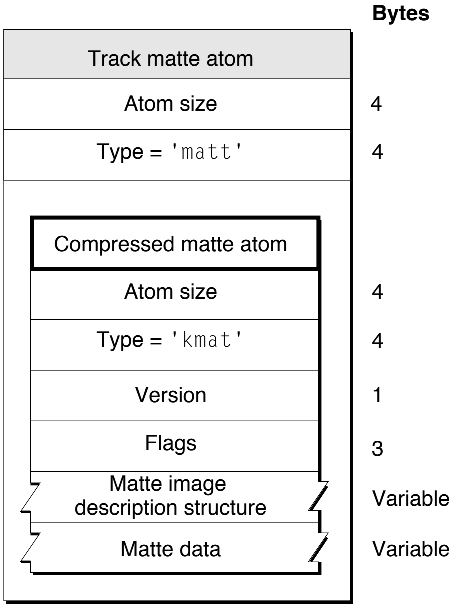

Track matte atoms contain the following data elements.

Size
: A 32-bit integer that specifies the number of bytes in this track matte atom.

Type
: A 32-bit integer that identifies the atom type; this field must be set to `'matt'`.

Copyright © 2004, 2012 Apple Inc. All Rights Reserved.

Compressed matte atom

The actual matte data.

See "Compressed Matte Atoms" (page 69) for details.

Compressed Matte Atoms

The compressed matte atom specifies the image description structure and the matte data associated with a particular matte atom. Compressed matte atoms have an atom type value of `'kmat'`.

The layout of the compressed matte atom is shown in Figure 2-14 (page 68).

Compressed matte atoms contain the following data elements.

Size

A 32-bit integer that specifies the number of bytes in this compressed matte atom.

Type

A 32-bit integer that identifies the atom type; this field must be set to `'kmat'`.

Version

A 1-byte specification of the version of this compressed matte atom.

Flags

Three bytes of space for flags. Set this field to 0.

Matte image description structure

An image description structure associated with this matte data. The image description contains detailed information that governs how the matte data is used. See "Video Sample Description" (page 155) for more information about image descriptions.

Matte data

The compressed matte data, which is of variable length.

Edit Atoms

You use edit atoms to define the portions of the media that are to be used to build up a track for a movie. The edits themselves are contained in an edit list table, which consists of time offset and duration values for each segment. Edit atoms have an atom type value of `'edts'`.

Figure 2-15 (page 70) shows the layout of an edit atom.

In the absence of an edit list, the presentation of a track starts immediately. An empty edit is used to offset the start time of a track.

2012-08-14 | Copyright © 2004, 2012 Apple Inc. All Rights Reserved.

Note: If the edit atom or the edit list atom is missing, you can assume that the entire media is used by the track.

Figure 2-15 The layout of an edit atom

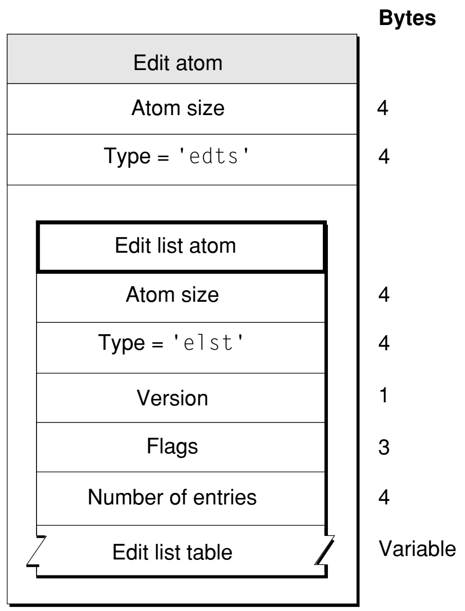

Edit atoms contain the following data elements.

Size

A 32-bit integer that specifies the number of bytes in this edit atom.

Type

A 32-bit integer that identifies the atom type; this field must be set to `'edts'`.

Edit list atom

See "Edit List Atoms" (page 70).

Edit List Atoms

You use the edit list atom, also shown in Figure 2-15 (page 70), to map from a time in a movie to a time in a media, and ultimately to media data. This information is in the form of entries in an edit list table, shown in Figure 2-16 (page 71). Edit list atoms have an atom type value of `'elst'`.

Edit list atoms contain the following data elements.

Size

A 32-bit integer that specifies the number of bytes in this edit list atom.

2012-08-14 | Copyright © 2004, 2012 Apple Inc. All Rights Reserved.

Type

A 32-bit integer that identifies the atom type; this field must be set to `'elst'`.

Version

A 1-byte specification of the version of this edit list atom.

Flags

Three bytes of space for flags. Set this field to 0.

Number of entries

A 32-bit integer that specifies the number of entries in the edit list atom that follows.

Edit list table

An array of 32-bit values, grouped into entries containing 3 values each. Figure 2-16 (page 71) shows the layout of the entries in this table.

Figure 2-16 The layout of an edit list table entry

Track duration	Media time	Media rate	Field
4	4	4	Bytes

An edit list table entry contains the following elements.

Track duration

A 32-bit integer that specifies the duration of this edit segment in units of the movie's time scale.

Media time

A 32-bit integer containing the starting time within the media of this edit segment (in media timescale units). If this field is set to –1, it is an empty edit. The last edit in a track should never be an empty edit. Any difference between the movie's duration and the track's duration is expressed as an implicit empty edit.

Media rate

A 32-bit fixed-point number that specifies the relative rate at which to play the media corresponding to this edit segment. This rate value cannot be 0 or negative.

Track Load Settings Atoms

Track load settings atoms contain information that indicates how the track is to be used in its movie. Applications that read QuickTime files can use this information to process the movie data more efficiently. Track load settings atoms have an atom type value of `'load'`.

2012-08-14 | Copyright © 2004, 2012 Apple Inc. All Rights Reserved.

Figure 2-17 (page 72) shows the layout of this atom.

Figure 2-17 The layout of a track load settings atom

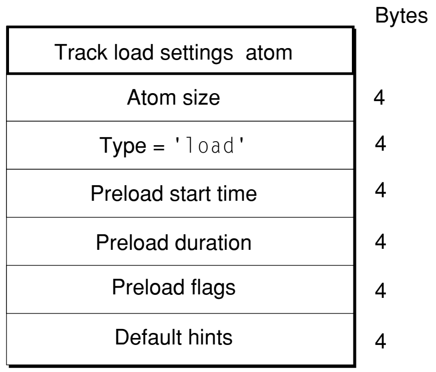

Track load settings atoms contain the following data elements.

Size
: A 32-bit integer that specifies the number of bytes in this track load settings atom.

Type
: A 32-bit integer that identifies the atom type; this field must be set to `'load'`.

Preload start time
: A 32-bit integer specifying the starting time, in the movie's time coordinate system, of a segment of the track that is to be preloaded. Used in conjunction with the preload duration.

Preload duration
: A 32-bit integer specifying the duration, in the movie's time coordinate system, of a segment of the track that is to be preloaded. If the duration is set to –1, it means that the preload segment extends from the preload start time to the end of the track. All media data in the segment of the track defined by the preload start time and preload duration values should be loaded into memory when the movie is to be played.

Preload flags
: A 32-bit integer containing flags governing the preload operation. Only two flags are defined, and they are mutually exclusive. If this flag is set to 1, the track is to be preloaded regardless of whether it is enabled. If this flag is set to 2, the track is to be preloaded only if it is enabled.

Copyright © 2004, 2012 Apple Inc. All Rights Reserved.

Default hints

A 32-bit integer containing playback hints. More than one flag may be enabled. Flags are enabled by setting them to 1. The following flags are defined.

Double buffer

This flag indicates that the track should be played using double-buffered I/O. This flag's value is 0x0020.

High quality

This flag indicates that the track should be displayed at highest possible quality, without regard to real-time performance considerations. This flag's value is 0x0100.

Track Reference Atoms

Track reference atoms define relationships between tracks. Track reference atoms allow one track to specify how it is related to other tracks. For example, if a movie has three video tracks and three sound tracks, track references allow you to identify the related sound and video tracks. Track reference atoms have an atom type value of `'tref'`.

Track references are unidirectional and point from the recipient track to the source track. For example, a video track may reference a time code track to indicate where its time code is stored, but the time code track would not reference the video track. The time code track is the source of time information for the video track.

A single track may reference multiple tracks. For example, a video track could reference a sound track to indicate that the two are synchronized and a time code track to indicate where its time code is stored.

A single track may also be referenced by multiple tracks. For example, both a sound and video track could reference the same time code track if they share the same timing information.

If this atom is not present, the track is not referencing any other track in any way. Note that the array of track reference type atoms is sized to fill the track reference atom. Track references with a reference index of 0 are permitted. This indicates no reference.

For more information about Track References, see "Track References" (page 248).

2012-08-14 | Copyright © 2004, 2012 Apple Inc. All Rights Reserved.

Figure 2-18 (page 74) shows the layout of a track reference atom.

Figure 2-18 The layout of a track reference atom

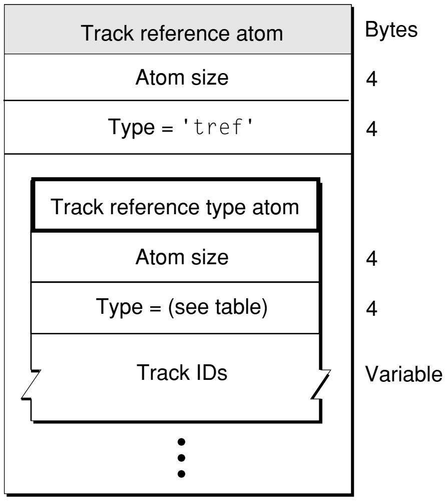

A track reference atom contains the following data elements.

Size

A 32-bit integer that specifies the number of bytes in this track reference atom.

Type

A 32-bit integer that identifies the atom type; this field must be set to `'tref'`.

Track reference type atoms

A list of track reference type atoms containing the track reference information. These atoms are described next.

Each track reference atom defines relationships with tracks of a specific type. The reference type implies a track type. Table 2-2 (page 74) shows the track reference types and their descriptions.

Table 2-2 Track reference types

Reference type	Description
`'chap'`	Chapter or scene list. Usually references a text track.
`'clcp'`	Closed caption. In any track, this identifies a closed captioning track that contains text that is appropriate for the referring track. See "Closed Captioning Media" (page 198) for more information.

Copyright © 2004, 2012 Apple Inc. All Rights Reserved.

Reference type	Description
'fall'	In a sound track, this references a track in a different format but with identical content, if one exists; for example, an AC3 track might reference an AAC track with identical content. See "Alternate Sound Tracks" (page 335).
'folw'	In a sound track, this references a subtitle track that is to be used as the sound track's default subtitle track. If the subtitle track is part of a subtitle track pair, this should reference the the forced subtitle track of the pair. This is needed only if language tagging cannot be used. See "Relationships Across Alternate Groups" (page 336).
'forc'	Forced subtitle track. In the regular track of a subtitle track pair, this references the forced track. See "Subtitle Sample Data" (page 202) for more information.
'hint'	The referenced tracks contain the original media for this hint track.
'scpt'	Transcript. Usually references a text track.
'ssrc'	Non-primary source. Indicates that the referenced track should send its data to this track, rather than presenting it. The referencing track will use the data to modify how it presents its data. See "Track Input Map Atoms" (page 76) for more information.
'sync'	Synchronization. Usually between a video and sound track. Indicates that the two tracks are synchronized. The reference can be from either track to the other, or there may be two references.
'tmcd'	Time code. Usually references a time code track.

Each track reference type atom contains the following data elements.

Size

A 32-bit integer that specifies the number of bytes in this track reference type atom.

Type

A 32-bit integer that identifies the atom type; this field must be set to one of the values shown in Table 2-2 (page 74).

Track IDs

A list of track ID values (32-bit integers) specifying the related tracks. Note that this is one case where track ID values can be set to 0. Unused entries in the atom may have a track ID value of 0. Setting the track ID to 0 may be more convenient than deleting the reference.

You can determine the number of track references stored in a track reference type atom by subtracting its header size from its overall size and then dividing by the size, in bytes, of a track ID.

2012-08-14 | Copyright © 2004, 2012 Apple Inc. All Rights Reserved.

Track Input Map Atoms

Track input map atoms define how data being sent to this track from its nonprimary sources is to be interpreted. Track references of type 'ssrc' define a track's secondary data sources. These sources provide additional data that is to be used when processing the track. Track input map atoms have an atom type value of 'imap'.

Figure 2-19 (page 76) shows the layout of a track input atom. This atom contains one or more track input atoms. Note that the track input map atom is a QT atom structure.

Figure 2-19 The layout of a track input map atom

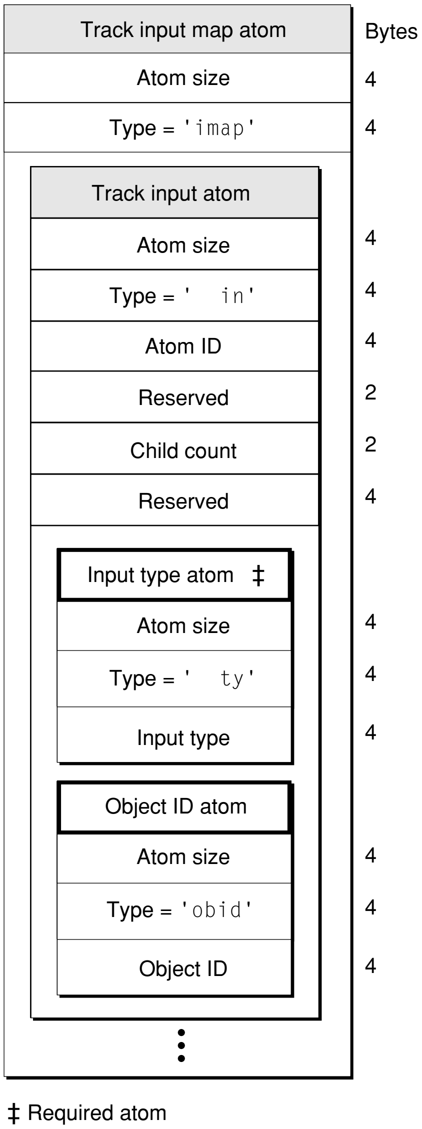

Each track input map atom contains the following data elements.

Size
: A 32-bit integer that specifies the number of bytes in this track input map atom.

Type
: A 32-bit integer that identifies the atom type; this field must be set to `'imap'`.

Track input atoms
: A list of track input atoms specifying how to use the input data.

The input map defines all of the track's secondary inputs. Each secondary input is defined using a separate track input atom.

Each track input atom contains the following data elements.

Size
: A 32-bit integer that specifies the number of bytes in this track input atom.

Type
: A 32-bit integer that identifies the atom type; this field must be set to `'  in'` (note that the two leading bytes must be set to 0x00).

Atom ID
: A 32-bit integer relating this track input atom to its secondary input. The value of this field corresponds to the index of the secondary input in the track reference atom. That is, the first secondary input corresponds to the track input atom with an atom ID value of 1; the second to the track input atom with an atom ID of 2, and so on.

Reserved
: A 16-bit integer that must be set to 0.

Child count
: A 16-bit integer specifying the number of child atoms in this atom.

Reserved
: A 32-bit integer that must be set to 0.

The track input atom, in turn, may contain two other types of atoms: input type atoms and object ID atoms. The input type atom is required; it specifies how the data is to be interpreted.

The input type atom contains the following data elements.

Size
: A 32-bit integer that specifies the number of bytes in this input type atom.

Type
: A 32-bit integer that identifies the atom type; this field must be set to `'  ty'` (note that the two leading bytes must be set to 0x00).

2012-08-14 | Copyright © 2004, 2012 Apple Inc. All Rights Reserved.

Input type

A 32-bit integer that specifies the type of data that is to be received from the secondary data source. Table 2-3 (page 78) lists valid values for this field.

Table 2-3 Input types

Input identifier	Value	Description
`kTrackModifier-TypeMatrix`	1	A 3 × 3 transformation matrix to transform the track's location, scaling, and so on.
`kTrackModifierTypeClip`	2	A QuickDraw clipping region to change the track's shape.
`kTrackModifier-TypeVolume`	3	An 8.8 fixed-point value indicating the relative sound volume. This is used for fading the volume.
`kTrackModifier-TypeBalance`	4	A 16-bit integer indicating the sound balance level. This is used for panning the sound location.
`kTrackModifierType-GraphicsMode`	5	A graphics mode record (32-bit integer indicating graphics mode, followed by an RGB color) to modify the track's graphics mode for visual fades.
`kTrackModifier-ObjectMatrix`	6	A 3 × 3 transformation matrix to transform an object within the track's location, scaling, and so on.
`kTrackModifierObject-GraphicsMode`	7	A graphics mode record (32-bit integer indicating graphics mode, followed by an RGB color) to modify an object within the track's graphics mode for visual fades.
`kTrackModifierTypeImage`	`'vide'`	Compressed image data for an object within the track. Note that this was `kTrackModifierType-SpriteImage`.

If the input is operating on an object within the track (for example, a sprite within a sprite track), an object ID atom must be included in the track input atom to identify the object.

The object ID atom contains the following data elements.

Size

A 32-bit integer that specifies the number of bytes in this object ID atom.

Type

A 32-bit integer that identifies the atom type; this field must be set to `'obid'`.

2012-08-14 | Copyright © 2004, 2012 Apple Inc. All Rights Reserved.

Object ID

A 32-bit integer identifying the object.

Media Atoms

Media atoms describe and define a track's media type and sample data. The media atom contains information that specifies:

- The media type, such as sound or video
- The media handler component used to interpret the sample data
- The media timescale and track duration
- Media-and-track-specific information, such as sound volume or graphics mode
- The media data references, which typically specify the file where the sample data is stored
- The sample table atoms, which, for each media sample, specify the sample description, duration, and byte offset from the data reference

The media atom has an atom type of `'mdia'`. It must contain a media header atom (`'mdhd'`), and it can contain a handler reference (`'hdlr'`) atom, media information (`'minf'`) atom, and user data (`'udta'`) atom.

Note: Do not confuse the media atom (`'mdia'`) with the media *data* atom (`'mdat'`). The media atom contains only *references* to media data; the media data atom contains the actual media samples.

Figure 2-20 (page 79) shows the layout of a media atom.

Figure 2-20 The layout of a media atom

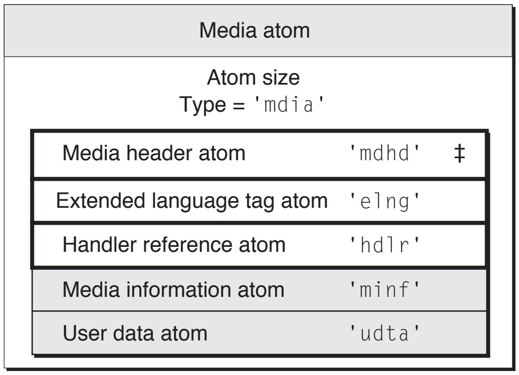

‡ Required atom

2012-08-14 | Copyright © 2004, 2012 Apple Inc. All Rights Reserved.

Media atoms contain the following data elements.

Size

A 32-bit integer that specifies the number of bytes in this media atom.

Type

A 32-bit integer that identifies the atom type; this field must be set to `'mdia'`.

Media header atom

This atom contains the standard media information. See "Media Header Atoms" (page 80).

Extended language tag atom

This atom contains the extended language tag describing the media language. See "Extended Language Tag Atom" (page 82).

Handler reference atom

This atom identifies the media handler component that is to be used to interpret the media data. See "Handler Reference Atoms" (page 84) for more information.

Note that the handler reference atom tells you the kind of media this media atom contains—for example, video or sound. The layout of the media information atom is specific to the media handler that is to interpret the media. "Media Information Atoms" (page 85) discusses how data may be stored in a media, using the video media format defined by Apple as an example.

Media information atom

This atom contains data specific to the media type for use by the media handler component. See "Media Information Atoms" (page 85).

User data atom

See "User Data Atoms" (page 47).

Media Header Atoms

The media header atom specifies the characteristics of a media, including time scale and duration. The media header atom has an atom type of `'mdhd'`.

2012-08-14 | Copyright © 2004, 2012 Apple Inc. All Rights Reserved.

Figure 2-21 (page 81) shows the layout of the media header atom.

Figure 2-21 The layout of a media header atom

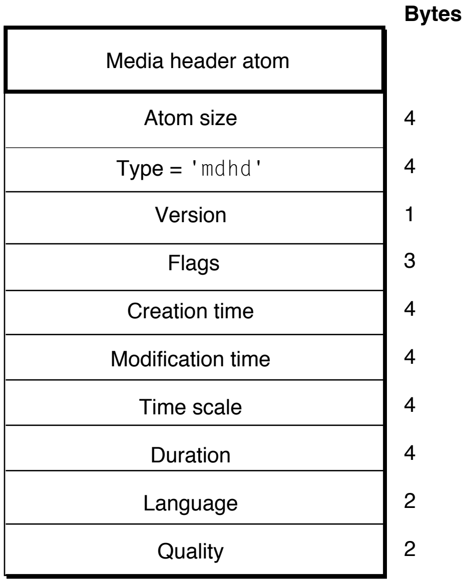

Media header atoms contain the following data elements.

Size
: A 32-bit integer that specifies the number of bytes in this media header atom.

Type
: A 32-bit integer that identifies the atom type; this field must be set to `'mdhd'`.

Version
: One byte that specifies the version of this header atom.

Flags
: Three bytes of space for media header flags. Set this field to 0.

Creation time
: A 32-bit integer that specifies (in seconds since midnight, January 1, 1904) when the media atom was created. It is strongly recommended that this value should be specified using coordinated universal time (UTC).

Modification time
: A 32-bit integer that specifies (in seconds since midnight, January 1, 1904) when the media atom was changed. It is strongly recommended that this value should be specified using coordinated universal time (UTC).

2012-08-14 | Copyright © 2004, 2012 Apple Inc. All Rights Reserved.

Time scale

A time value that indicates the time scale for this media—that is, the number of time units that pass per second in its time coordinate system.

Duration

The duration of this media in units of its time scale.

Language

A 16-bit integer that specifies the language code for this media. See "Language Code Values" (page 316) for valid language codes. Also see "Extended Language Tag Atom" (page 82) for the preferred code to use here if an extended language tag is also included in the media atom.

Quality

A 16-bit integer that specifies the media's playback quality—that is, its suitability for playback in a given environment.

Extended Language Tag Atom

The extended language tag atom represents media language information based on the RFC 4646 (Best Common Practices (BCP) #47) industry standard. It is an optional peer of the media header atom and should follow the definition of the media header atom in a QuickTime movie. There is at most one extended language tag atom per media atom and, in turn, per track. The extended language tag atom has an atom type of `'elng'`.

Until the introduction of this atom type, QuickTime had support for languages via codes based on either ISO 639 or the classic "Macintosh Language Codes" (page 317). These language codes are associated to a media (per track) in a QuickTime movie and are referred to as the **media language**.

To distinguish the extended language support from the old system, it is referred to as the *extended language tag* as opposed to *language code*. The major advantage of the extended language tag is that it includes additional information such as region, script, variation, and so on, as parts (or subtags). For instance, this additional information allows distinguishing content in French as spoken in Canada from content in French as spoken in France.

2012-08-14 | Copyright © 2004, 2012 Apple Inc. All Rights Reserved.

Figure 2-22 (page 83) shows the layout of this atom.

Figure 2-22 The layout of an extended language tag atom

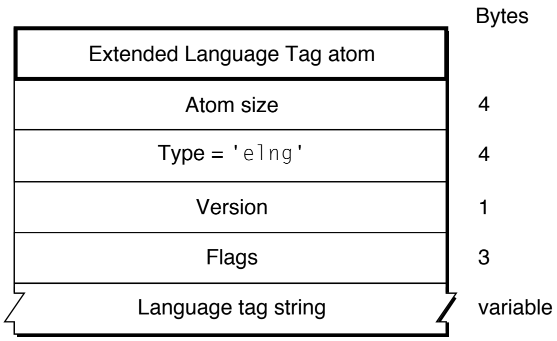

Extended language tag atoms contain the following data elements.

Size

A 32-bit integer that specifies the number of bytes in this media header atom.

Type

A 32-bit integer that identifies the atom type; this field must be set to `'elng'`.

Version

One byte that specifies the version of this header atom.

Flags

Three bytes of space for media header flags. Set this field to 0.

Language tag string

A NULL-terminated C string containing an RFC 4646 (BCP 47) compliant language tag string in ASCII encoding, such as "en-US", "fr-FR", or "zh-CN".

Additional notes:

- The extended language tag overrides the media language if they are not consistent.
- The extended language tag atom is optional, and if it is absent the media language should be used.
- No validation of the language tag string is performed. Applications parsing QuickTime movies need to be prepared for an invalid language tag, and are expected to behave as if no information is found.
- For best compatibility with earlier players, if an extended language tag is specified, the most compatible language code should be specified in the language field of the 'mdhd' atom (for example, "eng" if the extended language tag is "en-UK"). If there is no reasonably compatible tag, the packed form of 'und' can be specified in the language code of the 'mdhd' atom.

2012-08-14 | Copyright © 2004, 2012 Apple Inc. All Rights Reserved.

Handler Reference Atoms

The handler reference atom specifies the media handler component that is to be used to interpret the media's data. The handler reference atom has an atom type value of `'hdlr'`.

Historically, the handler reference atom was also used for data references. However, this use is no longer current and may now be safely ignored.

The handler atom within a media atom declares the process by which the media data in the stream may be presented, and thus, the nature of the media in a stream. For example, a video handler would handle a video track.

Figure 2-23 (page 84) shows the layout of a handler reference atom.

Figure 2-23 The layout of a handler reference atom

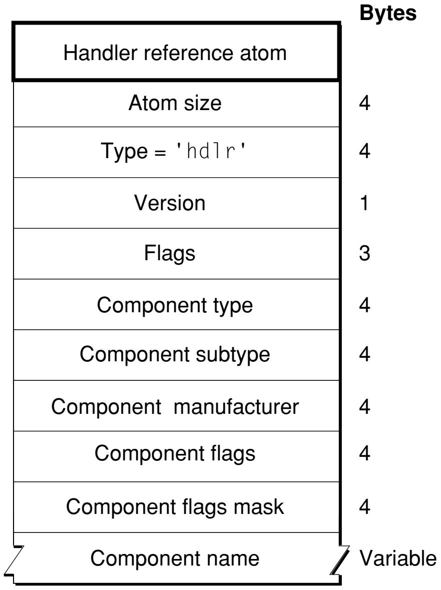

Handler reference atoms contain the following data elements.

Size

A 32-bit integer that specifies the number of bytes in this handler reference atom.

Type

A 32-bit integer that identifies the atom type; this field must be set to `'hdlr'`.

Version

A 1-byte specification of the version of this handler information.

Flags

A 3-byte space for handler information flags. Set this field to 0.

2012-08-14 | Copyright © 2004, 2012 Apple Inc. All Rights Reserved.

Component type

A four-character code that identifies the type of the handler. Only two values are valid for this field: `'mhlr'` for media handlers and `'dhlr'` for data handlers.

Component subtype

A four-character code that identifies the type of the media handler or data handler. For media handlers, this field defines the type of data—for example, `'vide'` for video data, `'soun'` for sound data or `‘subt’` for subtitles. See “Media Data Atom Types” (page 154) for information about defined media data types.

For data handlers, this field defines the data reference type; for example, a component subtype value of `'alis'` identifies a file alias.

Component manufacturer

Reserved. Set to 0.

Component flags

Reserved. Set to 0.

Component flags mask

Reserved. Set to 0.

Component name

A (counted) string that specifies the name of the component—that is, the media handler used when this media was created. This field may contain a zero-length (empty) string.

Media Information Atoms

Media information atoms (defined by the `'minf'` atom type) store handler-specific information for a track’s media data. The media handler uses this information to map from media time to media data and to process the media data.

These atoms contain information that is specific to the type of data defined by the media. Further, the format and content of media information atoms are dictated by the media handler that is responsible for interpreting the media data stream. Another media handler would not know how to interpret this information.

This section describes the atoms that store media information for the video (`'vmhd'`), sound (`'smhd'`), and base (`'gmhd'`) portions of QuickTime movies.

2012-08-14 | Copyright © 2004, 2012 Apple Inc. All Rights Reserved.

Note: "Using Sample Atoms " (page 116) discusses how the video media handler locates samples in a video media.

Video Media Information Atoms

Video media information atoms are the highest-level atoms in video media. These atoms contain a number of other atoms that define specific characteristics of the video media data. Figure 2-24 (page 86) shows the layout of a video media information atom.

Figure 2-24 The layout of a media information atom for video

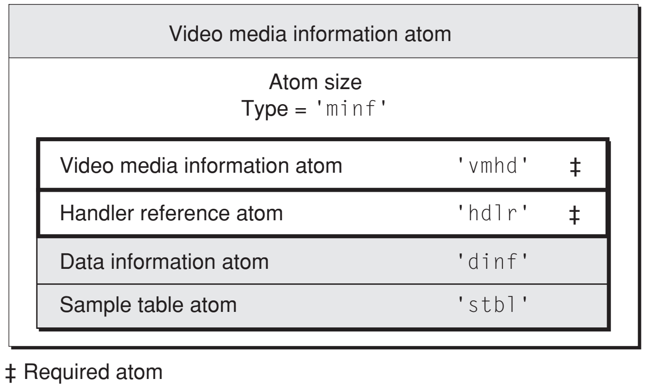

The video media information atom contains the following data elements.

Size

A 32-bit integer that specifies the number of bytes in this video media information atom.

Type

A 32-bit integer that identifies the atom type; this field must be set to `'minf'`.

Video media information atom

See "Video Media Information Header Atoms" (page 87).

Handler reference atom

See "Handler Reference Atoms" (page 84).

Data information atom

See "Data Information Atoms" (page 92).

Sample table atom

See "Sample Table Atoms" (page 96).

Video Media Information Header Atoms

Video media information header atoms define specific color and graphics mode information.

Figure 2-25 (page 87) shows the structure of a video media information header atom.

Figure 2-25 The layout of a media information header atom for video

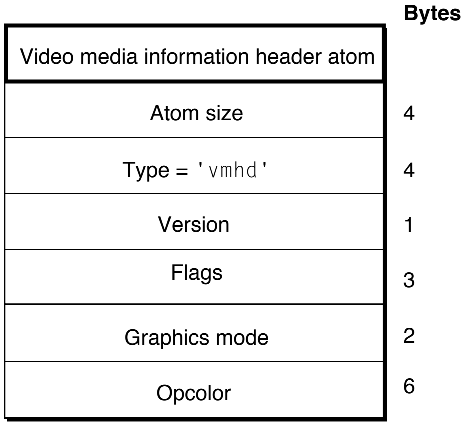

The video media information header atom contains the following data elements.

Size
: A 32-bit integer that specifies the number of bytes in this video media information header atom.

Type
: A 32-bit integer that identifies the atom type; this field must be set to `'vmhd'`.

Version
: A 1-byte specification of the version of this video media information header atom.

Flags
: A 3-byte space for video media information flags. There is one defined flag.

No lean ahead

This is a compatibility flag that allows QuickTime to distinguish between movies created with QuickTime 1.0 and newer movies. You should always set this flag to 1, unless you are creating a movie intended for playback using version 1.0 of QuickTime. This flag's value is 0x0001.

Graphics mode
: A 16-bit integer that specifies the transfer mode. The transfer mode specifies which Boolean operation QuickDraw should perform when drawing or transferring an image from one location to another. See "Graphics Modes" (page 321) for a list of graphics modes supported by QuickTime.

Copyright © 2004, 2012 Apple Inc. All Rights Reserved.

Opcolor
: Three 16-bit values that specify the red, green, and blue colors for the transfer mode operation indicated in the graphics mode field.

Sound Media Information Atoms

Sound media information atoms are the highest-level atoms in sound media. These atoms contain a number of other atoms that define specific characteristics of the sound media data. Figure 2-26 (page 88) shows the layout of a sound media information atom.

Figure 2-26 The layout of a media information atom for sound

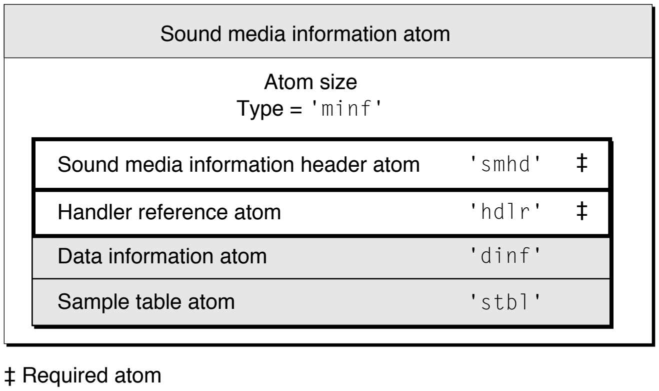

The sound media information atom contains the following data elements.

Size
: A 32-bit integer that specifies the number of bytes in this sound media information atom.

Type
: A 32-bit integer that identifies the atom type; this field must be set to `'minf'`.

Sound media information header atom
: See “Sound Media Information Header Atoms” (page 89).

Handler reference atom
: See “Handler Reference Atoms” (page 84).

Data information atom
: See “Data Information Atoms” (page 92).

Sample table atom
: See “Sample Table Atoms” (page 96).

Copyright © 2004, 2012 Apple Inc. All Rights Reserved.

Sound Media Information Header Atoms

The sound media information header atom stores the sound media's control information, such as balance.

Figure 2-27 (page 89) shows the layout of this atom.

Figure 2-27 The layout of a sound media information header atom

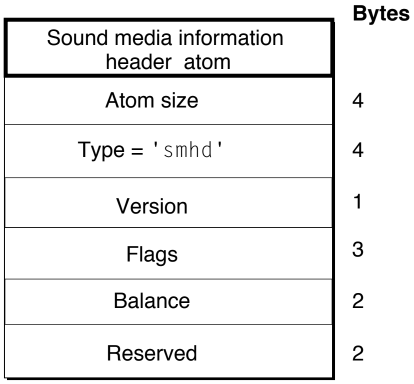

The sound media information header atom contains the following data elements.

Size
: A 32-bit integer that specifies the number of bytes in this sound media information header atom.

Type
: A 32-bit integer that identifies the atom type; this field must be set to `'smhd'`.

Version
: A 1-byte specification of the version of this sound media information header atom.

Flags
: A 3-byte space for sound media information flags. Set this field to 0.

Balance
: A 16-bit integer that specifies the sound balance of this sound media. Sound balance is the setting that controls the mix of sound between the two speakers of a computer. This field is normally set to 0. See "Balance" (page 323) for more information about balance values.

Reserved
: Reserved for use by Apple. Set this field to 0.

Base Media Information Atoms

The base media information atom (shown in Figure 2-28 (page 90)) stores the media information for media types such as text, MPEG, time code, and music.

Copyright © 2004, 2012 Apple Inc. All Rights Reserved.

Media types that are derived from the base media handler may add other atoms within the base media information atom, as appropriate. At present, the only media type that defines additional atoms is timecode media. See “Timecode Media” (page 189) for more information about this media types.

Figure 2-28 The layout of a base media information atom

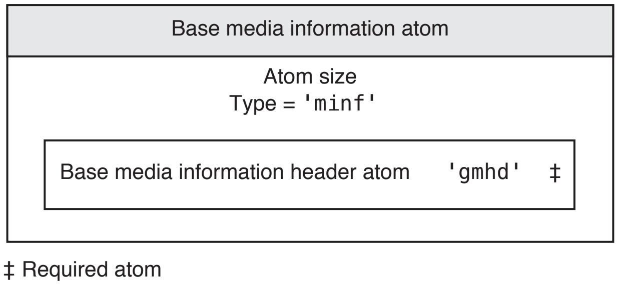

The base media information atom contains the following data elements.

Size
: A 32-bit integer that specifies the number of bytes in this base media information atom.

Type
: A 32-bit integer that identifies the atom type; this field must be set to `'minf'`.

Base media information header atom
: See “Base Media Information Header Atoms” (page 90).

Base Media Information Header Atoms

The base media information header atom indicates that this media information atom pertains to a base media.

Figure 2-29 (page 90) shows the layout of this atom.

Figure 2-29 The layout of a base media information header atom

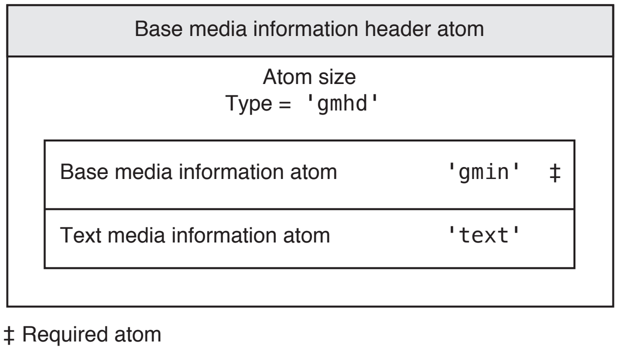

2012-08-14 | Copyright © 2004, 2012 Apple Inc. All Rights Reserved.

The base media information header atom contains the following data elements.

Size
: A 32-bit integer that specifies the number of bytes in this base media information header atom.

Type
: A 32-bit integer that identifies the atom type; this field must be set to `'gmhd'`.

Base media info atom
: See "Base Media Info Atoms" (page 91).

Text media information atom
: See "Text Media Information Atom" (page 196).

Base Media Info Atoms

The base media info atom, contained in the base media information header atom (`'gmhd'`), defines the media's control information, including graphics mode and balance information.

Figure 2-30 (page 91) shows the layout of the base media info atom.

Figure 2-30 The layout of a base media info atom

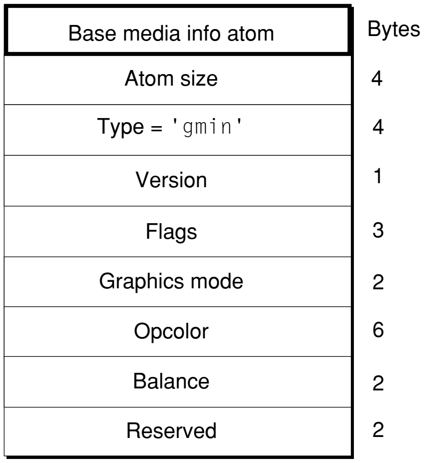

The base media info atom contains the following data elements.

Size
: A 32-bit integer that specifies the number of bytes in this base media info atom.

Type
: A 32-bit integer that identifies the atom type; this field must be set to `'gmin'`.

Version
: A 1-byte specification of the version of this base media information header atom.

2012-08-14 | Copyright © 2004, 2012 Apple Inc. All Rights Reserved.

Flags
: A 3-byte space for base media information flags. Set this field to 0.

Graphics mode
: A 16-bit integer that specifies the transfer mode. The transfer mode specifies which Boolean operation QuickDraw should perform when drawing or transferring an image from one location to another. See "Graphics Modes" (page 321) for more information about graphics modes supported by QuickTime.

Opcolor
: Three 16-bit values that specify the red, green, and blue colors for the transfer mode operation indicated in the graphics mode field.

Balance
: A 16-bit integer that specifies the sound balance of this media. Sound balance is the setting that controls the mix of sound between the two speakers of a computer. This field is normally set to 0. See "Balance" (page 323) for more information about balance values.

Reserved
: Reserved for use by Apple. Set this field to 0.

Data Information Atoms

The handler reference atom (described in "Handler Reference Atoms" (page 84)) contains information specifying the data handler component that provides access to the media data. The data handler component uses the data information atom to interpret the media's data. Data information atoms have an atom type value of `'dinf'`.

2012-08-14 | Copyright © 2004, 2012 Apple Inc. All Rights Reserved.

Figure 2-31 (page 93) shows the layout of the data information atom.

Figure 2-31 The layout of a data information atom

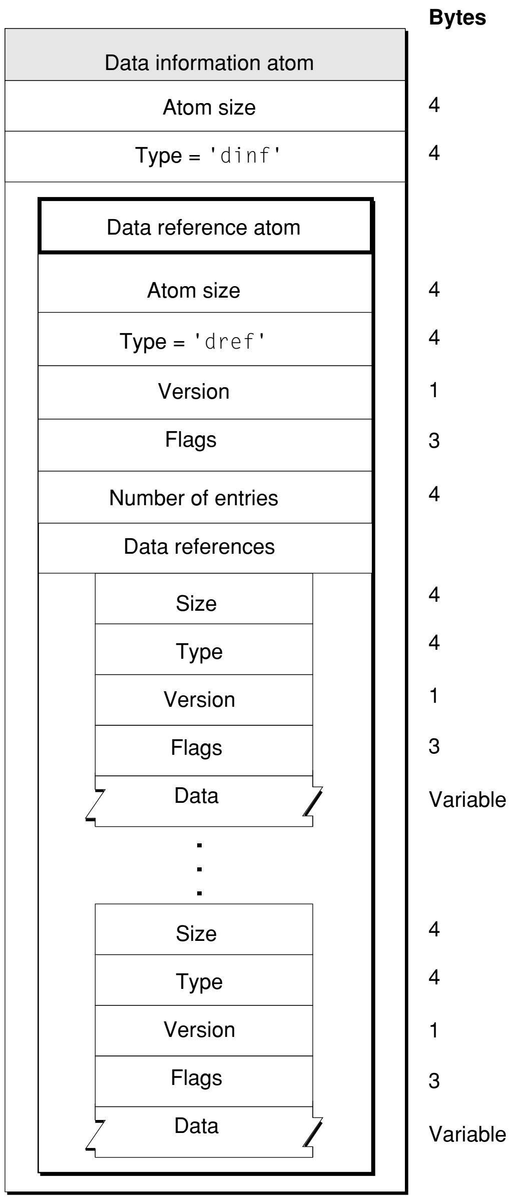

The data information atom contains the following data elements.

Size

A 32-bit integer that specifies the number of bytes in this data information atom.

Type

A 32-bit integer that identifies the atom type; this field must be set to `'dinf'`.

Copyright © 2004, 2012 Apple Inc. All Rights Reserved.

Data reference atom

See "Data Reference Atoms" (page 94).

Data Reference Atoms

Data reference atoms contain tabular data that instructs the data handler component how to access the media's data. Figure 2-31 (page 93) shows the data reference atom.

The data reference atom contains the following data elements.

Size

A 32-bit integer that specifies the number of bytes in this data reference atom.

Type

A 32-bit integer that identifies the atom type; this field must be set to `'dref'`.

Version

A 1-byte specification of the version of this data reference atom.

Flags

A 3-byte space for data reference flags. Set this field to 0.

Number of entries

A 32-bit integer containing the count of data references that follow.

Data references

An array of data references.

Each data reference is formatted like an atom and contains the following data elements.

Size

A 32-bit integer that specifies the number of bytes in the data reference.

Type

A 32-bit integer that specifies the type of the data in the data reference. Table 2-4 (page 95) lists valid type values.

Version

A 1-byte specification of the version of the data reference.

2012-08-14 | Copyright © 2004, 2012 Apple Inc. All Rights Reserved.

Flags

A 3-byte space for data reference flags. There is one defined flag.

Self reference

This flag indicates that the media's data is in the same file as the movie atom. On the Macintosh, and other file systems with multi-fork files, set this flag to 1 even if the data resides in a different fork from the movie atom. This flag's value is 0x0001.

Data

The data reference information.

Table 2-4 (page 95) shows the currently defined data reference types that can be stored in a header atom.

Table 2-4 Data reference types

Data reference type	Description
`'alis'`	Data reference is a Macintosh alias. An alias contains information about the file it refers to, including its full path name.
`'rsrc'`	Data reference is a Macintosh alias. Appended to the end of the alias is the resource type (stored as a 32-bit integer) and ID (stored as a 16-bit signed integer) to use within the specified file. This data reference type is deprecated in the QuickTime file format. This information is intended to document existing content containing 'rsrc' data references and should not be used for new development.
`'url '`	A C string that specifies a URL. There may be additional data after the C string.

Sample Atoms

QuickTime stores media data in samples. A sample is a single element in a sequence of time-ordered data. Samples are stored in the media, and they may have varying durations.

2012-08-14 | Copyright © 2004, 2012 Apple Inc. All Rights Reserved.

Samples are stored in a series of chunks in a media. Chunks are a collection of data samples in a media that allow optimized data access. A chunk may contain one or more samples. Chunks in a media may have different sizes, and the individual samples within a chunk may have different sizes from one another, as shown in Figure 2-32 (page 96).

Figure 2-32 Samples in a media

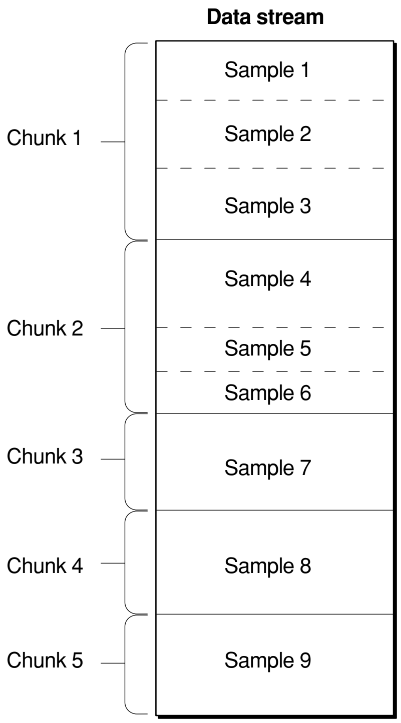

One way to describe a sample is to use a sample table atom. The sample table atom acts as a storehouse of information about the samples and contains a number of different types of atoms. The various atoms contain information that allows the media handler to parse the samples in the proper order. This approach enforces an ordering of the samples without requiring that the sample data be stored sequentially with respect to movie time in the actual data stream.

The next section discusses the sample table atom. Subsequent sections discuss each of the atoms that may reside in a sample table atom.

Sample Table Atoms

The sample table atom contains information for converting from media time to sample number to sample location. This atom also indicates how to interpret the sample (for example, whether to decompress the video data and, if so, how). This section describes the format and content of the sample table atom.

2012-08-14 | Copyright © 2004, 2012 Apple Inc. All Rights Reserved.

The sample table atom has an atom type of `'stbl'`. It can contain the sample description atom, the time-to-sample atom, the sync sample atom, the sample-to-chunk atom, the sample size atom, the chunk offset atom, and the shadow sync atom. Recent additions to the list of atom types that a sample table atom can contain are the optional sample group description and sample-to-group atoms included in Appendix G: "Audio Priming - Handling Encoder Delay in AAC" (page 421).

The sample table atom contains all the time and data indexing of the media samples in a track. Using tables, it is possible to locate samples in time, determine their type, and determine their size, container, and offset into that container.

If the track that contains the sample table atom references no data, then the sample table atom does not need to contain any child atoms (not a very useful media track).

If the track that the sample table atom is contained in does reference data, then the following child atoms are required: sample description, sample size, sample to chunk, and chunk offset. All of the subtables of the sample table use the same total sample count.

The sample description atom must contain at least one entry. A sample description atom is required because it contains the data reference index field that indicates which data reference atom to use to retrieve the media samples. Without the sample description, it is not possible to determine where the media samples are stored. The sync sample atom is optional. If the sync sample atom is not present, all samples are implicitly sync samples.

2012-08-14 | Copyright © 2004, 2012 Apple Inc. All Rights Reserved.

Figure 2-33 (page 98) shows the layout of the sample table atom.

Figure 2-33 The layout of a sample table atom

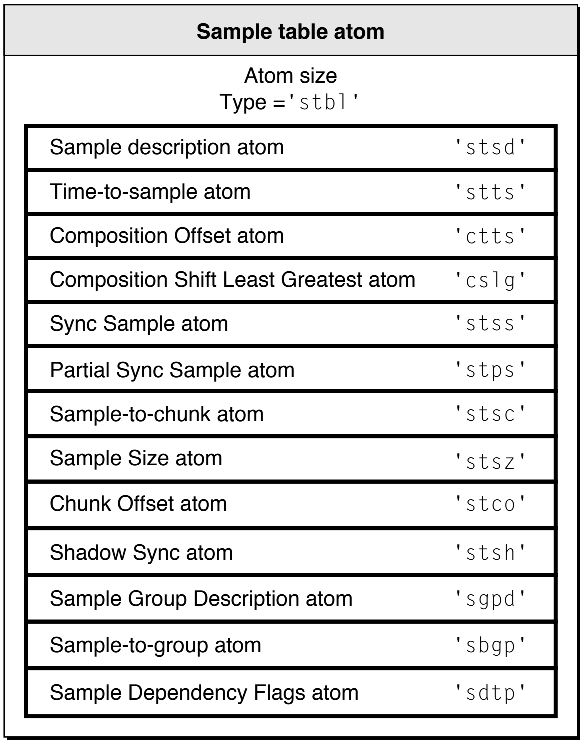

The sample table atom contains the following data elements.

Size
: A 32-bit integer that specifies the number of bytes in this sample table atom.

Type
: A 32-bit integer that identifies the atom type; this field must be set to `'stbl'`.

Sample description atom
: See “Sample Description Atoms” (page 99).

Time-to-sample atom
: See “Time-to-Sample Atoms” (page 101).

Composition offset atom
: See “ Composition Offset Atom” (page 103).

Composition Shift Least Greatest atom
: See “Composition Shift Least Greatest Atom” (page 105).

2012-08-14 | Copyright © 2004, 2012 Apple Inc. All Rights Reserved.

Sync sample atom
: See "Sync Sample Atoms" (page 107).

Partial sync sample atom
: See "Partial Sync Sample Atom" (page 108).

Sample-to-chunk atom
: See "Sample-to-Chunk Atoms" (page 109).

Sample size atom
: See "Sample Size Atoms" (page 111).

Chunk offset atom
: See "Chunk Offset Atoms" (page 113).

Sample Dependency Flags atom
: See "Sample Dependency Flags Atom" (page 115).

Shadow sync atom
: Reserved for future use.

Sample Description Atoms

The sample description atom stores information that allows you to decode samples in the media. The data stored in the sample description varies, depending on the media type. For example, in the case of video media, the sample descriptions are image description structures. The sample description information for each media type is explained in "Media Data Atom Types" (page 154)

Figure 2-34 (page 99) shows the layout of the sample description atom.

Figure 2-34 The layout of a sample description atom

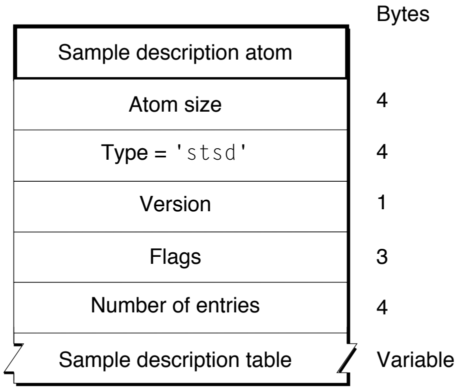

2012-08-14 | Copyright © 2004, 2012 Apple Inc. All Rights Reserved.

The sample description atom has an atom type of `'stsd'`. The sample description atom contains a table of sample descriptions. A media may have one or more sample descriptions, depending upon the number of different encoding schemes used in the media and on the number of files used to store the data. The sample-to-chunk atom identifies the sample description for each sample in the media by specifying the index into this table for the appropriate description (see "Sample-to-Chunk Atoms" (page 109)).

The sample description atom contains the following data elements.

Size
: A 32-bit integer that specifies the number of bytes in this sample description atom.

Type
: A 32-bit integer that identifies the atom type; this field must be set to `'stsd'`.

Version
: A 1-byte specification of the version of this sample description atom.

Flags
: A 3-byte space for sample description flags. Set this field to 0.

Number of entries
: A 32-bit integer containing the number of sample descriptions that follow.

Sample description table
: An array of sample descriptions. For details, see "General Structure of a Sample Description" (page 100).

General Structure of a Sample Description

While the exact format of the sample description varies by media type, the first four fields of every sample description are the same.

Sample description size
: A 32-bit integer indicating the number of bytes in the sample description.

> **Important:** When parsing sample descriptions in the `'stsd'` atom, be aware of the sample description size value in order to read each table entry correctly. Some sample descriptions terminate with four zero bytes that are not otherwise indicated.

Data format
: A 32-bit integer indicating the format of the stored data. This depends on the media type, but is usually either the compression format or the media type.

Reserved
: Six bytes that must be set to 0.

2012-08-14 | Copyright © 2004, 2012 Apple Inc. All Rights Reserved.

Data reference index

A 16-bit integer that contains the index of the data reference to use to retrieve data associated with samples that use this sample description. Data references are stored in data reference atoms.

These four fields may be followed by additional data specific to the media type and data format. See "Media Data Atom Types" (page 154) for additional details regarding specific media types and media formats.

Time-to-Sample Atoms

Time-to-sample atoms store duration information for a media's samples, providing a mapping from a time in a media to the corresponding data sample. The time-to-sample atom has an atom type of `'stts'`.

You can determine the appropriate sample for any time in a media by examining the time-to-sample atom table, which is contained in the time-to-sample atom.

The atom contains a compact version of a table that allows indexing from time to sample number. Other tables provide sample sizes and pointers from the sample number. Each entry in the table gives the number of consecutive samples with the same time delta, and the delta of those samples. By adding the deltas, a complete time-to-sample map can be built.

The atom contains time deltas: `DT(n+1) = DT(n) + STTS(n)` where `STTS(n)` is the (uncompressed) table entry for sample n and DT is the display time for sample (n). The sample entries are ordered by time stamps; therefore, the deltas are all nonnegative. The DT axis has a zero origin; `DT(i) = SUM` (for `j=0` to `i-1` of `delta(j)`), and the sum of all deltas gives the length of the media in the track (not mapped to the overall time scale, and not considering any edit list). The edit list atom provides the initial DT value if it is nonempty (nonzero).

Figure 2-35 (page 101) shows the layout of the time-to-sample atom.

Figure 2-35 The layout of a time-to-sample atom

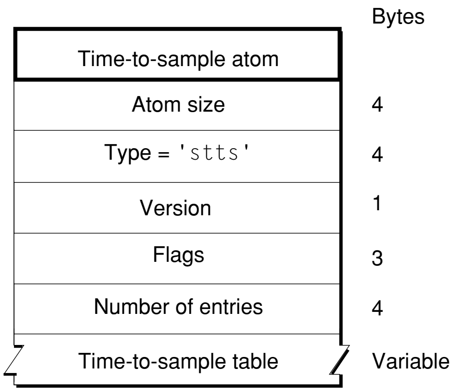

The time-to-sample atom contains the following data elements.

2012-08-14 | Copyright © 2004, 2012 Apple Inc. All Rights Reserved.

Size

A 32-bit integer that specifies the number of bytes in this time-to-sample atom.

Type

A 32-bit integer that identifies the atom type; this field must be set to `'stts'`.

Version

A 1-byte specification of the version of this time-to-sample atom.

Flags

A 3-byte space for time-to-sample flags. Set this field to 0.

Number of entries

A 32-bit integer containing the count of entries in the time-to-sample table.

Time-to-sample table

A table that defines the duration of each sample in the media. Each table entry contains a count field and a duration field. The structure of the time-to-sample table is shown in Figure 2-36 (page 102).

Figure 2-36 The layout of a time-to-sample table entry

Sample count	Sample duration	Field
4	4	Bytes

You define a time-to-sample table entry by specifying these fields:

Sample count

A 32-bit integer that specifies the number of consecutive samples that have the same duration.

Sample duration

A 32-bit integer that specifies the duration of each sample.

Entries in the table describe samples according to their order in the media and their duration. If consecutive samples have the same duration, a single table entry can be used to define more than one sample. In these cases, the count field indicates the number of consecutive samples that have the same duration. For example, if a video media has a constant frame rate, this table would have one entry and the count would be equal to the number of samples.

2012-08-14 | Copyright © 2004, 2012 Apple Inc. All Rights Reserved.

Figure 2-37 (page 103) presents an example of a time-to-sample table that is based on the chunked media data shown in Figure 2-32 (page 96). That data stream contains a total of nine samples that correspond in count and duration to the entries of the table shown here. Even though samples 4, 5, and 6 are in the same chunk, sample 4 has a duration of 3, and samples 5 and 6 have a duration of 2.

Figure 2-37 An example of a time-to-sample table

Sample count	Sample duration
4	3
2	1
3	2

Composition Offset Atom

Video samples in encoded formats have a decode order and a presentation order (also called *composition order* or *display order*). The composition offset atom is used when there are out-of-order video samples.

- If the decode and presentation orders are the same, no composition offset atom will be present. The time-to-sample atom provides both the decode and presentation ordering of the video stream, and allows calculation of the start and end times.
- If video samples are stored out of presentation order, the time-to-sample atom provides the decode order and the composition offset atom provides the time of presentation for the decoded samples expressed as a delta on a sample-by-sample basis.

Note: Decode time does not directly imply presentation time when working with out of order video samples. The ordering is significant.

The composition offset atom contains a sample-by-sample mapping of the decode-to-presentation time. Each entry in the composition offset table is a time delta from decode to presentation time: CT(n) = DT(n) + CTTS(n) where CTTS(n) is the (uncompressed) table entry for sample n DT is the decode time and CT is the composition (or display) time. The delta expressed in the composition offset table can be positive or negative.

When the time-to-sample atom and the composition offset atom are present, a reader parsing out-of-order video samples has all the information necessary to calculate the start and end times, as well as the minimum and maximum offsets between decode time and presentation time. The sample tables are scanned to obtain these values.

2012-08-14 | Copyright © 2004, 2012 Apple Inc. All Rights Reserved.

Note: At the last displayed frame, the decode duration is used as presentation duration.

The type of the composition offset atom is `'ctts'`.

Figure 2-38 (page 104) shows the layout of this atom.

Figure 2-38 The layout of a composition offset atom

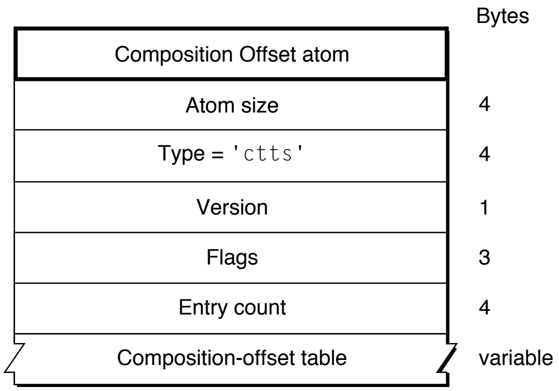

Size

A 32-bit integer that specifies the number of bytes in the composition offset atom.

Type

A 32-bit integer that identifies the atom type; this field must be set to `'ctts'`.

Version

A 1-byte specification of the version of this atom.

Flags

A 3-byte space reserved for offset flags. Set this field to 0.

2012-08-14 | Copyright © 2004, 2012 Apple Inc. All Rights Reserved.

Entry count

A 32-bit unsigned integer that specifies the number of sample numbers in the array that follows.

Following the entry count is a composition-offset table, shown in Figure 2-39 (page 105).

Figure 2-39 The layout of a composition-offset table entry

SampleCount	compositionOffset	Field
4	4	Bytes

sampleCount

A 32-bit unsigned integer that provides the number of consecutive samples with the calculated composition offset in the field.

compositionOffset

A 32-bit signed integer indicating the value of the calculated compositionOffset.

Composition Shift Least Greatest Atom

The optional composition shift least greatest atom summarizes the calculated minimum and maximum offsets between decode and composition time, as well as the start and end times, for all samples. This allows a reader to determine the minimum required time for decode to obtain proper presentation order without needing to scan the sample table for the range of offsets. The type of the composition shift least greatest atom is `'cslg'`.

Figure 2-40 (page 105) shows the layout of this atom.

Figure 2-40 The layout of a composition shift least greatest atom

Composition Shift Least Greatest atom	Bytes
Atom size	4
Type = `'cslg'`	4
Version	1
Flags	3
compositionOffsetToDisplayOffsetShift	4
leastDisplayOffset	4
displayStartTime	4
displayEndTime	4

Copyright © 2004, 2012 Apple Inc. All Rights Reserved.

Size

A 32-bit integer that specifies the number of bytes in the composition shift least greatest atom atom.

Type

A 32-bit integer that identifies the atom type; this field must be set to `'cslg'`.

Version

A 1-byte specification of the version of this atom.

Flags

A 3-byte space reserved for flags. Set this field to 0.

compositionOffsetToDisplayOffsetShift

A 32-bit unsigned integer that specifies the calculated value.

leastDisplayOffset

A 32-bit signed integer that specifies the calculated value.

greatestDisplayOffset

A 32-bit signed integer that specifies the calculated value.

displayStartTime

A 32-bit signed integer that specifies the calculated value.

displayEndTime

A 32-bit signed integer that specifies the calculated value.

Using Composition Offset and Composition Shift Least Greatest Atoms

When storing an out of order video stream's sample table, the offset shift should be calculated.

```
leastDisplayOffset = min { display offsets of all samples }
  greatestDisplayOffset = max { display offsets of all samples }
  if( leastDisplayOffset < 0 )
    compositionOffsetToDisplayOffsetShift = leastDisplayOffset;
  else
    compositionOffsetToDisplayOffsetShift = 0;
```

These values are stored in a composition shift least greatest atom within the sample table atom.

Then a composition offset table atom should be written that stores the display offsets, adjusting each offset by subtracting compositionOffsetToDisplayOffsetShift:

```
compositionOffset[n] = displayOffset[n] - compositionOffsetToDisplayOffsetShift;
```

2012-08-14 | Copyright © 2004, 2012 Apple Inc. All Rights Reserved.

> **Note:** If a composition shift least greatest atom is not present, a reader must assume `compositionOffsetToDisplayOffsetShift = 0`. The sample tables will need to be scanned to find the least and greatest offsets, as well as the presentation start and end times, to determine the decode time offset required for presentation.

Sync Sample Atoms

The sync sample atom identifies the key frames in the media. In a media that contains compressed data, key frames define starting points for portions of a temporally compressed sequence. The key frame is self-contained—that is, it is independent of preceding frames. Subsequent frames may depend on the key frame.

The sync sample atom provides a compact marking of the random access points within a stream. The table is arranged in strictly increasing order of sample number. If this table is not present, every sample is implicitly a random access point.

Sync sample atoms have an atom type of `'stss'`. The sync sample atom contains a table of sample numbers. Each entry in the table identifies a sample that is a key frame for the media. If no sync sample atom exists, then all the samples are key frames.

Figure 2-41 (page 107) shows the layout of a sync sample atom.

Figure 2-41 The layout of a sync sample atom

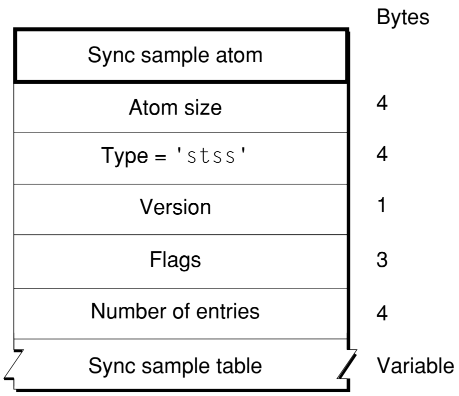

The sync sample atom contains the following data elements.

Size

A 32-bit integer that specifies the number of bytes in this sync sample atom.

Type

A 32-bit integer that identifies the atom type; this field must be set to `'stss'`.

2012-08-14 | Copyright © 2004, 2012 Apple Inc. All Rights Reserved.

Version

A 1-byte specification of the version of this sync sample atom.

Flags

A 3-byte space for sync sample flags. Set this field to 0.

Number of entries

A 32-bit integer containing the count of entries in the sync sample table.

Sync sample table

A table of sample numbers; each sample number corresponds to a key frame. Figure 2-42 (page 108) shows the layout of the sync sample table.

Figure 2-42 The layout of a sync sample table

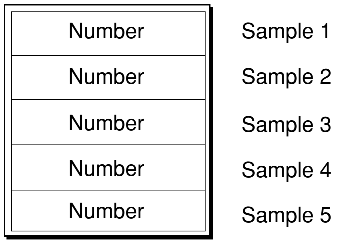

Partial Sync Sample Atom

This atom lists the partial sync samples. Since such samples are not full sync samples, they should not also be listed in the sync sample atom.

The type of the partial sync sample atom is `'stps'`.

Figure 2-43 (page 108) shows the layout of this atom.

Figure 2-43 The layout of a partial sync sample atom

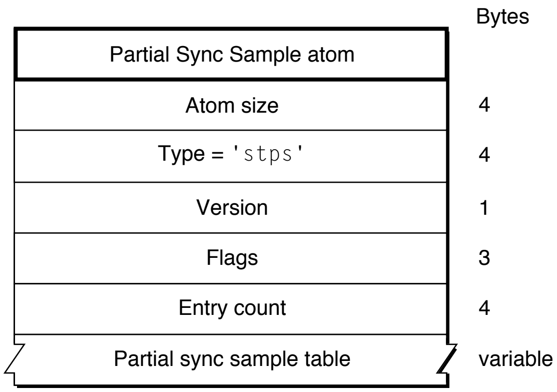

Copyright © 2004, 2012 Apple Inc. All Rights Reserved.

Size
: A 32-bit integer that specifies the number of bytes in the partial sync sample atom.

Type
: A 32-bit integer that identifies the atom type; this field must be set to `‘stps’`.

Version
: A 1-byte specification of the version of this atom.

Flags
: A 3-byte space reserved for flags. Set this field to 0.

Entry count
: A 32-bit unsigned integer that specifies the number of sample numbers in the array that follows.

Partial sync sample table
: A table of sample numbers. Figure 2-44 (page 109) shows the layout of the partial sync sample table.

Figure 2-44 The layout of a partial sync sample table

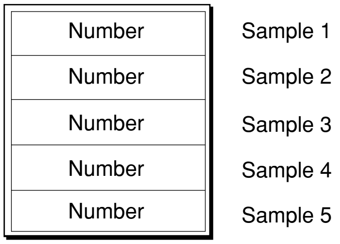

Sample-to-Chunk Atoms

As samples are added to a media, they are collected into chunks that allow optimized data access. A chunk contains one or more samples. Chunks in a media may have different sizes, and the samples within a chunk may have different sizes. The sample-to-chunk atom stores chunk information for the samples in a media.

Sample-to-chunk atoms have an atom type of `'stsc'`. The sample-to-chunk atom contains a table that maps samples to chunks in the media data stream. By examining the sample-to-chunk atom, you can determine the chunk that contains a specific sample.

2012-08-14 | Copyright © 2004, 2012 Apple Inc. All Rights Reserved.

Figure 2-45 (page 110) shows the layout of the sample-to-chunk atom.

Figure 2-45 The layout of a sample-to-chunk atom

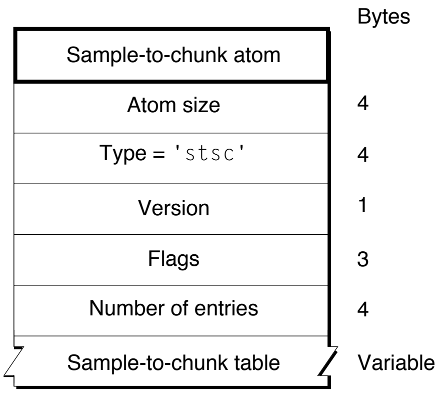

The sample-to-chunk atom contains the following data elements.

Size
: A 32-bit integer that specifies the number of bytes in this sample-to-chunk atom.

Type
: A 32-bit integer that identifies the atom type; this field must be set to `'stsc'`.

Version
: A 1-byte specification of the version of this sample-to-chunk atom.

Flags
: A 3-byte space for sample-to-chunk flags. Set this field to 0.

Number of entries
: A 32-bit integer containing the count of entries in the sample-to-chunk table.

Sample-to-chunk table
: A table that maps samples to chunks. Figure 2-46 (page 110) shows the structure of an entry in a sample-to-chunk table. Each sample-to-chunk atom contains such a table, which identifies the chunk for each sample in a media. Each entry in the table contains a first chunk field, a samples per chunk field, and a sample description ID field. From this information, you can ascertain where samples reside in the media data.

Figure 2-46 The layout of a sample-to-chunk table entry

First chunk	Samples per chunk	Sample description ID	Fields
4	4	4	Bytes

Copyright © 2004, 2012 Apple Inc. All Rights Reserved.

You define a sample-to-chunk table entry by specifying the following data elements.

First chunk
: The first chunk number using this table entry.

Samples per chunk
: The number of samples in each chunk.

Sample description ID
: The identification number associated with the sample description for the sample. For details on sample description atoms, see “Sample Description Atoms” (page 99).

Figure 2-47 (page 111) shows an example of a sample-to-chunk table that is based on the data stream shown in Figure 2-32 (page 96).

Figure 2-47 An example of a sample-to-chunk table

First chunk	Samples per chunk	Sample description ID
1	3	23
3	1	23
5	1	24

Each table entry corresponds to a set of consecutive chunks, each of which contains the same number of samples. Furthermore, each of the samples in these chunks must use the same sample description. Whenever the number of samples per chunk or the sample description changes, you must create a new table entry. If all the chunks have the same number of samples per chunk and use the same sample description, this table has one entry.

Sample Size Atoms

You use sample size atoms to specify the size of each sample in the media. Sample size atoms have an atom type of `'stsz'`.

The sample size atom contains the sample count and a table giving the size of each sample. This allows the media data itself to be unframed. The total number of samples in the media is always indicated in the sample count. If the default size is indicated, then no table follows.

2012-08-14 | Copyright © 2004, 2012 Apple Inc. All Rights Reserved.

Figure 2-48 (page 112) shows the layout of the sample size atom.

Figure 2-48 The layout of a sample size atom

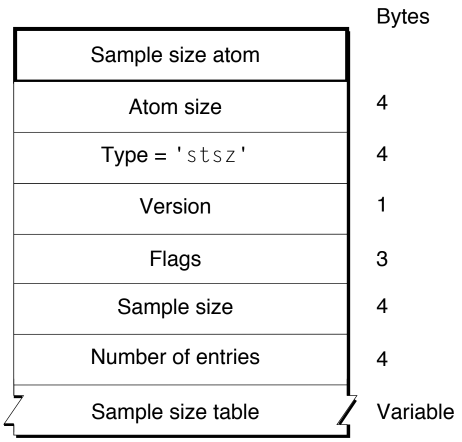

The sample size atom contains the following data elements.

Size
: A 32-bit integer that specifies the number of bytes in this sample size atom.

Type
: A 32-bit integer that identifies the atom type; this field must be set to `'stsz'`.

Version
: A 1-byte specification of the version of this sample size atom.

Flags
: A 3-byte space for sample size flags. Set this field to 0.

Sample size
: A 32-bit integer specifying the sample size. If all the samples are the same size, this field contains that size value. If this field is set to 0, then the samples have different sizes, and those sizes are stored in the sample size table.

Number of entries
: A 32-bit integer containing the count of entries in the sample size table.

Sample size table
: A table containing the sample size information. The sample size table contains an entry for every sample in the media's data stream. Each table entry contains a size field. The size field contains the size, in bytes, of the sample in question. The table is indexed by sample number—the first entry corresponds to the first sample, the second entry is for the second sample, and so on.

Copyright © 2004, 2012 Apple Inc. All Rights Reserved.

Figure 2-49 (page 113) shows the layout of an arbitrary sample size table.

Figure 2-49 An example of a sample size table

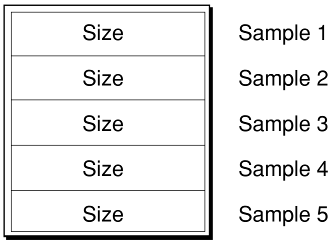

Chunk Offset Atoms

Chunk offset atoms identify the location of each chunk of data in the media's data stream. Chunk offset atoms have an atom type of `'stco'`.

The chunk-offset table gives the index of each chunk into the containing file. There are two variants, permitting the use of 32-bit or 64-bit offsets. The latter is useful when managing very large movies. Only one of these variants occurs in any single instance of a sample table atom.

Note that offsets are file offsets, not the offset into any atom within the file (for example, a `'mdat'` atom). This permits referring to media data in files without any atom structure. However, be careful when constructing a self-contained QuickTime file with its metadata (movie atom) at the front because the size of the movie atom affects the chunk offsets to the media data.

Copyright © 2004, 2012 Apple Inc. All Rights Reserved.

Note: The sample table atom can contain a 64-bit chunk offset atom (`STChunkOffset64AID = 'co64'`). When this atom appears, it is used in place of the original chunk offset atom, which can contain only 32-bit offsets. When QuickTime writes movie files, it uses the 64-bit chunk offset atom only if there are chunks that use the high 32-bits of the chunk offset. Otherwise, the original 32-bit chunk offset atom is used to ensure compatibility with previous versions of QuickTime.

Figure 2-50 (page 114) shows the layout of a chunk offset atom.

Figure 2-50 The layout of a chunk offset atom

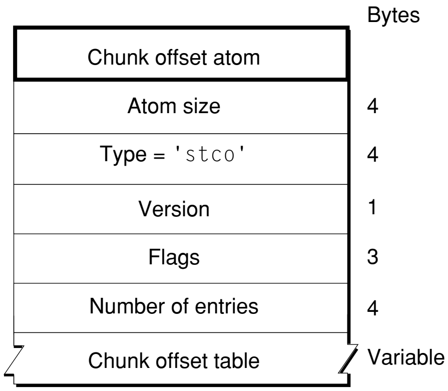

The chunk offset atom contains the following data elements.

Size
: A 32-bit integer that specifies the number of bytes in this chunk offset atom.

Type
: A 32-bit integer that identifies the atom type; this field must be set to `'stco'`.

Version
: A 1-byte specification of the version of this chunk offset atom.

Flags
: A 3-byte space for chunk offset flags. Set this field to 0.

Number of entries
: A 32-bit integer containing the count of entries in the chunk offset table.

Chunk offset table
: A chunk offset table consisting of an array of offset values. There is one table entry for each chunk in the media. The offset contains the byte offset from the beginning of the data stream to the chunk. The table is indexed by chunk number—the first table entry corresponds to the first chunk, the second table entry is for the second chunk, and so on.

2012-08-14 | Copyright © 2004, 2012 Apple Inc. All Rights Reserved.

Figure 2-51 (page 115) shows the layout of an arbitrary chunk offset table.

Figure 2-51 An example of a chunk offset table

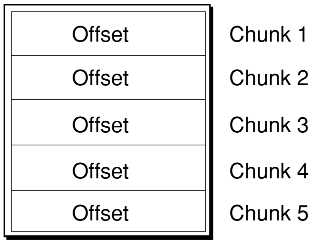

Sample Dependency Flags Atom

The sample dependency flags atom uses one byte per sample as a bit field that describes dependency information. The sample dependency flags atom has a type of `'sdtp'`.

Figure 2-52 (page 115) shows the layout of this atom.

Figure 2-52 The layout of a sample dependency flags atom

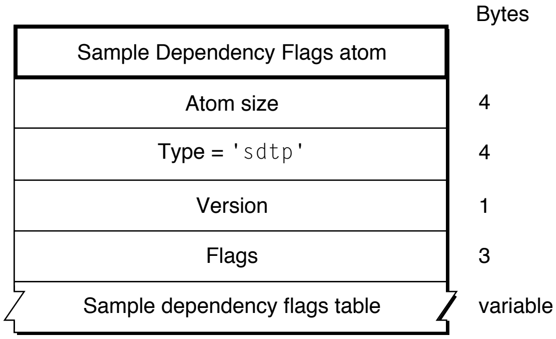

Size

A 32-bit integer that specifies the number of bytes in the sample dependency flags atom.

Type

A 32-bit integer that identifies the atom type; this field must be set to `‘sdtp’`.

Version

A 1-byte specification of the version of this atom.

Flags

A 3-byte space reserved for flags. Set this field to 0.

2012-08-14 | Copyright © 2004, 2012 Apple Inc. All Rights Reserved.

Sample dependency flags table

A table of 8-bit values indicating the sample flag settings. The number of entries in the table is obtained from the associated sample size atom's number of samples field. Figure 2-53 (page 116) shows the layout of an arbitrary sample dependency flags table.

Figure 2-53 An example of a sample dependency flags table

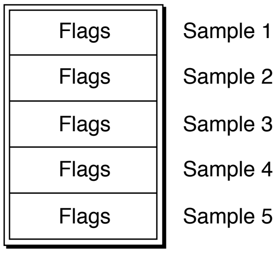

Flag values are specified as follows:

```
enum {
// bit 0x80 is reserved; bit combinations 0x30, 0xC0 and 0x03 are reserved
kQTSampleDependency_EarlierDisplayTimesAllowed = 1<<6, //
mediaSampleEarlierDisplayTimesAllowed
kQTSampleDependency_SampleDoesNotDependOnOthers = 1<<5, // ie: an I picture
kQTSampleDependency_SampleDependsOnOthers = 1<<4, // ie: not an I picture
kQTSampleDependency_NoOtherSampleDependsOnThisSample = 1<<3,  //
mediaSampleDroppable
kQTSampleDependency_OtherSamplesDependOnThisSample = 1<<2,
kQTSampleDependency_ThereIsNoRedundantCodingInThisSample = 1<<1,
kQTSampleDependency_ThereIsRedundantCodingInThisSample = 1<<0
};
```

Using Sample Atoms

This section presents examples using the atoms just described. These examples are intended to help you understand the relationships between these atoms.

The first section, "Finding a Sample" (page 117), describes the steps that the video media handler uses to find the sample that contains the media data for a particular time in a media. The second section, "Finding a Key Frame" (page 117), describes the steps that the video media handler uses to find an appropriate key frame for a specific time in a movie.

2012-08-14 | Copyright © 2004, 2012 Apple Inc. All Rights Reserved.

Finding a Sample

When QuickTime displays a movie or track, it directs the appropriate media handler to access the media data for a particular time. The media handler must correctly interpret the data stream to retrieve the requested data. In the case of video media, the media handler traverses several atoms to find the location and size of a sample for a given media time.

The media handler performs the following steps:

1. Determines the time in the media time coordinate system.
2. Examines the time-to-sample atom to determine the sample number that contains the data for the specified time.
3. Scans the sample-to-chunk atom to discover which chunk contains the sample in question.
4. Extracts the offset to the chunk from the chunk offset atom.
5. Finds the offset within the chunk and the sample's size by using the sample size atom.

Finding a Key Frame

Finding a key frame for a specified time in a movie is slightly more complicated than finding a sample for a specified time. The media handler must use the sync sample atom and the time-to-sample atom together in order to find a key frame.

The media handler performs the following steps:

1. Examines the time-to-sample atom to determine the sample number that contains the data for the specified time.
2. Scans the sync sample atom to find the key frame that precedes the sample number chosen in step 1.
3. Scans the sample-to-chunk atom to discover which chunk contains the key frame.
4. Extracts the offset to the chunk from the chunk offset atom.
5. Finds the offset within the chunk and the sample's size by using the sample size atom.

Compressed Movie Resources

Most QuickTime movies have metadata in addition to their media data. Media data can be compressed using a variety of video and sound compression algorithms. Beginning with QuickTime 3, it also became possible to compress the metadata—more commonly known as the movie resource. However, the movie resource cannot be compressed by means of a lossy compression algorithm because it contains critical information, such as the video and audio compression types used, individual frame offsets, and timing information. To compress the movie resource, therefore, lossless data compression algorithms must be used.

2012-08-14 | Copyright © 2004, 2012 Apple Inc. All Rights Reserved.

Compressing movie resources using data compression typically reduces the size of the movie resource by 50% or more. For QuickTime movies that are streamed over the Internet, this can substantially reduce the startup latency of the movie, and therefore has a number of distinct advantages.

Allowing QuickTime to Compress the Movie Resource

Most application developers won't need to know the details of how movie resources are compressed. The Movie Toolbox `FlattenMovie` and `FlattenMovieData` functions compress the movie resource if so requested by the application. To accomplish this, applications only need to set the `flattenCompressMovieResource` flag when calling either function. The QuickTime movie export component also provides users with the option of compressing the movie resource when exporting or creating a new movie through export.

Structure of a Compressed Movie Resource

A compressed movie resource, similar to an uncompressed movie resource, is made up of a group of QuickTime atoms arranged in a hierarchy.

Like an uncompressed movie resource, the outermost atom is a movie atom. Within the movie atom, there is a single compressed movie atom, which contains all other required atoms. The compressed movie atom has two sub atoms. The first is a data compression atom, which contains a single 32-bit integer that identifies what lossless data compression algorithm was used to compress the movie resource. The second child atom is the compressed movie data, which contains the compressed movie resource itself. The first 32-bit integer in the compressed movie data atom indicates the uncompressed size of the movie resource, and then the compressed movie resource data follows.

The contents of a complete compressed movie are shown in Table 2-5 (page 118). The constants that define the atom types are defined in `MoviesFormat.h`. The four-character codes for each atom type are also shown.

Table 2-5 Contents of complete compressed movie

Atom type	Four-character code
Movie	`'moov'`
Compressed movie	`'cmov'`
Data compression atom	`'dcom'`
Compressed movie data	`'cmvd'`
32-bit integer	Uncompressed size

Copyright © 2004, 2012 Apple Inc. All Rights Reserved.

Reference Movies

A QuickTime movie can act as a container for a set of alternate movies that should be displayed under specified conditions. One of these movies may be contained within the same file; any others are included by reference.

For example, a QuickTime movie can contain a list of references to movies having different data rates, allowing an application to choose the best-looking movie that can play smoothly as it downloads over the Internet, based on the user's connection speed.

A movie that contains references to alternate movies is called a reference movie.

A reference movie contains a reference movie atom (`'rmra'`) at the top level of the movie atom as shown in Figure 2-54 (page 119). The movie atom may also contain a movie header atom, or it may contain the reference movie atom alone.

Figure 2-54 A movie atom containing a `'rmra'` atom instead of a `'mvhd'` atom

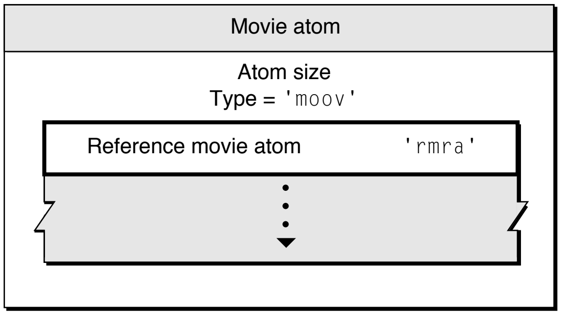

The reference movie atom contains one or more reference movie descriptor atoms, each of which describes an alternate movie.

Each reference movie descriptor atom contains a data reference atom, which specifies the location of a movie.

Note: Movie locations are specified using QuickTime data references. QuickTime supports multiple types of data reference, but alternate movies are generally specified using data reference types of either url (`'url '`) or file alias (`'alis'`).

A reference movie descriptor atom may contain other atoms that specify the movie's system requirements and the movie quality. If so, there will be an atom of an appropriate type for each requirement that must be met for the movie to play, and there may be a quality atom as well.

2012-08-14 | Copyright © 2004, 2012 Apple Inc. All Rights Reserved.

Applications should play the highest-quality movie whose requirements are met by the user's system. If the data reference to the selected movie cannot be resolved—because the file cannot be found, for example—the application should recursively attempt to play the next-highest-quality movie until it succeeds or has exhausted the list of movies whose requirements are met.

If a movie contains both a reference movie atom and a movie header atom, applications should play the appropriate movie indicated by the reference movie atom.

If the user's system does not meet any of the alternate movies' criteria, or none of the qualifying data references can be resolved, applications should play the movie defined in the movie header atom. (The movie defined in the movie header atom can also be indicated by one of the alternate movie references.)

The movie header atom is sometimes used to provide a fallback movie for applications that can play older QuickTime movies but do not understand reference movies.

When parsing a reference movie, the reader should treat the URL or file reference in the reference movie atom as a new starting point, making no assumptions that the reference is a valid URL, or an existing file, or a well-formed and playable QuickTime movie.

Reference Movie Atom

A reference movie atom contains references to one or more movies as shown in Figure 2-55 (page 120). It can optionally contain a list of system requirements in order for each movie to play, and a quality rating for each movie. It is typically used to specify a list of alternate movies to be played under different conditions.

A reference movie atom's parent is always a movie atom (`'moov'`). Only one reference movie atom is allowed in a given movie atom.

Figure 2-55 A `'rmra'` atom with multiple `'rmda'` atoms

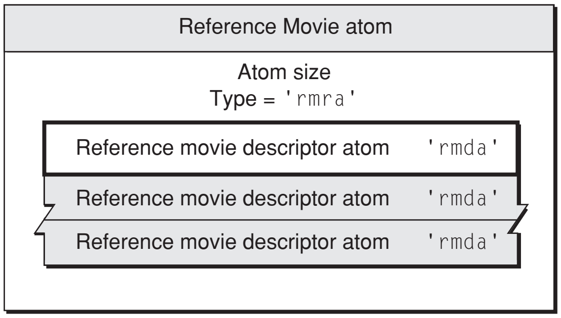

A reference movie atom may contain the following fields:

2012-08-14 | Copyright © 2004, 2012 Apple Inc. All Rights Reserved.

Size

The number of bytes in this reference movie atom.

Type

The type of this atom; this field must be set to 'rmra'.

Reference movie descriptor atom

A reference movie atom must contain at least one reference movie descriptor atom, and typically contains more than one. See "Reference Movie Descriptor Atom" (page 121) for more information.

Reference Movie Descriptor Atom

Each reference movie descriptor atom contains other atoms that describe where a particular movie can be found, and optionally what the system requirements are to play that movie, as well as an optional quality rating for that movie.

A reference movie descriptor atom's parent is always a movie reference atom ('rmra'). Multiple reference movie descriptor atoms are allowed in a given movie reference atom, and more than one is usually present.

Figure 2-56 (page 121) shows the layout of this atom.

Figure 2-56 Reference movie descriptor atom

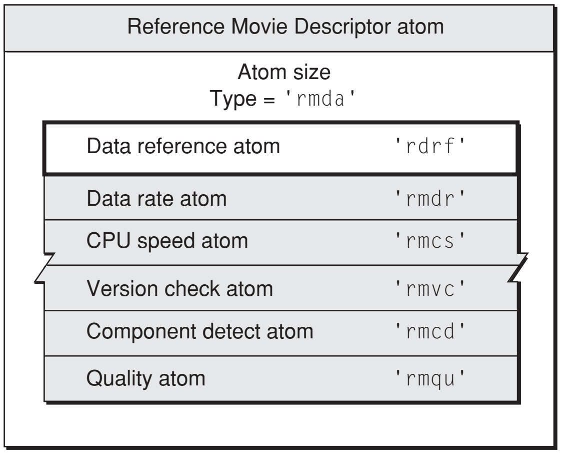

A reference movie descriptor atom may contain the following fields:

Size

The number of bytes in this reference movie descriptor atom.

Type

The type of this atom; this field must be set to 'rmda'.

Copyright © 2004, 2012 Apple Inc. All Rights Reserved.

Data reference atom

Each reference movie atom must contain exactly one data reference atom. See "Data Reference Atoms" (page 94) for more information.

Data rate atom

A reference movie atom may contain an optional data rate atom. Only one data rate atom can be present. See "Data Rate Atom" (page 123) for more information.

CPU speed atom

A reference movie atom may contain an optional CPU speed atom. Only one CPU speed atom can be present. See "CPU Speed Atom" (page 124) for more information.

Version check atom

A reference movie atom may contain an optional version check atom. Multiple version check atoms can be present. See "Version Check Atom" (page 124) for more information.

Component detect atom

A reference movie atom may contain an optional component detect atom. Multiple component detect atoms can be present. See "Component Detect Atom" (page 125) for more information.

Quality atom

A reference movie atom may contain an optional quality atom. Only one quality atom can be present. See "Quality Atom" (page 127) for more information.

Data Reference Atom

A data reference atom contains the information necessary to locate a movie, or a stream or file that QuickTime can play, typically in the form of a URL or a file alias.

Only one data reference atom is allowed in a given movie reference descriptor atom.

A data reference atom may contain the following fields:

Size

The number of bytes in this data reference atom.

Type

The type of this atom; this field must be set to `'rdrf'`.

Flags

A 32-bit integer containing flags. One flag is currently defined.

Movie is self-contained

If the least-significant bit is set to 1, the movie is self-contained. This requires that the parent movie contain a movie header atom as well as a reference movie atom. In other words, the current

Copyright © 2004, 2012 Apple Inc. All Rights Reserved.

'moov' atom must contain both a 'rmra' atom and a 'mvhd' atom. To resolve this data reference, an application uses the movie defined in the movie header atom, ignoring the remainder of the fields in this data reference atom, which are used only to specify external movies.

Data reference type
: The data reference type. A value of 'alis' indicates a file system alias record. A value of 'url ' indicates a string containing a uniform resource locator. Note that the fourth character in 'url ' is an ASCII blank (0x20).

Data reference size
: The size of the data reference in bytes, expressed as a 32-bit integer.

Data reference
: A data reference to a QuickTime movie, or to a stream or file that QuickTime can play. If the reference type is 'alis' this field contains the contents of an AliasHandle. If the reference type is 'url ' this field contains a NULL-terminated string that can be interpreted as a URL. The URL can be absolute or relative, and can specify any protocol that QuickTime supports, including http://, ftp://, rtsp://, file:///, and data:.

Data Rate Atom

A data rate atom specifies the minimum data rate required to play a movie. This is normally compared to the connection speed setting in the user's QuickTime Settings control panel. Applications should play the movie with the highest data rate less than or equal to the user's connection speed. If the connection speed is slower than any movie's data rate, applications should play the movie with the lowest data rate. The movie with the highest data rate is assumed to have the highest quality.

Only one data rate atom is allowed in a given reference movie descriptor atom.

A data rate atom may contain the following fields:

Size
: The number of bytes in this data rate atom.

Type
: The type of this atom; this field must be set to 'rmdr'.

Flags
: A 32-bit integer that is currently always 0.

Data rate
: The required data rate in bits per second, expressed as a 32-bit integer.

2012-08-14 | Copyright © 2004, 2012 Apple Inc. All Rights Reserved.

CPU Speed Atom

A CPU speed atom specifies the minimum computing power needed to display a movie. QuickTime performs an internal test to determine the speed of the user's computer.

This is not a simple measurement of clock speed—it is a measurement of performance for QuickTime-related operations. Speed is expressed as a relative value between 100 and 2^31, in multiples of 100.

> **Note:** Typical scores might range from a minimum score of 100, which would describe a computer as slow as, or slower than, a 166 MHz Pentium or 120 MHz PowerPC, to a maximum score of 600 for a 500 MHz Pentium III or 400 MHz G4 PowerPC. A computer with a graphics accelerator and a Gigahertz clock speed might score as high as 1000. Future computers will score higher.

Applications should play the movie with the highest specified CPU speed that is less than or equal to the user's speed. If the user's speed is lower than any movie's CPU speed, applications should play the movie with the lowest CPU speed requirement. The movie with the highest CPU speed is assumed to be the highest quality.

Only one CPU speed atom is allowed in a given reference movie descriptor atom.

A CPU speed atom may contain the following fields:

Size
: The number of bytes in this CPU speed atom.

Type
: The type of this atom; this field must be set to `'rmcs'`.

Flags
: A 32-bit integer that is currently always 0.

CPU speed
: A relative ranking of required computer speed, expressed as a 32-bit integer divisible by 100, with larger numbers indicating higher speed.

Version Check Atom

A version check atom specifies a software package, such as QuickTime or QuickTime VR, and the version of that package needed to display a movie. The package is specified using a Macintosh Gestalt type, such a `'qtim'` for QuickTime (QuickTime provides support for these Gestalt tests in the Windows computing environment).

You can specify a minimum required version to be returned by the Gestalt check, or you can require that a specific value be returned after performing a binary `AND` operation on the Gestalt bitfield and a mask.

2012-08-14 | Copyright © 2004, 2012 Apple Inc. All Rights Reserved.

Multiple version check atoms are allowed within a given reference movie descriptor atom. Applications should not attempt to play a movie unless all version checks are successful.

A version check atom may contain the following fields:

Size
: The number of bytes in this version check atom.

Type
: The type of this atom; this field must be set to `'rmvc'`.

Flags
: A 32-bit integer that is currently always 0.

Software package
: A 32-bit Gestalt type, such as `'qtim'`, specifying the software package to check for.

Version
: An unsigned 32-bit integer containing either the minimum required version or the required value after a binary `AND` operation.

Mask
: The mask for a binary `AND` operation on the Gestalt bitfield.

Check type
: The type of check to perform, expressed as 16-bit integer. Set to 0 for a minimum version check, set to 1 for a required value after a binary `AND` of the Gestalt bitfield and the mask.

Component Detect Atom

A component detect atom specifies a QuickTime component, such as a particular video decompressor, required to play the movie. The component type, subtype, and other required attributes can be specified, as well as a minimum version.

Multiple component detect atoms are allowed within a given reference movie descriptor atom. Applications should not attempt to play a movie unless at least the minimum versions of all required components are present.

A component detect atom may contain the following fields:

Size
: The number of bytes in this component detect atom.

Type
: The type of this atom; this field must be set to `'rmcd'`.

Copyright © 2004, 2012 Apple Inc. All Rights Reserved.

Flags

A 32-bit integer that is currently always 0.

Component description

A component description record. For details, see "Component Description Record" (page 126).

Minimum version

An unsigned 32-bit integer containing the minimum required version of the specified component.

Component Description Record

Describes a class of components by their attributes. Fields that are set to 0 are treated as "don't care."

```
struct ComponentDescription {
OSType          componentType;
OSType          componentSubType;
OSType          componentManufacturer;
unsigned long   componentFlags;
unsigned long   componentFlagsMask;
};
```

`componentType`

A four-character code that identifies the type of component.

`componentSubType`

A four-character code that identifies the subtype of the component. For example, the subtype of an image compressor component indicates the compression algorithm employed by the compressor. A value of 0 matches any subtype.

`componentManufacturer`

A four-character code that identifies the manufacturer of the component. Components provided by Apple have a manufacturer value of `'appl'`. A value of 0 matches any manufacturer.

`componentFlags`

A 32-bit field that contains flags describing required component capabilities. The high-order 8 bits should be set to 0. The low-order 24 bits are specific to each component type. These flags can be used to indicate the presence of features or capabilities in a given component.

`componentFlagsMask`

A 32-bit field that indicates which flags in the `componentFlags` field are relevant to this operation. For each flag in the `componentFlags` field that is to be considered as a search criterion, set the corresponding bit in this field to 1. To ignore a flag, set the bit to 0.

2012-08-14 | Copyright © 2004, 2012 Apple Inc. All Rights Reserved.

Constants

`canMovieImportInPlace`
Set this bit if a movie import component must be able to create a movie from a file without having to write to a separate disk file. Examples include MPEG and AIFF import components.

`movieImportSubTypeIsFileExtension`
Set this bit if the component's subtype is a file extension instead of a Macintosh file type. For example, if you require an import component that opens files with an extension of `.doc`, set this flag and set your component subtype to `'DOC '`.

`canMovieImportFiles`
Set this bit if a movie import component must import files.

Quality Atom

A quality atom describes the relative quality of a movie. This acts as a tiebreaker if more than one movie meets the specified requirements, and it is not otherwise obvious which movie should be played.

This would be the case if two qualified movies have the same data rate and CPU speed requirements, for example, or if one movie requires a higher data rate and another requires a higher CPU speed, but both can be played on the current system. In these cases, applications should play the movie with the highest quality, as specified in the quality atom.

Only one quality atom is allowed in a given reference movie descriptor atom.

A quality atom may contain the following fields:

Size
The number of bytes in this quality atom.

Type
The type of this atom; this field must be set to `'rmqu'`.

Quality
The relative quality of the movie, expressed as a 32-bit integer. A larger number indicates higher quality. A unique value should be given to each movie.

2012-08-14 | Copyright © 2004, 2012 Apple Inc. All Rights Reserved.

Metadata

This chapter describes how to store metadata in QuickTime Movie files. It also defines keys for some common metadata types as examples of how to employ the metadata capabilities in the QuickTime file format.

Overview

Metadata can be defined as useful information related to media. This section describes a method of associating metadata with media in a QuickTime file that is extensible and allows for language and country tagging. In addition, it provides a means to store the type of the metadata and associate a name with metadata. This method of storing metadata is supported in both QuickTime 7 and QuickTime X.

This metadata format uses a key–value pair for each type of metadata being stored. Standard keys, with specific formats for the values they indicate, have been defined. See "QuickTime Metadata Keys" (page 144) for details.

> **Note:** The QuickTime file format also defines user data which, in some limited cases, can be used to store metadata. The method of storing metadata defined in this section provides an extensible and flexible design for handling a wide variety of metadata types.

Data Type

The storage type of metadata items is defined via an enumerated list of data types, defined statically; an example might be "plain Unicode text". See the "Well-Known Types" (page 143) table for details of the standard, defined data types.

Meaning or Purpose

The meaning of a metadata item identifies what it represents: a copyright notice, the performer's name, and so on. It uses an extensible namespace allowing for meanings or keys to be added, and then referenced, from metadata items. These keys may be four-character codes, names in reverse-address format (such as "com.apple.quicktime.windowlocation") or any other key format including native formats from external metadata standards. A key is tagged with its namespace allowing for extension in the future. It is recommended that reverse-address format be used in the general case: this provides an extensible syntax for vendor data or for other organizations or standards bodies.

2012-08-14 | Copyright © 2004, 2012 Apple Inc. All Rights Reserved.

Data Location

Metadata is stored immediately in the corresponding atom structures, by value.

Localization

A metadata item can be identified as specific to a country or set of countries, to a language or set of languages, or to some combination of languages and countries. This identification allows for a default value (suitable for any language or country not explicitly called out), a single value, or a list of values.

Storage Location in a QuickTime File

Within a QuickTime file, metadata can be stored within a movie atom (`'moov'`), a track atom (`'trak'`) or a media atom (`'mdia'`). Only one metadata atom is allowed for each location. If there is user data and metadata stored in the same location, and both declare the same information, for example, declare a copyright notice, the metadata takes precedence.

Metadata Structure

The container for metadata is an atom of type `'meta'`. The metadata atom must contain the following subatoms: metadata handler atom (`'hdlr'`), metadata item keys atom (`'keys'`), and metadata item list atom (`'ilst'`). Other optional atoms that may be contained in a metadata atom include the country list atom (`'ctry'`), language list atom (`'lang'`) and free space atom (`'free'`). The country list and language list atoms can be used to store localized data in an efficient manner. The free space atom may be used to reserve space in a metadata atom for later additions to it, or to zero out bytes within a metadata atom after editing and removing elements from it. The free space atom may not occur within any other subatom contained in the metadata atom.

Metadata Atom

The metadata atom is the container for carrying metadata.

2012-08-14 | Copyright © 2004, 2012 Apple Inc. All Rights Reserved.

Figure 3-1 (page 130) shows a sample layout for this atom.

Figure 3-1 Sample of a metadata atom and subatoms

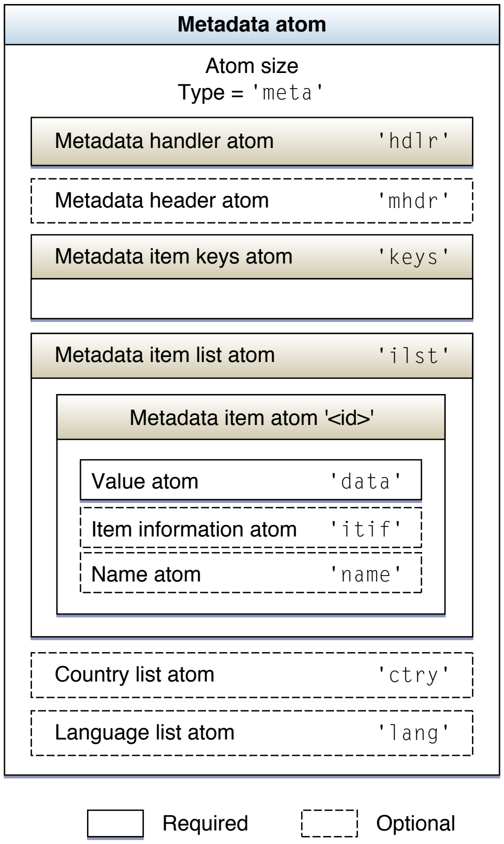

Metadata Handler Atom

The metadata handler atom is a full atom with an atom type of `'hdlr'`. It defines the structure used for all types of metadata stored within the metadata atom.

The layout of the metadata handler atom is defined:

Size

A 32-bit unsigned integer that indicates the size in bytes of the atom structure

Type

A 32-bit unsigned integer value set to `'hdlr'`

Version

One byte that is set to 0

Flags

Three bytes that are set to 0

Copyright © 2004, 2012 Apple Inc. All Rights Reserved.

Predefined
: A 32-bit integer that is set to 0

Handler type
: A 32-bit integer that indicates the structure used in the metadata atom, set to `'mdta'`

Reserved
: An array of 3 const unsigned 32-bit integers set to 0

Name
: The name is a NULL-terminated string in UTF-8 characters which gives a human-readable name for a metadata type, for debugging and inspection purposes. The string may be empty or a single byte containing 0.

Note: A reader parsing a metadata atom should confirm the handler type in the metadata handler atom is `'mdta'` before interpreting any other structures in the metadata atom according to the specification presented here. If the handler type is not `'mdta'`, the interpretation is defined by another specification.

Metadata Header Atom

The metadata format optionally assigns unique identifiers to metadata items for such purposes as defining stable identifiers for external references into the set of metadata items. This is accomplished by including an item information atom in added metadata item atoms contained by the metadata item list atom. Such unique identifiers must be guaranteed to be unique.

To make the assignment of unique item identifiers more efficient, the metadata atom may contain a metadata header atom holding the integer value for the next unique item identifier to assign stored in the nextItemID field. In general it holds a value one greater than the largest identifier used so far.

Important: The metadata header atom must exist if there are metadata item atoms containing an item information atom indicating the item's unique ID.

Upon assigning the identifier to a metadata item, if the value of the nextItemID field is less than 0xFFFFFFFF, it should be incremented to the next unused value. If the value of nextItemID is equal to 0xFFFFFFFF, it should not be changed: in that case, a search for an unused item identifier value in the range from 0 to 0xFFFFFFFF is needed for all additions.

The metadata header atom is a full atom with an atom type of `'mhdr'`. It contains the following fields:

Size
: A 32-bit unsigned integer that indicates the size in bytes of the atom structure

2012-08-14 | Copyright © 2004, 2012 Apple Inc. All Rights Reserved.

Type
: A 32-bit unsigned integer value set to 'mdhr'

Version
: One byte that is set to 0.

Flags
: Three bytes that are set to 0.

nextItemID
: A 32-bit unsigned integer indicating the value to use for the item ID of the next item created or assigned an item ID. If the value is all ones, it indicates that future additions will require a search for an unused item ID.

> **Note:** If the last metadata item with an item information atom is removed and value of nextItemID is 0xFFFFFFFF, an implementation may reset the metadata header atom's nextItemID value to 0 so that new assignments are again efficient (that is, they do not require a search for unused identifiers).

Extensibility

In order to allow metadata to be rewritten easily and without the need to rewrite the entire QuickTime movie file, free space atoms may occur anywhere in the definition of the metadata atom between the positions of other atoms contained by the metadata atom. Free space atoms may not be inserted between items in the metadata item list atom or within atoms in the metadata item list atom. This restriction on free space atom definition avoids the risk of confusing a free space atom with a meaning of a 'free' identifier or a value atom of type 'free' defined in the context of the metadata atom structure.

Similarly, UUID atoms for specific extensions may be placed in any position where a succession of atoms is permitted. Note that UUID atoms should not be created for atoms already defined using four-character codes.

Unrecognized atoms (that is, those atoms whose types not defined in the context of the metadata atom structure and are contained within the metadata item list atom) are ignored.

Localization List Sets

When metadata items have individual values associated with more than one country or more than one language, a country list atom and/or language list atom are required. Alternatively, if all values are associated with zero or one country, no country list atom is required, and if all values are associated with zero or one language, no language list atom is required.

Copyright © 2004, 2012 Apple Inc. All Rights Reserved.

Country List Atom

When one or more items must be identified as being suitable for more than one country, each list of countries is stored in this otherwise optional atom. The country list atom is a full atom with an atom type of `'ctry'`.

Each list starts with a two-byte count of the number of items in the list, and then each ISO 3166 code representing countries in the list.

The atom consists of a count of the number of lists, expressed as a 32-bit integer, and then these lists, appended end-to-end. The country list atom contains the following fields:

Size
: A 32-bit unsigned integer that indicates the size in bytes of the atom structure

Type
: A 32-bit unsigned integer value set to `'ctry'`

Version
: One byte that is set to 0.

Flags
: Three bytes that are set to 0.

Entry_count
: A 32-bit integer indicating the number of Country arrays to follow in this atom.

Country_count
: A 16-bit integer indicating the number of Countries in the array.

Country[Country_count]
: An array of 16-bit integers, defined according to the ISO 3166 definition of country codes.

Note that:

- Indexes into the country list atom are 1-based.
- Zero (0) is reserved and never used as an index.
- Currently, there is a limit of 255 countries that may be recorded in a country list atom.

An example country list atom consisting of two country lists with two and three countries, respectively, is shown in Table 3-1 (page 133).

Table 3-1 Example country list atom

Field Size	Field	Field Contents	Comment
32-bit	atom_size	26	Size of this country list atom in bytes.

Copyright © 2004, 2012 Apple Inc. All Rights Reserved.

Field Size	Field	Field Contents	Comment
32-bit	atom_type	'ctry'	
32-bit	entry_count	2	Number of country lists.
16-bit	country_count	2	Number of countries in country list 1.
16-bit	country	'US'	
16-bit	country	'UK'	
16-bit	country_count	3	Number of countries in country list 2.
16-bit	country	'JP'	
16-bit	country	'US'	
16-bit	country	'FR'	

Language List Atom

When one or more items must be identified as being suitable for more than one language, each list of languages is stored in this otherwise optional atom. The language list atom is a full atom with an atom type of `'lang'`.

Each list starts with a 2-byte count of the number of items in the list, and then each ISO 639-2/T code, packed into two bytes, according to the ISO Language Code definition in the MP4 specification.

The atom consists of a count of the number of lists, expressed as a 32-bit integer, and then these lists, appended end-to-end. The language list atom contains the following fields:

Size
: A 32-bit unsigned integer that indicates the size in bytes of the atom structure

Type
: A 32-bit unsigned integer value set to `'lang'`

Version
: One byte that is set to 0.

Flags
: Three bytes that are set to 0.

Entry_count
: A 32-bit integer indicating the number of language arrays to follow in this atom.

2012-08-14 | Copyright © 2004, 2012 Apple Inc. All Rights Reserved.

Language_count
A 16-bit integer indicating the number of languages in the array.

Language[Language_count]
An array of 16-bit integers, defined according to the ISO 639-2/T definition of language codes.

Note that:

- Indexes into the Language List atom are 1-based.
- Zero (0) is reserved and never used as an index.
- Currently, there is a limit of 255 languages that may be recorded in a Language List atom.

Table 3-2 (page 135) shows an example Language List atom consisting of two language lists with three and two languages, respectively.

Table 3-2 Example Language List atom

Field Size	Field	Field Contents	Comment
32-bit	atom_size	26	Size of this Language List atom in bytes.
32-bit	atom_type	'lang'	
32-bit	entry_count	2	Number of language lists.
16-bit	language_count	3	Number of languages in language list 1.
16-bit	language	5575	Packed ISO code for 'eng' (English)
16-bit	language	6721	Packed ISO code for 'fra' (French)
16-bit	language	4277	Packed ISO code for 'deu' (German)
16-bit	language_count	2	Number of languages in language list 2.
16-bit	language	19969	Packed ISO code for 'spa' (Spanish)
16-bit	language	16882	Packed ISO code for 'por' (Portuguese)

Metadata Item Keys Atom

The metadata item keys atom holds a list of the metadata keys that may be present in the metadata atom. This list is indexed starting with 1; 0 is a reserved index value. The metadata item keys atom is a full atom with an atom type of `'keys'`.

2012-08-14 | Copyright © 2004, 2012 Apple Inc. All Rights Reserved.

This atom has the following structure:

Size
: A 32-bit unsigned integer that indicates the size in bytes of the atom structure

Type
: A 32-bit unsigned integer value set to `'keys'`

Version
: One byte that is set to 0

Flags
: Three bytes that are set to 0

Entry_count
: A 32-bit integer indicating the number of key arrays to follow in this atom

Key_size
: A 32-bit integer indicating the size of the entire structure containing a key definition. Therefore the key_size = sizeof(key_size) + sizeof(key_namespace) + sizeof(key_value). Since key_size and key_namespace are both 32 bit integers, together they have a size of 8 bytes. Hence, the key_value structure will be equal to key_size - 8.

Key_namespace
: A 32-bit integer defining a naming scheme used for metadata keys. Location metadata keys, for example, use the `‘mdta’` key namespace.

Key_value[Key_size-8]
: An array of 8-bit integers, each containing the actual name of the metadata key. Keys with the ‘mdta’ namespace use a reverse DNS naming convention. For example, the location metadata coordinates use a metadata key_value of ‘com.apple.quicktime.location.ISO6709’.

Note that:

- Indexes into the metadata item keys atom are 1-based (1…entry_count).
- Zero (0) is reserved and never used as an index.
- The structure of key_value depends upon the key namespace.

2012-08-14 | Copyright © 2004, 2012 Apple Inc. All Rights Reserved.

Figure 3-2 (page 137) shows a sample layout for this atom.

Figure 3-2 A typical metadata item keys atom

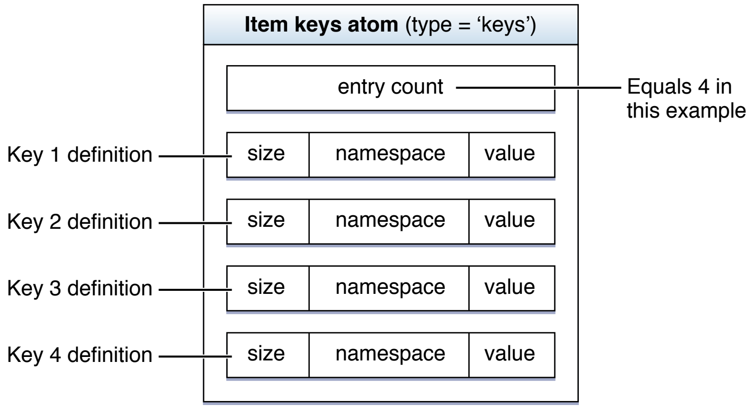

Figure 3-3 (page 137) shows an example of a metadata item keys atom consisting of three keys: two from one key namespace and a third from another key namespace.

Figure 3-3 An example of a metadata item keys atom

	**'keys'**		
	entry_count = 3		
	key_size (uint32)	key_namespace (uint32)	key_value (uint8[ ])
1	38	'mdta'	com.apple.quicktime.copyright
2	35	'mdta'	com.apple.quicktime.author
3	12	'udta'	©cpy

Metadata Item List Atom

The metadata item list atom holds a list of actual metadata values that are present in the metadata atom. The metadata items are formatted as a list of items. The metadata item list atom is of type `'ilst'` and contains a number of metadata items, each of which is an atom.

2012-08-14 | Copyright © 2004, 2012 Apple Inc. All Rights Reserved.

Figure 3-4 (page 138) shows the connection between keys.

Figure 3-4 The metadata item list atom and the item/key Connection

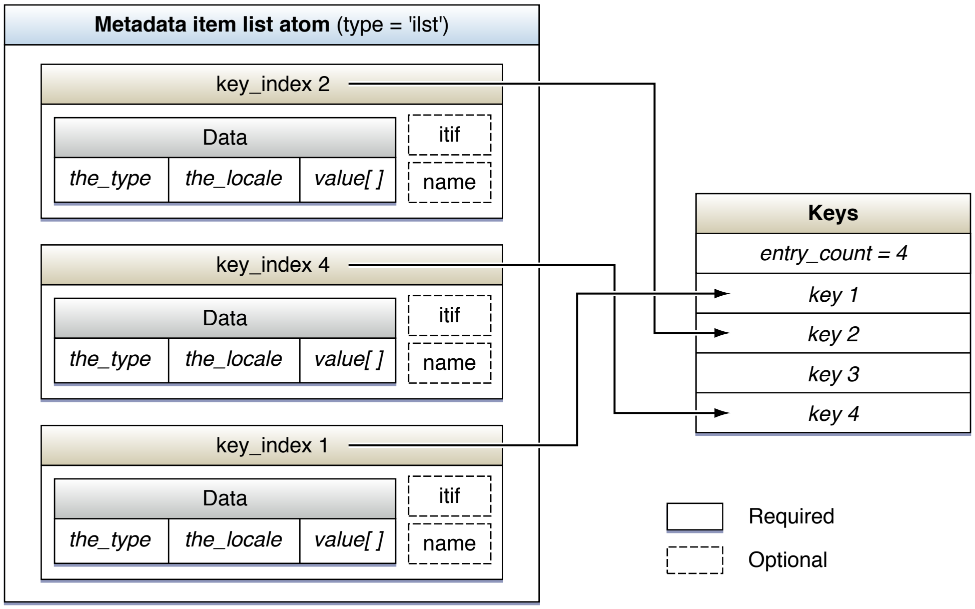

Metadata Item Atom

Each item in the metadata item list atom is identified by its key. The atom type for each metadata item atom should be set equal to the index of the key for the metadata within the item atom, taking this index from the metadata item keys atom. In addition, each metadata item atom contains a "Value Atom" (page 139), to hold the value of the metadata item.

The metadata item atom has the following structure:

Item_info

An optional item information atom, see "Item Information Atom (ID and flags)" (page 141)

Name

An optional name atom, defined below.

Data value atom[]

An array of value atoms, defined below.

2012-08-14 | Copyright © 2004, 2012 Apple Inc. All Rights Reserved.

Value Atom

The value of the metadata item is expressed as immediate data in a value atom. The value atom starts with two fields: a type indicator, and a locale indicator. Both the type and locale indicators are four bytes long. There may be multiple ‘value’ entries, using different type, country or language codes (see the Data Ordering section below for the required ordering).

The Value atom structure contains the following fields:

Type
: A type indicator, defined in “Type Indicator” (page 139).

Locale
: A locale indicator, defined in “Locale Indicator” (page 139).

Type Indicator

The type indicator is formed of four bytes split between two fields. The first byte indicates the set of types from which the type is drawn. The second through fourth byte forms the second field and its interpretation depends upon the value in the first field.

The indicator byte must have a value of 0, meaning the type is drawn from the well-known set of types. All other values are reserved.

If the type indicator byte is 0, the following 24 bits hold the well-known type. Please refer to the list of Well-Known Types, in the Well-Known Types section below.

Locale Indicator

The locale indicator is formatted as a four-byte value. It is formed from two two-byte values: a country indicator, and a language indicator. In each case, the two-byte field has the possible values shown in Table 3-3 (page 139).

Table 3-3 Country and language indicators

Value	Meaning
0	This atom provides the default value of this datum for any locale not explicitly listed.
1 to 255	The value is an index into the country or language list (the upper byte is 0).

2012-08-14 | Copyright © 2004, 2012 Apple Inc. All Rights Reserved.

Value	Meaning
otherwise	The value is an ISO 3166 code (for the country code) or a packed ISO 639-2/T code (for the language).

Note that both ISO 3166 and ISO 639-2/T codes have a nonzero value in their top byte, and so will have a value > 255.

Software applications that read metadata may be customized for a specific set of countries or languages. If a metadata writer does not want to limit a metadata item to a specific set of countries, it should use the reserved value ZZ from ISO 3166 as its country code. Similarly if the metadata writer does not want to limit the user's language (this is not recommended) it uses the value 'und' (undetermined) from the ISO 639-2/T specification.

A software application matches a country code if either (a) the value to be matched to is 0 (default) or (b) the codes are equal. A software application matches to a list of codes if its value is a member of that list. A software application matches to a locale if both country and language match.

Table 3-4 (page 140) shows some example metadata tags.

Table 3-4 Example metadata tags

Country	Language	Meaning
0	eng	All speakers of English, regardless of country
GB	0	All people in the United Kingdom, regardless of language
CA	fra	French speakers in Canada
DE,GB,FR,IT	0	People in Germany, France, United Kingdom, and Italy, regardless of language
DE,GB,FR,IT	deu,fra	People in Germany, France, United Kingdom, and Italy, who speak German or French
0	0	Default, all speakers in all countries

To reiterate, if the country_indicator value is in the range 1 to 255, it is interpreted as the 1-based index for a country list in the Country Language atom in the Metadata atom. If the language_indicator value is in the range 1 to 255, it is interpreted as the 1-based index for a language list in the Language List atom in the Metadata atom. Otherwise, the country_indicator or language_indicator is unspecified (0) or holds the immediate value for a single country or language.

Copyright © 2004, 2012 Apple Inc. All Rights Reserved.

Item Information Atom (ID and flags)

The optional item information atom contains information about the item: item-specific flags and item optional identifier. This ID must be unique within the metadata atom. To simplify assignment of item identifiers, the metadata header atom's nextItemInfo field can be used as described in "Metadata Header Atom" (page 131).

The item information atom must be present if the item has an assigned ID or has nonzero flags.

No flags are currently defined; they should be set to 0 in this version of the specification.

The item information atom is a full atom with an atom type of `'itif'`. This atom has the following structure:

Size
: A 32-bit unsigned integer that indicates the size in bytes of the atom structure

Type
: A 32-bit unsigned integer value set to `'itif'`

Version
: One byte that is set to 0.

Flags
: Three bytes that are set to 0.

Item_ID
: An unsigned 32-bit integer, unique within the container.

Name

The Name atom is a full atom with an atom type of 'name'. This atom contains a metadata name formatted as a string of UTF-8 characters, to fill the atom. It is optional. If it is not present, the item is unnamed, and cannot be referred to by name. Names are not user visible; they provide a way to refer to metadata items. The maximum length of a name may be limited in specific environments.

No two metadata items may have the same name.

This atom has the following structure:

Version
: One byte that is set to 0.

Flags
: Three bytes that are set to 0.

Copyright © 2004, 2012 Apple Inc. All Rights Reserved.

Name
: An array of bytes, constituting a UTF-8 string, containing the name.

Data Atom Structure

The Data atom has an atom type of 'data', and contains four bytes each of type and locale indicators, as specified in "Type Indicator" (page 139) and "Locale Indicator" (page 139), and then the actual value of the metadata, formatted as required by the type.

This atom has the following structure:

Type Indicator
: The type indicator, as defined in "Type Indicator" (page 139).

Locale Indicator
: The locale indicator, as defined in "Locale Indicator" (page 139).

Value
: An array of bytes containing the value of the metadata.

Data Ordering

Multiple values for the same tag represent multiple representations of the same information, differing either by language or storage type or by the size or nature of the data. For example, an artist name may be supplied in three ways:

- as a large JPEG of their signature
- as a smaller 'thumbnail' JPEG of their signature
- as text

An application may then choose the variation of the the artist name to display based on the size it needs.

Data must be ordered in each item from the most-specific data to the most general. An application may, if it wishes, stop 'searching' for a value once it finds a value that it can display (it has an acceptable locale and type).

2012-08-14 | Copyright © 2004, 2012 Apple Inc. All Rights Reserved.

Well-Known Types

The basic data-type list is in Table 3-5 (page 143).

Table 3-5 Well-known data types

Code	Type	Comment
0	reserved	Reserved for use where no type needs to be indicated
1	UTF-8	Without any count or NULL terminator
2	UTF-16	Also known as UTF-16BE
3	S/JIS	Deprecated unless it is needed for special Japanese characters
4	UTF-8 sort	Variant storage of a string for sorting only
5	UTF-16 sort	Variant storage of a string for sorting only
13	JPEG	In a JFIF wrapper
14	PNG	In a PNG wrapper
21	BE Signed Integer	A big-endian signed integer in 1,2,3 or 4 bytes
22	BE Unsigned Integer	A big-endian unsigned integer in 1,2,3 or 4 bytes; size of value determines integer size
23	BE Float32	A big-endian 32-bit floating point value (IEEE754)
24	BE Float64	A big-endian 64-bit floating point value (IEEE754)
27	BMP	Windows bitmap format graphics
28	QuickTime Metadata atom	A block of data having the structure of the Metadata atom defined in this specification

The sorting variant of text is used for languages in which sorting is not evident from the written form (for example, some forms of Asian languages). In these cases, the sorting can only be performed by a human who can identify the actual words by understanding the context. For these languages, an alternative form of the same information can be stored using a different representation of the same text which can be machine sorted. This alternative representation is still sorted according to the sort rules of the language in question, as defined for the text system in use (for example, Unicode). In general, a simple lexical sorting which compares the values of the characters alone is not sufficient.

2012-08-14 | Copyright © 2004, 2012 Apple Inc. All Rights Reserved.

Location Metadata

Many systems have the ability to detect or establish their position in a coordinate reference system. The specification “ISO 6709:2008 Standard representation of geographic point location by coordinates” describes one way of storing such information. One of the common systems is the Global Positioning System (GPS) developed by the US Department of Defense. Other systems include the ability of some cellular telephone systems to triangulate the position of cell-phones, and the possibility that IEEE 802.11 Wireless Base Stations are tagged with their position (whereupon mobile units that can ‘see’ their signal can establish that they are probably ‘near’ that location).

This support is increasingly common in still and movie cameras, or composite devices (such as camera-phones) that can function as cameras.

Apple has defined a key for storing the location coordinates as metadata, as well as several auxiliary pieces of information about a location. For all the location metadata keys defined in this specification, the Metadata atom handler-type should be ‘mdta’. See the com.apple.quicktime.location.ISO6709 entry in the following table for a description of the main location metadata key, and the additional table describing auxiliary location metadata keys.

QuickTime Metadata Keys

QuickTime has defined the keys shown in Table 3-6 (page 144) for holding data in a Metadata atom:

Table 3-6 Metadata keys

Key	Key Type	Value Payload	Definition	Example
com.apple.quicktime.album	'mdta’	A UTF-8 string (value type 1). Can have multiple values with different language and country code designations.	Album or collection name of which the movie content forms a part.	Technical documents performed to blues tunes Volume 1.

Copyright © 2004, 2012 Apple Inc. All Rights Reserved.

Key	Key Type	Value Payload	Definition	Example
com.apple.quicktime.artist	'mdta'	A UTF-8 string (value type 1). Can have multiple values with different language and country code designations.	Name of the artist who created the movie file content.	Grandma Doe and the Spe Writers.
com.apple.quicktime.artwork	'mdta'	An representative image for the movie content in a format such as JPEG (value type 13), PNG (value type 14) or BMP (value type 27). This might be album artwork, a movie poster, etc.	A single image that can represent the movie file content.	(a picture of the cover art)
com.apple.quicktime.author	'mdta'	A UTF-8 string (value type 1). Can have multiple values with different language and country code designations.	Name of the author of the movie file content.	Technical writer (anonymou

2012-08-14 | Copyright © 2004, 2012 Apple Inc. All Rights Reserved.

Key	Key Type	Value Payload	Definition	Example
com.apple.quicktime.comment	'mdta'	A UTF-8 string (value type 1). Can have multiple values with different language and country code designations.	User entered comment regarding the movie file content.	Great for a laugh.
com.apple.quicktime.copyright	'mdta'	A UTF-8 string (value type 1). Can have multiple values with different language and country code designations.	Copyright statement for the movie file content.	Copyright © 2012 Grandma Doe
com.apple.quicktime.creationdate	'mdta'	A UTF-8 string (value type 1). Can have multiple values with different language and country code designations.	The date the movie file content was created.	4/21/2012
com.apple.quicktime.description	'mdta'	A UTF-8 string (value type 1). Can have multiple values with different language and country code designations.	Description of the movie file content.	This group of engineers tak popular technical documen and does music videos performing them to novel blues tunes they write.

2012-08-14 | Copyright © 2004, 2012 Apple Inc. All Rights Reserved.

Key	Key Type	Value Payload	Definition	Example
com.apple.quicktime.director	'mdta'	A UTF-8 string (value type 1). Can have multiple values with different language and country code designations.	Name of the director of the movie content.	Papa Doe
com.apple.quicktime.title	'mdta'	A UTF-8 string (value type 1). Can have multiple values with different language and country code designations.	The title of the movie file content. This is typically a single text line.	Technical Writers Do the Blues
com.apple.quicktime.genre	'mdta'	A UTF-8 string (value type 1). Can have multiple values with different language and country code designations.	Text describing the genre or genres to which the movie content conforms. There is no prescribed vocabulary for names of genres.	Blues
com.apple.quicktime.information	'mdta'	A UTF-8 string (value type 1). Can have multiple values with different language and country code designations.	Information about the movie file content.	Recorded live on location.

2012-08-14 | Copyright © 2004, 2012 Apple Inc. All Rights Reserved.

Key	Key Type	Value Payload	Definition	Example
com.apple.quicktime.keywords	'mdta'	A UTF-8 string (value type 1). Can have multiple values with different language and country code designations.	Keywords associated with the movie file content.	Blues Specifications Group Video Music
com.apple.quicktime.location.ISO6709	'mdta'	Defined in ISO 6709:2008.	Geographic point location by coordinates as defined in ISO 6709:2008.	"+27.5916+086.5640+8850/
com.apple.quicktime.producer	'mdta'	A UTF-8 string (value type 1). Can have multiple values with different language and country code designations.	Name of producer of movie file content.	Jimmy Doe Junior
com.apple.quicktime.publisher	'mdta'	A UTF-8 string (value type 1). Can have multiple values with different language and country code designations.	Name of publisher of movie file content.	The Do-Doe Art House, Inc

2012-08-14 | Copyright © 2004, 2012 Apple Inc. All Rights Reserved.

Key	Key Type	Value Payload	Definition	Example
com.apple.quicktime.software	'mdta'	A UTF-8 string (value type 1). Can have multiple values with different language and country code designations.	Name of software used to create the movie file content.	Do-the-Blues v2.3
com.apple.quicktime.year	'mdta'	A UTF-8 string (value type 1). Can have multiple values with different language and country code designations.	Year when the movie file or the original content was created or recorded.	2012
com.apple.quicktime.collection.user	'mdta'	A UTF-8 string (value type 1). Can have multiple values with different language and country code designations.	A name indicating a user-defined collection that includes this movie.	Blues Specification Group

2012-08-14 | Copyright © 2004, 2012 Apple Inc. All Rights Reserved.

Key	Key Type	Value Payload	Definition	Example
com.apple.quicktime.rating.user	'mdta'	A BE Float32 (value type 23). The range of this number is 0.0 to 5.0, inclusive.	A number, assigned by the user, that indicates the rating or relative value of the movie. This number can range from 0.0 to 5.0. A value of 0.0 indicates that the user has not rated the movie.	4.5

In addition, QuickTime recommends the auxiliary keys shown in Table 3-7 (page 150) for holding additional metadata to be associated with a location.

Table 3-7 Auxiliary keys for metadata

Key	Key Type	Value Payload	Definition	Example
com.apple.quicktime.location.name	'mdta'	A UTF-8 string (value type 1). Can have multiple values with different language and country code designations.	Name of the location.	"Sweden" or "Grandmother's house"

Copyright © 2004, 2012 Apple Inc. All Rights Reserved.

Key	Key Type	Value Payload	Definition	Example
com.apple.quicktime.location.body	'mdta'	A UTF-8 string (value type 1). Can have multiple values with different language and country code designations.	The astronomical body, for compatibility with the 3GPP format. 'earth' is assumed if not present.	"earth"
com.apple.quicktime.location.note	'mdta'	A UTF-8 string (value type 1). Can have multiple values with different language and country code designations.	Descriptive comment.	"following a dog"
com.apple.quicktime.location.role	'mdta'	An unsigned integer (value type 22).	A single byte, binary value containing a value from the set: 0 indicates "shooting location", 1 indicates "real location", 2 indicates "fictional location". Other values are reserved.	1, for shooting location

Copyright © 2004, 2012 Apple Inc. All Rights Reserved.

Key	Key Type	Value Payload	Definition	Example
com.apple.quicktime.location.date	'mdta'	Defined in ISO8601:2004.	A date and time, stored using the extended format defined in ISO 8601:2004- Data elements and interchange format.	"2012-02-24T17:56Z" for a date of February 24, 2012, time of 17:56, UTC.
com.apple.quicktime.direction.facing	'mdta'	A UTF-8 string (value type 1)holding a machine readable direction value, as described below. This should not be tagged with a country or language code.	An indication of the direction the camera is facing during the shot.	"+20.34M/-5.3" for a heading of 20.34º magnetic, looking or going down at 5.3º below the horizontal.
com.apple.quicktime.direction.motion	'mdta'	A UTF-8 string (value type 1) holding a machine readable direction value, as described below. This should not be tagged with a country or language code.	An indication of the direction the camera is moving during the shot.	"+20.34M/-5.3" for a heading of 20.34º magnetic, looking or going down at 5.3º below the horizontal.

Direction Definition

For the metadata keys which define a direction, com.apple.quicktime.direction.facing and com.apple.quicktime.direction.motion, directions are specified as a string consisting of one or two angles, separated by a slash if two occur. The first is a compass direction, expressed in degrees and decimal degrees, optionally preceded by the characters "+" or "-", and optionally followed by the character "M". The direction is determined as accurately as possible; the nominal due north (zero degrees) is defined as facing along a line of longitude of the location system, unless the angle is followed by the "M" character indicating a magnetic heading. The second is an elevation direction, expressed in degrees and decimal degrees between +90.0 and -90.0, with 0 being horizontal (level), +90.0 being straight up, and -90.0 being straight down (and for these two cases, the compass direction is irrelevant).

2012-08-14 | Copyright © 2004, 2012 Apple Inc. All Rights Reserved.

Media Data Atom Types

QuickTime uses atoms of different types to store different types of media data—video media atoms for video data, sound media atoms for audio data, and so on. This chapter discusses in detail each of these different media data atom types.

If you are a QuickTime application or tool developer, you'll want to read this chapter in order to understand the fundamentals of how QuickTime uses atoms for storage of different media data. For the latest updates and postings, be sure to see Apple's QuickTime developer website.

This chapter is divided into the following major sections:

- "Video Media" (page 155) describes video media, which is used to store compressed and uncompressed image data in QuickTime movies.
- "Sound Media" (page 176) discusses sound media used to store compressed and uncompressed audio data in QuickTime movies.
- "Timecode Media" (page 189) describes time code media used to store time code data in QuickTime movies.
- "Text Media" (page 193) discusses text media used to store text data in QuickTime movies.
- "Closed Captioning Media" (page 198) discusses text media used to store CEA-608 closed captioning data in QuickTime movies.
- "Subtitle Media" (page 199) discusses tx3g text media used to store subtitle data in QuickTime movies.
- "Music Media" (page 206) discusses music media used to store note-based audio data, such as MIDI data, in QuickTime movies.
- "MPEG-1 Media" (page 207) discusses MPEG-1 media used to store MPEG-1 video and MPEG-1 multiplexed audio/video streams in QuickTime movies.
- "Sprite Media" (page 208) discusses sprite media used to store character-based animation data in QuickTime movies.
- "Flash Media" (page 234) is deprecated; included only for legacy.
- "Tween Media" (page 235) discusses tween media used to store pairs of values to be interpolated between in QuickTime movies.
- "Modifier Tracks" (page 247) discusses the capabilities of modifier tracks.
- "Track References" (page 248) describes a feature of QuickTime that allows you to relate a movie's tracks to one another.

2012-08-14 | Copyright © 2004, 2012 Apple Inc. All Rights Reserved.

- "3D Media" (page 249) discusses briefly how QuickTime movies store 3D image data in a base media.
- "Streaming Media" (page 250) describes how streaming media is stored in a QuickTime file.
- "Hint Media" (page 251) describes the additions to the QuickTime file format for streaming QuickTime movies over the Internet.
- "VR Media" (page 267) describes the QuickTime VR world and node information atom containers, as well as cubic panoramas, which are new to QuickTime VR 3.0.
- "Movie Media" (page 310) discusses movie media which is used to encapsulate embedded movies within QuickTime movies.

Video Media

Video media is used to store compressed and uncompressed image data in QuickTime movies. It has a media type of `'vide'`.

Video Sample Description

The video sample description contains information that defines how to interpret video media data. A video sample description begins with the four fields described in "General Structure of a Sample Description" (page 100).

Note: Some video sample descriptions contain an optional 4-byte terminator with all bytes set to 0, following all other sample description and sample description extension data. If this optional terminator is present, the sample description size value will include it. It is important to check the sample description size when parsing: more than or fewer than these four optional bytes, if present in the size value, indicates a malformed sample description.

The data format field of a video sample description indicates the type of compression that was used to compress the image data, or the color space representation of uncompressed video data. Table 4-1 (page 155) shows some of the formats supported. The list is not exhaustive, and is subject to addition.

Table 4-1 Some image compression formats

Compression type	Description
`'cvid'`	Cinepak
`'jpeg'`	JPEG
`'smc '`	Graphics

2012-08-14 | Copyright © 2004, 2012 Apple Inc. All Rights Reserved.

Compression type	Description
'rle '	Animation
'rpza'	Apple video
'kpcd'	Kodak Photo CD
'png '	Portable Network Graphics
'mjpa'	Motion-JPEG (format A)
'mjpb'	Motion-JPEG (format B)
'SVQ1'	Sorenson video, version 1
'SVQ3'	Sorenson video 3
'mp4v'	MPEG-4 video
'avc1'	H.264 video
'dvc '	NTSC DV-25 video
'dvcp'	PAL DV-25 video
'gif '	CompuServe Graphics Interchange Format
'h263'	H.263 video
'tiff'	Tagged Image File Format
'raw '	Uncompressed RGB
'2vuY´	Uncompressed Y´CbCr, 8-bit-per-component 4:2:2
'yuv2'	Uncompressed Y´CbCr, 8-bit-per-component 4:2:2
'v308'	Uncompressed Y´CbCr, 8-bit-per-component 4:4:4
'v408'	Uncompressed Y´CbCr, 8-bit-per-component 4:4:4:4
'v216'	Uncompressed Y´CbCr, 10, 12, 14, or 16-bit-per-component 4:2:2
'v410'	Uncompressed Y´CbCr, 10-bit-per-component 4:4:4
'v210'	Uncompressed Y´CbCr, 10-bit-per-component 4:2:2

2012-08-14 | Copyright © 2004, 2012 Apple Inc. All Rights Reserved.

The video media sample description adds the following fields to the general sample description.

Version
: A 16-bit integer indicating the version number of the compressed data. This is set to 0, unless a compressor has changed its data format.

Revision level
: A 16-bit integer that must be set to 0.

Vendor
: A 32-bit integer that specifies the developer of the compressor that generated the compressed data. Often this field contains '`appl`' to indicate Apple Computer, Inc.

Temporal quality
: A 32-bit integer containing a value from 0 to 1023 indicating the degree of temporal compression.

Spatial quality
: A 32-bit integer containing a value from 0 to 1024 indicating the degree of spatial compression.

Width
: A 16-bit integer that specifies the width of the source image in pixels.

Height
: A 16-bit integer that specifies the height of the source image in pixels.

Horizontal resolution
: A 32-bit fixed-point number containing the horizontal resolution of the image in pixels per inch.

Vertical resolution
: A 32-bit fixed-point number containing the vertical resolution of the image in pixels per inch.

Data size
: A 32-bit integer that must be set to 0.

Frame count
: A 16-bit integer that indicates how many frames of compressed data are stored in each sample. Usually set to 1.

Compressor name
: A 32-byte Pascal string containing the name of the compressor that created the image, such as `"jpeg"`.

Depth
: A 16-bit integer that indicates the pixel depth of the compressed image. Values of 1, 2, 4, 8 ,16, 24, and 32 indicate the depth of color images. The value 32 should be used only if the image contains an alpha channel. Values of 34, 36, and 40 indicate 2-, 4-, and 8-bit grayscale, respectively, for grayscale images.

Copyright © 2004, 2012 Apple Inc. All Rights Reserved.

Color table ID

A 16-bit integer that identifies which color table to use. If this field is set to –1, the default color table should be used for the specified depth. For all depths below 16 bits per pixel, this indicates a standard Macintosh color table for the specified depth. Depths of 16, 24, and 32 have no color table.

If the color table ID is set to 0, a color table is contained within the sample description itself. The color table immediately follows the color table ID field in the sample description. See "Color Table Atoms" (page 46) for a complete description of a color table.

Video Sample Description Extensions

Video sample descriptions can be extended by appending other atoms. These atoms are placed after the color table, if one is present. These extensions to the sample description may contain display hints for the decompressor or may simply carry additional information associated with the images. Table 4-2 (page 158) lists the currently defined extensions to video sample descriptions.

Table 4-2 Video sample description extensions

Extension type	Description
`'gama'`	A 32-bit fixed-point number indicating the gamma level at which the image was captured. The decompressor can use this value to gamma-correct at display time.
`'fiel'`	Two 8-bit integers that define field handling. This information is used by applications to modify decompressed image data or by decompressor components to determine field display order. This extension is mandatory for all uncompressed Y´CbCr data formats. The first byte specifies the field count, and may be set to 1 or 2. A value of 1 is used for progressive-scan images; a value of 2 indicates interlaced images. When the field count is 2, the second byte specifies the field ordering: which field contains the topmost scan-line, which field should be displayed earliest, and which is stored first in each sample. Each sample consists of two distinct compressed images, each coding one field: the field with the topmost scan-line, T, and the other field, B. The following defines the permitted variants: 0 – There is only one field. 1 – T is displayed earliest, T is stored first in the file. 6 – B is displayed earliest, B is stored first in the file. 9 – B is displayed earliest, T is stored first in the file. 14 – T is displayed earliest, B is stored first in the file.
`'mjqt'`	The default quantization table for a Motion-JPEG data stream.
`'mjht'`	The default Huffman table for a Motion-JPEG data stream.
`'esds'`	An MPEG-4 elementary stream descriptor atom. This extension is required for MPEG-4 video. For details, see "MPEG-4 Elementary Stream Descriptor Atom ('esds')" (page 160).

2012-08-14 | Copyright © 2004, 2012 Apple Inc. All Rights Reserved.

Extension type	Description
'avcC'	An H.264 AVCConfigurationBox. This extension is required for H.264 video as defined in ISO/IEC 14496-15. For details, see "AVC Decoder Configuration Atom ('avcC')" (page 161).
'pasp'	Pixel aspect ratio. This extension is mandatory for video formats that use non-square pixels. For details, see "Pixel Aspect Ratio ('pasp')" (page 159).
'colr'	Color parameters—an image description extension required for all uncompressed Y´CbCr video types. For details, see "Color Parameter Atoms ('colr')" (page 161).
'clap'	Clean aperture—spatial relationship of Y´CbCr components relative to a canonical image center. This allows accurate alignment for compositing of video images captured using different systems. This is a mandatory extension for all uncompressed Y´CbCr data formats. For details, see "Clean Aperture ('clap')" (page 167).

Pixel Aspect Ratio ('pasp')

This extension specifies the height-to-width ratio of pixels found in the video sample. This is a required extension for MPEG-4 and uncompressed Y´CbCr video formats when non-square pixels are used. It is optional when square pixels are used.

Size
: An unsigned 32-bit integer holding the size of the pixel aspect ratio atom.

Type
: An unsigned 32-bit field containing the four-character code `'pasp'`.

hSpacing
: An unsigned 32-bit integer specifying the horizontal spacing of pixels, such as luma sampling instants for Y´CbCr or YUV video.

vSpacing
: An unsigned 32-bit integer specifying the vertical spacing of pixels, such as video picture lines.

The units of measure for the `hSpacing` and `vSpacing` parameters are not specified, as only the ratio matters. The units of measure for height and width must be the same, however.

Table 4-3 (page 160) shows some common pixel aspect ratios.

2012-08-14 | Copyright © 2004, 2012 Apple Inc. All Rights Reserved.

Table 4-3 Common pixel aspect ratios

Description	hSpacing	vSpacing
4:3 square pixels (composite NTSC or PAL)	1	1
4:3 non-square 525 (NTSC)	10	11
4:3 non-square 625 (PAL)	59	54
16:9 analog (composite NTSC or PAL)	4	3
16:9 digital 525 (NTSC)	40	33
16:9 digital 625 (PAL)	118	81
1920x1035 HDTV (per SMPTE 260M-1992)	113	118
1920x1035 HDTV (per SMPTE RP 187-1995)	1018	1062
1920x1080 HDTV or 1280x720 HDTV	1	1

MPEG-4 Elementary Stream Descriptor Atom ('esds')

This atom contains an MPEG-4 elementary stream descriptor atom. This is a required extension to the video sample description for MPEG-4 video. This extension appears in video sample descriptions only when the codec type is `'mp4v'`.

> **Note:** The elementary stream descriptor which this atom contains is defined in the MPEG-4 specification ISO/IEC FDIS 14496-1.

Size
: An unsigned 32-bit integer holding the size of the elementary stream descriptor atom.

Type
: An unsigned 32-bit field containing the four-character code `'esds'`

Version
: An unsigned 8-bit integer set to zero.

Flags
: A 24-bit field reserved for flags, currently set to zero.

Elementary Stream Descriptor
: An elementary stream descriptor for MPEG-4 video, as defined in the MPEG-4 specification ISO/IEC 14496-1 and subject to the restrictions for storage in MPEG-4 files specified in ISO/IEC 14496-14.

2012-08-14 | Copyright © 2004, 2012 Apple Inc. All Rights Reserved.

AVC Decoder Configuration Atom (‘avcC’)

This atom contains an MPEG-4 decoder configuration atom. This is a required extension to the video sample description for H.264 video. This extension appears in video sample descriptions only when the codec type is `‘avc1’`.

> **Note:** The decoder configuration record that this atom contains is defined in the MPEG-4 specification ISO/IEC FDIS 14496-15.

Size
: An unsigned 32-bit integer holding the size of the AVC decoder configuration atom.

Type
: An unsigned 32-bit field containing the four-character-code `'avcC'`.

AVC Decoder Configuration Record
: An AVCDecoderConfigurationRecord for H.264 video, as defined in the MPEG-4 specification ISO/IEC 14496-15, and subject to the restrictions for storage in an MPEG-4 file, also specified in ISO/IEC 14496-15.

Color Parameter Atoms ('colr')

This atom is a required extension for uncompressed Y´CbCr data formats. The `'colr'` extension is used to map the numerical values of pixels in the file to a common representation of color in which images can be correctly compared, combined, and displayed. The common representation is the CIE XYZ tristimulus values (defined in Publication CIE No. 15.2).

Use of a common representation also allows you to correctly map between Y´CbCr and RGB color spaces and to correctly compensate for gamma on different systems.

The `'colr'` extension supersedes the previously defined `'gama'` Image Description extension. Writers of QuickTime files should never write both into an Image Description, and readers of QuickTime files should ignore `'gama'` if `'colr'` is present.

The `'colr'` extension is designed to work for multiple imaging applications such as video and print. Each application, driven by its own set of historical and economic realities, has its own set of parameters needed to map from pixel values to CIE XYZ.

The CIE XYZ representation is mapped to various stored Y´CbCr formats using a common set of transfer functions and matrixes. The transfer function coefficients and matrix values are stored as indexes into a table of canonical references. This provides support for multiple video systems while limiting the scope of possible values to a set of recognized standards.

2012-08-14 | Copyright © 2004, 2012 Apple Inc. All Rights Reserved.

The 'colr' atom contains four fields: a color parameter type and three indexes. The indexes are to a table of primaries, a table of transfer function coefficients, and a table of matrixes.

Figure 4-1 (page 162) shows the layout of this atom.

Figure 4-1 The layout of a color atom

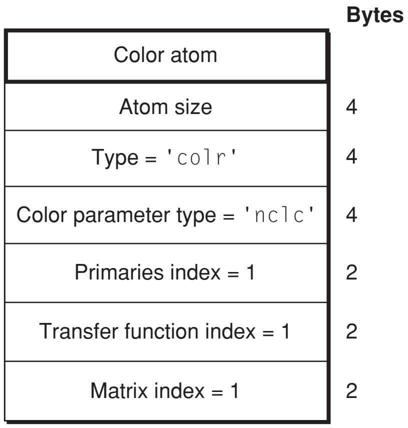

The table of matrixes specifies the matrix used during the translation, as shown in Figure 4-2 (page 163).

Color parameter type

A 32-bit field containing a four-character code for the color parameter type. The currently defined types are 'nclc' for video, and 'prof' for print. The color parameter type distinguishes between print and video mappings.

If the color parameter type is 'prof', then this field is followed by an ICC profile. This is the color model used by Apple's ColorSync. The contents of this type are not defined in this document. Contact Apple Computer for more information on the 'prof' type 'colr' extension.

If the color parameter type is 'nclc' then this atom contains the following fields:

Primaries index

A 16-bit unsigned integer containing an index into a table specifying the CIE 1931 xy chromaticity coordinates of the white point and the red, green, and blue primaries. The table of primaries specifies the white point and the red, green, and blue primary color points for a video system.

Transfer function index

A 16-bit unsigned integer containing an index into a table specifying the nonlinear transfer function coefficients used to translate between RGB color space values and Y´CbCr values. The table of transfer function coefficients specifies the nonlinear function coefficients used to translate between the stored Y´CbCr values and a video capture or display system, as shown in Figure 4-2 (page 163).

Copyright © 2004, 2012 Apple Inc. All Rights Reserved.

Matrix index

A 16-bit unsigned integer containing an index into a table specifying the transformation matrix coefficients used to translate between RGB color space values and Y´CbCr values. The table of matrixes specifies the matrix used during the translation, as shown in Figure 4-2 (page 163).

The transfer function and matrix are used as shown in Figure 4-2 (page 163).

Figure 4-2 Transfer between RGB and Y´CbCr color spaces

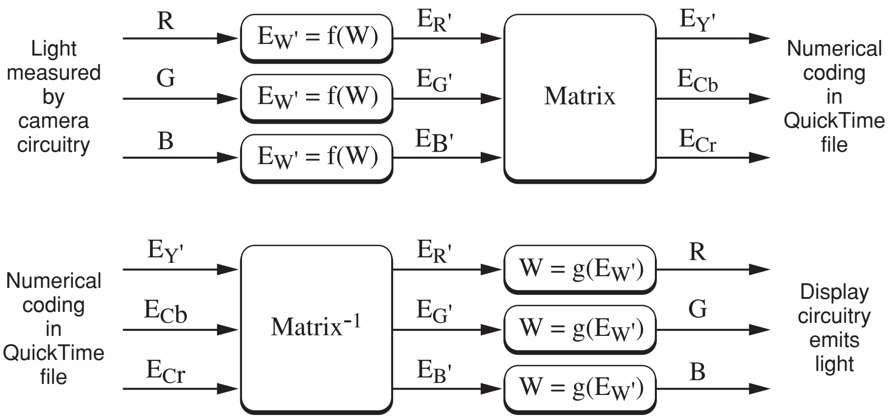

The Y´CbCr values stored in a file are normalized to a range of [0,1]for Y´ and [-0.5, +0.5] for Cb and Cr when performing these operations. The normalized values are then scaled to the proper bit depth for a particular Y´CbCr format before storage in the file as shown in Figure 4-3 (page 163).

Figure 4-3 Normalized values, using the symbol E with a subscript for Y´, Cb, or Cr

$E_{Y'}$ has the range [0, 1]

E_{Cb} has the range [-0.5, +0.5]

E_{Cr} has the range [-0.5, +0.5]

Note: The symbols used for these values are not intended to correspond to the use of these same symbols in other standards. In particular, "E" should not be interpreted as voltage.

These normalized values can be mapped onto the stored integer values of a particular compression type's Y´, Cb, and Cr components using two different schemes, which we will call Scheme A and Scheme B.

Warning: Other, slightly different encoding/mapping schemes exist in the video industry, and data encoded using these schemes must be converted to one of the QuickTime schemes defined here.

Copyright © 2004, 2012 Apple Inc. All Rights Reserved.

Scheme A uses "Wide-Range" mapping (full scale) with unsigned Y´ and twos-complement Cb and Cr values as shown in Figure 4-4 (page 164).

Figure 4-4 Equations for stored Y´CbCr values of bit-depth of n in scheme A

$$Y' = \text{floor}\,(0.5 + (2^{n-1}) * E_{Y'})$$

$$Cb = \text{floor}\,(0.5 + (2^{n-2}) * E_{Cb})$$

$$Cr = \text{floor}\,(0.5 + (2^{n-2}) * E_{Cr})$$

This maps normalized values to stored values so that, for example, 8-bit unsigned values for Y´ go from 0-255 as the normalized value goes from 0 to 1, and 8-bit signed valued for Cb and Cr go from -127 to +127 as the normalized values go from -0.5 to +0.5.

Warning: In specifications such as ITU-R BT.601-4, JFIF 1.02, and SPIFF (Rec. ITU-T T.84), the symbols Cb and Cr are used to describe offset binary integers, not twos-complement signed integers shown here.

Scheme B uses "Video-Range" mapping with unsigned Y´ and offset binary Cb and Cr values.

Note: Scheme B, shown in Figure 4-5 (page 164), comes from digital video industry specifications such as Rec. ITU-R BT. 601-4. All standard digital video tape formats (e.g., SMPTE D-1, SMPTE D-5) and all standard digital video links (e.g., SMPTE 259M-1997 serial digital video) use this scheme. Professional video storage and processing equipment from vendors such as Abekas, Accom, and SGI also use this scheme. MPEG-2, DVC and many other codecs specify source Y´CbCr pixels using this scheme.

Figure 4-5 Equations for stored Y´CbCr values of bit-depth n in scheme B

$$Y' = \text{floor}\,(0.5 + 2^{n-8} * (219 * E_{Y'} + 16))$$

$$Cb = \text{floor}\,(0.5 + 2^{n-8} * (224 * E_{Cb} + 128))$$

$$Cr = \text{floor}\,(0.5 + 2^{n-8} * (224 * E_{Cr} + 128))$$

This maps the normalized values to stored values so that, for example, 8-bit unsigned values for Y´ go from 16 to 235 as the normalized value goes from 0 to1, and 8-bit unsigned valued for Cb and Cr go from 16 to 240 as the normalized values go from -0.5 to +0.5.

For 10-bit samples, Y´ has a range of 64 to 940 as the normalized value goes from 0 to 1, and Cb and Cr have the range of 65–960 as the normalized values go from –0.5 to +0.5.

2012-08-14 | Copyright © 2004, 2012 Apple Inc. All Rights Reserved.

Y´ is an unsigned integer. Cb and Cr are offset binary integers.

Certain Y´, Cb, and Cr component values v are reserved as synchronization signals and must not appear in a buffer. For n = 8 bits, these are values 0 and 255. For n = 10 bits, these are values 0, 1, 2, 3, 1020, 1021, 1022, and 1023. The writer of a QuickTime image is responsible for omitting these values. The reader of a QuickTime image may assume that they are not present.

The remaining component values that fall outside the mapping for scheme B (1 to 15 and 241 to 254 for n = 8 bits and 4 to 63 and 961 to 1019 for n = 10 bits) accommodate occasional filter undershoot and overshoot in image processing. In some applications, these values are used to carry other information (e.g., transparency). The writer of a QuickTime image may use these values and the reader of a QuickTime image must expect these values.

The following tables show the primary values, transfer functions, and matrixes indicated by the index entries in the `'colr'` atom.

The R, G, and B values in Table 4-4 (page 165) are tristimulus values (such as candelas/meter^2), whose relationship to CIE XYZ values can be derived from the primaries and white point specified in the table, using the method described in SMPTE RP 177-1993. In this instance, the R, G, and B values are normalized to the range [0,1].

Table 4-4 Table of primaries, index, and values

Index	Values
0	Reserved
1	Recommendation ITU-R BT.709-2, SMPTE 274M-1995, and SMPTE 296M-1997 white x = 0.3127 y = 0.3290 (CIE III. D65) red x=0.640 y = 0.330 green x = 0.300 y = 0.600 blue x = 0.150 y = 0.060
2	Primary values are unknown
3–4	Reserved
5	SMPTE RP 145-1993, SMPTE170M-1994, 293M-1996, 240M-1995, and SMPTE 274M-1995 white x = 0.3127 y = 0.3290 (CIE III. D65) red x = 0.64 y = 0.33 green x = 0.29 y = 0.60 blue x = 0.15 y = 0.06
6	ITU-R BT.709-2, SMPTE 274M-1995, and SMPTE 296M-1997 white x = 0.3127 y = 0.3290 (CIE III. D65) red x = 0.630 y = 0.340 green x = 0.310 y = 0.595 blue x = 0.155 y = 0.070
7–65535	Reserved

The transfer functions listed in Table 4-5 (page 166) are used as shown in Figure 4-2 (page 163).

2012-08-14 | Copyright © 2004, 2012 Apple Inc. All Rights Reserved.

Table 4-5 Table of transfer function index and values

Index	Video Standards
0	Reserved
1	Recommendation ITU-R BT.709-2, SMPTE 274M-1995, 296M-1997, 293M-1996, 170M-1994 See below for transfer function equations.
2	Coefficient values are unknown
3–6	Reserved
7	Recommendation SMPTE 240M-1995 and 274M-1995 See below for transfer function equations.
8–65535	Reserved

The MPEG-2 sequence display extension `transfer_sics` defines a code 6 whose transfer function is identical to that in code 1. QuickTime writers should map 6 to 1 when converting from `transfer_characteristics` to `transferFunction`.

Recommendation ITU-R BT.470-4 specified an "assumed gamma value of the receiver for which the primary signals are pre-corrected" as 2.2 for NTSC and 2.8 for PAL systems. This information is both incomplete and obsolete. Modern 525- and 625-line digital and NTSC/PAL systems use the transfer function with code 1.

Figure 4-6 Equations for index code 1

$$E_{W}' = 4.500\ W \text{ for } 0 <= W < 0.018$$

$$E_{W}' = 1.099\ W^{0.45} - 0.099 \text{ for } 0.018 <= W <= 1$$

Figure 4-7 Equations for index code 7

$$E_{W}' = 4\ W \text{ for } 0 <= W < 0.0228$$

$$E_{W}' = 1.1115\ W^{0.45} - 0.115 \text{ for } 0.0228 <= W <= 1$$

The matrix values are shown in Table 4-6 (page 167) and in Figure 4-8 (page 167), Figure 4-9 (page 167), and Figure 4-10 (page 167). These figures show a formula for obtaining the normalized value of Y´ in the range [0,1]. You can derive the formula for normalized values of Cb and Cr as follows:

If the equation for normalized Y´ has the form:

$$E_{Y'} = K_{G'} E_{G'} + K_{B'} E_{B'} + K_{R'} E_{R'}$$

Then the formulas for normalized Cb and Cr are:

Copyright © 2004, 2012 Apple Inc. All Rights Reserved.

$E_{Cb} = (0.5/(1 - K_B')) (E_B' - E_Y')$

$E_{Cr} = (0.5/(1 - K_R')) (E_R' - E_Y')$

Table 4-6 Table of matrix index and values

Index	Video Standard
0	Reserved
1	Recommendation ITU-R BT.709-2 (1125/60/2:1 only), SMPTE 274M-1995, 296M-1997 See below for matrix values.
2	Coefficient values are unknown
3–5	Reserved
6	Recommendation ITU-R BT.601-4 and BT.470-4 System B and G, SMPTE 170M-1994, 293M-1996 See below for matrix values
7	SMPTE 240M-1995, 274M-1995 See below for matrix values
8–65535	Reserved

Figure 4-8 Matrix values for index code 1

$$E_{Y}' = 0.7152\ E_{G}' + 0.0722\ E_{B}' + 0.2126\ E_{R}'$$

Figure 4-9 Matrix values for index code 6

$$E_{Y}' = 0.587\ E_{G}' + 0.114\ E_{B}' + 0.299\ E_{R}'$$

Figure 4-10 Matrix values for index code 7

$$E_{Y}' = 0.701\ E_{G}' + 0.087\ E_{B}' + 0.212\ E_{R}'$$

Clean Aperture ('clap')

The clean aperture extension defines the relationship between the pixels in a stored image and a canonical rectangular region of a video system from which it was captured or to which it will be displayed. This can be used to correlate pixel locations in two or more images—possibly recorded using different systems—for accurate compositing. This is necessary because different video digitizer devices can digitize different regions of the incoming video signal, causing pixel misalignment between images. In particular, a stored image may contain "edge" data outside the canonical display area for a given system.

2012-08-14 | Copyright © 2004, 2012 Apple Inc. All Rights Reserved.

The clean aperture is either coincident with the stored image or a subset of the stored image; if it is a subset, it may be centered on the stored image, or it may be offset positively or negatively from the stored image center.

The clean aperture extension contains a width in pixels, a height in picture lines, and a horizontal and vertical offset between the stored image center and a canonical image center for the given video system. The width is typically the width of the canonical clean aperture for a video system divided by the pixel aspect ratio of the stored data. The offsets also take into account any "overscan" in the stored image. The height and width must be positive values, but the offsets may be positive, negative, or zero.

These values are given as ratios of two 32-bit numbers, so that applications can calculate precise values with minimum roundoff error. For whole values, the value should be stored in the numerator field while the denominator field is set to 1.

Size
: A 32-bit unsigned integer containing the size of the `'clap'` atom.

Type
: A 32-bit unsigned integer containing the four-character code `'clap'`.

apertureWidth_N (numerator)
: A 32-bit signed integer containing either the width of the clean aperture in pixels or the numerator portion of a fractional width.

apertureWidth_D (denominator)
: A 32-bit signed integer containing either the denominator portion of a fractional width or the number 1.

apertureHeight_N (numerator)
: A 32-bit signed integer containing either the height of the clean aperture in picture lines or the numerator portion of a fractional height.

apertureHeight_D (denominator)
: A 32-bit signed integer containing either the denominator portion of a fractional height or the number 1.

horizOff_N (numerator)
: A 32-bit signed integer containing either the horizontal offset of the clean aperture center minus (width–1)/2 or the numerator portion of a fractional offset. This value is typically zero.

horizOff_D (denominator)
: A 32-bit signed integer containing either the denominator portion of the horizontal offset or the number 1.

2012-08-14 | Copyright © 2004, 2012 Apple Inc. All Rights Reserved.

vertOff_N (numerator)

A 32-bit signed integer containing either the vertical offset of the clean aperture center minus (height−1)/2 or the numerator portion of a fractional offset. This value is typically zero.

vertOff_D (denominator)

A 32-bit signed integer containing either the denominator portion of the vertical offset or the number 1.

Video Sample Data

The format of the data stored in video samples is completely dependent on the type of the compression used, as indicated in the video sample description. The following sections discuss some of the video encoding schemes supported by QuickTime.

Uncompressed RGB

Uncompressed RGB data is stored in a variety of different formats. The format used depends on the depth field of the video sample description. For all depths, the image data is padded on each scan line to ensure that each scan line begins on an even byte boundary.

- For depths of 1, 2, 4, and 8, the values stored are indexes into the color table specified in the color table ID field.
- For a depth of 16, the pixels are stored as 5-5-5 RGB values with the high bit of each 16-bit integer set to 0.
- For a depth of 24, the pixels are stored packed together in RGB order.
- For a depth of 32, the pixels are stored with an 8-bit alpha channel, followed by 8-bit RGB components.

RGB data can be stored in composite or planar format. Composite format stores the RGB data for each pixel contiguously, while planar format stores the R, G, and B data separately, so the RGB information for a given pixel is found using the same offset into multiple tables. For example, the data for two pixels could be represented in composite format as RGB-RGB or in planar format as RR-GG-BB.

Uncompressed Y´CbCr (including yuv2)

The Y´CbCr color space is widely used for digital video. In this data format, luminance is stored as a single value (Y), and chrominance information is stored as two color-difference components (Cb and Cr). Cb is the difference between the blue component and a reference value; Cr is the difference between the red component and a reference value.

Copyright © 2004, 2012 Apple Inc. All Rights Reserved.

This is commonly referred to as “YUV” format, with “U” standing-in for Cb and “V” standing-in for Cr. This usage is not strictly correct, as YUV, YIC, and Y´CbCr are distinct color models for PAL, NTSC, and digital video, but most Y´CbCr data formats and codecs are described or even named as some variant of “YUV.”

The values of Y, Cb, and Cr can be represented using a variety of bit depths, trading off accuracy for file size. Similarly, the chrominance values can be subsampled, recording only one pixel’s color value out of two, for example, or averaging the color value of adjacent pixels. This subsampling is a form of compression, but if no additional lossy compression is performed on the sampled video, it is still referred to as “uncompressed” Y´CbCr video. In addition, a fourth component can be added to Y´CbCr video to record an alpha channel.

The number of components (Y´CbCr with or without alpha) and any subsampling are denoted using ratios of three or four numbers, such as 4:2:2 to indicate 4 bits of Y to 2 bits each of Cb and Cr (chroma subsampling), or 4:4:4 for equal storage of Y, Cb, and Cr (no subsampling), or 4:4:4:4 for Y´CbCr plus alpha with no subsampling. The ratios do not typically denote actual bit depths.

Uncompressed Y´CbCr video data is typically stored as follows:

- Y´, Cb, and Cr components of each line are stored spatially left to right and temporally from earliest to latest.
- The lines of a field or frame are stored spatially top to bottom and temporally earliest to latest.
- Y´ is an unsigned integer. Cb and Cr are twos-complement signed integers.

The yuv2 stream, for example, is encoded in a series of 4-byte packets. Each packet represents two adjacent pixels on the same scan line. The bytes within each packet are ordered as follows:

```
y0 u y1 v
```

`y0` is the luminance value for the left pixel; `y1` the luminance for the right pixel. `u` and `v` are chromatic values that are shared by both pixels.

Accurate conversion between RGB and Y´CbCr color spaces requires a computation for each component of each pixel. An example conversion from yuv2 into RGB is represented by the following equations:

$r = 1.402 * v + y + .5$

$g = y - .7143 * v - .3437 * u + .5$

$b = 1.77 * u + y + .5$

The r, g, and b values range from 0 to 255.

2012-08-14 | Copyright © 2004, 2012 Apple Inc. All Rights Reserved.

The coefficients in these equations are derived from matrix operations and depend on the reference values used for the primary colors and for white. QuickTime uses canonical values for these reference coefficients based on published standards. The sample description extension for Y´CbCr formats includes a `'colr'` atom, which contains indexes into a table of canonical references. This provides support for multiple video standards without opening the door to data entry errors for stored coefficient values. Refer to the published standards for the formulas and methods used to derive conversion coefficients from the table entries.

JPEG

QuickTime stores JPEG images according to the rules described in the ISO JPEG specification, document number DIS 10918-1.

MPEG-4 Video

MPEG-4 video uses the `'mp4v'` data format. The sample description requires the elementary stream descriptor (`'esds'`) extension to the standard video sample description. If non-square pixels are used, the pixel aspect ratio (`'pasp'`) extension is also required. For details on these extensions, see "Pixel Aspect Ratio ('pasp')" (page 159) and "MPEG-4 Elementary Stream Descriptor Atom ('esds')" (page 160).

MPEG-4 video conforms to ISO/IEC documents 14496-1/2000(E) and 14496-2:1999/Amd.1:2000(E).

Motion-JPEG

Motion-JPEG (M-JPEG) is a variant of the ISO JPEG specification for use with digital video streams. Instead of compressing an entire image into a single bitstream, Motion-JPEG compresses each video field separately, returning the resulting JPEG bitstreams consecutively in a single frame.

2012-08-14 | Copyright © 2004, 2012 Apple Inc. All Rights Reserved.

There are two flavors of Motion-JPEG currently in use. These two formats differ based on their use of markers. Motion-JPEG format A supports markers; Motion-JPEG format B does not. The following paragraphs describe how QuickTime stores Motion-JPEG sample data. Figure 4-11 (page 172) shows an example of Motion-JPEG A dual-field sample data. Figure 4-12 (page 174) shows an example of Motion- JPEG B dual-field sample data.

Figure 4-11 Motion-JPEG A dual-field sample data

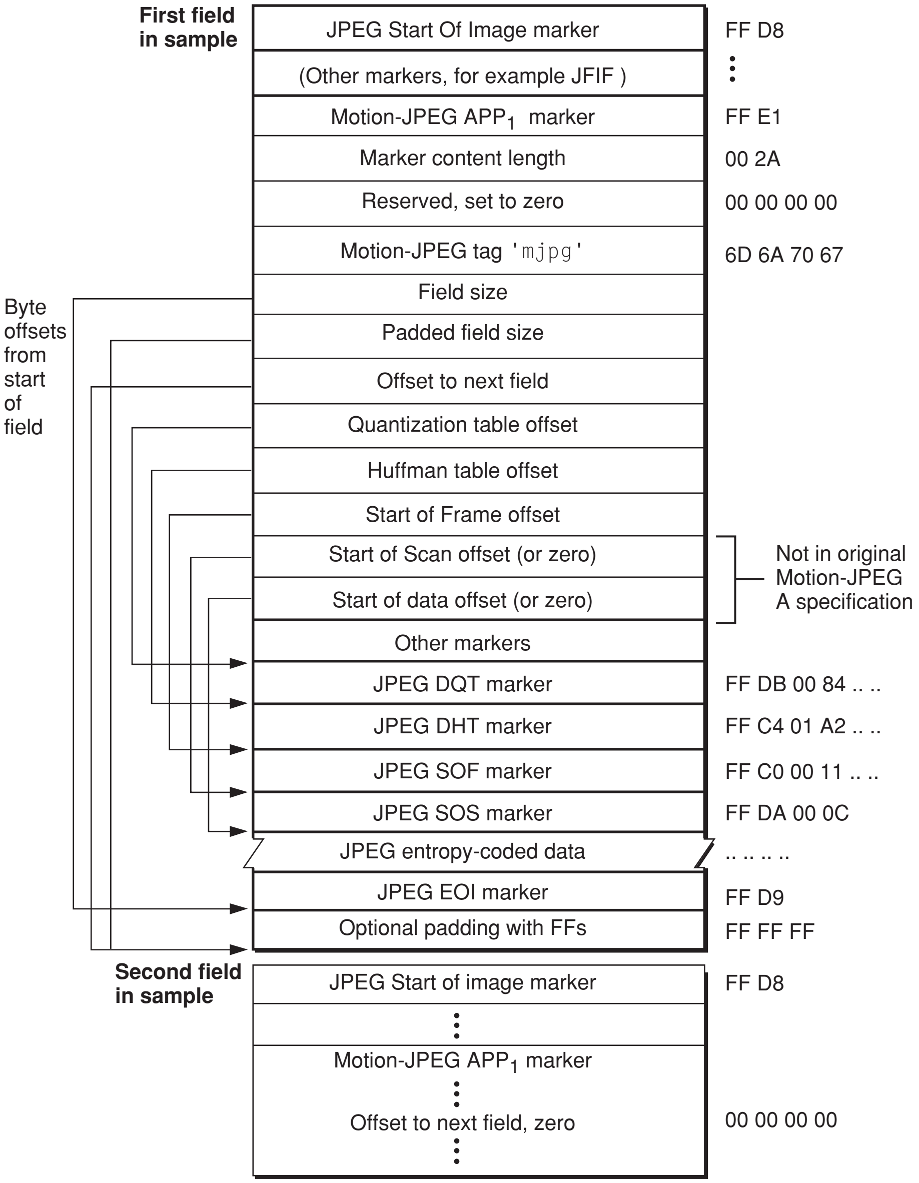

Each field of Motion-JPEG format A fully complies with the ISO JPEG specification, and therefore supports application markers. QuickTime uses the APP1 marker to store control information, as follows (all of the fields are 32-bit integers):

Reserved
: Unpredictable; should be set to 0.

Tag
: Identifies the data type; this field must be set to `'mjpg'`.

Field size
: The actual size of the image data for this field, in bytes.

Padded field size
: Contains the size of the image data, including pad bytes. Some video hardware may append pad bytes to the image data; this field, along with the field size field, allows you to compute how many pad bytes were added.

Offset to next field
: The offset, in bytes, from the start of the field data to the start of the next field in the bitstream. This field should be set to 0 in the last field's marker data.

Quantization table offset
: The offset, in bytes, from the start of the field data to the quantization table marker. If this field is set to 0, check the image description for a default quantization table.

Huffman table offset
: The offset, in bytes, from the start of the field data to the Huffman table marker. If this field is set to 0, check the image description for a default Huffman table.

Start of frame offset
: The offset from the start of the field data to the start of image marker. This field should never be set to 0.

Start of scan offset
: The offset, in bytes, from the start of the field data to the start of the scan marker. This field should never be set to 0.

Start of data offset
: The offset, in bytes, from the start of the field data to the start of the data stream. Typically, this immediately follows the start of scan data.

2012-08-14 | Copyright © 2004, 2012 Apple Inc. All Rights Reserved.

> **Note:** The last two fields have been added since the original Motion-JPEG specification, and so they may be missing from some Motion-JPEG A files. You should check the length of the APP1 marker before using the start of scan offset and start of data offset fields.

Motion-JPEG format B does not support markers. In place of the marker, therefore, QuickTime inserts a header at the beginning of the bitstream. Again, all of the fields are 32-bit integers.

Figure 4-12 Motion-JPEG B dual-field sample data

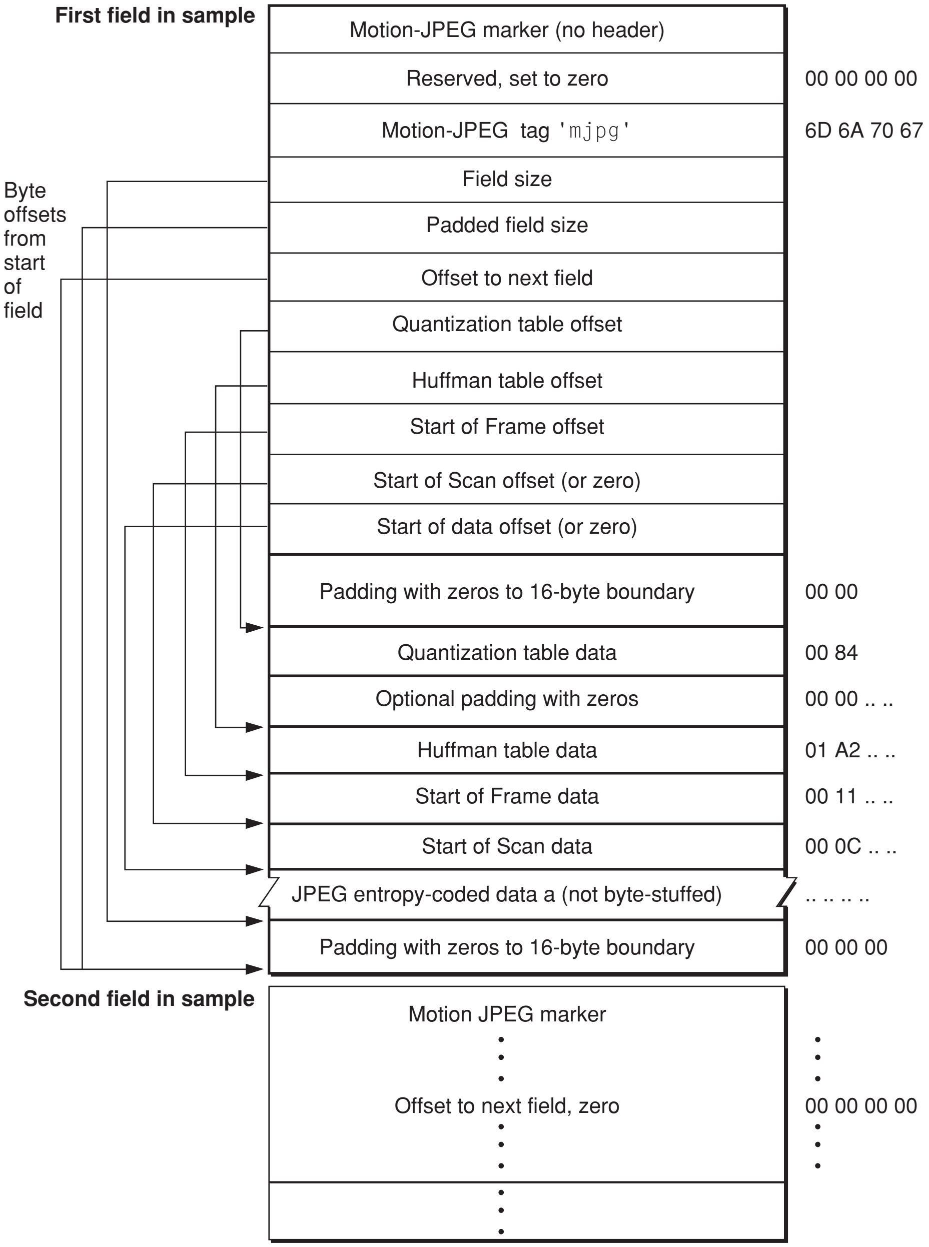

2012-08-14 | Copyright © 2004, 2012 Apple Inc. All Rights Reserved.

Reserved
: Unpredictable; should be set to 0.

Tag
: The data type; this field must be set to `'mjpg'`.

Field size
: The actual size of the image data for this field, in bytes.

Padded field size
: The size of the image data, including pad bytes. Some video hardware may append pad bytes to the image data; this field, along with the field size field, allows you to compute how many pad bytes were added.

Offset to next field
: The offset, in bytes, from the start of the field data to the start of the next field in the bitstream. This field should be set to 0 in the second field's header data.

Quantization table offset
: The offset, in bytes, from the start of the field data to the quantization table. If this field is set to 0, check the image description for a default quantization table.

Huffman table offset
: The offset, in bytes, from the start of the field data to the Huffman table. If this field is set to 0, check the image description for a default Huffman table.

Start of frame offset
: The offset from the start of the field data to the field's image data. This field should never be set to 0.

Start of scan offset
: The offset, in bytes, from the start of the field data to the start of scan data.

Start of data offset
: The offset, in bytes, from the start of the field data to the start of the data stream. Typically, this immediately follows the start of scan data.

Note: The last two fields were "reserved, must be set to zero" in the original Motion-JPEG specification.

The Motion-JPEG format B header must be a multiple of 16 in size. When you add pad bytes to the header, set them to 0.

Because Motion-JPEG format B does not support markers, the JPEG bitstream does not have NULL bytes (0x00) inserted after data bytes that are set to 0xFF.

2012-08-14 | Copyright © 2004, 2012 Apple Inc. All Rights Reserved.

Sound Media

Sound media is used to store compressed and uncompressed audio data in QuickTime movies. It has a media type of 'soun'. This section describes the sound sample description and the storage format of sound files using various data formats.

Sound Sample Descriptions

The sound sample description contains information that defines how to interpret sound media data. This sample description is based on the standard sample description, as described in "Sample Description Atoms" (page 99).

The data format field contains the format of the audio data. This may specify a compression format or one of several uncompressed audio formats. Table 4-7 (page 176) shows a list of some supported sound formats.

Table 4-7 Partial list of supported QuickTime audio formats.

Format	4-Character code	Description
Not specified	0x00000000	This format descriptor should not be used, but may be found in some files. Samples are assumed to be stored in either 'raw ' or 'twos' format, depending on the sample size field in the sound description.
kSoundNotCompressed	'NONE'	This format descriptor should not be used, but may be found in some files. Samples are assumed to be stored in either 'raw ' or 'twos' format, depending on the sample size field in the sound description.
k8BitOffsetBinary-Format	'raw '	Samples are stored uncompressed, in offset-binary format (values range from 0 to 255; 128 is silence). These are stored as 8-bit offset binaries.
k16BitBigEndianFormat	'twos'	Samples are stored uncompressed, in two's-complement format (sample values range from -128 to 127 for 8-bit audio, and -32768 to 32767 for 1- bit audio; 0 is always silence). These samples are stored in 16-bit big-endian format.
k16BitLittleEndian-Format	'sowt'	16-bit little-endian, twos-complement

2012-08-14 | Copyright © 2004, 2012 Apple Inc. All Rights Reserved.

Format	4-Character code	Description
kMACE3Compression	'MAC3 '	Samples have been compressed using MACE 3:1. (Obsolete.)
kMACE6Compression	'MAC6 '	Samples have been compressed using MACE 6:1. (Obsolete.)
kIMACompression	'ima4'	Samples have been compressed using IMA 4:1.
kFloat32Format	'fl32'	32-bit floating point
kFloat64Format	'fl64'	64-bit floating point
k24BitFormat	'in24'	24-bit integer
k32BitFormat	'in32'	32-bit integer
kULawCompression	'ulaw'	uLaw 2:1
kALawCompression	'alaw'	uLaw 2:1
kMicrosoftADPCMFormat	0x6D730002	Microsoft ADPCM-ACM code 2
kDVIIntelIMAFormat	0x6D730011	DVI/Intel IMAADPCM-ACM code 17
kDVAudioFormat	'dvca'	DV Audio
kQDesignCompression	'QDMC'	QDesign music
kQDesign2Compression	'QDM2'	QDesign music version 2
kQUALCOMMCompression	'Qclp'	QUALCOMM PureVoice
kMPEGLayer3Format	0x6D730055	MPEG-1 layer 3, CBR only (pre-QT4.1)
kFullMPEGLay3Format	'.mp3'	MPEG-1 layer 3, CBR & VBR (QT4.1 and later)
kMPEG4AudioFormat	'mp4a'	MPEG-4, Advanced Audio Coding (AAC)
kAC3AudioFormat	'ac-3'	Digital Audio Compression Standard (AC-3, Enhanced AC-3)

Copyright © 2004, 2012 Apple Inc. All Rights Reserved.

Sound Sample Description (Version 0)

There are currently three versions of the sound sample description, versions 0, 1 and 2. Version 0 supports only uncompressed audio in raw (`'raw '`) or twos-complement (`'twos'`) format, although these are sometimes incorrectly specified as either `'NONE'` or 0x00000000.

Version
: A 16-bit integer that holds the sample description version (currently 0 or 1).

Revision level
: A 16-bit integer that must be set to 0.

Vendor
: A 32-bit integer that must be set to 0.

Number of channels
: A 16-bit integer that indicates the number of sound channels used by the sound sample. Set to 1 for monaural sounds, 2 for stereo sounds. Higher numbers of channels are not supported.

Sample size (bits)
: A 16-bit integer that specifies the number of bits in each uncompressed sound sample. Allowable values are 8 or 16. Formats using more than 16 bits per sample set this field to 16 and use sound description version 1.

Compression ID
: A 16-bit integer that must be set to 0 for version 0 sound descriptions. This may be set to –2 for some version 1 sound descriptions; see "Redefined Sample Tables" (page 180).

Packet size
: A 16-bit integer that must be set to 0.

Sample rate
: A 32-bit unsigned fixed-point number (16.16) that indicates the rate at which the sound samples were obtained. The integer portion of this number should match the media's time scale. Many older version 0 files have values of 22254.5454 or 11127.2727, but most files have integer values, such as 44100. Sample rates greater than 2^16 are not supported.

Version 0 of the sound description format assumes uncompressed audio in `'raw '` or `'twos'` format, 1 or 2 channels, 8 or 16 bits per sample, and a compression ID of 0.

Sound Sample Description (Version 1)

The version field in the sample description is set to 1 for this version of the sound description structure. In version 1 of the sound description, introduced in QuickTime 3, the sound description record is extended by 4 fields, each 4 bytes long, and includes the ability to add atoms to the sound description.

2012-08-14 | Copyright © 2004, 2012 Apple Inc. All Rights Reserved.

These added fields are used to support out-of-band configuration settings for decompression and to allow some parsing of compressed QuickTime sound tracks without requiring the services of a decompressor.

These fields introduce the idea of a **packet**. For uncompressed audio, a packet is a sample from a single channel. For compressed audio, this field has no real meaning; by convention, it is treated as 1/number-of-channels.

These fields also introduce the idea of a **frame**. For uncompressed audio, a frame is one sample from each channel. For compressed audio, a frame is a compressed group of samples whose format is dependent on the compressor.

> **Important:** The value of all these fields has different meaning for compressed and uncompressed audio. The meaning may not be easily deducible from the field name.

The four new fields are:

Samples per packet
The number of uncompressed frames generated by a compressed frame (an uncompressed frame is one sample from each channel). This is also the frame duration, expressed in the media's timescale, where the timescale is equal to the sample rate. For uncompressed formats, this field is always 1.

Bytes per packet
For uncompressed audio, the number of bytes in a sample for a single channel. This replaces the older `sampleSize` field, which is set to 16.This value is calculated by dividing the frame size by the number of channels. The same calculation is performed to calculate the value of this field for compressed audio, but the result of the calculation is not generally meaningful for compressed audio.

Bytes per frame
The number of bytes in a frame: for uncompressed audio, an uncompressed frame; for compressed audio, a compressed frame. This can be calculated by multiplying the bytes per packet field by the number of channels.

Bytes per sample
The size of an uncompressed sample in bytes. This is set to 1 for 8-bit audio, 2 for all other cases, even if the sample size is greater than 2 bytes.

When capturing or compressing audio using the QuickTime API, the value of these fields can be obtained by calling the Apple Sound Manager's `GetCompression` function. Historically, the value returned for the bytes per frame field was not always reliable, however, so this field was set by multiplying bytes per packet by the number of channels.

To facilitate playback on devices that support only one or two channels of audio in `'raw '` or `'twos'` format (such as most early Macintosh and Windows computers), all other uncompressed audio formats are treated as compressed formats, allowing a simple "decompressor" component to perform the necessary format conversion during playback. The audio samples are treated as opaque compressed frames for these data types, and the fields for sample size and bytes per sample are not meaningful.

2012-08-14 | Copyright © 2004, 2012 Apple Inc. All Rights Reserved.

The new fields correspond to the `CompressionInfo` structure used by the Macintosh Sound Manager (which uses 16-bit values) to describe the compression ratio of fixed ratio audio compression algorithms. If these fields are not used, they are set to 0. File readers only need to check to see if `samplesPerPacket` is 0.

Redefined Sample Tables

If the compression ID in the sample description is set to –2, the sound track uses redefined sample tables optimized for compressed audio.

Unlike video media, the data structures for QuickTime sound media were originally designed for uncompressed samples. The extended version 1 sound description structure provides a great deal of support for compressed audio, but it does not deal directly with the sample table atoms that point to the media data.

The ordinary sample tables do not point to compressed frames, which are the fundamental units of compressed audio data. Instead, they appear to point to individual uncompressed audio samples, each one byte in size, within the compressed frames. When used with the QuickTime API, QuickTime compensates for this fiction in a largely transparent manner, but attempting to parse the sound samples using the original sample tables alone can be quite complicated.

With the introduction of support for the playback of variable bit-rate (VBR) audio in QuickTime 4.1, the contents of a number of these fields were redefined, so that a frame of compressed audio is treated as a single media sample. The sample-to-chunk and chunk offset atoms point to compressed frames, and the sample size table documents the size of the frames. The size is constant for CBR audio, but can vary for VBR.

The time-to-sample table documents the duration of the frames. If the time scale is set to the sampling rate, which is typical, the duration equals the number of uncompressed samples in each frame, which is usually constant even for VBR (it is common to use a fixed frame duration). If a different media timescale is used, it is necessary to convert from timescale units to sampling rate units to calculate the number of samples.

This change in the meaning of the sample tables allows you to use the tables accurately to find compressed frames.

To indicate that this new meaning is used, a version 1 sound description is used and the compression ID field is set to –2. The `samplesPerPacket` field and the `bytesPerSample` field are not necessarily meaningful for variable bit rate audio, but these fields should be set correctly in cases where the values are constant; the other two new fields (`bytesPerPacket` and `bytesPerFrame`) are reserved and should be set to 0.

If the compression ID field is set to zero, the sample tables describe uncompressed audio samples and cannot be used directly to find and manipulate compressed audio frames. QuickTime has built-in support that allows programmers to act as if these sample tables pointed to uncompressed 1-byte audio samples.

2012-08-14 | Copyright © 2004, 2012 Apple Inc. All Rights Reserved.

Sound Sample Description (Version 2)

QuickTime 7 introduced a new version of the sound sample description, version 2, which extends QuickTime capabilities to include high resolution audio with another expansion of the sound sample description structure. In QuickTime 7, the sound and audio facilities are based on the Core Audio framework facilities and the Sound Manager has been deprecated. In this version of the sound sample description, the format field is set to `'lpcm'` for uncompressed data. For compressed data formats, the format field is set to the compression type code (normally `'mp4a'`) and the compression specifics and other features of QuickTime 7 are supplied by extensions.

The version field is set to 2 for this version of the sound sample description structure.

The sound sample description v2 structure adds the following new fields, appending to the v1 structure and renaming the four fields added in v1 to help ensure backwards compatibility with older applications. The version 2 fields are:

> **Important:** The values contained in these fields have different meanings for compressed and uncompressed audio. The meaning may not be easily deducible from the field name and require reference to the appropriate sound sample description extensions to understand fully.

Version
: A 16-bit integer that holds the sample description version (set to 2).

Revision level
: A 16-bit integer that must be set to 0.

Vendor
: A 32-bit integer that must be set to 0.

always3
: A 16-bit integer field that must be set to 3.

always16
: A 16-bit integer field that must be set to 16 (0x0010).

alwaysMinus2
: A 16-bit integer field that must be set to -2 (0xFFFE).

always0
: A 16-bit integer field that must be set to 0.

always65536
: A 32-bit integer field that must be set to 65536.

sizeOfStructOnly
: A 32-bit integer field providing the offset to sound sample description structure's extensions.

2012-08-14 | Copyright © 2004, 2012 Apple Inc. All Rights Reserved.

audioSampleRate
: A 64-bit floating point number representing the number of audio frames per second, for example: 44,100.0.

numAudioChannels
: A 32-bit integer field set to the number of audio channels; any channel assignment will be expressed in an extension.

always7F000000
: A 32-bit integer field that must be set to 0x7F000000.

constBitsPerChannel
: A 32-bit integer field which is set only if constant and only for uncompressed audio. For all other cases set to 0.

formatSpecificFlags
: A 32-bit integer field which carries LPCM flag values defined in "LPCM flag values" (page 183) below.

constBytesPerAudioPacket
: A 32-bit unsigned integer set to the number of bytes per packet only if this value is constant. For other cases set to 0.

constLPCMFramesPerAudioPacket
: A 32-bit unsigned integer set to the number of PCM frames per packet only if this value is constant. For other cases set to 0.

Some definitions for sound sample description version 2:

- LPCM Frame: one uncompressed sample in each of the channels (for instance, 44100Hz audio has 44100 **LPCM** frames per second, whether it is mono, stereo, 5.1, or other possible values). In other words, LPCM Frames divided by the `audioSampleRate` value is duration in seconds.
- Audio Packet: For compressed audio, an audio packet is the natural compressed access unit of that format. For uncompressed audio, an audio packet is simply one LPCM frame.
- Fields prefixed by "const": Note the three sound sample description v2 fields whose names start with "const". These fields are only nonzero if the value is a constant. A zero in each field implies that the value is variable. For example: AAC audio would have a zero in `constBytesPerAudioPacket` because AAC has variable sized audio packets. Codecs with variable duration audio packets set a zero in `constLPCMFramesPerAudioPacket`.

2012-08-14 | Copyright © 2004, 2012 Apple Inc. All Rights Reserved.

LPCM flag values

The `formatSpecificFlags` field carries flags significant to the layout and formatting of audio streams defined in the Core Audio underpinnings for sound sample description v2. These are enumerated in the Apple QuickTime/CoreAudioFormat.h interface file and are subject to a fuller interpretation in the context of the `AudioStreamBasicDescription` data type. See the CoreAudio, “Core Audio Framework Reference” in the OS X Developer Library.

```
enum
{
    kAudioFormatFlagIsFloat                     = (1 << 0),  // 0x1
    kAudioFormatFlagIsBigEndian                 = (1 << 1),  // 0x2
    kAudioFormatFlagIsSignedInteger             = (1 << 2),  // 0x4
    kAudioFormatFlagIsPacked                    = (1 << 3),  // 0x8
    kAudioFormatFlagIsAlignedHigh               = (1 << 4),  // 0x10
    kAudioFormatFlagIsNonInterleaved            = (1 << 5),  // 0x20
    kAudioFormatFlagIsNonMixable                = (1 << 6),  // 0x40
    kAudioFormatFlagsAreAllClear                = (1 << 31),

    kLinearPCMFormatFlagIsFloat                 = kAudioFormatFlagIsFloat,
    kLinearPCMFormatFlagIsBigEndian             = kAudioFormatFlagIsBigEndian,
    kLinearPCMFormatFlagIsSignedInteger         = kAudioFormatFlagIsSignedInteger,
    kLinearPCMFormatFlagIsPacked                = kAudioFormatFlagIsPacked,
    kLinearPCMFormatFlagIsAlignedHigh           = kAudioFormatFlagIsAlignedHigh,
    kLinearPCMFormatFlagIsNonInterleaved        = kAudioFormatFlagIsNonInterleaved,
    kLinearPCMFormatFlagIsNonMixable            = kAudioFormatFlagIsNonMixable,
    kLinearPCMFormatFlagsSampleFractionShift = 7,
    kLinearPCMFormatFlagsSampleFractionMask  = (0x3F <<
kLinearPCMFormatFlagsSampleFractionShift),
    kLinearPCMFormatFlagsAreAllClear            = kAudioFormatFlagsAreAllClear,

    kAppleLosslessFormatFlag_16BitSourceData = 1,
    kAppleLosslessFormatFlag_20BitSourceData = 2,
    kAppleLosslessFormatFlag_24BitSourceData = 3,
    kAppleLosslessFormatFlag_32BitSourceData = 4
};
```

Sound Sample Description Extensions

All extensions to the `SoundDescription` record are made using atoms. That means one or more atoms can be appended to the end of the `SoundDescription` record using the standard [size, type] mechanism used throughout the QuickTime movie architecture. Extensions were first added with sound sample description v1.

To illustrate this, for sound sample description v1, the extensions are added by following the last field of the struct with QuickTime atoms. The struct implementation looks like this:

```
struct SoundDescriptionV1 {
    // original fields
```

Copyright © 2004, 2012 Apple Inc. All Rights Reserved.

```
    SoundDescription    desc;
    // fixed compression ratio information
    unsigned long   samplesPerPacket;
    unsigned long   bytesPerPacket;
    unsigned long   bytesPerFrame;
    unsigned long   bytesPerSample;
    // optional, additional atom-based fields --
    // ([long size, long type, some data], repeat)
};
```

Version 2 of the sound sample description maintains the same mechanism for the addition of extensions. In the sound sample description v2 structure, the `sizeOfStructOnly` field value provides the offset to the extensions.

siSlopeAndIntercept Atom

Note: The siSlopeAndIntercept atom is deprecated in the QuickTime file format. The information that follows is intended to document existing content containing this atom and should not be used for new development.

The siSlopeAndIntercept atom contains `slope`, `intercept`, `minClip`, and `maxClip` parameters relevant to a decompressor component.

At runtime, the contents of the type `siSlopeAndIntercept` and `siDecompressorSettings` atoms are provided to the decompressor component through the standard `SetInfo` mechanism of the Sound Manager.

```
struct SoundSlopeAndInterceptRecord {
    Float64                 slope;
    Float64                 intercept;
    Float64                 minClip;
    Float64                 maxClip;
};
typedef struct SoundSlopeAndInterceptRecord SoundSlopeAndInterceptRecord;
```

2012-08-14 | Copyright © 2004, 2012 Apple Inc. All Rights Reserved.

siDecompressionParam Atom ('wave')

The `siDecompressionParam` atom provides the ability to store data specific to a given audio decompressor in the `SoundDescription` record. As example, some audio decompression algorithms, such as Microsoft's ADPCM, require a set of out-of-band values to configure the decompressor. These are stored in an atom of this type.

This atom contains other atoms with audio decompressor settings and is a required extension to the sound sample description for MPEG-4 audio. A `'wave'` chunk for `'mp4a'` typically contains (in order) at least a `'frma'` atom, an `'mp4a'` atom, an `'esds'` atom, and a "Terminator Atom (0x00000000)" atom.

The contents of other `siDecompressionParam` atoms are dependent on the audio decompressor.

Size
: An unsigned 32-bit integer holding the size of the decompression parameters atom.

Type
: An unsigned 32-bit field containing the four-character code `'wave'`.

Extension atoms
: Atoms containing the necessary out-of-band decompression parameters for the sound decompressor. For MPEG-4 audio (`'mp4a'`), this includes elementary stream descriptor (`'esds'`), format (`'frma'`), and terminator atoms.

Format Atom ('frma')

This atom shows the data format of the stored sound media.

Size
: An unsigned 32-bit integer holding the size of the format atom.

Type
: An unsigned 32-bit field containing the four-character code `'frma'`.

Data format
: The value of this field is copied from the data-format field of the sound sample description.

Terminator Atom (0x00000000)

This atom is present to indicate the end of the sound description. It contains no data, and has a type field of zero (0x00000000) instead of a four-character code.

Size
: An unsigned 32-bit integer holding the size of the decompression parameters atom (always set to 8).

2012-08-14 | Copyright © 2004, 2012 Apple Inc. All Rights Reserved.

Type

An unsigned 32-bit integer set to zero (0x00000000). This is a rare instance in which the type field is *not* a four-character ASCII code.

MPEG-4 Elementary Stream Descriptor Atom ('esds')

This atom is a required extension to the sound sample description for MPEG-4 audio. This atom contains an elementary stream descriptor, which is defined in ISO/IEC FDIS 14496.

Size

An unsigned 32-bit integer holding the size of the elementary stream descriptor atom.

Type

An unsigned 32-bit field containing the four-character code `'esds'`.

Version

An unsigned 32-bit field set to zero.

Elementary Stream Descriptor

An elementary stream descriptor for MPEG-4 audio, as defined in the MPEG-4 specification ISO/IEC 14496.

Audio Channel Layout Atom (‘chan’)

This atom is an optional extension to the sound sample description specifying audio channel layouts for sound media contained in QuickTime movies. It is a full atom followed by a big-endian audio channel layout structure as defined by Apple’s Core Audio framework. Audio channel layouts can be applied to both compressed and uncompressed sound formats.

Note: Audio channel layouts with more than two channels per track require implementation with a version 2 sound sample description.

Size

An unsigned 32-bit integer holding the size of the audio channel layout atom.

Type

An unsigned 32-bit field containing the four-character code `'chan'`

Version

A 1-byte specification of the version of the audio channel layout atom.

Flags

A 3-byte space for audio channel layout flags.

2012-08-14 | Copyright © 2004, 2012 Apple Inc. All Rights Reserved.

Audio channel layout

A big-endian `AudioChannelLayout` structure as defined in CoreAudioTypes.h. See the OS X Developer Library for CoreAudio framework details.

Subtitle Follows Track Reference Atom

Sound tracks can have a track reference of type `'folw'` (for "follows") to a single subtitle track from among the subtitle tracks in the same alternate group; this subtitle track should be considered the default to select if the sound track is selected. Use this only if compatibility between language tags is not possible for some reason making it impossible to otherwise select a default track. See "Preparing Sound and Subtitle Alternate Groups for Use with Apple Devices" (page 334) for related information.

Sound Sample Data

The format of data stored in sound samples is completely dependent on the type of the compressed data stored in the sound sample description. The following sections discuss some of the formats supported by QuickTime.

Uncompressed 8-Bit Sound

Eight-bit audio is stored in offset-binary encodings. If the data is in stereo, the left and right channels are interleaved.

Uncompressed 16-Bit Sound

Sixteen-bit audio may be stored in two's-complement encodings. If the data is in stereo, the left and right channels are interleaved.

IMA, uLaw, and aLaw

- IMA 4:1

  The IMA encoding scheme is based on a standard developed by the International Multimedia Association for pulse code modulation (PCM) audio compression. QuickTime uses a slight variation of the format to allow for random access. IMA is a 16-bit audio format which supports 4:1 compression. It is defined as follows:

  ```
  kIMACompression = FOUR_CHAR_CODE('ima4'), /*IMA 4:1*/
  ```

- uLaw 2:1 and aLaw 2:1

2012-08-14 | Copyright © 2004, 2012 Apple Inc. All Rights Reserved.

The uLaw (mu-law) encoding scheme is used on North American and Japanese phone systems, and is coming into use for voice data interchange, and in PBXs, voice-mail systems, and Internet talk radio (via MIME). In uLaw encoding, 14 bits of linear sample data are reduced to 8 bits of logarithmic data.

The aLaw encoding scheme is used in Europe and the rest of the world.

The kULawCompression and the kALawCompression formats are typically found in `.au` formats.

Floating-Point Formats

Both `kFloat32Format` and `kFloat64Format` are floating-point uncompressed formats. Depending upon codec-specific data associated with the sample description, the floating-point values may be in big-endian (network) or little-endian (Intel) byte order. This differs from the 16-bit formats, where there is a single format for each endian layout.

24- and 32-Bit Integer Formats

Both `k24BitFormat` and `k32BitFormat` are integer uncompressed formats. Depending upon codec-specific data associated with the sample description, the floating-point values may be in big-endian (network) or little-endian (Intel) byte order.

kMicrosoftADPCMFormat and kDVIIntelIMAFormat Sound Codecs

The `kMicrosoftADPCMFormat` and the `kDVIIntelIMAFormat` codec provide QuickTime interoperability with AVI and WAV files. The four-character codes used by Microsoft for their formats are numeric. To construct a QuickTime-supported codec format of this type, the Microsoft numeric ID is taken to generate a four-character code of the form `'msxx'` where *xx* takes on the numeric ID.

kDVAudioFormat Sound Codec

The DV audio sound codec, `kDVAudioFormat`, decodes audio found in a DV stream. Since a DV frame contains both video and audio, this codec knows how to skip video portions of the frame and only retrieve the audio portions. Likewise, the video codec skips the audio portions and renders only the image.

kQDesignCompression Sound Codec

The `kQDesignCompression` sound codec is the QDesign 1 (pre-QuickTime 4) format. Note that there is also a QDesign 2 format whose four-character code is `'QDM2'`.

2012-08-14 | Copyright © 2004, 2012 Apple Inc. All Rights Reserved.

MPEG-1 Layer 3 (MP3) Codecs

The QuickTime MPEG layer 3 (MP3) codecs come in two particular flavors, as shown in Table 4-7 (page 176). The first (`kMPEGLayer3Format`) is used exclusively in the constant bit rate (CBR) case (pre-QuickTime 4). The other (`kFullMPEGLay3Format`) is used in both the CBR and variable bit rate (VBR) cases. Note that they are the same codec underneath.

MPEG-4 Audio

MPEG-4 audio is stored as a sound track with data format `'mp4a'` and certain additions to the sound sample description and sound track atom. Specifically:

- The compression ID is set to -2 and redefined sample tables are used (see "Redefined Sample Tables" (page 180)).
- The sound sample description includes an `siDecompressionParam` atom (see "siDecompressionParam Atom ('wave')" (page 185)). The `siDecompressionParam` atom includes:
  - An MPEG-4 elementary stream descriptor extension atom (see "MPEG-4 Elementary Stream Descriptor Atom ('esds')" (page 186)).
  - The inclusion of a format atom is strongly recommended. See "Format Atom ('frma')" (page 185).
  - The last atom in the `siDecompressionParam` atom must be a terminator atom. See "Terminator Atom (0x00000000)" (page 185).
- Other atoms may be present as well; unknown atoms should be ignored.

The audio data is stored as an elementary MPEG-4 audio stream, as defined in ISO/IEC specification 14496-1.

Formats Not Currently in Use: MACE 3:1 and 6:1

These compression formats are obsolete: MACE 3:1 and 6:1.

These are 8-bit sound codec formats, defined as follows:

```
kMACE3Compression = FOUR_CHAR_CODE('MAC3'), /*MACE 3:1*/
kMACE6Compression = FOUR_CHAR_CODE('MAC6'), /*MACE 6:1*/
```

Timecode Media

Timecode media is used to store time code data in QuickTime movies. It has a media type of `'tmcd'`.

2012-08-14 | Copyright © 2004, 2012 Apple Inc. All Rights Reserved.

Timecode Sample Description

The timecode sample description contains information that defines how to interpret time code media data. This sample description is based on the standard sample description header, as described in "Sample Description Atoms" (page 99).

The data format field in the sample description is always set to `'tmcd'`.

The timecode media handler also adds some of its own fields to the sample description.

Reserved
: A 32-bit integer that is reserved for future use. Set this field to 0.

Flags
: A 32-bit integer containing flags that identify some timecode characteristics. The following flags are defined.

  Drop frame
  : Indicates whether the timecode is drop frame. Set it to 1 if the timecode is drop frame. This flag's value is 0x0001.

  24 hour max
  : Indicates whether the timecode wraps after 24 hours. Set it to 1 if the timecode wraps. This flag's value is 0x0002.

  Negative times OK
  : Indicates whether negative time values are allowed. Set it to 1 if the timecode supports negative values. This flag's value is 0x0004.

  Counter
  : Indicates whether the time value corresponds to a tape counter value. Set it to 1 if the timecode values are tape counter values. This flag's value is 0x0008.

Time scale
: A 32-bit integer that specifies the time scale for interpreting the frame duration field.

Frame duration
: A 32-bit integer that indicates how long each frame lasts in real time.

Number of frames
: An 8-bit integer that contains the number of frames per second for the timecode format. If the time is a counter, this is the number of frames for each counter tick.

Reserved
: A 24-bit quantity that must be set to 0.

2012-08-14 | Copyright © 2004, 2012 Apple Inc. All Rights Reserved.

Source reference
: A user data atom containing information about the source tape. The only currently used user data list entry is the `'name'` type. This entry contains a text item specifying the name of the source tape.

Timecode Media Information Atom

The timecode media also requires a media information atom. This atom contains information governing how the timecode text is displayed. This media information atom is stored in a base media information atom (see "Base Media Information Atoms" (page 89) for more information). The type of the timecode media information atom is `'tcmi'`.

The timecode media information atom contains the following fields:

Size
: A 32-bit integer that specifies the number of bytes in this time code media information atom.

Type
: A 32-bit integer that identifies the atom type; this field must be set to `'tcmi'`.

Version
: A 1-byte specification of the version of this timecode media information atom.

Flags
: A 3-byte space for timecode media information flags. Set this field to 0.

Text font
: A 16-bit integer that indicates the font to use. Set this field to 0 to use the system font. If the font name field contains a valid name, ignore this field.

Text face
: A 16-bit integer that indicates the font's style. Set this field to 0 for normal text. You can enable other style options by using one or more of the bit masks listed in Table 4-8 (page 191).

Table 4-8 Text face values

Value	Meaning
0x0001	Bold
0x0002	Italic
0x0004	Underline
0x0008	Outline
0x0010	Shadow

2012-08-14 | Copyright © 2004, 2012 Apple Inc. All Rights Reserved.

Value	Meaning
0x0020	Condense
0x0040	Extend

Text size
: A 16-bit integer that specifies the point size of the time code text.

Reserved
: A 16-bit integer that is reserved for use by Apple. Set this field to 0.

Text color
: A 48-bit RGB color value for the timecode text.

Background color
: A 48-bit RGB background color for the timecode text.

Font name
: A Pascal string specifying the name of the timecode text's font.

Timecode Sample Data

A timecode media sample is recorded as a 32-bit integer, interpreted based on the value of the Counter flag in the timecode sample description.

If the Counter flag is set to 1 in the timecode sample description, the sample data is an unsigned 32-bit integer. The timecode counter value is determined by dividing this unsigned 32-bit integer by the number of frames field in the timecode sample description.

If the Counter flag is set to 0 in the timecode sample description, the sample data format is a signed 32-bit integer and is used to calculate a timecode record, defined as follows.

Hours
: An 8-bit unsigned integer that indicates the starting number of hours.

Negative
: A 1-bit value indicating the time's sign. If bit is set to 1, the timecode record value is negative.

Minutes
: A 7-bit integer that contains the starting number of minutes.

Seconds
: An 8-bit unsigned integer indicating the starting number of seconds.

2012-08-14 | Copyright © 2004, 2012 Apple Inc. All Rights Reserved.

Frames

An 8-bit unsigned integer that specifies the starting number of frames. This field's value cannot exceed the value of the number of frames field in the timecode sample description.

Text Media

Text media is used to store text data in QuickTime movies. It has a media type of `'text'`.

Text Sample Description

The text sample description contains information that defines how to interpret text media data. This sample description is based on the standard sample description header, as described in "Sample Description Atoms" (page 99).

The data format field in the sample description is always set to `'text'`.

The text media handler also adds some of its own fields to the sample description.

Copyright © 2004, 2012 Apple Inc. All Rights Reserved.

Display flags

A 32-bit integer containing flags that describe how the text should be drawn. The following flags are defined.

Don't auto scale

Controls text scaling. If this flag is set to 1, the text media handler reflows the text instead of scaling when the track is scaled. This flag's value is 0x0002.

Use movie background color

Controls background color. If this flag is set to 1, the text media handler ignores the background color field in the text sample description and uses the movie's background color instead. This flag's value is 0x0008.

Scroll in

Controls text scrolling. If this flag is set to 1, the text media handler scrolls the text until the last of the text is in view. This flag's value is 0x0020.

Scroll out

Controls text scrolling. If this flag is set to 1, the text media handler scrolls the text until the last of the text is gone. This flag's value is 0x0040.

Horizontal scroll

Controls text scrolling. If this flag is set to 1, the text media handler scrolls the text horizontally; otherwise, it scrolls the text vertically. This flag's value is 0x0080.

Reverse scroll

Controls text scrolling. If this flag is set to 1, the text media handler scrolls down (if scrolling vertically) or backward (if scrolling horizontally; note that horizontal scrolling also depends upon text justification). This flag's value is 0x0100.

Continuous scroll

Controls text scrolling. If this flag is set to 1, the text media handler displays new samples by scrolling out the old ones. This flag's value is 0x0200.

Drop shadow

Controls drop shadow. If this flag is set to 1, the text media handler displays the text with a drop shadow. This flag's value is 0x1000.

Anti-alias

Controls anti-aliasing. If this flag is set to 1, the text media handler uses anti-aliasing when drawing text. This flag's value is 0x2000.

Key text

2012-08-14 | Copyright © 2004, 2012 Apple Inc. All Rights Reserved.

Controls background color. If this flag is set to 1, the text media handler does not display the background color, so that the text overlay background tracks. This flag's value is 0x4000.

Text justification
A 32-bit integer that indicates how the text should be aligned. Set this field to 0 for left-justified text, to 1 for centered text, and to –1 for right-justified text.

Background color
A 48-bit RGB color that specifies the text's background color.

Default text box
A 64-bit rectangle that specifies an area to receive text (top, left, bottom, right). Typically this field is set to all zeros.

Reserved
A 64-bit value that must be set to 0.

Font number
A 16-bit value that must be set to 0.

Font face
A 16-bit integer that indicates the font's style. Set this field to 0 for normal text. You can enable other style options by using one or more of the bit masks listed in Table 4-9 (page 195).

Table 4-9 Font face values

Value	Meaning
0x0001	Bold
0x0002	Italic
0x0004	Underline
0x0008	Outline
0x0010	Shadow
0x0020	Condense
0x0040	Extend

Reserved
An 8-bit value that must be set to 0.

Reserved
A 16-bit value that must be set to 0.

2012-08-14 | Copyright © 2004, 2012 Apple Inc. All Rights Reserved.

Foreground color
A 48-bit RGB color that specifies the text’s foreground color.

Text name
A Pascal string specifying the name of the font to use to display the text.

Text Media Information Atom

The text media also requires a text media information atom. This media information atom is stored in a base media information atom (`'minf'`) in the base media information header atom (`'gmhd'`) (see “Base Media Information Atoms” (page 89)). The type of the text media information atom is `'text'`.

The timecode media information atom contains the following fields:

Size
A 32-bit integer that specifies the number of bytes in this text media information atom.

Type
A 32-bit integer that identifies the atom type; this field must be set to `'text'`.

Matrix structure
A matrix structure associated with this text media. This should be the identity matrix. A matrix shows how to map points from one coordinate space into another. See “Matrices” (page 320) for a discussion of how display matrices are used in QuickTime and see Figure 2-3 (page 44) for an illustration of a matrix structure within an atom.

Text Sample Data

The format of the text data is a 16-bit length word followed by the actual text. The length word specifies the number of bytes of text, not including the length word itself. Following the text, there may be one or more atoms containing additional information for drawing and searching the text.

Table 4-10 (page 196) lists the currently defined text sample extensions.

Table 4-10 Text sample extensions

Text sample extension	Description
`'styl'`	Style information for the text. Allows you to override the default style in the sample description or to define more than one style for a sample. The data is a TextEdit style scrap.

2012-08-14 | Copyright © 2004, 2012 Apple Inc. All Rights Reserved.

Text sample extension	Description
'ftab'	Table of font names. Each table entry contains a font number (stored in a 16-bit integer) and a font name (stored in a Pascal string).This atom is required if the 'styl' atom is present.
'hlit'	Highlight information. The atom data consists of two 32-bit integers. The first contains the starting offset for the highlighted text, and the second has the ending offset. A highlight sample can be in a key frame or in a differenced frame. When it's used in a differenced frame, the sample should contain a zero-length piece of text.
'hclr'	Highlight color. This atom specifies the 48-bit RGB color to use for highlighting.
'drpo'	Drop shadow offset. When the display flags indicate drop shadow style, this atom can be used to override the default drop shadow placement. The data consists of two 16-bit integers. The first indicates the horizontal displacement of the drop shadow, in pixels; the second, the vertical displacement.
'drpt'	Drop shadow transparency. The data is a 16-bit integer between 0 and 256 indicating the degree of transparency of the drop shadow. A value of 256 makes the drop shadow completely opaque.
'imag'	Image font data. This atom contains two more atoms. An 'idat' atom contains compressed image data to be used to draw the text when the required fonts are not available. An 'idsc' atom contains a video sample description describing the format of the compressed image data.
'metr'	Image font highlighting. This atom contains metric information that governs highlighting when an 'imag' atom is used for drawing.

Hypertext and Wired Text

Hypertext is used as an action that takes you to a Web URL; like a Web URL, it appears blue and underlined. Hypertext is stored in a text track sample atom stream as type 'htxt'. The same mechanism is used to store wired actions linked to text strings. A text string can be wired to act as a hypertext link when clicked or to perform any defined QuickTime wired action when clicked. For details on wired actions, see "Wired Action Grammar" (page 225).

The data stored is a QTAtomContainer. The root atom of hypertext in this container is a wired-text atom of type 'wtxt'. This is the parent for all individual hypertext objects.

For each hypertext item, the parent atom is of type 'htxt'. This is the atom container atom type. Two children of this atom that define the offset of the hypertext in the text stream are:

2012-08-14 | Copyright © 2004, 2012 Apple Inc. All Rights Reserved.

```
kRangeStart         strt // unsigned long
kRangeEnd           end  // unsigned long
```

Child atoms of the parent atom are the events of type `kQTEventType` and the ID of the event type. The children of these event atoms follow the same format as other wired events.

```
kQTEventType, (kQTEventMouseClick, kQTEventMouseClickEnd,
                    kQTEventMouseClickEndTriggerButton,
                    kQTEventMouseEnter, kQTEventMouseExit)
...
kTextWiredObjectsAtomType, 1
    kHyperTextItemAtomType, 1..n
         kRangeStart, 1
            long
        kRangeEnd, 1
            long

    kAction     // The known range of track movie sprite actions
```

Closed Captioning Media

A closed caption media track contains text data used for closed captioning in QuickTime movies. It has a media type of `'clcp'`. Closed captions are used to display the audio portions of a movie as text. They transcribe dialog and indicate other sounds.

Other tracks can identify this track as being a related closed captioning track by using the `'clcp'` track reference to refer to this track.

Closed Captioning Sample Description

The closed captioning sample description contains information that defines how to interpret closed captioning media data. This sample description is based on the standard sample description header, as described in "Sample Description Atoms" (page 99), and adds no additional fields.

Copyright © 2004, 2012 Apple Inc. All Rights Reserved.

The data format field in the sample description is always set to `'c608'`.

Note: Closed caption tracks with data formats other than `'c608'` are not defined by this specification. Unrecognized data formats should be ignored.

Closed Captioning Sample Data

The format of the closed captioning sample data is a sequence of one or more atoms, one of which must be a 'cdat' atom. Unrecognized atoms should be ignored. For details about the content, refer to the specification *CEA-608-E, Line 21 Data Services, April, 2008*.

Size
: A 32-bit integer that specifies the number of bytes in this closed captioning media data atom.

Type
: A 32-bit integer that identifies the atom type; this field must be set to `'cdat'`.

Note: Apple reserves all atom types with lowercase letters and numbers.

Byte pair array
: An array of one or more byte pairs for data channel 1/field 1 (“CC1”) of a CEA-608 data stream, each byte pair corresponding to a video frame.

The durations of closed caption media samples can vary but should not be shorter than the number of byte pairs in the byte pair array. A closed caption media sample duration that is longer than the array length in video frames should treat additional durations as though null (0) byte pair bytes are received.

Note: The carriage of byte pairs for other elements of the source CEA-608-E frame data are not described here. If supported, other atom types and their content will be documented.

Subtitle Media

Subtitle media is used to store text data used for subtitles in QuickTime movies. It has a media type of `'sbtl'`. Subtitles provide written versions of audio or visual content, such as to offer alternate language translations or to supplement the content. Subtitles differ from closed captions in that subtitles are usually a translation of the sound track into a different language rather than a transcription of the sound track in the same language.

2012-08-14 | Copyright © 2004, 2012 Apple Inc. All Rights Reserved.

Subtitle Sample Description

The subtitle sample description contains information that defines how to interpret subtitle media data. This sample description is based on the standard sample description header, as described in "Sample Description Atoms" (page 99).

Note: Only one sample description is permitted in a `'sbtl'` track.

The data format field in the sample description is currently always set to `'tx3g'`. Unrecognized data formats should be ignored. The text media described here is based on the text box defined in the 3GPP Timed Text specification but provides a different track type and media handler designed specifically for subtitles.

The subtitle media handler adds some of its own fields to the sample description.

Display flags

A 32-bit integer containing flags that describe how the subtitle text should be drawn. The following flags are defined.

Vertical placement

Controls vertical placement of the subtitle text. If this flag is set, the subtitle media handler uses the top coordinate of the display bounds of the override `'tbox'` text box to determine the subtitle's vertical placement as described in "Subtitle Track Header Size and Placement" (page 205). Otherwise, the subtitle displays at the bottom of the video. This flag's value is 0x20000000.

Some samples are forced

Indicates whether any subtitle samples contain forced atoms. If this flag is set, at least one sample contains a forced (`'frcd'`) atom as described in "Subtitle Sample Data" (page 202). This flag's value is 0x40000000.

All samples are forced

If this flag is set, the subtitle media handler treats all samples as forced subtitles, regardless of the presence or absence of a `'frcd'` atom. This flag's value is 0x80000000. If this flag is set, the Some Samples Are Forced flag must also be set (making 0xC0000000).

Reserved

An 8-bit integer that must be set to 1.

Reserved

An 8-bit integer that must be set to -1 (negative one).

Reserved

A 32-bit integer that must be set to 0.

2012-08-14 | Copyright © 2004, 2012 Apple Inc. All Rights Reserved.

Default text box

A 64-bit rectangle that specifies an area to receive text (each 16 bits indicate top, left, bottom, and right, respectively) within the subtitle track. This rectangle must fill the track header dimensions exactly; that is, top is 0, left is 0, bottom is the height of the subtitle track header, and right is the width of the subtitle track header. See "Subtitle Track Header Size and Placement" (page 205).

Reserved

A 32-bit value that must be set to 0.

Font identifier

A 16-bit value that must be set to the same font identifier as in the font table (`'ftab'` extension).

Font face

An 8-bit integer that indicates the font's style. Set this field to 0 for normal text. You can enable other style options by using one or more of the bit masks listed in Table 4-11 (page 201).

Table 4-11 Font face values

Value	Meaning
0x0001	Bold
0x0002	Italic
0x0004	Underline

Font size

An 8-bit value that should always be 0.05 multiplied by the video track header height. For example, if the video track header is 720 points in height, this should be 36 (points). This size should be used in the default style record and in any per-sample style records. If a subtitle does not fit in the text box, the subtitle media handler may choose to shrink the font size so that the subtitle fits.

Foreground color

A 32-bit RGBA color that specifies the text's color, 8 bits each for red, green, blue, and alpha (transparency). For example, this would be (0,0,0,255) for opaque black or (255,255,255,255) for opaque white. Dark colors are not recommended, as the text could be placed onto a dark background.

Font table

An atom of type `'ftab'` that identifies the font to use to display the text. See "Font Table Atom" (page 201).

Font Table Atom

This atom specifies the font used to display the subtitle.

Copyright © 2004, 2012 Apple Inc. All Rights Reserved.

Size
: An unsigned 32-bit integer holding the size of the font table atom.

Type
: An unsigned 32-bit field containing the four-character code `'ftab'`.

Count
: An unsigned 16-bit integer specifying how many fonts are described in this table. This must be 1.

Font identifier
: An unsigned 16-bit integer that identifies the font. This can be any number to uniquely identify this font in this table, but it must match the font number specified in the subtitle sample description and in any per-sample style records (`'styl'`).

Font name length
: An unsigned 8-bit integer specifying the length of the font name in bytes.

Font name
: Must be either "Serif" or "Sans-Serif".

Subtitle Sample Data

Subtitle sample data consists of a 16-bit word that specifies the length (number of bytes) of the subtitle text, followed by the subtitle text and then by optional sample extensions. The subtitle text is Unicode text, encoded either as UTF-8 text or UTF-16 text beginning with a UTF-16 BYTE ORDER MARK ('\uFEFF') in big or little endian order. There is no null termination for the text.

Following the subtitle text, there may be one or more atoms containing additional information for selecting and drawing the subtitle.

Table 4-12 (page 203) lists the currently defined subtitle sample extensions.

2012-08-14 | Copyright © 2004, 2012 Apple Inc. All Rights Reserved.

Table 4-12 Subtitle sample extensions

Subtitle sample extension	Description
`'frcd'`	The presence of this atom indicates that the sample contains a forced subtitle. This extension has no data.<br>Forced subtitles are shown automatically when appropriate without any interaction from the user. If any sample contains a forced subtitle, the Some Samples Are Forced (0x40000000) flag must also be set in the display flags.<br>Consider an example where the primary language of the content is English, but the user has chosen to listen to a French dub of the audio. If a scene in the video displays something in English that is important to the plot or the content (such as a newspaper headline), a forced subtitle displays the content translated into French. In this case, the subtitle is linked ("forced") to the French language sound track.<br>If this atom is not present, the subtitle is typically simply a translation of the audio content, which a user can choose to display or hide.
`'styl'`	Style information for the subtitle. This atom allows you to override the default style in the sample description or to define more than one style within a sample. See "Subtitle Style Atom" (page 203).
`'tbox'`	Override of the default text box for this sample. Used only if the 0x20000000 display flag is set in the sample description and, in that case, only the top is considered. Even so, all fields should be set as though they are considered. See "Text Box atom" (page 204).
`'twrp'`	Text wrap. Set the one-byte payload to 0x00 for no wrapping or 0x01 for automatic soft wrapping.

Subtitle Style Atom

This extension specifies changes to the appearance of a subtitle. The style information in the subtitle sample description provides the default style for the subtitle text. This extension allows you to override the default style for different parts, or all, of the subtitle text.

Size
: An unsigned 32-bit integer holding the size of the subtitle style atom.

Type
: An unsigned 32-bit field containing the four-character code `'styl'`.

Entry count
: An unsigned 16-bit integer specifying how many subtitle text style records follow this entry count.

Copyright © 2004, 2012 Apple Inc. All Rights Reserved.

Subtitle text style record

One or more records that provide details about the subtitle's style. One record consists of the following fields.

Start character

A 16-bit value that is the offset of the first character that is to use the style specified in this record. Zero (0) is the first character in the subtitle.

End character

A 16-bit value that is the offset of the character that follows the last character to use this style.

Font identifier

A 16-bit value that must be set to the same font identifier as in the font table (`'ftab'` extension).

Font face

An 8-bit integer that indicates the font's style. Set this field to 0 for normal text. You can enable other style options by using one or more of the bit masks listed in Text.

Font size

An 8-bit value that specifies the font's size. See "Subtitle Sample Description" (page 200) for more information.

Foreground color

A 32-bit RGBA color that specifies the text's color. See "Subtitle Sample Description" (page 200) for more information.

Text Box atom

This optional extension defines a text box for a subtitle sample, to be used as described in Table 4-12 (page 203). If present, this overrides the default text box in the associated sample description. If the subtitle sample description's Display flags do not include the Vertical Placement flag (0x20000000), the Text Box atom should not be included in any sample of the subtitle track.

Size

An unsigned 32-bit integer holding the size of the subtitle style atom.

Type

An unsigned 32-bit field containing the four-character code `'tbox'`.

Text box

A 64-bit rectangle that specifies an area to receive text (each 16 bits indicate top, left, bottom, and right, respectively) within the subtitle track. This rectangle must fill the track width dimensions exactly. The top and bottom coordinates can vary because they are used to place and size the subtitle text vertically. The

Copyright © 2004, 2012 Apple Inc. All Rights Reserved.

top is used to place the text; the height is determined by the bottom minus the top. Neither the top nor the bottom should be outside the subtitle track dimensions. See "Subtitle Track Header Size and Placement" (page 205).

Subtitle Track Header Size and Placement

Individual subtitles can be placed only within the subtitle track's dimensions, adjusted by the subtitle track's matrix. This is expressed relative to the main video track, allowing subtitles to overlay the video. Typically, all subtitles are placed at the bottom of the video. Alternatively, subtitles can be placed at a different vertical location, which allows individual subtitles at the bottom or the top of the associated video. This section describes how this is controlled and how track and subtitle geometry is established.

The value of the track dimensions and track matrix differ depending upon the absence or presence of the Vertical Placement (0x20000000) flag in the subtitle sample description's display flags. When Vertical Placement is not set, subtitles are always placed at the bottom of the video. When Vertical Placement is set, the vertical position of subtitles can vary based upon the Text Box atom (`'tbox'`) in each sample.

In both cases, the subtitle track width must be the same as that of its associated main video (`'vide'`) track.

If the the Vertical Placement flag (0x20000000) display flag of the sample description is not set, the following should be true:

- The subtitle track's height should be 0.15 * the `'vide'` track header height. This allows room for two lines of subtitle text. For example, if the `'vide'` track header height is 720 pixels, then the `'sbtl'` track header height should be 108 (pixels).
- The subtitle track's vertical placement is determined by the track matrix, which should be a simple vertical translation matrix that shifts the subtitle down by 0.85 * the `'vide'` track header height. For a subtitle media handler that obeys the tx3g rules, this positions the subtitles atop the bottom 15 percent of the video. Media handlers may choose to shift the subtitles further down in some modes; for example, in a playback mode that displays black bars above and below content, the video could be shifted up and the subtitles moved down into the black area.
- Subtitle samples must not contain a text box sample data extension (`'tbox'`) because no control over vertical placement is allowed.

Alternatively, if the the Vertical Placement flag (0x20000000) display flag of the sample description is set, the following should be true:

- The height of the subtitle track should be the height of the video track header instead of 0.15 * the video track height. Because the subtitle track dimensions match the video track dimensions, subtitle text can be positioned at the bottom or top of the video, unlike when the Vertical Placement flag is not set.
- The track matrix should be the identity matrix.

2012-08-14 | Copyright © 2004, 2012 Apple Inc. All Rights Reserved.

- A subtitle's placement is determined by the top coordinate of one of two rectangles. If the override text box sample data extension ('tbox') is present, it is used. Otherwise, the default text box in the sample description is used. Some players will use the top coordinate to determine whether the subtitle is in the top half of the track dimensions and place the subtitle at the top of the video, otherwise placing it at the bottom of the video. Other players might use the top coordinate precisely, placing the subtitle at the specified vertical coordinate. As both playback environments are possible for a piece of content, it is recommend that a top coordinate of 0 be used for placing at the top and a top coordinate equal to the track height minus the subtitle height be used. In this way, if the content is played in either kind of player, its placement is predictable.

Referencing a Related Forced Subtitle Track

A subtitle track can contain a track reference of type `'forc'` to a paired subtitle track that contains only forced subtitles.

Pairing two subtitle tracks might be necessary if the timing of forced subtitle samples (see `'frcd'`) differs from the regular subtitle text, such as when a forced subtitle display would overlap in time with the display of the regular subtitle. If timings are the same, a single subtitle track should be used.

To pair two tracks, one subtitle track can contain any combination of forced and non-forced (regular) subtitle samples and the other track must contain only forced subtitles. The tracks must be in the same alternate group and be tagged with the same extended language tag and language code. The first, regular track then uses a track reference of type 'forc' to reference the second, forced-only track. (Mixing extended language tags or codes for the same language in the same alternate group is undefined.)

Note: The regular track in a pair provides a complete transcription of the audio as subtitle text. This allows a user to listen in one language but to read subtitled dialogue in another language.

See "Alternate Subtitle Tracks" (page 335) and "Track Reference Atoms" (page 73) for more information.

Music Media

Music media is used to store note-based audio data, such as MIDI data, in QuickTime movies. It has a media type of `'musi'`.

2012-08-14 | Copyright © 2004, 2012 Apple Inc. All Rights Reserved.

Music Sample Description

The music sample description uses the standard sample description header, as described in the section "Sample Description Atoms" (page 99).

The data format field in the sample description is always set to `'musi'`. The music media handler adds an additional 32-bit integer field to the sample description containing flags. Currently no flags are defined, and this field should be set to 0.

Following the flags field, there may be appended data in the QuickTime music format. This data consists of part-to-instrument mappings in the form of General events containing note requests. One note request event should be present for each part that will be used in the sample data.

Music Sample Data

The sample data for music samples consists entirely of data in the QuickTime music format. Typically, up to 30 seconds of notes are grouped into a single sample.

MPEG-1 Media

MPEG-1 media is used to store MPEG-1 video streams, MPEG-1, layer 2 audio streams, and multiplexed MPEG-1 audio and video streams in QuickTime movies. It has a media type of `'MPEG'`.

MPEG-1 Sample Description

The MPEG-1 sample description uses the standard sample description header, as described in "Sample Description Atoms" (page 99).

The data format field in the sample description is always set to `'MPEG'`. The MPEG-1 media handler adds no additional fields to the sample description.

Note: This data format is not used for MPEG-1, layer 3 audio, however (see "MPEG-1 Layer 3 (MP3) Codecs" (page 189)).

MPEG-1 Sample Data

Each sample in an MPEG-1 media is an entire MPEG-1 stream. This means that a single MPEG-1 sample may be several hundred megabytes in size. The MPEG-1 encoding used by QuickTime corresponds to the ISO standard, as described in ISO document CD 11172.

Copyright © 2004, 2012 Apple Inc. All Rights Reserved.

Sprite Media

> **Note:** Sprite media is deprecated in the QuickTime file format. The information that follows is intended to document existing content containing sprite media and should not be used for new development.

Sprite media is used to store character-based animation data in QuickTime movies. It has a media type of `'sprt'`.

Sprite Sample Description

The sprite sample description uses the standard sample description header, as described in "Sample Description Atoms" (page 99).

The data format field in the sample description is always set to `'sprt'`. The sprite media handler adds no additional fields to the sample description.

Sprite Sample Data

All sprite samples are stored in QT atom structures. The sprite media uses both key frames and differenced frames. The key frames contain all of the sprite's image data, and the initial settings for each of the sprite's properties.

A key frame always contains a shared data atom of type `'dflt'`. This atom contains data to be shared between the sprites, consisting mainly of image data and sample descriptions. The shared data atom contains a single sprite image container atom, with an atom type value of `'imct'` and an ID value of 1.

The sprite image container atom stores one or more sprite image atoms of type `'imag'`. Each sprite image atom contains an image sample description immediately followed by the sprite's compressed image data. The sprite image atoms should have ID numbers starting at 1 and counting consecutively upward.

The key frame also must contain definitions for each sprite in atoms of type `'sprt'`. Sprite atoms should have ID numbers start at 1 and count consecutively upward. Each sprite atom contains a list of properties. Table 4-13 (page 209) shows all currently defined sprite properties.

2012-08-14 | Copyright © 2004, 2012 Apple Inc. All Rights Reserved.

Table 4-13 Sprite properties

Property name	Value	Description
`kSpriteProperty-Matrix`	1	Describes the sprite's location and scaling within its sprite world or sprite track. By modifying a sprite's matrix, you can modify the sprite's location so that it appears to move in a smooth path on the screen or so that it jumps from one place to another. You can modify a sprite's size, so that it shrinks, grows, or stretches. Depending on which image compressor is used to create the sprite images, other transformations, such as rotation, may be supported as well. Translation-only matrices provide the best performance.
`kSpriteProperty-Visible`	4	Specifies whether or not the sprite is visible. To make a sprite visible, you set the sprite's visible property to `true`.
`kSpritePropertyLayer`	5	Contains a 16-bit integer value specifying the layer into which the sprite is to be drawn. Sprites with lower layer numbers appear in front of sprites with higher layer numbers. To designate a sprite as a background sprite, you should assign it the special layer number `kBackgroundSpriteLayerNum`.
`kSpriteProperty-GraphicsMode`	6	Specifies a graphics mode and blend color that indicates how to blend a sprite with any sprites behind it and with the background. To set a sprite's graphics mode, you call `SetSpriteProperty`, passing a pointer to a `ModifierTrackGraphicsModeRecord` structure.
`kSpriteProperty-ActionHandlingSprite-ID`	8	Specifies another sprite by ID that delegates QT events.
`kSpriteProperty-ImageIndex`	100	Contains the atom ID of the sprite's image atom.

The override sample differs from the key frame sample in two ways. First, the override sample does not contain a shared data atom. All shared data must appear in the key frame. Second, only those sprite properties that change need to be specified. If none of a sprite's properties change in a given frame, then the sprite does not need an atom in the differenced frame.

The override sample can be used in one of two ways: combined, as with video key frames, to construct the current frame; or the current frame can be derived by combining only the key frame and the current override sample.

2012-08-14 | Copyright © 2004, 2012 Apple Inc. All Rights Reserved.

Refer to the section "Sprite Track Media Format" (page 211) for information on how override samples are indicated in the file, using `kSpriteTrackPropertySampleFormat` and the default behavior of the `kKeyFrameAndSingleOverride` format.

Sprite Track Properties

Note: Sprite media is deprecated in the QuickTime file format. The information that follows is intended to document existing content containing sprite media and should not be used for new development.

In addition to defining properties for individual sprites, you can also define properties that apply to an entire sprite track. These properties may override default behavior or provide hints to the sprite media handler. The following sprite track properties are supported:

`kSpriteTrackPropertyBackgroundColor`
: Specifies a background color for the sprite track. The background color is used for any area that is not covered by regular sprites or background sprites. If you do not specify a background color, the sprite track uses black as the default background color.

`kSpriteTrackPropertyOffscreenBitDepth`
: Specifies a preferred bit depth for the sprite track's offscreen buffer. The allowable values are 8 and 16. To save memory, you should set the value of this property to the minimum depth needed. If you do not specify a bit depth, the sprite track allocates an offscreen buffer with the depth of the deepest intersecting monitor.

`kSpriteTrackPropertySampleFormat`
: Specifies the sample format for the sprite track. If you do not specify a sample format, the sprite track uses the default format, `kKeyFrameAndSingleOverride`.

To specify sprite track properties, you create a single QT atom container and add a leaf atom for each property you want to specify. To add the properties to a sprite track, you call the media handler function `SetMediaPropertyAtom`. To retrieve a sprite track's properties, you call the media handler function `GetMediaPropertyAtom`.

The sprite track properties and their corresponding data types are listed in Table 4-14 (page 211).

Copyright © 2004, 2012 Apple Inc. All Rights Reserved.

Table 4-14 Sprite track properties

Atom type	Atom ID	Leaf data type
kSpriteTrackPropertyBackgroundColor	1	RGBColor
kSpriteTrackPropertyOffscreenBitDepth	1	unsigned short
kSpriteTrackPropertySampleFormat	1	long
kSpriteTrackPropertyHasActions	1	Boolean
kSpriteTrackPropertyQTIdleEventsFrequency	1	UInt32
kSpriteTrackPropertyVisible	1	Boolean
kSpriteTrackPropertyScaleSpritesToScaleWorld	1	Boolean

Note: When pasting portions of two different tracks together, the Movie Toolbox checks to see that all sprite track properties match. If, in fact, they do match, the paste results in a single sprite track instead of two.

Sprite Track Media Format

Note: Sprite media is deprecated in the QuickTime file format. The information that follows is intended to document existing content containing sprite media and should not be used for new development.

The sprite track media format is hierarchical and based on QT atoms and atom containers. A sprite track is defined by one or more key frame samples, each followed by any number of override samples. A key frame sample and its subsequent override samples define a scene in the sprite track. A key frame sample is a QT atom container that contains atoms defining the sprites in the scene and their initial properties. The override samples are other QT atom containers that contain atoms that modify sprite properties, thereby animating the sprites in the scene. In addition to defining properties for individual sprites, you can also define properties that apply to an entire sprite track.

2012-08-14 | Copyright © 2004, 2012 Apple Inc. All Rights Reserved.

Figure 4-13 (page 212) shows the high-level structure of a sprite track key frame sample. Each atom in the atom container is represented by its atom type, atom ID, and, if it is a leaf atom, the type of its data.

Figure 4-13 A key frame sample atom container

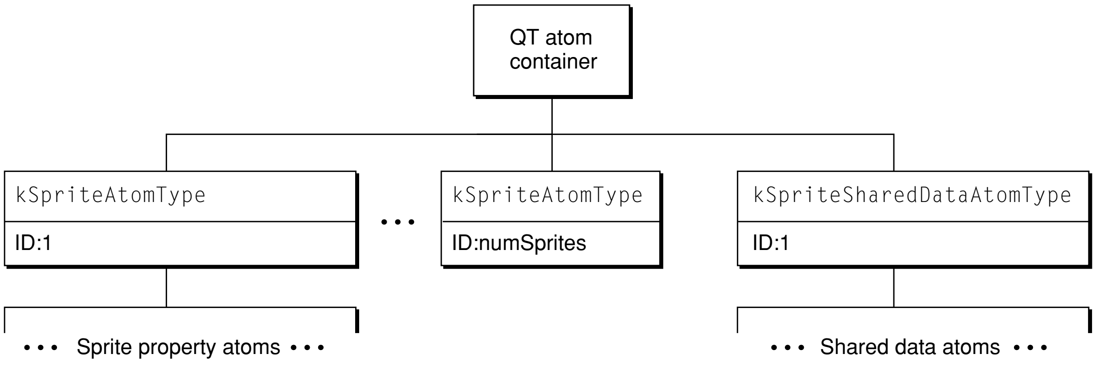

The QT atom container contains one child atom for each sprite in the key frame sample. Each sprite atom has a type of `kSpriteAtomType`. The sprite IDs are numbered from 1 to the number of sprites defined by the key frame sample (`numSprites`).

Each sprite atom contains leaf atoms that define the properties of the sprite, as shown in Figure 4-14 (page 212). For example, the `kSpritePropertyLayer` property defines a sprite's layer. Each sprite property atom has an atom type that corresponds to the property and an ID of 1.

Figure 4-14 Atoms that describe a sprite and its properties

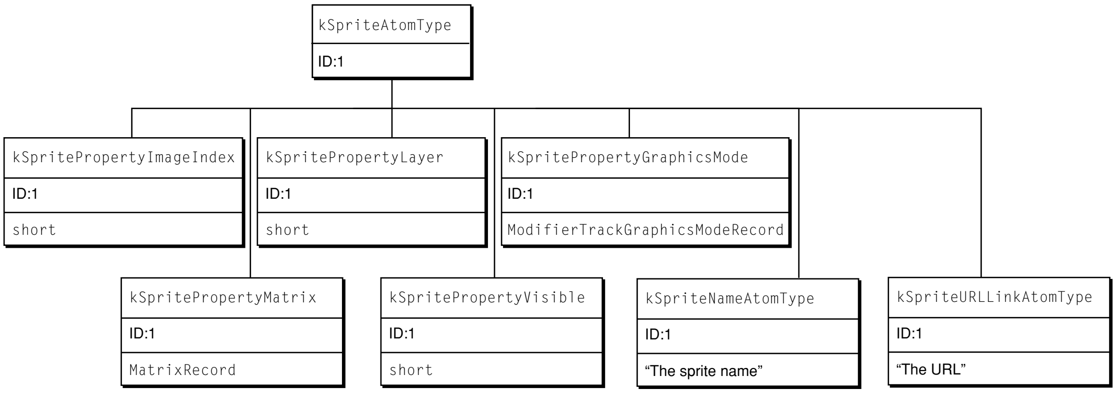

In addition to the sprite atoms, the QT atom container contains one atom of type `kSpriteSharedDataAtomType` with an ID of 1. The atoms contained by the shared data atom describe data that is shared by all sprites. The shared data atom contains one atom of type `kSpriteImagesContainerAtomType` with an ID of 1 (Figure 4-15 (page 213)).

2012-08-14 | Copyright © 2004, 2012 Apple Inc. All Rights Reserved.

The image container atom contains one atom of type `kImageAtomType` for each image in the key frame sample. The image atom IDs are numbered from 1 to the number of images (`numImages`). Each image atom contains a leaf atom that holds the image data (type `kSpriteImageDataAtomType`) and an optional leaf atom (type `kSpriteNameAtomType`) that holds the name of the image.

Figure 4-15 Atoms that describe sprite images

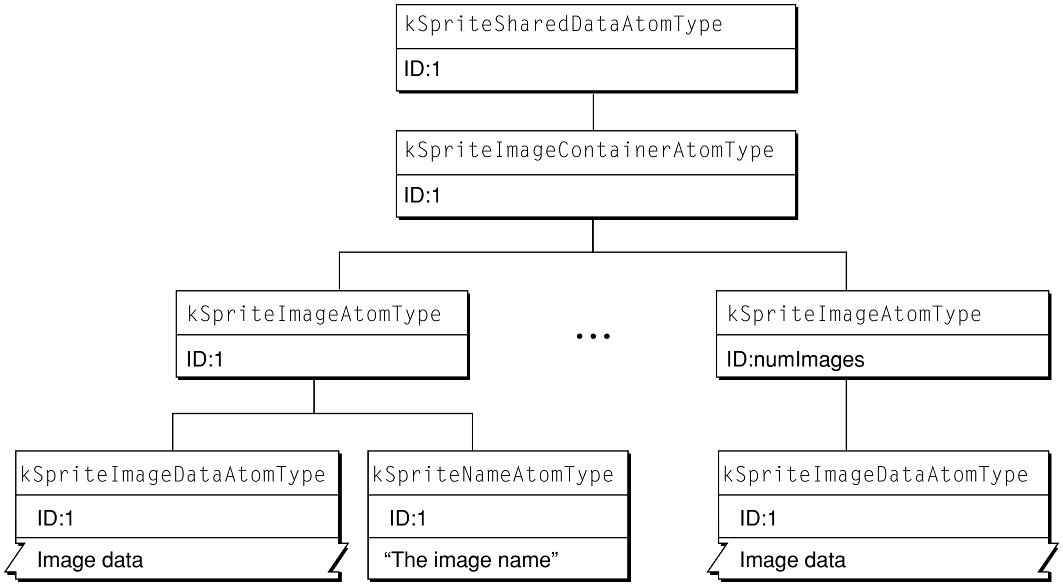

Sprite Media Format Atoms

The sprite track's sample format enables you to store the atoms necessary to describe action lists that are executed in response to QuickTime events. "QT Atom Container Description Key" (page 221) defines a grammar for constructing valid action sprite samples, which may include complex expressions.

Both key frame samples and override samples support the sprite action atoms. Override samples override actions at the QuickTime event level. In effect, what you do by overriding is to completely replace one event handler and all its actions with another. The sprite track's `kSpriteTrackPropertySampleFormat` property has no effect on how actions are performed. The behavior is similar to the default `kKeyFrameAndSingleOverride` format where, if in a given override sample there is no handler for the event, the key frame's handler is used, if there is one.

Sprite Media Format Extensions

This section describes some of the atom types and IDs used to extend the sprite track's media format, thus enabling action sprite capabilities.

A complete description of the grammar for sprite media handler samples, including action sprite extensions, is included in the section "Sprite Media Handler Track Properties QT Atom Container Format" (page 222).

Copyright © 2004, 2012 Apple Inc. All Rights Reserved.

> **Important:** Some sprite track property atoms were added in QuickTime 4. In particular, you must set the `kSpriteTrackPropertyHasActions` track property in order for your sprite actions to be executed.

Sprite Track Property Atoms

The following constants represent atom types for sprite track properties. These atoms are applied to the whole track, not just to a single sample.

Constant Descriptions

`kSpriteTrackPropertyHasActions`

You must add an atom of this type with its leaf data set to `true` if you want the movie controller to execute the actions in your sprite track's media. The atom's leaf data is of type `Boolean`. The default value is `false`, so it is very important to add an atom of this type if you want interactivity to take place.

`kSpriteTrackPropertyQTIdleEventsFrequency`

You must add an atom of this type if you want the sprites in your sprite track to receive `kQTEventIdle` QuickTime events. The atom's leaf data is of type `UInt32`. The value is the minimum number of ticks that must pass before the next `QTIdle` event is sent. Each tick is 1/60th of one second. To specify "Idle as fast as possible," set the value to 0. The default value is `kNoQTIdleEvents`, which means don't send any idle events.

It is possible that for small idle event frequencies, the movie will not be able to keep up, in which case idle events will be sent as fast as possible.

Since sending idle events takes up some time, it is best to specify the largest frequency that produces the results that you desire, or `kNoQTIdleEvents` if you do not need them.

`kSpriteTrackPropertyVisible`

You can cause the entire sprite track to be invisible by setting the value of this `Boolean` property to `false`. This is useful for using a sprite track as a hidden button track—for example, placing an invisible sprite track over a video track would allow the characters in the video to be clickable. The default value is visible (`true`).

`kSpriteTrackPropertyScaleSpritesToScaleWorld`

You can cause each sprite to be rescaled when the sprite track is resized by setting the value of this `Boolean` property to `true`. Setting this property can improve the drawing performance and quality of a scaled sprite track. This is particularly useful for sprite images compressed with codecs that are resolution-independent, such as the Curve codec. The default value for this property is `false`.

2012-08-14 | Copyright © 2004, 2012 Apple Inc. All Rights Reserved.

Sprite Media Atom and Data Types

The following constants represent atom types for sprite media:

```
enum {
    kSpriteAtomType                    = 'sprt',
    kSpriteImagesContainerAtomType     = 'imct',
    kSpriteImageAtomType               = 'imag',
    kSpriteImageDataAtomType           = 'imda',
    kSpriteImageDataRefAtomType        = 'imre',
    kSpriteImageDataRefTypeAtomType    = 'imrt',
    kSpriteImageGroupIDAtomType        = 'imgr',
    kSpriteImageRegistrationAtomType   = 'imrg',
    kSpriteImageDefaultImageIndexAtomType ='defi',
    kSpriteSharedDataAtomType          = 'dflt',
    kSpriteNameAtomType                = 'name',
    kSpriteImageNameAtomType           = 'name',
    kSpriteUsesImageIDsAtomType        = 'uses',
    kSpriteBehaviorsAtomType           = 'beha',
    kSpriteImageBehaviorAtomType       = 'imag',
    kSpriteCursorBehaviorAtomType      = 'crsr',
    kSpriteStatusStringsBehaviorAtomType = 'sstr',
    kSpriteVariablesContainerAtomType    = 'vars',
    kSpriteStringVariableAtomType        = 'strv',
    kSpriteFloatingPointVariableAtomType = 'flov'
    kSpriteSharedDataAtomType          = 'dflt',
    kSpriteURLLinkAtomType             = 'url '
    kSpritePropertyMatrix              = 1
    kSpritePropertyVisible             = 4
    kSpritePropertyLayer               = 5
    kSpritePropertyGraphicsMode        = 6
    kSpritePropertyImageIndex          = 100
    kSpritePropertyBackgroundColor     = 101
    kSpritePropertyOffscreenBitDepth   = 102
    kSpritePropertySampleFormat        = 103
};
```

Copyright © 2004, 2012 Apple Inc. All Rights Reserved.

Constant Descriptions

`kSpriteAtomType`
: The atom is a parent atom that describes a sprite. It contains atoms that describe properties of the sprite. Optionally, it may also include an atom of type `kSpriteNameAtomType` that defines the name of the sprite.

`kSpriteImagesContainerAtomType`
: The atom is a parent atom that contains atoms of type `kSpriteImageAtomType`.

`kSpriteImageAtomType`
: The atom is a parent atom that contains an atom of type `kSpriteImageDataAtomType`. Optionally, it may also include an atom of type `kSpriteNameAtomType` that defines the name of the image.

`kSpriteImageDataAtomType`
: The atom is a leaf atom that contains image data.

`kSpriteSharedDataAtomType`
: The atom is a parent atom that contains shared sprite data, such as an atom container of type `kSpriteImagesContainerAtomType`.

`kSpriteNameAtomType`
: The atom is a leaf atom that contains the name of a sprite or an image. The leaf data is composed of one or more ASCII characters.

`kSpritePropertyImageIndex`
: A leaf atom containing the image index property which is of type `short`. This atom is a child atom of `kSpriteAtom`.

`kSpritePropertyLayer`
: A leaf atom containing the layer property which is of type `short`. This atom is a child atom of `kSpriteAtom`.

`kSpritePropertyMatrix`
: A leaf atom containing the matrix property which is of type `MatrixRecord`. This atom is a child atom of `kSpriteAtom`.

`kSpritePropertyVisible`
: A leaf atom containing the visible property which is of type `short`. This atom is a child atom of `kSpriteAtom`.

`kSpritePropertyGraphicsMode`
: A leaf atom containing the graphics mode property which is of type `ModifyerTrackGraphicsModeRecord`. This atom is a child atom of `kSpriteAtom`.

2012-08-14 | Copyright © 2004, 2012 Apple Inc. All Rights Reserved.

`kSpritePropertyBackgroundColor`
: A leaf atom containing the background color property which is of type `RGBColor`. This atom is used in a sprite track's `MediaPropertyAtom` atom container.

`kSpritePropertyOffscreenBitDepth`
: A leaf atom containing the preferred offscreen bit depth which is of type `short`. This atom is used in a sprite track's `MediaPropertyAtom` atom container.

`kSpritePropertySampleFormat`
: A leaf atom containing the sample format property, which is of type `short`. This atom is used in a sprite track's `MediaPropertyAtom` atom container.

`kSpriteImageRegistrationAtomType`
: Sprite images have a default registration point of 0, 0. To specify a different point, add an atom of type `kSpriteImageRegistrationAtomType` as a child atom of the `kSpriteImageAtomType` and set its leaf data to a `FixedPoint` value with the desired registration point.

`kSpriteImageGroupIDAtomType`
: You must assign group IDs to sets of equivalent images in your key frame sample. For example, if the sample contains ten images where the first two images are equivalent, and the last eight images are equivalent, then you could assign a group ID of 1000 to the first two images, and a group ID of 1001 to the last eight images. This divides the images in the sample into two sets. The actual ID does not matter, it just needs to be a unique positive integer.

: Each image in a sprite media key frame sample is assigned to a group. Add an atom of type `kSpriteImageGroupIDAtomType` as a child of the `kSpriteImageAtomType` atom and set its leaf data to a long containing the group ID.

> **Important:** You must assign group IDs to your sprite sample if you want a sprite to display images with non-equivalent image descriptions (i.e., images with different dimensions).

For each of the following atom types (added to QuickTime 4)—except `kSpriteBehaviorsAtomType`—you fill in the structure `QTSpriteButtonBehaviorStruct`, which contains a value for each of the four states.

`kSpriteBehaviorsAtomType`
: This is the parent atom of `kSpriteImageBehaviorAtomType`, `kSpriteCursorBehaviorAtomType`, and `kSpriteStatusStringsBehaviorAtomType`.

`kSpriteImageBehaviorAtomType`
: Specifies the `imageIndex`.

`kSpriteCursorBehaviorAtomType`
: Specifies the `cursorID`.

2012-08-14 | Copyright © 2004, 2012 Apple Inc. All Rights Reserved.

`kSpriteStatusStringsBehaviorAtomType`
: Specifies an ID of a string variable contained in a sprite track to display in the status area of the browser.

Note: All sprite media—specifically the leaf data in the QT atom containers for sample and sprite track properties—should be written in big-endian format.

`kSpriteUsesImageIDsAtomType`
: This atom allows a sprite to specify which images it uses—in other words, the subset of images that its `imageIndex` property can refer to.

  You add an atom of type `kSpriteUsesImageIDsAtomType` as a child of a `kSpriteAtomType` atom, setting its leaf data to an array of QT atom IDs. This array contains the IDs of the images used, not the indices.

Although QuickTime does not currently use this atom internally, tools that edit sprite media can use the information provided to optimize certain operations, such as cut, copy, and paste.

`kSpriteImageRegistrationAtomType`
: Sprite images have a default registration point of 0, 0. To specify a different point, you add an atom of type `kSpriteImageRegistrationAtomType` as a child atom of the `kSpriteImageAtomType` and set its leaf data to a `FixedPoint` value with the desired registration point.

`kSpriteImageGroupIDAtomType`
: You must assign group IDs to sets of equivalent images in your key frame sample. For example, if the sample contains ten images where the first two images are equivalent, and the last eight images are equivalent, then you could assign a group ID of 1000 to the first two images, and a group ID of 1001 to the last eight images. This divides the images in the sample into two sets. The actual ID does not matter; it just needs to be a unique positive integer.

  Each image in a sprite media key frame sample is assigned to a group. You add an atom of type `kSpriteImageGroupIDAtomType` as a child of the `kSpriteImageAtomType` atom and set its leaf data to a long containing the group ID.

  Important: You must assign group IDs to your sprite sample if you want a sprite to display images with non-equivalent image descriptions (that is, images with different dimensions).

You use the following atom types, which were added to QuickTime 4, to specify that an image is referenced and how to access it.

2012-08-14 | Copyright © 2004, 2012 Apple Inc. All Rights Reserved.

`kSpriteImageDataRefAtomType`
Add this atom as a child of the `kSpriteImageAtomType` atom instead of a `kSpriteImageDataAtomType`. Its ID should be 1. Its data should contain the data reference (similar to the `dataRef` parameter of `GetDataHandler`).

`kSpriteImageDataRefTypeAtomType`
Add this atom as a child of the `kSpriteImageAtomType` atom. Its ID should be 1. Its data should contain the data reference type (similar to the `dataRefType` parameter of `GetDataHandler`).

`kSpriteImageDefaultImageIndexAtomType`
You may optionally add this atom as a child of the `kSpriteImageAtomType` atom. Its ID should be 1. Its data should contain a `short`, which specifies an image index of a traditional image to use while waiting for the referenced image to load.

The following constants represent formats of a sprite track. The value of the constant indicates how override samples in a sprite track should be interpreted. You set a sprite track's format by creating a `kSpriteTrackPropertySampleFormat` atom.

```
enum {
    kKeyFrameAndSingleOverride      = 1L << 1,
    kKeyFrameAndAllOverrides        = 1L << 2
};
```

Constant Descriptions

`kKeyFrameAndSingleOverride`
The current state of the sprite track is defined by the most recent key frame sample and the current override sample. This is the default format.

`kKeyFrameAndAllOverrides`
The current state of the sprite track is defined by the most recent key frame sample and all subsequent override samples up to and including the current override sample.

2012-08-14 | Copyright © 2004, 2012 Apple Inc. All Rights Reserved.

Sprite Button Behaviors

Note: Sprite media is deprecated in the QuickTime file format. The information that follows is intended to document existing content containing sprite media and should not be used for new development.

In QuickTime 4 and later, sprites in a sprite track can specify simple button behaviors. These behaviors can control the sprite's image, the system cursor, and the status message displayed in a Web browser. They also provide a shortcut for a common set of actions that may result in more efficient QuickTime movies.

Button behaviors can be added to a sprite. These behaviors are intended to make the common task of creating buttons in a sprite track easy—you basically just fill in a template.

Three types of behaviors are available; you may choose one or more behaviors. Each change a type of property associated with a button and are triggered by the mouse states `notOverNotPressed`, `overNotPressed`, `overPressed`, and `notOverPressed`. The three properties changed are:

- The sprite's `imageIndex`value
- The ID of a cursor to be displayed
- The ID of a status string variable displayed in the URL status area of a Web browser.

Setting a property's value to –1 means don't change it.

Note: The cursor is automatically set back to the default system cursor when leaving a sprite.

The sprite track handles letting one sprite act as an active button at a time.

The behaviors are added at the beginning of the sprite's list of actions, so they may be overridden by actions if desired.

To use the behaviors, you fill in the new atoms as follows, using the description key specified in "QT Atom Container Description Key" (page 221):

```
kSpriteAtomType
    <kSpriteBehaviorsAtomType>, 1

        <kSpriteImageBehaviorAtomType>
            [QTSpriteButtonBehaviorStruct]
```

2012-08-14 | Copyright © 2004, 2012 Apple Inc. All Rights Reserved.

```
        <kSpriteCursorBehaviorAtomType>
            [QTSpriteButtonBehaviorStruct]
        <kSpriteStatusStringsBehaviorAtomType>
            [QTSpriteButtonBehaviorStruct]
```

QT Atom Container Description Key

Because QT atom container–based data structures are widely used in QuickTime, a description key is presented here. Its usage is illustrated in the following sections, "Sprite Media Handler Track Properties QT Atom Container Format" (page 222) and "Sprite Media Handler Sample QT Atom Container Formats" (page 223).

```
[(QTAtomFormatName)] =
    atomType_1, id, index
        data
    atomType_n, id, index
        data
```

The atoms may be required or optional:

```
 // optional atom
 // required atom
<atomType>
atomType
```

The atom ID may be a number if it is required to be a constant, or it may be a list of valid atom IDs, indicating that multiple atoms of this type are allowed.

```
3               // one atom with id of 3
(1..3)          // three atoms with id's of 1, 2, and 3
(1, 5, 7)       // three atoms with id's of 1, 5, and 7
(anyUniqueIDs)  // multiple atoms each with a unique id
```

The atom index may be a 1 if only one atom of this type is allowed, or it may be a range from 1 to some constant or variable.

Copyright © 2004, 2012 Apple Inc. All Rights Reserved.

```
1               // one atom of this type is allowed, index is always  1
(1..3)          // three atoms with indexes 1, 2, and 3
(1..numAtoms)   // numAtoms atoms with indexes of 1 to numAtoms
```

The data may be leaf data in which its data type is listed inside of brackets [], or it may be a nested tree of atoms.

```
[theDataType]   // leaf data of type theDataType
childAtoms      // a nested tree of atoms
```

Nested `QTAtom` format definitions [(AtomFormatName)] may appear in a definition.

Sprite Media Handler Track Properties QT Atom Container Format

Note: Sprite media is deprecated in the QuickTime file format. The information that follows is intended to document existing content containing sprite media and should not be used for new development.

```
[(SpriteTrackProperties)]
    <kSpriteTrackPropertyBackgroundColor, 1, 1>
        [RGBColor]
    <kSpriteTrackPropertyOffscreenBitDepth, 1, 1>
        [short]
    <kSpriteTrackPropertySampleFormat, 1, 1>
        [long]
    <kSpriteTrackPropertyScaleSpritesToScaleWorld, 1, 1>
        [Boolean]
    <kSpriteTrackPropertyHasActions, 1, 1>
        [Boolean]
    <kSpriteTrackPropertyVisible, 1, 1>
        [Boolean]
    <kSpriteTrackPropertyQTIdleEventsFrequency, 1, 1>
        [UInt32]
```

2012-08-14 | Copyright © 2004, 2012 Apple Inc. All Rights Reserved.

Sprite Media Handler Sample QT Atom Container Formats

Note: Sprite media is deprecated in the QuickTime file format. The information that follows is intended to document existing content containing sprite media and should not be used for new development.

```
[(SpriteKeySample)] =
    [(SpritePropertyAtoms)]
    [(SpriteImageAtoms)]



[(SpriteOverrideSample)] =
    [(SpritePropertyAtoms)]



[(SpriteImageAtoms)]
    kSpriteSharedDataAtomType, 1, 1
        <kSpriteVariablesContainerAtomType>, 1
            <kSpriteStringVariableAtomType>, (1..n) ID is  SpriteTrack
                            Variable ID to be set
                                                [CString]
            <kSpriteFloatingPointVariableAtomType>, (1..n)  ID is
                            SpriteTrack Variable ID to be set
                                                [float]

        kSpriteImagesContainerAtomType, 1, 1
            kSpriteImageAtomType, theImageID, (1 .. numImages)
                kSpriteImageDataAtomType, 1, 1
                    [ImageData is ImageDescriptionHandle prepended  to
                                                            image  data]
                <kSpriteImageRegistrationAtomType, 1, 1>
                    [FixedPoint]
                <kSpriteImageNameAtomType, 1, 1>
                    [pString]
                <kSpriteImageGroupIDAtomType, 1, 1>
```

Copyright © 2004, 2012 Apple Inc. All Rights Reserved.

```
                    [long]


[(SpritePropertyAtoms)]
    <kQTEventFrameLoaded>, 1, 1
        [(ActionListAtoms)]
        <kCommentAtomType>, (anyUniqueIDs), (1..numComments)
            [CString]

    kSpriteAtomType, theSpriteID, (1 .. numSprites)
        <kSpritePropertyMatrix, 1, 1>
            [MatrixRecord]
        <kSpritePropertyVisible, 1, 1>
            [short]
        <kSpritePropertyLayer, 1, 1>
            [short]
        <kSpritePropertyImageIndex, 1, 1>
            [short]
        <kSpritePropertyGraphicsMode, 1, 1>
            [ModifierTrackGraphicsModeRecord]

        <kSpriteUsesImageIDsAtomType, 1, 1>
            [array of QTAtomID's, one per image used]

        <kSpriteBehaviorsAtomType>, 1

        <kSpriteImageBehaviorAtomType>
            [QTSpriteButtonBehaviorStruct]
        <kSpriteCursorBehaviorAtomType>
            [QTSpriteButtonBehaviorStruct]
        <kSpriteStatusStringsBehaviorAtomType>
            [QTSpriteButtonBehaviorStruct]

        <[(SpriteActionAtoms)]>
```

Copyright © 2004, 2012 Apple Inc. All Rights Reserved.

```
[(SpriteActionAtoms)] =
    kQTEventType, theQTEventType, (1 .. numEventTypes)
            [(ActionListAtoms)] //see the next section Wired Action
                                //Grammar for a description
            <kCommentAtomType>, (anyUniqueIDs), (1..numComments)
                [CString]
```

Wired Action Grammar

The wired action grammar shown in this section allows QT event handlers to be expressed in a QuickTime movie. The sprite, text, VR, 3D, and Flash media handlers all support the embedding of QT event handlers in their media samples.

```
[(ActionListAtoms)] =
    kAction, (anyUniqueIDs), (1..numActions)
        kWhichAction    1, 1
            [long whichActionConstant]
        <kActionParameter>  (anyUniqueIDs), (1..numParameters)
            [(parameterData)] ( whichActionConstant, paramIndex  )
    // either leaf data or child atoms
        <kActionFlags>  parameterID,  (1..numParamsWithFlags)
            [long actionFlags]
        <kActionParameterMinValue>  parameterID,  (1.. numParamsWithMin)
            [data depends on param type]
        <kActionParameterMaxValue>  parameterID,  (1.. numParamsWithMax)
            [data depends on param type]
        [(ActionTargetAtoms)]

        <kCommentAtomType>, (anyUniqueIDs), (1..numComments)
            [CString]

[(ActionTargetAtoms)] =
    <kActionTarget>
```

Copyright © 2004, 2012 Apple Inc. All Rights Reserved.

```
        <kTargetMovie>
            [no data]
    <kTargetChildMovieTrackName>
        <PString childMovieTrackName>
    <kTargetChildMovieTrack>
        [IDlong childMovieTrackID]
    <kTargetChildMovieTrackIndex>
            [long childMovieTrackIndex]
        <kTargetChildMovieMovieName>
            [PString childMovieName]
        <kTargetChildMovieMovieID>
            [long childMovieID]
        <kTargetTrackName>
            [PString trackName]
        <kTargetTrackType>
            [OSType trackType]
        <kTargetTrackIndex>
            [long trackIndex]
            OR
            [(kExpressionAtoms)]
        <kTargetTrackID>
            [long trackID]
            OR
            [(kExpressionAtoms)]
        <kTargetSpriteName>
            [PString spriteName]
        <kTargetSpriteIndex>
            [short spriteIndex]
            OR
            [(kExpressionAtoms)]
        <kTargetSpriteID>
            [QTAtomID spriteIID]
            OR
            [(kExpressionAtoms)]
```

Copyright © 2004, 2012 Apple Inc. All Rights Reserved.

```
        <kTargetQD3DNamedObjectName>
            [CString objectName]

[(kExpressionAtoms)] =
    kExpressionContainerAtomType, 1, 1
        <kOperatorAtomType, theOperatorType, 1>
            kOperandAtomType, (anyUniqueIDs), (1..numOperands)
                [(OperandAtoms)]
        OR
        <kOperandAtomType, 1, 1>
            [(OperandAtoms)]
[(ActionTargetAtoms)] =
    <kActionTarget>


        <kTargetMovieName>
            [Pstring MovieName]
        OR
        <kTargetMovieID>
            [long MovieID]
            OR
            [(kExpressionAtoms)]


[(OperandAtoms)] =
    <kOperandExpression> 1, 1
        [(kExpressionAtoms)]        // allows for recursion
    OR
    <kOperandConstant> 1, 1
        [ float theConstant ]
    OR
    <kOperandSpriteTrackVariable> 1, 1
        [(ActionTargetAtoms)]
        kActionParameter, 1, 1
            [QTAtomID spriteVariableID]
    OR
    <kOperandKeyIsDown> 1, 1
```

Copyright © 2004, 2012 Apple Inc. All Rights Reserved.

```
        kActionParameter, 1, 1
            [UInt16 modifierKeys]
        kActionParameter, 2, 2
            [UInt8 asciiCharCode]
    OR
    <kOperandRandom> 1, 1
        kActionParameter, 1, 1
            [short minimum]
        kActionParameter, 2, 2
            [short maximum]
    OR
    <any other operand atom type>
        [(ActionTargetAtoms)]
```

The format for parameter data depends on the action and parameter index.

In most cases, the `kActionParameter` atom is a leaf atom containing data; for a few parameters, it contains child atoms.

`whichAction` corresponds to the action type that is specified by the leaf data of a `kWhichAction` atom.

`paramIndex` is the index of the parameter's `kActionParameter` atom.

```
[(parameterData)] ( whichAction, paramIndex ) =
{
    kActionMovieSetVolume:
        param1:     short volume

    kActionMovieSetRate
        param1:     Fixed rate

    kActionMovieSetLoopingFlags
        param1:     long loopingFlags

    kActionMovieGoToTime
        param1:     TimeValue time
```

Copyright © 2004, 2012 Apple Inc. All Rights Reserved.

```
kActionMovieGoToTimeByName
    param1:     Str255 timeName

kActionMovieGoToBeginning
    no params

kActionMovieGoToEnd
    no params

kActionMovieStepForward
    no params

kActionMovieStepBackward
    no params

kActionMovieSetSelection
    param1:     TimeValue startTime
    param2:     TimeValue endTime

kActionMovieSetSelectionByName
    param1:     Str255 startTimeName
    param2:     Str255 endTimeName

kActionMoviePlaySelection
    param1:     Boolean selectionOnly

kActionMovieSetLanguage
    param1:     long language

kActionMovieChanged
    no params

kActionTrackSetVolume
    param1:     short volume
```

Copyright © 2004, 2012 Apple Inc. All Rights Reserved.

```
kActionTrackSetBalance
    param1:     short balance

kActionTrackSetEnabled
    param1:     Boolean enabled

kActionTrackSetMatrix
    param1:     MatrixRecord matrix

kActionTrackSetLayer
    param1:     short layer

kActionTrackSetClip
    param1:     RgnHandle clip

kActionSpriteSetMatrix
    param1:     MatrixRecord matrix

kActionSpriteSetImageIndex
    parm1:      short imageIndex

kActionSpriteSetVisible
    param1:     short visible

kActionSpriteSetLayer
    param1:     short layer

kActionSpriteSetGraphicsMode
    param1:     ModifierTrackGraphicsModeRecord graphicsMode

kActionSpritePassMouseToCodec
    no params
```

2012-08-14 | Copyright © 2004, 2012 Apple Inc. All Rights Reserved.

```
kActionSpriteClickOnCodec
    param1:     Point localLoc

kActionSpriteTranslate
    param1:     Fixed x
    param2:     Fixed y
    param3:     Boolean isRelative

kActionSpriteScale
    param1:     Fixed xScale
    param2:     Fixed yScale

kActionSpriteRotate
    param1:     Fixed degrees

kActionSpriteStretch
    param1:     Fixed p1x
    param2:     Fixed p1y
    param3:     Fixed p2x
    param4:     Fixed p2y
    param5:     Fixed p3x
    param6:     Fixed p3y
    param7:     Fixed p4x
    param8:     Fixed p4y



kActionQTVRSetPanAngle
    param1:     float panAngle

kActionQTVRSetTiltAngle
    param1:     float tileAngle

kActionQTVRSetFieldOfView
    param1:     float fieldOfView
```

Copyright © 2004, 2012 Apple Inc. All Rights Reserved.

```
kActionQTVRShowDefaultView
    no params

kActionQTVRGoToNodeID
    param1:     UInt32 nodeID

kActionMusicPlayNote
    param1:     long sampleDescIndex
    param2:     long partNumber
    param3:     long delay
    param4:     long pitch
    param5:     long velocity
    param6:     long duration

kActionMusicSetController
    param1:     long sampleDescIndex
    param2:     long partNumber
    param3:     long delay
    param4:     long controller
    param5:     long value

kActionCase
    param1:     [(CaseStatementActionAtoms)]

kActionWhile
    param1:     [(WhileStatementActionAtoms)]

kActionGoToURL
    param1:     CString urlLink

kActionSendQTEventToSprite
    param1:     [(SpriteTargetAtoms)]
    param2:     QTEventRecord theEvent
```

2012-08-14 | Copyright © 2004, 2012 Apple Inc. All Rights Reserved.

```
    kActionDebugStr
        param1:     Str255 theMessageString

    kActionPushCurrentTime
        no params

    kActionPushCurrentTimeWithLabel
        param1:     Str255 theLabel

    kActionPopAndGotoTopTime
        no params

    kActionPopAndGotoLabeledTime
        param1:     Str255 theLabel

    kActionSpriteTrackSetVariable
        param1:     QTAtomID variableID
        param2:     float value

    kActionApplicationNumberAndString
        param1:     long aNumber
        param2:     Str255 aString
}
```

Both [(CaseStatementActionAtoms)] and [(WhileStatementActionAtoms)] are child atoms of a `kActionParameter` 1, 1 atom.

```
[(CaseStatementActionAtoms)] =
    kConditionalAtomType, (anyUniqueIDs), (1..numCases)
        [(kExpressionAtoms)]
        kActionListAtomType 1, 1
            [(ActionListAtoms)] // may contain nested conditional  actions

[(WhileStatementActionAtoms)] =
    kConditionalAtomType, 1, 1
```

```
        [(kExpressionAtoms)]
        kActionListAtomType 1, 1
            [(ActionListAtoms)] // may contain nested conditional  actions
```

Flash Media

Note: Flash media is deprecated in the QuickTime file format. The information that follows is intended to document existing content containing flash media and should not be used for new development.

Flash is a vector-based graphics and animation technology designed for the Internet. As an authoring tool, Flash lets content authors and developers create a wide range of interactive vector animations. The files exported by this tool are called SWF (pronounced "swiff") files. SWF files are commonly played back using Macromedia's ShockWave plug-in. In an effort to establish Flash as an industrywide standard, Macromedia has published the SWF File Format and made the specification publicly available on its website at `http://www.macromedia.com/software/flash/open/spec/`.

The Flash media handler, introduced in QuickTime 4, allows a Macromedia Flash SWF 3.0 file to be treated as a track within a QuickTime movie. Thus, QuickTime 4 extends the SWF file format, enabling the execution of any of its wired actions. See "Adding Wired Actions To a Flash Track" (page 347) for an example of how to add wired actions.

Because a QuickTime movie may contain any number of tracks, multiple SWF tracks may be added to the same movie. The Flash media handler also provides support for an optimized case using the alpha channel graphics mode, which allows a Flash track to be composited cleanly over other tracks.

QuickTime supports all Flash actions except for the Flash load movie action. For example, when a Flash track in a QuickTime movie contains an action that goes to a particular Flash frame, QuickTime converts this to a wired action that goes to the QuickTime movie time in the corresponding Flash frame.

2012-08-14 | Copyright © 2004, 2012 Apple Inc. All Rights Reserved.

Note: As a time-based media playback format, QuickTime may drop frames when necessary to maintain its schedule. As a consequence, frames of a SWF file may be dropped during playback. If this is not satisfactory for your application, you can set the playback mode of the movie to Play All Frames, which will emulate the playback mode of ShockWave. QuickTime's SWF file importer sets the Play All Frames mode automatically when adding a SWF file to an empty movie.

QuickTime support for Flash 3.0 also includes the `DoFSCommand` mechanism. This allows JavaScript routines with a specific function prototype to be invoked with parameters passed from the Flash track. Refer to Macromedia's Flash 3 documentation for more details on how to author a `.SWF` 3.0 file with a Flash `FSCommand`.

Tween Media

Note: Tween media is deprecated in the QuickTime file format. The information that follows is intended to document existing content containing tween media and should not be used for new development.

Tween media is used to store pairs of values to be interpolated between in QuickTime movies. These interpolated values modify the playback of other media types by using track references and track input maps. For example, a tween media could generate gradually changing volume levels to cause a sound track to fade out. It has a media type of `'twen'`.

Every tween operation is based on a collection of one or more values from which a range of output values can be algorithmically derived. Each tween is assigned a time duration, and an output value can be generated for any time value within the duration. In the simplest kind of tween operation, a pair of values is provided as input and values between the two values are generated as output.

A tween track is a special track in a movie that is used exclusively as a modifier track. The data it contains, known as tween data, is used to generate values that modify the playback of other tracks, usually by interpolating values. The tween media handler sends these values to other media handlers; it never presents data.

Tween Sample Description

The tween sample description uses the standard sample description header, as described in "Sample Table Atoms" (page 96).

The data format field in the sample description is always set to `'twen'`. The tween media handler adds no additional fields to the sample description.

2012-08-14 | Copyright © 2004, 2012 Apple Inc. All Rights Reserved.

Tween Sample Data

Tween sample data is stored in QT atom structures.

At the root level, there are one or more tween entry atoms; these atoms have an atom type value of `'twen'`. Each tween entry atom completely describes one interpolation operation. These atoms should be consecutively numbered starting at 1, using the atom ID field.

Each tween entry atom contains several more atoms that describe how to perform the interpolation. The atom ID field in each of these atoms must be set to 1.

Tween start atom (atom type is `'twst'`).
: This atom specifies the time at which the interpolation is to start. The time is expressed in the media's time coordinate system. If this atom is not present, the starting offset is assumed to be 0.

Tween duration atom (atom type is `'twdu'`).
: This atom specifies how long the interpolation is to last. The time is expressed in the media's time coordinate system. If this atom is not present, the duration is assumed to be the length of the sample.

Tween data atom (atom type is `'twdt'`).
: This atom contains the actual values for the interpolation. The contents depend on the value of the tween type atom.

Tween type atom (atom type is `'twnt'`).
: Describes the type of interpolation to perform.

Table 4-15 (page 236) shows all currently defined tween types. All tween types are currently supported using linear interpolation.

Table 4-15 Tween type values

Tween type	Value	Tween data
16-bit integer	1	Two 16-bit integers.
32-bit integer	2	Two 32-bit integers.
32-bit fixed-point	3	Two 32-bit fixed-point numbers.
Point: two 16-bit integers	4	Two points.
Rectangle: four 16-bit integers	5	Two rectangles.
QuickDraw region	6	Two rectangles and a region. The tween entry atom must contain a `'qdrg'` atom with an atom ID value of 1. The region is transformed through the resulting matrices.

Tween type	Value	Tween data
Matrix	7	Two matrices.
RGB color: three 16-bit integers	8	Two RGB colors.
Graphics mode with RGB color	9	Two graphics modes with RGB color. Only the RGB color is interpolated. The graphics modes must be the same.

Each tween type is distinguished from other types by these characteristics:

- Input values or structures of a particular type
- A particular number of input values or structures (most often one or two)
- Output values or structures of a particular type
- A particular algorithm used to derive the output values

Tween operations for each tween type are performed by a tween component that is specific to that type or, for a number of tween types that are native to QuickTime, by QuickTime itself. Movies and applications that use tweening do not need to specify the tween component to use; QuickTime identifies a tween type by its tween type identifier and automatically routes its data to the correct tween component or to QuickTime.

When a movie contains a tween track, the tween media handler invokes the necessary component (or built-in QuickTime code) for tween operations and delivers the results to another media handler. The receiving media handler can then use the values it receives to modify its playback. For example, the data in a tween track can be used to alter the volume of a sound track.

Tweening can also be used outside of movies by applications or other software that can use the values it generates.

Tween Type Categories

Each of the tween types supported by QuickTime belongs to one of these categories:

- Numeric tween types, which have pairs of numeric values, such as long integers, as input. For these types, linear interpolation is used to generate output values.
- QuickDraw tween types, most of which have pairs of QuickDraw structures, such as points or rectangles, as input. For these types, one or more structure elements are interpolated, such as the `h` and `v` values for points, and each element that is interpolated is interpolated separately from others.
- 3D tween types, which have a QuickDraw 3D structure such as `TQ3Matrix4x4` or `TQ3RotateAboutAxisTransformData` as input. For these types, a specific 3D transformation is performed on the data to generate output.

Copyright © 2004, 2012 Apple Inc. All Rights Reserved.

- The polygon tween type, which takes three four-sided polygons as input. One polygon (such as the bounds for a sprite or track) is transformed, and the two others specify the start and end of the range of polygons into which the tween operation maps it. You can use the output (a `MatrixRecord` data structure) to map the source polygon into any intermediate polygon. The intermediate polygon is interpolated from the start and end polygons for each particular time in the tween duration.
- Path tween types, which have as input a QuickTime vector data stream for a path. Four of the path tween types also have as input a percentage of path's length; for these types, either a point on the path or a data structure is returned. Two other path tween types treat the path as a function: one returns the `y` value of the point on the path with a given `x` value, and the other returns the `x` value of the point on the path with a given `y` value.
- The list tween type, which has as input a QT atom container that contains leaf atoms of a specified atom type. For this tween type category, the duration of the tween operation is divided by the number of leaf atoms of the specified type. For time points within the first time division, the data for the first leaf atom is returned; for the second time division, the data for the second leaf atom is returned; and so on. The resulting tween operation proceeds in discrete steps (one step for each leaf atom), instead of the relatively continuous tweening produced by other tween type categories.

Tween QT Atom Container

The characteristics of a tween are specified by the atoms in a tween QT atom container.

A tween QT atom container can contain the atoms described in the following sections.

General Tween Atoms

`kTweenEntry`

Specifies a tween atom, which can be either a single tween atom, a tween atom in a tween sequence, or an interpolation tween atom.

Its parent is the tween QT atom container (which you specify with the constant `kParentAtomIsContainer`).

The index of a `kTweenEntry` atom specifies when it was added to the QT atom container`r`; the first added has the index 1, the second 2, and so on. The ID of a `kTweenEntry` atom can be any ID that is unique among the `kTweenEntry` atoms contained in the same QuickTime atom container.

This atom is a parent atom. It must contain the following child atoms:

- A `kTweenType` atom that specifies the tween type.
- One or more `kTweenData` atoms that contain the data for the tween atom. Each `kTweenData` atom can contain different data to be processed by the tween component, and a tween component can process data from only one `kTweenData` atom a time. For example, an application can use a

2012-08-14 | Copyright © 2004, 2012 Apple Inc. All Rights Reserved.

list tween to animate sprites. The `kTweenEntry` atom for the tween atom could contain three sets of animation data, one for moving the sprite from left to right, one for moving the sprite from right to left, and one for moving the sprite from top to bottom. In this case, the `kTweenEntry` atom for the tween atom would contain three `kTweenData` atoms, one for each data set. The application specifies the desired data set by specifying the ID of the `kTweenData` atom to use.

A `kTweenEntry` atom can contain any of the following optional child atoms:

- A `kTweenStartOffset` atom that specifies a time interval, beginning at the start of the tween media sample, after which the tween operation begins. If this atom is not included, the tween operation begins at the start of the tween media sample.
- A `kTweenDuration` atom that specifies the duration of the tween operation. If this atom is not included, the duration of the tween operation is the duration of the media sample that contains it.

If a `kTweenEntry` atom specifies a path tween, it can contain the following optional child atom:

- A `kTweenFlags` atom containing flags that control the tween operation. If this atom is not included, no flags are set.

Note that interpolation tween tracks are tween tracks that modify other tween tracks. The output of an interpolation tween track must be a time value, and the time values generated are used in place of the input time values of the tween track being modified.

If a `kTweenEntry` atom specifies an interpolation tween track, it must contain the following child atoms:

- A `kTweenInterpolationID` atom for each `kTweenData` atom to be interpolated. The ID of each `kTweenInterpolationID` atom must match the ID of the `kTweenData` atom to be interpolated. The data for a `kTweenInterpolationID` atom specifies a `kTweenEntry` atom that contains the interpolation tween track to use for the `kTweenData` atom.

If this atom specifies an interpolation tween track, it can contain either of the following optional child atoms:

- A `kTweenOutputMin` atom that specifies the minimum output value of the interpolation tween atom. The value of this atom is used only if there is also a `kTweenOutputMax` atom with the same parent. If this atom is not included and there is a `kTweenOutputMax` atom with the same parent, the tween component uses `0` as the minimum value when scaling output values of the interpolation tween track.
- A `kTweenOutputMax` atom that specifies the maximum output value of the interpolation tween atom. If this atom is not included, the tween component does not scale the output values of the interpolation tween track.

2012-08-14 | Copyright © 2004, 2012 Apple Inc. All Rights Reserved.

`kTweenStartOffset`

For a tween atom in a tween track of a QuickTime movie, specifies a time offset from the start of the tween media sample to the start of the tween atom. The time units are the units used for the tween track.

Its parent atom is a `kTweenEntry` atom.

A `kTweenEntry` atom can contain only one `kTweenStartOffset` atom. The ID of this atom is always 1. The index of this atom is always 1.

This atom is a leaf atom. The data type of its data is `TimeValue`.

This atom is optional. If it is not included, the tween operation begins at the start of the tween media sample.

`kTweenDuration`

Specifies the duration of a tween operation. When a QuickTime movie includes a tween track, the time units for the duration are those of the tween track. If a tween component is used outside of a movie, the application using the tween data determines how the duration value and values returned by the component are interpreted.

Its parent atom is a `kTweenEntry` atom.

A `kTweenEntry` atom can contain only one `kTweenDuration` atom. The ID of this atom is always 1. The index of this atom is always 1.

This atom is a leaf atom. The data type of its data is `TimeValue`.

This atom is optional. If it is not included, the duration of the tween operation is the duration of the media sample that contains it.

2012-08-14 | Copyright © 2004, 2012 Apple Inc. All Rights Reserved.

`kTweenData`

Contains data for a tween atom.

Its parent atom is a `kTweenEntry` atom.

A `kTweenEntry` atom can contain any number of `kTweenData` atoms.

The index of a `kTweenData` atom specifies when it was added to the `kTweenEntry` atom; the first added has the index 1, the second 2, and so on. The ID of a `kTweenData` atom can be any ID that is unique among the `kTweenData` atoms contained in the same `kTweenEntry` atom.

At least one `kTweenData` atom is required in a `kTweenEntry` atom.

For single tween atoms, a `kTweenData` atom is a leaf atom. It can contain data of any type.

For polygon tween atoms, a `kTweenData` atom is a leaf atom. The data type of its data is `Fixed[27]`, which specifies three polygons.

For path tweens, a `kTweenData` atom is a leaf atom. The data type of its data is `Handle`, which contains a QuickTime vector.

In interpolation tween atoms, a `kTweenData` atom is a leaf atom. It can contain data of any type. An interpolation tween atom can be any tween atoms other than a list tween atom that returns a time value.

In list tween atoms, a `kTweenData` atom is a parent atom that must contain the following child atoms:

- A `kListElementType` atom that specifies the atom type of the elements of the tween atom.
- One or more leaf atoms of the type specified by the `kListElementType` atom. The data for each atom is the result of a list tween operation.

`kNameAtom`

Specifies the name of a tween atom. The name, which is optional, is not used by tween components, but it can be used by applications or other software.

Its parent atom is a `kTweenEntry` atom.

A `kTweenEntry` atom can contain only one `kNameAtom` atom. The ID of this atom is always 1. The index of this atom is always 1.

This atom is a leaf atom. Its data type is `String`.

This atom is optional. If it is not included, the tween atom does not have a name.

2012-08-14 | Copyright © 2004, 2012 Apple Inc. All Rights Reserved.

`kTweenType`

Specifies the tween type (the data type of the data for the tween operation).

Its parent atom is a `kTweenEntry` atom.

A `kTweenEntry` atom can contain only one `kTweenType` atom. The ID of this atom is always 1. The index of this atom is always 1.

This atom is a leaf atom. The data type of its data is `OSType`.

This atom is required.

Path Tween Atoms

`kTweenFlags`

Contains flags that control the tween operation. One flag that controls path tween atoms is defined:

- The `kTweenReturnDelta` flag applies only to path tween atoms (tweens of type `kTweenTypePathToFixedPoint`, `kTweenTypePathToMatrixTranslation`, `kTweenTypePathToMatrixTranslationAndRotation`, `kTweenTypePathXtoY`, or `kTweenTypePathYtoX`). If the flag is set, the tween component returns the change in value from the last time it was invoked. If the flag is not set, or if the tween component has not previously been invoked, the tween component returns the normal result for the tween atom.

  Its parent atom is a `kTweenEntry` atom.

  A `kTweenEntry` atom can contain only one `kTweenFlags` atom. The ID of this atom is always 1. The index of this atom is always 1.

  This atom is a leaf atom. The data type of its data is `Long`.

  This atom is optional. If it is not included, no flags are set.

`kInitialRotationAtom`

Specifies an initial angle of rotation for a path tween atom of type `kTweenTypePathToMatrixRotation`, `kTweenTypePathToMatrixTranslation`, or `kTweenTypePathToMatrixTranslationAndRotation`.

Its parent atom is a `kTweenEntry` atom.

A `kTweenEntry` atom can contain only one `kInitialRotationAtom` atom. The ID of this atom is always 1. The index of this atom is always 1.

This atom is a leaf atom. Its data type is `Fixed`.

This atom is optional. If it is not included, no initial rotation of the tween atom is performed.

Copyright © 2004, 2012 Apple Inc. All Rights Reserved.

List Tween Atoms

kListElementType

Specifies the atom type of the elements in a list tween atom.

Its parent atom is a kTweenData atom.

A kTweenEntry atom can contain only one kListElementType atom. The ID of this atom is always 1. The index of this atom is always 1.

This atom is a leaf atom. Its data type is QTAtomType.

This atom is required in the kTweenData atom for a list tween atom.

3D Tween Atoms

kTween3dInitialCondition

Specifies an initial transform for a 3D tween atom whose tween type is one of the following: kTweenType3dCameraData, kTweenType3dMatrix, kTweenType3dQuaternion, kTweenType3dRotate, kTweenType3dRotateAboutAxis, kTweenType3dRotateAboutAxis, kTweenType3dRotateAboutPoint, kTweenType3dRotateAboutVector, kTweenType3dScale, or kTweenType3dTranslate.

Its parent atom is a kTweenEntry atom.

A kTweenEntry atom can contain only one kTween3dInitialCondition atom. The ID of this atom is always 1. The index of this atom is always 1.

This atom is a leaf atom. The data type of its data is one of the values listed in Table 4-16 (page 243).

Table 4-16 Tween types

Tween Type	Data Type
kTweenType3dCameraData	TQ3CameraData
kTweenType3dMatrix	TQ3Matrix4x4
kTweenType3dQuaternion	TQ3Quaternion
kTweenType3dRotate	TQ3RotateTransformData
kTweenType3dRotateAboutAxis	TQ3RotateAboutAxisTransformData
kTweenType3dRotateAboutPoint	TQ3RotateAboutPointTransformData
kTweenType3dRotateAboutVector	TQ3PlaneEquation
kTweenType3dScale	TQ3Vector3D

2012-08-14 | Copyright © 2004, 2012 Apple Inc. All Rights Reserved.

Tween Type	Data Type
kTweenType3dTranslate	TQ3Vector3D

This atom is optional. For each tween type, the default value is the data structure that specifies an identity transform, that is, a transform that does not alter the 3D data.

Interpolation Tween Atoms

`kTweenOutputMax`

Specifies the maximum output value of an interpolation tween atom. If a `kTweenOutputMax` atom is included for an interpolation tween, output values for the tween atom are scaled to be within the minimum and maximum values. The minimum value is either the value of the `kTweenOutputMin` atom or, if there is no `kTweenOutputMin` atom, 0. For example, if an interpolation tween atom has values between 0 and 4, and it has `kTweenOutputMin` and `kTweenOutputMax` atoms with values 1 and 2, respectively, a value of 0 (the minimum value before scaling) is scaled to 1 (the minimum specified by the `kTweenOutputMin` atom), a value of 4 (the maximum value before scaling) is scaled to 2 (the maximum specified by the `kTweenOutputMax` atom), and a value of 3 (three-quarters of the way between the maximum and minimum values before scaling) is scaled to 1.75 (three-quarters of the way between the values of the `kTweenOutputMin` and `kTweenOutputMax` atoms).

Its parent atom is a `kTweenEntry` atom.

A `kTweenEntry` atom can contain only one `kTweenOutputMax` atom. The ID of this atom is always 1. The index of this atom is always 1.

This atom is a leaf atom. The data type of its data is `Fixed`.

This atom is optional. If it is not included, QuickTime does not scale interpolation tween values.

`kTweenOutputMin`

Specifies the minimum output value of an interpolation tween atom. If both `kTweenOutputMin` and `kTweenOutputMax` atoms are included for an interpolation tween atom, output values for the tween atom are scaled to be within the minimum and maximum values. For example, if an interpolation tween atom has values between 0 and 4, and it has `kTweenOutputMin` and `kTweenOutputMax` atoms with values 1 and 2, respectively, a value of 0 (the minimum value before scaling) is scaled to 1 (the minimum specified by the `kTweenOutputMin` atom), a value of 4 (the maximum value before scaling) is scaled to 2 (the maximum specified by the `kTweenOutputMax` atom), and a value of 3 (three-quarters of the way

2012-08-14 | Copyright © 2004, 2012 Apple Inc. All Rights Reserved.

between the maximum and minimum values before scaling) is scaled to 1.75 (three-quarters of the way between the values of the kTweenOutputMin and kTweenOutputMax atoms).

If a kTweenOutputMin atom is included but a kTweenOutputMax atom is not, QuickTime does not scale interpolation tween values.

Its parent atom is a kTweenEntry atom.

A kTweenEntry atom can contain only one kTweenOutputMin atom. The ID of this atom is always 1. The index of this atom is always 1.

This atom is a leaf atom. The data type of its data is Fixed.

This atom is optional. If it is not included but a kTweenOutputMax atom is, the tween component uses 0 as the minimum value for scaling values of an interpolation tween atom.

kTweenInterpolationID

Specifies an interpolation tween atom to use for a specified kTweenData atom. There can be any number of kTweenInterpolationID atoms for a tween atom, one for each kTweenData atom to be interpolated.

Its parent atom is a kTweenEntry atom.

The index of a kTweenInterpolationID atom specifies when it was added to the kTweenEntry atom; the first added has the index 1, the second 2, and so on. The ID of a kTweenInterpolationID atom must match the atom ID of the kTweenData atom to be interpolated, and be unique among the kTweenInterpolationID atoms contained in the same kTweenEntry atom.

This atom is a leaf atom. The data type of its data is QTAtomID.

This atom is required for an interpolation tween atom.

Region Tween Atoms

kTweenPictureData

Contains the data for a QuickDraw picture. Used only by a kTweenTypeQDRegion atom.

Its parent atom is a kTweenEntry atom.

A kTweenEntry atom can contain only one kTweenPictureData or kTweenRegionData atom. The ID of this atom is always 1. The index of this atom is always 1.

This atom is a leaf atom. The data type of its data is Picture.

Either a kTweenPictureData or kTweenRegionData atom is required for a kTweenTypeQDRegion atom.

2012-08-14 | Copyright © 2004, 2012 Apple Inc. All Rights Reserved.

`kTweenRegionData`

Contains the data for a QuickDraw region. Used only by a `kTweenTypeQDRegion` atom.

Its parent atom is a `kTweenEntry` atom.

A `kTweenEntry` atom can contain only one `kTweenRegionData` or `kTweenPictureData` atom. The ID of this atom is always 1. The index of this atom is always 1.

This atom is a leaf atom. The data type of its data is `Region`.

Either a `kTweenPictureData` or `kTweenRegionData` atom is required for a `kTweenTypeQDRegion` tween.

Sequence Tween Atoms

`kTweenSequenceElement`

Specifies an entry in a tween sequence.

Its parent is the tween QT atom container (which you specify with the constant `kParentAtomIsContainer`).

The ID of a `kTweenSequenceElement` atom must be unique among the `kTweenSequenceElement` atoms in the same QT atom container. The index of a `kTweenSequenceElement` atom specifies its order in the sequence; the first entry in the sequence has the index 1, the second 2, and so on.

This atom is a leaf atom. The data type of its data is `TweenSequenceEntryRecord`, a data structure that contains the following fields:

`endPercent`

A value of type `Fixed` that specifies the point in the duration of the tween media sample at which the sequence entry ends. This is expressed as a percentage; for example, if the value is 75.0, the sequence entry ends after three-quarters of the total duration of the tween media sample have elapsed. The sequence entry begins after the end of the previous sequence entry or, for the first entry in the sequence, at the beginning of the tween media sample.

`tweenAtomID`

A value of type `QTAtomID` that specifies the `kTweenEntry` atom containing the tween for the sequence element. The `kTweenEntry` atom and the `kTweenSequenceElement` atom must both be a child atoms of the same tween QT atom container.

`dataAtomID`

A value of type `QTAtomID` that specifies the `kTweenData` atom containing the data for the tween. This atom must be a child atom of the atom specified by the `tweenAtomID` field.

2012-08-14 | Copyright © 2004, 2012 Apple Inc. All Rights Reserved.

Modifier Tracks

The addition of modifier tracks in QuickTime 2.1 introduced the capability for creating dynamic movies. (A modifier track sends data to another track; by comparison, a track reference is an association.) For example, instead of playing video in a normal way, a video track could send its image data to a sprite track. The sprite track then could use that video data to replace the image of one of its sprites. When the movie is played, the video track appears as a sprite.

Modifier tracks are not a new type of track. Instead, they are a new way of using the data in existing tracks. A modifier track does not present its data, but sends it to another track that uses the data to modify how it presents its own data. Any track can be either a sender or a presenter, but not both. Previously, all tracks were presenters.

Another use of modifier tracks is to store a series of sound volume levels, which is what occurs when you work with a tween track. These sound levels can be sent to a sound track as it plays to dynamically adjust the volume. A similar use of modifier tracks is to store location and size information. This data can be sent to a video track to cause it to move and resize as it plays.

Because a modifier track can send its data to more than one track, you can easily synchronize actions between multiple tracks. For example, a single modifier track containing matrices as its samples can make two separate video tracks follow the same path.

See "Creating Movies with Modifier Tracks" (page 343) for more information about using modifier tracks.

Limitations of Spatial Modifier Tracks

A modifier track may cause a track to move outside of its original boundary regions. This may present problems, since applications do not expect the dimensions or location of a QuickTime movie to change over time.

To ensure that a movie maintains a constant location and size, the Movie Toolbox limits the area in which a spatially modified track can be displayed. A movie's "natural" shape is defined by the region returned by the `GetMovieBoundsRgn` function. The toolbox clips all spatially modified tracks against the region returned by `GetMovieBoundsRgn`. This means that a track can move outside of its initial boundary regions, but it cannot move beyond the combined initial boundary regions of all tracks in the movie. Areas uncovered by a moving track are handled by the toolbox in the same way as areas uncovered by tracks with empty edits.

If a track has to move through a larger area than that defined by the movie's boundary region, the movie's boundary region can be enlarged to any desired size by creating a spatial track (such as a video track) of the desired size but with no data. As long as the track is enabled, it contributes to the boundary regions of the movie.

2012-08-14 | Copyright © 2004, 2012 Apple Inc. All Rights Reserved.

Track References

Although QuickTime has always allowed the creation of movies that contain more than one track, it has not been able to specify relationships between those tracks. Track references are a feature of QuickTime that allows you to relate a movie's tracks to one another. The QuickTime track-reference mechanism supports many-to-many relationships. That is, any movie track may contain one or more track references, and any track may be related to one or more other tracks in the movie.

Track references can be useful in a variety of ways. For example, track references can be used to relate timecode tracks to other movie tracks. You can use track references to identify relationships between video and sound tracks such as identifying the track that contains dialog and the track that contains background sounds. Another use of track references is to associate one or more text tracks that contain subtitles with the appropriate sound track or tracks.

Track references are also used to create chapter lists, as described in "Chapter Lists" (page 248).

Every movie track contains a list of its track references. Each track reference identifies another related track. That related track is identified by its track identifier. The track reference itself contains information that allows you to classify the references by type. This type information is stored in an `OSType` data type. You are free to specify any type value you want. Note, however, that Apple has reserved all lowercase type values.

You may create as many track references as you want, and you may create more than one reference of a given type. Each track reference of a given type is assigned an index value. The index values start at 1 for each different reference type. The Movie Toolbox maintains these index values, so that they always start at 1 and count by 1.

Using the `AddTrackReference` function, you can relate one track to another. The `DeleteTrackReference` function will remove that relationship. The `SetTrackReference` and `GetTrackReference` functions allow you to modify an existing track reference so that it identifies a different track. The `GetNextTrackReferenceType` and `GetTrackReferenceCount` functions allow you to scan all of a track's track references.

For a list of track reference types, see "Track Reference Atoms" (page 73).

Chapter Lists

A chapter list provides a set of named entry points into a movie, allowing the user to jump to a preselected point in the movie from a convenient pop-up list.

2012-08-14 | Copyright © 2004, 2012 Apple Inc. All Rights Reserved.

The movie controller automatically recognizes a chapter list and will create a pop-up list from it. When the user makes a selection from the pop-up, the controller will jump to the appropriate point in the movie. Note that if the movie is sized so that the controller is too narrow to display the chapter names, the pop-up list will not appear.

To create a chapter list, you must create a text track with one sample for each chapter. The display time for each sample corresponds to the point in the movie that marks the beginning of that chapter. You must also create a track reference of type `'chap'` from an enabled track of the movie to the text track. It is the `'chap'` track reference that makes the text track into a chapter list. The track containing the reference can be of any type (audio, video, MPEG, and so on), but it must be enabled for the chapter list to be recognized.

Given an enabled track `myVideoTrack`, for example, you can use the `AddTrackReference` function to create the chapter reference:

```
AddTrackReference( myVideoTrack, theTextTrack,
    kTrackReferenceChapterList,
    &addedIndex );
```

`kTrackReferenceChapterList` is defined in `Movies.h`. It has the value `'chap'`.

The text track that constitutes the chapter list does not need to be enabled, and normally is not. If it is enabled, the text track will be displayed as part of the movie, just like any other text track, in addition to functioning as a chapter list.

If more than one enabled track includes a `'chap'` track reference, QuickTime uses the first chapter list that it finds.

3D Media

Note: 3D Media is deprecated in the QuickTime file format. The information that follows is intended to document existing content containing 3D media and should not be used for new development.

QuickTime movies store 3D image data in a base media. This media has a media type of `'qd3d'`.

3D Sample Description

The 3D sample description uses the standard sample description header, as described in "Sample Table Atoms" (page 96).

2012-08-14 | Copyright © 2004, 2012 Apple Inc. All Rights Reserved.

The data format field in the sample description is always set to 'qd3d'. The 3D media handler adds no additional fields to the sample description.

3D Sample Data

The 3D samples are stored in the 3D Metafile format developed for QuickDraw 3D.

Streaming Media

QuickTime movies store streaming data in a streaming media track. This media has a media type of 'strm'.

Streaming Media Sample Description

The streaming media sample description contains information that defines how to interpret streaming media data. This sample description is based on the standard sample description header, as described in "Sample Table Atoms" (page 96).

The streaming media sample description is documented in the QuickTime header file QTSMovie.h, as shown in Listing 4-1 (page 250).

Listing 4-1 Streaming media sample description

```
struct QTSSampleDescription {
    long                            descSize;
    long                            dataFormat;
    long                            resvd1;     /* set to 0*/
    short                           resvd2;     /* set to 0*/
    short                           dataRefIndex;
    UInt32                          version;
    UInt32                          resvd3;     /* set to 0*/
    SInt32                          flags;
                                                /* qt atoms follow:*/
                                    /* long size, long type, some  data*/
                                                /* repeat as necessary*/
};
typedef struct QTSSampleDescription     QTSSampleDescription;
```

Copyright © 2004, 2012 Apple Inc. All Rights Reserved.

The sample format depends on the `dataFormat` field of the `QTSSampleDescription`. The `dataFormat` field can be any value you specify. The currently defined values are `'rtsp'` and `'sdp '`.

If `'rtsp'`, the sample can be just an rtsp URL. It can also be any value that you can put in a `.rtsp` file, as defined at

`http://streaming.apple.com/qtstreaming/documentation/userdocs/rtsptags.htm`

If `'sdp '`, then the sample is an SDP file. This would be used to receive a multicast broadcast.

Hint Media

The QuickTime file format supports streaming of media data over a network as well as local playback. The process of sending protocol data units is time-based, just like the display of time-based data, and is therefore suitably described by a time-based format. A QuickTime file or movie that supports streaming includes information about the data units to stream. This information is included in additional tracks of the movie called hint tracks.

Hint tracks contain instructions for a streaming server which assist in the formation of packets. These instructions may contain immediate data for the server to send (for example, header information) or reference segments of the media data. These instructions are encoded in the QuickTime file in the same way that editing or presentation information is encoded in a QuickTime file for local playback.

Instead of editing or presentation information, information is provided which allows a server to packetize the media data in a manner suitable for streaming, using a specific network transport.

The same media data is used in a QuickTime file which contains hints, whether it is for local playback, or streaming over a number of different transport types. Separate hint tracks for different transport types may be included within the same file and the media will play over all such transport types without making any additional copies of the media itself. In addition, existing media can be easily made streamable by the addition of appropriate hint tracks for specific transports. The media data itself need not be recast or reformatted in any way.

Typically, hinting is performed by media packetizer components. QuickTime selects an appropriate media packetizer for each track and routes each packetizer's output through an Apple-provided packet builder to create a hint track. One hint track is created for each streamable track in the movie.

Hint tracks are quite small compared with audio or video tracks. A movie that contains hint tracks can be played from a local disk or streamed over HTTP, similar to any other QuickTime movie. Hint tracks are only used when streaming a movie over a real-time media streaming protocol, such as RTP.

Support for streaming in the QuickTime file format is based upon the following considerations:

2012-08-14 | Copyright © 2004, 2012 Apple Inc. All Rights Reserved.

- Media data represented as a set of network-independent standard QuickTime tracks, which may be played or edited, as normal.
- A common declaration and base structure for server hint tracks; this common format is protocol independent, but contains the declarations of which protocols are described in the server tracks.
- A specific design of the server hint tracks for each protocol which may be transmitted; all these designs use the same basic structure.

The resulting streams, sent by the servers under the direction of hint tracks, do not need to contain any trace of QuickTime information. This approach does not require that QuickTime, or its structures or declaration style, be used either in the data on the wire or in the decoding station. For example, a QuickTime file using H.261 video and DVI audio, streamed under Real-Time Protocol (RTP), results in a packet stream which is fully compliant with the IETF specifications for packing those codings into RTP.

Hint tracks are built and flagged, so that when the movie is viewed directly (not streamed), they are ignored.

The next section describes a generic format for streaming hints to be stored in a QuickTime movie.

Adding Hint Tracks to a Movie

To store packetization hints, one or more hint tracks are added to a movie. Each hint track contains hints for at least one actual media track to be streamed. A streamed media track may have more than one hint track. For example, it might have a separate hint track for the different packet sizes the server supports, or it might have different hint tracks for different protocols. It is not required that all media tracks have corresponding hint tracks in a movie.

The sample time of a hint sample corresponds to the sample time of the media contained in the packets generated by that hint sample. The hint sample may also contain a transmission time for each packet. (The format for the hint sample is specific to the hint track type.)

The hint track may have a different time scale than its referenced media tracks.

The `flags` field in the track header atom (`'tkhd'`) must be set to 0x000000, indicating that the track is inactive and is not part of the movie, preview, or poster.

The `subType` field of the handler description atom (`'hdlr'`) contains `'hint'`, indicating that the media type is packetization hints.

Note that if a QuickTime media track is edited, any previously stored packetization hints may become invalid. Comparing the modification dates of the media track and the hint track is one way to determine this scenario, but it is far from being foolproof. Since the hint track keeps track of which original track media samples and

2012-08-14 | Copyright © 2004, 2012 Apple Inc. All Rights Reserved.

sample descriptions to play at specific times, changes that affect those parts of the original track or media make those hints invalid. Changes to a movie that do not invalidate existing hint tracks include flattening (when there are no edit lists), and adding new tracks. Changes that invalidate hint tracks include:

- Flattening (when there are edit lists)
- Adding or deleting samples
- Changing a track's time scale
- Changing sample descriptions

> **Warning:** Hint tracks are marked as inactive, so calling the `FlattenMovie` function with the `flattenActiveTracksOnly` bit set deletes all hint tracks from a movie.

Packetization Hint Media Header Atom

In QuickTime movies, the media information atom (`'minf'`) contains header data specific to the media. For hint tracks, the media header is a base media information atom (`'gmhd'`). The hint track must contain the base media information atom.

Hint Track User Data Atom

Each hint track may contain track user data atoms that apply to only to the corresponding hint track. There are currently two such atoms defined.

- User data atom type `'hinf'`.

This contains statistics for the hint track. The `'hinf'` atom contains child atoms as defined in Table 4-17 (page 254). In some cases, there are both 32-bit and 64-bit counters available. Any unknown types should be ignored.

- User data atom type `'hnti'`.

This may contain child atoms. Child atoms that start with `'sdp '` (note, again, the space) contain SDP text for this track. Text from these child atoms must be inserted into the proper place in the SDP text for the movie, after any common SDP text. This is analogous to the movie-level `'hnti'` atom.

2012-08-14 | Copyright © 2004, 2012 Apple Inc. All Rights Reserved.

Movie Hint Info Atom

A movie may contain an 'hnti' movie user data atom, which may contain one or more child atoms. The child atom contents start with 4 bytes that specify the transport and 4 bytes that specify the type of data contained in the rest of the child atom. Currently, the only defined transport is 'rtp ' (note the space) and the only content data type defined is 'sdp ' (note the space). Child atoms whose transport or type combinations you don't recognize should be skipped.

The text in an atom of type 'rtp sdp ' should be inserted (in the proper place) into the SDP information generated from this file (for example, by a streaming server) before any SDP information for specific tracks.

Table 4-17 (page 254) describes the type and values of the 'hnti' atom.

Table 4-17 The 'hinf' atom type containing child atoms

Type	Value	Description
'trpY´	8 bytes	The total number of bytes that will be sent, including 12-byte RTP headers, but not including any network headers.
'totl'	4 bytes	4-byte version of 'trpY´
'nump'	8 bytes	The total number of network packets that will be sent (if the application knows there is a 28-byte network header, it can multiply 28 by this number and add it to the 'trpY´ value to get the true number of bytes sent.
'npck'	4 bytes	4-byte version of 'nump'
'tpyl'	8 bytes	The total number of bytes that will be sent, not including 12-byte RTP headers.
'tpaY´	4 bytes	4-byte version of 'tpyl'
'maxr'	8 bytes	The maximum data rate. This atom contains two numbers: g, followed by m (both 32-bit values). g is the granularity, in milliseconds. m is the maximum data rate given that granularity.<br>For example, if g is 1 second, then m is the maximum data rate over any 1 second. There may be multiple 'maxr' atoms, with different values for g. The maximum data rate calculation does not include any network headers (but does include 12-byte RTP headers).
'dmed'	8 bytes	The number of bytes from the media track to be sent.
'dimm'	8 bytes	The number of bytes of immediate data to be sent.
'drep'	8 bytes	The number of bytes of repeated data to be sent.

Copyright © 2004, 2012 Apple Inc. All Rights Reserved.

Type	Value	Description
`'tmin'`	4 bytes	The smallest relative transmission time, in milliseconds.
`'tmax'`	4 bytes	The largest relative transmission time, in milliseconds.
`'pmax'`	4 bytes	The largest packet, in bytes; includes 12-byte RTP header.
`'dmax'`	4 bytes	The largest packet duration, in milliseconds.
`'payt'`	Variable	The payload type, which includes payload number (32-bits) followed by `rtpmap` payload string (Pascal string).

Note: Any of the atoms shown in Table 4-17 (page 254) may or may not be present. These atoms are not guaranteed.

Finding an Original Media Track From a Hint Track

Like any other QuickTime track, hint tracks can contain track reference atoms. Exactly one of these must be of track reference type `'hint'`, and its internal list must contain at least one track ID, which is the track ID of the original media track. Like other track reference atoms, there may be empty references in this list, indicated by a track ID of 0. For hint tracks that refer to more than one track, the index number (starting at 1, and including any 0 entries) is used in the media track reference index field in some of the packet data table entry modes.

For example, if you have MPEG-1 video at track ID 11 and MPEG-1 layer 2 audio at track ID 12, and you are creating a RTP hint track that encapsulates these in an MPEG-2 transport, you need to refer to both tracks. You can also assume that there are some empty entries and other track references in your hint track atom reference atom's list. So it might look like this: 11, 0, 0, 14, 0, 12, 0. When you are assembling packets from audio and video tracks 11 and 12, you use their list indexes (1 and 6) in the media track ref index field.

If you have only one media track listed in your hint track reference, you may simply use a 0 in the media track ref index field.

RTP Hint Tracks

RTP hint tracks contain information that allows a streaming server to create RTP streams from a QuickTime movie, without requiring the server to know anything about the media type, compression, or payload format.

2012-08-14 | Copyright © 2004, 2012 Apple Inc. All Rights Reserved.

In RTP, each media stream, such as an audio or video track, is sent as a separate RTP stream. Consequently, each media track in the movie has an associated RTP hint track containing the data necessary to packetize it for RTP transport, and each hint track contains a track reference back to its associated media track.

Media tracks that do not have an associated RTP hint track cannot be streamed over RTP and should be ignored by RTP streaming servers.

It is possible for a media track to have more than one associated hint track. The hint track contains information such as the packet size and time scale in the hint track's sample description. This minimizes the runtime server load, but in order to support multiple packet sizes it is necessary to have multiple RTP hint tracks for each media track, each with different a packet size. A similar mechanism could be used to provide hint tracks for multiple protocols in the future.

It is also possible for a single hint track to refer to more than one media stream. For example, audio and video MPEG elementary streams could be multiplexed into a single systems stream RTP payload format, and a single hint track would contain the necessary information to combine both elementary streams into a single series of RTP packets.

This is the exception rather than the rule, however. In general, multiplexing is achieved by using IP's port-level multiplexing, not by interleaving the data from multiple streams into a single RTP session.

The hint track is related to each base media track by a track reference declaration. The sample description for RTP declares the maximum packet size that this hint track will generate. Partial session description (SDP) information is stored in the track's user data atom.

Hint Sample Data Format

The sample description atom (`'stsd'`) contains information about the hint track samples. It specifies the data format (note that currently only RTP data format is defined) and the data reference to use (if more than one is defined) to locate the hint track sample data. It also contains some general information about this hint track, such as the hint track version number, the maximum packet size allowed by this hint track, and the RTP time scale. It may contain additional information, such as the random offsets to add to the RTP time stamp and sequence number.

The sample description atom can contain a table of sample descriptions to accommodate media that are encoded in multiple formats, but a hint track can be expected to have a single sample description at this time.

The sample description for hint tracks is defined in Table 4-18 (page 257).

2012-08-14 | Copyright © 2004, 2012 Apple Inc. All Rights Reserved.

Table 4-18 Hint track sample description

Field	Bytes
Size	4
Data format	4
Reserved	6
Data reference index	2
Hint track version	2
Last compatible hint track version	2
Max packet size	4
Additional data table	variable

Field descriptions

Size

A 32-bit integer specifying the size of this sample description in bytes.

Data format

A four-character code indicating the data format of the hint track samples. Only `'rtp '` is currently defined. Note that the fourth character in `'rtp '` is an ASCII blank space (0x20). Do not attempt to packetize data whose format you do not recognize.

Reserved

Six bytes that must be set to 0.

Data reference index

This field indirectly specifies where to find the hint track sample data. The data reference is a file or resource specified by the data reference atom (`'dref'`) inside the data information atom (`'dinf'`) of the hint track. The data information atom can contain a table of data references, and the data reference index is a 16-bit integer that tells you which entry in that table should be used. Normally, the hint track has a single data reference, and this index entry is set to 0.

Hint track version

A 16-bit unsigned integer indicating the version of the hint track specification. This is currently set to 1.

Last compatible hint track version

A 16-bit unsigned integer indicating the oldest hint track version with which this hint track is backward-compatible. If your application understands the hint track version specified by this field, it can work with this hint track.

2012-08-14 | Copyright © 2004, 2012 Apple Inc. All Rights Reserved.

Max packet size

A 32-bit integer indicating the packet size limit, in bytes, used when creating this hint track. The largest packet generated by this hint track will be no larger than this limit.

Additional data table

A table of variable length containing additional information. Additional information is formatted as a series of tagged entries.

This field always contains a tagged entry indicating the RTP time scale for RTP data. All other tagged entries are optional.

Three data tags are currently defined for RTP data. One tag is defined for use with any type of data. You can create additional tags. Tags are identified using four-character codes. Tags using all lowercase letters are reserved by Apple. Ignore any tagged data you do not understand.

Table entries are structured like atoms. The structure of table entries is shown in Table 4-19 (page 258).

Table 4-19 The structure of table entries

Field	Format	Bytes
Entry length	32-bit integer	4
Data tag	4-char code	4
Data	Variable	Entry length - 8

Tagged entries for the `'rtp '` data format are defined as follows:

`'tims'`

A 32-bit integer specifying the RTP time scale. This entry is required for RTP data.

`'tsro'`

A 32-bit integer specifying the offset to add to the stored time stamp when sending RTP packets. If this entry is not present, a random offset should be used, as specified by the IETF. If this entry is 0, use an offset of 0 (no offset).

`'snro'`

A 32-bit integer specifying the offset to add to the sequence number when sending RTP packets. If this entry is not present, a random offset should be used, as specified by the IETF. If this entry is 0, use an offset of 0 (no offset).

Copyright © 2004, 2012 Apple Inc. All Rights Reserved.

Packetization Hint Sample Data for Data Format 'rtp '

This section describes the sample data for the `'rtp '` format. The `'rtp '` format assumes that the server is sending data using Real-Time Transport Protocol (RTP). This format also assumes that the server "knows" about RTP headers but does not require that the server know anything about specific media headers, including media headers defined in various IETF drafts.

Each sample in the hint track will generate one or more RTP packets. Each entry in the sample data table in a hint track sample corresponds to a single RTP packet. Samples in the hint track may or may not correspond exactly to samples in the media track. Data in the hint track sample is byte aligned, but not 32-bit aligned.

The RTP timestamps of all packets in a hint sample are the same as the hint sample time. In other words, packets that do not have the same RTP timestamp cannot be placed in the same hint sample.

The RTP hint track time scale should be reasonably chosen so that there is adequate spacing between samples (as well as adequate spacing between transmission times for packets within a sample).

The packetization hint sample data contains the data elements listed in Table 4-20 (page 259).

Table 4-20 Packetization hint data elements

Packetization hint sample data	Bytes
Entry count	2
Reserved	2
Packet entry table	Variable
Additional data	Variable

Field descriptions

Entry count
: A 16-bit unsigned integer indicating the number of packet entries in the table. Each entry in the table corresponds to a packet. Multiple entries in a single sample indicate that the media sample had to be split into multiple packets. A sample with an entry count of 0 is reserved and, if encountered, must be skipped.

Reserved
: Two bytes that must be set to 0.

Packet entry table
: A variable length table containing packet entries. Packet entries are defined below.

2012-08-14 | Copyright © 2004, 2012 Apple Inc. All Rights Reserved.

Additional data

A variable length field containing data pointed to by the entries in the data table.

The packet entry contains the data elements listed in Table 4-21 (page 260).

Table 4-21 Packet entry data elements

Packet entry	Bytes
Relative packet transmission time	4
RTP header info	2
RTP sequence number	2
Flags	2
Entry count	2
Extra information TLVs	0 or variable
Data table	variable

Relative packet transmission time

A 32-bit signed integer value, indicating the time, in the hint track's time scale, to send this packet relative to the hint sample's actual time. Negative values mean that the packet will be sent earlier than real time, which is useful for smoothing the data rate. Positive values are useful for repeating packets at later times. Within each hint sample track, each packet time stamp must be non-decreasing.

RTP header info

A 16-bit integer specifying various values to be set in the RTP header. The bits of the field are defined as follows.

0	1	2	3	4	5	6	7	8	9	0	1	2	3	4	5
reserved		P	X	reserved				M	payload type						

The RTP header information field contains the elements listed in Table 4-22 (page 261).

Copyright © 2004, 2012 Apple Inc. All Rights Reserved.

Table 4-22 RTP header information elements

Field	Bit#	Description
P	2	A 1-bit number corresponding to the padding (P) bit in the RTP header. This bit should probably not be set, since a server that needs different packet padding would need to unpad and repad the packet itself.
X	3	A 1-bit number corresponding to the extension (X) bit in the RTP header. This bit should probably not be set, since a server that needs to send its own RTP extension would either not be able to, or would be forced to replace any extensions from the hint track.
M	8	A 1-bit number corresponding to the marker (M) bit in the RTP header.
Payload type	9-15	A 7-bit number corresponding to the payload type (PT) field of the RTP header.

All undefined bits are reserved and must be set to zero. Note that the location of the defined bits are in the same bit location as in the RTP header.

RTP sequence number

A 16-bit integer specifying the RTP sequence number for this packet. The RTP server adds a random offset to this sequence number before transmitting the packet. This field allows re-transmission of packets—for example, the same packet can be assembled with the same sequence number and a different (later) packet transmission time. A text sample with a duration of 5 minutes can be retransmitted every 10 seconds, so that clients that miss the original sample transmission (perhaps they started playing the movie in the middle) will be refreshed after a maximum of 10 seconds.

Flags

A 16-bit field indicating certain attributes for this packet. Defined bits are shown in Figure 4-16 (page 261).

Figure 4-16 Packet attribute flags

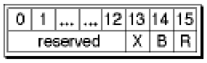

Entry count

A 16-bit unsigned integer specifying the number of entries in the data table.

Extra information TLVs

The extra information TLVs listed in Table 4-23 (page 262) are present if and only if the X bit is set in the flags field. This provides a way of extending the hint track format without changing the version, while allowing backward compatibility.

Copyright © 2004, 2012 Apple Inc. All Rights Reserved.

Table 4-23 Extra information TLVs

Extra information TLVs	Bytes
Extra information size	4
TLV size	4
TLV type	4
TLV data	Padded to 4-byte boundary(int(TLV Size -8 +3) / 4 * 4
TLV size	4
TLV type	4
TLV data	Padded to 4-byte boundary(int(TLV Size -8 +3) / 4 * 4
TLV size and so forth	...

Extra information size

A 32-bit number that is the total size of all extra information TLVs in this packet, including the 4 bytes used for this field. An empty Extra information TLVs table would just be the extra information size, having the value 4. (In this case, it would be more efficient simply to not set the X bit and save 4 bytes just to represent the empty table.)

TLV size

A 32-bit number that is the total size of this one TLV entry, including 4 bytes for the size, 4 bytes for the type, and any data bytes, but not including padding required to align to the next 4 byte boundary.

TLV type

A 32-bit tag (a four-character `OSType`) identifying the TLV. Servers must ignore TLV types that they do not recognize. Note that TLV types containing all lowercase letters are reserved by Apple Computer.

TLV data

The data for the TLV.

In order to support MPEG (and other data types) whose RTP timestamp is not monotonically increasing and directly calculated from the sample timestamp, the TLV type listed in Table 4-24 (page 262) is defined.

Table 4-24 TLV type

Size	Type	Data Description
12	`'rtpo'`	A signed 32-bit integer to be added to the RTP timestamp, which is derived from the hint sample timestamp.

2012-08-14 | Copyright © 2004, 2012 Apple Inc. All Rights Reserved.

Data table

A table that defines the data to be put in the payload portion of the RTP packet. This table defines various places the data can be retrieved. Table entries are listed in Table 4-25 (page 263).

Table 4-25 Data table entries

Data table entry	Bytes
Data source	1
Data	15

The data source field of the entry table indicates how the other 15 bytes of the entry are to be interpreted. Values of 0 through 4 are defined. The various data table formats are defined below.

Although there are various schemes, note that the entries in the various schemes are the same size, 16 bytes long.

Data Modes

No-Op Data Mode

The data table entry has the format for no-op mode shown in Figure 4-17 (page 263).

Figure 4-17 No-op data mode format

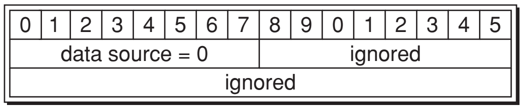

Field descriptions

Data source = 0

A value of 0 indicates that this data table entry is to be ignored.

Copyright © 2004, 2012 Apple Inc. All Rights Reserved.

Immediate Data Mode

The data table entry has the format for immediate mode shown in Figure 4-18 (page 264).

Figure 4-18 Immediate data mode table entry

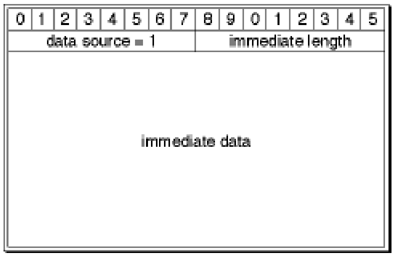

Field descriptions

Data source = 1
: A value of 1 indicates that the data is to be immediately taken from the bytes of data that follow.

Immediate length
: An 8-bit integer indicating the number of bytes to take from the data that follows. Legal values range from 0 to 14.

Immediate data
: 14 bytes of data to place into the payload portion of the packet. Only the first number of bytes indicated by the immediate length field is used.

Sample Mode

The data table entry has the format for sample mode shown in Figure 4-19 (page 264).

Figure 4-19 Sample mode table entry format

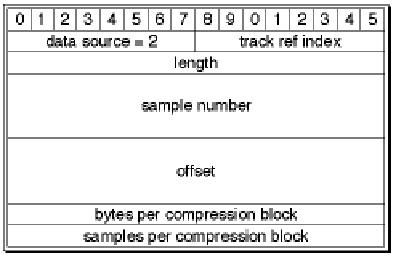

Field descriptions

Data source = 2
: A value of 2 indicates that the data is to be taken from a track's sample data.

Copyright © 2004, 2012 Apple Inc. All Rights Reserved.

Track ref index
: A value that indicates which track the sample data will come from. A value of 0 means that there is exactly one media track referenced, so use that. Values from 1 to 127 are indexes into the hint track reference atom entries, indicating which original media track the sample is to be read from. A value of -1 means the hint track itself, that is, get the sample from the same track as the hint sample you are currently parsing.

Length
: A 16-bit integer specifying the number of bytes in the sample to copy.

Sample number
: A 32-bit integer specifying sample number of the track.

Offset
: A 32-bit integer specifying the offset from the start of the sample from which to start copying. If you are referencing samples in the hint track, this will generally points into the Additional Data area.

Bytes per compression block
: A 16-bit unsigned integer specifying the number of bytes that results from compressing the number of samples in the Samples per compression block field. A value of 0 is equivalent to a value of 1.

Samples per compression block
: A 16-bit unsigned integer specifying the uncompressed samples per compression block. A value of 0 is equivalent to a value of 1.

If the bytes per compression block and/or the samples per compression block is greater than 1, than this ratio is used to translate a sample number into an actual byte offset.

This ratio mode is typically used for compressed sound tracks. Note that for QuickTime sound tracks, the bytes per compression block also factors in the number of sound channels in that stream, so a QuickTime stereo sound stream's BPCB would be twice that of a mono stream of the same sound format.

(CB = NS * BPCB / SPCB)

where CB = compressed bytes, NS = number of samples, BPCB = bytes per compression block, and SPCB = samples per compression block.

An example:

A GSM compression block is typically 160 samples packed into 33 bytes.

So, BPCB = 33 and SPCB = 160.

The hint sample requests 33 bytes of data starting at the 161st media sample. Assume that the first QuickTime chunk contains at least 320 samples. So after determining that this data will come from chunk 1, and knowing where chunk 1 starts, you must use this ratio to adjust the offset into the file where the requested samples will be found:

```
chunk_number = 1; /* calculated by walking the sample-to-chunk atom  */
```

2012-08-14 | Copyright © 2004, 2012 Apple Inc. All Rights Reserved.

```
first_sample_in_this_chunk = 1; /* also calculated from that atom  */
chunk_offset = chunk_offsets[chunk_number]; /* from the stco atom  */
data_offset = (sample_number - first_sample_in_this_chunk) * BPCB  / SPCB;
read_from_file(chunk_offset + data_offset, length); /* read our  data */
```

Sample Description Mode

The data table entry has the format for sample description mode shown in Figure 4-20 (page 266).

Figure 4-20 Sample description mode format

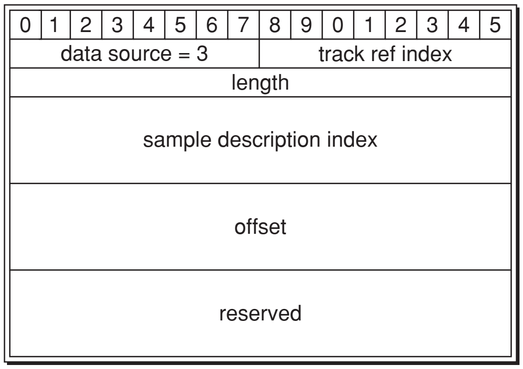

Field descriptions

Data source = 3

A value of 3 indicates that the data is to be taken from the media track's sample description table.

Track ref index

A value that indicates which track the sample description will come from. A value of 0 means that there is exactly one hint track reference, so use that. Values from 1 to 127 are indexes into the hint track reference atom entries, indicating which original media track the sample is to be read from. A value of -1 means the hint track itself, that is, get the sample description from the same track as the hint sample you are currently parsing.

Length

A 16-bit integer specifying the number of bytes to copy.

Sample description index

A 32-bit integer specifying the index into the media's sample description table.

Offset

A 32-bit integer specifying the offset from the start of the sample description from which to start copying.

Copyright © 2004, 2012 Apple Inc. All Rights Reserved.

Reserved
    Four bytes that must be set to 0.

Additional data
    A variable length field containing data pointed to by hint track sample mode entries in the data table.

VR Media

Note: VR Media is deprecated in the QuickTime file format. The information that follows is intended to document existing content containing VR Media and should not be used for new development.

This section describes the QuickTime VR world and node information atom containers, which can be obtained by calling the QuickTime VR Manager routines `QTVRGetVRWorld` and `QTVRGetNodeInfo`. Those routines, as well as a complete discussion of QuickTime VR and how your application can create QuickTime VR movies, are described in detail in *QuickTime VR*.

Many atom types contained in the VR world and node information atom containers are unique within their container. For example, each has a single header atom. Most parent atoms within an atom container are unique as well, such as the node parent atom in the VR world atom container or the hot spot parent atom in the node information atom container. For these one-time-only atoms, the atom ID is always set to 1. Unless otherwise mentioned in the descriptions of the atoms that follow, assume that the atom ID is 1.

Note that many atom structures contain two version fields, `majorVersion` and `minorVersion`. The values of these fields correspond to the constants `kQTVRMajorVersion` and `kQTVRMinorVersion` found in the header file `QuickTimeVRFormat.h`. For QuickTime 2.0 files, these values are 2 and 0.

QuickTime provides a number of routines for both creating and accessing atom containers.

Some of the leaf atoms within the VR world and node information atom containers contain fields that specify the ID of string atoms that are siblings of the leaf atom. For example, the VR world header atom contains a field for the name of the scene. The string atom is a leaf atom whose atom type is `kQTVRStringAtomType` (`'vrsg'`). Its atom ID is that specified by the referring leaf atom.

A string atom contains a string. The structure of a string atom is defined by the `QTVRStringAtom` data type.

```
typedef struct QTVRStringAtom {
    UInt16                          stringUsage;
    UInt16                          stringLength;
    unsigned char                   theString[4];
```

Copyright © 2004, 2012 Apple Inc. All Rights Reserved.

```
} QTVRStringAtom, *QTVRStringAtomPtr;
```

Field descriptions

`stringUsage`
: The string usage. This field is unused.

`stringLength`
: The length, in bytes, of the string.

`theString`
: The string. The string atom structure is extended to hold this string.

Each string atom may also have a sibling leaf atom, called the string encoding atom. The string encoding atom's atom type is `kQTVRStringEncodingAtomType` (`'vrse'`). Its atom ID is the same as that of the corresponding string atom. The string encoding atom contains a single variable, `TextEncoding`, a `UInt32`, as defined in the header file `TextCommon.h`. The value of `TextEncoding` is handed, along with the string, to the routine `QTTextToNativeText` for conversion for display on the current machine. The routine `QTTextToNativeText` is found in the header file `Movies.h`.

Note: The header file `TextCommon.h` contains constants and routines for generating and handling text encodings.

VR World Atom Container

The VR world atom container (VR world for short) includes such information as the name for the entire scene, the default node ID, and default imaging properties, as well as a list of the nodes contained in the QTVR track.

A VR world can also contain custom scene information. QuickTime VR ignores any atom types that it doesn't recognize, but you can extract those atoms from the VR world using standard QuickTime atom functions.

Copyright © 2004, 2012 Apple Inc. All Rights Reserved.

The structure of the VR world atom container is shown in Figure 4-21 (page 269). The component atoms are defined and their structures are shown in the sections that follow.

Figure 4-21 Structure of the VR world atom container

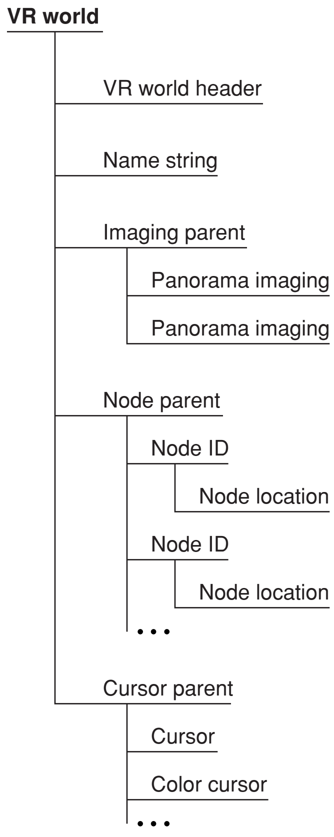

VR World Header Atom Structure

The VR world header atom is a leaf atom. Its atom type is `kQTVRWorldHeaderAtomType` (`'vrsc'`). It contains the name of the scene and the default node ID to be used when the file is first opened as well as fields reserved for future use.

The structure of a VR world header atom is defined by the `QTVRWorldHeaderAtom` data type.

```
typedef struct VRWorldHeaderAtom {
    UInt16                          majorVersion;
    UInt16                          minorVersion;
    QTAtomID                        nameAtomID;
    UInt32                          defaultNodeID;
    UInt32                          vrWorldFlags;
    UInt32                          reserved1;
```

Copyright © 2004, 2012 Apple Inc. All Rights Reserved.

```
    UInt32                              reserved2;
} VRWorldHeaderAtom, *QTVRWorldHeaderAtomPtr;
QT
QT
```

Field descriptions

`majorVersion`
: The major version number of the file format.

`minorVersion`
: The minor version number of the file format.

`nameAtomID`
: The ID of the string atom that contains the name of the scene. That atom should be a sibling of the VR world header atom. The value of this field is 0 if no name string atom exists.

`defaultNodeID`
: The ID of the default node (that is, the node to be displayed when the file is first opened).

`vrWorldFlags`
: A set of flags for the VR world. This field is unused.

`reserved1`
: Reserved. This field must be 0.

`reserved2`
: Reserved. This field must be 0.

Imaging Parent Atom

The imaging parent atom is the parent atom of one or more node-specific imaging atoms. Its atom type is `kQTVRImagingParentAtomType` (`'imgp'`). Only panoramas have an imaging atom defined.

Panorama-Imaging Atom

A panorama-imaging atom describes the default imaging characteristics for all the panoramic nodes in a scene. This atom overrides QuickTime VR's own defaults.

The panorama-imaging atom has an atom type of `kQTVRPanoImagingAtomType` (`'impn'`). Generally, there is one panorama-imaging atom for each imaging mode, so the atom ID, while it must be unique for each atom, is ignored. QuickTime VR iterates through all the panorama-imaging atoms.

The structure of a panorama-imaging atom is defined by the `QTVRPanoImagingAtom` data type:

2012-08-14 | Copyright © 2004, 2012 Apple Inc. All Rights Reserved.

```
typedef struct QTVRPanoImagingAtom {
    UInt16                              majorVersion;
    UInt16                              minorVersion;
    UInt32                              imagingMode;
    UInt32                              imagingValidFlags;
    UInt32                              correction;
    UInt32                              quality;
    UInt32                              directDraw;
    UInt32                              imagingProperties[6];
    UInt32                              reserved1;
    UInt32                              reserved2;
} QTVRPanoImagingAtom, *VRPanoImagingAtomPtr;
```

Field descriptions

`majorVersion`
: The major version number of the file format.

`minorVersion`
: The minor version number of the file format.

`imagingMode`
: The imaging mode to which the default values apply. Only `kQTVRStatic` and `kQTVRMotion` are allowed here.

`imagingValidFlags`
: A set of flags that indicate which imaging property fields in this structure are valid.

`correction`
: The default correction mode for panoramic nodes. This can be either `kQTVRNoCorrection`, `kQTVRPartialCorrection`, or `kQTVRFullCorrection`.

`quality`
: The default imaging quality for panoramic nodes.

`directDraw`
: The default direct-drawing property for panoramic nodes. This can be `true` or `false`.

`imagingProperties`
: Reserved for future panorama-imaging properties.

`reserved1`
: Reserved. This field must be 0.

2012-08-14 | Copyright © 2004, 2012 Apple Inc. All Rights Reserved.

`reserved2`
Reserved. This field must be 0.

The `imagingValidFlags` field in the panorama-imaging atom structure specifies which imaging property fields in that structure are valid. You can use these bit flags to specify a value for that field:

```
enum {
    kQTVRValidCorrection                    = 1 << 0,
    kQTVRValidQuality                       = 1 << 1,
    kQTVRValidDirectDraw                    = 1 << 2,
    kQTVRValidFirstExtraProperty            = 1 << 3
};
```

Constant Descriptions

`kQTVRValidCorrection`
The default correction mode for panorama-imaging properties. If this bit is set, the `correction` field holds a default correction mode.

`kQTVRValidQuality`
The default imaging quality for panorama-imaging properties. If this bit is set, the `quality` field holds a default imaging quality.

`kQTVRValidDirectDraw`
The default direct-draw quality for panorama-imaging properties. If this bit is set, the `directDraw` field holds a default direct-drawing property.

`kQTVRValidFirstExtraProperty`
The default imaging property for panorama-imaging properties. If this bit is set, the first element in the array in the `imagingProperties` field holds a default imaging property. As new imaging properties are added, they will be stored in this array.

2012-08-14 | Copyright © 2004, 2012 Apple Inc. All Rights Reserved.

Node Parent Atom

> **Note:** VR Media is deprecated in the QuickTime file format. The information that follows is intended to document existing content containing VR Media and should not be used for new development.

The node parent atom is the parent of one or more node ID atoms. The atom type of the node parent atom is `kQTVRNodeParentAtomType` (`'vrnp'`) and the atom type of the each node ID atom is `kQTVRNodeIDAtomType` (`'vrni'`).

There is one node ID atom for each node in the file. The atom ID of the node ID atom is the node ID of the node. The node ID atom is the parent of the node location atom. The node location atom is the only child atom defined for the node ID atom. Its atom type is `kQTVRNodeLocationAtomType` (`'nloc'`).

Node Location Atom Structure

> **Note:** VR Media is deprecated in the QuickTime file format. The information that follows is intended to document existing content containing VR Media and should not be used for new development.

The node location atom is the only child atom defined for the node ID atom. Its atom type is `kQTVRNodeLocationAtomType` (`'nloc'`). A node location atom describes the type of a node and its location.

The structure of a node location atom is defined by the `QTVRNodeLocationAtom` data type:

```
typedef struct VRNodeLocationAtom {
    UInt16                          majorVersion;
    UInt16                          minorVersion;
    OSType                          nodeType;
    UInt32                          locationFlags;
    UInt32                          locationData;
    UInt32                          reserved1;
    UInt32                          reserved2;
} VRNodeLocationAtom, *QTVRNodeLocationAtomPtr;
QT
QT
```

2012-08-14 | Copyright © 2004, 2012 Apple Inc. All Rights Reserved.

Field descriptions

`majorVersion`
The major version number of the file format.

`minorVersion`
The minor version number of the file format.

`nodeType`
The node type. This field should contain either `kQTVRPanoramaType` or `kQTVRObjectType`.

`locationFlags`
The location flags. This field must contain the value `kQTVRSameFile`, indicating that the node is to be found in the current file. In the future, these flags may indicate that the node is in a different file or at some URL location.

`locationData`
The location of the node data. When the `locationFlags` field is `kQTVRSameFile`, this field should be 0. The nodes are found in the file in the same order that they are found in the node list.

`reserved1`
Reserved. This field must be 0.

`reserved2`
Reserved. This field must be 0.

Custom Cursor Atoms

Note: VR Media is deprecated in the QuickTime file format. The information that follows is intended to document existing content containing VR Media and should not be used for new development.

The hot spot information atom, discussed in "Hot Spot Information Atom" (page 278), allows you to indicate custom cursor IDs for particular hot spots that replace the default cursors used by QuickTime VR. QuickTime VR allows you to store your custom cursors in the VR world of the movie file.

Copyright © 2004, 2012 Apple Inc. All Rights Reserved.

Note:

The use of resource forks for the storage of QuickTime media is deprecated in the QuickTime file format. The information that follows is intended to document existing content and should not be used for new development.

In Mac OS with a two-fork file system, custom cursors could be stored in the resource fork of the movie file. However, this implementation does not work on any other operating system platform (such as Windows).

The cursor parent atom is the parent of all of the custom cursor atoms stored in the VR world. Its atom type is `kQTVRCursorParentAtomType` (`'vrcp'`). The child atoms of the cursor parent are either cursor atoms or color cursor atoms. Their atom types are `kQTVRCursorAtomType ('CURS'`) and `kQTVRColorCursorAtomType` (`'crsr'`). These atoms are stored exactly as cursors or color cursors would be stored as a resource.

Node Information Atom Container

Note: VR Media is deprecated in the QuickTime file format. The information that follows is intended to document existing content containing VR Media and should not be used for new development.

The node information atom container includes general information about the node such as the node's type, ID, and name. The node information atom container also contains the list of hot spot atoms for the node. A QuickTime VR movie contains one node information atom container for each node in the file. The routine `QTVRGetNodeInfo` allows you to obtain the node information atom container for the current node or for any other node in the movie.

2012-08-14 | Copyright © 2004, 2012 Apple Inc. All Rights Reserved.

Figure 4-22 (page 276) shows the structure of the node information atom container.

Figure 4-22 Structure of the node information atom container

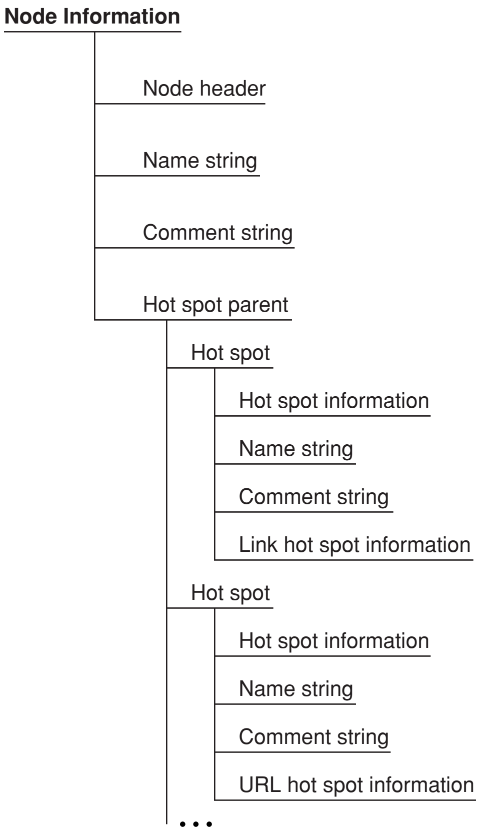

Node Header Atom Structure

A node header atom is a leaf atom that describes the type and ID of a node, as well as other information about the node. Its atom type is `kQTVRNodeHeaderAtomType` (`'ndhd'`).

The structure of a node header atom is defined by the `QTVRNodeHeaderAtom` data type:

```
typedef struct VRNodeHeaderAtom {
    UInt16                      majorVersion;
    UInt16                      minorVersion;
    OSType                      nodeType;
    QTAtomID                    nodeID;
    QTAtomID                    nameAtomID;
    QTAtomID                    commentAtomID;
    UInt32                      reserved1;
```

Copyright © 2004, 2012 Apple Inc. All Rights Reserved.

```
    UInt32                              reserved2;
} VRNodeHeaderAtom, *VRNodeHeaderAtomPtr;
```

Field descriptions

`majorVersion`
: The major version number of the file format.

`minorVersion`
: The minor version number of the file format.

`nodeType`
: The node type. This field should contain either `kQTVRPanoramaType` or `kQTVRObjectType`.

`nodeID`
: The node ID.

`nameAtomID`
: The ID of the string atom that contains the name of the node. This atom should be a sibling of the node header atom. The value of this field is 0 if no name string atom exists.

`commentAtomID`
: The ID of the string atom that contains a comment for the node. This atom should be a sibling of the node header atom. The value of this field is 0 if no comment string atom exists.

`reserved1`
: Reserved. This field must be 0.

`reserved2`
: Reserved. This field must be 0.

Hot Spot Parent Atom

The hot spot parent atom is the parent for all hot spot atoms for the node. The atom type of the hot spot parent atom is `kQTVRHotSpotParentAtomType` (`'hspa'`) and the atom type of the each hot spot atom is `kQTVRHotSpotAtomType` (`'hots'`). The atom ID of each hot spot atom is the hot spot ID for the corresponding hot spot. The hot spot ID is determined by its color index value as it is stored in the hot spot image track.

The hot spot track is an 8-bit video track that contains color information that indicates hot spots. For more information, refer to Programming With QuickTime VR.

Each hot spot atom is the parent of a number of atoms that contain information about each hot spot.

2012-08-14 | Copyright © 2004, 2012 Apple Inc. All Rights Reserved.

Hot Spot Information Atom

The hot spot information atom contains general information about a hot spot. Its atom type is `kQTVRHotSpotInfoAtomType` (`'hsin'`). Every hot spot atom should have a hot spot information atom as a child.

The structure of a hot spot information atom is defined by the `QTVRHotSpotInfoAtom` data type:

```
typedef struct VRHotSpotInfoAtom {
    UInt16                              majorVersion;
    UInt16                              minorVersion;
    OSType                              hotSpotType;
    QTAtomID                            nameAtomID;
    QTAtomID                            commentAtomID;
    SInt32                              cursorID[3];
    Float32                             bestPan;
    Float32                             bestTilt;
    Float32                             bestFOV;
    FloatPoint                          bestViewCenter;
    Rect                                hotSpotRect;
    UInt32                              flags;
    UInt32                              reserved1;
    UInt32                              reserved2;
} VRHotSpotInfoAtom, *QTVRHotSpotInfoAtomPtr;
```

Field descriptions

`majorVersion`
: The major version number of the file format.

`minorVersion`
: The minor version number of the file format.

`hotSpotType`
: The hot spot type. This type specifies which other information atoms—if any—are siblings to this one. QuickTime VR recognizes three types: `kQTVRHotSpotLinkType`, `kQTVRHotSpotURLType`, and `kQTVRHotSpotUndefinedType`.

2012-08-14 | Copyright © 2004, 2012 Apple Inc. All Rights Reserved.

`nameAtomID`
: The ID of the string atom that contains the name of the hot spot. This atom should be a sibling of the hot spot information atom. This string is displayed in the QuickTime VR controller bar when the mouse is moved over the hot spot.

`commentAtomID`
: The ID of the string atom that contains a comment for the hot spot. This atom should be a sibling of the hot spot information atom. The value of this field is 0 if no comment string atom exists.

`cursorID`
: An array of three IDs for custom hot spot cursors (that is, cursors that override the default hot spot cursors provided by QuickTime VR). The first ID (`cursorID[0]`) specifies the cursor that is displayed when it is in the hot spot. The second ID (`cursorID[1]`) specifies the cursor that is displayed when it is in the hot spot and the mouse button is down. The third ID (`cursorID[2]`) specifies the cursor that is displayed when it is in the hot spot and the mouse button is released. To retain the default cursor for any of these operations, set the corresponding cursor ID to 0. Custom cursors should be stored in the VR world atom container, as described in "VR World Atom Container" (page 268).

`bestPan`
: The best pan angle for viewing this hot spot.

`bestTilt`
: The best tilt angle for viewing this hot spot.

`bestFOV`
: The best field of view for viewing this hot spot.

`bestViewCenter`
: The best view center for viewing this hot spot; applies only to object nodes.

`hotSpotRect`
: The boundary box for this hot spot, specified as the number of pixels in full panoramic space. This field is valid only for panoramic nodes.

`flags`
: A set of hot spot flags. This field is unused.

`reserved1`
: Reserved. This field must be 0.

`reserved2`
: Reserved. This field must be 0.

2012-08-14 | Copyright © 2004, 2012 Apple Inc. All Rights Reserved.

Note: In QuickTime VR movie files, all angular values are stored as 32-bit floating-point values that specify degrees. In addition, all floating-point values conform to the IEEE Standard 754 for binary floating-point arithmetic, in big-endian format.

Specific Information Atoms

Depending on the value of the `hotSpotType` field in the hot spot info atom there may also be a type specific information atom. The atom type of the type-specific atom is the hot spot type.

Link Hot Spot Atom

The link hot spot atom specifies information for hot spots of type `kQTVRHotSpotLinkType` (`'link'`). Its atom type is thus `'link'`. The link hot spot atom contains specific information about a link hot spot.

The structure of a link hot spot atom is defined by the `QTVRLinkHotSpotAtom` data type:

```
typedef struct VRLinkHotSpotAtom {
    UInt16                                  majorVersion;
    UInt16                                  minorVersion;
    UInt32                                  toNodeID;
    UInt32                                  fromValidFlags;
    Float32                                 fromPan;
    Float32                                 fromTilt;
    Float32                                 fromFOV;
    FloatPoint                              fromViewCenter;
    UInt32                                  toValidFlags;
    Float32                                 toPan;
    Float32                                 toTilt;
    Float32                                 toFOV;
    FloatPoint                              toViewCenter;
    Float32                                 distance;
    UInt32                                  flags;
    UInt32                                  reserved1;
    UInt32                                  reserved2;
} VRLinkHotSpotAtom, *VRLinkHotSpotAtomPtr;
```

2012-08-14 | Copyright © 2004, 2012 Apple Inc. All Rights Reserved.

Field descriptions

`majorVersion`
: The major version number of the file format.

`minorVersion`
: The minor version number of the file format.

`toNodeID`
: The ID of the destination node (that is, the node to which this hot spot is linked).

`fromValidFlags`
: A set of flags that indicate which source node view settings are valid.

`fromPan`
: The preferred from-pan angle at the source node (that is, the node containing the hot spot).

`fromTilt`
: The preferred from-tilt angle at the source node.

`fromFOV`
: The preferred from-field of view at the source node.

`fromViewCenter`
: The preferred from-view center at the source node.

`toValidFlags`
: A set of flags that indicate which destination node view settings are valid.

`toPan`
: The pan angle to use when displaying the destination node.

`toTilt`
: The tilt angle to use when displaying the destination node.

`toFOV`
: The field of view to use when displaying the destination node.

`toViewCenter`
: The view center to use when displaying the destination node.

`distance`
: The distance between the source node and the destination node.

`flags`
: A set of link hot spot flags. This field is unused and should be set to 0.

`reserved1`
: Reserved. This field must be 0.

2012-08-14 | Copyright © 2004, 2012 Apple Inc. All Rights Reserved.

`reserved2`
: Reserved. This field must be 0.

Certain fields in the link hot spot atom are not used by QuickTime VR. The `fromValidFlags` field is generally set to 0 and the other `from` fields are not used. However, these fields could be quite useful if you have created a transition movie from one node to another. The `from` angles can be used to swing the current view of the source node to align with the first frame of the transition movie. The `distance` field is intended for use with 3D applications, but is also not used by QuickTime VR.

Link Hot Spot Valid Flags

The `toValidFlags` field in the link hot spot atom structure specifies which view settings are to be used when moving to a destination node from a hot spot. You can use these bit flags to specify a value for that field:

```
enum {
    kQTVRValidPan                              = 1 << 0,
    kQTVRValidTilt                             = 1 << 1,
    kQTVRValidFOV                              = 1 << 2,
    kQTVRValidViewCenter                       = 1 << 3
};
```

Constant Descriptions

`kQTVRValidPan`
: The setting for using the destination pan angle.

`kQTVRValidTilt`
: The setting for using the destination tilt angle.

`kQTVRValidFOV`
: The setting for using the destination field of view.

`kQTVRValidViewCenter`
: The setting for using the destination view center.

2012-08-14 | Copyright © 2004, 2012 Apple Inc. All Rights Reserved.

URL Hot Spot Atom

Note: VR Media is deprecated in the QuickTime file format. The information that follows is intended to document existing content containing VR Media and should not be used for new development.

The URL hot spot atom has an atom type of `kQTVRHotSpotURLType` (`'url '`). The URL hot spot atom contains a URL string for a particular Web location (for example, `http://quicktimevr.apple.com`). QuickTime VR automatically links to this URL when the hot spot is clicked.

Support for Wired Actions

Note: VR Media is deprecated in the QuickTime file format. The information that follows is intended to document existing content containing VR Media and should not be used for new development.

Certain actions on a QuickTime VR movie can trigger wired actions if the appropriate event handler atoms have been added to the file. This section discusses what atoms must be included in the QuickTime VR file to support wired actions.

As with sprite tracks, the presence of a certain atom in the media property atom container of the QTVR track enables the handling of wired actions. This atom is of type `kSpriteTrackPropertyHasActions`, which has a single Boolean value that must be set to `true`.

When certain events occur and the appropriate event handler atom is found in the QTVR file, then that atom is passed to QuickTime to perform any actions specified in the atom. The event handler atoms themselves must be added to the node information atom container in the QTVR track. There are two types of event handlers for QTVR nodes: global and hot spot specific. The currently supported global event handlers are `kQTEventFrameLoaded` and `kQTEventIdle`. The event handler atoms for these are located at the root level of the node information atom container. A global event handler atom's type is set to the event type and its ID is set to 1.

Hot spot–specific event handler atoms are located in the specific hot spot atom as a sibling to the hot spot info atom. For these atoms, the atom type is always `kQTEventType` and the ID is the `event` type. Supported hot spot–specific event types are `kQTEventMouseClick`, `kQTEventMouseClickEnd`, `kQTEventMouseClickEndTriggerButton`, and `kQTEventMouseEnter`, `kQTEventMouseExit`.

The specific actions that cause these events to be generated are described as follows:

2012-08-14 | Copyright © 2004, 2012 Apple Inc. All Rights Reserved.

`kQTEventFrameLoaded ('fram')`
: A wired action that is generated when a node is entered, before any application-installed entering-node procedure is called (this event processing is considered part of the node setup that occurs before the application's routine is called).

`kQTEventIdle ('idle')`
: A wired action that is generated every n ticks, where n is defined by the contents of the `kSpriteTrackPropertyQTIdleEventsFrequency` atom (`SInt32`) in the media property atom container. When appropriate, this event is triggered before any normal idle processing occurs for the QuickTime VR movie.

`kQTEventMouseClick ('clik')`
: A wired action that is generated when the mouse goes down over a hot spot.

`kQTEventMouseClickEnd ('cend')`
: A wired action that is generated when the mouse goes up after a `kQTEventMouseClick` is generated, regardless of whether the mouse is still over the hot spot originally clicked. This event occurs prior to QuickTime VR's normal mouse-up processing.

`kQTEventMouseClickEndTriggerButton ('trig')`
: A wired action that is generated when a click end triggers a hot spot (using the same criterion as used by QuickTime VR in 2.1 for link/url hot spot execution). This event occurs prior to QuickTime VR's normal hot spot–trigger processing.

`kQTEventMouseEnter ('entr')`, `kQTEventMouseExit('exit')`
: Wired action that are generated when the mouse rolls into or out of a hot spot, respectively. These events occur whether or not the mouse is down and whether or not the movie is being panned. These events occur after any application-installed `MouseOverHotSpotProc` is called, and will be cancelled if the return value from the application's routine indicates that QuickTimeVR's normal over–hot spot processing should not take place.

QuickTime VR File Format

Note: VR Media is deprecated in the QuickTime file format. The information that follows is intended to document existing content containing VR Media and should not be used for new development.

A QuickTime VR movie is stored on disk in a format known as the QuickTime VR file format. Beginning in QuickTime VR 2.0, a QuickTime VR movie could contain one or more nodes. Each node is either a panorama or an object. In addition, a QuickTime VR movie could contain various types of hot spots, including links between any two types of nodes.

Copyright © 2004, 2012 Apple Inc. All Rights Reserved.

> **Important:** This section describes the file format supported by version 2.1 of the QuickTime VR Manager.

All QuickTime VR movies contain a single QTVR track, a special type of QuickTime track that maintains a list of the nodes in the movie. Each individual sample in a QTVR track contains general information and hot spot information for a particular node.

If a QuickTime VR movie contains any panoramic nodes, that movie also contains a single panorama track, and if it contains any object nodes, it also contains a single object track. The panorama and object tracks contain information specific to the panoramas or objects in the movie. The actual image data for both panoramas and objects is usually stored in standard QuickTime video tracks, hereafter referred to as image tracks. (An image track can also be any type of track that is capable of displaying an image, such as a QuickTime 3D track.) The individual frames in the image track for a panorama make up the diced frames of the original single panoramic image. The frames for the image track of an object represent the many different views of the object. Hot spot image data is stored in parallel video tracks for both panoramas and objects.

Single-Node Panoramic Movies

Figure 4-23 (page 285) illustrates the basic structure of a single-node panoramic movie. As you can see, every panoramic movie contains at least three tracks: a QTVR track, a panorama track, and a panorama image track.

Figure 4-23 The structure of a single-node panoramic movie file

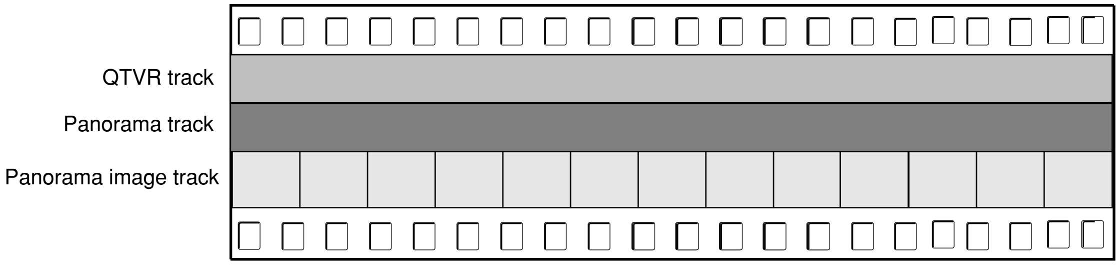

For a single-node panoramic movie, the QTVR track contains just one sample. There is a corresponding sample in the panorama track, whose time and duration are the same as the time and duration of the sample in the QTVR track. The time base of the movie is used to locate the proper video samples in the panorama image track. For a panoramic movie, the video sample for the first diced frame of a node's panoramic image is located at the same time as the corresponding QTVR and panorama track samples. The total duration of all the video samples is the same as the duration of the corresponding QTVR sample and the panorama sample.

A panoramic movie can contain an optional hot spot image track and any number of standard QuickTime tracks. A panoramic movie can also contain panoramic image tracks with a lower resolution. The video samples in these low-resolution image tracks must be located at the same time and must have the same total duration as the QTVR track. Likewise, the video samples for a hot spot image track, if one exists, must be located at the same time and must have the same total duration as the QTVR track.

2012-08-14 | Copyright © 2004, 2012 Apple Inc. All Rights Reserved.

Single-Node Object Movies

Figure 4-24 (page 286) illustrates the basic structure of a single-node object movie. As you can see, every object movie contains at least three tracks: a QTVR track, an object track, and an object image track.

Figure 4-24 The structure of a single-node object movie file

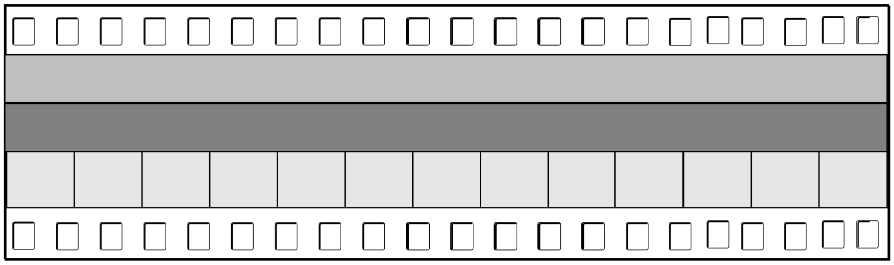

For a single-node object movie, the QTVR track contains just one sample. There is a corresponding sample in the object track, whose time and duration are the same as the time and duration of the sample in the QTVR track. The time base of the movie is used to locate the proper video samples in the object image track.

For an object movie, the frame corresponding to the first row and column in the object image array is located at the same time as the corresponding QTVR and object track samples. The total duration of all the video samples is the same as the duration of the corresponding QTVR sample and the object sample.

In addition to these three required tracks, an object movie can also contain a hot spot image track and any number of standard QuickTime tracks (such as video, sound, and text tracks). A hot spot image track for an object is a QuickTime video track that contains images of colored regions delineating the hot spots; an image in the hot spot image track must be synchronized to match the appropriate image in the object image track. A hot spot image track should be 8 bits deep and can be compressed with any lossless compressor (including temporal compressors). This is also true of panoramas.

Note: To assign a single fixed-position hot spot to all views of an object, you should create a hot spot image track that consists of a single video frame whose duration is the entire node time.

To play a time-based track with the object movie, you must synchronize the sample data of that track to the start and stop times of a view in the object image track. For example, to play a different sound with each view of an object, you might store a sound track in the movie file with each set of sound samples synchronized to play at the same time as the corresponding object's view image. (This technique also works for video samples.) Another way to add sound or video is simply to play a sound or video track during the object's view animation; to do this, you need to add an active track to the object that is equal in duration to the object's row duration.

Copyright © 2004, 2012 Apple Inc. All Rights Reserved.

> **Important:** In a QuickTime VR movie file, the panorama image tracks and panorama hot spot tracks must be disabled. For an object, the object image tracks must be enabled and the object hot spot tracks must be disabled.

Multinode Movies

A multinode QuickTime VR movie can contain any number of object and panoramic nodes. Figure 4-25 (page 287) illustrates the structure of a QuickTime VR movie that contains five nodes (in this case, three panoramic nodes and two object nodes).

Figure 4-25 The structure of a multinode movie file

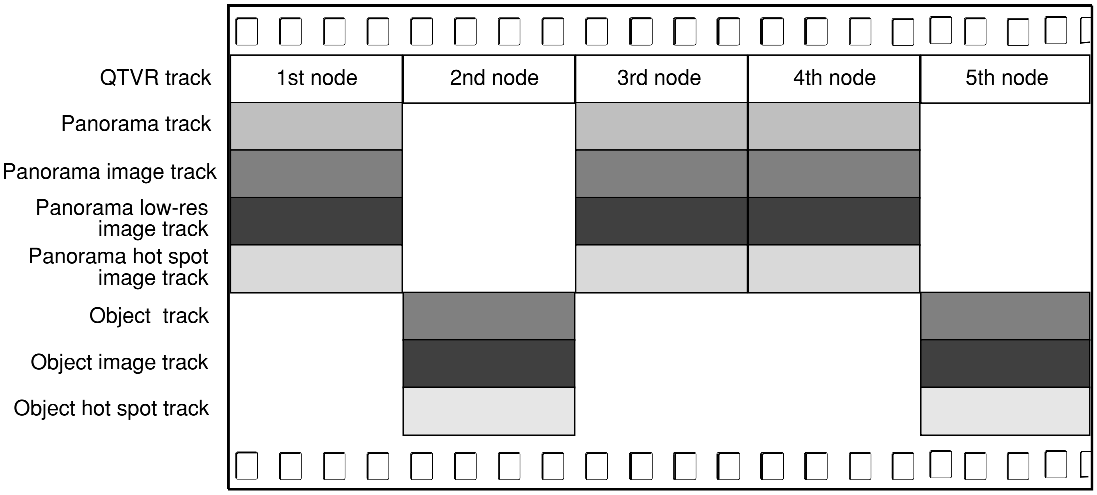

2012-08-14 | Copyright © 2004, 2012 Apple Inc. All Rights Reserved.

Important: Panoramic tracks and object tracks must never be located at the same time.

QTVR Track

Note: VR Media is deprecated in the QuickTime file format. The information that follows is intended to document existing content containing VR Media and should not be used for new development.

A QTVR track is a special type of QuickTime track that maintains a list of all the nodes in a movie. The media type for a QTVR track is `'qtvr'`. All the media samples in a QTVR track share a common sample description. This sample description contains the VR world atom container. The track contains one media sample for each node in the movie. Each QuickTime VR media sample contains a node information atom container.

QuickTime VR Sample Description Structure

Whereas the QuickTime VR media sample is simply the node information itself, all sample descriptions are required by QuickTime to have a certain structure for the first several bytes. The structure for the QuickTime VR sample description is as follows:

```
typedef struct QTVRSampleDescription {
    UInt32                              size;
    UInt32                              type;
    UInt32                              reserved1;
    UInt16                              reserved2;
    UInt16                              dataRefIndex;
    UInt32                              data;
} QTVRSampleDescription, *QTVRSampleDescriptionPtr, **QTVRSampleDescriptionHandle;
```

Field descriptions

`size`
: The size, in bytes, of the sample description header structure, including the VR world atom container contained in the `data` field.

`type`
: The sample description type. For QuickTime VR movies, this type should be `'qtvr'`.

`reserved1`
: Reserved. This field must be 0.

`reserved2`
: Reserved. This field must be 0.

`dataRefIndex`
: Reserved. This field must be 0.

`data`
: The VR world atom container. The sample description structure is extended to hold this atom container.

Panorama Tracks

Note: VR Media is deprecated in the QuickTime file format. The information that follows is intended to document existing content containing VR Media and should not be used for new development.

A movie's panorama track is a track that contains information about the panoramic nodes in a scene. The media type of the panorama track is `'pano'`. Each sample in a panorama track corresponds to a single panoramic node. This sample parallels the corresponding sample in the QTVR track. Panorama tracks do not have a sample description (although QuickTime requires that you specify a dummy sample description when you call `AddMediaSample` to add a sample to a panorama track). The sample itself contains an atom container that includes a panorama sample atom and other optional atoms.

Panorama Sample Atom Structure

A panorama sample atom has an atom type of `kQTVRPanoSampleDataAtomType` (`'pdat'`). It describes a single panorama, including track reference indexes of the scene and hot spot tracks and information about the default viewing angles and the source panoramic image.

The structure of a panorama sample atom is defined by the `QTVRPanoSampleAtom` data type:

```
typedef struct VRPanoSampleAtom {
    UInt16                              majorVersion;
    UInt16                              minorVersion;
    UInt32                              imageRefTrackIndex;
    UInt32                              hotSpotRefTrackIndex;
    Float32                             minPan;
    Float32                             maxPan;
```

Copyright © 2004, 2012 Apple Inc. All Rights Reserved.

```
    Float32                            minTilt;
    Float32                            maxTilt;
    Float32                            minFieldOfView;
    Float32                            maxFieldOfView;
    Float32                            defaultPan;
    Float32                            defaultTilt;
    Float32                            defaultFieldOfView;
    UInt32                             imageSizeX;
    UInt32                             imageSizeY;
    UInt16                             imageNumFramesX;
    UInt16                             imageNumFramesY;
    UInt32                             hotSpotSizeX;
    UInt32                             hotSpotSizeY;
    UInt16                             hotSpotNumFramesX;
    UInt16                             hotSpotNumFramesY;
    UInt32                             flags;
    OSType                             panoType;
    UInt32                             reserved2;
} VRPanoSampleAtom, *VRPanoSampleAtomPtr;
```

Field descriptions

`majorVersion`
The major version number of the file format.

`minorVersion`
The minor version number of the file format.

`imageRefTrackIndex`
The index of the image track reference. This is the index returned by the `AddTrackReference` function when the image track is added as a reference to the panorama track. There can be more than one image track for a given panorama track and hence multiple references. (A panorama track might have multiple image tracks if the panoramas have different characteristics, which could occur if the panoramas were shot with different size camera lenses.) The value in this field is 0 if there is no corresponding image track.

`hotSpotRefTrackIndex`
The index of the hot spot track reference.

Copyright © 2004, 2012 Apple Inc. All Rights Reserved.

`minPan`
The minimum pan angle, in degrees. For a full panorama, the value of this field is usually 0.0.

`maxPan`
The maximum pan angle, in degrees. For a full panorama, the value of this field is usually 360.0.

`minTilt`
The minimum tilt angle, in degrees. For a high-resolution panorama, a typical value for this field is –42.5.

`maxTilt`
The maximum tilt angle, in degrees. For a high-resolution panorama, a typical value for this field is +42.5.

`minFieldOfView`
The minimum vertical field of view, in degrees. For a high-resolution panorama, a typical value for this field is 5.0. The value in this field is 0 for the default minimum field of view, which is 5 percent of the maximum field of view.

`maxFieldOfView`
The maximum vertical field of view, in degrees. For a high-resolution panorama, a typical value for this field is 85.0. The value in this field is 0 for the default maximum field of view, which is `maxTilt` – `minTilt`.

`defaultPan`
The default pan angle, in degrees.

`defaultTilt`
The default tilt angle, in degrees.

`defaultFieldOfView`
The default vertical field of view, in degrees.

`imageSizeX`
The width, in pixels, of the panorama stored in the highest resolution image track.

`imageSizeY`
The height, in pixels, of the panorama stored in the highest resolution image track.

`imageNumFramesX`
The number of frames into which the panoramic image is diced horizontally. The width of each frame (which is `imageSizeX`/`imageNumFramesX`) should be divisible by 4.

`imageNumFramesY`
The number of frames into which the panoramic image is diced vertically. The height of each frame (which is `imageSizeY`/`imageNumFramesY`) should be divisible by 4.

`hotSpotSizeX`
The width, in pixels, of the panorama stored in the highest resolution hot spot image track.

`hotSpotSizeY`
The height, in pixels, of the panorama stored in the highest resolution hot spot image track.

2012-08-14 | Copyright © 2004, 2012 Apple Inc. All Rights Reserved.

`hotSpotNumFramesX`
: The number of frames into which the panoramic image is diced horizontally for the hot spot image track.

`hotSpotNumFramesY`
: The number of frames into which the panoramic image is diced vertically for the hot spot image track.

`flags`
: A set of panorama flags. `kQTVRPanoFlagHorizontal` has been superseded by the `panoType` field. It is used only when the `panoType` field is `nil` to indicate a horizontally-oriented cylindrical panorama. `kQTVRPanoFlagAlwaysWrap` is set if the panorama should wrap horizontally, regardless of whether or not the pan range is 360 degrees. Note that these flags are currently supported only under OS X.

`panoType`
: An `OSType` describing the type of panorama. Types supported are:

  `kQTVRHorizontalCylinder`

  `kQTVRVerticalCylinder`

  `kQTVRCube`

`reserved2`
: Reserved. This field must be 0.

> **Important:** A new flag has been added to the flags field of the `QTVRPanoSampleAtom` data structure. This flag controls how panoramas wrap horizontally. If `kQTVRPanoFlagAlwaysWrap` is set, then the panorama wraps horizontally, regardless of the number of degrees in the panorama. If the flag is not set, then the panorama wraps only when the panorama range is 360 degrees. This is the default behavior.

The minimum and maximum values in the panorama sample atom describe the physical limits of the panoramic image. QuickTime VR allows you to set further constraints on what portion of the image a user can see by calling the `QTVRSetConstraints` routine. You can also preset image constraints by adding constraint atoms to the panorama sample atom container. The three constraint atom types are `kQTVRPanConstraintAtomType`, `kQTVRTiltConstraintAtomType`, and `kQTVRFOVConstraintAtomType`. Each of these atom types share a common structure defined by the `QTVRAngleRangeAtom` data type:

```
typedef struct QTVRAngleRangeAtom {
    Float32                             minimumAngle;
    Float32                             maximumAngle;
} QTVRAngleRangeAtom, *QTVRAngleRangeAtomPtr;
```

Field descriptions

2012-08-14 | Copyright © 2004, 2012 Apple Inc. All Rights Reserved.

`minimumAngle`
The minimum angle in the range, in degrees.

`maximumAngle`
The maximum angle in the range, in degrees.

Panorama Image Track

The actual panoramic image for a panoramic node is contained in a panorama image track, which is a standard QuickTime video track. The track reference to this track is stored in the `imageRefTrackIndex` field of the panorama sample atom.

QuickTime VR 2.1 required the original panoramic image to be rotated 90 degrees counterclockwise. This orientation has changed in QuickTime VR 2.2, however, as discussed later in this section.

2012-08-14 | Copyright © 2004, 2012 Apple Inc. All Rights Reserved.

The rotated image is diced into smaller frames, and each diced frame is then compressed and added to the video track as a video sample, as shown in Figure 4-26 (page 294). Frames can be compressed using any spatial compressor; however, temporal compression is not allowed for panoramic image tracks.

Figure 4-26 Creating an image track for a panorama

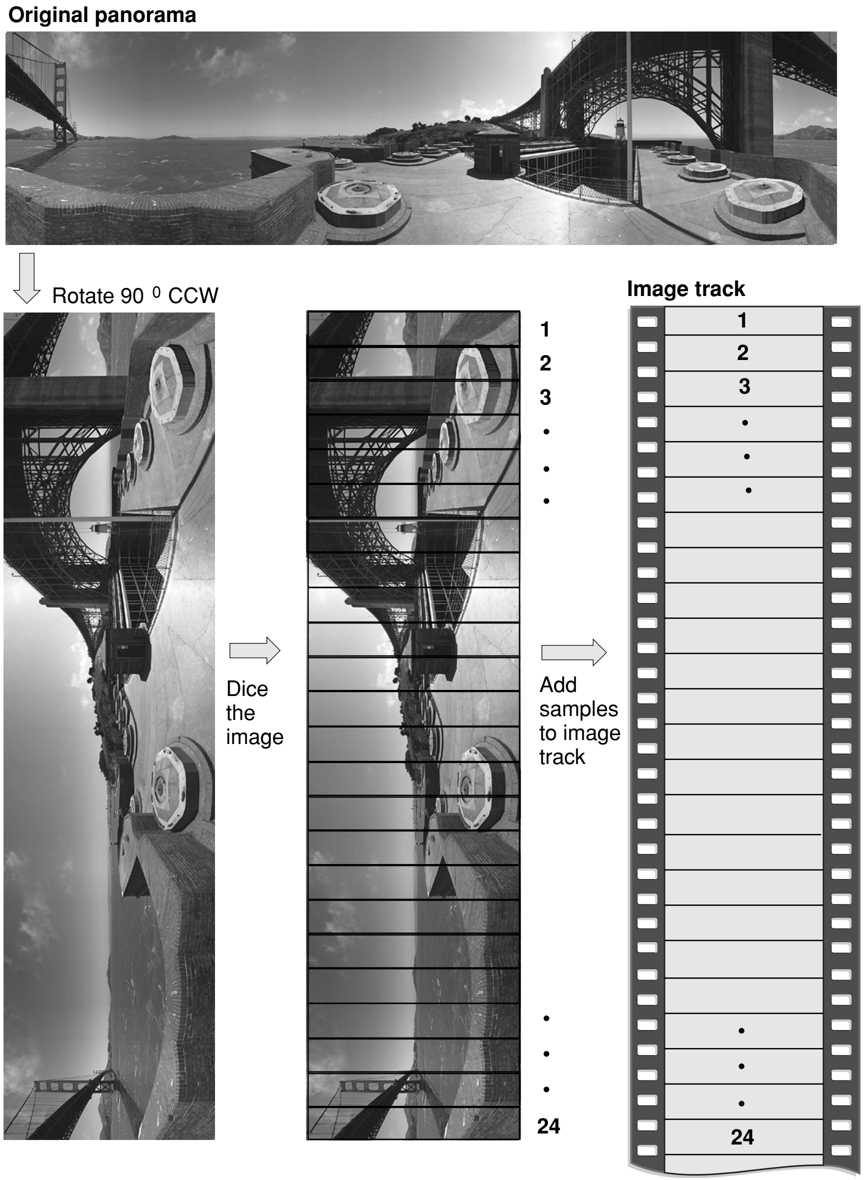

Copyright © 2004, 2012 Apple Inc. All Rights Reserved.

QuickTime VR 2.2 does not require the original panoramic image to be rotated 90 degrees counterclockwise, as was the case in QuickTime VR 2.1. The rotated image is still diced into smaller frames, and each diced frame is then compressed and added to the video track as a video sample, as shown in Figure 4-27 (page 295).

Figure 4-27 Creating an image track for a panorama, with the image track oriented horizontally

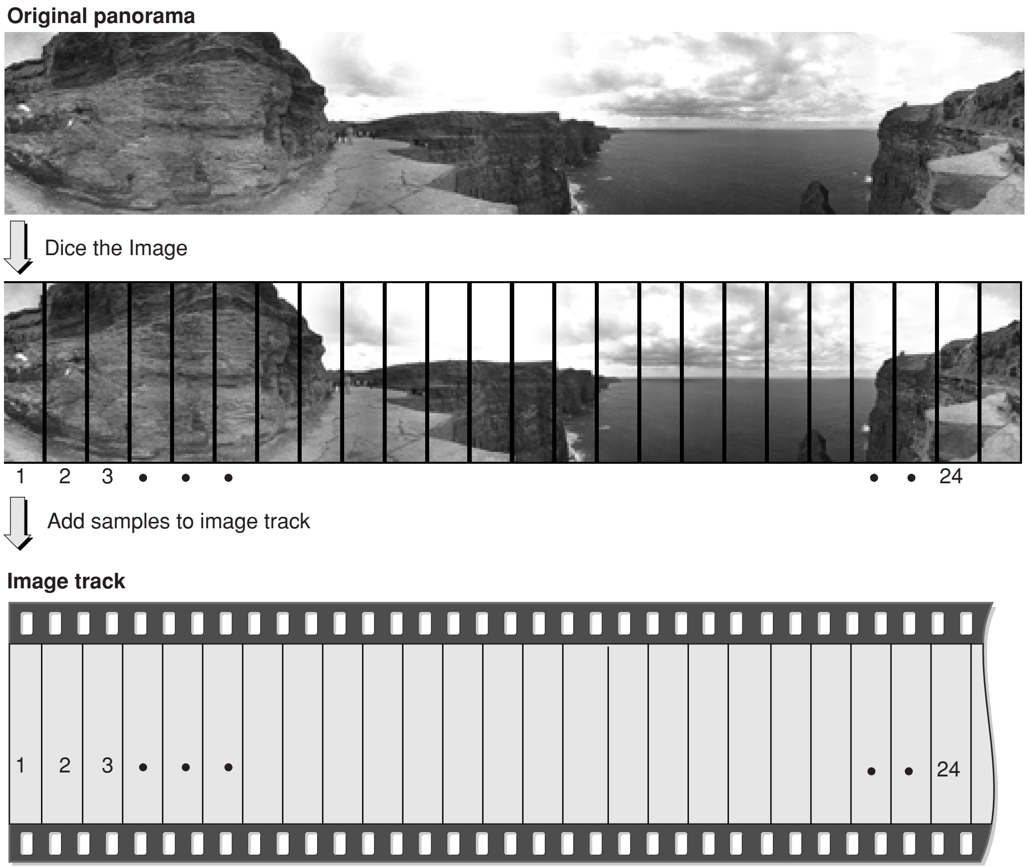

In QuickTime 3.0, a panorama sample atom (which contains information about a single panorama) contains the `panoType` field, which indicates whether the diced panoramic image is oriented horizontally or vertically.

Cylindrical Panoramas

The primary change to cylindrical panoramas in QuickTime VR 2.2 is that the panorama, as stored in the image track of the movie, can be oriented horizontally. This means that the panorama does not need to be rotated 90 degrees counterclockwise, as required previously.

To indicate a horizontal orientation, the field in the `VRPanoSampleAtom` data structure formerly called `reserved1` has been renamed `panoType`. Its type is `OSType`. The `panoType` field value for a horizontally oriented cylinder is `kQTVRHorizontalCylinder ('hcyl')`, while a vertical cylinder is

Copyright © 2004, 2012 Apple Inc. All Rights Reserved.

`kQTVRVerticalCylinder` (`'vcyl'`). For compatibility with older QuickTime VR files, when the `panoType` field is `nil`, then a cylinder is assumed, with the low order bit of the flags field set to 1 to indicate if the cylinder is horizontal and 0 if the cylinder is vertical.

One consequence of reorienting the panorama horizontally is that, when the panorama is divided into separate tiles, the order of the samples in the file is now the reverse of what it was for vertical cylinders. Since vertical cylinders were rotated 90 degrees counterclockwise, the first tile added to the image track was the rightmost tile in the panorama. For unrotated horizontal cylinders, the first tile added to the image track is the left-most tile in the panorama.

Cubic Panoramas

Note: VR Media is deprecated in the QuickTime file format. The information that follows is intended to document existing content containing VR Media and should not be used for new development.

A new type of panorama was introduced in the current version of QuickTime: the cubic panorama. This panorama in its simplest form is represented by six faces of a cube, thus enabling the viewer to see all the way up and all the way down. The file format and the cubic rendering engine actually allow for more complicated representations, such as special types of cubes with elongated sides or cube faces made up of separate tiles. Atoms that describe the orientation of each face allow for these nonstandard representations. If these atoms are not present, then the simplest representation is assumed. The following describes this simplest representation: a cube with six square sides.

Tracks in a cubic movie are laid out as they are for cylindrical panoramas. This includes a QTVR track, a panorama track, and an image track. Optionally, there may also be a hot spot track and a fast-start preview track. The image, hot spot, and preview tracks are all standard QuickTime video tracks.

2012-08-14 | Copyright © 2004, 2012 Apple Inc. All Rights Reserved.

Image Tracks in Cubic Nodes

For a cubic node the image track contains six samples that correspond to the six square faces of the cube. The same applies to hot spot and preview tracks. Figure 4-28 (page 297) shows how the order of samples in the track corresponds to the orientation of the cube faces.

Figure 4-28 Cubic node sample order versus cube face orientation

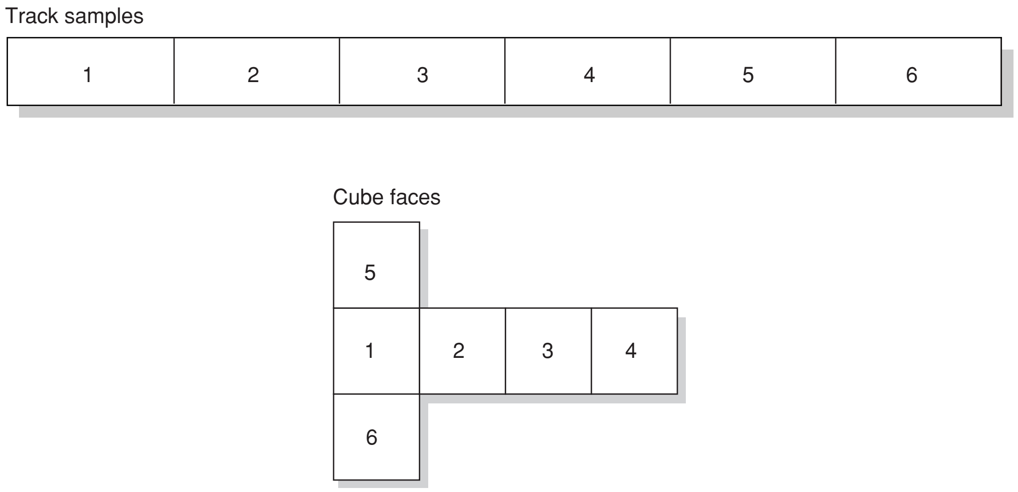

Note that the frames are oriented horizontally. There is no provision for frames that are rotated 90 counterclockwise as there are for cylindrical panoramas.

Panorama Tracks in Cubic Nodes

Note: VR Media is deprecated in the QuickTime file format. The information that follows is intended to document existing content containing VR Media and should not be used for new development.

The media sample for a panorama track contains the pano sample atom container. For cubes, some of the fields in the pano sample data atom have special values, which provide compatibility back to QuickTime VR 2.2. The cubic projection engine ignores these fields. They allow one to view cubic movies in older versions of QuickTime VR using the cylindrical engine, although the view will be somewhat incorrect, and the top and bottom faces will not be visible. The special values are shown in Table 4-26 (page 298).

Copyright © 2004, 2012 Apple Inc. All Rights Reserved.

Table 4-26 Fields and their special values as represented in the pano sample data atom, providing backward compatibility to QuickTime VR 2.2

Field	Value
`imageNumFramesX`	4
`imageNumFramesY`	1
`imageSizeX`	Frame width * 4
`imageSizeY`	Frame height
`minPan`	0.0
`maxPan`	360.0
`minTilt`	-45.0
`maxTilt`	45.0
`minFieldOfView`	5.0
`maxFieldOfView`	90.0
`flags`	1

A 1 value in the flags field tells QuickTime VR 2.2 that the frames are not rotated. QuickTime VR 2.2 treats this as a four-frame horizontal cylinder. The `panoType` field (formerly `reserved1`) must be set to `kQTVRCube` (`'cube'`) so that QuickTime VR 3.0 can recognize this panorama as a cube.

Since certain viewing fields in the pano sample data atom are being used for backward compatibility, a new atom must be added to indicate the proper viewing parameters for the cubic image. This atom is the cubic view atom (atom type `'cuvw'`). The data structure of the cubic view atom is as follows:

```
struct QTVRCubicViewAtom {
    Float32         minPan;
    Float32         maxPan;
    Float32         minTilt;
    Float32         maxTilt;
    Float32         minFieldOfView;
    Float32         maxFieldOfView;
```

Copyright © 2004, 2012 Apple Inc. All Rights Reserved.

```
    Float32         defaultPan;
    Float32         defaultTilt;
    Float32         defaultFieldOfView;
};
typedef struct QTVRCubicViewAtom    QTVRCubicViewAtom;
```

The fields are filled in as desired for the cubic image. This atom is ignored by older versions of QuickTime VR. Typical minimum and maximum field values are shown in Table 4-27 (page 299).

Table 4-27 Values for min and max fields

Field	Value
minPan	0.0
maxPan	360.0
minTilt	-90.0
maxTilt	90.0
minFieldOfView	5.0
maxFieldOfView	120.0

You add the cubic view atom to the pano sample atom container (after adding the pano sample data atom). Then use AddMediaSample to add the atom container to the panorama track.

Nonstandard Cubes

Note: VR Media is deprecated in the QuickTime file format. The information that follows is intended to document existing content containing VR Media and should not be used for new development.

Although the default representation for a cubic panorama is that of six square faces of a cube, it is possible to depart from this standard representation. When doing so, a new atom must be added to the pano sample atom container. The atom type is 'cufa'. The atom is an array of data structures of type QTVRCubicFaceData. Each entry in the array describes one face of whatever polyhedron is being defined. QTVRCubicFaceData is defined as follows:

Copyright © 2004, 2012 Apple Inc. All Rights Reserved.

```
struct QTVRCubicFaceData {
    float   orientation[4];
    float   center[2];
    float   aspect;
    float   skew;
};
typedef struct QTVRCubicFaceData    QTVRCubicFaceData;
```

The mathematical explanation of these data structures is beyond the scope of this document but will be described in a separate Apple Technote. Table 4-28 (page 300) shows what values QuickTime VR uses for the default representation of six square sides.

Table 4-28 Values used for representing sides

Orien-tation	Orien-tation	Orien-tation	Orien-tation	Center	Center	Aspect	Skew	Side
1	0	0	0	0	0	1	0	# front
–.5	0	.5	0	0	0	1	0	# right
0	0	1	0	0	0	1	0	# back
.5	0	.5	0	0	0	1	0	# left
.5	.5	0	0	0	0	1	0	# top
–.5	.5	0	0	0	0	1	0	# bottom

Hot Spot Image Tracks

Note: VR Media is deprecated in the QuickTime file format. The information that follows is intended to document existing content containing VR Media and should not be used for new development.

When a panorama contains hot spots, the movie file contains a hot spot image track, a video track that contains a parallel panorama, with the hot spots designated by colored regions. Each diced frame of the hot spot panoramic image must be compressed with a lossless compressor (such as QuickTime's graphics compressor).

2012-08-14 | Copyright © 2004, 2012 Apple Inc. All Rights Reserved.

The dimensions of the hot spot panoramic image are usually the same as those of the image track's panoramic image, but this is not required. The dimensions must, however, have the same aspect ratio as the image track's panoramic image. A hot spot image track should be 8 bits deep.

Low-Resolution Image Tracks

It's possible to store one or more low-resolution versions of a panoramic image in a movie file; those versions are called low-resolution image tracks. If there is not enough memory at runtime to use the normal image track, QuickTime VR uses a lower resolution image track if one is available. A low-resolution image track contains diced frames just like the higher resolution track, but the reconstructed panoramic image is half the height and half the width of the higher resolution image.

> **Important:** The panoramic images in the lower resolution image tracks and the hot spot image tracks, if present, must have the same orientation (horizontal or vertical) as the panorama image track.

Track Reference Entry Structure

Since there are no fields in the pano sample data atom to indicate the presence of low-resolution image tracks, a separate sibling atom must be added to the panorama sample atom container. The track reference array atom contains an array of track reference entry structures that specify information about any low-resolution image tracks contained in a movie. Its atom type is `kQTVRTrackRefArrayAtomType` (`'tref'`).

A track reference entry structure is defined by the `QTVRTrackRefEntry` data type:

```
typedef struct QTVRTrackRefEntry {
    UInt32                              trackRefType;
    UInt16                              trackResolution;
    UInt32                              trackRefIndex;
} QTVRTrackRefEntry;
```

Field descriptions

`trackRefType`
The track reference type.

`trackResolution`
The track resolution.

`trackRefIndex`
The index of the track reference.

2012-08-14 | Copyright © 2004, 2012 Apple Inc. All Rights Reserved.

The number of entries in the track reference array atom is determined by dividing the size of the atom by `sizeof` (`QTVRTrackRefEntry`).

`kQTVRPreviewTrackRes` is a special value for the `trackResolution` field in the `QTVRTrackRefEntry` structure. This is used to indicate the presence of a special preview image track.

Object Tracks

Note: VR Media is deprecated in the QuickTime file format. The information that follows is intended to document existing content containing VR Media and should not be used for new development.

A movie's object track is a track that contains information about the object nodes in a scene. The media type of the object track is `'obje'`. Each sample in an object track corresponds to a single object node in the scene. The samples of the object track contain information describing the object images stored in the object image track.

These object information samples parallel the corresponding node samples in the QTVR track and are equal in time and duration to a particular object node's image samples in the object's image track as well as the object node's hot spot samples in the object's hot spot track.

Object tracks do not have a sample description (although QuickTime requires that you specify a dummy sample description when you call `AddMediaSample` to add a sample to an object track). The sample itself is an atom container that contains a single object sample atom and other optional atoms.

Object Sample Atom Structure

object sample atom describes a single object, including information about the default viewing angles and the view settings. The structure of an object sample atom is defined by the `QTVRObjectSampleAtom` data type:

```
typedef struct VRObjectSampleAtom {
    UInt16                          majorVersion;
    UInt16                          minorVersion;
    UInt16                          movieType;
    UInt16                          viewStateCount;
    UInt16                          defaultViewState;
    UInt16                          mouseDownViewState;
    UInt32                          viewDuration;
```

Copyright © 2004, 2012 Apple Inc. All Rights Reserved.

```
    UInt32                              columns;
    UInt32                              rows;
    Float32                             mouseMotionScale;
    Float32                             minPan;
    Float32                             maxPan;
    Float32                             defaultPan;
    Float32                             minTilt;
    Float32                             maxTilt;
    Float32                             defaultTilt;
    Float32                             minFieldOfView;
    Float32                             fieldOfView;
    Float32                             defaultFieldOfView;
    Float32                             defaultViewCenterH;
    Float32                             defaultViewCenterV;
    Float32                             viewRate;
    Float32                             frameRate;
    UInt32                              animationSettings;
    UInt32                              controlSettings;
} VRObjectSampleAtom, *VRObjectSampleAtomPtr;
QT
QT
QT
```

Field descriptions

`majorVersion`
: The major version number of the file format.

`minorVersion`
: The minor version number of the file format.

`movieType`
: The movie controller type.

`viewStateCount`
: The number of view states of the object. A view state selects an alternate set of images for an object's views. The value of this field must be positive.

Copyright © 2004, 2012 Apple Inc. All Rights Reserved.

`defaultViewState`
: The 1-based index of the default view state. The default view state image for a given view is displayed when the mouse button is not down.

`mouseDownViewState`
: The 1-based index of the mouse-down view state. The mouse-down view state image for a given view is displayed while the user holds the mouse button down and the cursor is over an object movie.

`viewDuration`
: The total movie duration of all image frames contained in an object's view. In an object that uses a single frame to represent a view, the duration is the image track's sample duration time.

`columns`
: The number of columns in the object image array (that is, the number of horizontal positions or increments in the range defined by the minimum and maximum pan values). The value of this field must be positive.

`rows`
: The number of rows in the object image array (that is, the number of vertical positions or increments in the range defined by the minimum and maximum tilt values). The value of this field must be positive.

`mouseMotionScale`
: The mouse motion scale factor (that is, the number of degrees that an object is panned or tilted when the cursor is dragged the entire width of the VR movie image). The default value is 180.0.

`minPan`
: The minimum pan angle, in degrees. The value of this field must be less than the value of the `maxPan` field.

`maxPan`
: The maximum pan angle, in degrees. The value of this field must be greater than the value of the `minPan` field.

`defaultPan`
: The default pan angle, in degrees. This is the pan angle used when the object is first displayed. The value of this field must be greater than or equal to the value of the `minPan` field and less than or equal to the value of the `maxPan` field.

`minTilt`
: The minimum tilt angle, in degrees. The default value is +90.0. The value of this field must be less than the value of the `maxTilt` field.

`maxTilt`
: The maximum tilt angle, in degrees. The default value is –90.0. The value of this field must be greater than the value of the `minTilt` field.

2012-08-14 | Copyright © 2004, 2012 Apple Inc. All Rights Reserved.

`defaultTilt`
The default tilt angle, in degrees. This is the tilt angle used when the object is first displayed. The value of this field must be greater than or equal to the value of the `minTilt` field and less than or equal to the value of the `maxTilt` field.

`minFieldOfView`
The minimum field of view to which the object can zoom. The valid range for this field is from 1 to the value of the `fieldOfView` field. The value of this field must be positive.

`fieldOfView`
The image field of view, in degrees, for the entire object. The value in this field must be greater than or equal to the value of the `minFieldOfView` field.

`defaultFieldOfView`
The default field of view for the object. This is the field of view used when the object is first displayed. The value in this field must be greater than or equal to the value of the `minFieldOfView` field and less than or equal to the value of the `fieldOfView` field.

`defaultViewCenterH`
The default horizontal view center.

`defaultViewCenterV`
The default vertical view center.

`viewRate`
The view rate (that is, the positive or negative rate at which the view animation in the object plays, if view animation is enabled). The value of this field must be from –100.0 through +100.0, inclusive.

`frameRate`
The frame rate (that is, the positive or negative rate at which the frame animation in a view plays, if frame animation is enabled). The value of this field must be from –100.0 through +100.0, inclusive.

`animationSettings`
A set of 32-bit flags that encode information about the animation settings of the object.

`controlSettings`
A set of 32-bit flags that encode information about the control settings of the object.

The `movieType` field of the object sample atom structure specifies an object controller type, that is, the user interface to be used to manipulate the object.

QuickTime VR supports the following controller types:

```
enum ObjectUITypes {
    kGrabberScrollerUI                        = 1,
    kOldJoyStickUI                            = 2,
```

Copyright © 2004, 2012 Apple Inc. All Rights Reserved.

```
    kJoystickUI                         = 3,
    kGrabberUI                          = 4,
    kAbsoluteUI                         = 5
};
```

Constant Descriptions

`kGrabberScrollerUI`
The default controller, which displays a hand for dragging and rotation arrows when the cursor is along the edges of the object window.

`kOldJoyStickUI`
A joystick controller, which displays a joystick-like interface for spinning the object. With this controller, the direction of panning is reversed from the direction of the grabber.

`kJoystickUI`
A joystick controller, which displays a joystick-like interface for spinning the object. With this controller, the direction of panning is consistent with the direction of the grabber.

`kGrabberUI`
A grabber-only interface, which displays a hand for dragging but does not display rotation arrows when the cursor is along the edges of the object window.

`kAbsoluteUI`
An absolute controller, which displays a finger for pointing. The absolute controller switches views based on a row-and-column grid mapped into the object window.

Animation Settings

The `animationSettings` field of the object sample atom is a long integer that specifies a set of animation settings for an object node. Animation settings specify characteristics of the movie while it is playing. Use these constants to specify animation settings:

```
enum QTVRAnimationSettings {
    kQTVRObjectAnimateViewFramesOn      = (1 << 0),
    kQTVRObjectPalindromeViewFramesOn   = (1 << 1),
    kQTVRObjectStartFirstViewFrameOn    = (1 << 2),
    kQTVRObjectAnimateViewsOn           = (1 << 3),
    kQTVRObjectPalindromeViewsOn        = (1 << 4),
    kQTVRObjectSyncViewToFrameRate      = (1 << 5),
```

2012-08-14 | Copyright © 2004, 2012 Apple Inc. All Rights Reserved.

```
    kQTVRObjectDontLoopViewFramesOn            = (1 << 6),
    kQTVRObjectPlayEveryViewFrameOn            = (1 << 7)
};
```

Constant Descriptions

`kQTVRObjectAnimateViewFramesOn`
: The animation setting to play all frames in the current view state.

`kQTVRObjectPalindromeViewFramesOn`
: The animation setting to play a back-and-forth animation of the frames of the current view state.

`kQTVRObjectStartFirstViewFrameOn`
: The animation setting to play the frame animation starting with the first frame in the view (that is, at the view start time).

`kQTVRObjectAnimateViewsOn`
: The animation setting to play all views of the current object in the default row of views.

`kQTVRObjectPalindromeViewsOn`
: The animation setting to play a back-and-forth animation of all views of the current object in the default row of views.

`kQTVRObjectSyncViewToFrameRate`
: The animation setting to synchronize the view animation to the frame animation and use the same options as for frame animation.

`kQTVRObjectDontLoopViewFramesOn`
: The animation setting to stop playing the frame animation in the current view at the end.

`kQTVRObjectPlayEveryViewFrameOn`
: The animation setting to play every view frame regardless of play rate. The play rate is used to adjust the duration in which a frame appears but no frames are skipped so the rate is not exact.

Control Settings

The `controlSettings` field of the object sample atom is a long integer that specifies a set of control settings for an object node. Control settings specify whether the object can wrap during panning and tilting, as well as other features of the node. The control settings are specified using these bit flags:

```
enum QTVRControlSettings {
    kQTVRObjectWrapPanOn                       = (1 << 0),
    kQTVRObjectWrapTiltOn                      = (1 << 1),
    kQTVRObjectCanZoomOn                       = (1 << 2),
```

Copyright © 2004, 2012 Apple Inc. All Rights Reserved.

```
    kQTVRObjectReverseHControlOn            = (1 << 3),
    kQTVRObjectReverseVControlOn            = (1 << 4),
    kQTVRObjectSwapHVControlOn              = (1 << 5),
    kQTVRObjectTranslationOn                = (1 << 6)
};
```

Constant Descriptions

`kQTVRObjectWrapPanOn`
: The control setting to enable wrapping during panning. When this control setting is enabled, the user can wrap around from the current pan constraint maximum value to the pan constraint minimum value (or vice versa) using the mouse or arrow keys.

`kQTVRObjectWrapTiltOn`
: The control setting to enable wrapping during tilting. When this control setting is enabled, the user can wrap around from the current tilt constraint maximum value to the tilt constraint minimum value (or vice versa) using the mouse or arrow keys.

`kQTVRObjectCanZoomOn`
: The control setting to enable zooming. When this control setting is enabled, the user can change the current field of view using the zoom-in and zoom-out keys on the keyboard (or using the VR controller buttons).

`kQTVRObjectReverseHControlOn`
: The control setting to reverse the direction of the horizontal control.

`kQTVRObjectReverseVControlOn`
: The control setting to reverse the direction of the vertical control.

`kQTVRObjectSwapHVControlOn`
: The control setting to exchange the horizontal and vertical controls.

`kQTVRObjectTranslationOn`
: The control setting to enable translation. When this setting is enabled, the user can translate using the mouse when either the translate key is held down or the controller translation mode button is toggled on.

Copyright © 2004, 2012 Apple Inc. All Rights Reserved.

Track References for Object Tracks

> **Note:** VR Media is deprecated in the QuickTime file format. The information that follows is intended to document existing content containing VR Media and should not be used for new development.

The track references to an object's image and hot spot tracks are not handled the same way as track references to panoramas. The track reference types are the same (`kQTVRImageTrackRefType` and `kQTVRHotSpotTrackRefAtomType`), but the location of the reference indexes is different. There is no entry in the object sample atom for the track reference indexes. Instead, separate atoms using the `VRTrackRefEntry` structure are stored as siblings to the object sample atom. The types of these atoms are `kQTVRImageTrackRefAtomType` and `kQTVRHotSpotTrackRefAtomType`. If either of these atoms is not present, then the reference index to the corresponding track is assumed to be 1.

> **Note:** The `trackResolution` field in the `VRTrackRefEntry` structure is ignored for object tracks.

The actual views of an object for an object node are contained in an object image track, which is usually a standard QuickTime video track. (An object image track can also be any type of track that is capable of displaying an image, such as a QuickTime 3D track.)

As described in Chapter 1 of *QuickTime VR*, these views are often captured by moving a camera around the object in a defined pattern of pan and tilt angles. The views must then be ordered into an object image array, which is stored as a one-dimensional sequence of frames in the movie's video track (see Figure 4-29 (page 309)).

Figure 4-29 The structure of an image track for an object

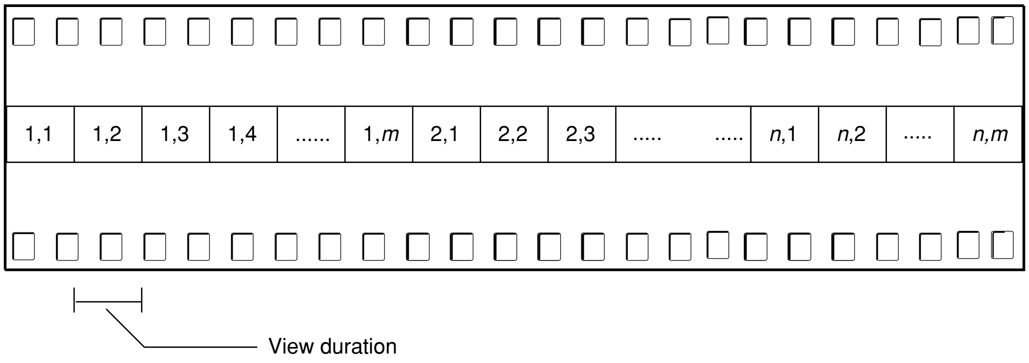

For object movies containing frame animation, each animated view in the object image array consists of the animating frames. It is not necessary that each view in the object image array contain the same number of frames, but the view duration of all views in the object movie must be the same.

2012-08-14 | Copyright © 2004, 2012 Apple Inc. All Rights Reserved.

For object movies containing alternate view states, alternate view states are stored as separate object image arrays that immediately follow the preceding view state in the object image track. Each state does not need to contain the same number of frames. However, the total movie time of each view state in an object node must be the same.

Movie Media

Movie media is used to encapsulate embedded movies within QuickTime movies. This feature is available in QuickTime 4.1.

Movie Sample Description

The movie media doesn't have a unique sample description. It uses the minimum sample description, which is `SampleDescriptionRecord`.

Movie Media Sample Format

Each sample in the movie media is a QuickTime atom container. All root-level atoms and their contents are enumerated in the following list. Note that the contents of all atoms are stored in big-endian format.

`kMovieMediaDataReference`

A data reference type and a data reference. The data reference type is stored as an `OSType` at the start of the atom. The data reference is stored following the data reference type. If the data reference type is URL and the data reference is for a movie on the Apple website, the contents of the atom would be `url http://www.apple.com/foo.mov`.

There may be more than one atom of this type. The first atom of this type should have an atom ID of 1. Additional data references should be numbered sequentially.

`kMovieMediaDefaultDataReferenceID`

This atom contains a `QTAtomID` that indicates the ID of the data reference to use when instantiating the embedded movie for this sample. If this atom is not present, the data reference with an ID of 1 is used.

`kMovieMediaSlaveTime`

A Boolean that indicates whether or not the `TimeBase` of the embedded movie should be slaved to the `TimeBase` of the parent movie. If the `TimeBase` is slaved, the embedded movie's zero time will correspond to the start time of its movie media sample. Further, the playback rate of the embedded movie will always be the same as the parent movie's. If the `TimeBase` is not slaved, the embedded movie will default to a rate of 0, and a default time of whatever default time value it instantiated with (which may not be 0). If the `TimeBase` is not slaved, the embedded movie can be played by either including an `AutoPlay` atom

2012-08-14 | Copyright © 2004, 2012 Apple Inc. All Rights Reserved.

in the movie media sample or by using a wired action. If this atom is not present, the embedded movie defaults to not slaved.

`kMovieMediaSlaveAudio`
A Boolean that indicates whether or not the audio properties of the embedded movie should be slaved to those of the parent movie. When audio is slaved, all audio properties of the containing track are duplicated in the embedded movie. These properties include sound volume, balance, bass and treble, and level metering. If this atom is not present, the embedded movie defaults to not slaved audio.

`kMovieMediaSlaveGraphicsMode`
A Boolean that indicates how the graphics mode of the containing track is applied to the embedded movie. If the graphics mode is not slaved, then the entire embedded movie is imaged using its own graphics modes. The result of the drawing of the embedded movie is composited onto the containing movie using the graphics mode of the containing track. If the graphics mode is slaved, then the graphics mode of each track in the embedded movie is ignored and instead the graphics mode of the containing track is used. In this case, the tracks of the embedded movie composite their drawing directly into the parent movie's contents. If this atom is not present, the graphics mode defaults to not slaved. Graphics mode slaving is useful for compositing semi-transparent media––for example, a PNG with an alpha channel––on top of other media.

`kMovieMediaSlaveTrackDuration`
A Boolean that indicates how the Movie Media Handler should react when the duration of the embedded movie is different than the duration of the movie media sample that it is contained by. When the movie media sample is created, the duration of the embedded movie may not yet be known. Therefore, the duration of the media sample may not be correct. In this case, the Movie Media Handler can do one of two things. If this atom is not present or it contains a value of false, the Movie Media Handler will respect the duration of media sample that contains the embedded movie. If the embedded movie has a longer duration than the movie media sample, the embedded movie will be truncated to the duration of the containing movie media sample. If the embedded movie is shorter, there will be a gap after it is finished playing. If this atom contains a value of true, the duration of the movie media sample will be adjusted to match the actual duration of the embedded movie. Because it is not possible to change an existing media sample, this will cause a new media sample to be added to the movie and the track's edit list to be updated to reference the new sample instead of the original sample.

2012-08-14 | Copyright © 2004, 2012 Apple Inc. All Rights Reserved.

Note: When the duration of the embedded movie's sample is adjusted, by default no other tracks are adjusted. This can cause the overall temporal composition to change in unintended ways. To maintain the complete temporal composition, a higher-level data structure which describes the temporal relationships between the various tracks must also be included with the movie.

`kMovieMediaAutoPlay`
: A Boolean that indicates whether or not the embedded movie should start playing immediately after being instantiated. This atom is only used if the `TimeBase` of the embedded movie is not slaved to the parent movie. See the `kMovieMediaSlaveTime` atom in "Movie Media Sample Format" (page 310) for more information. If auto play is requested, the movie will be played at its preferred rate after being instantiated. If this atom is not present, the embedded movie will not automatically play.

`kMovieMediaLoop`
: A `UInt8` that indicates how the embedded movie should loop. This atom is only used if the `TimeBase` of the embedded movie is not slaved to the parent movie. See the `kMovieMediaSlaveTime` atom in "Movie Media Sample Format" (page 310) for more information. If this atom contains a 0, or if this atom is not present, the embedded movie will not loop. If this atom contains a value of 1, the embedded movie loops normally—that is, when it reaches the end it loops back to the beginning. If this atom contains a value of 2, the embedded movie uses palindromic looping. All other values are reserved.

`kMovieMediaUseMIMEType`
: Text (not a C string or a pascal string) that indicates the MIME type of the movie import component that should be used to instantiate this media. This is useful in cases where the data reference may not contain MIME type information. If this atom is not present, the MIME type of the data reference as determined at instantiation time is used. This atom is intended to allow content creators a method for working around MIME type binding problems. It should not typically be required, and should not be included in movie media samples by default.

`kMovieMediaTitle`
: Currently unused. It would contain text indicating the name of the embedded movie.

`kMovieMediaAltText`
: Text (not a C string or a pascal string) that is displayed to the user when the embedded movie is being instantiated or if the embedded movie cannot be instantiated. If this atom is not present, the name of the data reference (typically the file name) is used.

`kMovieMediaClipBegin`
: A `MovieMediaTimeRecord` that indicates the time of the embedded movie that should be used. The clip begin atom provides a way to specify that a portion of the beginning of the embedded movie should not be used. If this atom is not present, the beginning of the embedded movie is not changed. Note that this atom does not change the time at which the embedded movie begins playing in the parent movie's

Copyright © 2004, 2012 Apple Inc. All Rights Reserved.

time line. If the time specified in the clip begin atom is greater than the duration of the embedded movie, then the embedded movie will not play at all.

```
struct MovieMediaTimeRecord {
 wide            time;
TimeScale       scale;
};
```

`kMovieMediaClipDuration`
A `MovieMediaTimeRecord` that indicates the duration of the embedded movie that should be used. The clip duration atom is applied by removing media from end of the embedded movie. If the clip duration atom is not present, then no media is removed from the end of the embedded movie. In situations where the sample contains both a clip duration and a clip begin atom, the clip begin is applied first. If the clip duration specifies a value that is larger than the duration of the embedded movie, no change is made to the embedded movie.

`kMovieMediaEnableFrameStepping`
A Boolean that indicates whether or not the embedded movie should be considered when performing step operations, specifically using the interesting time calls with the `nextTimeStep` flag. If this atom is not present or is set to `false`, the embedded movie is not included in step calculations. If the atom is set to `true`, it is included in step calculations.

`kMovieMediaBackgroundColor`
An `RGBColor` that is used for filling the background when the movie is being instantiated or when it fails to instantiate.

2012-08-14 | Copyright © 2004, 2012 Apple Inc. All Rights Reserved.

`kMovieMediaRegionAtom`

A number of child atoms, shown below, which describe how the Movie Media Handler should resize the embedded movie. If this atom is not present, the Movie Media Handler resizes the child movie to completely fill the containing track's box.

`kMovieMediaSpatialAdjustment`

This atom contains an `OSType` that indicates how the embedded movie should be scaled to fit the track box. If this atom is not present, the default value is `kMovieMediaFitFill`. These modes are all based on SMIL layout options.

`kMovieMediaFitClipIfNecessary`

If the media is larger than the track box, it will be clipped; if it is smaller, any additional area will be transparent.

`kMovieMediaFitFill`

The media will be scaled to completely fill the track box.

`kMovieMediaFitMeet`

The media is proportionally scaled so that it is entirely visible in the track box and fills the largest area possible without changing the aspect ratio.

`kMovieMediaFitSlice`

The media is scaled proportionally so that the smaller dimension is completely visible.

`kMovieMediaFitScroll`

Not currently implemented. It currently has the same behavior as `kMovieMediaFitClipIfNecessary`. When implemented, it will have the behavior described in the SMIL specification for a scrolling layout element.

`kMovieMediaRectangleAtom`

Four child atoms that define a rectangle. Not all child atoms must be present: top and left must both appear together, width and height must both appear together. The dimensions contained in this rectangle are used in place of the track box when applying the contents of the spatial adjustment atom. If the top and left are not specified, the top and left of the containing track's box are used. If the width and height

2012-08-14 | Copyright © 2004, 2012 Apple Inc. All Rights Reserved.

are not specified, the width and height of the containing track's box are used. Each child atom contains a `UInt32`.

`kMovieMediaTop`

If present, the top of the rectangle

`kMovieMediaLeft`

If present, the left boundary of the rectangle

`kMovieMediaWidth`

If present, width of rectangle

`kMovieMediaHeight`

If present, height of rectangle

2012-08-14 | Copyright © 2004, 2012 Apple Inc. All Rights Reserved.

Basic Data Types

This chapter describes a number of common data types that are used in QuickTime files.

Language Code Values

Some elements of a QuickTime file may be associated with a particular spoken language. To indicate the language associated with a particular object, the QuickTime file format uses either language codes from the Macintosh Script Manager or ISO language codes (as specified in *ISO 639-2/T*).

QuickTime stores language codes as unsigned 16-bit fields. All Macintosh language codes have a value that is less than 0x400 except for the single value 0x7FFF indicating an unspecified language. ISO language codes are three-character codes, and are stored inside the 16-bit language code field as packed arrays, as described in "ISO Language Codes" (page 319). If treated as an unsigned 16-bit integer, an ISO language code always has a value of 0x400 or greater unless the code is equal to the value 0x7FFF indicating an Unspecified Macintosh language code.

If the language is specified using a Macintosh language code, any associated text uses Macintosh text encoding.

If the language is specified using an ISO language code, any associated text uses Unicode text encoding. When Unicode is used, the text is in UTF-8 unless it starts with a byte-order-mark (BOM, 0xFEFF.), whereupon the text is in UTF-16. Both the BOM and the UTF-16 text should be big-endian.

2012-08-14 | Copyright © 2004, 2012 Apple Inc. All Rights Reserved.

Note: ISO language codes cannot be used for all elements of a QuickTime file. Currently, ISO language codes can be used *only for user data text*. All other elements, including text tracks, must be specified using Macintosh language codes.

Note: ISO 639-2/T codes do not distinguish between certain language variations. Use an extended language tag atom ('elng') to make these distinctions. For example, ISO 639-2T does not distinguish between traditional and simplified Chinese, so also use 'elng' with the value "zh-Hant" or "zh-Hans", respectively. See "Extended Language Tag Atom" (page 82).

Macintosh Language Codes

Table 5-1 (page 317) lists some of the Macintosh language codes supported by QuickTime.

Table 5-1 QuickTime language code values

Language	Value	Language	Value
English	0	Georgian	52
French	1	Moldavian	53
German	2	Kirghiz	54
Italian	3	Tajiki	55
Dutch	4	Turkmen	56
Swedish	5	Mongolian	57
Spanish	6	MongolianCyr	58
Danish	7	Pashto	59
Portuguese	8	Kurdish	60
Norwegian	9	Kashmiri	61
Hebrew	10	Sindhi	62
Japanese	11	Tibetan	63
Arabic	12	Nepali	64
Finnish	13	Sanskrit	65

2012-08-14 | Copyright © 2004, 2012 Apple Inc. All Rights Reserved.

Language	Value	Language	Value
Greek	14	Marathi	66
Icelandic	15	Bengali	67
Maltese	16	Assamese	68
Turkish	17	Gujarati	69
Croatian	18	Punjabi	70
Traditional Chinese	19	Oriya	71
Urdu	20	Malayalam	72
Hindi	21	Kannada	73
Thai	22	Tamil	74
Korean	23	Telugu	75
Lithuanian	24	Sinhala	76
Polish	25	Burmese	77
Hungarian	26	Khmer	78
Estonian	27	Lao	79
Lettish	28	Vietnamese	80
Latvian	28	Indonesian	81
Saami	29	Tagalog	82
Sami	29	MalayRoman	83
Faroese	30	MalayArabic	84
Farsi	31	Amharic	85
Russian	32	Galla	87
Simplified Chinese	33	Oromo	87
Flemish	34	Somali	88
Irish	35	Swahili	89

2012-08-14 | Copyright © 2004, 2012 Apple Inc. All Rights Reserved.

Language	Value	Language	Value
Albanian	36	Kinyarwanda	90
Romanian	37	Rundi	91
Czech	38	Nyanja	92
Slovak	39	Malagasy	93
Slovenian	40	Esperanto	94
Yiddish	41	Welsh	128
Serbian	42	Basque	129
Macedonian	43	Catalan	130
Bulgarian	44	Latin	131
Ukrainian	45	Quechua	132
Belarusian	46	Guarani	133
Uzbek	47	Aymara	134
Kazakh	48	Tatar	135
Azerbaijani	49	Uighur	136
AzerbaijanAr	50	Dzongkha	137
Armenian	51	JavaneseRom	138
Unspecified	32767		

ISO Language Codes

Because the language codes specified by ISO 639-2/T are three characters long, they must be packed to fit into a 16-bit field. The packing algorithm must map each of the three characters, which are always lowercase, into a 5-bit integer and then concatenate these integers into the least significant 15 bits of a 16-bit integer, leaving the 16-bit integer's most significant bit set to zero.

2012-08-14 | Copyright © 2004, 2012 Apple Inc. All Rights Reserved.

One algorithm for performing this packing is to treat each ISO character as a 16-bit integer. Subtract 0x60 from the first character and multiply by 2^10 (0x400), subtract 0x60 from the second character and multiply by 2^5 (0x20), subtract 0x60 from the third character, and add the three 16-bit values. This will result in a single 16-bit value with the three codes correctly packed into the 15 least significant bits and the most significant bit set to zero.

Example: The ISO language code `'jpn'` consists of the three hexadecimal values 0x6A, 0x70, 0x6E. Subtracting 0x60 from each value yields the values 0xA, 0x10, 0xE, as shown in Table 5-2 (page 320).

Table 5-2 5-bit values of UTF-8 characters

Character	UTF-8 code	5-bit value	Shifted value
j	0x6A	0xA (01010)	0x2800 (01010..........)
p	0x70	0x10 (10000)	0x200 (.....10000.....)
n	0x6E	0xE (01110)	0xE (..........01110)

The first value is shifted 10 bits to the left (multiplied by 0x400) and the second value is shifted 5 bits to the left (multiplied by 0x20). This yields the values 0x2800, 0x200, 0xE. When added, this results in the 16-bit packed language code value of 0x2A0E.

Calendar Date and Time Values

QuickTime movies store date and time information in Macintosh date format: a 32-bit value indicating the number of seconds that have passed since midnight January 1, 1904.

This value does not specify a time zone. Common practice is to use local time for the time zone where the value is generated.

It is strongly recommended that all calendar date and time values be stored using UTC time, so that all files have a time and date relative to the same time zone.

Matrices

QuickTime files use matrices to describe spatial information about many objects, such as tracks within a movie.

2012-08-14 | Copyright © 2004, 2012 Apple Inc. All Rights Reserved.

A transformation matrix defines how to map points from one coordinate space into another coordinate space. By modifying the contents of a transformation matrix, you can perform several standard graphics display operations, including translation, rotation, and scaling. The matrix used to accomplish two-dimensional transformations is described mathematically by a 3-by-3 matrix.

All values in the matrix are 32-bit fixed-point numbers divided as 16.16, except for the {u, v, w} column, which contains 32-bit fixed-point numbers divided as 2.30. Figure 5-1 (page 321) and Figure 5-2 (page 321) depict how QuickTime uses matrices to transform displayed objects.

Figure 5-1 How display matrices are used in QuickTime

$$\begin{bmatrix} x & y & 1 \end{bmatrix} \times \begin{bmatrix} a & b & u \\ c & d & v \\ t_x & t_y & w \end{bmatrix} = \begin{bmatrix} x' & y' & 1 \end{bmatrix}$$

Figure 5-2 Applying the transform

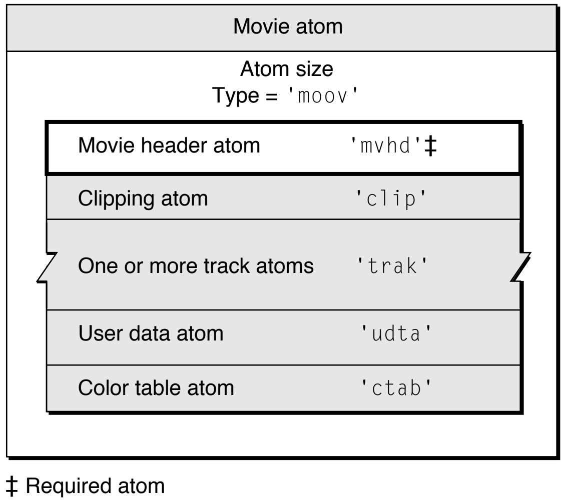

Graphics Modes

QuickTime files use graphics modes to describe how one video or graphics layer should be combined with the layers beneath it. Graphics modes are also known as transfer modes. Some graphics modes require a color to be specified for certain operations, such as blending to determine the blend level. QuickTime uses the graphics modes defined by Apple's QuickDraw.

The most common graphics modes are and `ditherCopy`, which simply indicate that the image should not blend with the image behind it, but overwrite it. QuickTime also defines several additional graphics modes.

Table 5-3 (page 322) lists the additional graphics modes supported by QuickTime.

2012-08-14 | Copyright © 2004, 2012 Apple Inc. All Rights Reserved.

Table 5-3 QuickTime graphics modes

Mode	Uses opcolor	Code	Description
Copy		0x0	Copy the source image over the destination.
Dither copy		0x40	Dither the image (if needed), otherwise do a copy.
Blend	yes	0x20	Replaces destination pixel with a blend of the source and destination pixel colors, with the proportion for each channel controlled by that channel in the opcolor.
Transparent	yes	0x24	Replaces the destination pixel with the source pixel if the source pixel isn't equal to the opcolor.
Straight alpha		0x100	Replaces the destination pixel with a blend of the source and destination pixels, with the proportion controlled by the alpha channel.
Premul white alpha		0x101	Premultiplied with white means that the color components of each pixel have already been blended with a white pixel, based on their alpha channel value. Effectively, this means that the image has already been combined with a white background. First, remove the white from each pixel and then blend the image with the actual background pixels.
Premul black alpha		0x102	Premultiplied with black is the same as pre-multiplied with white, except the background color that the image has been blended with is black instead of white.
Straight alpha blend	yes	0x104	Similar to straight alpha, but the alpha value used for each channel is the combination of the alpha channel and that channel in the opcolor.
Composition (dither copy)		0x103	(Tracks only) The track is drawn offscreen, and then composed onto the screen using dither copy

RGB Colors

Many atoms in the QuickTime file format contain RGB color values. These are usually stored as three consecutive unsigned 16-bit integers in the following order: red, green, blue.

2012-08-14 | Copyright © 2004, 2012 Apple Inc. All Rights Reserved.

Balance

Balance values are represented as 16-bit, fixed-point numbers that range from -1.0 to +1.0. The high-order 8 bits contain the integer portion of the value; the low-order 8 bits contain the fractional part. Negative values weight the balance toward the left speaker; positive values emphasize the right channel. Setting the balance to 0 corresponds to a neutral setting.

2012-08-14 | Copyright © 2004, 2012 Apple Inc. All Rights Reserved.

Some Useful Examples and Scenarios

This chapter contains a number of examples that can help you pull together all of the material in this book by examining the atom structure that results from a number of different scenarios.

The chapter is divided into the following topics:

- "Creating, Copying, and Disposing of Atom Containers" (page 325) discusses the various ways you can work with atom containers, along with illustrations and sample code that show usage.
- "Preparing Sound and Subtitle Alternate Groups for Use with Apple Devices" (page 334) discusses how multiple tracks with different languages can be associated with each other.
- "Creating an Effect Description" (page 337) discusses how you create an effect description by creating an atom container, inserting a QT atom that specifies the effect, and inserting a set of QT atoms that set its parameters.
- "Creating Movies with Modifier Tracks" (page 343) provides sample code showing you how to create a movie with modifier tracks.
- "Authoring Movies with External Movie Targets" (page 345) discusses how to author movies with external targets, using two new target atoms introduced in QuickTime 4.
- "Adding Wired Actions To a Flash Track" (page 347) explains the steps you need to follow in order to add wired actions to a Macromedia Flash track.
- "Creating Video Tracks at 30 Frames per Second" (page 349) discusses creating 30 fps video.
- "Creating Video Tracks at 29.97 Frames per Second" (page 350) describes creating 29.97 fps video.
- "Creating Sound Tracks at 44.1 kHz" (page 351) provides an example of creating a sound track.
- "Creating a Timecode Track for 29.97 FPS Video" (page 352) presents a timecode track example.
- "Playing with Edit Lists" (page 356) discusses how to interpret edit list data.
- "Interleaving Movie Data" (page 358) shows how a movie's tracks are interleaved in the movie data file.
- "Referencing Two Data Files With a Single Track" (page 359) shows how track data may reside in more than one file.
- "Getting the Name of a QuickTime VR Node" (page 361) discusses how you can use standard QuickTime atom container functions to retrieve the information in a QuickTime VR node header atom.
- "Adding Custom Atoms in a QuickTime VR Movie" (page 363) describes how to add custom atoms to either the QuickTime VR world or node information atom containers.

2012-08-14 | Copyright © 2004, 2012 Apple Inc. All Rights Reserved.

- "Adding Atom Containers in a QuickTime VR Movie" (page 364) shows the code you would use to add VR world and node information atom containers to a QTVR track.
- "Optimizing QuickTime VR Movies for Web Playback" (page 365) describes how to use the QTVR Flattener, a movie export component that converts an existing QuickTime VR single node movie into a new movie that is optimized for viewing on the Web.

Creating, Copying, and Disposing of Atom Containers

Before you can add atoms to an atom container, you must first create the container by calling `QTNewAtomContainer`. The code sample shown in Listing 6-1 (page 325) calls `QTNewAtomContainer` to create an atom container.

Listing 6-1 Creating a new atom container

```
QTAtomContainer spriteData;
OSErr err
// create an atom container to hold a sprite's data
err=QTNewAtomContainer (&spriteData);
```

When you have finished using an atom container, you should dispose of it by calling the `QTDisposeAtomContainer` function. The sample code shown in Listing 6-2 (page 325) calls `QTDisposeAtomContainer` to dispose of the `spriteData` atom container.

Listing 6-2 Disposing of an atom container

```
if (spriteData)
    QTDisposeAtomContainer (spriteData);
```

Creating New Atoms

You can use the `QTInsertChild` function to create new atoms and insert them in a QT atom container. The `QTInsertChild` function creates a new child atom for a parent atom. The caller specifies an atom type and atom ID for the new atom. If you specify a value of 0 for the atom ID, `QTInsertChild` assigns a unique ID to the atom.

`QTInsertChild` inserts the atom in the parent's child list at the index specified by the `index` parameter; any existing atoms at the same index or greater are moved toward the end of the child list. If you specify a value of 0 for the `index` parameter, `QTInsertChild` inserts the atom at the end of the child list.

Copyright © 2004, 2012 Apple Inc. All Rights Reserved.

The code sample in Listing 6-3 (page 326) creates a new QT atom container and calls `QTInsertChild` to add an atom. The resulting QT atom container is shown in Figure 6-1 (page 326). The offset value 10 is returned in the `firstAtom` parameter.

Listing 6-3 Creating a new QT atom container and calling `QTInsertChild` to add an atom.

```
QTAtom firstAtom;
QTAtomContainer container;
OSErr err
err = QTNewAtomContainer (&container);
if (!err)
    err = QTInsertChild (container, kParentAtomIsContainer, 'abcd',
        1000, 1, 0, nil, &firstAtom);
```

Figure 6-1 QT atom container after inserting an atom

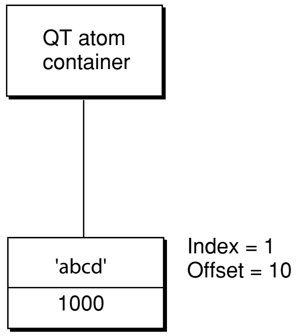

The following code sample calls `QTInsertChild` to create a second child atom. Because a value of 1 is specified for the `index` parameter, the second atom is inserted in front of the first atom in the child list; the index of the first atom is changed to 2. The resulting QT atom container is shown in Figure 6-2 (page 327).

```
QTAtom secondAtom;

FailOSErr (QTInsertChild (container, kParentAtomIsContainer, 'abcd',
```

Copyright © 2004, 2012 Apple Inc. All Rights Reserved.

```
    2000, 1, 0, nil, &secondAtom));
```

Figure 6-2 QT atom container after inserting a second atom

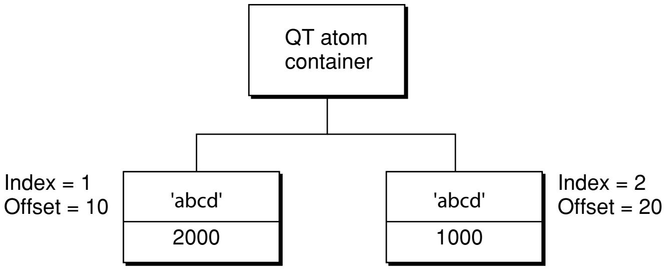

You can call the `QTFindChildByID` function to retrieve the changed offset of the first atom that was inserted, as shown in the following example. In this example, the `QTFindChildByID` function returns an offset of 20.

```
firstAtom = QTFindChildByID (container, kParentAtomIsContainer,  'abcd',
    1000, nil);
```

Listing 6-4 (page 327) shows how the `QTInsertChild` function inserts a leaf atom into the atom container `sprite`. The new leaf atom contains a sprite image index as its data.

Listing 6-4 Inserting a child atom

```
if ((propertyAtom = QTFindChildByIndex (sprite, kParentAtomIsContainer,
    kSpritePropertyImageIndex, 1, nil)) == 0)

    FailOSErr (QTInsertChild (sprite, kParentAtomIsContainer,
        kSpritePropertyImageIndex, 1, 1, sizeof(short),&imageIndex,
        nil));
```

Copying Existing Atoms

QuickTime provides several functions for copying existing atoms within an atom container. The `QTInsertChildren` function inserts a container of atoms as children of a parent atom in another atom container. Figure 6-3 (page 328) shows two example QT atom containers, A and B.

Figure 6-3 Two QT atom containers, A and B

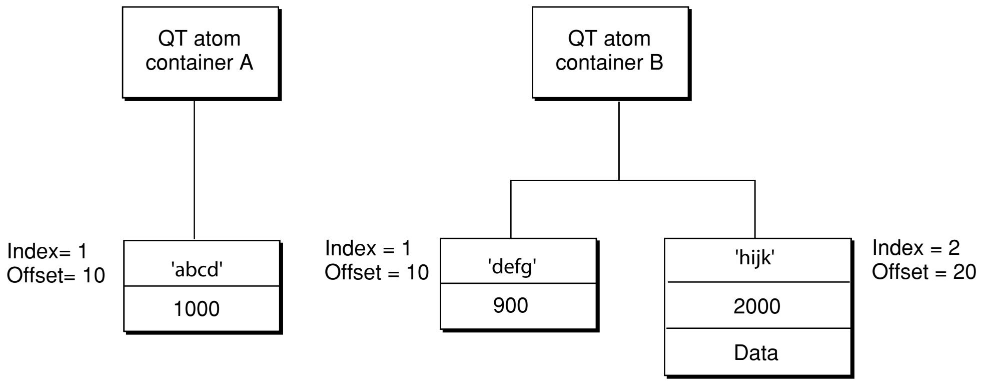

The following code sample calls `QTFindChildByID` to retrieve the offset of the atom in container A. Then, the code sample calls the `QTInsertChildren` function to insert the atoms in container B as children of the atom in container A. Figure 6-4 (page 329) shows what container A looks like after the atoms from container B have been inserted.

```
QTAtom targetAtom;

targetAtom = QTFindChildByID (containerA, kParentAtomIsContainer,  'abcd',
    1000, nil);
```

Copyright © 2004, 2012 Apple Inc. All Rights Reserved.

```
FailOSErr (QTInsertChildren (containerA, targetAtom, containerB));
```

Figure 6-4 QT atom container after child atoms have been inserted

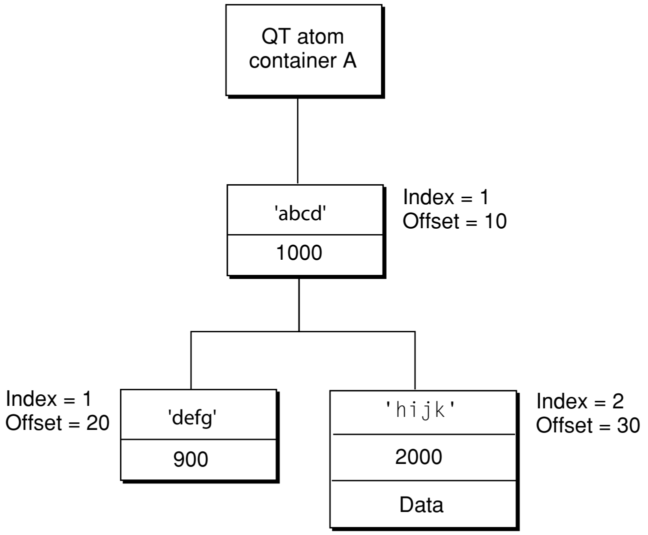

In Listing 6-5 (page 329), the `QTInsertChild` function inserts a parent atom into the atom container `theSample`. Then, the code calls `QTInsertChildren` to insert the container `theSprite` into the container `theSample`. The parent atom is `newSpriteAtom`.

Listing 6-5 Inserting a container into another container

```
FailOSErr (QTInsertChild (theSample, kParentAtomIsContainer,
    kSpriteAtomType, spriteID, 0, 0, nil, &newSpriteAtom));

FailOSErr (QTInsertChildren (theSample, newSpriteAtom, theSprite));
```

QuickTime provides three other functions you can use to manipulate atoms in an atom container. The `QTReplaceAtom` function replaces an atom and its children with a different atom and its children. You can call the `QTSwapAtoms` function to swap the contents of two atoms in an atom container; after swapping, the ID and index of each atom remains the same. The `QTCopyAtom` function copies an atom and its children to a new atom container.

Retrieving Atoms From an Atom Container

QuickTime provides functions you can use to retrieve information about the types of a parent atom's children, to search for a specific atom, and to retrieve a leaf atom's data.

Copyright © 2004, 2012 Apple Inc. All Rights Reserved.

You can use the QTCountChildrenOfType and QTGetNextChildType functions to retrieve information about the types of an atom's children. The QTCountChildrenOfType function returns the number of children of a given atom type for a parent atom. The QTGetNextChildType function returns the next atom type in the child list of a parent atom.

You can use the QTFindChildByIndex, QTFindChildByID, and QTNextChildAnyType functions to retrieve an atom. You call the QTFindChildByIndex function to search for and retrieve a parent atom's child by its type and index within that type.

Listing 6-6 (page 330) shows the sample code function SetSpriteData, which updates an atom container that describes a sprite. (For more information about sprites and the Sprite Toolbox, refer to the book Programming With Wired Movies and Sprite Animation, available at http://developer.apple.com/techpubs/quicktime/qtdevdocs/RM/PDF.htm.) For each property of the sprite that needs to be updated, SetSpriteData calls QTFindChildByIndex to retrieve the appropriate atom from the atom container. If the atom is found, SetSpriteData calls QTSetAtomData to replace the atom's data with the new value of the property. If the atom is not found, SetSpriteData calls QTInsertChild to add a new atom for the property.

Listing 6-6 Finding a child atom by index

```
OSErr SetSpriteData (QTAtomContainer sprite, Point *location,
    short *visible, short *layer, short *imageIndex)
{
    OSErr err = noErr;
    QTAtom propertyAtom;

    // if the sprite's visible property has a new value
    if (visible)
    {
        // retrieve the atom for the visible property --
        // if none exists, insert one
        if ((propertyAtom = QTFindChildByIndex (sprite,
            kParentAtomIsContainer, kSpritePropertyVisible, 1,
            nil)) == 0)
            FailOSErr (QTInsertChild (sprite, kParentAtomIsContainer,
                kSpritePropertyVisible, 1, 1, sizeof(short), visible,
                nil))
```

Copyright © 2004, 2012 Apple Inc. All Rights Reserved.

```
        // if an atom does exist, update its data
        else
            FailOSErr (QTSetAtomData (sprite, propertyAtom,
                sizeof(short), visible));
    }

    // ...
    // handle other sprite properties
    // ...
}
```

You can call the QTFindChildByID function to search for and retrieve a parent atom's child by its type and ID. The sample code function AddSpriteToSample, shown in Listing 6-7 (page 331), adds a sprite, represented by an atom container, to a key sample, represented by another atom container. AddSpriteToSample calls QTFindChildByID to determine whether the atom container theSample contains an atom of type kSpriteAtomType with the ID spriteID. If not, AddSpriteToSample calls QTInsertChild to insert an atom with that type and ID. A value of 0 is passed for the index parameter to indicate that the atom should be inserted at the end of the child list. A value of 0 is passed for the dataSize parameter to indicate that the atom does not have any data. Then, AddSpriteToSample calls QTInsertChildren to insert the atoms in the container theSprite as children of the new atom. FailIf and FailOSErr are macros that exit the current function when an error occurs.

Listing 6-7 Finding a child atom by ID

```
OSErr AddSpriteToSample (QTAtomContainer theSample,
    QTAtomContainer theSprite, short spriteID)
{
    OSErr err = noErr;
    QTAtom newSpriteAtom;

    FailIf (QTFindChildByID (theSample, kParentAtomIsContainer,
        kSpriteAtomType, spriteID, nil), paramErr);

    FailOSErr (QTInsertChild (theSample, kParentAtomIsContainer,
        kSpriteAtomType, spriteID, 0, 0, nil, &newSpriteAtom));
    FailOSErr (QTInsertChildren (theSample, newSpriteAtom, theSprite));
}
```

Copyright © 2004, 2012 Apple Inc. All Rights Reserved.

Once you have retrieved a child atom, you can call QTNextChildAnyType function to retrieve subsequent children of a parent atom. QTNextChildAnyType returns an offset to the next atom of any type in a parent atom's child list. This function is useful for iterating through a parent atom's children quickly.

QuickTime also provides functions for retrieving an atom's type, ID, and data. You can call QTGetAtomTypeAndID function to retrieve an atom's type and ID. You can access an atom's data in one of three ways.

- To copy an atom's data to a handle, you can use the QTCopyAtomDataToHandle function.
- To copy an atom's data to a pointer, you can use the QTCopyAtomDataToPtr function.
- To access an atom's data directly, you should lock the atom container in memory by calling QTLockContainer. Once the container is locked, you can call QTGetAtomDataPtr to retrieve a pointer to an atom's data. When you have finished accessing the atom's data, you should call the QTUnlockContainer function to unlock the container in memory.

Modifying Atoms

QuickTime provides functions that you can call to modify attributes or data associated with an atom in an atom container. To modify an atom's ID, you call the function QTSetAtomID.

You use the QTSetAtomData function to update the data associated with a leaf atom in an atom container. The QTSetAtomData function replaces a leaf atom's data with new data. The code sample in Listing 6-8 (page 332) calls QTFindChildByIndex to determine whether an atom container contains a sprite's visible property. If so, the sample calls QTSetAtomData to replace the atom's data with a new visible property.

Listing 6-8 Modifying an atom's data

```
QTAtom propertyAtom;

// if the atom isn't in the container, add it
if ((propertyAtom = QTFindChildByIndex (sprite, kParentAtomIsContainer,
    kSpritePropertyVisible, 1, nil)) == 0)
    FailOSErr (QTInsertChild (sprite, kParentAtomIsContainer,
        kSpritePropertyVisible, 1, 0, sizeof(short), visible, nil))

// if the atom is in the container, replace its data
else
    FailOSErr (QTSetAtomData (sprite, propertyAtom, sizeof(short),
        visible));
```

Copyright © 2004, 2012 Apple Inc. All Rights Reserved.

Removing Atoms From an Atom Container

To remove atoms from an atom container, you can use the `QTRemoveAtom` and `QTRemoveChildren` functions. The `QTRemoveAtom` function removes an atom and its children, if any, from a container. The `QTRemoveChildren` function removes an atom's children from a container, but does not remove the atom itself. You can also use `QTRemoveChildren` to remove all the atoms in an atom container. To do so, you should pass the constant `kParentAtomIsContainer` for the `atom` parameter.

The code sample shown in Listing 6-9 (page 333) adds override samples to a sprite track to animate the sprites in the sprite track. The `sample` and `spriteData` variables are atom containers. The `spriteData` atom container contains atoms that describe a single sprite. The `sample` atom container contains atoms that describes an override sample.

Each iteration of the `for` loop calls `QTRemoveChildren` to remove all atoms from both the `sample` and the `spriteData` containers. The sample code updates the index of the image to be used for the sprite and the sprite's location and calls `SetSpriteData` (Listing 6-6 (page 330)), which adds the appropriate atoms to the `spriteData` atom container. Then, the sample code calls `AddSpriteToSample` (Listing 6-7 (page 331)) to add the `spriteData` atom container to the `sample` atom container. Finally, when all the sprites have been updated, the sample code calls `AddSpriteSampleToMedia` to add the override sample to the sprite track.

Listing 6-9 Removing atoms from a container

```
QTAtomContainer sample, spriteData;

// ...
// add the sprite key sample
// ...

// add override samples to make the sprites spin and move
for (i = 1; i <= kNumOverrideSamples; i++)
{
    QTRemoveChildren (sample, kParentAtomIsContainer);
    QTRemoveChildren (spriteData, kParentAtomIsContainer);

    // ...
    // update the sprite:
    // - update the imageIndex
    // - update the location
    // ...
```

2012-08-14 | Copyright © 2004, 2012 Apple Inc. All Rights Reserved.

```
    // add atoms to spriteData atom container
    SetSpriteData (spriteData, &location, nil, nil, &imageIndex);

    // add the spriteData atom container to sample
    err = AddSpriteToSample (sample, spriteData, 2);

    // ...
    // update other sprites
    // ...

    // add the sample to the media
    err = AddSpriteSampleToMedia (newMedia, sample,
        kSpriteMediaFrameDuration, false);
}
```

Preparing Sound and Subtitle Alternate Groups for Use with Apple Devices

Alternate groups are collections of tracks that all serve the same purpose, where any track in the group can be substituted for another in a movie. Members of the same alternate group have the same identifier value in the alternate group field in the `'tkhd'` (track header) atom; see "Track Reference Atoms" (page 73) for an example of alternate tracks for different languages.

This section provides guidelines for the use of alternate groups in movies to be played on Apple devices.

General

For each alternate group:

- The group must contain tracks of only one type; for example, only subtitle tracks or only sound tracks.
- All tracks of the same type must be in a single alternate group.
- One track in the group must be enabled; that is, the Track Enabled flag must be set (0x0001) in its track header (`'tkhd'`).
- All other tracks in the group must be disabled.

Copyright © 2004, 2012 Apple Inc. All Rights Reserved.

Alternate Subtitle Tracks

For an alternate group that contains multiple subtitle ('sbtl') tracks:

- The player must pick a subtitle track even when subtitle display is turned off.
- If any of the requirements stated in "General" (page 334) does not hold, players may act as though there is no alternate subtitle track information.

As described in "Subtitle Sample Data" (page 202), a subtitle track can contain any combination of forced (must be displayed) and non-forced (optionally displayed) subtitle samples, including the possibility of a track that contains only forced subtitle samples. Even if a user indicates, directly or indirectly, that no subtitles should be displayed, any available appropriate subtitle track should be enabled so that any forced subtitles in the track can be displayed. A means by which a player can make a default selection among subtitle alternates in the absence of a user selection is covered in "Relationships Across Alternate Groups" (page 336).

For each language in a subtitle alternate group, subtitle tracks can be configured in either of the following ways:

- Single track: Contains any combination of forced and non-forced (regular) subtitle samples.
- Track pairs: One track contains any combination of forced and non-forced (regular) subtitle samples and has a track reference of type 'forc' that references the second track, which contains only forced samples, as described in "Subtitle Sample Data" (page 202).

If the user or player picks the language of a track pair, the player application then selects the appropriate track of the pair. It must select the regular subtitle track if subtitle display is on or the forced-only subtitle track if subtitle display is off.

If a track pair is present in a subtitle alternate group, a player that displays the track languages of subtitle tracks may choose to list the language only once. If a player lists both tracks of the pair, the player should display some kind of distinction between the tracks (for example, including the track names from the 'tnam' user data in a menu).

Alternate Sound Tracks

For an alternate group that contains multiple sound ('soun') tracks:

- If more than one alternate group contains sound tracks, the alternate group that contains the enabled sound track should be considered to be the primary alternate sound track group. Other alternate groups containing sound tracks should be ignored.
- If there is more than one enabled sound track, or if there is an enabled sound track and a non-sound track inside the same alternate group, the player may act as though there is no alternate sound track information.
- Players may ignore sound tracks that use codecs that are unavailable.

2012-08-14 | Copyright © 2004, 2012 Apple Inc. All Rights Reserved.

Relationships Across Alternate Groups

An audio alternate group and subtitle alternate group may need to use different languages because the first is the spoken language and the second is the written language and the BCP 47 language tags may differ. If there is not a need to differentiate, tracks in both alternate groups should use the same language code and optionally extended language tag.

Sound tracks can have a track reference of type `'folw'` (for "follows") to a single subtitle track; this track is the default to select if the sound track is selected. Use of a `'folw'` reference is a fallback in cases where the language tagging cannot be used for some reason (for example, the written language and spoken language use different extended language tags, as with Norwegian).

All subtitle tracks found in a subtitle track alternate group are candidate tracks for any chosen sound track. No facility to restrict the set of available subtitle tracks for a particular selected sound track is currently defined. However, to guide a player's behavior in making a selection among subtitle alternates in the absence of a user selection or preference, a single subtitle track may be associated with a sound track as follows:

- A sound track may include a `'folw'` ("follows") track reference to a corresponding subtitle track.
- If a sound track has a `'folw'` track reference to a subtitle track, that referenced subtitle track is the default subtitle track for that sound track.
- Each sound track can have either zero or one 'folw' track references to a subtitle track.
- If a subtitle track pair is to be made the default, the sound track should have a `'folw'` track reference to the forced subtitle track of the track pair.
- If there is no `'folw'` track reference to a subtitle track, a player most commonly determines the default subtitle track by matching aspects of the audio and subtitle tracks, typically by matching the extended language tag (or language code if there is no language tag) in the set of candidate tracks.

  If no match is found by language, a player might choose another candidate track based upon a user preference (for example, from a list of preferred languages). Another player might choose the first track in 'trak' box order in the 'moov' among the candidate tracks. Another player might have another mechanism to choose the default. Possible fall-back approaches are neither enumerated nor restricted here. Note that selecting no subtitle track may be appropriate.
- If the default matching between audio language tagging and subtitle track language tagging cannot be used, a 'folw' track reference must be authored in the media file. (For example, Norwegian as spoken uses a different language tag than the two language tags for Norwegian as written.)

2012-08-14 | Copyright © 2004, 2012 Apple Inc. All Rights Reserved.

Creating an Effect Description

An effect description tells QuickTime which effect to execute and contains the parameters that control how the effect behaves at runtime. You create an effect description by creating an atom container, inserting a QT atom that specifies the effect, and inserting a set of QT atoms that set its parameters.

There are support functions you can call to assist you in this process. `QTCreateStandardParameterDialog` returns a complete effect description that you can use, including user-selected settings; you only need to add `kEffectSourceName` atoms to the description for effects that require sources. At a lower level, `QTGetEffectsList` returns a list of the available effects and `ImageCodecGetParameterList` will return a description of the parameters for an effect, including the default value for each parameter in the form of a QT atom that can be inserted directly into an effect description.

Structure of an Effect Description

An effect description is the sole media sample for an effect track. An effect description is implemented as a `QTAtomContainer` structure, the general QuickTime structure for holding a set of QuickTime atoms. All effect descriptions must contain the set of required atoms, which specify attributes such as which effect component to use. In addition, effect descriptions can contain a variable number of parameter atoms, which hold the values of the parameters for the effect.

Each atom contains either data or a set of child atoms. If a parameter atom contains data, the data is the value of the parameter, and this value remains constant while the effect executes. If a parameter atom contains a set of child atoms, they typically contain a tween entry so the value of the parameter will be interpolated for the duration of the effect.

You assemble an effect description by adding the appropriate set of atoms to a `QTAtomContainer` structure.

You can find out what the appropriate atoms are by making an `ImageCodecGetParameterList` call to the effect component. This fills an atom container with a set of parameter description atoms. These atoms contain descriptions of the effect parameters, such as each parameter's atom type, data range, default value, and so on. The default value in each description atom is itself a `QTAtom` that can be inserted directly into your effect description.

You can modify the data in the parameter atoms directly, or let the user set them by calling `QTCreateStandardParameterDialog`, which returns a complete effect description (you need to add `kEffectSourceName` atoms for effects that require sources).

You then add the effect description to the media of the effect track.

Copyright © 2004, 2012 Apple Inc. All Rights Reserved.

Required Atoms of an Effects Description

There are several required atoms that an effect description must contain. The first is the `kParameterWhatName` atom. The `kParameterWhatName` atom contains the name of the effect. This specifies which of the available effects to use.

The code snippet shown in Listing 6-10 (page 338) adds a `kParameterWhatName` atom to the atom container `effectDescription`. The constant `kCrossFadeTransitionType` contains the name of the cross-fade effect.

Listing 6-10 Adding a `kParameterWhatName` atom to the atom container `effectDescription`

```
effectCode = kCrossFadeTransitionType;
QTInsertChild(effectDescription, kParentAtomIsContainer,
                kParameterWhatName, kParameterWhatID, 0,
                sizeof(effectCode), &effectCode, nil);
```

In addition to the `kParameterWhatName` atom, the effect description for an effect that uses sources must contain one or more `kEffectSourceName` atoms. Each of these atoms contains the name of one of the effect's sources. An input map is used to map these names to the actual tracks of the movie that are the sources. "Creating an Input Map" (page 340) describes how to create the input map.

Parameter Atoms of an Effects Description

In addition to the required atoms, the effects description contains a variable number of parameter atoms. The number and types of parameter atoms vary from effect to effect. For example, the cross fade effect has only one parameter, while the general convolution filter effect has nine. Some effects have no parameters at all, and do not require any parameter atoms.

You can obtain the list of parameter atoms for a given effect by calling the effect component using `ImageCodecGetParameterList`. The parameter description atoms it returns include default settings for each parameter in the form of parameter atoms that you can insert into your effect description.

The `QTInsertChild` function is used to add these parameters to the effect description, as seen in the code example in Listing 6-10 (page 338).

Consider, for instance, the push effect. Its effect description contains a `kParameterWhatName` atom, two `kEffectSourceName` atoms, and two parameter atoms, one of which is a tween.

The `kParameterWhatName` atom specifies that this is a `'push'` effect.

Copyright © 2004, 2012 Apple Inc. All Rights Reserved.

The two `kEffectSourceName` atoms specify the two sources that this effect will use, in this case `'srcA'` and `'srcB'`. The names correspond to entries in the effect track's input map.

The `'pcnt'` parameter atom defines which frames of the effect are shown. This parameter contains a tween entry, so that the value of this parameter is interpolated as the effect runs . The interpolation of the `'pcnt'` parameter causes consecutive frames of the effect to be rendered, creating the push effect.

The `'from'` parameter determines the direction of the push. This parameter is set from an enumeration list, with 2 being defined as the bottom of the screen.

In this example, the source `'srcB'` will push in from the bottom, covering the source `'srcA'`.

The `'pcnt'` parameter is normally tweened from 0 to 100, so that the effect renders completely, from 0 to 100 percent. In this example, the `'pcnt'` parameter is tweened from 25 to 75, so the effect will start 25 percent of the way through (with `'srcB'` already partly on screen) and finish 75 percent of the way through (with part of `'srcA'` still visible).

2012-08-14 | Copyright © 2004, 2012 Apple Inc. All Rights Reserved.

Figure 6-5 (page 340) shows the set of atoms that must be added to the entry description.

Figure 6-5 An example effect description for the Push effect

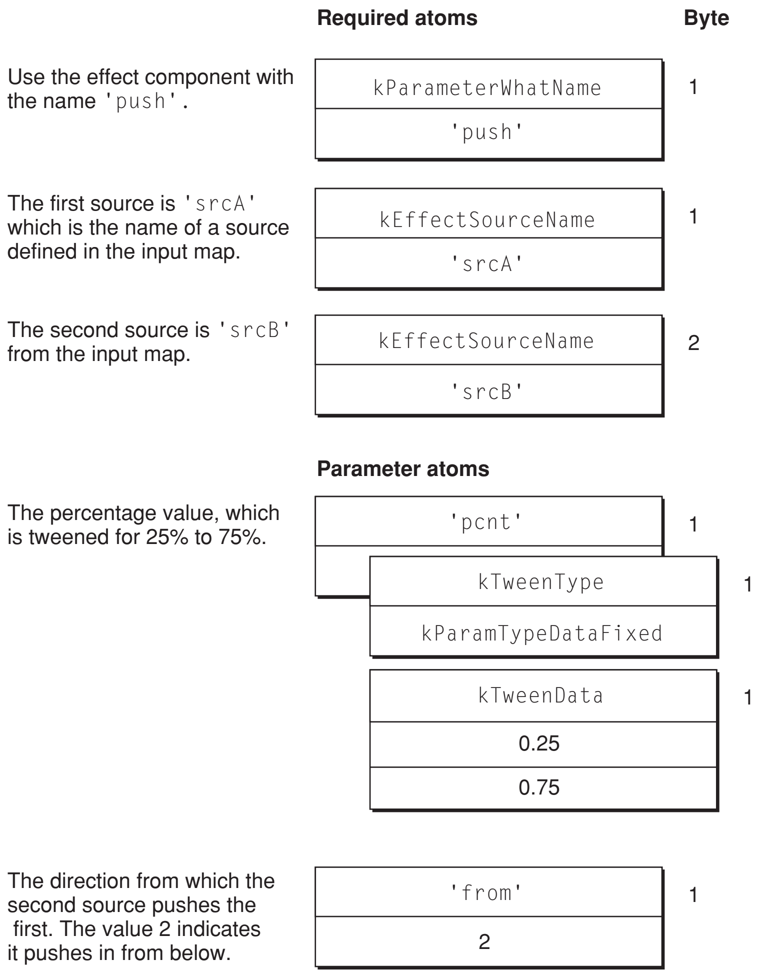

An important property of effect parameters is that most can be tweened (and some must be tweened). Tweening is QuickTime's general purpose interpolation mechanism (see "Tween Media" (page 235) for more information). For many parameters, it is desirable to allow the value of the parameter to change as the effect executes. In the example shown in Figure 6-5 (page 340), the `'pcnt'` parameter must be a tween. This parameter controls which frame of the effect is rendered at any given time, so it must change for the effect to progress. The `'from'` parameter is not a tween in the example above, but it could be if we wanted the direction of the push to change during the course of the effect.

Creating an Input Map

The input map is another QT atom container that you attach to the effects track. It describes the sources used in the effect and gives a name to each source. This name is used to refer to the source in the effects description.

2012-08-14 | Copyright © 2004, 2012 Apple Inc. All Rights Reserved.

An input map works in concert with track reference atoms in the source tracks. A track reference atom of type `kTrackModifierReference` is added to each source track, which causes that source track's output to be redirected to the effects track. An input map is added to the effects track to identify the source tracks and give a name to each source, such as `'srcA'` and `'srcB'`. The effect can then refer to the sources by name, specifying that `'srcB'` should slide in over `'srcA'`, for example.

Structure of an Input Map

The input map contains a set of atoms that refer to the tracks used as sources for the effect. Each source track is represented by one track reference atom of type `kTrackModifierInput`.

Each modifier input atom contains two children, one of type `kEffectDataSourceType`, and one of type `kTrackModifierType`, which hold the name and type of the source.

The name of the source is a unique identifier that you create, which is used in the effect description to reference the track. Any four-character name is valid, as long as it is unique in the set of source names.

> **Important:** Apple recommends you adopt the standard naming convention `'srcX'`, where X is a letter of the alphabet. Thus, your first source would be named `'srcA'`, the second `'srcB'`, and so forth. This convention is used here in this chapter.

The child atom of type `kTrackModifierType` indicates the type of the track being referenced. For a video track the type is `VideoMediaType`, for a sprite track it is `SpriteMediaType`, and so forth. Video tracks are the most common track type used as sources for effects. Only tracks that have a visible output, such as video and sprite tracks, can be used as sources for an effect. This means, for example, that sound tracks cannot be sources for an effect.

Figure 6-6 (page 342) shows a completed input map that references two sources. The first source is a video track and is called `'srcA'`. The second source, also a video track, is called `'srcB'`.

Copyright © 2004, 2012 Apple Inc. All Rights Reserved.

You refer to a `kTrackModifierInput` atom by its index number, which is returned by the `AddTrackReference` function when you create the atom.

Figure 6-6 An example of an input map referencing two sources

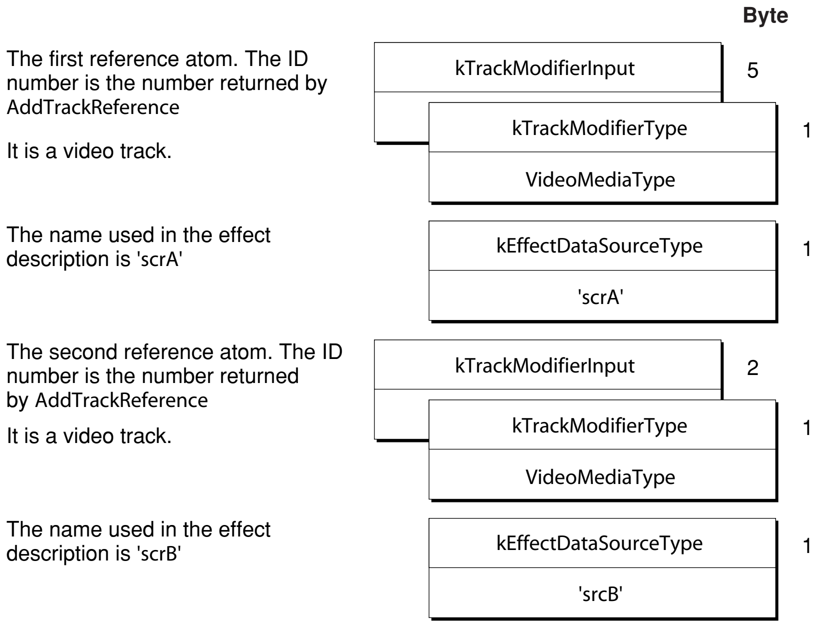

Building Input Maps

The first step in creating an input map is to create a new `QTAtomContainer` to hold the map. You use the standard QuickTime container creation function.

```
QTNewAtomContainer(&inputMap);
```

For each source you are creating, you need to call the `AddTrackReference` function. The track IDs of the effects track and the source track are passed as parameters to `AddTrackReference`, which creates an atom of type `kTrackModifierReference` and returns an index number. You use this index as the ID of the atom when you need to refer to it. You then insert the reference into the input map as an atom of type `kTrackModifierInput`.

The code in Listing 6-11 (page 342) creates a reference to the track `firstSourceTrack`, and adds it to the input map.

Listing 6-11 Adding an input reference atom to an input map

```
AddTrackReference(theEffectsTrack, firstSourceTrack,
```

2012-08-14 | Copyright © 2004, 2012 Apple Inc. All Rights Reserved.

```
                 kTrackModifierReference, &referenceIndex);

QTInsertChild(inputMap, kParentAtomIsContainer,
            kTrackModifierInput, referenceIndex, 0, 0, nil, &inputAtom);
```

The `QTInsertChild` function returns the offset of the new modifier input atom in the `inputAtom` parameter.

You now need to add the name and type of the source track to the modifier input atom. Again, calling the `QTInsertChild` function does this, as shown in the following code snippet:

```
inputType = VideoMediaType;
QTInsertChild(inputMap, inputAtom,
                kTrackModifierType, 1, 0, sizeof(inputType), &inputType,
                nil);

aType = 'srcA';
QTInsertChild(inputMap, inputAtom, kEffectDataSourceType, 1, 0,
            sizeof(aType), &aType, nil);
```

This process is repeated for each source for the effect.

Creating Movies with Modifier Tracks

QuickTime 2.1 added additional functionality for media handlers. By way of modifier tracks, a media handler can send its data to another media handler rather than presenting its media directly. See "Modifier Tracks" (page 247) for a complete discussion of this feature.

To create a movie with modifier tracks, first you create a movie with all the desired tracks, then you create the modifier track. To link the modifier track to the track that it modifies, you use the `AddTrackReference` function as shown in Listing 6-12 (page 343).

Listing 6-12 Linking a modifier track to the track it modifies

```
long addedIndex;
AddTrackReference(aVideoTrack, aModifierTrack,
                    kTrackModifierReference, &addedIndex);
```

Copyright © 2004, 2012 Apple Inc. All Rights Reserved.

The reference doesn't completely describe the modifier track's relationship to the track it modifies. Instead, the reference simply tells the modifier track to send its data to the specified track. The receiving track doesn't "know" what it should do with that data. A single track may also be receiving data from more than one modifier track.

To describe how each modifier input should be used, each track's media also has an input map. The media's input map describes how the data being sent to each input of a track should be interpreted by the receiving track. After creating the reference, it is necessary to update the receiving track's media input map. When `AddTrackReference` is called, it returns the index of the reference added. That index is the index of the input that needs to be described in the media input map. If the modifier track created above contains regions to change the shape of the video track, the code shown in Listing 6-13 (page 344) updates the input map appropriately.

Listing 6-13 Updating the input map

```
QTAtomContainer inputMap;
QTAtom inputAtom;
OSType inputType;



Media aVideoMedia = GetTrackMedia(aVideoTrack);
GetMediaInputMap (aVideoMedia, &inputMap);

QTInsertChild(inputMap, kParentAtomIsContainer, kTrackModifierInput,
        addedIndex, 0,0, nil, &inputAtom);

inputType = kTrackModifierTypeClip;
QTInsertChild (inputMap, inputAtom, kTrackModifierType, 1, 0,
        sizeof(inputType), &inputType, nil);

SetMediaInputMap(aVideoMedia, inputMap);
QTDisposeAtomContainer(inputMap);
```

The media input map allows you to store additional information for each input. In the preceding example, only the type of the input is specified. In other types of references, you may need to specify additional data.

Copyright © 2004, 2012 Apple Inc. All Rights Reserved.

When a modifier track is playing an empty track edit, or is disabled or deleted, all receiving tracks are notified that the track input is inactive. When an input becomes inactive, it is reset to its default value. For example, if a track is receiving data from a clip modifier track and that input becomes inactive, the shape of the track reverts to the shape it would have if there were no clip modifier track.

Authoring Movies with External Movie Targets

QuickTime 4 enables you to author movies with external movie targets. To specify an action that targets an element of an external movie, you must identify the external movie by either its name or its ID. Two new target atom types have been introduced for this purpose; these atoms are used in addition to the existing target atoms, which you may use to specify that the element is a particular track or object within a track, such as a sprite.

> **Note:** A movie ID may be specified by an expression.

These additional target atoms provided in QuickTime 4:

```
[(ActionTargetAtoms)] =
    <kActionTarget>

        <kTargetMovieName>
            [Pstring MovieName]
        OR
        <kTargetMovieID>
            [long MovieID]
            OR
            [(kExpressionAtoms)]
```

To tag a movie with a name or ID, you add a user data item of type 'plug' to the movie's user data. The index of the user data does not matter. The data specifies the name or ID.

You add a user data item of type 'plug' to the movie's user data with its data set to

```
"Movieid=MovieName"
```

where `MovieName` is the name of the movie.

2012-08-14 | Copyright © 2004, 2012 Apple Inc. All Rights Reserved.

You add a user data item of type `'plug'` to the movie's user data with its data set to

```
"Movieid=MovieID"
```

where the ID is a signed long integer.

The QuickTime plug-in additionally supports `EMBED` tag parameters, which allow you to override a movie's name or ID within an HTML page.

Target Atoms for Embedded Movies

QuickTime 4.1 introduced target atoms to accommodate the addition of embedded movies. These target atoms allow for paths to be specified in a hierarchical movie tree.

Target movies may be an external movie, the default movie, or any movie embedded within another movie. Targets are specified by using a movie path that may include parent and child movie relationships, and may additionally include track and track object target atoms as needed.

By using embedded `kActionTarget` atoms along with parent and child movie target atoms, you can build up paths for movie targets. Note that QuickTime looks for these embedded `kActionTarget` atoms only when evaluating a movie target, and any movie target type may contain a sibling `kActionTarget` atom.

Paths begin from the current movie, which is the movie containing the object that is handling an event. You may go up the tree using a `kTargetParentMovie` atom, or down the tree using one of five new child movie atoms. You may use a `kTargetRootMovie` atom as a shortcut to get to the top of the tree containing an embedded movie and may use the `movieByName` and `movieByID` atoms to specify a root external movie.

The target atoms are:

- `kTargetRootMovie` (leaf atom, no data). This is the root movie containing the action handler.
- `kTargetParentMovie` (leaf atom, no data). This is the parent movie.

Note that there are five ways to specify an embedded child movie. Three of them specify movie track properties. Two specify properties of the currently loaded movie in a movie track.

- `kTargetChildMovieTrackName.` A child movie track specified by track name.
- `kTargetChildMovieTrackID`. A child movie track specified by track ID.
- `kTargetChildMovieTrackIndex`. A child movie track specified by track index.
- `kTargetChildMovieMovieName`. A child movie specified by the currently loaded movie's movie name. The child movie must contain `movieName` user data with the specified name.

Copyright © 2004, 2012 Apple Inc. All Rights Reserved.

- `kTargetChildMovieMovieID`. A child movie specified by the currently loaded movie's movie ID. The child movie must contain `movieID` user data with the specified ID.

Adding Wired Actions To a Flash Track

This section explains the steps you need to follow in order to add wired actions to a Macromedia Flash track. The Flash media handler was introduced in QuickTime 4 to enable a SWF 3.0 file to be treated as a track within a QuickTime movie. See "Flash Media" (page 234) for more information about the Flash media handler.

Sample code (`AddFlashActions`) is provided on the QuickTime SDK, as well as on the QuickTime developer website, that lets you add wired actions to a Flash track.

Note: For more detailed information about working with Flash, you can download the Macromedia SWF File Format Specification at `http://www.macromedia.com/software/flash/open/spec/`, along with the SWF File Parser code also at the Macromedia website.

Extending the SWF Format

QuickTime 4 extends the SWF file format to allow the execution of any of its wired actions, in addition to the much smaller set of Flash actions. For example, you may use a SWF file as a user interface element in a QuickTime movie, controlling properties of the movie and other tracks. QuickTime also allows SWF files to be compressed using the zlib data compressor. This can significantly lower the bandwidth required when downloading a SWF file when it is in a QuickTime movie.

By using wired actions within a Flash track, compressing your Flash tracks, and combining Flash tracks with other types of QuickTime media, you can create compact and sophisticated multimedia content.

The SWF File Format Specification consists of a header followed by a series of tagged data blocks. The types of tagged data blocks you need to use are the `DefineButton2` and `DoAction`. The `DefineButton2` block allows Flash actions to be associated with a mouse state transition. `DoAction` allows actions to be executed when the tag is encountered. These are analogous to mouse-related QT event handlers and the frame loaded event in wired movies.

Flash actions are stored in an action record. Each Flash action has its own tag, such as `ActionPlay` and `ActionNextFrame`. QuickTime defines one new tag: `QuickTimeActions`, which is 0xAA. The data for the QuickTime actions tag is simply a QT atom container with the QuickTime wired actions to execute in it.

There are also fields you need to change in order to add wired actions to a SWF file. Additionally, there is one tag missing from the SWF file format that is described below.

2012-08-14 | Copyright © 2004, 2012 Apple Inc. All Rights Reserved.

What You Need to Modify

For `defineButton2`, you need to modify or add the following fields: file length, action records offset, the action offset, the condition, the record header size portion, and add action record.

File Length

A 32-bit field in the SWF file header.

`RecordHeader` for the `defineButton2`

`RecordHeader` contains the tag ID and length. You need to update the length. Note that there are short and long formats for record headers, depending on the size of the record. The tag ID for `defineButton2` is 34.

ActionRecordsOffset

The action records offset, a 16-bit field, is missing from the SWF File Format Specification. It occurs between the flags and buttons fields. It is initially set to 0 if there are no actions for the button. If there are actions for the button, then it must contain the offset from the point in the SWF file following this 16-bit value to the beginning of the action offset field.

```
DefineButton2 =


    Header
    ButtonID
    Flags


    ActionRecordsOffset     (this is missing from the spec)


    Buttons
    ButtonEndFlag
    Button2ActionCode
    ActionOffset
    Condition
    Action           [ActionRecords]
    ActionEndFlag
```

Copyright © 2004, 2012 Apple Inc. All Rights Reserved.

ActionOffset

There is one action offset per condition (mouse `overDownToIdle`). This is the offset used to skip over the condition and the following actions (the `ActionRecord`) for the condition. You need to update this value when adding actions.

Condition

The condition field is roughly equivalent to a wired movie event. The actions associated with button state transition condition are triggered when the transition occurs. You need to add or edit this field.

Actions

Flash actions each have their own action tag code. QuickTime actions use a single QuickTime actions code: `'AA'`. You may add a list of actions to a single QuickTime actions tag.

The format of the QuickTime actions tag is as follows:

```
1 byte:     // Tag = 'AA'
2 bytes:    // data length (size of the QTAtomContainer)
n bytes     // the data which is the QTAtomContainer holding  the
            // wired actions
```

DoAction

For `DoAction`, you need to modify a subset of the `defineButton2` fields in the same manner as described above. These fields are file length, the record header size portion, and the action record.

Note that you need to write the length fields in little-endian format.

Creating Video Tracks at 30 Frames per Second

The duration of a video frame is stored in the time-to-sample atom contained within a sample table atom. This duration cannot be interpreted without the media's time scale, which defines the units-per-second for the duration. In this example, each frame has the same duration, so the time-to-sample atom has one entry, which applies to all video frames in the media.

As long as the ratio between frame duration and media time scale remains 1:30, any combination of values can be used for the duration and time scale. The larger the time scale the shorter the maximum duration. Since a movie defaults to a time scale of 600, this is a good number to use. It is also the least common multiple for 24, 25, and 30, making it handy for much of the math you are likely to encounter when making a movie.

2012-08-14 | Copyright © 2004, 2012 Apple Inc. All Rights Reserved.

The movie time scale is independent of the media time scale. Since you want to avoid movie edits that don't land on frame boundaries, it is a good idea to keep the movie time scale and the media time scale the same, or the movie time scale should be an even multiple of the media time scale. The movie time scale is stored in the movie header atom.

With a time scale of 600 in the media header atom, the time-to-sample atom would contain the data values listed in Table 6-1 (page 350).

Table 6-1 Example data values for time scale of 600

Field	Value
Atom size	24
Atom type	`'stts'`
Version/Flags	0
Number of entries	1
Sample count	n
Sample duration	20

Creating Video Tracks at 29.97 Frames per Second

NTSC color video is not 30 frames per second (fps), but actually 29.97 fps. The previous example showed how the media time scale and the duration of the frames specify the video's frame rate. By setting the media's time scale to 2997 units per second and setting the frame durations to 100 units each, the effective rate is 29.97 fps exactly.

In this situation, it is also a good idea to set the movie time scale to 2997 in order to avoid movie edits that don't land on frame boundaries. The movie's time scale is stored in the movie header atom.

With a time scale of 2997 in the media header atom, the time-to-sample atom would contain the data values listed in Table 6-2 (page 350).

Table 6-2 Example data values for time scale of 2997

Field	Value
Atom size	24

Copyright © 2004, 2012 Apple Inc. All Rights Reserved.

Field	Value
Atom type	`'stts'`
Version/Flags	0
Number of entries	1
Sample count	n
Sample duration	100

Creating Sound Tracks at 44.1 kHz

The duration of an audio sample is stored in the time-to-sample atom contained in a sample table atom. This duration cannot be interpreted without the media's time scale, which defines the units per second for the duration. With audio, the duration of each audio sample is typically 1, so the time-to-sample atom has one entry, which applies to all audio samples.

With a time scale of 44100 in the media header atom, the time-to-sample atom would contain the data values listed in Table 6-3 (page 351).

Table 6-3 Example data values for time scale of 44100

Field	Value
Atom size	24
Atom type	`'stts'`
Version/Flags	0
Number of entries	1
Sample count	n
Sample duration	1

This atom does not indicate whether the audio is stereo or mono or whether it contains 8-bit or 16-bit samples. That information is stored in the sound sample description atom, which is contained in the sample table atom.

2012-08-14 | Copyright © 2004, 2012 Apple Inc. All Rights Reserved.

Creating a Timecode Track for 29.97 FPS Video

A timecode track specifies timecode information for other tracks. The timecode keeps track of the time codes of the original source of the video and audio. After a movie has been edited, the timecode can be extracted to determine the source tape and the time codes of the frames.

It is important that the timecode track have the same time scale as the video track. Otherwise, the timecode will not tick at the exact same time as the video track.

For each contiguous source tape segment, there is a single timecode sample that specifies the timecode value corresponding to the start of the segment. From this sample, the timecode value can be determined for any point in the segment.

The sample description for a timecode track specifies the timecode system being used (for example, a 30-fps drop frame) and the source information. Each sample is a timecode value.

Since the timecode media handler is a derived from the base media handler, the media information atom starts with a generic media header atom. The timecode atoms would contain the following data values:

Atom size	77		
Atom type	`'gmhd'`		
	Atom size	69	
	Atom type	`'gmin'`	
	Version/Flags	0	
	Graphics mode	0x0040	
	Opcolor (red)	0x8000	
	Opcolor (green)	0x8000	
	Opcolor (blue)	0x8000	
	Balance	0	
	Reserved	0	
	Atom size	45	
	Atom type	`'tmcd'`	
		Atom size	37

Copyright © 2004, 2012 Apple Inc. All Rights Reserved.

		Atom type	'tcmi'
		Version/Flags	0
		Text font	0 (system font)
		Text face	0 (plain)
		Text size	12
		Text color (red)	0
		Text color (green)	0
		Text color (blue)	0
		Background color (red)	0
		Background color (green)	0
		Background color (blue)	0
		Font name	'\pChicago' (Pascal string)

The sample table atom contains all the standard sample atoms and has the following data values:

Atom size	174		
Atom type	'stbl' (sample table)		
	Atom size	74	
	Atom type	'stsd' (sample description)	
	Version/Flags	0	
	Number of entries	1	
	Sample description size [1]	58	
	Data format [1]	'tmcd'	
	Reserved [1]	0	
	Data reference index [1]	1	

2012-08-14 | Copyright © 2004, 2012 Apple Inc. All Rights Reserved.

	Flags[1]	0	
	Flags (timecode) [1]	7 (drop frame + 24 hour + negative times OK)	
	Time scale[1]	2997	
	Frame duration[1]	100	
	Number of frames [1]	20	
		Atom size	24
		Atom type	`'name'`
		String length	12
		Language code	0 (English)
		Name	“my tape name”
	Atom size	24	
	Atom type	`'stts'` (time to sample)	
	Version/Flags	0	
	Number of entries	1	
	Sample count[1]	1	
	Sample duration[1]	1	
	Atom size	28	
	Atom type	`'stsc'` (sample to chunk)	
	Version/Flags	0	
	Number of entries	1	
	First chunk[1]	1	
	Samples per chunk[1]	1	
	Sample description ID[1]	1	

2012-08-14 | Copyright © 2004, 2012 Apple Inc. All Rights Reserved.

	Atom size	20	
	Atom type	'stsz' (sample size)	
	Version/Flags	0	
	Sample size	4	
	Number of entries	1	
	Atom size	20	
	Atom type	'stco' (chunk offset)	
	Version/Flags	0	
	Number of entries	1	
	Offset [1]	(offset into file of chunk 1)	

In the example, let's assume that the segment's beginning timecode is 1:15:32.4 (1 hour, 15 minutes, 32 seconds, and 4 frames). The time would be expressed in the data file as 0x010F2004 (0x01 = 1 hour; 0x0F = 15 minutes; 0x20 = 32 seconds; 0x04 = 4 frames).

The video and sound tracks must contain a track reference atom to indicate that they reference this timecode track. The track reference is the same for both and is contained in the track atom (at the same level as the track header and media atoms).

This track reference would contain the data values listed in Table 6-4 (page 355).

Table 6-4 Example track reference data values

Field	Value
Atom size	12
Atom type	'tref'
Reference type	'tmcd'
Track ID of referenced track (timecode track)	3

In this example, the video and sound tracks are tracks 1 and 2. The timecode track is track 3.

Copyright © 2004, 2012 Apple Inc. All Rights Reserved.

Playing with Edit Lists

A segment of a movie can be repeated without duplicating media data by using edit lists. Suppose you have a single-track movie whose media time scale is 100 and track duration is 1000 (10 seconds). For this example the movie's time scale is 600. If there are no edits in the movie, the edit atom would contain the data values listed in Table 6-5 (page 356).

Table 6-5 Example edit atom data values

Atom size	36	
Atom type	'edts'	
	Atom size	28
	Atom type	'elst'
	Version/Flags	0
	Number of entries	2
	Track duration	6000 (10 seconds)
	Media time	0
	Media rate	1.0

Because this is a single-track move, the track's duration in the track header atom is 6000 and the movie's duration in the movie header atom is 6000.

If you change the track to play the media from time 0 to time 2 seconds, and then play the media from time 0 to time 10 seconds, the edit atom would now contain these data values:

Atom size	48	
Atom type	'edts'	
	Atom size	40
	Atom type	'elst'
	Version/Flags	0
	Number of entries	2
	Track duration[1]	1200 (2 seconds)

Copyright © 2004, 2012 Apple Inc. All Rights Reserved.

	Media time[1]	0
	Media rate[1]	1.0
	Track duration[2]	6000 (10 seconds)
	Media time[2]	0
	Media rate[2]	1.0

Because the track is now 2 seconds longer, the track's duration in the track header atom must now be 7200, and the movie's duration in the movie header atom must also be 7200.

Currently, the media plays from time 0 to time 2, then plays from time 0 to time 10. If you take that repeated segment at the beginning (time 0 to time 2) and play it at double speed to maintain the original duration, the edit atom would now contain the following values:

Atom size	60	
Atom type	`'edts'`	
	Atom size	52
	Atom type	`'elst'`
	Version/Flags	0
	Number of entries	3
	Track duration[1]	600 (1 second)
	Media time[1]	0
	Media rate[1]	2.0
	Track duration[2]	600 (1 second)
	Media time[2]	0
	Media rate[2]	2.0
	Track duration[3]	4800 (8 seconds)
	Media time[3]	200
	Media rate[3]	1.0

2012-08-14 | Copyright © 2004, 2012 Apple Inc. All Rights Reserved.

Because the track is now back to its original duration of 10 seconds, its duration in the track header atom is 6000, and the movie's duration in the movie header atom is 6000.

Interleaving Movie Data

In order to get optimal movie playback, you must create the movie with interleaved data. Because the data for the movie is placed on disk in time order, the data for a particular time in the movie is close together in the file. This means that you must intersperse the data from different tracks. To illustrate this, consider a movie with a single video and a single sound track.

Figure 6-7 (page 358) shows how the movie data was collected, and how the data would need to be played back for proper synchronization. In this example, the video data is recorded at 10 frames per second and the audio data is grouped into half-second chunks.

Figure 6-7 Non-interleaved movie data

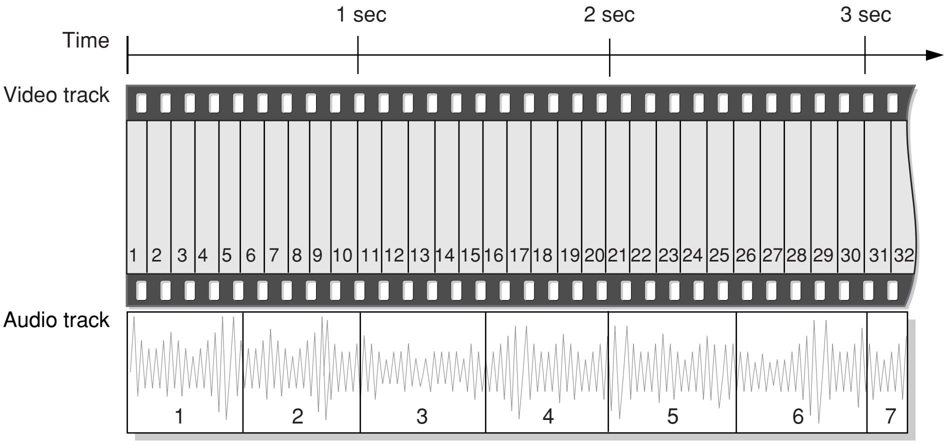

After the data has been interleaved on the disk, the movie data atom would contain movie data in the order shown in Figure 6-8 (page 358).

Figure 6-8 Interleaved movie data

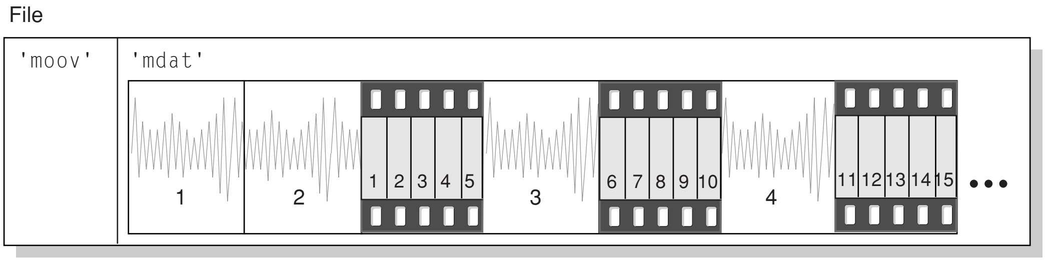

2012-08-14 | Copyright © 2004, 2012 Apple Inc. All Rights Reserved.

In this example, the file begins with the movie atom (`'moov'`), followed by the movie data atom (`'mdat'`). In order to overcome any latencies in sound playback, at least one second of sound data is placed at the beginning of the interleaved data. This means that the sound and video data are offset from each other in the file by one second.

Referencing Two Data Files With a Single Track

The data reference index to be used for a given media sample is stored within that sample's sample description. Therefore, a track must contain multiple sample descriptions in order for that track to reference multiple data files. A different sample description must be used whenever the data file changes or whenever the format of the data changes. The sample-to-chunk atom determines which sample description to use for a sample.

The sample description atom would contain the following data values:

Atom size	…	
Atom type	`'stsd'`	
Version/Flags	0	
Number of entries	2	
	Sample description size[1]	…
	Data format	`'tmcd'`
	Reserved	0
	Data reference index	1
	(sample data)	…
	Sample description size[1]	…
	Data format	`'tmcd'`
	Reserved	0
	Data reference index	2
	(sample data)	…

2012-08-14 | Copyright © 2004, 2012 Apple Inc. All Rights Reserved.

If there is only 1 sample per chunk and the first 10 samples are extracted from sample description 2 and the next 30 samples are extracted from sample description 1, the sample-to-chunk atom would contain the following data values:

Atom size	40
Atom type	`'stsc'`
Version/Flags	0
Number of entries	2
First chunk[1]	1
Samples per chunk[1]	1
Sample description ID[1]	2
First chunk[2]	11
Samples per chunk[2]	1
Sample description ID[2]	1

The data reference atom would contain the following data values:

Atom size	…	
Atom type	`'dinf'`	
	Atom size	…
	Atom type	`'dref'`
	Version/Flags	0
	Number of entries	2
	Size[1]	…
	Type[1]	`'alis'`
	Version[1]	0
	Flags[1]	0 (not self referenced)
	Data reference[1]	[alias pointing to file #1]

2012-08-14 | Copyright © 2004, 2012 Apple Inc. All Rights Reserved.

	Size[2]	...
	Type[2]	'rsrc'
	Version[2]	0
	Flags[2]	0 (not self referenced)
	Data reference[2]	[alias pointing to file #2]

Getting the Name of a QuickTime VR Node

You can use standard QuickTime atom container functions to retrieve the information in a QuickTime VR node header atom. For example, the `MyGetNodeName` function defined in Listing 6-14 (page 361) returns the name of a node, given its node ID.

Listing 6-14 Getting a node's name

```
OSErr MyGetNodeName (QTVRInstance theInstance, UInt32 theNodeID,
                                                                StringPtr
theStringPtr)
{
    OSErr                   theErr = noErr;
    QTAtomContainer         theNodeInfo;
    QTVRNodeHeaderAtomPtr   theNodeHeader;
    QTAtom                  theNodeHeaderAtom = 0;

    //Get the node information atom container.
    theErr = QTVRGetNodeInfo(theInstance, theNodeID, &theNodeInfo);

    //Get the node header atom.
    if (!theErr)
        theNodeHeaderAtom = QTFindChildByID(theNodeInfo, kParentAtomIsContainer,
                                                   kQTVRNodeHeaderAtomType,  1,
 nil);
    if (theNodeHeaderAtom != 0) {
        QTLockContainer(theNodeInfo);
```

Copyright © 2004, 2012 Apple Inc. All Rights Reserved.

```
        //Get a pointer to the node header atom data.
        theErr = QTGetAtomDataPtr(theNodeInfo, theNodeHeaderAtom,  nil,
                                                               (Ptr  *)&theNodeHeader);
        //See if there is a name atom.
        if (!theErr && theNodeHeader->nameAtomID != 0)  {
            QTAtom theNameAtom;
            theNameAtom = QTFindChildByID(theNodeInfo, kParentAtomIsContainer,
                                 kQTVRStringAtomType, theNodeHeader->nameAtomID,
nil);
            if (theNameAtom != 0) {
                VRStringAtomPtr theStringAtomPtr;

                //Get a pointer to the name atom data; copy it into  the string.
                theErr = QTGetAtomDataPtr(theNodeInfo, theNameAtom,  nil,
                                                                  (Ptr
*)&theStringAtomPtr);
                if (!theErr) {
                    short theLen = theStringAtomPtr->stringLength;
                    if (theLen > 255)
                        theLen = 255;
                  BlockMove(theStringAtomPtr->string, &theStringPtr[1],  theLen);
                    theStringPtr[0] = theLen;
                }
            }
        }
        QTUnlockContainer(theNodeInfo);
    }

    QTDisposeAtomContainer(theNodeInfo);
    return(theErr);
}
```

The `MyGetNodeName` function defined in Listing 6-14 (page 361) retrieves the node information atom container (by calling `QTVRGetNodeInfo`) and then looks inside that container for the node header atom with atom ID 1. If it finds one, it locks the container and then gets a pointer to the node header atom data. The desired information, the node name, is contained in the string atom whose atom ID is specified by the `nameAtomID`

Copyright © 2004, 2012 Apple Inc. All Rights Reserved.

field of the node header structure. Accordingly, the `MyGetNodeName` function then calls `QTFindChildByID` once again to find that string atom. If the string atom is found, `MyGetNodeName` calls `QTGetAtomDataPtr` to get a pointer to the string atom data. Finally, `MyGetNodeName` copies the string data into the appropriate location and cleans up after itself before returning.

Adding Custom Atoms in a QuickTime VR Movie

If you author a QuickTime VR movie, you may choose to add custom atoms to either the VR world or node information atom containers. Those atoms can be extracted within an application to provide additional information that the application may use.

Information that pertains to the entire scene might be stored in a custom atom within the VR world atom container. Node-specific information could be stored in the individual node information atom containers or as sibling atoms to the node location atoms within the VR world.

Custom hot spot atoms should be stored as siblings to the hot spot information atoms in the node information atom container. Generally, its atom type is the same as the custom hot spot type. You can set up an intercept procedure in your application in order to process clicks on the custom hot spots.

If you use custom atoms, you should install your hot spot intercept procedure when you open the movie. Listing 6-15 (page 363) is an example of such an intercept procedure.

Listing 6-15 Typical hot spot intercept procedure

```
QTVRInterceptProc MyProc = NewQTVRInterceptProc (MyHotSpot);
QTVRInstallInterceptProc (qtvr, kQTVRTriggerHotSpotSelector, myProc,  0, 0);

pascal void MyHotSpot (QTVRInstance qtvr, QTVRInterceptPtr qtvrMsg,
                        SInt32 refCon, Boolean *cancel)
{
    UInt32 hotSpotID = (UInt32) qtvrMsg->parameter[0];
    QTAtomContainer nodeInfo =
            (QTAtomContainer) qtvrMsg->parameter[1];
    QTAtom hotSpotAtom = (QTAtom) qtvrMsg->parameter[2];
    OSType hotSpotType;
    CustomData myCustomData;
    QTAtom myAtom;
```

2012-08-14 | Copyright © 2004, 2012 Apple Inc. All Rights Reserved.

```
    QTVRGetHotSpotType (qtvr, hotSpotID, &hotSpotType);
    if (hotSpotType != kMyAtomType) return;

    // It's our type of hot spot - don't let anyone else handle  it
    *cancel = true;

    // Find our custom atom
    myAtom = QTFindChildByID (nodeInfo, hotSpotAtom, kMyAtomType,  1, nil);
    if (myAtom != 0) {
        OSErr err;
        // Copy the custom data into our structure
        err = QTCopyAtomDataToPtr (nodeInfo, myAtom, false,
                        sizeof(CustomData), &myCustomData, nil);
        if (err == noErr)
            // Do something with it
            DoMyHotSpotStuff (hotSpotID, &myCustomData);
    }
}
```

Your intercept procedure is called for clicks on any hot spot. You should check to see if it is your type of hot spot and, if so, extract the custom hot spot atom and do whatever is appropriate for your hot spot type (`DoMyHotSpotStuff`).

When you no longer need the intercept procedure you should call `QTVRInstallInterceptProc` again with the same selector and a `nil` procedure pointer and then call `DisposeRoutineDescriptor` on `myProc`.

Apple reserves all hot spot and atom types with lowercase letters. Your custom hot spot type should contain all uppercase letters.

Adding Atom Containers in a QuickTime VR Movie

Assuming you have already created the QuickTime VR world and node information atom containers, you would use the code (minus error checking) Listing 6-16 (page 365) to add them to the QTVR track.

Listing 6-16 Adding atom containers to a track

```
long descSize;
QTVRSampleDescriptionHandle qtvrSampleDesc;



// Create a  QTVR sample description handle


descSize = sizeof(QTVRSampleDescription) + GetHandleSize((Handle)  vrWorld) -
                                        sizeof(UInt32);
qtvrSampleDesc = (QTVRSampleDescriptionHandle) NewHandleClear (descSize);
(*qtvrSampleDesc)->size = descSize;
(*qtvrSampleDesc)->type = kQTVRQTVRType;

// Copy the VR world atom container data into the QTVR sample description
BlockMove (*((Handle) vrWorld), &((*qtvrSampleDesc)->data),
                            GetHandleSize((Handle) vrWorld));
// Now add it to the QTVR track's media
err = BeginMediaEdits (qtvrMedia);
err = AddMediaSample (qtvrMedia, (Handle) nodeInfo, 0,
    GetHandleSize((Handle) nodeInfo), duration,
    (SampleDescriptionHandle) qtvrSampleDesc, 1, 0, &sampleTime);
err = EndMediaEdits (qtvrMedia);
InsertMediaIntoTrack (qtvrTrack, trackTime, sampleTime, duration,  1L<<16);
```

The `duration` value is computed based on the duration of the corresponding image track samples for the node. The value of `trackTime` is the time for the beginning of the current node (zero for a single node movie). The values of `duration` and `sampleTime` are in the time base of the media; the value of `trackTime` is in the movie's time base.

Optimizing QuickTime VR Movies for Web Playback

Originally, both QuickTime movies and QuickTime VR movies had to be completely downloaded to the user's local hard disk before they could be viewed. Starting with QuickTime 2.5, if the movie data is properly laid out in the file, standard linear QuickTime movies can be viewed almost immediately. The frames that have been downloaded so far are shown while subsequent frames continue to be downloaded.

2012-08-14 | Copyright © 2004, 2012 Apple Inc. All Rights Reserved.

The important change that took place to allow this to happen was for QuickTime to place global movie information at the beginning of the file. Originally, this information was at the end of the file. After that, the frame data simply needs to be in order in the file. Similarly, QuickTime VR files also need to be laid out in a certain manner in order to get some sort of quick feedback when viewing on the web. Roughly speaking this involves writing out all of the media samples in the file in a particular order. Apple now provides a movie export component that does this for you: the QTVR Flattener.

The QTVR Flattener

The QTVR Flattener is a movie export component that converts an existing QuickTime VR single node movie into a new movie that is optimized for the Web. Not only does the flattener reorder the media samples, but for panoramas it also creates a small preview of the panorama. When viewed on the Web, this preview appears after 5% to 10% of the movie data has been downloaded, allowing users to see a lower-resolution version of the panorama.

Using the QTVR flattener from your application is quite easy. After you have created the QuickTime VR movie, you simply open the QTVR Flattener component and call the `MovieExportToFile` routine as shown in Listing 6-17 (page 366).

Listing 6-17 Using the flattener

```
ComponentDescription desc;
Component flattener;
ComponentInstance qtvrExport = nil;
desc.componentType = MovieExportType;
desc.componentSubType = MovieFileType;
desc.componentManufacturer = QTVRFlattenerType;
flattener = FindNextComponent(nil, &desc);
if (flattener) qtvrExport = OpenComponent (flattener);
if (qtvrExport)
    MovieExportToFile (qtvrExport, &myFileSpec, myQTVRMovie,  nil, 0, 0);
```

The code fragment shown in Listing 6-17 (page 366) creates a flattened movie file specified by the `myFileSpec` parameter. If your QuickTime VR movie is a panorama, the flattened movie file includes a quarter size, blurred JPEG, compressed preview of the panorama image.

2012-08-14 | Copyright © 2004, 2012 Apple Inc. All Rights Reserved.

Note: The constants `MovieExportType` and `MovieFileType` used in Listing 6-17 (page 366) are defined in header files `QuickTimeComponents.h` and `Movies.h` respectively and are defined as `'spit'` and `'MooV'`.

You can present users with the QTVR Flattener's own dialog box to allow them to choose options such as how to compress the preview image or to select a separate preview image file. Use the following code to show the dialog box:

```
        err = MovieExportDoUserDialog (qtvrExport, myQTVRMovie,  nil, 0, 0,
&cancel);
```

If the user cancels the dialog box, then the Boolean cancel is set to `true`.

If you do not want to present the user with the flattener's dialog box, you can communicate directly with the component by using the `MovieExportSetSettingsFromAtomContainer` routine as described in the following paragraphs.

If you want to specify a preview image other than the default, you need to create a special atom container and then call `MovieExportSetSettingsFromAtomContainer` before calling `MovieExportToFile`. You can specify how to compress the image, what resolution to use, and you can even specify your own preview image file to be used. The atom container you pass in can have various atoms that specify certain export options. These atoms must all be children of a flattener settings parent atom.

The preview resolution atom is a 16-bit value that allows you to specify the resolution of the preview image. This value, which defaults to `kQTVRQuarterRes`, indicates how much to reduce the preview image.

The blur preview atom is a Boolean value that indicates whether to blur the image before compressing. Blurring usually results in a much more highly compressed image. The default value is `true`.

The create preview atom is a Boolean value that indicates whether a preview image should be created. The default value is `true`.

The import preview atom is a Boolean value that is used to indicate that the preview image should be imported from an external file rather than generated from the image in the panorama file itself. This allows you to have any image you want as the preview for the panorama. You can specify which file to use by also including the import specification atom, which is an `FSSpec` data structure that identifies the image file. If you do not include this atom, then the flattener presents the user with a dialog box asking the user to select a file. The default for import preview is `false.` If an import file is used, the image is used at its natural size and the resolution setting is ignored.

2012-08-14 | Copyright © 2004, 2012 Apple Inc. All Rights Reserved.

Sample Atom Container for the QTVR Flattener

The sample code in Listing 6-18 (page 368) creates an atom container and adds atoms to indicate an import preview file for the flattener to use.

Listing 6-18 Specifying a preview file for the flattener to use

```
Boolean yes = true;
QTAtomContainer exportData;
QTAtom parent;
err = QTNewAtomContainer(&exportData);
// create a parent for the other settings atoms
err = QTInsertChild (exportData, kParentAtomIsContainer,
            QTVRFlattenerParentAtomType, 1, 0, 0, nil, &parent);
// Add child atom to indicate we want to import the preview from  a file
err = QTInsertChild (exportData, parent, QTVRImportPreviewAtomType,  1, 0,
            sizeof (yes), &yes, nil);
// Add child atom to tell which file to import
err = QTInsertChild (exportData, parent, QTVRImportSpecAtomType,  1, 0,
            sizeof (previewSpec), &previewSpec, nil);
// Tell the export component
MovieExportSetSettingsFromAtomContainer (qtvrExport, exportData);
```

Overriding the compression settings is a bit more complicated. You need to open a standard image compression dialog component and make calls to obtain an atom container that you can then pass to the QTVR Flattener component.

Listing 6-19 Overriding the compression settings

```
ComponentInstance sc;
QTAtomContainer compressorData;
SCSpatialSettings ss;
sc = OpenDefaultComponent(StandardCompressionType,StandardCompressionSubType);
ss.codecType = kCinepakCodecType;
ss.codec = nil;
ss.depth = 0;
ss.spatialQuality = codecHighQuality
```

Copyright © 2004, 2012 Apple Inc. All Rights Reserved.

```
err = SCSetInfo(sc, scSpatialSettingsType, &ss);
err = SCGetSettingsAsAtomContainer(sc, &compressorData);
MovieExportSetSettingsFromAtomContainer (qtvrExport, compressorData);
```

QuickTime Image File Format

Note: The QuickTime Image File Format is deprecated in the QuickTime file format. The information that follows is intended to document existing content containing QuickTime image file media and should not be used for new development.

This appendix describes QuickTime image files, which provide a useful container for QuickTime-compressed still images.

Most still image file formats define both how images should be stored and compressed. However, the QuickTime image file format is a container format, which describes a storage mechanism independent of compression. The QuickTime image file format uses the same atom-based structure as a QuickTime movie.

Atom Types in QuickTime Image Files

There are two mandatory atom types: `'idsc'`, which contains an image description, and `'idat'`, which contains the image data. This is illustrated in Figure A-1 (page 371). A QuickTime image file can also contain other atoms.

In QuickTime 4, there is a new optional atom type `'iicc'`, which can store a ColorSync profile.

Copyright © 2004, 2012 Apple Inc. All Rights Reserved.

Figure A-1 (page 371) and Table A-1 (page 371) show an example QuickTime image file containing a JPEG-compressed image.

Figure A-1 An 'idsc' atom followed by an 'idat' atom

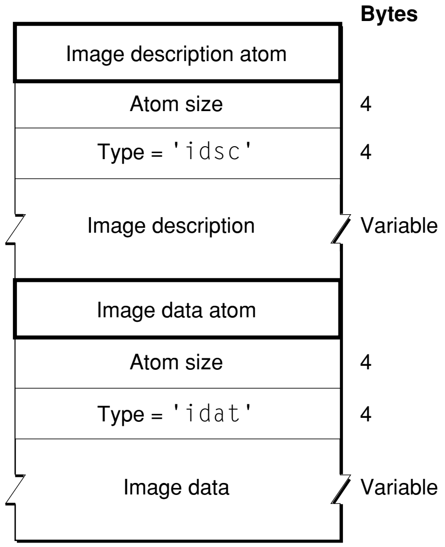

Table A-1 A QuickTime image file containing JPEG-compressed data

0000005E	Atom size, 94 bytes
69647363	Atom type, 'idsc'
00000056	Image description size, 86 bytes
6A706567	Compressor identifier, 'jpeg'
00000000	Reserved, set to 0
0000	Reserved, set to 0
0000	Reserved, set to 0
00000000	Major and minor version of this data, 0 if not applicable
6170706C	Vendor who compressed this data, 'appl'
00000000	Temporal quality, 0 (no temporal compression)
00000200	Spatial quality, codecNormalQuality
0140	Image width, 320
00F0	Image height, 240

2012-08-14 | Copyright © 2004, 2012 Apple Inc. All Rights Reserved.

00480000	Horizontal resolution, 72 dpi
00480000	Vertical resolution, 72 dpi
00003C57	Data size, 15447 bytes (use 0 if unknown)
0001	Frame count, 1
0C 50 68 6F 74 6F 20 2D20<br>4A 50 45 47 00 00 00<br>00 00 00 00 00 00 00 00<br>00 00 00 00 00 00 00 00	Compressor name, "Photo - JPEG" (32-byte Pascal string)
0018	Image bit depth, 24
FFFF	Color lookup table ID, -1 (none)
00003C5F	Atom size, 15455 bytes
69646174	Atom type, `'idat'`
FF D8 FF E0 00 10 4A 46<br>49 46 00 01 01 01 00 48<br>...	JPEG compressed data

Important: The exact order and size of atoms is not guaranteed to match the example in Figure A-1 (page 371). Applications reading QuickTime image files should always use the atom size to traverse the file and ignore atoms of unrecognized types.

Note: Like QuickTime movie files, QuickTime image files are big-endian. However, image data is typically stored in the same byte order as specified by the particular compression format.

Recommended File Type and Suffix

On Mac OS systems, QuickTime image files are identified by the file type `'qtif'`. Apple recommends using the filename extension `.QIF` to identify QuickTime image files.

2012-08-14 | Copyright © 2004, 2012 Apple Inc. All Rights Reserved.

Defining Media Data Layouts

The QuickTime file format provides a great deal of flexibility in how media data is physically arranged within a file. However, it also allows media layouts to be created that may be inefficient for playback on a given device. To complicate the matter, a media layout that is inefficient for one device may be, in fact, very efficient for another. The purpose of this appendix is to define some common uses of QuickTime files and describe the media layout in these circumstances.

Using QuickTime Files and Media Layouts

A QuickTime file can reference media data stored in a number of files, including the file itself. If a QuickTime file references only media data contained within itself, the file is said to be self-contained. A QuickTime file can also reference media data stored in files that are not QuickTime files. This is because the QuickTime file format references media within a URL by file offset, rather than by a data structuring mechanism of a particular file format. This allows a QuickTime file to refer to data stored in any container format.

Often, it is convenient to store a single media stream per file, for example, when encoding content. It is also useful for purposes of reusing content. (To reuse an elementary stream, it is not necessary to extract it from a larger, possibly multiplexed file.)

Because QuickTime can reference media stored in any file, it is not required that media be stored in the QuickTime file format. However, this is recommended. Putting the elementary streams in a QuickTime file has several advantages, particularly in enabling interchange of the content between different tools. Further, the QuickTime file format adds very little overhead to the media—as little as a few hundred bytes in many cases—so there is no great penalty in storage space.

One of the issues facing any device (a server or a local workstation) that is attempting to play back a QuickTime file in real time is the number of file seeks that must be performed.

It is possible to arrange the data in a QuickTime file to minimize, and potentially eliminate, any seeks during the course of normal playback. (Of course, random access and other kinds of interactivity require seeks.) Minimizing seeks is accomplished by interleaving the media data in the QuickTime file in such a way that the layout of the media in the file corresponds to the order in which the media data will be required. It is expected that most servers, for example, will stream QuickTime media using the facilities of the hint tracks.

2012-08-14 | Copyright © 2004, 2012 Apple Inc. All Rights Reserved.

Take a scenario where the QuickTime file contains a single hint track that references an audio and a visual media stream. In order to eliminate all seeks, the hint track media must be interleaved with the audio and visual stream data. Because the hint track sample must always be read before the audio and visual media that it references, the hint track samples must always immediately precede the samples they reference.

A simple illustration of the ordering of data (that is, time and file offset increasing from left to right) is as follows:

```
H0 A0 H1 V1 H2 V2 H3 A1 H4 A2 V3 H5 V4
```

When a single hint sample references multiple pieces of media data, those pieces of media data must occur in the order that they are referenced.

2012-08-14 | Copyright © 2004, 2012 Apple Inc. All Rights Reserved.

Random Access

This appendix describes how to seek with a QuickTime file using child atoms.

Seeking With a QuickTime File

Seeking with a QuickTime file is accomplished primarily by using the child atoms contained in the sample table atom. If an edit list is present, it must also be consulted. If you want to seek a given track to a time T, where T is in the time scale of the movie header atom, you could perform the following operations:

1. If the track contains an edit list, determine which edit contains the time T by iterating over the edits. The start time of the edit in the movie time scale must then be subtracted from the time T to generate T', the duration into the edit in the movie time scale. T' is next converted to the time scale of the track's media to generate T''. Finally, the time in the media scale to use is calculated by adding the media start time of the edit to T''.
2. The time-to-sample atom for a track indicates what times are associated with which sample for that track. Use this atom to find the first sample prior to the given time.
3. The sample that was located in step 1 may not be a random access point. Locating the nearest random access point requires consulting two atoms. The sync sample table indicates which samples are in fact random access points. Using this table, you can locate which is the first sync sample prior to the specified time. The absence of the sync sample table indicates that all samples are synchronization points, and makes this problem easy. The shadow sync atom gives the opportunity for a content author to provide samples that are not delivered in the normal course of delivery, but which can be inserted to provide additional random access points. This improves random access without impacting bit rate during normal delivery. This atom maps samples that are not random access points to alternate samples which are. You should also consult this table if present to find the first shadow sync sample prior to the sample in question. Having consulted the sync sample table and the shadow sync table, you probably wish to seek to whichever resultant sample is closest to, but prior to, the sample found in step 1.
4. At this point you know the sample that will be used for random access. Use the sample-to-chunk table to determine in which chunk this sample is located.
5. Knowing which chunk contained the sample in question, use the chunk offset atom to figure out where that chunk begins.

2012-08-14 | Copyright © 2004, 2012 Apple Inc. All Rights Reserved.

6. Starting from this offset, you can use the information contained in the sample-to-chunk atom and the sample size atom to figure out where within this chunk the sample in question is located. This is the desired information.

2012-08-14 | Copyright © 2004, 2012 Apple Inc. All Rights Reserved.

Metadata Handling

This appendix describes how metadata is handled when QuickTime imports other file formats. (For more information about metadata, refer to "Overview of QTFF" (page 20) and "Compressed Movie Resources" (page 117)).

These formats are grouped into the following categories and sections:

- "Digital Video File Formats" (page 377)
- "Digital Audio File Formats" (page 378)
- "Still Image File Formats" (page 379)
- "Animation and 3D File Formats " (page 381)

Each section includes a table with specific details on the following, where applicable:

- The format supported by QuickTime—for example, the movie import component or the graphics import component
- The Macintosh file type—for example, `'Mp3 '`
- File name extensions––for example, `.mp3`
- Specific details for metadata handling—for example, all Microsoft-defined "tombstone" data is transferred to the imported movie's user data. metadata fields that have QuickTime equivalents are mapped as follows.
- Software required—for example, QuickTime 3 or later

Digital Video File Formats

OpenDML and other AVI files	Description
Supported by	Movie import component
Macintosh file type	`'VfW '`
File name extensions	`.avi`

2012-08-14 | Copyright © 2004, 2012 Apple Inc. All Rights Reserved.

OpenDML and other AVI files	Description
metadata handling	All Microsoft-defined “tombstone” data is transferred to the imported movie’s user data. metadata fields that have QuickTime equivalents are mapped as follows: 'ICOP' maps to kUserDataTextCopyright, 'ISBJ' maps to kUserDataTextInformation, 'INAM' maps to kUserDataTextFullName, 'ICRD' maps to '©day', 'IMED' maps to '©fmt', 'ISRC' maps to '©src'.Where no QuickTime equivalent exists, the metadata item’s four-character code is modified by replacing the initial I with the symbol ©. All other characters remain unchanged.
Software required	QuickTime 3

Digital Audio File Formats

MPEG 1 layer 3	Description
Supported by	Movie import component
Macintosh file type	'Mp3 ', 'SwaT', 'MPEG', 'PLAY', 'MPG3', 'MP3 '
File name extensions	.mp3, .swa
Metadata handling	Metadata from ID3v1-style MP3 files is imported into the QuickTime movie.Title maps to kUserDataTextFullName, artist maps to '©ART', album maps to '©alb', year maps to '©day', comment maps to '©cmt', and track number maps to '©des'.
Software required	QuickTime 4

WAV	Description
Supported by	Movie import component
Macintosh file type	'WAVE', '.WAV'
File name extensions	.wav

Copyright © 2004, 2012 Apple Inc. All Rights Reserved.

WAV	Description
Metadata handling	All Microsoft-defined "tombstone" data is transferred to the imported movie's user data. metadata fields that have QuickTime equivalents are mapped as follows: `'ICOP'` maps to `kUserDataTextCopyright`, `'ISBJ'` maps to `kUserDataTextInformation`, `'INAM'` maps to `kUserDataTextFullName`, `'ICRD'` maps to `'©day'`, `'IMED'` maps to `'©fmt'`, `'ISRC'` maps to `'©src'`.Where no QuickTime equivalent exists, the metadata item's four-character code is modified by replacing the initial `I` with the symbol ©. All other characters remain unchanged.
Software required	QuickTime 2.5 or later

Still Image File Formats

FlashPix	Description
Supported by	Graphics import component
Macintosh file type	`'FPix'`
File name extensions	`.fpx`
Metadata handling	Information about copyright, authorship, caption text, content description notes, camera manufacturer name, camera model name are transferred to `kUserDataTextCopyright`, `kUserDataTextArtistField`, `kUserDataTextFullName`, `kParameterInfoWindowTitle`, `kParameterInfoManufacturer`, `kUserDataTextMakeField` user data items, respectively.
Formats supported	1.0
Software required	QuickTime 4

GIF	Description
Supported by	Graphics import component
Macintosh file type	`'GIFf'`, or `'GIF '`
File name extensions	`.gif`
Metadata handling	The GIF comment field is transferred to the `kUserDataDateText-Information` user data item.

Copyright © 2004, 2012 Apple Inc. All Rights Reserved.

GIF	Description
Software required	QuickTime 2.5 or later

JFIF/JPEG	Description
Supported by	Graphics import component
Macintosh file type	'JPEG'
File name extensions	.jpg
Metadata handling	The JFIF comment field is transferred to the imported Movie's user data in the kUserDataTextInformation field.
Software required	QuickTime 2.5 or later

Photoshop	Description
Supported by	Graphics import component
Macintosh file type	'8BPS'
File name extensions	.psd
Metadata handling	Photoshop files store their metadata based on the IPTC-NAA Information Interchange Model and Digital Newsphoto Parameter Record. This information is transferred into the importer Movie's user data. The entire ITPC-NAA record is placed into a user data item of type 'iptc'. In addition, those metadata items which are defined by QuickTime are mapped directly to QuickTime types as follows: 116 to kUserDataTextCopyright, 120 to kUserDataTextInformation, 105 to kUserDataTextFullName, 55 to '©day', 115 to '©src'.
Software required	QuickTime 2.5 or later. QuickTime 3 is required for metadata handling.

QuickTime Image File	Description
Supported by	Graphics import component
Macintosh file type	'qtif'
File name extensions	.qtif, .qif, .qti

Copyright © 2004, 2012 Apple Inc. All Rights Reserved.

QuickTime Image File	Description
Metadata handling	Metadata that is stored in `quickTimeImageFileMetaDataAtom` atom is copied directly to the Movie's user data.
Formats supported	All
Software required	QuickTime 2.5 or later

TIFF	Description
Supported by	Graphics Import Component
Macintosh file type	`'TIFF'`
File name extensions	`.tif`, `.tiff`
Metadata handling	Extracted from standard tags and from IPTC block
Software required	QuickTime 3 or later

Animation and 3D File Formats

Animated GIF	Description
Supported by	Movie import component
Macintosh file type	`'GIFf'`
File name extensions	`.gif`
Metadata handling	The GIF comment field is transferred to `kUserDataTextInformation` user data item.
Software required	QuickTime 3 or later

2012-08-14 | Copyright © 2004, 2012 Apple Inc. All Rights Reserved.

Summary of VR World and Node Atom Types

Note: VR Media is deprecated in the QuickTime file format. The information that follows is intended to document existing content containing VR media and should not be used for new development.

This appendix includes information that pertains to Chapter 3, "VR World Atom Container" (page 268) and "Node Information Atom Container" (page 275).

C Summary

Constants

VR World Atom Types

```
enum {
    kQTVRWorldHeaderAtomType    = FOUR_CHAR_CODE('vrsc'),
    kQTVRImagingParentAtomType  = FOUR_CHAR_CODE('imgp'),
    kQTVRPanoImagingAtomType    = FOUR_CHAR_CODE('impn'),
    kQTVRObjectImagingAtomType  = FOUR_CHAR_CODE('imob'),
    kQTVRNodeParentAtomType     = FOUR_CHAR_CODE('vrnp'),
    kQTVRNodeIDAtomType         = FOUR_CHAR_CODE('vrni'),
    kQTVRNodeLocationAtomType   = FOUR_CHAR_CODE('nloc')
};
```

Node Information Atom Types

```
enum {
    kQTVRNodeHeaderAtomType     = FOUR_CHAR_CODE('ndhd'),
    kQTVRHotSpotParentAtomType  = FOUR_CHAR_CODE('hspa'),
    kQTVRHotSpotAtomType        = FOUR_CHAR_CODE('hots'),
```

Copyright © 2004, 2012 Apple Inc. All Rights Reserved.

```
    kQTVRHotSpotInfoAtomType    = FOUR_CHAR_CODE('hsin'),
    kQTVRLinkInfoAtomType       = FOUR_CHAR_CODE('link')
};
```

Miscellaneous Atom Types

```
enum {
    kQTVRStringAtomType              = FOUR_CHAR_CODE('vrsg'),
    kQTVRPanoSampleDataAtomType      = FOUR_CHAR_CODE('pdat'),
    kQTVRObjectInfoAtomType          = FOUR_CHAR_CODE('obji'),
    kQTVRAltImageTrackRefAtomType    = FOUR_CHAR_CODE('imtr'),
    kQTVRAltHotSpotTrackRefAtomType = FOUR_CHAR_CODE('hstr'),
    kQTVRAngleRangeAtomType          = FOUR_CHAR_CODE('arng'),
    kQTVRTrackRefArrayAtomType       = FOUR_CHAR_CODE('tref'),
    kQTVRPanConstraintAtomType       = FOUR_CHAR_CODE('pcon'),
    kQTVRTiltConstraintAtomType      = FOUR_CHAR_CODE('tcon'),
    kQTVRFOVConstraintAtomType       = FOUR_CHAR_CODE('fcon'),
    kQTVRCubicViewAtomType           = FOUR_CHAR_CODE('cuvw'),
    kQTVRCubicFaceDataAtomType       = FOUR_CHAR_CODE('cufa')
};
```

Track Reference Types

```
enum {
    kQTVRImageTrackRefType      = FOUR_CHAR_CODE('imgt'),
    kQTVRHotSpotTrackRefType    = FOUR_CHAR_CODE('hott')
};
```

Imaging Property Valid Flags

```
enum {
    kQTVRValidCorrection                = 1 << 0,
    kQTVRValidQuality                   = 1 << 1,
    kQTVRValidDirectDraw                = 1 << 2,
    kQTVRValidFirstExtraProperty        = 1 << 3
```

Copyright © 2004, 2012 Apple Inc. All Rights Reserved.

```
};
```

Link Hot Spot Valid Bits

```
enum {
    kQTVRValidPan                           = 1 << 0,
    kQTVRValidTilt                          = 1 << 1,
    kQTVRValidFOV                           = 1 << 2,
    kQTVRValidViewCenter                    = 1 << 3
};
```

Animation Settings

```
enum QTVRAnimationSettings {
    kQTVRObjectAnimateViewFramesOn          = (1 << 0),
    kQTVRObjectPalindromeViewFramesOn       = (1 << 1),
    kQTVRObjectStartFirstViewFrameOn        = (1 << 2),
    kQTVRObjectAnimateViewsOn               = (1 << 3),
    kQTVRObjectPalindromeViewsOn            = (1 << 4),
    kQTVRObjectSyncViewToFrameRate          = (1 << 5),
    kQTVRObjectDontLoopViewFramesOn         = (1 << 6),
    kQTVRObjectPlayEveryViewFrameOn         = (1 << 7)
};
```

Control Settings

```
enum QTVRControlSettings {
    kQTVRObjectWrapPanOn                    = (1 << 0),
    kQTVRObjectWrapTiltOn                   = (1 << 1),
    kQTVRObjectCanZoomOn                    = (1 << 2),
    kQTVRObjectReverseHControlOn            = (1 << 3),
    kQTVRObjectReverseVControlOn            = (1 << 4),
    kQTVRObjectSwapHVControlOn              = (1 << 5),
    kQTVRObjectTranslationOn                = (1 << 6)
};
```

Copyright © 2004, 2012 Apple Inc. All Rights Reserved.

Controller Subtype and ID

```
enum {
    kQTControllerType                          = FOUR_CHAR_CODE('ctyp').
    kQTControllerID                            = 1
};
```

Object Controller Types

```
enum ObjectUITypes {
    kGrabberScrollerUI                         = 1,
    kOldJoyStickUI                             = 2,
    kJoystickUI                                = 3,
    kGrabberUI                                 = 4,
    kAbsoluteUI                                = 5
};
```

Node Location Flag

```
enum {
    kQTVRSameFile                              = 0
};
```

Panorama Sample Flag

```
enum {
    kQTVRPanoFlagHorizontal                    = 1 << 0,
    kQTVRPanoFlagAlwaysWrap                    = 1 << 2
};
```

Data Types

```
typedef float                                    Float32;
```

Copyright © 2004, 2012 Apple Inc. All Rights Reserved.

Sample Description Header Structure

```
typedef struct QTVRSampleDescription {
    UInt32                                      size;
    UInt32                                      type;
    UInt32                                      reserved1;
    UInt16                                      reserved2;
    UInt16                                      dataRefIndex;
    UInt32                                      data;
} QTVRSampleDescription, *QTVRSampleDescriptionPtr, **QTVRSampleDescriptionHandle;
```

String Atom Structure

```
typedef struct QTVRStringAtom {
    UInt16                                      stringUsage;
    UInt16                                      stringLength;
    unsigned char                               string[4];
} QTVRStringAtom, *QTVRStringAtomPtr;
```

VR World Header Atom Structure

```
typedef struct QTVRWorldHeaderAtom {
    UInt16                                      majorVersion;
    UInt16                                      minorVersion;
    QTAtomID                                    nameAtomID;
    UInt32                                      defaultNodeID;
    UInt32                                      vrWorldFlags;
    UInt32                                      reserved1;
    UInt32                                      reserved2;
} QTVRWorldHeaderAtom, *QTVRWorldHeaderAtomPtr;
```

Panorama-Imaging Atom Structure

```
typedef struct QTVRPanoImagingAtom {
    UInt16                                      majorVersion;
    UInt16                                      minorVersion;
```

2012-08-14 | Copyright © 2004, 2012 Apple Inc. All Rights Reserved.

```
    UInt32                                  imagingMode;
    UInt32                                  imagingValidFlags;
    UInt32                                  correction;
    UInt32                                  quality;
    UInt32                                  directDraw;
    UInt32                                  imagingProperties[6];
    UInt32                                  reserved1;
    UInt32                                  reserved2;
} QTVRPanoImagingAtom, *QTVRPanoImagingAtomPtr;
```

Node Location Atom Structure

```
typedef struct QTVRNodeLocationAtom {
    UInt16                                  majorVersion;
    UInt16                                  minorVersion;
    OSType                                  nodeType;
    UInt32                                  locationFlags;
    UInt32                                  locationData;
    UInt32                                  reserved1;
    UInt32                                  reserved2;
} QTVRNodeLocationAtom, *QTVRNodeLocationAtomPtr;
```

Node Header Atom Structure

```
typedef struct QTVRNodeHeaderAtom {
    UInt16                                  majorVersion;
    UInt16                                  minorVersion;
    OSType                                  nodeType;
    QTAtomID                                nodeID;
    QTAtomID                                nameAtomID;
    QTAtomID                                commentAtomID;
    UInt32                                  reserved1;
    UInt32                                  reserved2;
} QTVRNodeHeaderAtom, *QTVRNodeHeaderAtomPtr;
```

Copyright © 2004, 2012 Apple Inc. All Rights Reserved.

Hot Spot Information Atom Structure

```
typedef struct QTVRHotSpotInfoAtom {
    UInt16                              majorVersion;
    UInt16                              minorVersion;
    OSType                              hotSpotType;
    QTAtomID                            nameAtomID;
    QTAtomID                            commentAtomID;
    SInt32                              cursorID[3];
    Float32                             bestPan;
    Float32                             bestTilt;
    Float32                             bestFOV;
    FloatPoint                          bestViewCenter;
    Rect                                hotSpotRect;
    UInt32                              flags;
    UInt32                              reserved1;
    UInt32                              reserved2;
} QTVRHotSpotInfoAtom, *QTVRHotSpotInfoAtomPtr;
```

Link Hot Spot Atom Structure

```
typedef struct QTVRLinkHotSpotAtom {
    UInt16                              majorVersion;
    UInt16                              minorVersion;
    UInt32                              toNodeID;
    UInt32                              fromValidFlags;
    Float32                             fromPan;
    Float32                             fromTilt;
    Float32                             fromFOV;
    FloatPoint                          fromViewCenter;
    UInt32                              toValidFlags;
    Float32                             toPan;
    Float32                             toTilt;
    Float32                             toFOV;
    FloatPoint                          toViewCenter;
```

Copyright © 2004, 2012 Apple Inc. All Rights Reserved.

```
    Float32                             distance;
    UInt32                              flags;
    UInt32                              reserved1;
    UInt32                              reserved2;
} QTVRLinkHotSpotAtom, *QTVRLinkHotSpotAtomPtr;
```

Angle Range Atom Structure

```
typedef struct QTVRAngleRangeAtom {
    Float32                             minimumAngle;
    Float32                             maximumAngle;
} QTVRAngleRangeAtom, *QTVRAngleRangeAtomPtr;
```

Panorama Sample Atom Structure

```
typedef struct QTVRPanoSampleAtom {
    UInt16                              majorVersion;
    UInt16                              minorVersion;
    UInt32                              imageRefTrackIndex;
    UInt32                              hotSpotRefTrackIndex;
    Float32                             minPan;
    Float32                             maxPan;
    Float32                             minTilt;
    Float32                             maxTilt;
    Float32                             minFieldOfView;
    Float32                             maxFieldOfView;
    Float32                             defaultPan;
    Float32                             defaultTilt;
    Float32                             defaultFieldOfView;
    UInt32                              imageSizeX;
    UInt32                              imageSizeY;
    UInt16                              imageNumFramesX;
    UInt16                              imageNumFramesY;
    UInt32                              hotSpotSizeX;
    UInt32                              hotSpotSizeY;
```

```
    UInt16                                  hotSpotNumFramesX;
    UInt16                                  hotSpotNumFramesY;
    UInt32                                  flags;
    UInt32                                  reserved1;
    UInt32                                  reserved2;
} QTVRPanoSampleAtom, *QTVRPanoSampleAtomPtr;
```

Cubic View Atom Structure

```
struct QTVRCubicViewAtom {
    Float32         minPan;
    Float32         maxPan;
    Float32         minTilt;
    Float32         maxTilt;
    Float32         minFieldOfView;
    Float32         maxFieldOfView;

    Float32         defaultPan;
    Float32         defaultTilt;
    Float32         defaultFieldOfView;
};
typedef struct QTVRCubicViewAtom    QTVRCubicViewAtom;
```

Cubic Face Data Atom Structure

```
struct QTVRCubicFaceData {
    float   orientation[4];
    float   center[2];
    float   aspect;
    float   skew;
};
typedef struct QTVRCubicFaceData    QTVRCubicFaceData;
```

Copyright © 2004, 2012 Apple Inc. All Rights Reserved.

Object Sample Atom Structure

```
typedef struct QTVRObjectSampleAtom {
    UInt16                              majorVersion;
    UInt16                              minorVersion;
    UInt16                              movieType;
    UInt16                              viewStateCount;
    UInt16                              defaultViewState;
    UInt16                              mouseDownViewState;
    UInt32                              viewDuration;
    UInt32                              columns;
    UInt32                              rows;
    Float32                             mouseMotionScale;
    Float32                             minPan;
    Float32                             maxPan;
    Float32                             defaultPan;
    Float32                             minTilt;
    Float32                             maxTilt;
    Float32                             defaultTilt;
    Float32                             minFieldOfView;
    Float32                             fieldOfView;
    Float32                             defaultFieldOfView;
    Float32                             defaultViewCenterH;
    Float32                             defaultViewCenterV;
    Float32                             viewRate;
    Float32                             frameRate;
    UInt32                              animationSettings;
    UInt32                              controlSettings;
} QTVRObjectSampleAtom, *QTVRObjectSampleAtomPtr;
```

Track Reference Entry Structure

```
struct QTVRTrackRefEntry {
    UInt32                      trackRefType;
    UInt16                      trackResolution;
    UInt32                      trackRefIndex;
```

Copyright © 2004, 2012 Apple Inc. All Rights Reserved.

```
};
typedef struct QTVRTrackRefEntry QTVRTrackRefEntry;
```

Copyright © 2004, 2012 Apple Inc. All Rights Reserved.

Profile Atom Guidelines

Note: Profile atoms are deprecated in the QuickTime file format. The information that follows is intended to document existing content containing profile atoms and should not be used for new development.

This appendix introduces and defines some of the ways that profile information about a QuickTime movie file can be summarized in a profile atom near the beginning of the file, so that software reading the file can easily determine some aspects of its features and complexity.

The information in this appendix should not be seen as a replacement for, or even a functional overlap with, the definition of the file-type atom. The file-type atom expresses which specifications a file is compatible with: reading software should not attempt to play files unless they are compatible with one or more specifications the reader implements, and should not refuse to play a file if it is marked as so compatible. However, reading software may use profiling information to issue warnings, request user decisions, and so on.

Reading software should not present excessive warnings to the user in the absence of summarized features. Additionally, readers are encouraged to try to play content even though crucial profile information is missing or incomplete.

Profiles may exist at the movie level or the track level. Track-level profiles summarize features of that track only. Movie-level profiles may summarize features across tracks or summarize features that are only relevant at the movie level (for example, the movie's maximum bit rate).

If the movie contains runtime variables that might affect a feature, such as the presence of alternate tracks that would affect the movie bit-rate, the affected feature should either be absent or report the worst case (for example, the highest bit-rate).

If a feature value cannot be accurately represented (for example, the value is not an integer, but the field is formatted as an integer) then the value should be rounded up to the nearest representable value.

About This Appendix

The technical content of this appendix begins with a discussion of the structure of the profile atom, which holds an array of feature codes and values. Next is an enumeration of the currently included profile features, each described in a feature description section.

The responsibilities placed upon a writer of a movie (such as QuickTime or a consumer electronics (CE) device) are described in the feature's Writer Responsibilities section. A description of the algorithm to be used to calculate values is provided.

The feature's Reader Responsibilities section explains how reading software should interpret the value. In some cases, there are warnings to indicate how the reader must not use the value (for example, not interpreting the maximum bit rate value as the current bit rate).

Profile Atom Specification

Definition

Atom type
: `'prfl'`

Container
: Movie atom (`'moov'`) or track atom (`'trak'`)

Mandatory
: No

Quantity
: Zero or one

At the movie level, the profile atom must occur within the movie atom before the movie header atom. A reader may stop the search for the profile atom once the profile atom or the movie header atom is found. Because new atoms may be introduced into the movie atom (type `'moov'`) in the future, a reader must not expect the first child atom of the movie atom to be either the profile (type `'prfl'`) or the movie header (`'mvhd'`) atom. This rule allows for new atoms in the future but still accommodates readers that do not want to perform an exhaustive enumeration of all the child atoms in a movie atom.

The profile atom expresses profiles or feature codes for features that occur in the movie. The list is not necessarily exhaustive, and there may be multiple profile values recorded for the same profile code. For example, if there are two independent sequences of MPEG-4 video in the movie, using different profile-level IDs, both might be recorded here.

Each feature is either universal or is documented in a specific specification, identified by a brand as used in the file type atom. The only brands that should occur in a given profile atom are the universal brand or brands that occur in the file type atom in the same file.

Copyright © 2004, 2012 Apple Inc. All Rights Reserved.

Feature value ranges should in general never include an unknown point; if the value of a feature is unknown, the feature should be absent from the profile atom.

Feature values should be deducible by fairly simple inspection of the rest of the movie: for example, extracting the profile-level ID from a video header, or calculations using information from the sample table (for example, overall average bit rate by summing the sample sizes and the sample durations). It is not appropriate to have features which cannot be computed, or only computed with difficulty (e.g. a buffer model estimation which requires emulating a video decoder on the entire bit stream). The algorithm to extract or deduce the feature value from the rest of the file must be defined.

Empty slots in the profile atom structure must be filled with zeroes.

If there are multiple parts of the file to which the same feature apply, yet they have different feature values, then either there must be entries for each occurrence or none at all. For example, if there are two MPEG-4 visual sequences, using different visual profiles, there are either two profile entries in the profile table (one for each sequence) or none at all. Features must not be partially documented.

Profile atoms may also occur at the track level. A track-level profile atom must occur within the track atom before the track header atom (`'tkhd'`). A reader should stop searching for a track's profile atom if either the profile or the track header atom is found, ignoring any other atoms present.

A track profile atom should only summarize features within that track. If track profile atoms exist, a movie profile atom can be built largely by copying feature entries from the profile atom of the movie's tracks to the profile atom at the movie level. It is possible to have multiple track profiles with different values which must be resolved to a single value for the movie as whole, however—such as multiple video tracks with different maximum bit rates—so not all features can be copied directly from the track to the movie profile. Additionally, the movie profile may summarize features that cannot occur at the track level, such as total movie bit rate.

When building a movie profile, you must include either all instances of a track-level feature or no instances of that feature. For example, if you have multiple video tracks that use different codecs, you must either include an entry at the movie level for each codec, or put no codec feature entries at the movie level at all.

Copyright © 2004, 2012 Apple Inc. All Rights Reserved.

Figure F-1 (page 396) shows the layout of the profile atom.

Figure F-1 The layout of a profile atom

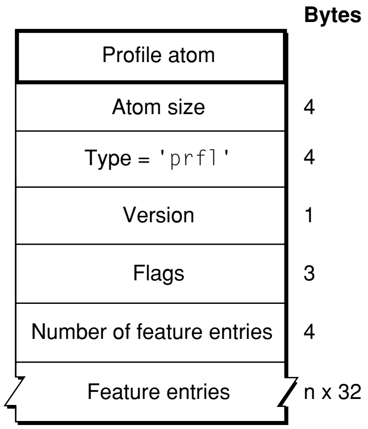

Syntax

```
aligned(8) class ProfileAtom
    extends FullAtom('prfl') {
    unsigned int(32) feature-record-count;
    for (i=1; i<feature-record-count; i++) {
        unsigned int(32) reserved = 0;
        unsigned int(32) part-ID;
        unsigned int(32) feature-code;
        unsigned int(32) feature-value
    }
}
```

Semantics

`reserved`

A 32-bit field that must be set to zero.

`part-ID`

Either a brand identifier that occurs in the file-type atom of the same file, indicating a feature that is specific to this brand, or the value 0x20202020 (four ASCII spaces) indicating a universal feature that can be found in any file type that allows the profile atom. The value 0 is reserved for an empty slot.

Copyright © 2004, 2012 Apple Inc. All Rights Reserved.

`feature-code`

A four-character code either documented here (universal features), or in the specification identified by the brand. The value of 0 is reserved for an empty slot with no meaningful `feature-value`.

`feature-value`

Either a value from an enumerated set (for example, 1 or 0 for true or false, or an MPEG-4 profile-level ID) or a value that can compared (for example, bit rate as an integer or dimensions as a 32-bit packed structure).

The profile atom is a full atom, so it has an 8-bit version and 24 bits of flags. For this specification, the version is 0 and the flags have the value 0. A reader compliant with this specification should treat any profile atom with a nonzero version value as if it did not exist.

Figure F-2 (page 397) shows the layout of a typical feature.

Figure F-2 Layout of a typical feature

	Bytes
Reserved = `0x00000000`	4
Part ID = `'    '(0x20202020)`	4
Feature Code = `'avbr'`	4
Value = `0x00000001`	4

Universal Features

A feature consists of four fields: a reserved field, which is set to zero; a part-ID, which specifies which brand the feature belongs to; a feature code, which identifies the feature; and a value field, which holds the feature value).

The part-ID can be either universal or brand-specific. Universal features have a part-ID of four ASCII spaces (0x20202020). Brand-specific features have a part-ID for a particular brand, which is taken from the Compatible_brands field of the file type atom. Brand-specific features of QuickTime files have a part-ID of `'qt  '`. All features listed in this section are universal features; that is, they can be used in any file that includes a profile atom.

It is permissible to use the feature code of 0x00000000 as a placeholder, paired with a feature value of 0x00000000 for one or more features. Readers should simply ignore features having a feature code of zero.

No feature will exist to describe the unit of other features, such as bit rate. The device should consider the magnitude and tailor its display appropriately.

2012-08-14 | Copyright © 2004, 2012 Apple Inc. All Rights Reserved.

This specification describes only how features are stored in files. It does not require that the values of features be reported (for example, in a user interface) in the same manner as they are stored, or require that they be reported at all.

Table of Features

Table F-1 (page 398) lists the universal features described in this appendix.

Table F-1 Universal features

Brand	Code	Description	Profile Parent
0x20202020	mvbr	"Maximum Video Bit Rate" (page 399)	Movie or Video Track
0x20202020	avvb	"Average Video Bit Rate" (page 400)	Movie or Video Track
0x20202020	mabr	"Maximum Audio Bit Rate" (page 401)	Movie or Track
0x20202020	avab	"Average Audio Bit Rate" (page 403)	Movie or Sound Track
0x20202020	vfmt	"QuickTime Video Codec Type" (page 404)	Movie or Video Track
0x20202020	afmt	"QuickTime Audio Codec Type" (page 405)	Movie or Video Track
0x20202020	m4vp	"MPEG-4 Video Profile" (page 406)	Movie or Video Track
0x20202020	mp4v	"MPEG-4 Video Codec" (page 407)	Movie or Video Track
0x20202020	m4vo	"MPEG-4 Video Object Type" (page 408)	Movie or Video Track
0x20202020	mp4a	"MPEG-4 Audio Codec" (page 409)	Movie or Sound Track
0x20202020	mvsz	"Maximum Video Size in a Movie" (page 411)	Movie
0x20202020	tvsz	"Maximum Video Size in a Track" (page 412)	Movie or Video Track
0x20202020	vfps	"Maximum Video Frame Rate in a Single Track" (page 414)	Movie or Video Track
0x20202020	tafr	"Average Video Frame Rate in a Single Track" (page 415)	Movie or Video Track
0x20202020	vvfp	"Video Variable Frame Rate Indication" (page 416)	Movie or Video Track
0x20202020	ausr	"Audio Sample Rate for a Sample Entry" (page 417)	Movie or Sound Track

Copyright © 2004, 2012 Apple Inc. All Rights Reserved.

Brand	Code	Description	Profile Parent
0x20202020	avbr	"Audio Variable Bit Rate Indication" (page 418)	Movie or Sound Track
0x20202020	achc	"Audio Channel Count" (page 419)	Movie or Sound Track

Maximum Video Bit Rate

Containing profile atom
    Track (video), movie

Reserved
    0x00000000

`part-ID`
    0x20202020 (universal feature)

`feature-code`
    `'mvbr'`

`feature-value`
    Unsigned `int(32)` indicating maximum video bit rate in bits per second

Feature Values

The value is an unsigned 32-bit integer indicating the maximum video bit rate in bits per second. The value may be larger than the actual video bit rate, so it should not be interpreted as a bit rate that will actually occur.

Example: 1 Mbps = 1000000.

Writer Responsibilities

A writer of the maximum video bit rate should record a value that is equal to or greater than the actual bit rate for the video track. A writer (such as a CE device) may choose to record a constant value so long as that value is greater than or equal to the bit rate that may be encoded. It is also permitted to record a value set by the video encoder during initialization, so long as the value is never exceeded.

Feature Value Algorithm

Apple recommends a sliding average over 1 second calculated from the sample tables.

If the feature is written for a newly encoded track (as by a CE device), it is permitted to record a value used to initialize the video encoder so long as the value is never exceeded. If the video track is edited and the maximum video bit rate recalculated, it may be calculated as a sliding average over 1 second, based on the sample table.

2012-08-14 | Copyright © 2004, 2012 Apple Inc. All Rights Reserved.

This can be calculated in the following manner:

1. For each sample, calculate the average 1-second bit rate; choose the shortest run of samples, including the candidate sample, that comprise 1 second or more of video, then divide the total data size of those samples by their total duration.
2. Choose the maximum value from the list of calculated 1-second averages.

Reader Responsibilities

A reader of the maximum video bit rate feature value should compare the recorded value with its own limits to determine if the content can be played. The reader should not perform an equality comparison (=) but instead a relative comparison (<, <=, >, or >=).

The recorded value may be larger than the actual maximum video bit rate. Since this value may be an over-estimate, the reader should not use it as a basis for refusing to play the file, though a warning may be appropriate. To determine the actual bit rate, the reader may need to perform an inspection of the video track's sample table.

Comments

The value of this feature should be deducible from information found in the sample table. Track edits must be considered in its calculation; if the track is edited, this value must be recalculated. Even though this value may exceed the actual maximum video bit rate, writers should attempt to minimize any over-estimation.

Average Video Bit Rate

Containing profile atom
    Track (video), movie

`part-ID`
    0x20202020 (universal feature)

`feature-code`
    `'avvb'`

`feature-value`
    Unsigned `int(32)` indicating average video bit rate in bits per second

Feature Values

The value is an unsigned 32-bit integer indicating the average video bit rate in bits per second.

Example: 1 Mbps = 1000000.

Copyright © 2004, 2012 Apple Inc. All Rights Reserved.

Writer Responsibilities

A writer of the average video bit rate feature should record a value that is equal to or greater than the average bit rate for the video track, measured across all media samples. A writer (such as a CE device) may choose to record a constant value so long as that value is greater than or equal to the average bit rate that may be encoded. It is also permitted to record a value set by the video encoder during initialization so long as the value equals or exceeds the average calculated from the resulting file.

Feature Value Algorithm

Ideally, the long-term average: total sample sizes divided by total sample durations.

If the feature is written for a newly encoded track (as by a CE device), it is permitted to record a value used to initialize the video encoder. If the video track is edited and the average video bit rate recalculated, it may be calculated as an overall average based on the sample table.

Reader Responsibilities

A reader of the average video bit rate feature value should compare the recorded value with its own limits to determine if the content can be played. The reader should not perform an equality comparison (=) but instead a relative comparison (<, <=, >, or >=).

Because a writer may record a larger value than the actual video bit rate, a reader should not interpret this as the actual video bit rate. To determine the current or actual bit rate, the reader may need to perform an inspection of the video track's sample table.

Comments

The value of this feature should be deducible from information found in the sample table. Track edits must be considered in its calculation. Note that for highly variable bit rate video, the average rate may not be a typical rate.

Maximum Audio Bit Rate

Containing profile atom
    Track (sound), movie

`part-ID`
    0x20202020 (universal feature)

`feature-code`
    `'mabr'`

Copyright © 2004, 2012 Apple Inc. All Rights Reserved.

`feature-value`
: Unsigned `int(32)` indicating maximum audio bit rate in bits per second

Feature Values

The value is an unsigned 32-bit integer indicating the maximum audio bit rate in bits per second that must be supported to guarantee playback of the audio. The actual maximum bit rate may be smaller, so a reader should not display this as the current bit rate.

Example: 128 kbps = 128000.

Writer Responsibilities

A writer of the maximum audio bit rate feature should record a value that is equal to or greater than the current bit rate for the sound track. While the value may exceed the actual maximum bit-rate, the writer should attempt to minimize any over-estimation.

While recording the precise bit rate is preferred, it is not required. A writer (such as a CE device) may choose instead to record a constant value so long as that value is greater than or equal to the bit rate that may be encoded. It is also permitted to record a value set by the audio encoder during initialization so long as the value is never exceeded.

Feature Value Algorithm

Apple recommends a sliding average over 1 second calculated from the sample tables.

If the feature is written for a newly encoded track (as by a CE device), it is permitted to record a value used to initialize the audio encoder so long as the value is never exceeded.

If the sound track is edited, and the audio bit rate is not constant, the maximum audio bit rate must be recalculated. Note that editing can change the duration of media samples, resulting in non-constant bit rate audio even when the sound track is encoded using a constant bit rate encoder. Maximum bit rate may be calculated as a sliding average over 1 second, based on the sample table. This can be calculated in the following manner:

1. For each sample, calculate the average 1-second bit rate; choose the shortest run of samples, including the candidate sample, that comprise 1 second or more of audio, then divide the total data size of those samples by their total duration.
2. Choose the maximum value from the list of calculated 1-second averages.

Copyright © 2004, 2012 Apple Inc. All Rights Reserved.

Reader Responsibilities

A reader of this feature code should compare the recorded value with its own limits to determine if the content can be played. The reader should not perform an equality comparison (=) but instead a relative comparison (<, <=, >, or >=).

Because this value may be an over-estimate of the true maximum bit rate, the reader should not refuse to play a file on the basis of this value, although a warning may be appropriate. To determine the current or actual bit rate, the reader may need to perform an inspection of the video track's sample table.

Average Audio Bit Rate

Containing profile atom
    Track (sound), movie

`part-ID`
    0x20202020 (universal feature)

`feature-code`
    `'avab'`

`feature-value`
    Unsigned `int(32)` indicating average audio bit rate in bits per second

Feature Values

The value is an unsigned 32-bit integer indicating the average audio bit rate in bits per second.

Example: 128 kbps = 128000.

Writer Responsibilities

A writer of the average audio bit rate feature should record a value that is equal to or greater than the average bit rate for the sound track, measured across all media samples. A writer (such as a CE device) may choose to record a constant value so long as that value is greater than or equal to the average bit rate that may be encoded. It is also permitted to record a value set by the audio encoder during initialization so long as the value is never exceeded on average.

Feature Value Algorithm

Normally, the long-term average: total sample sizes divided by total sample durations.

If the feature is written for a newly encoded track (as by a CE device), it is permitted to record a value used to initialize the audio encoder. If the sound track is edited and the average video bit rate recalculated, it may be calculated as an overall average based on the sample table.

2012-08-14 | Copyright © 2004, 2012 Apple Inc. All Rights Reserved.

Reader Responsibilities

A reader of the average audio bit rate feature value should compare the recorded value with its own limits to determine if the content can be played. The reader should not perform an equality comparison (=) but instead a relative comparison (<, <=, >, or >=).

Comments

The value of this feature should be deducible from information found in the sample table. Track edits normally need not be considered in the calculation for constant bit rate audio, but must be considered for variable bit rate audio or when track or movie segments containing constant bit rate audio are edited to alter their duration.

QuickTime Video Codec Type

Containing profile atom
: Track (video), movie

`part-ID`
: 0x20202020 (universal feature)

`feature-code`
: `'vfmt'`

`feature-value`
: Unsigned `int(32)` (a four-character-code) holding the QuickTime video codec type copied from the `ImageDescription` structure's `cType` field

Feature Values

This is the four-character-code found in a video sample description.

Examples: 'mp4v', 'jpeg'.

Writer Responsibilities

A writer of the QuickTime video codec type feature should record the four-character code corresponding to the QuickTime video format type or types also recorded in the video track's sample descriptions.

Copyright © 2004, 2012 Apple Inc. All Rights Reserved.

Note: A writer that records the QuickTime Video Codec type for the 'mp4v' codec is encouraged also to write the MPEG-4 Video Profile feature.

Feature Value Algorithm

The feature value is the video codec type read from a QuickTime ImageDescription's `cType` field. If there are multiple sample descriptions with different video codec types, multiple video codec type features should be recorded in the profile atom.

Reader Responsibilities

A reader of this feature code should compare the recorded value by an equality comparison (using =) with the format codes supported by the reader.

QuickTime Audio Codec Type

Containing profile atom
: Track (sound), movie

`part-ID`
: 0x20202020 (universal feature)

`feature-code`
: `'afmt'`

`feature-value`
: Unsigned `int(32)` (a four-character-code) holding the QuickTime audio codec type copied from `SoundDescription` structure's `dataFormat` field

Feature Values

This is the four-character-code found in a sound sample description.

Examples: 'mp4a', 'twos'.

Writer Responsibilities

A writer of the QuickTime audio codec type feature should record the four-character-code corresponding to the QuickTime audio format type or types also recorded in the sound track's sample descriptions.

Copyright © 2004, 2012 Apple Inc. All Rights Reserved.

Note: A writer that records the QuickTime Audio Codec type for the 'mp4a' codec is encouraged also to write the MPEG-4 Audio Codec feature.

Feature Value Algorithm

The feature value is the audio codec type read from a `SoundDescription` structure's `dataFormat` field. If there are multiple sample descriptions with different audio codec types, either all QuickTime Audio Codec Type features must be recorded in the profile atom or none must be recorded.

Reader Responsibilities

A reader of this feature code should compare the recorded value by an equality comparison (using =) with the format codes supported by the reader.

MPEG-4 Video Profile

Containing profile atom
: Track (video), movie

`part-ID`
: 0x20202020 (universal feature)

`feature-code`
: `'m4vp'`

`feature-value`
: Unsigned `int(32)` where least significant 8 bits hold the `profile_and_level_indication` from the `visual_object_sequence`, as defined in specification ISO/IEC 14496-2, retrieved from the video parameters for the MPEG-4 video codec description. The top 24 bits must be set to 0.

Feature Values

The least significant 8 bits hold the value. The most significant 24 bits of the feature value should be set to 0.

Writer Responsibilities

A writer of the MPEG-4 video profile feature should record the 8 bits corresponding to the `profile_and_level_indication` from the `visual_object_sequence`, as defined in specification ISO/IEC 14496-2, found in the video parameters encoded in the esds of the MPEG-4 video codec sample description (with QuickTime codec type 'mp4v').

2012-08-14 | Copyright © 2004, 2012 Apple Inc. All Rights Reserved.

Note: A writer that records the MPEG-4 video profile feature is encouraged also to write the QuickTime Video Codec Type feature.

Feature Value Algorithm

The feature value is the `profile_and_level_indication` from the `visual_object_sequence`, as defined in specification ISO/IEC 14496-2, retrieved from the video parameters for the MPEG-4 video codec description.

Reader Responsibilities

A reader of this feature code should compare the recorded value with the set of profiles and levels supported by the reader.

Comments

This feature may be present only if MPEG-4 video is used. Normally, the video codec type profile entry will also record that MPEG-4 video is present, unless no codec types are present (when, for example, an exhaustive list cannot be formed).

MPEG-4 Video Codec

Containing profile atom
    Track (video), movie

`part-ID`
    0x20202020 (universal feature)

`feature-code`
    `'mp4v'`

`feature-value`
    Unsigned `int(32)` where the least significant 4 bits holds the `visual_object_type` as found in the `VisualObject` (as defined in specification ISO/IEC 14496-2, subclause 6.2.2) found in the esds of the MPEG-4 video codec (QuickTime type 'mp4v') sample description

Feature Values

The least significant 4 bits hold the value. The most significant 28 bits of the feature value should be set to 0.

The list of visual object type constants is defined in specification ISO/IEC 14496-2, subclause 6.3.2.

Example: Video ID is indicated by the value 1.

Copyright © 2004, 2012 Apple Inc. All Rights Reserved.

Writer Responsibilities

A writer of the MPEG-4 Video Codec feature should record the 4 bits corresponding to the `visual_object_type` found in the `VisualObject` within the `ES_descriptor`'s video `DecoderSpecificConfig`. The most significant 28 bits of the value should be set to 0.

> **Note:** A writer that records the MPEG-4 Video Codec feature is encouraged also to write the QuickTime Video Codec Type feature.

Feature Value Algorithm

The MPEG-4 video codec is the 4 bits of the `visual_object_type` found in the `VisualObject`. See ISO/IEC 14496-2, subclause 6.2.2. The `VisualObject` is found in the MPEG-4 Elementary Stream Descriptor Atom within the `'esds'` sample description atom of the video sample description for the QuickTime video codec of type 'mp4v'.

Reader Responsibilities

A reader of this feature code should compare the recorded value with the set of MPEG-4 video decoders supported by the reader.

Comments

Because the QuickTime `'mp4v'` codec may implement multiple video decoders defined in specification ISO/IEC 14496 in the future, this feature allows the reader to determine the specific video decoder needed to interpret the video bit-stream.

MPEG-4 Video Object Type

Containing profile atom
: Track (video), movie

`part-ID`
: 0x20202020 (universal feature)

`feature-code`
: `'m4vo'`

`feature-value`
: Unsigned `int(32)` where the least significant 8 bits hold the `video_object_type_indication` found in the `VideoObjectLayer` (Described in ISO/IEC 14496-2, subclause 6.2.3). The `VideoObjectLayer`

Copyright © 2004, 2012 Apple Inc. All Rights Reserved.

is found in the MPEG-4 Elementary Stream Descriptor Atom within the `'esds'` sample description atom of the video sample description for the QuickTime video codec of type `'mp4v'`.

Feature Values

The value is a video object type constant that indicates a set of video tools. The list of video object type constants is defined in specification ISO/IEC 14496-2, subclause 6.3.3. The least significant 8 bits hold the value. The most significant 24 bits should be set to 0.

Example: The Simple Object Type video object is indicated by the value 1.

Writer Responsibilities

A writer of the MPEG-4 Video Object Type feature should record the 8 bits corresponding to the `video_object_type_indication` found in the `VideoObjectLayer` within the ES_descriptor's video `DecoderSpecificConfig`. The most significant 24 bits of the value should be set to 0.This feature should be written only for MPEG-4 video of video object type 1 (Video ID). If the MPEG-4 video does not use Video ID (1) for `visual_object_type`, the esds will have no `VideoObjectLayer` and consequently no `video_object_type_indication`. In this case, no MPEG-4 Video Object Type feature should be written.

> **Note:** A writer that records the MPEG-4 Video Object Type feature for encoded video using the Video ID visual object type is encouraged to write the MPEG-4 Video Codec and MPEG-4 Video Profile features as well.

Feature Value Algorithm

The MPEG-4 video object type is the least significant 8 bits of the `video_object_type_indication` found in the `VideoObjectLayer`. See ISO/IEC 14496-2, subclause 6.2.3. The `VideoObjecLayer` is found in the MPEG-4 Elementary Stream Descriptor Atom within the `'esds'` sample description atom of the video sample description for the QuickTime video codec of type 'mp4v'.

Reader Responsibilities

A reader of this feature code should compare the recorded value with the set of MPEG-4 video tools supported by the reader.

MPEG-4 Audio Codec

Containing profile atom
    Track (sound), movie

Copyright © 2004, 2012 Apple Inc. All Rights Reserved.

`part-ID`
: 0x20202020 (universal feature)

`feature-code`
: `'mp4a'`

`feature-value`
: Unsigned `int(32)` where least significant 5 bits hold the `AudioObjectType` as found in the `AudioSpecificInfo` (as defined in specification ISO/IEC 14496-3, subclause 1.6) found in the esds of the MPEG-4 audio codec (QuickTime type `'mp4a'`) sample description

Feature Values

The least significant 5 bits hold the value. The most significant 27 bits of the feature value should be set to 0.

The list of audio object type constants is defined in specification ISO/IEC 14496-3, subclause 1.5.1.1.

Examples: AAC LC is indicated by the value 2, CELP is indicated by the value 8.

Writer Responsibilities

A writer of the MPEG-4 Audio Codec feature should record the 5 bits corresponding to the `AudioObjectType` found in the `ES_descriptor`'s audio `DecoderSpecificConfig`. The most significant 27 bits of the value should be set to 0.

> **Note:** A writer that records the MPEG-4 Audio Codec feature is encouraged also to write the QuickTime Audio Codec Type feature.

Feature Value Algorithm

The MPEG-4 audio codec value is the 5 bits of the `AudioObjectType` found in the `AudioSpecificInfo` (a `DecoderSpecificInfo`). See specification ISO/IEC 14496, subclause 1.6. The `AudioSpecificInfo` is found in the MPEG-4 Elementary Stream Descriptor Atom within the `siDecompressionParam` atom of the audio sample description for the QuickTime audio codec of type `'mp4a'`.

Reader Responsibilities

A reader of this feature code should compare the recorded value with the set of MPEG-4 audio decoders supported by the reader.

2012-08-14 | Copyright © 2004, 2012 Apple Inc. All Rights Reserved.

Comments

Because the QuickTime `'mp4a'` codec may implement multiple audio decoders defined in specification ISO/IEC 14496 in the future, this feature allows the reader to determine the specific audio decoder needed to interpret the audio bit stream. The MPEG-4 Audio Codec feature should be present only if the `'mp4a'` audio codec is used in a sound track.

Maximum Video Size in a Movie

Containing profile atom
    Movie

`part-ID`
    0x20202020 (universal feature)

`feature-code`
    `'mvsz'`

`feature-value`
    A 32-bit packed structure holding width and height of the largest bounds needed to display the movie

Feature Values

A packed structure in a 32-bit value:

```
struct {
    unsigned integer(16) width;
    unsigned integer(16) height;
};
```

In big-endian order, the top 16 bits correspond to the width. The lower 16 bits correspond to the height.

Writer Responsibilities

A writer of the Maximum Movie Video Size feature should record a value that is equal to or greater than the display size needed by the movie—the actual width and height needed to display the movie at its normal size, taking into account all matrices (all track matrices and the movie matrix).

A writer (such as a CE device) may choose to record a constant size based upon its current recording mode even if the actual size recorded in the movie is smaller.

2012-08-14 | Copyright © 2004, 2012 Apple Inc. All Rights Reserved.

Feature Value Algorithm

This value is calculated by examining the dimensions of all visual tracks and computing the maximum combined dimensions, including the effect of track matrices and the movie matrix. For example, if two video tracks play side-by-side in the movie, and the tracks and movie all use the identity matrix, this value will be the largest of the two tracks' heights and their combined width.

Reader Responsibilities

A reader of this feature code should compare the recorded value with its own video size limits.

The reader should not interpret the value of this feature as the current video size. To determine the current video size, the reader should use the dimensions of all currently displaying video tracks, their matrices, and the movie matrix.

Comments

The width and height correspond to the maximum visual area needed to display the movie.

The summarized width and height should take into account all components of all track matrices and the movie matrix. The goal is to understand the maximum contribution of all tracks to the movie's bounds.

For the case where there is a single video track with an identity track matrix, the movie's maximum video size feature would typically have the same value as the track's maximum video size feature.

Maximum Video Size in a Track

Containing profile atom
: Track (video), movie

`part-ID`
: 0x20202020 (universal feature)

`feature-code`
: `'tvsz'`

`feature-value`
: A 32-bit packed structure holding width and height of the largest picture buffer needed for a video track.

Feature Values

A packed structure in a 32-bit value:

```
struct {
```

Copyright © 2004, 2012 Apple Inc. All Rights Reserved.

```
    unsigned integer(16) width;
    unsigned integer(16) height;
};
```

In big-endian order, the top 16 bits correspond to the width. The lower 16 bits correspond to the height.

Writer Responsibilities

A writer of the Maximum Track Video Size feature should record a value that is equal to or greater than the largest height and width of any sample description in the video track. This does not include the effect of any scaling or offset applied by the track matrix and may not be the same as the track height and track width.

A writer (such as a CE device) may choose to record a constant size based upon its current recording mode even if the actual size recorded in the track is smaller.

Feature Value Algorithm

Examine all sample descriptions for the track, and use the maximum width and maximum height found in any sample. The maximum width and maximum height may come from independent sample descriptions.

Reader Responsibilities

A reader of this feature code should compare the recorded value with its own image buffer limits.

The reader should not interpret the value of this feature as the current video size. To determine the current video size, the reader should use the dimensions of all currently displaying video tracks, their matrices, and the movie matrix.

Comments

The width and height correspond to the largest image buffer dimensions needed for a visual track. When present in a movie-level profile, these atoms document the maximum video size found in each of the movie's tracks.

The summarized width and height do not take into account any scaling or translation caused by the track or movie matrices, and are not necessarily the same as the track height and width.

For the case where there is a single video track with an identity track and matrix and an identity movie matrix, the movie's maximum video size feature would have the same value as the track's feature.

2012-08-14 | Copyright © 2004, 2012 Apple Inc. All Rights Reserved.

Warning: Use of the "clean aperture" sample description extension does not affect the value of the track visual size, as the picture buffer is needed immediately after decoding, prior to any clean aperture clipping.

Maximum Video Frame Rate in a Single Track

Containing profile atom
Track (video), movie

`part-ID`
0x20202020 (universal feature)

`feature-code`
`'vfps'`

`feature-value`
An unsigned fixed-point (16.16) number holding the maximum video frame rate

Feature Values

This is an unsigned fixed-point (16.16) number holding the maximum video frame rate. The integer portion of the number can range from 0 to 65535.

Examples: 25 fps = 0x00190000; 24 fps = 0x00180000; 29.97 = 0x001DF853 (close approximation of a 30000/1001 ratio). The value may be rounded up to the nearest integer.

Writer Responsibilities

A writer of the Maximum Video Frame Rate feature should record a 16.16 fixed-point value that is equal to or greater than the current video frame rate. A writer (such as a CE device) may choose to record a constant for the feature based on its current recording mode, even if the actual frame rate is less.

A writer of a new video track (such as a CE device recorder) may set the maximum frame rate feature value to a value set during video encoder initialization, so long as this frame rate is never exceeded.

If the current calculated frame rate is fractional (such as 22.3 fps), a writer may choose to round the value up to the nearest integer value (such as 23.0 fps for 22.3 fps).

A writer calculating the video frame rate using the video track's sample table should not consider the first or the last sample duration if they differ from the other sample durations. The reason for this is that captured movie files often have longer or shorter first and last sample durations. By not considering them in the calculation, a more accurate calculation is achieved.

2012-08-14 | Copyright © 2004, 2012 Apple Inc. All Rights Reserved.

Feature Value Algorithm

This feature value may be calculated as the inverse of the smallest sample duration in the video track or tracks.

If the value is written for a newly recorded video track it may be a value established during initialization of the video encoder, so long as the frame rate is not exceeded.

Reader Responsibilities

A reader of this feature code should compare the recorded value with its own video frame rate limits. It should not expect exact values.

The reader should not interpret the value of this feature as the current frame rate. To determine the current frame rate, the reader should use the video track's sample table.

Comments

A writer may choose to round up any fractional value of the fixed-point number to the nearest 16-bit integer leaving the lower 16 bits of the Fixed value set to 0. So, in the case of the 29.97 approximation of 0x001DF853, the writer could round this up to 0x001E0000 (which equals 30).

Average Video Frame Rate in a Single Track

Containing profile atom
: Track (video), movie

`part-ID`
: 0x20202020 (universal feature)

`feature-code`
: `'tafr'`

`feature-value`
: An unsigned fixed-point (16.16) number holding the average video frame rate

Feature Values

This is an unsigned fixed-point (16.16) number holding the average video frame rate. The integer portion of the number can range from 0 to 65535.

Examples: 25 fps = 0x00190000; 24 fps = 0x00180000; 29.97 = 0x001DF853 (close approximation of a 30000/1001 ratio). The value may be rounded up to the nearest integer.

When present in a movie-level profile, these atoms document the average video frame rate of each track in the movie.

Copyright © 2004, 2012 Apple Inc. All Rights Reserved.

Writer Responsibilities

A writer of the Average Video Frame Rate feature should record a 16.16 fixed-point value that is equal to or greater than the average video frame rate. A writer (such as a CE device) may choose to record a constant for the feature based on its current recording mode, even if the actual frame rate is less.

A writer of a new video track (such as a CE device recorder) may set the average frame rate feature value to a value set during video encoder initialization, so long as this frame rate is not exceeded by the actual average, as determined by the feature value algorithm described below.

If the average calculated frame rate is fractional (such as 22.3 fps), a writer may choose to round the value up to the nearest integer value (such as 23.0 fps for 22.3 fps).

Feature Value Algorithm

This feature value is calculated by dividing the the total number of frames (samples) by the duration of the track. It is permissible to omit the first and last frames from this calculation, as they may have significantly different duration than the average.

Reader Responsibilities

A reader of this feature code should understand that each frame is a video sample with its own independent and explicit duration. While it is possible for all frames to have the same duration, it is equally possible for the duration of any frame to be radically different from any other. Therefore, the average frame rate may not always be meaningful information.

The reader should not interpret the value of this feature as the current frame rate. To determine the current frame rate, the reader should use the video track's sample table.

Comments

A writer may choose to round up any fractional value of the fixed-point number to the nearest 16-bit integer leaving the lower 16 bits of the Fixed value set to 0. So, in the case of the 29.97 approximation of 0x001DF853, the writer could round this up to 0x001E0000 (which equals 30).

Video Variable Frame Rate Indication

Containing profile atom

Track (video), movie

`part-ID`

0x20202020 (universal feature)

Copyright © 2004, 2012 Apple Inc. All Rights Reserved.

`feature-code`
: `'vvfp'`

`feature-value`
: Unsigned `int(32)` holding the value 0 if the frame rate is constant or the value 1 if the frame durations vary

Feature Values

The feature value holds one of the following two values: 0 if all video samples have the same display duration, or 1 if any video samples vary in duration.

Writer Responsibilities

A writer of the Video Variable Frame Rate Indication feature should compare the video track sample durations. If all considered durations have the same value, the value 0 indicating constant frame rate should be recorded. If any durations differ, the value 1 should be recorded for the feature. No other value should be recorded.

Feature Value Algorithm

If the Time to Sample table records a constant duration for all samples, then record 0, else record 1.

Reader Responsibilities

A reader of this feature code should only expect the values 0 or 1.

Audio Sample Rate for a Sample Entry

Containing profile atom
: Track (sound), movie

`part-ID`
: 0x20202020 (universal feature)

`feature-code`
: `'ausr'`

`feature-value`
: Unsigned `int(32)` holding the audio sample rate in units per second (for example, 44100 for 44.1 kHz)

Feature Values

This feature value is an unsigned 32-bit integer holding the audio sample rate in units per seconds (cycles per second). The value should be rounded up to the nearest integer if it has a fractional portion.

2012-08-14 | Copyright © 2004, 2012 Apple Inc. All Rights Reserved.

Examples: 24 kHz = 24000, 44.1 kHz = 44100.

Writer Responsibilities

A writer of the Audio Sample Rate feature should record the integer portion (rounded up if there is a fractional portion) of the audio sample rate found in a sound track's `SoundDescription` structure.

If multiple audio sample rates are used in the movie, then either all must recorded in the profile atom, or none must be recorded.

Feature Value Algorithm

This is the integer portion of the sample rate from a QuickTime audio sample description (rounded up if there is a fractional portion). If the sample rate is greater than 64 kHz, the integer portion can be recorded here.

If a sample rate has a fractional portion, the writer should round up to the nearest integer. So, the 22254.54545 value that may occur in QuickTime audio as a Fixed value represented as 0x56EE8BA3 can be recorded as 22255.

Reader Responsibilities

A reader of this feature code should compare the recorded value with its own audio sample rate limits. If the reader only supports discrete values (such as 44100), it can perform equality comparisons (=). If the reader supports ranges of audio sample rates (such as all rates less than or equal to 32000), the reader can perform relative comparisons (<, <=, >, or >=).

Audio Variable Bit Rate Indication

Containing profile atom
: Track (sound), movie

`part-ID`
: 0x20202020 (universal feature)

`feature-code`
: `'avbr'`

`feature-value`
: Unsigned `int(32)` holding the value 0 if the audio is constant bit rate or 1 if the audio is variable bit rate

2012-08-14 | Copyright © 2004, 2012 Apple Inc. All Rights Reserved.

Feature Values

The feature value holds one of the following two values: 0 if the audio is constant bit rate, or 1 if the audio is variable bit rate.

Writer Responsibilities

A writer of the Audio Variable Bit Rate Indication feature should determine if the audio frames are constant or variable bit rate in nature and record either 0 or 1, respectively.

Feature Value Algorithm

Consult the audio sample descriptions. If the `compressionID` field in the sample descriptions is 0 or -1, then the audio is constant bit rate. If the field is -2, then the same algorithm as for video applies: if all the samples have both constant duration and constant size, then the audio is constant bit rate; otherwise it is variable.

Reader Responsibilities

A reader of this feature code should only expect the values 0 or 1.

Audio Channel Count

Containing profile atom
: Track (sound), movie

`part-ID`
: 0x20202020 (universal feature)

`feature-code`
: `'achc'`

`feature-value`
: Unsigned `int(32)` holding the number of audio channels

Feature Values

The feature value is an unsigned 32-bit integer holding the number of audio channels encoded by a Sound Track in the movie. For monaural, the value would be 1. For stereo, the value would be 2. Note that the audio channel count is a standard field in the sound sample description.

Writer Responsibilities

A writer of the Audio Channel Count feature should determine the number of audio channels encoded in the sound track or tracks of the movie.

Copyright © 2004, 2012 Apple Inc. All Rights Reserved.

Feature Value Algorithm

Consult the audio sample descriptions.

Reader Responsibilities

The reader should be prepared to either play the specified number of channels or to map the audio to the number of channels the reader supports (for example, mixing down stereo sound for a monaural speaker).

2012-08-14 | Copyright © 2004, 2012 Apple Inc. All Rights Reserved.

Audio Priming - Handling Encoder Delay in AAC

This appendix describes temporal positioning of a source audio signal after AAC encoding into a sound track for QuickTime media files. The mechanisms described here are specified in ISO MPEG-4 standards (ISO/IEC 14496-12, 2008) and are used here with additional constraints.

Note on language use:

AAC implementations typically represent 1024 **PCM** audio samples in one **AAC** packet (synonymous in this context with a QuickTime media sample, and also referred to in ISO documents as an "access unit"). The terms "sample" and "audio sample" in this appendix are used to refer to PCM samples. For the encoded audio data, the terms "AAC packet" and QuickTime "media sample" are used.

Background – AAC Encoding

AAC requires data beyond the source PCM audio samples in order to correctly encode and decode audio samples due to the nature of the encoding algorithm. AAC encoding uses a transform over consecutive sets of 2048 audio samples, applied every 1024 audio samples (overlapped). For correct audio to be decoded, both transforms for any period of 1024 audio samples are needed. For this reason, encoders add at least 1024 samples of silence before the first 'true' audio sample, and often add more. This is called variously "priming", "priming samples", or "encoder delay". A couple of definitions for use in this discussion:

- **Encoder delay** is the delay incurred during encoding to produce properly formed, encoded audio packets. It typically refers to the number of silent media samples (priming samples) added to the front of an AAC encoded bitstream.
- **Decoder delay** is the number of "pre-roll" audio samples required to reproduce an encoded source audio signal for a given time index. For AAC this number is typically 1024 and is algorithmically based. This is in contrast to encoder delay which is determined by the encoder and encoding configuration used. However, decoder delay establishes the minimum encoder delay possible (that is, 1024 for AAC).

The common practice is to propagate the encoder delay in the AAC bitstream. When these audio packets are then decoded back to the PCM domain, the source waveform represented will be offset in its entirety by this encoder delay amount. Since encoded audio packets hold a fixed number of audio samples (for instance, 1024 samples) additional trailing or 'remainder' silent samples following the last source sample are required so as to pad the final audio packet to the required length.

2012-08-14 | Copyright © 2004, 2012 Apple Inc. All Rights Reserved.

Figure G-1 (page 422) gives an example of a typical encoded AAC audio bitstream. The upper portion of the illustration represents the AAC encoded domain with equal-sized AAC packets, the lower portion represents the PCM sample domain:

Figure G-1 AAC encoded audio

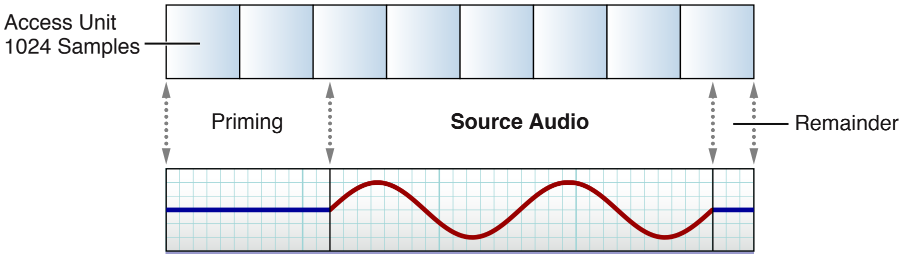

The source audio to be encoded, shown as the red waveform, is 5389 samples long. You can see how it is represented with the blue priming and remainder samples against the fixed size access units–AAC packets–drawn above it.

This data will be represented in 8 AAC packets, where each packet represents 1024 audio samples. The total duration represented by these 8 AAC packets is 8192 audio samples (note that this is longer than the duration of the source audio).

The result breaks down into the following values:

2112 priming samples at the start—Required to correctly encode the start of the audio.

5389 samples of actual audio.

691 remainder samples—Required to pad out to the AAC packet size.

Therefore, to correctly extract the original 5389 samples of source audio, the first 2112 samples of priming and the last 691 samples of the remainder must be removed.

8192 - 2112 - 691 = 5389 original source samples.

The Timing and Synchronization Problem

If an audio playback system attempting to synchronize AAC encoded audio and video does not compensate for encoder delay (that is, does not discard the silent priming samples), the audio and video will be out of synchronization. In the example above, it will be off by 2112 samples—The audio will be 2112 samples behind the video because the first real audio sample is actually the 2113th sample after the beginning of the decoded PCM data.

Copyright © 2004, 2012 Apple Inc. All Rights Reserved.

Therefore, a playback system must trim the silent priming samples to preserve correct synchronization. This trimming by the playback system should be done in two places:

- When playback first begins
- When the playback position is moved to another location. For example, the user skips ahead or back to another part of the media and begins playback from that new location.

Historical Solution—Implicit Encoder Delay

In the original AAC implementations, as stated above the common practice was to propagate the encoder delay in the provided AAC bitstream. With these original implementations, the most common delay used was 2112 audio samples. An AAC bitstream would therefore generally be 3 AAC packets larger than what was theoretically required by the original signal.

A playback implementation would then need to discard these first 2112 silent samples from the decoded output since they contain none of the original source audio data; these samples are an artifact of the encoding/decoding process.

Because there was no explicit way to represent the extent of the priming or remainder samples with the first implementations of an AAC bitstream, Apple chose to assume that the AAC bitstream always contained an encoding delay with a fixed number of samples and advised developers accordingly. A fixed encoder delay of 2112 samples was chosen because at that time this was the common encoding delay used, for various reasons, by most of the shipping implementations of AAC encoders (commercial and otherwise).

Note:

The lack of explicit representation for encoder delay and remainder samples is not a problem unique to AAC encoding. With MPEG-4 and ADTS/MPEG-2 bitstreams and file containers, there is still no satisfactory, explicit representation for either the encoder delay or remainder samples. MP3 also has these data dependencies and delays in its bitstream, as do proprietary codecs such as AC-3 and others.

In all of these cases the conventional solution is as described above: an implicit assumption is made about the size of the encoder delay and the playback engine is required to trim this designated number of samples from its output at the start of playback. Adjustments must also be made for the remainder samples as required.

In summary, the historical technique to handle the timing and synchronization problem is to assume an *implicit* 2112 sample standard encoder delay in AAC data streams and indicate start time—the first media sample or AAC packet—in the sound track edit list (see "Edit List Atoms" (page 70)) at the start of encoder delay.

2012-08-14 | Copyright © 2004, 2012 Apple Inc. All Rights Reserved.

Using Track Structures to Represent Encoder Delay Explicitly

In QuickTime movie files (.mov) and related MPEG-4 files, AAC encoded audio is carried in a sound track as a series of media samples—each media sample corresponding to an AAC encoded audio packet. A track uses an edit list (see "Edit List Atoms" (page 70)) to indicate the range of time from the media samples to present. The edit list atom along with additional atoms known as Sample Group Structures, introduced in "Sample Group Structures" (page 425), can now be used to explicitly represent encoder delay.

> **Important:** A *complete* implementation using the sample group structures is required to explicitly represent the placement of the source signal in the encoded track. An incomplete implementation will result in unspecified interpretation by Apple software and tools. In the absence of the sample group structures, the classic solution of expecting an implicit encoding delay of 2112 samples and the edit list to start at the beginning of encoder delay will be assumed as described in the previous section.

Edit List Atom

See "Edit Atoms" (page 69) and "Edit List Atoms" (page 70) for details of edit lists in track atom structures.

A sound track of AAC encoded audio uses an edit list to indicate the placement of the source signal in the time represented by the encoded AAC packets. The media time field of the edit list must indicate the first sample to be presented and will correspond in time to the first audio sample following the encoder delay in that track. The edit list track duration field should be set to the duration of the source waveform in media samples. The edit list must not extend into the encoder delay or into any remainder samples of the encoded sound track. Note that for a single waveform encoded into a sound track, the sound track requires only a single edit list atom with one entry.

2012-08-14 | Copyright © 2004, 2012 Apple Inc. All Rights Reserved.

Note: Track duration uses the movie timescale instead of the media timescale used by media time. If the media timescale and movie timescale differ, the track duration may not be sample accurate.

Sample Group Structures

Note: This implementation of sample group structures in QuickTime is designed to be compatible with the ISO Sample Group Structures definition and allow for future generalization. A full description of ISO 14996-12 Sample Group Structures is not required in order to provide explicit encoder delay representation. A short description of the more general characteristics of these structures is included in the descriptions below, followed by the information required for use with QuickTime.

Sample group structures of roll-group type with a constant roll distance are used to represent decoder dependencies for AAC encoded media. The sample group structures are intended to serve two purposes:

- To indicate the amount of decoder delay in AAC packets
- To signal to readers parsing QuickTime movies that the sound track includes explicit information for encoder delay and remainder samples for the AAC packets encoded in the file

Note: The effect of using sample group structures in the track in this manner is that the edit list's media time and track duration fields do not include encoder delay, as specified above in "Edit List Atom" (page 424).

Two sample group structure atoms are used to represent the amount of encoder delay and remainder samples which must be trimmed.

Sample Group Description Atom

Sample group description atoms give information about the characteristics of sample groups. The sample group description atom has an atom type of `'sgpd'`.

2012-08-14 | Copyright © 2004, 2012 Apple Inc. All Rights Reserved.

In a general case:

Each instance of a sample group description atom has a type code that distinguishes different sample groupings. There can be multiple instances of this atom if there is more than one sample grouping for the samples in a track. At most one instance of a sample group description with a particular grouping type exists in a track. An associated sample-to-group atom has the same value associated with that grouping type. The information or "payload data" is stored in the sample group description atom, after the entry count, as an array of entries for which the meanings vary according to the characteristics of grouping type.

For use in AAC encoder delay representation, there is one instance of a sample group description atom in a given QuickTime sound track with grouping type `'roll'`. The specifics for audio data (`AudioRollRecovery()`) are used and articulate the rolling decode dependency. Because the sample group description atom for this purpose is describing the entirety of the AAC audio stream, the payload data field resolves to a single signed 16-bit integer representing the roll distance, which is set to -1. In other words, one AAC packet (1024 encoded PCM audio samples) preceding the media sample is indicated as being of the same type as the encoded source data, allowing the decode transform to operate over the required two AAC packets for the first media sample specified in the edit list.

Note: The payload data value (roll distance in this use) of -1 is a typical value for existing AAC codecs, but the payload data can have other values. Codecs could use alternative values depending upon their implementation details.

Figure G-2 (page 426) shows the layout of this atom.

Figure G-2 The layout of a sample group description atom

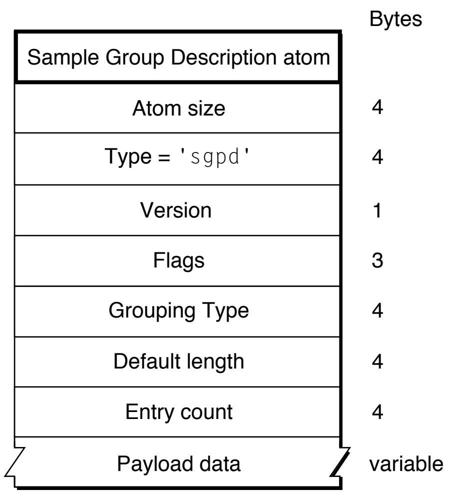

2012-08-14 | Copyright © 2004, 2012 Apple Inc. All Rights Reserved.

The sample group description atom contains the following data elements:

Size
: A 32-bit integer that specifies the number of bytes in this sample group description atom.

Type
: A 32-bit integer that identifies the atom type, set to `'sgpd'`.

Version
: A 1-byte specification of the version of this sample group description, set to 1.

Flags
: A 3-byte reserved space, set to 0

Grouping type
: A 32-bit integer that identifies the grouping type of this sample group description, set to `‘roll’`.

Default length
: A 32-bit integer indicating the length of the group entry in the payload data, set to 2 (bytes).

Entry count
: A 32-bit integer giving the number of entries in the payload data field, set to 1.

Payload data
: A 16-bit signed integer giving the roll distance, set to -1 value for AAC audio.

Sample-To-Group Atoms

Sample-to-group atoms are used to find the group that a sample belongs to and the associated description of that sample group. The sample-to-group atom has an atom type of `‘sbgp’`.

2012-08-14 | Copyright © 2004, 2012 Apple Inc. All Rights Reserved.

In a general case:

There may be multiple instances of sample-to-group atoms if there is more than one sample grouping for the samples in a track. Each instance of the sample-to group atom has a grouping type code that distinguishes different sample groupings. Within a track there can be at most one instance of this atom with a particular grouping type. An associated sample group description atom indicates the same value for the grouping type.

The sample-to-group atom contains a table with a sample count and group description index pairs. The sample count is the number of media samples in the run of samples with the same sample group description. The group description index is an index into the array of payload data entries in the associated sample group description atom's payload data table, the association defined by having the same grouping type value.

For use in AAC encoder delay representation, there is one sample-to-group atom instance in a given QuickTime sound track with grouping type `‘roll’` matching the single instance of the sample group description atom. The entry count field value is set to 1, indicating one entry in the table data array. That entry is describing all the AAC packets in the track. The sample count in the table data array is typically the same as the sample size atom’s number of entries field, see “Sample Size Atoms” (page 111), which represents the number of media samples in the track (in this use, AAC packets). For AAC encoder delay representation, the only entry in the associated sample group description atom’s payload data table is the first, which provides the value of 1 for the group description index.

Figure G-3 (page 428) shows the layout of this atom.

Figure G-3 The layout of a sample-to-group atom

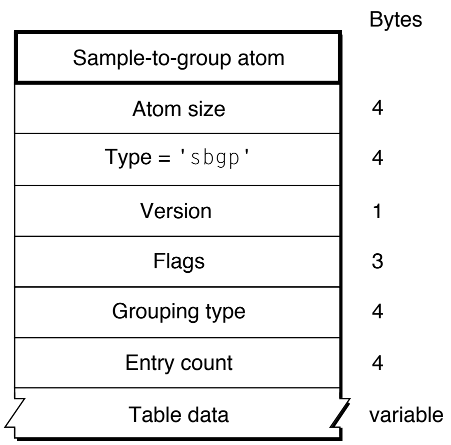

The sample-to-group atom contains the following data elements:

2012-08-14 | Copyright © 2004, 2012 Apple Inc. All Rights Reserved.

Size

A 32-bit integer that specifies the number of bytes in this sample-to-group atom.

Type

A 32-bit integer that identifies the atom type; set to `'sbgp'`.

Version

A 1-byte specification of the version of this sample-to-group atom, set to 0

Flags

A 3-byte reserved space, set to 0.

Grouping type

A 32-bit integer identifying the grouping type, set to `‘roll’`.

Entry count

A 32-bit integer giving the number of entries in the table table data that follows.

Table data

A table of sample count and group description index pairs as shown in Figure G-4 (page 429).

Figure G-4 The layout of the table data format

Sample count	Group description Index	Field
4	4	Bytes

Sample count

A 32-bit integer that provides the number of consecutive media samples with the same sample group descriptor. The value is typically the same as in the sample size atom’s number of entries field.

Group description index

A 32-bit integer the value of which is the index into the sample group description atom’s payload data table which describes the samples in this group. The index ranges from 1 to the number of payload data entries in the sample group description atom, or takes the value 0 to indicate that this group of samples is a member of no group of this type.

Example—Representing Encoder Delay Explicitly

Consider the following example of a typical case of PCM source sound data to be encoded as AAC:

2012-08-14 | Copyright © 2004, 2012 Apple Inc. All Rights Reserved.

The goal is to represent the temporal position of 5 seconds of 48kHz PCM audio encoded in a 48kHz AAC sound track. Assume a media timescale of 48000 and an encoder delay of 2112. For convenience, assume a movie timescale of 48000 as well.

Audio Data

Source PCM audio data prior to encoding:

Sample rate: 48000 per second

Sample count: 240000 PCM samples (5 seconds)

Duration in timescale ticks: 240000 (with media timescale the same as sample rate)

AAC in the encoded sound track:

Encoder delay: 2112 audio samples

Samples per AAC packet: 1024

Decoder delay: 1024 samples (or 1 AAC packet)

Number of AAC packets: 237 (=((2112+240000) / 1024) rounded up to an integer value)

Sample count: 242688 (= 237 * 1024)

Remainder samples: 576 (= 242688 - (2112 + 240000))

The transformation from source PCM to encoded AAC results in a sound track with 237 AAC media samples corresponding to 242688 PCM audio samples if decoded and presented in its entirety. Of that total, only 240000 audio samples of source starting at sample offset 2112 (skipping the first 2111 samples) are to be presented. From this, the edit list atom and sample group atom described in "Track Structures" (page 430) are used to represent the encoder delay.

Track Structures

Based on the "Audio Data" (page 430), the following edit list and sample group atoms are used to represent the encoder delay.

The edit list atom contains this data:

Size: 28

Type: `'elst'`

Version: 0

Flags: 0

2012-08-14 | Copyright © 2004, 2012 Apple Inc. All Rights Reserved.

Entry count: 1

Number of entries: 1

with this table data:

Track duration: 240000 (source duration)

Media time: 2112 (accounts for the encoder delay)

Media rate: 1.0

Note: For this example, the conventional encoder delay value of 2112 audio samples has been used. Alternative encoder delay values can also be explicitly represented with this mechanism, depending upon codec implementation.

The sample group description atom contains this data:

Size: 26

Type: `'sgpd'`

Version: 1

Flags: 0

Grouping type: `'roll'`

Default length: 2

Entry count: 1

with this table data:

payload data: -1

Note: Recall that the payload data value could be an alternative value, depending upon the particular codec in use. -1 is typical for current AAC codecs.

And the sample-to-group atom contains this data:

Size: 22

Type: `'sbgp'`

Version: 0

Flags: 0

2012-08-14 | Copyright © 2004, 2012 Apple Inc. All Rights Reserved.

Grouping type: `'roll'`

Entry count: 1

with this table data:

Sample count: 237 (number of AAC packets representing the encoder delay, original audio samples, and remainder audio samples)

Group description index: 1 (corresponds to first and only entry in the sample group description atom's payload data table for these sample units)

Other things to note from this example:

- You cannot use the edit list by itself to determine the encoder delay or remainder sample count. The sample group atoms provide the encoder delay. The placement of the end of the edit in a compressed audio packet allows calculation of the remainder samples.
- If the encoder delay was the theoretical minimum for AAC of 1024, then the media time field value in the edit list table data shown in this example would be 1024, not 2112.

Summary—Using Track Structures to Represent Encoder Delay

When using sample group structures in representing encoder delay for AAC sound tracks:

- Include a version 1 sample group description atom with grouping type set to `'roll'`. Set default length to 2 (bytes) for audio entries. Follow that with the payload data: the typical value is -1, meaning one preceding AAC packet, which is the theoretical minimum decoder delay of 1024 samples.
- Include a version 0 sample-to-group atom with a `'roll'` grouping type. By including this, you associate the AAC packets with the corresponding sample group description atom. All AAC packets including the encoder delay must be associated with the sample group in the table data's sample count field. Typically, the sample count for this sample-to-group atom's table data corresponds with the number of media samples in the track.

These two sample group structure atoms in addition to the edit list atom, properly composed, form a complete implementation to explicitly represent the temporal position of the source audio samples in an AAC encoded track.

Copyright © 2004, 2012 Apple Inc. All Rights Reserved.

Document Revision History

This table describes the changes to *QuickTime File Format Specification* .

Date	Notes
2012-08-14	Corrected the values for kQTSampleDependency_OtherSamplesDependOnThisSample and kQTSampleDependency_NoOtherSampleDependsOnThisSample.
2012-08-01	The following updates are included in this revision: In "User Data Atoms" (page 47), expanded the description of `'tnam'` and added `'tagc'` with related section "Media Characteristic Tags" (page 52). Added "Track Exclude From Autoselection Atoms" (page 61). Explained alternate groups in "Track Header Atoms" (page 57). Added `clcp`, `fall`, `folw`, and `forc` to "Track Reference Atoms" (page 73). Corrected structure of gmhd and gmin in "Base Media Information Atoms" (page 89). "General Structure of a Sample Description" (page 100) includes emphasized importance of data size due to occasional terminating zeroes. Added "Text Media Information Atom" (page 196), "Closed Captioning Media" (page 198), and "Subtitle Media" (page 199). "Timecode Media Information Atom" (page 191) now documents the reserved integer after the text size. Added "Preparing Sound and Subtitle Alternate Groups for Use with Apple Devices" (page 334). "Audio track" changed to "sound track" for consistency. Deprecated 'rsrc' data reference.

Date	Notes
2011-07-13	The following updates are included in this revision: The sound sample description v2 format along with the definition of two new sound sample description extensions are incorporated, see "Sound Sample Description (Version 2)" (page 181). New atoms for the display of track aperture in different modes have been documented, see "Track Aperture Mode Dimension Atoms" (page 61). New sample atoms for handling out-of-order movie samples have been added, see "Sample Atoms" (page 95). Appendix G, "Audio Priming - Handling Encoder Delay in AAC" (page 421), has been added which treats how to handle temporal positioning of AAC audio data explicitly. The Macintosh language codes table has been updated with current language names, see Table 5-1 (page 317) and the related "Extended Language Tag Atom" (page 82) defined.
2010-08-03	Corrected the order of fields described in the Metadata Handler Atom structure.
2010-05-03	Added description of clip-based metadata and specific key/value pairs for location metadata.
2007-09-04	First public release of complete, updated *QuickTime File Format Specification* with information about atoms and atom types. Added licensing information and disclaimer for developers. Modified introductory sections and atom descriptions; updated artwork and edited for technical accuracy.

2012-08-14 | Copyright © 2004, 2012 Apple Inc. All Rights Reserved.

Date	Notes
	A QuickTime file may now contain a file type compatibility atom. See "The File Type Compatibility Atom" (page 33). A movie atom may now contain a movie profile atom. See "The Movie Profile Atom" (page 42). A track atom may now contain a track profile atom. See "Track Profile Atom" (page 57). Video sample descriptions may now contain a pixel aspect ratio atom for non-square pixels. See "Pixel Aspect Ratio ('pasp')" (page 159). Video sample descriptions may now also contain a color parameter atom. See "Color Parameter Atoms ('colr')" (page 161). Video sample descriptions may now a clean aperture atom. See "Clean Aperture ('clap')" (page 167). The sound description record has been expanded to represent variable bit-rate compression more accurately. See "Sound Sample Descriptions" (page 176). The section describing MPEG-4 audio has been modified. See "MPEG-4 Audio" (page 189).<br><br>It is now recommended that the file creation and modification times be set using UTC, rather than local time zones. See "Calendar Date and Time Values" (page 320). User data text may now be encoded using either Macintosh text encoding or ISO text encoding (Unicode). See "User Data Text Strings and Language Codes" (page 51). MPEG-4 video and audio sample descriptions may now contain elementary stream descriptor atoms. See "MPEG-4 Elementary Stream Descriptor Atom ('esds')" (page 160) and "MPEG-4 Elementary Stream Descriptor Atom ('esds')" (page 186). It is now possible to specify languages using either Macintosh language codes or ISO language codes. See "Language Code Values" (page 316).

2012-08-14 | Copyright © 2004, 2012 Apple Inc. All Rights Reserved.

Glossary

AAC Advanced Audio Coding (AAC) is a standardized, lossy compression and encoding scheme for digital audio. AAC generally achieves better sound quality than MP3 encoding at similar bit rates.

alpha channel The upper bits of a display pixel, which control the blending of video and graphical image data for a video digitizer component.

alternate group A set of related movie tracks (alternate tracks) that contain the same alternate group identifier in the track header.

alternate track A movie track that contains alternate data for another track. QuickTime chooses one track from an alternate group to be used when the movie is played. The choice may be based on such considerations as image quality or localization. See also track.

API (Application Programming Interface) The set of function calls, data structures, and other programming elements by which a structure of code (such as a system-level toolbox) can be accesses by other code (such as an application program).

atom The basic unit of data in a movie resource, sprite, or other QuickTime data structure. There are a number of different atom types. There are two varieties of atoms: QT atoms, which may contain other atoms, and classic atoms, which do not contain any other atoms. See also movie resource, sprite , QT atom, and classic atom.

atom container A tree-structured hierarchy of QT atoms. See also QT atom.

atom ID A 32-bit integer that uniquely identifies an atom among other child atoms of the same parent atom. The root atom has an atom ID value of 0x0001. See also child atom, parent atom , and root atom.

atom type A 32-bit value that uniquely identifies the data type of an atom. It is normally an `OSType`, rendered by four ASCII characters. An atom's data type helps determine how the atom's contents are interpreted.

audio track An alternative name for the sound track for a QuickTime file. See sound track.

background color The color of the background behind a sprite or other image.

bit depth The number of bits used to encode the color of each pixel in a graphics buffer.

chapter list A set of named entry points into a movie, presented to the viewer as a text list.

child atom A QT atom inside a container atom, which is its parent atom. See also QT atom, container atom , and parent atom.

chunk A collection of sample data in a media. Chunks, which may contain one or more samples, allow optimized data access. Chunks in a media may have different sizes, and the samples within a chunk may have different sizes.

2012-08-14 | Copyright © 2004, 2012 Apple Inc. All Rights Reserved.

classic atom A QuickTime atom that contains no other atoms. A classic atom, however, may contain a table. An example of a classic atom is an edit list atom, containing the edit list table. Compare QT atom.

clipped movie boundary region The region that combines the union of all track movie boundary regions for a movie, which is the movie's movie boundary region, with the movie's movie clipping region, which defines the portion of the movie boundary region that is to be used. See also movie boundary region and movie clipping region.

clipping The process of defining the boundaries of a graphics area.

closed caption track The closed caption text for a QuickTime movie, which is typically a direct transcription of a sound track and which is stored in a track with a media handler type of `'clcp'`.

container atom An atom that contains other atoms, possibly including other container atoms.

creator signature In the Macintosh file system, a four-character code that identifies the application program to which a file belongs.

data fork In a legacy two-fork Macintosh file, the section that corresponds to a DOS/Windows file.

data handler A piece of software that is responsible for reading and writing a media's data. The data handler provides data input and output services to the media's media handler. See also media handler.

data reference A reference to a media's data.

decoder delay The number of "pre-roll" audio samples needed to reproduce the source audio signal in an encoded audio stream for a given time index. See also encoder delay.

display coordinate system The QuickDraw graphics world, which can be used to display QuickTime movies, as opposed to the movie's time coordinate system, which defines the basic time unit for each of the movie's tracks. Compare time coordinate system.

dithering A technique used to improve picture quality when you are attempting to display an image that exists at a higher bit-depth representation on a lower bit-depth device. For example, you might want to dither a 24 bits per pixel image for display on an 8-bit screen.

dropframe A synchronizing technique that skips time codes to keep them current with video frames.

duration A time interval. Durations are time values that are interpreted as spans of time, rather than as points in time.

edit list A data structure that arranges a media into a time sequence.

edit state Information defining the current state of a movie or track with respect to an edit session. QuickTime uses edit states to support undo facilities.

effect description A data structure that specifies which component will be used to implement an effect in a movie, and how the component will be configured.

effect track A modifier track that applies an effect (such as a wipe or dissolve) to a movie. See modifier track.

encoder delay The term used to describe the delay incurred during encoding to produce properly formed, encoded audio packets. See also decoder delay and "Background – AAC Encoding" (page 421).

file fork A section of a Macintosh file. See also data fork, resource fork.

2012-08-14 | Copyright © 2004, 2012 Apple Inc. All Rights Reserved.

file preview A thumbnail picture from a movie that is displayed in the Open File dialog box. See also thumbnail picture.

fixed point A point that uses fixed-point numbers to represent its coordinates. QuickTime uses fixed points to provide greater display precision for graphical and image data.

fixed rectangle A rectangle that uses fixed points to represent its vertices. QuickTime uses fixed rectangles to provide greater display precision.

Flash A vector-based graphics and animation technology. Flash data is exported as SWF files.

flattening The process of copying all of the original data referred to by reference in QuickTime tracks into a QuickTime movie file. This can also be called resolving references. Flattening is used to bring in all of the data that may be referred to from multiple files after QuickTime editing is complete. It makes a QuickTime movie stand-alone—that is, it can be played on any system without requiring any additional QuickTime movie files or tracks, even if the original file referenced hundreds of files. The flattening operation is essential if QuickTime movies are to be used with CD-ROM discs.

frame A single image in a sequence of images. For uncompressed audio, one sample from each channel. For compressed audio, a compressed group of samples whose format is dependent on the compressor.

frame rate The rate at which a movie is displayed—that is, the number of frames per second that are actually being displayed. In QuickTime the frame rate at which a movie was recorded may be different from the frame rate at which it is displayed. On very fast machines, the playback frame rate may be faster than the record frame rate; on slow machines, the playback frame rate may be slower than the record frame rate. Frame rates may be fractional.

free atom An atom of type '`free`', which you can include in a QuickTime file as a placeholder for unused space.

file type atom An atom of type '`ftyp`', which defines which file specifications a file is compatible with.

graphics mode The method by which two overlapping images are blended together to produce a composite image.

graphics world A software environment in which a movie track or set of images may be defined before importing them into a movie.

handler reference atom A QT atom of type 'hdlr' that specifies the media handler to be used to interpret a media. See also QT atom, media, media handler.

hint track A track in a streaming movie that contains information for a packetizer about the data units to stream. See also streaming.

hot spot An area, typically in a VR presentation, that the user can click to invoke an action.

hypertext Action media that contains a URL and takes the user to a website.

identity matrix A transformation matrix that specifies no change in the coordinates of the source image. The resulting image corresponds exactly to the source image. See also transformation matrix.

image In sprite programming, one of a sprite's properties. See also sprite, PCM.

2012-08-14 | Copyright © 2004, 2012 Apple Inc. All Rights Reserved.

image sequence A series of visual representations usually represented by video over time. Image sequences may also be generated synthetically, such as from an animation sequence.

image track Any track in a QuickTime movie that contains visual images. The term particularly applies to video tracks that contain VR data.

input map A data structure that describes where to find information about tracks that are targets of a modifier track. See modifier track.

interlacing A video mode that updates half the scan lines on one pass and goes through the second half during the next pass.

interleaving A technique in which sound and video data are alternated in small pieces, so the data can be read off disk as it is needed. Interleaving allows for movies of almost any length with little delay on startup.

ISO Acronym for the International Standards Organization. ISO establishes standards for multimedia data formatting and transmission, such as JPEG and MPEG.

Joint Photographic Experts Group (JPEG) Refers to an international standard for compressing still images. This standard supplies the algorithm for image compression. The version of JPEG supplied with QuickTime complies with the baseline ISO standard bitstream, version 9R9. This algorithm is best suited for use with natural images.

key frame A sample in a sequence of temporally compressed samples that does not rely on other samples in the sequence for any of its information. Key frames are placed into temporally compressed sequences at a frequency that is determined by the key frame rate. Typically, the term key frame is used with respect to temporally compressed sequences of image data. See also sync sample. See also key frame rate.

key frame rate The frequency with which key frames are placed into temporally compressed data sequences. See also key frame.

layer A mechanism for prioritizing the tracks in a movie or the overlapping of sprites. When it plays a movie, QuickTime displays the movie's tracks according to their layer—tracks with lower layer numbers are displayed first; tracks with higher layer numbers are displayed over those tracks.

leaf atom An atom that contains only data, and no other atoms.

LPCM Linear pulse-code modulation (LPCM) is a method of encoding audio information digitally. The term also refers collectively to formats using this method of encoding. The term pulse-code modulation (PCM), though strictly more general, is often used to describe data encoded as LPCM. LPCM is PCM with linear quantization. See also PCM.

matrix See transformation matrix.

matte A defined region of a movie display that can be clipped and filled with another display.

media A data structure that contains information that describes the data for a track in a movie. Note that a media does not contain its data; rather, a media contains a reference to its data, which may be stored on disk, CD-ROM disc, or any other mass storage device. Also called a *media structure*.

media handler A piece of software that is responsible for mapping from the movie's time coordinate system to the media's time coordinate system. The media handler also interprets the

2012-08-14 | Copyright © 2004, 2012 Apple Inc. All Rights Reserved.

media's data. The data handler for the media is responsible for reading and writing the media's data. See also data handler.

MIDI Acronym for Musical Instrument Digital Interface, a standard format for sending instructions to a musical synthesizer.

modifier track A track in a movie that modifies the data or presentation of other tracks. For example, a tween track is a modifier track. See also tween track.

movie A structure of time-based data that is managed by QuickTime. A movie may contain sound, video, animation, or a combination of any of these types of data. A QuickTime movie contains one or more tracks; each track represents a single data stream in the movie. See also time-based data, track.

movie boundary region A region that describes the area occupied by a movie in the movie coordinate system, before the movie has been clipped by the movie clipping region. A movie's boundary region is built up from the track movie boundary regions for each of the movie's tracks. See also movie clipping region, track movie boundary region.

movie clipping region The clipping region of a movie in the movie's coordinate system. QuickTime applies the movie's clipping region to the movie boundary region to obtain a clipped movie boundary region. Only that portion of the movie that lies in the clipped movie boundary region is then transformed into an image in the display coordinate system. See also movie boundary region.

movie display boundary region A region that describes the display area occupied by a movie in the display coordinate system, before the movie has been clipped by the movie display clipping region. See also movie display clipping region.

movie display clipping region The clipping region of a movie in the display coordinate system. Only that portion of the movie that lies in the clipping region is visible to the user. QuickTime applies the movie's display clipping region to the movie display boundary region to obtain the visible image. See also movie display boundary region.

movie file A QuickTime file that stores a movie and its associated data.

movie header atom A QT atom that specifies the characteristics of an entire QuickTime movie.

movie poster A single visual image representing a QuickTime movie. You specify a poster as a point in time in the movie and specify the tracks that are to be used to constitute the poster image.

movie preview A short dynamic representation of a QuickTime movie. Movie previews typically last no more than 3 to 5 seconds, and they should give the user some idea of what the movie contains. You define a movie preview by specifying its start time, its duration, and its tracks.

movie resource One of several data structures that provide the medium of exchange for movie data between applications on a Macintosh computer and between computers, even computers of different types.

movie sprite A sprite that lives in a sprite track and acts in a movie. See also sprite track.

MPEG-4 An ISO standard (based on the QuickTime file format) that supports video and audio streaming. See also streaming.

music One of the QuickTime media types, in which sequences of sounds and tones are generated.

2012-08-14 | Copyright © 2004, 2012 Apple Inc. All Rights Reserved.

National Television System Committee (NTSC) Refers to the color-encoding method adopted by the committee in 1953. This standard was the first monochrome-compatible, simultaneous color transmission system used for public broadcasting. This method is used widely in the United States.

node Either a panorama or an object in a QuickTime VR movie.

NTSC See National Television System Committee.

object track A track in a QuickTime VR movie that contains a set of views of a VR object.

offset-binary encoding A method of digitally encoding sound that represents the range of amplitude values as an unsigned number, with the midpoint of the range representing silence. For example, an 8-bit sound sample stored in offset-binary format would contain sample values ranging from 0 to 255, with a value of 128 specifying silence (no amplitude). Samples in Macintosh sound resources are stored in offset-binary form. Compare twos-complement encoding.

PAL See Phase Alternation Line (PAL).

packet For uncompressed audio, a sample from a single channel. For compressed audio, this field has no real meaning; by convention, it is treated as 1/number-of-channels.

panorama A structure of QuickTime VR data that forms a virtual-world environment within which the user can navigate.

panorama track A track in a QuickTime VR movie that contains a panorama.

parent atom A QT atom that contains other QT atoms, which are its child atoms. See also child atom.

Phase Alternation Line (PAL) A color-encoding system used widely in Europe, in which one of the subcarrier phases derived from the color burst is inverted in phase from one line to the next. This technique minimizes hue errors that may result during color video transmission. Sometimes called Phase Alternating Line.

playback quality A relative measure of the fidelity of a track in a QuickTime movie. You can control the playback (or language) quality of a movie during movie playback. QuickTime chooses tracks from alternate tracks that most closely correspond to the display quality desired. See also alternate track.

poster A frame shot from a movie, used to represent its content to the user.

preferred rate The default playback rate for a QuickTime movie.

preferred volume The default sound volume for a QuickTime movie.

preview A short, potentially dynamic, visual representation of the contents of a file. The Standard File Package can use previews in file dialog boxes to give the user a visual cue about a file's contents. See also file preview.

preview atom An atom of type 'pnot', which can appear in a QuickTime file to contain a movie's file preview.

profile atom An atom of type 'prfl', which summarizes the features of a movie or track.

property Information about a sprite that describes its location or appearance. One sprite property is its image, the original bitmapped graphic of the sprite.

Copyright © 2004, 2012 Apple Inc. All Rights Reserved.

PCM Pulse-code modulation (PCM) is a method used to digitally represent sampled analog signals (typically audio related in QuickTime references). See also LPCM.

QT atom A QuickTime atom that contains other atoms, possibly including other QT atoms and classic atoms. A data reference atom is an example of a QT atom. Compare classic atom.

QTMA (QuickTime Music Architecture) The part of QuickTime that lets other code create and manipulate music tracks in movies.

QTVR track A track in a QuickTime movie that maintains a list of VR nodes.

QuickDraw The original Mac OS two-dimensional drawing software, used by QuickTime.

QuickTime A set of Macintosh system extensions or a Windows dynamic-link library that other code can use to create and manipulate time-based data.

QuickTime VR A QuickTime media type that lets users interactively explore and examine photorealistic three-dimensional virtual worlds. QuickTime VR data structures are also called panoramas.

rate A value that specifies the pace at which time passes for a time base. A time base's rate is multiplied by the time scale to obtain the number of time units that pass per second. For example, consider a time base that operates in a time coordinate system that has a time scale of 60. If that time base has a rate of 1, 60 time units are processed per second. If the rate is set to 1/2, 30 time units pass per second. If the rate is 2, 120 time units pass per second. See also time base and time unit.

resource In Macintosh programming, an entity in a file or in memory that may contain executable code or a description of a user interface item. Resources are loaded as needed by a resource manager, and are identified by their type and ID number.

resource fork In a legacy Macintosh file, the section that contains resources. The use of the resource fork for storage of QuickTime media is deprecated in the QuickTime file format.

root atom The largest atom container in a hierarchy, with atom type 'sean'.

sample A single element of a sequence of time-ordered data.

sample format The format of data samples in a track, such as a sprite track.

sample number A number that identifies the sample with data for a specified time.

SECAM (Systeme Electronique Couleur avec Memoire) Sequential Color With Memory; refers to a color-encoding system in which the red and blue color-difference information is transmitted on alternate lines, requiring a one-line memory in order to decode green information.

skip atom An atom of type 'skip', which you can include in a QuickTime file as a placeholder for unused space.

SMPTE Acronym for Society of Motion Picture and Television Engineers, an organization that sets video and movie technical standards.

sound track The audio for a QuickTime file, which is stored in a track with a media handler type of `'soun'`.

2012-08-14 | Copyright © 2004, 2012 Apple Inc. All Rights Reserved.

sprite An animated image that is managed by QuickTime. A sprite is defined once and is then animated by commands that change its position or appearance.

sprite track A movie track populated by movie sprites.

streaming Delivery of video or audio data over a network in real time, to support applications such as videophone and video conferencing. See MPEG-4.

string atom An atom in VR media that contains text.

subtitle track The subtitle text for a QuickTime movie, which is typically a translation from the language of the sound track into a different language and which is stored in a track with a media handler type of `'sbtl'`.

SWF files Files that contain Flash data. See Flash.

sync sample A sample that does not rely on preceding frames for content. See also key frame.

Systeme Electronique Couleur avec Memoire See SECAM.

temporal compression Image compression that is performed between frames in a sequence. This compression technique takes advantage of redundancy between adjacent frames in a sequence to reduce the amount of data that is required to accurately represent each frame in the sequence. Sequences that have been temporally compressed typically contain key frames at regular intervals.

text track General-purpose text that displays in a QuickTime movie, which is represented as an atom of type 'text'.

thumbnail picture A picture that can be created from an existing image that is stored as a pixel map, a picture, or a picture file. A thumbnail picture is useful for creating small representative images of a source image and in previews for files that contain image data.

time base A set of values that define the time basis for an entity, such as a QuickTime movie. A time base consists of a time coordinate system (that is, a time scale and a duration) along with a rate value. The rate value specifies the speed with which time passes for the time base.

time-based data Data that changes or interacts with the user along a time dimension. QuickTime is designed to handle time-based data.

timecode media A media of type 'tmcd' that is used to store timecode data.

timecode track A movie track that stores external timing information, such as SMPTE time codes.

time coordinate system A set of values that defines the context for a time base. A time coordinate system consists of a time scale and a duration. Together, these values define the coordinate system in which a time value or a time base has meaning.

time scale The number of time units that pass per second in a time coordinate system. A time coordinate system that measures time in sixtieths of a second, for example, has a time scale of 60.

time unit The basic unit of measure for time in a time coordinate system. The value of the time unit for a time coordinate system is represented by the formula (1/time scale) seconds. A time coordinate system that has a time scale of 60 measures time in terms of sixtieths of a second.

time value A value that specifies a number of time units in a time coordinate system. A time value may contain information about a point in time or about a duration.

2012-08-14 | Copyright © 2004, 2012 Apple Inc. All Rights Reserved.

track A Movie Toolbox data structure that represents a single data stream in a QuickTime movie. A movie may contain one or more tracks. Each track is independent of other tracks in the movie and represents its own data stream. Each track has a corresponding media, which describes the data for the track.

track boundary region A region that describes the area occupied by a track in the track's coordinate system. QuickTime obtains this region by applying the track clipping region and the track matte to the visual image contained in the track rectangle.

track clipping region The clipping region of a track in the track's coordinate system. QuickTime applies the track's clipping region and the track matte to the image contained in the track rectangle to obtain the track boundary region. Only that portion of the track that lies in the track boundary region is then transformed into an image in the movie coordinate system.

track header atom A QT atom that specifies the characteristics of a track in a QuickTime movie.

track height The height, in pixels, of the track rectangle.

track input map A structure of QT atoms that specifies how secondary data for a track is to be interpreted (clipping, blending, etc.).

track load settings Information that specifies how and when a track is to be preloaded before running in a movie.

track matte A pixel map that defines the blending of track visual data. The value of each pixel in the pixel map governs the relative intensity of the track data for the corresponding pixel in the result image. QuickTime applies the track matte, along with the track clipping region, to the image contained in the track rectangle to obtain the track boundary region. See track matte, track rectangle, and track boundary region.

track movie boundary region A region that describes the area occupied by a track in the movie coordinate system, before the movie has been clipped by the movie clipping region. The movie boundary region is built up from the track movie boundary regions for each of the movie's tracks.

track offset The blank space that represents the intervening time between the beginning of a movie and the beginning of a track's data. In an audio track, the blank space translates to silence; in a video track, the blank space generates no visual image. All of the tracks in a movie use the movie's time coordinate system. That is, the movie's time scale defines the basic time unit for each of the movie's tracks. Each track begins at the beginning of the movie, but the track's data might not begin until some time value other than 0.

track reference A data structure that defines the relation between movie tracks, such as the relation between a timecode track and other tracks. See timecode track.

track rectangle A rectangle that completely encloses the visual representation of a track in a QuickTime movie. The width of this rectangle in pixels is referred to as the track width; the height, as the track height.

track width The width, in pixels, of the track rectangle.

transformation matrix A 3-by-3 matrix that defines how to map points from one coordinate space into another coordinate space.

tween data The data in a tween track, such as interpolation values.

2012-08-14 | Copyright © 2004, 2012 Apple Inc. All Rights Reserved.

tween track A modifier track that performs a specific kind of tweening, such as path-to-matrix rotation.

tweening A process interpolating new data between given values in conformance to an algorithm. It is an efficient way to expand or smooth a movie's presentation between its actual frames.

twos-complement encoding A system for digitally encoding sound that stores the amplitude values as a signed number—silence is represented by a sample with a value of 0. For example, with 8-bit sound samples, twos-complement values would range from –128 to 127, with 0 meaning silence. The Audio Interchange File Format (AIFF) stores samples in twos-complement form. Compare offset-binary encoding.

URL The address of a website.

user data Auxiliary data that your application can store in a QuickTime movie, track, or media structure. The user data is stored in a user data list; items in the list are referred to as user data items. Examples of user data include a copyright, date of creation, name of a movie's director, and special hardware and software requirements. See also user data list, user data item

user data item A single element in a user data list, such as a modification date or copyright notice.

user data list The collection of user data for a QuickTime movie, track, or media. Each element in the user data list is called a user data item.

VR (virtual reality) See QuickTime VR.

Wired Sprite A sprite such as a clickable button that has wired actions associated with it.

2012-08-14 | Copyright © 2004, 2012 Apple Inc. All Rights Reserved.

Apple Inc.
Copyright © 2004, 2012 Apple Inc.
All rights reserved.

No part of this publication may be reproduced, stored in a retrieval system, or transmitted, in any form or by any means, mechanical, electronic, photocopying, recording, or otherwise, without prior written permission of Apple Inc., with the following exceptions: Any person is hereby authorized to store documentation on a single computer for personal use only and to print copies of documentation for personal use provided that the documentation contains Apple's copyright notice.

No licenses, express or implied, are granted with respect to any of the technology described in this document. Apple retains all intellectual property rights associated with the technology described in this document. This document is intended to assist application developers to develop applications only for Apple-labeled computers.

Apple Inc.
1 Infinite Loop
Cupertino, CA 95014
408-996-1010

Apple, the Apple logo, Aperture, Chicago, ColorSync, Mac, Mac OS, Macintosh, OS X, QuickDraw, and QuickTime are trademarks of Apple Inc., registered in the U.S. and other countries.

Intel and Intel Core are registered trademarks of Intel Corporation or its subsidiaries in the United States and other countries.

Java is a registered trademark of Oracle and/or its affiliates.

PowerPC and the PowerPC logo are trademarks of International Business Machines Corporation, used under license therefrom.

Times is a registered trademark of Heidelberger Druckmaschinen AG, available from Linotype Library GmbH.

Even though Apple has reviewed this document, APPLE MAKES NO WARRANTY OR REPRESENTATION, EITHER EXPRESS OR IMPLIED, WITH RESPECT TO THIS DOCUMENT, ITS QUALITY, ACCURACY, MERCHANTABILITY, OR FITNESS FOR A PARTICULAR PURPOSE. AS A RESULT, THIS DOCUMENT IS PROVIDED "AS IS," AND YOU, THE READER, ARE ASSUMING THE ENTIRE RISK AS TO ITS QUALITY AND ACCURACY.

IN NO EVENT WILL APPLE BE LIABLE FOR DIRECT, INDIRECT, SPECIAL, INCIDENTAL, OR CONSEQUENTIAL DAMAGES RESULTING FROM ANY DEFECT OR INACCURACY IN THIS DOCUMENT, even if advised of the possibility of such damages.

THE WARRANTY AND REMEDIES SET FORTH ABOVE ARE EXCLUSIVE AND IN LIEU OF ALL OTHERS, ORAL OR WRITTEN, EXPRESS OR IMPLIED. No Apple dealer, agent, or employee is authorized to make any modification, extension, or addition to this warranty.

Some states do not allow the exclusion or limitation of implied warranties or liability for incidental or consequential damages, so the above limitation or exclusion may not apply to you. This warranty gives you specific legal rights, and you may also have other rights which vary from state to state.
